# Comprehensive exploration of chemical space using trisubstituted carboranes

**DOI:** 10.1038/s41598-021-03459-6

**Published:** 2021-12-16

**Authors:** Yasunobu Asawa, Saki Hatsuzawa, Atsushi Yoshimori, Kentaro Yamada, Akira Katoh, Hiroyuki Kouji, Hiroyuki Nakamura

**Affiliations:** 1 School of Life Science and Technology, Tokyo Institute of Technology, 4259, Nagatsuta-cho, Midori-ku, Yokohama, 226-8503 Japan; 2 School of Science, Kitasato University, 1-15-1, Kitazato, Minami-ku, Sagamihara-shi, Kanagawa 252-0373 Japan; 3 Institute for Theoretical Medicine, Inc., 26-1, Muraoka-Higashi 2-Chome, Fujisawa, Kanagawa 251-0012 Japan; 4 Faculty of Agriculture, Miyazaki University, 1-1, Gakuenkibanadai-Nishi, Miyazaki, 889-2192 Japan; 5 Faculty of Medicine, Oita University, 1-1, Idaigaoka, Hasama-machi, Yufu-City, Oita 879-5593 Japan; 6 Oita University Institute of Advanced Medicine, Inc., 17-20, Higashi Kasuga-machi, Oita City, Oita 870-0037 Japan; 7 Laboratory for Chemistry and Life Science, Institute of Innovative Research, Tokyo Institute of Technology, 4259, Nagatsuta-cho, Midori-ku, Yokohama, Kanagawa 226-8503 Japan

**Keywords:** Chemical libraries, Drug discovery and development, Organometallic chemistry, Diversity-oriented synthesis

## Abstract

A total of 42 trisubstituted carboranes categorised into five scaffolds were systematically designed and synthesized by exploiting the different reactivities of the twelve vertices of *o-*, *m*-, and *p*-carboranes to cover all directions in chemical space. Significant inhibitors of hypoxia inducible factor transcriptional activitay were mainly observed among scaffold V compounds (e.g., **Vi–m**, and **Vo**), whereas anti-rabies virus activity was observed among scaffold V (**Va–h**), scaffold II (**IIb–g**), and scaffold IV (**IVb**) compounds. The pharmacophore model predicted from compounds with scaffold V, which exhibited significant anti-rabies virus activity, agreed well with compounds **IIb–g** with scaffold II and compound IVb with scaffold IV. Normalized principal moment of inertia analysis indicated that carboranes with scaffolds I–V cover all regions in the chemical space. Furthermore, the first compounds shown to stimulate the proliferation of the rabies virus were found among scaffold V carboranes.

## Introduction

Biomolecules play an important role in maintaining living systems in a crowded biological environment. The structure of biomolecules is three-dimensional, and these molecules interact with each other to construct complex biological networks and systems that comprise robust and redundant functionalities. Dysfunctions of such networks and systems cause diseases, and drugs help restore these networks and systems correct physiological functions. Therefore, drugs need to work in the three-dimensional chemical space of biomolecular interactions. More complex molecules are deemed to have the ability to access a larger chemical space^1,2^, and natural products meet this complexity requirement due to their enormous structural diversity^3,4^. In fact, many small molecular drugs on the market are derived from natural products^5^. Lovering et al*.* suggested that the higher the fraction of sp^3^ carbon centres (Fsp^3^) in a small molecule, the higher the probability that the small molecule will make its way from drug discovery through clinical trials to becoming a drug^6–8^. Diversity-oriented synthesis (DOS), a concept for synthesis to access structurally complex and diverse compounds^9^, has been developed with the goal of producing three-dimensional complex drug-like compounds and chemical modulators^10–14^. However, the systematic design of three-dimensional divergent molecules as a way to explore the chemical space has not been fully investigated yet^8^.

**Figure 1:**
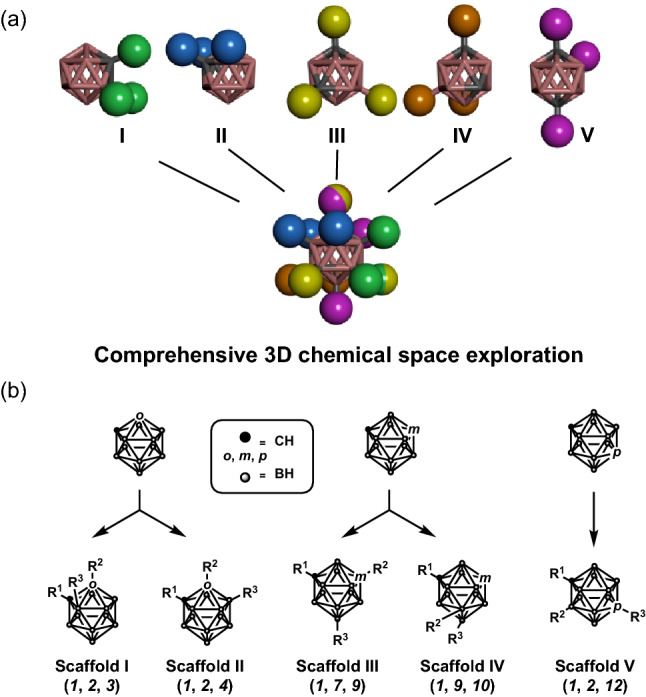
(**a**) The five types of trisubstituted carboranes that can cover all directions in a chemical space. (**b**) Synthetic strategy for the production of the described trisubstituted carboranes.

We focused on the icosahedral structure of carborane (dicarba-*closo*-dodecaborane). Notably, carborane consists of two carbon atoms, ten boron atoms, and ten hydrogen atoms, and three different isomers of this species are possible, depending on the relative position of two carbons: *ortho* (1,2), *meta* (1,7), and *para* (1,12)^15,16^. Given its three-dimensional hydrophobic features, over the past two decades, carborane has attracted much attention as a hydrophobic pharmacophore for drug discovery^17–20^. Indeed, by introducing substituents on the carbon centres of carborane, various promising drug candidates have been synthesised, including 17β-estrogen mimics^17,21^, antifolates^22^, HSP60 inhibitors^23,24^, COX-2 inhibitors^25,26^, vitamin D receptor ligands^27,28^, and nicotinamide phosphoribosyl-tranferase inhibitors^29,30^. We assumed that, by introducing three substituents at arbitrary carbon positions on carborane, it would be possible to spatially cover all directions. In fact, five types of trisubstituted carboranes can be designed that satisfy all directions in a chemical space, as can be evinced from Fig. 1a. Since the twelve vertices of carborane have mutually different reactivities, it should be possible to design different synthetic approaches to producing the various trisubstituted carboranes^31,32^. Although a limited number of trisubstituted carboranes has been reported^31^, the systematic design of trisubstituents undertaken to achieve the DOS for the development of drug candidates has not yet been investigated. In the present study, we synthesised five types of trisubstituted carboranes: scaffolds I and II from *o*-carborane, scaffolds III and IV from *m*-carborane, and scaffold V from *p*-carboranes (Fig. 1b).

## Results and discussion

### Synthesis of trisubstituted carboranes

We developed tricyclic three-dimensional scaffolds that are rich in sp^3^-hybridized carbon centres to access unexplored chemical spaces, and we used these scaffolds to design peptidomimetic molecules^33–35^. The α-helix is one of the important elements of a protein’s secondary structure that affords the construction of three-dimensional tertiary structures of proteins. It is also a structure found in many protein–protein interactions (PPIs)^36^, and the α-helix plays an important role in biological networks^37–39^. Although PPIs have been attracting the attention of researchers as drug targets, their contact surfaces are relatively large (750–1500 Å^2^)^36,40^ compared with their counterparts between proteins and small molecules (~ 300 Å^2^)^41^. Therefore, the development of small molecule inhibitors targeting PPIs remains a challenging proposition. Recently, the FDA approved Venetoclax (ABT-199)^42^ as a first-in-class small molecule-based PPI inhibitor targeting the Bcl-2/Bax interaction^43,44^. The X-ray co-crystal structure of Bcl-x_L_ with ABT-737 revealed that ABT-737 binds in the same cleft as the Bax native helix at the Bcl-x_L_/Bax interface^37,45^. The protein–protein interfaces usually contain crucial residues for the PPI called ‘hot spots’^46^, which are often composed of hydrophobic amino acid residues^47,48^. In this study, benzyl (Bn) and isobutyl (^*i*^Bu) groups were introduced into the carborane scaffolds as mimics of hydrophobic amino acid residues phenylalanine and leucine, respectively^34^.

**Scheme 1:**
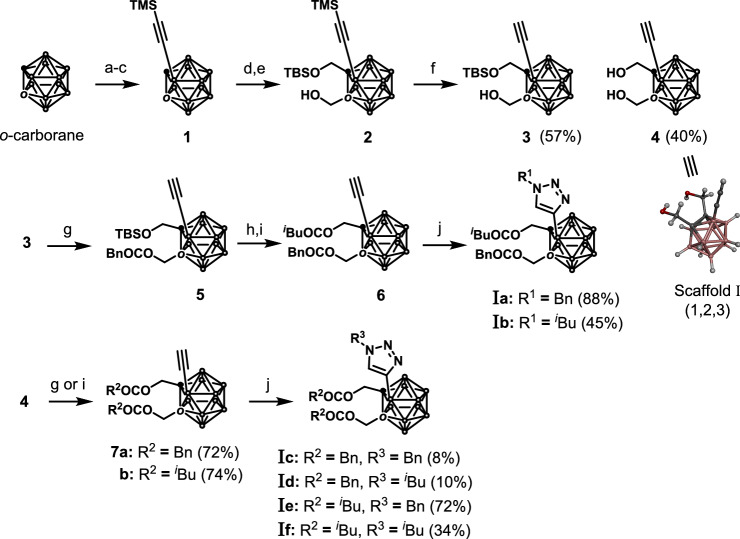
Synthesis of 1,2,3-trisubstituted *o*-carboranes (scaffold I). Reaction conditions: (a) KF, ethanol, reflux; then, HCl, (CH_3_)_3_NHCl, r.t., 87%; (b) ^*n*^BuLi, diethyl ether, r.t.; then, BI_3_, toluene, r.t., 81%; (c) TMSC_2_MgBr, Pd(PPh_3_)_2_Cl_2_, THF, 80 °C, 70%; (d) (i) ^*n*^BuLi, (CHO)_n_, THF, − 78 °C to 0 °C; (ii) TBSOTf, 2,6-lutidine, CH_2_Cl_2_, 0 °C to r.t., 20% in two steps; (e) ^*n*^BuLi, (CHO)_n_, THF, − 78 °C to 0 °C, 20%; (f) K_2_CO_3_, methanol, r.t.; (g) BnCOCl, Et_3_N, CH_2_Cl_2_, r.t., 81%; (h) HCl-dioxane, CH_2_Cl_2_, r.t., 90%; (i) ^*i*^BuCOCl, Et_3_N, CH_2_Cl_2_, r.t., quant.; (j) BnN_3_ or ^*i*^BuN_3_. CuI, Na-ascorbate, DMF/H_2_O, r.t.; TMS: trimethylsilyl; TBS: *tert*-butyldimethylsilyl; Bn: benzyl; ^*i*^Bu: isobutyl; DMF: dimethylformamide; Et: ethyl; PPh_3_: triphenylphosphine; r.t.: room temperature; OTf: triflate.

The approach to the synthesis of scaffold I (1,2,3-trisubstituted *o*-carboranes) implemented in this study is detailed in Scheme 1. We were concern that a direct amide bond to the o-carborane cluster could cause degradation via deboronation induced by the amide group^26^. Therefore, o-carborane was functionalized without introducing any amide bonds. Selective iodination at the 3-position of *o*-carborane was performed according to a literature procedure^49^, and the resulting B3-iodocarborane was subjected to the Kumada–Tamao–Corriu cross coupling to produce **1** in 70% yield^50^. After the lithiation on C1 of **1** with ^*n*^BuLi followed by the addition to paraformaldehyde, the resulting hydroxy group was protected with the *tert*-butyldimethylsilyl (TBS) group. The modification of another hydroxy methyl group on C2 achieved in the same manner described for C1 afforded compound **2**. Removing the trimethylsilyl (TMS) group of **2** with potassium carbonate produced a mixture of mono-alcohol **3** and di-alcohol **4** in 57% and 40% yields, respectively. Notably, the structure of **4** was unambiguously determined by X-ray structural analysis. Esterification of **3** with phenylacetyl chloride gave compound **5** in 46% yields over two steps. The TBS group was then removed, and the resulting alcohol was esterified with isovaleryl chloride to afford compound **6** in 90% yields over two steps. Finally, acetylene **6** was subjected to a Hüsgen cycloaddition reaction with benzyl azide or isobutyl azide in the presence of CuI and sodium ascorbate to obtain the trisubstituted carboranes **Ia** and **Ib** in 45% and 88% yields, respectively. On the other hand, esterification of two hydroxy groups on **4** with the identical substituents afforded **7a** and **7b** in 72% and 74% yields, respectively; subjecting **7a** and **7b** to the Hüsgen cycloaddition reaction with benzyl azide or isobutyl azide gave **Ic**–**f** in yields ranging between 8 and 72%. Thus, the synthesis of 1,2,3-trisubstituted carboranes with different substituents on the three tethers was achieved in scaffold I.

**Scheme 2:**
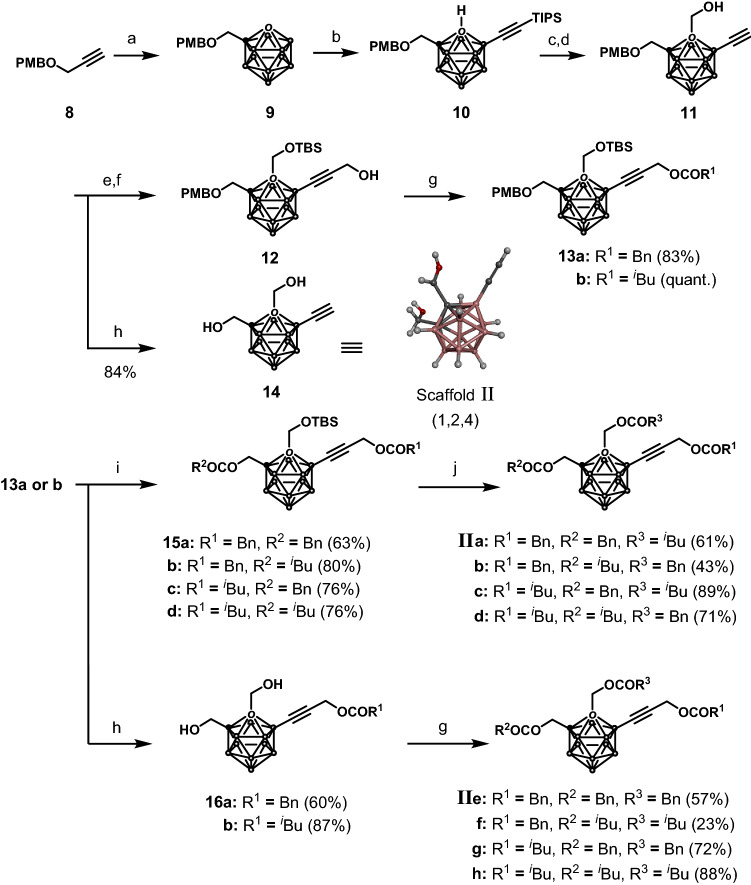
Synthesis of 1,2,4-trisubstituted *o*-carboranes (scaffold II). Reaction conditions: (a) B_10_H_14_, *N*,*N*-dimethylaniline, PhCl, 130 °C, MW, 79%; (b) (i) ^*n*^BuLi, CO_2_, diethyl ether, − 78 °C to r.t.; (ii) TIPSC≡CH, Pd(OAc)_2_, AgOAc, K_2_HPO_4_, toluene, 80 °C, 31% in two steps; (c) ^*n*^BuLi, (CHO)_n_, THF, − 78 °C to 0 °C, 80%; (d) TBAF, THF, 0 °C, quant.; (e) TBSOTf, 2,6-lutidine, CH_2_Cl_2_, 0 °C to r.t., 98%; (f) ^*n*^BuLi, (CHO)_n_, THF, − 78 °C to 0 °C, 79%; (g) BnCOCl or ^*i*^BuCOCl, Et_3_N, CH_2_Cl_2_, r.t.; (h) DDQ, NaH_2_PO_4_, CH_2_Cl_2_·H_2_O, r.t.; (i) i—DDQ, NaH_2_PO_4_, CH_2_Cl_2_·H_2_O, r.t., ii—BnCOCl or ^*i*^BuCOCl, Et_3_N, CH_2_Cl_2_, r.t.; (j) i—HCl-dioxane, CH_2_Cl_2_, r.t.; ii—BnCOCl or ^*i*^BuCOCl, Et_3_N, CH_2_Cl_2_, r.t.. Bn: benzyl; Bu: butyl; Et: ethyl; ^*i*^Bu: isobutyl; OAc: acetate; Ph: phenyl; OTf: triflate; PMB: *p*-methoxybenzyl; MW: microwave irradiation; TIPS: triisopropylsilyl; TBAF: tetrabutylammonium fluoride; TBS: *tert*-butyldimethylsilyl; DDQ: 2,3-dichloro-5,6-dicyano-*p*-benzoquinone; r.t.: room temperature.

Next, the synthesis of scaffold II (1,2,4-trisubstituted *o*-carboranes) was carried out according to the approach detailed in Scheme 2. Propargyl *p*-methoxybenzyl (PMB) ether (**8**) was made to react with decaborane in the presence of *N*,*N*-dimethylaniline under microwave (MW) irradiation in chlorobenzene to obtain the 1-substituted *o*-carborane **9** in 79% yield^51^. A carboxylic acid moiety was then introduced into the C2-position of **9** using ^*n*^BuLi and carbon dioxide, which functioned as a directing group to afford the selective alkynylation of the B4-position via an approach developed by Xie and co-workers, producing compound **10** in 31% yield over two steps^31^. Notably, the carboxylic acid intermediate had to be immediately subjected to the alkynylation reaction to avoid the deprotection of the PMB group induced by the acidity of the intermediate. After introducing a hydroxy methyl group into the C2-position of compound **10** via an approach similar to that of step d in Scheme 1, the triisopropylsilyl (TIPS) group of the alkyne moiety was removed. The TBS protection of the hydroxy group of compound **11**, followed by the introduction of a hydroxy methyl group into the terminal alkyne, afforded compound **12**. The hydroxy group on compound **12** was converted into either a Bn group (**13a**, 83% yield) or an ^*i*^Bu group (**13b**, quant.). On the other hand, removal of the PMB group of compound **11** afforded dialcohol **14**, whose structure was determined by X-ray structural analysis. (It should be noted that good quality crystals even made several measurements were not obtained due to the unnecessary reflections.). The PMB group of compound **13** was selectively removed using 2,3-dichloro-5,6-dicyano-*p*-benzoquinone (DDQ), and the subsequent esterification of the deprotected derivatives of compounds **13a** and **13b** with phenylacetyl chloride or isovaleryl chloride afforded compounds **15a**–**d**. After removing the TBS group, Bn and ^*i*^Bu groups were introduced into the C2-position of **15** to obtain the 1,2,4-trisubstituted carboranes **IIa**–**d** in 43–89% yields. Furthermore, compounds **13a** and **13b** were treated with HCl in dioxane, and Bn and ^*i*^Bu groups were introduced into the resulting diols **16** to obtain compounds **IIe**–**h** in 23–88% yields.

**Scheme 3:**
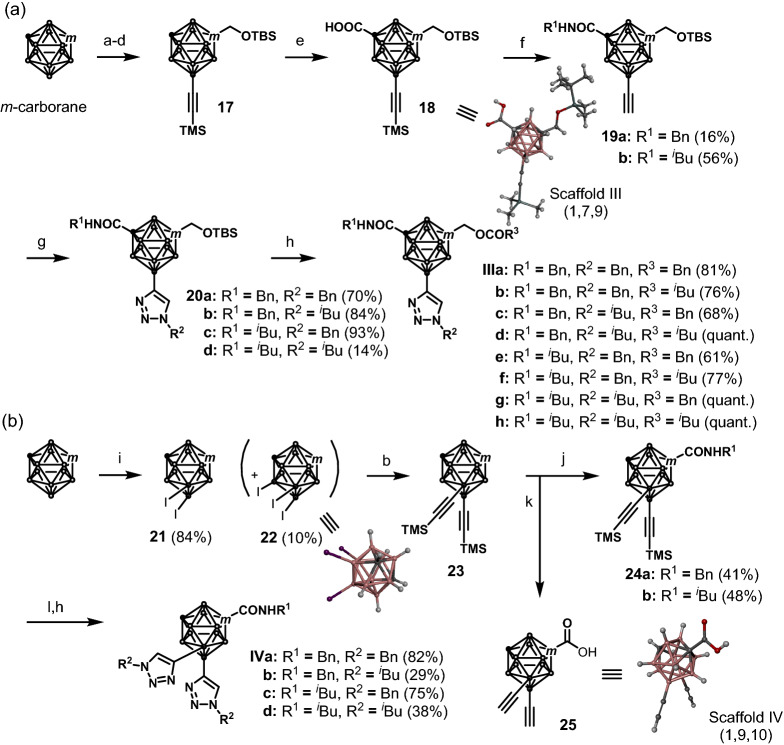
Syntheses of 1,7,9-substituted carboranes (scaffold III) (a) and 1,9,10-substituted carboranes (scaffold IV) (b). Reaction conditions: (a) I_2_, AlCl_3,_ CH_2_Cl_2_, r.t., 96%; (b) TMSC_2_MgBr, Pd(PPh_3_)_2_Cl_2,_ THF, 80 °C, 96%; (c) ^*n*^BuLi, (CHO)_n_, THF, − 78 °C to 0 °C, 46%; (d) TBSOTf, 2,6-lutidine, CH_2_Cl_2_, 0 °C to r.t. 99%; (e) ^*n*^BuLi, CO_2_, THF, − 78 °C to r.t., 92%; (f) i—EDCI, HOBt, DIEA, BnNH_2,_ or ^*i*^BuNH_2_, THF, r.t.; ii—K_2_CO_3_, methanol, r.t.; (g) BnN_3_ or ^*i*^BuN_3_, CuI, Na-ascorbate, DMF/H_2_O, r.t.; (h) i—HCl-dioxane, CH_2_Cl_2_, r.t.; ii—BnCOCl or ^*i*^BuCOCl, Et_3_N, CH_2_Cl_2_, r.t.; (i) I_2_, HNO_3_, H_2_SO_4,_ AcOH, 80 °C; (j) i—^*n*^BuLi, CO_2_, THF, − 78 °C to r.t.; ii—EDCI, HOBt, DIEA, BnNH_2, or_^*i*^BuNH_2_, THF, r.t.; (k) i—^*n*^BuLi, CO_2_, THF, − 78 °C to r.t.; ii—K_2_CO_3_, methanol, r.t., 30% in two steps; (l) K_2_CO_3_, MeOH, r.t.. AcO: acetate; Bn: benzyl; Bu: butyl; Et: ethyl; ^*i*^Bu: isobutyl; Ph: phenyl; OTf: triflate; r.t.: room temperature; TBS: *tert*-butyldimethylsilyl; TMS: trimethylsilyl; EDCI: 1-(3-dimethylaminopropyl)-3-ethylcarbodiimide hydrochloride; HOBt: 1-hydroxybenzotriazole monohydrate; DIEA: *N*,*N*-diisopropylethylamine.

Next, we synthesized 1,7,9-substituted carboranes (scaffold III) and 1,9,10-substituted carboranes (scaffold IV) using *m*-carborane as the starting building block (Scheme 3). Selective electrophilic iodination at the B9-position of *m*-carborane followed by the Kumada–Tamao–Corriu cross coupling were performed implementing a literature procedure^50^. A hydroxy methyl group was then introduced into the C1 position of the resulting 9-(trimethylsilyletynyl)-*m*-carborane in a similar manner to that described for the synthesis of compounds **1** to **2** (see Scheme 1), followed by TBS protection to give the 1,9-disubstituted *m*-carborane **17** in 42% yield over four steps. Afterwards, a carboxylic acid group was introduced into the C7-position of compound **17** using ^*n*^BuLi and carbon dioxide, and the resulting carboxylic acid **18** was subjected to amidation with either benzyl amine or isobutyl amine in the presence of 1-(3-dimethylaminopropyl)-3-ethylcarbodiimide hydrochloride (EDCI), 1-hydroxybenzotriazole monohydrate (HOBt), and *N*,*N*-diisopropylethylamine (DIEA); the subsequent removal of the TMS group afforded compounds **19a** and **19b** in 16% and 55% yields over two steps, respectively. These two compounds were then subjected to Hüsgen cycloaddition reactions with benzyl azide or isobutyl azide implementing the approach detailed in Scheme 1 to give compounds **20a**–**d** in 14–93% yields. Finally, removal of the TBS group followed by an esterification process afforded 1,7,9-substituted carboranes **IIIa**–**h** in 61% to quantitative yields over two steps. Scaffold IV was obtained according to the synthetic procedure depicted in Scheme 3b. The selective electrophilic diiodination at the 9,10-positions of *m*-carborane was achieved under microwave irradiation to afford 9,10-diiodo-*m*-carborane **21** in 84% yield. Surprisingly, 5,9,10-triiodo-*m*-carborane **22**, which has not been reported previously, was obtained as a by-product in 10% yield.

The Kumada–Tamao–Corriu cross coupling between **21** and trimethylsilylacetylene was performed under reaction conditions similar to those described in Scheme 2 to give 9,10-bis(trimethylsilyletynyl)-*m*-carborane **23** in 94% yield. After introducing a carboxylic acid into the C1-position of **23**, the resulting carboxylic acid was subjected to amidation reactions with benzyl amine or isobutyl amine using COMU^52^ to afford compounds **24a** and **24b** in 41% and 48% yields over two steps, respectively. After removal of the TMS group, the Hüsgen cycloaddition reaction was carried out to obtain compounds **IVa**–**d** in 29–82% yields over the two steps. Notably, although we were able to synthesize 1,9,10-trisubstituted carboranes, we found it difficult to introduce different substituents at the 9,10-positions of scaffold IV. The structures of **18** (scaffold III), 5,9,10-triiodo-*m*-carborane **22**, and compound **25** (scaffold IV) were determined by X-ray structural analysis.

**Scheme 4:**
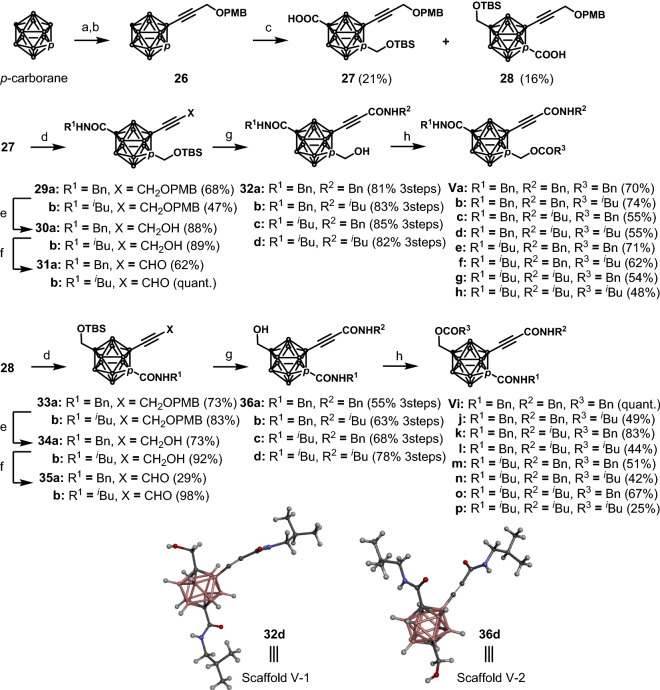
Synthesis of scaffold V. Reaction conditions: (a) I_2_, HNO_3_, H_2_SO_4,_ AcOH, 80 °C, 99%; (b) propargyl PMB ether, PdCl_2_(PPh_3_)_2_, CuI, toluene, piperidine, 80 °C, 49%; (c) i—^*n*^BuLi, (CHO)_n_, THF, − 78 °C to 0 °C; ii—TBSOTf, 2,6-lutidine, CH_2_Cl_2_, 0 °C to r.t.; iii—^*n*^BuLi, CO_2_, THF, − 78 °C to r.t.; (d) EDCI, HOBt, DIEA, BnNH_2_ or ^*i*^BuNH_2_, THF, r.t.; (e) DDQ, NaH_2_PO_4_, CH_2_Cl_2_, H_2_O, r.t.; (f) Dess–Martin periodinane, CH_2_Cl_2_, r.t.; (g) i—NaClO_2_, NaH_2_PO_4_, 2-methyl-2-butene, acetone/H_2_O, r.t.; ii—EDCI, HOBt, DIEA, BnNH_2_ or ^*i*^BuNH_2_, THF, r.t.; HCl-dioxane, CH_2_Cl_2_, r.t.; (h) BnCOCl or ^*i*^BuCOCl, Et_3_N, CH_2_Cl_2_, r.t.. AcO: acetate; Bn: benzyl; Bu: butyl; Et: ethyl; ^*i*^Bu: isobutyl; Ph: phenyl; DDQ: 2,3-dichloro-5,6-dicyano-*p*-benzoquinone; PMB: *p*-methoxybenzyl TBS: *tert*-butyldimethylsilyl; r.t.: room temperature; EDCI: 1-(3-dimethylaminopropyl)-3-ethylcarbodiimide hydrochloride; HOBt: 1-hydroxybenzotriazole monohydrate; DIEA: *N*,*N*-diisopropylethylamine.

Finally, the synthesis of 1,2,12-substituted carborane was carried out using *p*-carborane (Scheme 4). The electrophilic iodination at the B2-position of *p*-carborane^50^ followed by the Sonogashira–Hagihara cross coupling with propargyl PMB ether produced B2-substituted carborane **26** in 49% yield over two steps. By introducing a hydroxymethyl group into **26** via the lithiation with ^*n*^BuLi followed by the addition to paraformaldehyde, 1,2-disubstituted and 1,7-disubstituted carboranes were generated as regioisomers. After a TBS group was introduced to protect the hydroxy group, a carboxylic acid group was introduced into another carbon atom; subsequently, a separation procedure was implemented using preparative HPLC to obtain the 1,7,12-trisubstituted carborane **27** and 1,2,12-trisubstituted carborane **28** in 21% and 16% yields, respectively, over three steps. The carboxylic acids **27** and **28** were subjected to amidation reactions with benzyl amine or isobutyl amine in a manner similar to that described in Scheme 3; subsequently, removal of the PMB group followed by the Dess–Martin oxidation produced aldehydes **31a**,**b** and **35a**,**b** in 15–75% yields over three steps. These aldehydes were then subjected to Pinnick–Kraus oxidations, and the resulting carboxylic acids were again subjected to amidation with benzyl amine or isobutyl amine followed by removal of the TBS group to afford **32a**–**d** and **36a**–**d** in 55–85% yields over three steps. Finally, esterification with phenylacetyl chloride against methanol gave compounds **Va**–**p** in 25% to quantitative yields. The absolute structures of the two isomers were confirmed by X-ray crystallography to be **32d** and **36d** (see Scheme 4).

### Biological evaluation of trisubstituted carborane library

In order to validate the biological activity of the thus synthesized trisubstituted carborane library, we performed cell-based assays to determine: hypoxia inducible factor (HIF)-1 transcriptional activity, anti-rabies virus activity, and anti-proliferative activity. The experiments designed to determine the prepared carboranes’ HIF-1 transcriptional activity were conducted using HeLa cells stably transfected with the hypoxic response element (HRE)-dependent luciferase reporter gene. The cells were incubated in the presence of the various compounds under hypoxic conditions, and fluorescence intensity was recorded to determine the trisubstituted carboranes’ IC_50_ values. The three leucine residues Leu818, Leu819, and Leu822 of the *C*-terminal helix (helix 3) of HIF-1α are involved in the PPI between HIF-1α and p300, which induces transcriptional activity. The anti-rabies virus activity of the synthesized compounds was investigated using N2a cells. N2a cells infected with a recombinant rabies virus expressing gaussia luciferase reporter were incubated in the presence of various carboranes. The IC_50_ values were determined by measuring the luciferase activity in the cell supernatant secreted extracellularly. In addition, since the reporter gene assay was affected by the carboranes’ cytotoxicity, cell viability toward both HeLa cells and N2a cells was also measured performing the MTT (3-[4,5-dimethylthiazol-2-yl]-2,5 diphenyl tetrazolium bromide) assay. The results of the said experiments are summarized in Table 1. Among the compounds synthesized, **Vi–m** and **Vo**, characterized by scaffold V, were found to significantly inhibit HIF-1 transcriptional activity (IC_50_ = 3.24–23.2 µM), and carboranes **IIb–g** characterized by scaffold II and carboranes **Va–h** characterized by scaffold V exhibited antiviral activity toward RABV (IC_50_ = 2.17–8.37 µM) and low cytotoxicity toward host cells (IC_50_ > 30 µM). Even though the same substituents had been introduced in the various molecules, the positions of the said substituents dramatically affected the carboranes’ biological activities (e.g., see the activities of **Vf** vs. **Vn**). This observation suggests that the protein can strictly recognise the geometrical configuration of substituents. In fact, **IId** with scaffold II and **Vh** with scaffold V exhibited similar antiviral activities, with IC_50_ values of 3.30 and 4.00 µM, respectively. Superimposing the structures of **IId** and **Vh** revealed that three isobutyl groups overlapped completely in each configuration, resulting in similar biological activity (Fig. S1). On the other hand, **IVb** with scaffold IV exhibited high antiviral activity (IC_50_ = 5.96 µM) and a moderate inhibition of HIF-1 transcriptional activity (IC_50_ = 17.4 µM). In order to confirm the possibility of the non-specific effect on the luciferase assay, we selected **Vj**, which showed the most significant inhibition of the HIF-1 transcriptional activity, and examined the direct luciferase inhibition test^53^. As shown in Fig. S2, **Vj** did not inhibit luciferase itself, indicating that inhibitions by compounds demonstrated in Table 1 were not caused by a non-specific effect on the luciferase assay. We also examined the effects of compounds **Vo**, **Vi**, **Vj**, and **Vk** on protein levels of HIF-1α and CA9 downstream of HIF-1α under hypoxia, and found that the protein levels of HIF-1α treated with the compounds were similar to that of control, whereas the suppression of the protein levels of CA9 were observed in the cells treated with the compounds, and the CA9 mRNA significantly suppressed by **Vk** under hypoxia. These results suggest that the compounds inhibit the HIF-1α-induced transcriptional pathway without affecting the HIF-1α protein expression (Fig. S3).

**Table 1:** Results of the evaluation of the biological activities of various members of the trisubstituted carborane compound library.

Scaffold	Compound	HIF-1^[a]^	RABV^[b]^	HeLa cells	N2a cells
	**Ia**	> 30	> 9	> 30	> 30
	**Ib**	> 30	> 9	> 30	> 30
	**Ic**	> 30	> 9	> 30	> 30
	**Id**	> 30	> 9	> 30	> 30
	**Ie**	> 30	> 9	> 30	> 30
	**If**	> 30	> 9	> 30	> 30
	**IIa**	> 30	> 9	> 30	> 30
	**IIb**	> 30	5.74	> 30	> 30
	**IIc**	> 30	6.87	> 30	> 30
	**IId**	> 30	3.30	> 30	> 30
	**IIe**	> 30	8.14	> 30	> 30
	**IIf**	> 30	5.78	> 30	> 30
	**IIg**	> 30	8.37	> 30	> 30
	**IIh**	> 30	> 9	> 30	> 30
	**IIIa**	> 30	> 9	> 30	> 30
	**IIIb**	26.3	> 9	> 30	> 30
	**IIIc**	> 30	> 9	> 30	> 30
	**IIId**	23.0	> 9	> 30	> 30
	**IIIe**	> 30	> 9	> 30	> 30
	**IIIf**	> 30	> 9	> 30	> 30
	**IIIg**	> 30	> 9	> 30	> 30
	**IIIh**	> 30	> 9	> 30	> 30
	**IVa**	> 30	> 9	> 30	> 30
	**IVb**	17.4	5.96	> 30	> 30
	**IVc**	> 30	> 30	> 30	> 30
	**IVd**	> 30	> 30	> 30	> 30
	**Va**	> 30	3.87	> 30	> 30
	**Vb**	> 30	2.17	> 30	> 30
	**Vc**	> 30	4.44	> 30	> 30
	**Vd**	> 30	3.95	> 30	> 30
	**Ve**	> 30	3.46	> 30	> 30
	**Vf**	> 30	2.56	> 30	> 30
	**Vg**	> 30	5.08	> 30	> 30
	**Vh**	> 30	4.00	> 30	> 30
	**Vi**	5.46	Activation	16.7	> 30
	**Vj**	3.24	Activation	> 30	22.6
	**Vk**	3.64	Activation	15.0	21.2
	**Vl**	18.1	Activation	21.7	25.2
	**Vm**	23.2	> 9	> 30	> 30
	**Vn**	> 30	> 9	> 30	> 30
	**Vo**	9.21	Activation	20.9	7.90
	**Vp**	> 30	Activation	> 30	8.00

The compound concentration required to inhibit the relative light units by 50% (IC_50_) was determined based on semi-logarithmic dose–response plots. All the samples were tested in triplicate. ^[a]^ YC-1 was used as positive control with IC50 of 0.17 µM. ^[b]^ Favipiravir was used as a control with IC_50_ of 38 µM.

**Figure 2:**
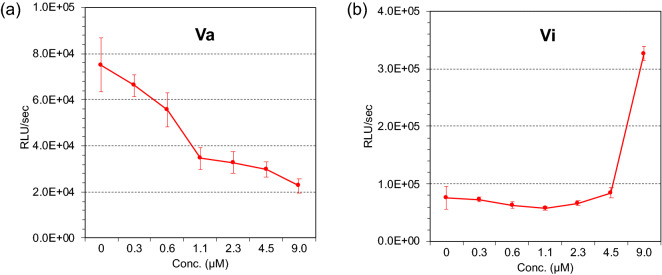
Compound concentration-dependent effects on the proliferation of the rabies virus: (**a**) Antiviral activity of Va and (**b**) activation of rabies virus proliferation by Vi. Compound concentration is reported on the x axis and fluorescent intensity on the y axis.

Furthermore, to our surprise, **Vi–l**, **Vo**, and **Vp** were found to stimulate the proliferation of the rabies virus. These compounds are the first to have ever been found to activate the said process. In fact, even a carborane with the same scaffold, compound **Va**, exhibited significant antivirus activity (IC_50_ = 3.87 µM), whereas **Vi** activated the proliferation of the rabies virus (Fig. 2). Notably compounds **Va** and **Vi** comprise the same substituents (Bn groups). A similar divergence of activity was observed between compounds **Va–h** and compounds **Vi–p**. In addition, the intracellular viral protein accumulation and the virus titer were measured using **Vp**. As shown in Fig. S4, the intracellular viral proteins treated with **Vp** was significantly increased without affecting the virus titer. Since viral the ribonucleoproteins (RNPs) are formed from the replicated genomic RNA and Rabies N proteins are released from the cell to infecting other cells^54^, these results suggest that **Vp** may have inhibited the formation of viral ribonucleoproteins, resulting in increased accumulation of viral proteins in the cell.

The compound concentration required to inhibit the relative light units by 50% (IC_50_) was determined based on semi-logarithmic dose–response plots. All the samples were tested in triplicate. [a] YC-1 was used as positive control with IC_50_ of 0.17 µM. [b] Favipiravir was used as a control with IC_50_ of 38 µM.

### Assessment of trisubstituted carboranes for comprehensive chemical space search

**Figure 3:**
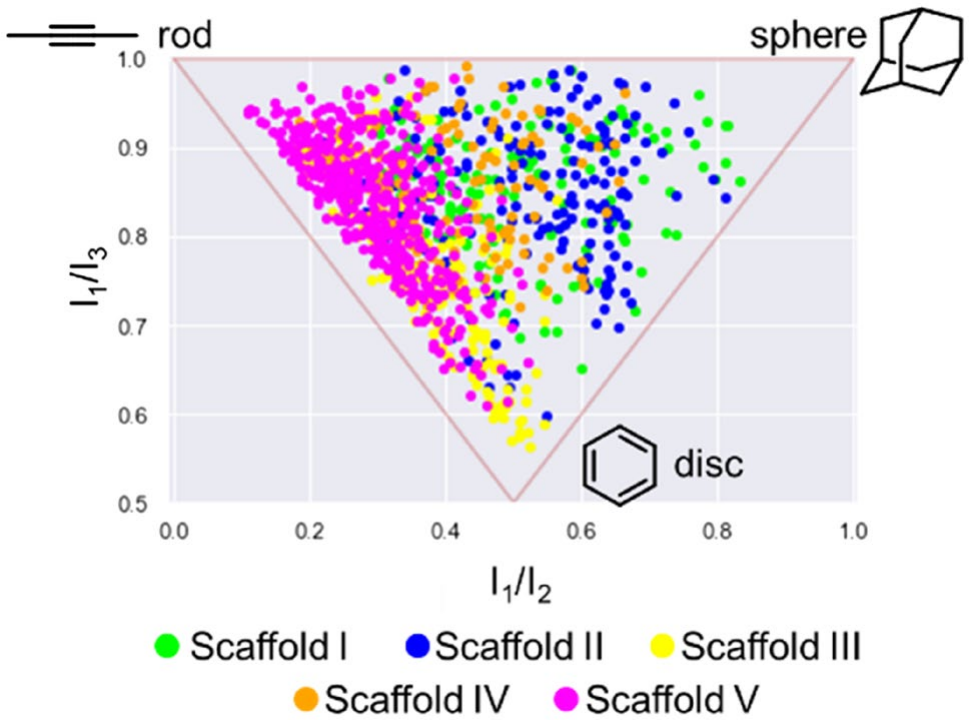
Normalized principal moment of inertia analysis of members of the synthesized trisubstituted carborane library; parameters I_1_/I_2_ and I_1_/I_3_ were defined by Sauer and Schwartz^53^. These values were calculated with the aid of RDKit^54^.

Finally, we investigated the morphologies of the herein synthesized compounds using the normalized principal moment of inertia (PMI)^55^, which is an approach commonly employed to assess the molecular shape of compound libraries in comparison to 2-butyne (rod), benzene (disc), and adamantane (sphere). For all compounds, 25 conformations were generated in each compound using the ‘iCon fast’ option of LigandScout 4.4, and the normalized PMI values calculated with the aid of the RDKit^56^ were plotted (Fig. 3; see also Fig. S5). Notably, a higher number of compounds characterized by scaffolds I and II (green and blue dots in Fig. 3) occupies the sphere-like shape section of the graph in Fig. 3 than compounds characterized by other scaffolds. On the other hand, compounds characterized by scaffold V tended to occupy the rod–disc area of the graph, as a result of the presence of the two substituents in para positions to each other aligned in a linear fashion. Scaffold III and IV-based compounds, derived from *m*-carborane, occupied the middle section of the morphology graph. The results of the PMI analysis indicate, therefore, that the herein synthesized carboranes characterized by scaffolds I–V cover all the regions of the described three-dimensional shape space.

## Conclusion

We successfully synthesized a total of 42 trisubstituted carboranes characterized by five basic of scaffolds that were found to occupy the entire rod–disc–sphere chemical morphology space; we achieved this goal by exploiting the different reactivities of the twelve vertices of *o-*, *m-*, and *p*-carboranes. The typical three-dimensional structures of each scaffold type of trisubstituted carboranes, **4**, **14**, **18**, **25**, **32d**, and **36d,** were unambiguously determined by X-ray structural analysis. The synthesized compounds were utilized in cell-based biological assays performed to determine the effects of various carboranes on HIF-1 transcriptional activity and anti-rabies virus activity. Compounds characterized by scaffold V (e.g., **Vi–m**, and **Vo**) were mainly observed to exhibit significant inhibition of HIF-1 transcriptional; by contrast, anti-rabies virus activity was observed not only for compounds with scaffold V (compounds **Va–h**) but also for carboranes with scaffold II (compounds **IIb–g**) and IV (compound **IVb**). The pharmacophore model predicted by the scaffold V carboranes, which exhibited significant anti-rabies virus activities, agreed well especially with compounds **IIb** and **IId** with scaffold **IVb** and compound **IVb** with scaffold IV. Furthermore, the first compounds to have ever been found to stimulate the proliferation of the rabies virus were identified in the present study, and they were determined to be scaffold V compounds. Therefore, we believe that our strategy for the systematic design of three-dimensionally divergent molecules based on five different types of trisubstituted carboranes has great potential to satisfy all directions in the chemical space and to afford the identification of novel biologically active molecules that have not been recognised by other drug discovery approaches.

## Experimental section

### Synthesis of trisubstituted carboranes

The synthetic procedures and characterization were provided in “Supplementary information S1”.

### X-ray crystallography

All data generated or analyzed were provided in “Supplementary information S1”. CCDC 2110331, 2110336–2110341.

### Cell culture

Human epithelioid cervical carcinoma (HeLa) cells were obtained from the Cell Resource Center for Biomedical Research, Institute of Development, Aging and Cancer, Tohoku University (Sendai, Japan). The cells were incubated with RPMI-1640 medium (FUJIFILM Wako Pure Chemical) containing 10% fetal bovine serum (Gibco; Thermo Fisher Scientific) and 1% peniciline/streptomycine (FUJIFILM Wako Pure Chemical). Cells were incubated in a cell incubator with 5% CO_2_ at 37 ℃. Neuro 2a (N2a) cells were incubated with D-MEM medium (FUJIFILM Wako Pure Chemical) containing 10% fetal bovine serum (Gibco; Thermo Fisher Scientific) and 1% peniciline/streptomycine (FUJIFILM Wako Pure Chemical). Cells were incubated in a cell incubator with 5% CO_2_ at 37 ℃.

### MTT assay

HeLa cells or N2a cells (5 × 10^3^ cells per well of a 96-well plate) were incubated under 5% CO_2_ at 37 ℃ in RPMI-1640 media (for HeLa cells) or D-MEM media (for N2a cells) containing 10% fetal bovine serum, 1% peniciline/streptomycine, and various concentrations of compound (10 mM in DMSO) for 72 h. After the incubation, 3′-(4,5-dimethylthiazole-2-yl)-2,5-diphenyltetrazolium bromide (MTT) in PBS (5 mg/mL, 10 μL) were added, and the cells were further incubated at 37 ℃ for 2 h. After removal of the medium, DMSO (100 μL) was added and the absorbance at 590 nm was measured with a microplate reader. The IC_50_ value was determined from semilogarithmic dose–response plots.

### Luciferase reporter gene assay for HIF transcriptional activity

HeLa cells transfected with hypoxia response element dependent firefly luciferase reporter construct (HRE-Luc) were seeded in a 96-well plate (2.5 × 10^4^ cells per a well) and incubated for 6 h under 5% CO_2_ at 37 ℃ 42 with RPMI-1640 media containing 10% fetal bovine serum, 1% peniciline/streptomycine. After the incubation, cells were treated with various concentration of compound (10 mM in DMSO) and incubated at the same condition for 1 h. Then the cells were incubated at normoxic condition (1% O_2_, 5% CO_2_, 37 ℃) for 12 h. At the same time, cells without compounds were incubated at hypoxic condition for 12 h. After removal of the media cells were washed with PBS and the luciferase reporter gene assay was performed with Luciferase Assay System (Promega, Madison, WI, USA) according to the manufacturer’s instructions. The compound concentration required to reduce relative luminescence units (RLU) by 50% (IC_50_) was determined from semilogarithmic dose response plots.

### Luciferase reporter gene assay for antiviral activity against rabies virus

The recombinant rabies virus strain 1088 expressing gaussia luciferase (1088/GLuc) was generated as described previously^4^. The virus titer was determined using focus assay as reported^5^ and expressed as focus forming units (FFU). N2a cells (4 × 10^4^ cells per well) and 1088/GLuc (4 × 10^2^ FFU per well) were prepared in E-MEM containing 10% fetal bovine serum and antibiotics, and the mixed solution was applied to a 96-well black plate with clear bottoms (Greiner). Subsequently, various concentrations of compounds were prepared in the medium and added to each well. The microplates were incubated at 37 ℃ under 5% CO_2_ for 3 days, and then, a luciferase reporter gene assay was performed using Pierce Gaussia Luciferase Glow Assay Kit (Thermo Fisher Scientific). The substrate solution was added to each well, and RLU was immediately measured using a microplate luminometer LuMate (Awareness Technology). Based on the RLU value, IC_50_ was determined from semilogarithmic dose–response plots.

## Supplementary Information

Supplementary Information.
