# Supplementary material for: Comprehensive exploration of chemical space using trisubstituted carboranes
Source: Sci Rep. 2021 Dec 16;11:24101. doi: 10.1038/s41598-021-03459-6 (PMC8677773; doi:10.1038/s41598-021-03459-6)

Supporting Information

Comprehensive Exploration of Chemical Space Using Trisubstituted Carboranes

Yasunobu Asawa,[a] Saki Hatsuzawa,[b] Atsushi Yoshimori,[c] Kentaro Yamada,[d,e] Akira Katoh,[e] Hiroyuki Kouji,[f] Hiroyuki Nakamura*[a,g]

a. School of Life Science and Technology, Tokyo Institute of Technology, 4259, Nagatsuta-cho, Midori-ku, Yokohama 226-8503 (Japan)
b. School of Science, Kitasato University, 1-15-1, Kitazato, Minami-ku, Sagamihara-shi, Kanagawa 252-0373 (Japan)
c. Institute for Theoretical Medicine, Inc., 26-1, Muraoka-Higashi 2-chome, Fujisawa, Kanagawa 251-0012 (Japan)
d. Faculty of Agriculture, Miyazaki University, 1-1, Gakuenkibanadai-Nishi, Miyazaki 889-2192 (Japan)
e. Faculty of Medicine, Oita University, 1-1, Idaigaoka, Hasama-machi, Yufu-city, Oita, 879-5593 (Japan)
f. Oita University Institute of Advanced Medicine, Inc., 17-20, Higashi Kasuga-machi, Oita City, Oita 870-0037 (Japan)
g. Laboratory for Chemistry and Life Science, Institute of Innovative Research, Tokyo Institute of Technology, 4259, Nagatsuta-cho, Midori-ku, Yokohama, Kanagawa 226-8503 (Japan)

List of contents

1. Synthesis of trisubstituted carboranes S2
    General S2
    Synthesis of Scaffold I-type compounds S2~S7
    Synthesis of Scaffold II-type compounds S8~S16
    Synthesis of Scaffold III-type compounds S16~S22
    Synthesis of Scaffold IV-type compounds S22~S26
    Synthesis of Scaffold V-type compounds S26~S38
2. X-ray Crystallography S39~S43
3. Pharmacophore fitting toward RABV inhibitors (Figure S1) S44
4. Inhibitory activity of **Vj** against luciferase (Figure S2) S45
5. Western Blot and RT-PCR with HIF inhibitors (Figure S3) S46
6. RABV protein accumulation with compound **Vp** (Figure S4) S47
7. PMI analysis (Figure S5) S48
8. Reference S48
9. NMR Spectra S49~S206

1. Synthesis of trisubstituted carboranes
General

NMR spectra were recorded on a Bruker biospin AVANCE II (400 MHz for ^{1}H, 100 MHz for ^{13}C) or a Bruker biospin AVANCE III (500 MHz for ^{1}H, 125 MHz for ^{13}C, 470 MHz for ^{11}B) instrument in the indicated solvent. Chemical shifts are reported in units per million (ppm) relative to the signal (0.00 ppm) for internal tetramethylsilane solutions in $CDCl_3$ (7.26 ppm for ^{1}H, 77.16 ppm for ^{13}C). Multiplicities are reported using the following abbreviations: s; singlet, d; doublet, dd; doublet of doublets, t; triplet, q; quartet, m; multiplet, br; broad, J; coupling constants in Hertz. Mass spectra were measured using a JMS-700 Mstation. High-resolution mass spectra (HRMS) were recorded on a Bruker ESI-TOF-MS (micrOTOF II). All reactions were monitored by thin-layer chromatography carried out on 0.2 mm E. Merck silica gel plates (60F-254) with UV light (254 nm) and were visualized using Hanessian's stain solution. Column chromatography was performed on Silica Gel 60 N, purchased from Fuji Silysia Chemical Ltd.

➢ Synthesis of scaffold I-type compounds

Synthesis of 3-((trimethylsilyl)ethynyl)-1,2-dicarba-*closo*-dodecaborane (1)

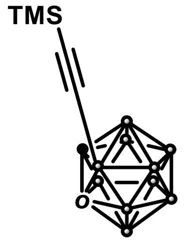

1

To a solution of 3-iodo-1,2-dicarba-*closo*-dodecaborane (909 mg, 3.36 mmol) prepared according to the reported literature[1] and dichlorobis(triphenylphosphine)palladium (236 mg, 0.336 mmol) in THF (3 mL), was added trimethylsilylethynylmagnesium bromide (0.9 M, 9.3 mL, 8.41 mmol) freshly prepared from trimethyl acetylene and ethynylmagnesium bromide. The resulting mixture was stirred at 50 °C for 24 h. Then, the resulting mixture was cooled to room temperature and quenched with water, which was filtered on celite pad. The filtrate was wash with brine and dried over sodium sulfate and concentrated under vacuum. The crude material was purified by column chromatography on silica gel (5% to 10% EtOAc in Hexane) afforded **1201** (573 mg, 2.38 mmol, 71%) as a white solid; m.p. 76-77 °C; ^{1}H NMR (500 MHz; $CDCl_3$): δ 3.68 (s, 2H), 3.00-1.50 (m, 9H), 0.19 (s, 9H); ^{13}C NMR (125 MHz; $CDCl_3$): δ 105.3, 57.5, -0.22; ^{11}B NMR (160 MHz; $CDCl_3$): δ -2.6, -8.7, -11.4, -12.7, -13.7, -14.5; HRMS (ESI, negative) for $C_7H_{20}B_{10}Si$ (m/z): calculated 277.1977 $(M+Cl)^-$, found 277.1966.

Synthesis of 1-((*tert*-butyl)dimethylsilyl)hydroxymethyl)-3-((trimethylsilyl)ethynyl)-1,2-dicarba-*closo*-dodecaborane (S1)

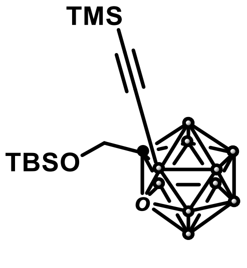

S1

To a solution of **1** (572 mg, 2.38 mmol) in THF (9.5 mL), was slowly added *n*-BuLi 1.6 M solution in hexane (1.45 mL, 2.38 mmol) at -78 °C. After the resulting mixture was stirred at 0 °C under argon atmosphere for 2 h, paraformaldehyde (79 mg, 2.62 mmol) was added at 0 °C. Then, the resulting mixture was warmed up to room temperature and stirred for 3 h under argon atmosphere. The reaction mixture was quenched with aq. 1N HCl. The resulting mixture was partitioned between the aqueous and organic layers. The aqueous layer was washed with EtOAc, and the mixture was extracted with EtOAc, washed with brine, dried over sodium sulfate, and concentrated under vacuum. The residue was used in the next step without further purification.

To a solution of the above material and 2,6-lutidine (141 µL, 1.21 mmol) in DCM (3.2 mL) was slowly added TBSOTf (222 µL, 0.967 mmol) at 0 °C under argon atmosphere. The resulting mixture was stirred at 0 °C until the starting material disappeared as monitored by TLC analysis. Then, the reaction mixture was quenched with a small amount of aq. $NaHCO_3$ and filtered on celite pad. The filtrate was concentrated under vacuum. The crude material was purified by column

chromatography on silica gel (0% to 10% EtOAc in Hexane) to afford **S1** (187 mg, 0.486 mmol, 20% 2 steps) as white solid. ^{1}H NMR (500 MHz; $CDCl_3$): δ 4.26 (d, *J* = 10.5 Hz, 1H), 4.03 (d, *J* = 10.5 Hz, 1H), 3.00-1.50 (m,9H), 0.90 (s, 9H), 0.19 (s, 9H), 0.10 (*J* = 1.5 Hz, 6H); ^{13}C NMR (125 MHz; $CDCl_3$): δ 74.9, 63.8, 58.9, 25.8, 18.4, -0.13, -5.3, -5.5; ^{11}B NMR (160 MHz; $CDCl_3$): δ -3.9, -5.0, -8.2, -10.5, -11.3, -13.7; HRMS (ESI, positive) for $C_{14}H_{36}B_{10}OSi_2$ (m/z): calculated 409.3156 $(M+Na)^+$, found 409.3157.

Synthesis of 1-((*tert*-butyl)dimethylsilyl)hydroxymethyl)-2-hydroxymethyl-3-((trimethylsilyl)ethynyl)-1,2-dicarba-*closo*-dodecaborane (2)

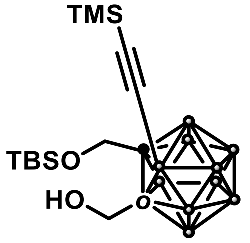

2

To a solution of **S1** (127 mg, 0.329 mmol) in THF (2.4 mL), was slowly added *n*-BuLi 1.6 M solution in hexane (221 µL, 0.362 mmol) at -78 °C. After the resulting mixture was stirred at 0 °C under argon atmosphere for 2 h, paraformaldehyde (12 mg, 0.395 mmol) was added at 0 °C. Then, the resulting mixture was warmed up to room temperature and stirred for 3 h under argon atmosphere. The reaction mixture was quenched with aq. 1N HCl. The resulting mixture was partitioned between the aqueous and organic layers. The aqueous layer was washed with EtOAc, and the mixture was extracted with EtOAc, washed with brine, dried over sodium sulfate, and concentrated under vacuum. The crude material was purified by column chromatography on silica gel (10% EtOAc in Hexane) to afford **2** (27.4 mg, 0.0661 mmol, 20%) as colorless oil.

^{1}H NMR (500 MHz; $CDCl_3$): δ 4.50 (d, *J* = 12.3 Hz, 1H), 4.24 (dd, *J* = 5.2 Hz, *J* = 13.7 Hz, 1H), 4.11 (d, *J* = 12.3 Hz, 1H), 4.03 (dd, *J* = 5.2 Hz, *J* = 13.7 Hz, 1H), 2.90-2.87 (m, 2H), 2.90-1.40 (m, 9H), 0.92 (s, 9H), 0.22 (s, 9H), 0.14 (*J* = 6.8 Hz, 6H); ^{13}C NMR (125 MHz; $CDCl_3$): δ 110.0, 78.3, 77.7, 64.1, 63.5, 25.9, 18.5, -0.2, -5.3; ^{11}B NMR (160 MHz; $CDCl_3$): δ -3.94, -10.8, -11.6; HRMS (ESI, positive) for $C_{15}H_{38}B_{10}O_2Si_2$ (m/z): calculated 439.3263 $(M+Na)^+$, found 439.3270.

Synthesis of 1-((*tert*-butyl)dimethylsilyl)hydroxymethyl)-2-hydroxymethyl-3-ethynyl-1,2-dicarba-*closo*-dodecaborane (3); 1,2-bis(hydroxymethyl)-3-ethynyl-1,2-dicarba-*closo*-dodecaborane (4)

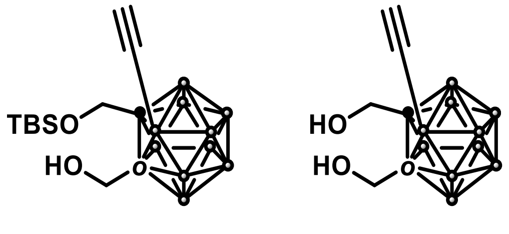

3 **4**

To a solution of **2** (96 mg, 0.232 mmol) in MeOH (1 mL), potassium carbonate (160 mg, 1.16 mmol) was added. The resulting mixture was stirred at room temperature until the full conversion of a starting material was observed. After that, the reaction mixture was concentrated under pressure. To the resulting mixture was added water and EtOAc after which the product was partitioned between the aqueous and organic layers. The aqueous layer was washed with EtOAc and combined organic layers were washed with brine, dried over sodium sulfate and concentrated under vacuum. The crude material was purified by column chromatography on silica gel (10% to 50% EtOAc in Hexane) to afford faster eluted product **3** (45 mg, 0.132 mmol, 57%) as colorless oil and later eluted product **4** (21 mg, 0.0924 mmol, 40%) as white solid in total 97% yield.

3: ^{1}H NMR (400 MHz; $CDCl_3$): δ 4.49 (d, *J* = 12.4 Hz, 1H), 4.29 (d, *J* = 13.7 Hz, 1H), 4.13 (d, *J* = 12.4 Hz, 1H), 4.07 (d, *J* = 13.7 Hz, 1H), 2.60-1.50 (m, 9H), 2.48 (s, 1H), 0.92 (s, 9H), 0.15 (d, *J* = 7.4 Hz, 6H); ^{13}C NMR (100 MHz; $CDCl_3$): δ 89.3, 78.4, 77.8, 63.9, 63.3, 25.7, 18.3, -5.5, -5.7; ^{11}B NMR (128 MHz; $CDCl_3$): δ -3.8, -9.7, -10.5, -11.5, -12.7; HRMS (ESI, positive) for $C_{12}H_{30}B_{10}O_2Si$ (m/z): calculated 367.2862 $(M+Na)^+$, found 367.2861.

4: ^{1}H NMR (500 MHz; $CDCl_3$): δ 3.46 (s, 2H), 2.98 (s, 1H), 1.77 (s, 1H), 2.90-1.60 (m, 9H); ^{13}C NMR (125 MHz; $CDCl_3$): δ 89.8, 78.3, 63.7; ^{11}B NMR (160 MHz; $CDCl_3$): δ -3.6, -9.9, -11.4, -12.8; HRMS (ESI, positive) for $C_6H_{16}B_{10}O_2$ (m/z): calculated 253.1985 $(M+Na)^+$, found 253.1978; m.p. 130-131°C.

Synthesis of 1-((*tert*-butyl)dimethylsilyl)hydroxymethyl)-2-phenylacetoxymethyl-3-ethynyl-1,2-dicarba-*closo*-dodecaborane (5)

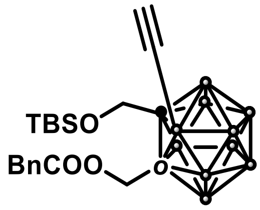

5

To a solution of **3** (35.3 mg, 0.103 mmol) and triethylamine (21 µL, 0.155 mmol) in CH_2Cl_2 (500 µL), was slowly added phenylacetyl chloride (16 µL, 0.124 mmol) in CH_2Cl_2 at 0 °C. After the resulting mixture was stirred 0 °C under argon atmosphere for 1 h, aq. $NaHCO_3$ and CH_2Cl_2 were added. The product was partitioned between the aqueous and organic layers. The aqueous layer was washed with CH_2Cl_2 and combined organic layers were dried over sodium sulfate and concentrated under vacuum. The crude material was purified by column chromatography on silica gel (10% EtOAc in Hexane) to afford **5** (38.3 mg, 0.0831 mmol, 81%) as colorless oil.

^{1}H NMR (400 MHz; $CDCl_3$): δ 7.39-7.27 (m, 5H), 4.74 (d, *J* = 13.1 Hz, 1H), 4.59 (d, *J* = 13.1 Hz, 1H), 4.32 (d, *J* = 12.4 Hz, 1H), 4.11 (d, *J* = 12.4 Hz, 1H), 3.68 (s, 2H), 3.00-1.50 (m, 9H), 0.90 (s, 9H), 0.09 (d, *J* = 8.2 Hz, 1H); ^{13}C NMR (100 MHz; $CDCl_3$): δ 170.1, 133.0, 129.6, 128.8, 127.6, 89.3, 79.2, 74.1, 63.6, 62.3, 41.1, 25.8, 18.3, -5.4, -5.5; ^{11}B NMR (128 MHz; $CDCl_3$): δ -3.4, -4.4, -9.9, -11.0, -11.8; HRMS (ESI, positive) for $C_{20}H_{36}B_{10}O_3Si$ (m/z): calculated 485.3289 $(M+Na)^+$, found 485.3287.

Synthesis of 1-hydroxymethyl-2-phenylacetoxymethyl-3-ethynyl-1,2-dicarba-*closo*-dodecaborane (S2)

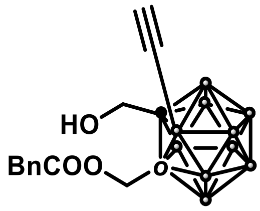

S2

To a solution of **5** (38.3 mg, 83.1 µmol) in CH_2Cl_2 (500 µL), was added 4N HCl in Dioxane (500 µL). The resulting mixture was stirred at room temperature until the full conversion of a starting material was observed. After that, the reaction mixture was concentrated under pressure. The crude material was purified by column chromatography on silica gel (25% EtOAc in Hexane) to afford **S2** (26.0 mg, 0.0751 mmol, 90%) as colorless oil.

^{1}H NMR (400 MHz; $CDCl_3$): δ 7.41-7.29 (m, 5H), 4.89 (d, *J* = 13.6 Hz, 1H), 4.64 (d, *J* = 13.6 Hz, 1H), 4.26 (d, *J* = 14.0 Hz, 1H), 4.14 (d, *J* = 14.0 Hz, 1H), 3.73 (s, 2H), 3.00-1.50 (m, 9H); ^{13}C NMR (100 MHz; $CDCl_3$): δ 170.8, 132.7, 129.5, 129.0, 127.8, 79.8, 74.9, 63.3, 62.4, 41.2; ^{11}B NMR (128 MHz; $CDCl_3$): δ -3.4, -3.9, -9.5, -10.5, -12.3; HRMS (ESI, positive) for $C_{14}H_{22}B_{10}O_3$ (m/z): calculated 367.2862 $(M+Na)^+$, found 367.2861.

Synthesis of 1-(3-methylbutanoyl)oxymethyl-2-phenylacetoxymethyl-3-ethynyl-1,2-dicarba-*closo*-dodecaborane (6)

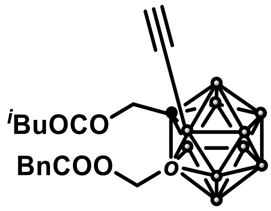

6

To a solution of **S2** (18.9 mg, 43.9 µmol) and triethylamine (18 µL, 0.132 mmol) in CH_2Cl_2 (1 mL), was slowly added isovaleryl chloride (8.0 µL, 65.8 µmol) in CH_2Cl_2 at 0 °C. After the resulting mixture was stirred 0 °C under argon atmosphere for 1 h, aq. $NaHCO_3$ and CH_2Cl_2 were added. The product was partitioned between the aqueous and organic layers. The aqueous layer was washed with CH_2Cl_2 and combined organic layers were dried over sodium sulfate and concentrated under vacuum. The crude material was purified by column chromatography on silica gel (10% Et_2O in Hexane) to afford **6** (20.9 mg, 48.5 µmol, quant.) as colorless oil.

^{1}H NMR (500 MHz; $CDCl_3$): δ 7.36-7.26 (m, 5H), 4.77-4.67 (m, 3H), 4.61 (d, *J* = 13.5 Hz, 1H), 3.68 (s, 2H), 3.00-1.50 (m, 9H), 2.44 (s, 1H), 2.20 (d, *J* = 6.5 Hz, 2H), 2.13-2.07 (m, 1H), 0.96 (*J* = 1.2 Hz, *J* = 6.6 Hz, 6H); ^{13}C NMR (125

MHz; $CDCl_3$): δ 171.4, 169.9, 132.8, 129.5, 128.9, 127.7, 89.8, 75.5, 75.2, 62.2, 61.7, 42.8, 41.0, 25.6, 22.50 22.48; ^{11}B NMR (160 MHz; $CDCl_3$): δ -3.5, -9.4, -10.5, -12.0; HRMS (ESI, positive) for $C_{19}H_{30}B_{10}O_4$ (m/z): calculated 455.2997 $(M+Na)^+$, found 455.2997.

Synthesis of 1,2-bis(phenylacetoxymethyl)-3-ethynyl-1,2-dicarba-*closo*-dodecaborane (7a)

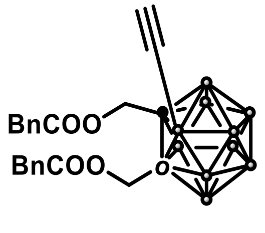

7a

This compound was prepared from **4** (19.5 mg, 85.4 μmol) and 2.0 equivalent of phenylacetyl chloride using the procedure described before for **5** in 72% yield as colorless oil.

^{1}H NMR (500 MHz; $CDCl_3$): δ 7.34-7.24 (m, 10H), 4.71 (d, *J* = 13.5 Hz, 2H), 4.63 (d, *J* = 13.5 Hz, 2H), 3.00-1.50 (m, 9H), 3.61 (s, 4H); ^{13}C NMR (125 MHz; $CDCl_3$): δ 169.9, 132.8, 129.5, 128.9, 127.7, 89.9, 75.3, 62.2, 40.9; ^{11}B NMR (160 MHz; $CDCl_3$): δ -3.4, -9.4, -10.6, -12.1; HRMS (ESI, positive) for $C_{22}H_{28}B_{10}O_4$ (m/z): calculated 489.2844 $(M+Na)^+$, found 489.2842.

Synthesis of 1,2-bis((3-methylbutanoyl)oxymethyl)-3-ethynyl-1,2-dicarba-*closo*-dodecaborane (7b)

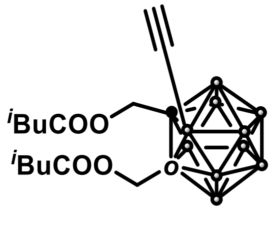

7b

This compound was prepared from **4** (15.3 mg, 67.0 μmol) and 2.0 equivalent of isovaleryl chloride using the procedure described before for **6** in 74% yield as colorless oil.

^{1}H NMR (500 MHz; $CDCl_3$): δ 4.78 (d, *J* = 13.5 Hz, 2H), 4.66 (d, *J* = 13.5 Hz, 2H), 3.00-1.50 (m, 9H), 2.48 (s, 1H), 2.26 (d, *J* = 6.5 Hz, 4H), 2.15-2.10 (m, 2H), 0.98 (d, *J* = 6.7 Hz, 12H); ^{13}C NMR (125 MHz; $CDCl_3$): δ 171.4, 89.8, 75.4, 61.8, 42.9, 25.6, 22.5; ^{11}B NMR (160 MHz; $CDCl_3$): δ -3.5, -9.4, -10.6, -12.0; HRMS (ESI, positive) for $C_{16}H_{32}B_{10}O_4$ (m/z): calculated 421.3149 $(M+Na)^+$, found 421.3148.

Synthesis of 1-(3-methylbutanoyl)oxymethyl-2-phenylacetoxymethyl-3-(1*H*-1,2,3-triazol-4-yl)benzylcarbamoyl-1,2-dicarba-*closo*-dodecaborane (Ia)

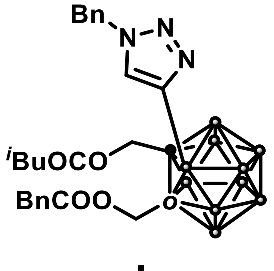

Ia

To a solution of **6** (9.8 mg, 22.8 μmol) in DMF:H_2O (4:1, v/v, 500 μL), were added CuI (0.43 mg, 2.28 μmol), sodium ascorbate (0.90 mg, 4.46 μmol), and benzylazide (6.0 μL, 45.5 μmol). The mixture was stirred vigorously at room temperature for 12 h. The reaction was quenched with water and the mixture was extracted with EtOAc, washed with brine, dried over sodium sulfate, and concentrated under vacuum. The residue was purified by column chromatography on silica gel (20% EtOAc in Hexane) to afford **Ia** (11 mg, 20.0 μmol, 88%) as colorless oil.

^{1}H NMR (500 MHz; $CDCl_3$):δ 7.72 (s, 1H), 7.40-7.24 (m, 10H), 5.55 (s, 2H), 4.99 (d, *J* = 13.7 Hz, 2H), 4.97-4.81 (m, 3H), 3.61 (s, 2H), 2.90-1.60 (m, 9H), 2.15 (d, *J* = 6.5 Hz, 2H), 2.09-2.03 (m, 1H), 0.94 (dd, *J* = 1.8 Hz, *J* = 6.6 Hz, 6H); ^{13}C NMR (125 MHz; $CDCl_3$): δ 171.3, 169.9, 134.3, 133.0, 131.6, 129.5, 129.1, 128.8, 128.4, 127.6, 76.1, 75.7, 62.0, 61.4, 54.1, 42.9, 41.0, 25.5, 22.52, 22.51, ; ^{11}B NMR (160 MHz; $CDCl_3$): δ -3.6, -7.2, -9.5, -10.9, -11.8; HRMS (ESI, positive) for $C_{26}H_{37}B_{10}N_3O_4$ (m/z): calculated 588.3644 $(M+Na)^+$, found 588.3645.

Synthesis of 1-(3-methylbutanoyl)oxymethyl-2-phenylacetoxymethyl-3-((1*H*-1,2,3-triazol-4-yl)-3-methyl-butanoyl)-1,2-dicarba-*closo*-dodecaborane (Ib)

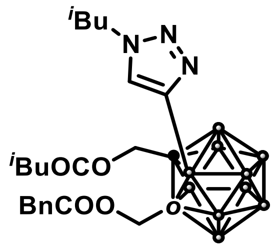

Ib

This compound was prepared from **6** (11.1 mg, 25.8 μmol) and 2.0 equivalent of isovalerylazide using the procedure described before for **Ia** in 45% yield as colorless oil.
^{1}H NMR (500 MHz; $CDCl_3$):δ 7.76 (s, 1H), 7.36-7.26 (m, 5H), 5.00 (d, *J* = 13.7 Hz, 2H), 4.91-4.83 (m, 3H), 4.19 (d, *J* = 7.3 Hz, 2H), 3.64 (s, 2H), 2.27-2.21 (m, 1H), 2.16 (d, *J* = 6.5 Hz, 2H), 2.10-2.04 (m, 1H), 0.97-0.94 (m, 12H); ^{13}C NMR (125 MHz; $CDCl_3$): δ 171.3, 169.9, 133.0, 132.1, 129.5, 128.8, 127.6, 76.0, 75.7, 62.0, 61.4, 57.5, 42.9, 41.1, 29.8, 25.6, 22.5, 20.1; ^{11}B NMR (160 MHz; $CDCl_3$): δ -3.6, -7.2, -9.6, -12.0; HRMS (ESI, positive) for $C_{23}H_{39}B_{10}N_3O_4$ (m/z): calculated 554.3798 $(M+Na)^+$, found 554.3796.

Synthesis of 1,2-bis(phenylacetoxymethyl)-3-(1*H*-1,2,3-triazol-4-yl)benzylcarbamoyl-1,2-dicarba-*closo*-dodecaborane (Ic)

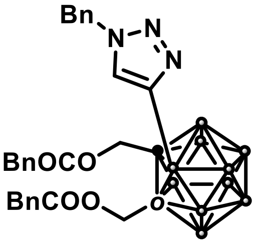

Ic

This compound was prepared from **7a** (27.6 mg, 59.4 μmol) using the procedure described before for **Ia** in 8% yield as colorless oil. ^{1}H NMR (500 MHz; $CDCl_3$):δ 7.68 (s, 1H), 7.38-7.21 (m, 15H), 5.53 (s, 2H), 4.93 (d, *J* = 13.7 Hz, 2H), 4.80 (d, *J* = 13.7 Hz, 2H), 3.55 (s, 4H), 2.80-1.60 (m, 9H); ^{13}C NMR (125 MHz; $CDCl_3$): δ 169.8, 134.3, 133.0, 131.6, 129.5, 129.4, 129.1, 128.8, 128.4, 127.6, 75.8, 62.0, 54.1, 40.9; ^{11}B NMR (160 MHz; $CDCl_3$): δ -3.5, -7.5, -9.5, -11.8; HRMS (ESI, positive) for $C_{29}H_{35}B_{10}N_3O_4$ (m/z): calculated 622.3491 $(M+Na)^+$, found 622.3491.

Synthesis of 1,2-bis(phenylacetoxymethyl)-3-((1*H*-1,2,3-triazol-4-yl)-3-methyl-butanoyl)-1,2-dicarba-*closo*-dodecaborane (Id)

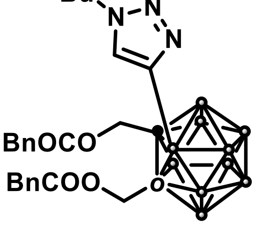

Id

This compound was prepared from **7a** (26.5 mg, 57.0 μmol) and 2.0 equivalent of isovalerylazide using the procedure described before for **Ia** in 10% yield as colorless oil. ^{1}H NMR (500 MHz; $CDCl_3$):δ 7.71 (s, 1H), 7.34-7.23 (m, 10H), 4.92 (d, *J* = 13.7 Hz, 2H), 4.81 (d, *J* = 13.7 Hz, 2H), 4.16 (d, *J* = 7.2 Hz, 2H), 3.58 (s, 4H), 2.90-1.80 (m, 9H), 2.24-2.19 (m, 1H), 0.95 (d, *J* = 6.7 Hz, 6H); ^{13}C NMR (125 MHz; $CDCl_3$): δ 169.9, 133.0, 132.1, 129.5, 128.8, 127.6, 75.7, 62.0, 57.5, 41.0, 29.8, 20.0; ^{11}B NMR (160 MHz; $CDCl_3$): δ –3.4, -7.3, -9.4, -10.8; HRMS (ESI, positive) for $C_{25}H_{37}B_{10}N_3O_4$ (m/z): calculated 588.3644 $(M+Na)^+$, found 588.3642.

Synthesis of 1,2-bis((3-methylbutanoyl)oxymethyl)-3-(1*H*-1,2,3-triazol-4-yl)benzylcarbamoyl-1,2-dicarba-*closo*-dodecaborane (Ie)

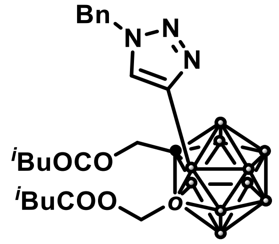

Ie

This compound was prepared from **7b** (18.4 mg, 46.4 μmol) using the procedure described before for **Ia** in 72% yield as colorless oil. ^{1}H NMR (500 MHz; $CDCl_3$):δ 7.76 (s, 1H), 7.41-7.39 (m, 3H), 7.32-7.31 (m, 2H), 5.57 (s, 2H), 4.95 (d, *J* = 13.6 Hz, 2H), 4.87 (d, *J* = 13.6 Hz, 2H), 3.00-1.70 (m, 9H), 2.20 (d, *J* = 6.6 Hz, 2H), 2.13-2.06 (m, 1H), 0.95 (dd, *J* = 1.2 Hz, *J* = 6.6 Hz, 12H); ^{13}C NMR (125 MHz; $CDCl_3$): δ 171.3, 134.3, 131.7, 129.4, 129.1, 128.4, 76.0, 61.5, 54.2, 43.0, 28.5, 25.6, 22.53, 22.51; ^{11}B NMR (160 MHz; $CDCl_3$): δ -3.5, -7.4, -9.6, -10.8, -11.8; HRMS (ESI, positive) for $C_{23}H_{39}B_{10}N_3O_4$ (m/z): calculated 554.3798 $(M+Na)^+$, found 554.3812.

Synthesis of 1,2-bis((3-methylbutanoyl)oxymethyl)-3-((1*H*-1,2,3-triazol-4-yl)-3-methyl-butanoyl)-1,2-dicarba-*closo*-dodecaborane (If)

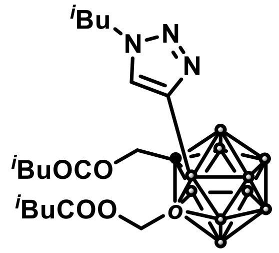

If

This compound was prepared from **7b** (23.7 mg, 59.8 μmol) and 2.0 equivalent of isovalerylazide using the procedure described before for **Ia** in 34% yield as colorless oil. ^{1}H NMR (500 MHz; $CDCl_3$):δ 7.82 (s, 1H), 4.95 (d, *J* = 13.7 Hz, 2H), 4.89 (d, *J* = 13.7 Hz, 2H), 4.21 (d, *J* = 7.2 Hz, 2H), 2.29-2.23 (m, 1H), 2.21 (d, *J* = 6.7 Hz, 2H), 2.14-2.07 (m, 2H), 0.98-0.95 (m, 18H); ^{13}C NMR (125 MHz; $CDCl_3$): δ 171.3, 132.2, 76.0, 61.5, 57.6, 43.0, 29.8, 25.6, 22.53, 22.52, 20.1; ^{11}B NMR (160 MHz; $CDCl_3$): δ -3.5, -7.2, -9.5, -10.9, -11.8; HRMS (ESI, positive) for $C_{20}H_{41}B_{10}N_3O_4$ (m/z): calculated 520.3951 $(M+Na)^+$, found 520.3934.

➢ Synthesis of scaffold II-type compounds

Synthesis of 1-(3-(4-methoxybenzyl)hydroxymethyl)-1,2-dicarba-*closo*-dodecaborane (9)

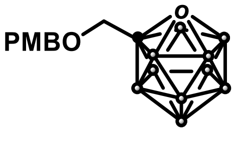

9

To a solution of 1-methoxy-4-((prop-2-yn-1-yloxy)methyl)benzene (**8**) (370 mg, 2.10 mmol) prepared according to the reported literature[2] and *N,N*-dimethylaniline (399 μL, 3.15 mmol) in chlorobenzene, was added decaborane (308 mg, 2.52 mmol). The resulting mixture was stirred at 130 °C under argon atmosphere for 10 min with microwave synthesizer. To the resulting mixture was added 1M HCl and Et_2O after which the product was partitioned between the aqueous and organic layers. The aqueous layer was washed with Et_2O and combined organic layers were washed with brine, dried over sodium sulfate, and concentrated under vacuum. Then, the crude material was purified by column chromatography on silica gel (10% EtOAc in Hexane) to afford **9** (489 mg, 1.66 mmol, 79%) as a white solid. m.p. 65-67 °C; ^{1}H NMR (500 MHz; $CDCl_3$): δ 7.19 (d, *J* = 8.8 Hz, 2H), 6.89 (d, *J* = 8.8 Hz, 2H), 4.46 (s, 2H), 3.95 (s, 1H), 3.83 (s, 2H), 3.82 (s, 3H), 3.00-1.60 (m, 10H); ^{13}C NMR (125 MHz; $CDCl_3$): δ 159.8, 129.6, 128.4, 114.2, 73.5, 72.7, 70.6, 57.8, 55.4; ^{11}B NMR (160 MHz; $CDCl_3$): δ -3.13, -4.95, -9.17, -11.71, -13.37; HRMS (ESI, positive) for $C_{11}H_{22}B_{10}O_2$ (m/z): calculated 319.2461 $(M+Na)^+$, found 319.2460.

Synthesis of 1-(3-(4-methoxybenzyl)hydroxymethyl)-4-((triisopropylsilyl)ethynyl)-1,2-dicarba-*closo*-dodecaborane (10)

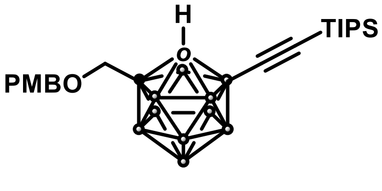

10

To a solution of **9** (1.56 g, 5.30 mmol) in Et_2O (22 mL), was slowly added *n*-BuLi 1.6 M solution in hexane (3.69 mL, 5.83 mmol) at -78 °C. After the resulting mixture was stirred at -78 °C under argon atmosphere for 2 h, CO_2 balloon was attached. Then, the resulting mixture was warmed up to room temperature and stirred for 12 h under CO_2 atmosphere. After that, the reaction mixture was concentrated under pressure. Hexane was added after which the product was partitioned between the aqueous and organic layers. The aqueous layer was washed with hexane. Then, to the aqueous layer, were added 6 M HCl and Et_2O after which the product was partitioned between the aqueous and organic layers. The aqueous layer was washed with hexane and combined organic layers were dried over sodium sulfate and concentrated under vacuum. The crude material was used in the next step without further purification.

To a suspension of the crude material, K_2HPO_4 (1.85 g, 10.6 mmol), $Pd(OAc)_2$ (119 mg, 0.530 mmol), and AgOAc (2.65 g, 15.9 mmol) in toluene (50 mL), was slowly added (triisopropylsilyl)acetylene (3.5 mL, 15.9 mmol) in toluene (3 mL). The resulting mixture was stirred at 80 °C for 16 h. Then, the resulting mixture was cooled to room temperature and quenched with water, which was filtered on celite pad. The filtrate was extracted with Et_2O, washed with brine, dried over sodium sulfate, and concentrated under vacuum. The crude material was purified by column chromatography on silica gel (5% to 10% EtOAc in Hexane) afforded **10** (776 mg, 1.63 mmol, 31%) as colorless oil.

^{1}H NMR (500 MHz; $CDCl_3$): δ 7.20 (d, *J* = 8.7 Hz, 2H), 6.90 (d, *J* = 8.7 Hz, 2H), 4.48 (s, 2H), 4.10 (s, 1H), 3.84-3.81 (m, 5H), 2.80-1.60 (m, 9H), 1.09-1.05 (m, 21H); ^{13}C NMR (125 MHz; $CDCl_3$): δ 159.9, 129.6, 128.4, 114.2, 101.7, 73.6, 72.5, 70.5, 59.2, 55.5, 18.7, 11.3; ^{11}B NMR (160 MHz; $CDCl_3$): δ -3.63, -4.12, -8.40, -9.76, -12.10, -12.66; HRMS (ESI, positive) for $C_{22}H_{42}B_{10}O_2Si$ (m/z): calculated 499.3823 $(M+Na)^+$, found 499.3822.

Synthesis of 1-(3-(4-methoxybenzyl)hydroxymethyl)-2-(hydroxymethyl)-4-((triisopropylsilyl)ethynyl)-1,2-dicarba-*closo*-dodecaborane (S3)

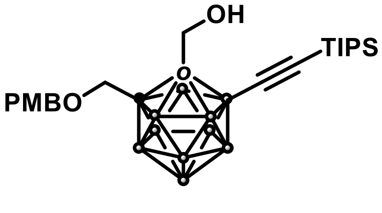

S3

To a solution of **10** (776 mg, 1.63 mmol) in THF (13 mL), was slowly added *n*-BuLi 1.6 M solution in hexane (1.14 mL, 1.80 μmol) at 0 °C. After the resulting mixture was stirred at room temperature under argon atmosphere for 2 h, paraformaldehyde (59 mg, 1.96 mmol) was added at 0 °C. Then, the resulting mixture was warmed up to room temperature and stirred for 3 h under argon atmosphere. The reaction mixture was quenched with aq. 1N HCl. The resulting mixture was partitioned between the aqueous and organic layers. The aqueous layer was washed with EtOAc, and the mixture was extracted with EtOAc, washed with brine, dried over sodium sulfate, and concentrated under vacuum. The crude material was purified by column chromatography on silica gel (5% to 10% EtOAc in Hexane) afforded **S3** (647 mg, 1.30 mmol, 80%) as a colorless oil.
^{1}H NMR (500 MHz; $CDCl_3$): δ 7.23 (d, *J* = 8.7 Hz, 2H), 6.90 (d, *J* = 8.7 Hz, 2H), 4.54 (d, *J* = 11.5 Hz, 1H), 4.50 (d, *J* = 11.5 Hz, 1H), 4.33-4.23 (m, 2H), 4.04 (d, *J* = 12.1 Hz, 1H), 3.97 (d, *J* = 12.1 Hz, 1H), 3.82 (s, 3H), 3.10-3.07 (m, 1H), 3.00-1.70 (m, 9H), 1.07-1.03 (m, 21H); ^{13}C NMR (125 MHz; $CDCl_3$): δ 159.8, 129.6, 127.9, 114.2, 103.9, 79.0, 76.0, 73.6, 69.9, 63.4, 55.3, 18.6, 11.1; ^{11}B NMR (160 MHz; $CDCl_3$): δ -2.70, -3.67, -9.51, -10.66, -12.07; HRMS (ESI, positive) for $C_{23}H_{44}B_{10}O_3Si$ (m/z): calculated 529.3919 $(M+Na)^+$, found 529.3919.

Synthesis of 1-(3-(4-methoxybenzyl)hydroxymethyl)-2-(hydroxymethyl)-4-ethynyl-1,2-dicarba-*closo*-dodecaborane (11)

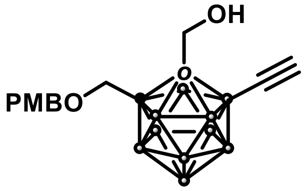

11

To a solution of **S3** (21 mg, 41.6 μmol) in THF (500 μL), was slowly added TBAF 1 M solution in THF (42 μL, 41.6 μmol) at 0 °C. After the resulting mixture was stirred at 0 °C under argon atmosphere for 30 min, the reaction mixture was quenched with water. The resulting mixture was partitioned between the aqueous and organic layers. The aqueous layer was extracted with Et_2O, dried over sodium sulfate, and concentrated under vacuum. The crude material was purified by column chromatography on silica gel (20% EtOAc in Hexane) afforded **11** (16 mg, quant.) as colorless oil.
^{1}H NMR (500 MHz; $CDCl_3$): δ 7.23 (d, *J* = 8.6 Hz, 2H), 6.90 (d, *J* = 8.6 Hz, 2H), 4.53 (d, *J* = 11.5 Hz, 1H), 4.50 (d, *J* = 11.5 Hz, 1H), 4.33-4.29 (m, 1H), 4.21 (m, 1H), 4.05 (d, *J* = 12.0 Hz, 1H), 4.00 (d, *J* = 12.0 Hz, 1H), 3.82 (s, 3H), 3.00 (t, *J* = 7.8 Hz, 1H), 3.00-1.70 (m, 9H), 2.34 (s, 1H); ^{13}C NMR (125 MHz; $CDCl_3$): δ 160.0, 129.8, 127.8, 114.3, 87.3, 79.2, 76.1, 73.8, 70.1, 63.3, 55.4; ^{11}B NMR (160 MHz; $CDCl_3$): δ -2.56, -3.37, -9.60, -10.71, -11.3, -11.9; HRMS (ESI, positive) for $C_{14}H_{24}B_{10}O_3$ (m/z): calculated 373.2571 $(M+Na)^+$, found 373.2561.

Synthesis of 1-(3-(4-methoxybenzyl)hydroxymethyl)-2-((*tert*-butyl)dimethylsilyl)-hydroxymethyl)-4-ethynyl-1,2-dicarba-*closo*-dodecaborane (S4)

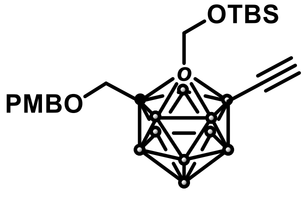

S4

To a solution of **11** (343 mg, 1.00 mmol) and 2,6-lutidine (233 μL, 2.00 mmol) in DCM (4 mL) was slowly added TBSOTf (276 μL, 1.20 mmol) at 0 °C under argon atmosphere. The resulting mixture was stirred at 0 °C until the starting material disappeared as monitored by TLC analysis. Then, the reaction mixture was quenched with a small amount of aq. $NaHCO_3$ and filtered on celite pad. The filtrate was concentrated under vacuum. The crude material was purified by

column chromatography on silica gel (0% to 10% EtOAc in Hexane) to afford **S4** (451 mg, 0.98 mmol, 98%) as colorless oil. ^{1}H NMR (500 MHz; $CDCl_3$): δ 7.22 (d, *J* = 8.7 Hz, 2H), 6.89 (d, *J* = 8.7 Hz, 2H), 4.53 (d, *J* = 11.7 Hz, 1H), 4.47 (d, *J* = 11.7 Hz, 1H), 4.41 (d, *J* = 12.3 Hz, 1H), 4.20 (d, *J* = 12.3 Hz, 1H), 3.99 (d, *J* = 11.9 Hz, 1H), 3.96 (d, *J* = 11.9 Hz, 1H), 3.82 (s, 3H), 3.00-1.50 (m, 9H), 2.29 (s, 1H), 0.90 (s, 9H), 0.19 (s, 9H), 0.08 (d, *J* = 3.3 Hz, 6H); ^{13}C NMR (125 MHz; $CDCl_3$): δ 159.7, 129.4, 128.8, 114.1, 86.8, 78.9, 76.6, 73.4, 69.6, 63.0, 55.4, 25.8, 18.3, -5.46, -5.47; ^{11}B NMR (160 MHz; $CDCl_3$): δ -2.68, -3.70, -10.9, -11.8; HRMS (ESI, positive) for $C_{20}H_{38}B_{10}O_3Si$ (m/z): calculated 487.3446 $(M+Na)^+$, found 487.3453.

Synthesis of 1-(3-(4-methoxybenzyl)hydroxymethyl)-2-((*tert*-butyl)dimethylsilyl)-hydroxymethyl)-4-(3-oxoprop-1-yn-1-yl)-1,2-dicarba-*closo*-dodecaborane (12)

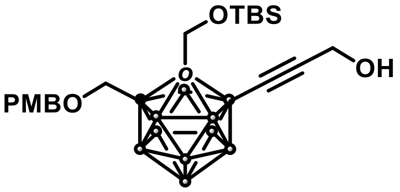

12

To a solution of **S4** (154 mg, 0.333 mmol) in THF (2.7 mL), was slowly added *n*-BuLi 1.6 M solution in hexane (232 μL, 0.366 mmol) at -78 °C. After the resulting mixture was stirred at 0 °C under argon atmosphere for 2 h, paraformaldehyde (12 mg, 0.399 mmol) was added at 0 °C. Then, the resulting mixture was warmed up to room temperature and stirred for 3 h under argon atmosphere. The reaction mixture was quenched with aq. 1N HCl. The resulting mixture was partitioned between the aqueous and organic layers. The aqueous layer was washed with EtOAc, and the mixture was extracted with EtOAc, washed with brine, dried over sodium sulfate, and concentrated under vacuum. The crude material was purified by column chromatography on silica gel (25% to 40% Et_2O in Hexane) to afford **12** (130 mg, 0.263 mmol, 79%) as colorless oil.

^{1}H NMR (500 MHz; $CDCl_3$): δ 7.21 (d, *J* = 8.7 Hz, 2H), 6.89 (d, *J* = 8.7 Hz, 2H), 4.52 (d, *J* = 11.7 Hz, 1H), 4.46 (d, *J* = 11.7 Hz, 1H), 4.38 (d, *J* = 12.3 Hz, 1H), 4.27 (s, 2H), 4.17 (d, *J* = 12.3 Hz, 1H), 3.98 (d, *J* = 11.9 Hz, 1H), 3.94 (d, *J* = 11.9 Hz, 1H), 3.81 (s, 3H), 3.00-1.50 (m, 9H), 0.89 (s, 9H), 0.19 (s, 9H), 0.07 (d, *J* = 1.3 Hz, 6H); ^{13}C NMR (125 MHz; $CDCl_3$): δ 159.7, 129.4, 128.8, 114.1, 97.2, 78.6, 78.5, 73.4, 69.7, 63.0, 55.4, 51.8, 18.3, -5.46, -5.48; ^{11}B NMR (160 MHz; $CDCl_3$): δ -2.77, -3.72, -10.6, -11.9; HRMS (ESI, positive) for $C_{21}H_{40}B_{10}O_4Si$ (m/z): calculated 517.3553 $(M+Na)^+$, found 517.3556.

Synthesis of 1-(3-(4-methoxybenzyl)hydroxymethyl)-2-((*tert*-butyl)dimethylsilyl)-hydroxymethyl)-4-(2-phenylacetoxy)prop-1-yn-1-yl)-1,2-dicarba-*closo*-dodecaborane (13a)

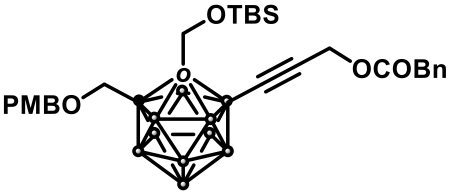

13a

To a solution of **12** (70 mg, 0.121 mmol) and triethylamine (100 μL, 0.723 mmol) in CH_2Cl_2 (2 mL), was slowly added phenylacetyl chloride (48 μL, 0.361 mmol) in CH_2Cl_2 at 0 °C. After the resulting mixture was stirred 0 °C under argon atmosphere for 1 h, aq. $NaHCO_3$ and CH_2Cl_2 were added. The product was partitioned between the aqueous and organic layers. The aqueous layer was washed with CH_2Cl_2 and combined organic layers were dried over sodium sulfate and concentrated under vacuum. The crude material was purified by column chromatography on silica gel (25% Et_2O in Hexane) to afford **13a** (122 mg, 0.200 mmol, 83%) as colorless oil.

^{1}H NMR (500 MHz; $CDCl_3$): δ 7.33-7.26 (m, 5H), 7.22 (d, *J* = 8.7 Hz, 2H), 6.88 (d, *J* = 8.7 Hz, 2H), 4.71 (s, 2H), 4.52 (d, *J* = 11.7 Hz, 1H), 4.46 (d, *J* = 11.7 Hz, 1H), 4.35 (d, *J* = 12.4 Hz, 1H), 4.14 (d, *J* = 12.4 Hz, 1H), 3.97 (d, *J* = 11.9 Hz, 1H), 3.94 (d, *J* = 11.9 Hz, 1H), 3.81 (s, 3H), 3.66 (s, 2H), 3.00-1.50 (m, 9H), 0.88 (s, 9H), 0.05 (d, *J* = 3.7 Hz, 1H); ^{13}C NMR (125 MHz; $CDCl_3$): δ 170.9, 159.7, 133.7, 129.4, 128.82, 128.78, 127.4, 114.1, 73.4, 69.7, 63.0, 55.4, 53.3, 41.1, 25.8, 18.3, -5.50, -5.51; ^{11}B NMR (160 MHz; $CDCl_3$): δ –2.5, -3.6, -10.8, -11.8; HRMS (ESI, positive) for $C_{29}H_{46}B_{10}O_5Si$ (m/z): calculated 635.3980 $(M+Na)^+$, found 635.3992.

Synthesis of 1-(3-(4-methoxybenzyl)hydroxymethyl)-2-((*tert*-butyl)dimethylsilyl)-hydroxymethyl)-4-(3-((3-methylbutanoyl)oxy)prop-1-yn-1-yl)-1,2-dicarba-*closo*-dodecaborane (13b)

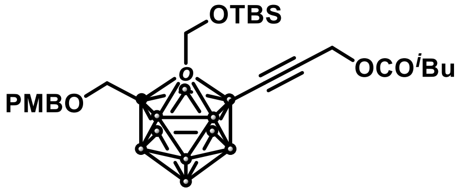

13b

To a solution of **12** (186 mg, 0.377 mmol) and triethylamine (157 µL, 1.13 mmol) in CH_2Cl_2 (2 mL), was slowly added isovaleryl chloride (69 µL, 0.566 mmol) in CH_2Cl_2 at 0 °C. After the resulting mixture was stirred 0 °C under argon atmosphere for 1 h, aq. $NaHCO_3$ and CH_2Cl_2 were added. The product was partitioned between the aqueous and organic layers. The aqueous layer was washed with CH_2Cl_2 and combined organic layers were dried over sodium sulfate and concentrated under vacuum. The crude material was purified by column chromatography on silica gel (20% EtOAc in Hexane) to afford **13b** (225 mg, quant.) as colorless oil.

^{1}H NMR (400 MHz; $CDCl_3$): δ 7.21 (d, *J* = 8.7 Hz, 2H), 6.88 (d, *J* = 8.7 Hz, 2H), 4.68 (s, 2H), 4.51 (d, *J* = 11.7 Hz, 1H), 4.45 (d, *J* = 11.7 Hz, 1H), 4.38 (d, *J* = 12.4 Hz, 1H), 4.16 (d, *J* = 12.4 Hz, 1H), 3.98 (d, *J* = 11.9 Hz, 1H), 3.94 (d, *J* = 11.9 Hz, 1H), 3.81 (s, 3H), 3.00-1.50 (m, 9H), 2.21 (d, *J* = 6.8 Hz, 2H), 2.15-2.10 (m, 1H), 0.96 (d, *J* = 6.6 Hz, 6H), 0.89 (s, 9H), 0.08 (d, *J* = 1.7 Hz, 1H); ^{13}C NMR (100 MHz; $CDCl_3$): δ 172.2, 159.7, 129.3, 128.8, 114.1, 92.8, 78.8, 77.3, 76.6, 73.4, 49.7, 63.0, 55.4, 52.5, 43.2, 25.9, 25.8, 22.5, 18.3, -5.53, -5.55; ^{11}B NMR (128 MHz; $CDCl_3$): δ -3.8, -11.0, -11.8; HRMS (ESI, positive) for $C_{26}H_{48}B_{10}O_5Si$ (m/z): calculated 601.4134 $(M+Na)^+$, found 601.4136.

Synthesis of 1,2-bis(hydroxymethyl)-4-ethynyl-1,2-dicarba-*closo*-dodecaborane (14)

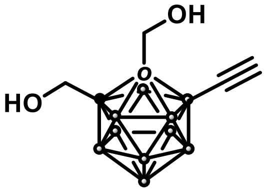

14

To a solution of **11** (18.1 mg, 51.9 µmol) and NaH_2PO_4 (12.5 mg, 0.104 mmol) in DCM/H_2O (1 mL, 10:1, v/v) was added DDQ (24.0 mg, 0.104 mmol) at 0 °C under argon atmosphere. After the resulting mixture was stirred at room temperature until the starting material disappeared as monitored by TLC analysis. Then, the reaction mixture was concentrated under pressure. Then, the reaction mixture was quenched with a small amount of aq. $NaHCO_3$ and filtered on celite pad. The filtrate was concentrated under vacuum. The crude material was purified by column chromatography on silica gel (25% EtOAc in Hexane) to afford **14** (10 mg, 43.8 µmol, 84%) as white solid. m.p. 167-168 °C; ^{1}H NMR (500 MHz; $CDCl_3$): δ 4.47 (d, *J* = 4.8 Hz, 1H), 4.44 (d, *J* = 4.7 Hz, 1H), δ 4.39 (d, *J* = 7.5 Hz, 1H), 4.37 (d, *J* = 7.6 Hz, 2H), 4.22 (d, *J* = 6.0 Hz, 1H), 3.20-1.60 (m, 9H), 2.94 (t, *J* = 6.9 Hz, 1H), 2.85 (d, *J* = 6.9 Hz, 1H), 2.36 (s, 1H); ^{13}C NMR (125 MHz; $CDCl_3$): δ 87.4, 78.6, 64.4, 63.6; ^{11}B NMR (128 MHz; $CDCl_3$): δ -2.2, -2.9, -3.7, -9.1, -10.1, -11.4, -12.3; HRMS (ESI, positive) for $C_6H_{16}B_{10}O_2$ (m/z): calculated 253.1985 $(M+Na)^+$, found 253.1982.

Synthesis of 1-(2-phenylacetoxymethyl)-2-((*tert*-butyl)dimethylsilyl)-hydroxymethyl)-4-(2-phenylacetoxy)prop-1-yn-1-yl)-1,2-dicarba-*closo*-dodecaborane (15a)

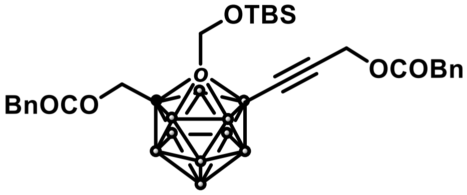

15a

To a solution of **13a** (62.9 mg, 0.103 mmol) and NaH_2PO_4 (24.7 mg, 0.206 mmol) in DCM/H_2O (1 mL, 10:1, v/v) was added DDQ (46.7 mg, 0.206 mmol) at 0 °C under argon atmosphere. After the resulting mixture was stirred at room temperature until the starting material disappeared as monitored by TLC analysis. Then, the reaction mixture was concentrated under pressure. Then, the reaction mixture was quenched with a small amount of aq. $NaHCO_3$ and filtered on celite pad. The filtrate was concentrated under vacuum. The crude material was shortly purified by column chromatography on silica gel (20% to 40% EtOAc in Hexane) to use in the next step without further purification.

To a solution of crude material and triethylamine (43 µL, 0.309 mmol) in CH_2Cl_2 (1 mL), was slowly added phenylacetyl

chloride (20 μL, 0.155 mmol) in CH_2Cl_2 at 0 °C. After the resulting mixture was stirred 0 °C under argon atmosphere for 1 h, aq. $NaHCO_3$ and CH_2Cl_2 were added. The product was partitioned between the aqueous and organic layers. The aqueous layer was washed with CH_2Cl_2 and combined organic layers were dried over sodium sulfate and concentrated under vacuum. The crude material was purified by column chromatography on silica gel (25% Et_2O in Hexane) to afford **15a** (39.7 mg, 65.2 μmol, 63%, 2 steps) as colorless oil.

^{1}H NMR (500 MHz; $CDCl_3$): δ 7.37-7.26 (m, 10H), 4.73-4.71 (m, 3H), 6.88 (d, *J* = 8.7 Hz, 2H), 4.64 (d, *J* = 13.1 Hz, 1H), 4.32 (d, *J* = 12.6 Hz, 1H), 4.20 (d, *J* = 12.6 Hz, 1H), 3.67 (d, *J* = 3.2 Hz, 1H), 3.00-1.50 (m, 9H), 0.89 (s, 9H), 0.08 (d, *J* = 1.1 Hz, 6H); ^{13}C NMR (125 MHz; $CDCl_3$): δ 170.8, 169.9, 133.6, 132.9, 129.5, 129.4, 128.9, 128.8, 127.7, 127.4, 92.8, 79.4, 74.3, 63.5, 62.8, 53.1, 41.10, 41.08, 25.7, 18.2, -5.53, -5.56; ^{11}B NMR (160 MHz; $CDCl_3$): δ -2.1, -4.0, -11.4; HRMS (ESI, positive) for $C_{29}H_{44}B_{10}O_3Si$ (m/z): calculated 633.3823 $(M+Na)^+$, found 633.3837.

Synthesis of 1-((3-methylbutanoyl)oxymethyl)-2-((*tert*-butyl)dimethylsilyl)-hydroxymethyl)-4-(2-phenylacetoxy)prop-1-yn-1-yl)-1,2-dicarba-*closo*-dodecaborane (15b)

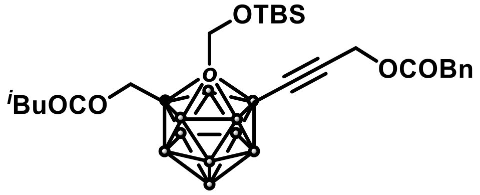

15b

This compound was prepared from **13a** (53.1 mg, 86.9 μmol) and 2.0 equivalent of isovaleryl chloride using the procedure described before for **15a** in 80% yield as colorless oil.

^{1}H NMR (500 MHz; $CDCl_3$):δ 7.33-7.28 (m, 5H), 4.72 (m, 3H), 4.63 (d, *J* = 13.1 Hz, 1H), 4.40 (d, *J* = 12.5 Hz, 1H), 4.25 (d, *J* = 12.5 Hz, 1H), 3.66 (s, 2H), 2.25 (d, *J* = 7.1 Hz, 2H), 2.90-1.60 (m, 9H), 2.16-2.09 (m, 1H), 0.98 (d, *J* = 6.6 Hz, 6H), 0.90 (s, 9H), 0.10 (s, 6H); ^{13}C NMR (125 MHz; $CDCl_3$): δ 171.5, 170.8, 133.6, 129.4, 128.8, 127.4, 79.5, 74.6, 63.5, 62.4, 53.2, 43.0, 41.1, 25.7, 25.6, 22.5, 18.3, -5.51, -5.54; ^{11}B NMR (160 MHz; $CDCl_3$): δ -2.3, -4.1, -11.4; HRMS (ESI, positive) for $C_{26}H_{46}B_{10}O_3Si$ (m/z): calculated 599.3977 $(M+Na)^+$, found 599.3971.

Synthesis of 1-(2-phenylacetoxymethyl)-2-((*tert*-butyl)dimethylsilyl)-hydroxymethyl)-4-((3-methylbutanoyl)prop-1-yn-1-yl)-1,2-dicarba-*closo*-dodecaborane (15c)

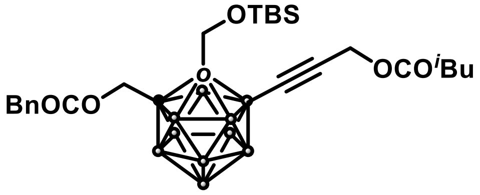

15c

This compound was prepared from **13b** (66.0 mg, 144.5 μmol) and benzyl chloride using the procedure described before for **13a** in 76% yield as colorless oil.

^{1}H NMR (500 MHz; $CDCl_3$):δ 7.37-7.26 (m, 5H), 4.71 (d, *J* = 13.1 Hz, 1H), 4.68 (s, 2H), 4.64 (d, *J* = 13.1 Hz, 1H), 4.34 (d, *J* = 12.5 Hz, 1H), 4.21 (d, *J* = 12.5 Hz, 1H), 3.67 (s, 2H), 2.23 (d, *J* = 7.0 Hz, 2H), 2.90-1.60 (m, 9H), 2.15-2.10 (m, 1H), 0.97 (d, *J* = 6.6 Hz, 6H), 0.90 (s, 9H), 0.10 (d, *J* = 3.0 Hz, 6H); ^{13}C NMR (125 MHz; $CDCl_3$): δ 172.3, 169.9, 132.9, 129.5, 128.9, 127.7, 93.1, 79.4, 74.3, 63.5, 62.8, 52.5, 43.2, 41.1, 25.9, 25.7, 22.5, 18.3, -5.5, -5.6; ^{11}B NMR (160 MHz; $CDCl_3$): δ -2.1, -4.1, -11.4; HRMS (ESI, positive) for $C_{26}H_{46}B_{10}O_3Si$ (m/z): calculated 599.3977 $(M+Na)^+$, found 599.3972.

Synthesis of 1-(3-methylbutanoyl)-2-((*tert*-butyl)dimethylsilyl)-hydroxymethyl)-4-((3-methylbutanoyl)prop-1-yn-1-yl)-1,2-dicarba-*closo*-dodecaborane (15d)

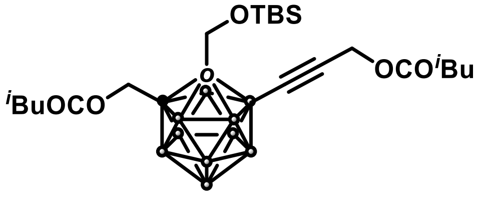

15d

This compound was prepared from **13b** (66.0 mg, 144.5 µmol) and isovaleryl chloride using the procedure described before for **13a** in 76% yield as colorless oil.
^{1}H NMR (500 MHz; $CDCl_3$):δ 4.70 (d, J = 13.1 Hz, 1H), 4.68 (s, 2H), 4.63 (d, J = 13.1 Hz, 1H), 4.41 (d, J = 12.5 Hz, 1H), 4.27 (d, J = 12.5 Hz, 1H), 2.24 (d, J = 7.1 Hz, 2H), 2.22 (d, J = 7.0 Hz, 2H), 2.90-1.60 (m, 9H), 2.14-2.09 (m, 2H), 0.97 (d, J = 6.6 Hz, 6H), 0.96 (d, J = 6.6 Hz, 6H), 0.91 (s, 9H), 0.12 (d, J = 1.3 Hz, 6H); ^{13}C NMR (125 MHz; $CDCl_3$): δ 172.3, 171.5, 93.2, 79.5, 74.6, 63.5, 62.3, 52.5, 43.2, 43.0, 25.9, 25.7, 25.6, 22.53, 22.48, 18.3, -5.5, -5.6; ^{11}B NMR (160 MHz; $CDCl_3$): δ -2.2, -4.1, -11.4; HRMS (ESI, positive) for $C_{23}H_{48}B_{10}O_3Si$ (m/z): calculated 565.4131 $(M+Na)^+$, found 565.4128.

Synthesis of 1,2-bis(hydroxymethyl)-4-(2-phenylacetoxy)prop-1-yn-1-yl)-1,2-dicarba-*closo*-dodecaborane (16a)

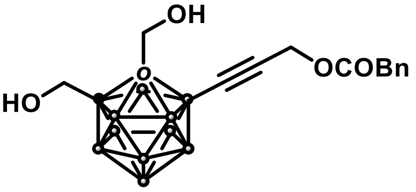

16a

To a solution of **13a** (66 mg, 0.109 mmol) in CH_2Cl_2 (1 mL), was added 4N HCl in Dioxane (1 mL). The resulting mixture was stirred at room temperature until the full conversion of a starting material was observed. After that, the reaction mixture was concentrated under pressure. The crude material was purified by column chromatography on silica gel (25% to 50% EtOAc in Hexane) to afford **16a** (24.7 mg, 0.0656 mmol, 60%) as colorless oil.
^{1}H NMR (500 MHz; $CDCl_3$): δ 7.35-7.29 (m, 5H), 4.69 (d, J = 15.7 Hz, 1H), 4.64 (d, J = 15.7 Hz, 1H), 4.27 (d, J = 13.9 Hz, 1H), 4.16-4.13 (m, 3H), 3.68 (s, 2H), 3.00-1.50 (m, 9H); ^{13}C NMR (100 MHz; $CDCl_3$): δ 172.3, 133.4, 129.3, 129.0, 127.6, 92.3, 79.1, 78.8, 64.2, 63.1, 53.7, 41.3; ^{11}B NMR (128 MHz; $CDCl_3$): δ -3.24, -9.97, -10.9, 11.6; HRMS (ESI, positive) for $C_{15}H_{24}B_{10}O_4$ (m/z): calculated 401.2522 $(M+Na)^+$, found 401.2519.

Synthesis of 1,2-bis(hydroxymethyl)-4-((3-methylbutanoyl)prop-1-yn-1-yl)-1,2-dicarba-*closo*-dodecaborane (16b)

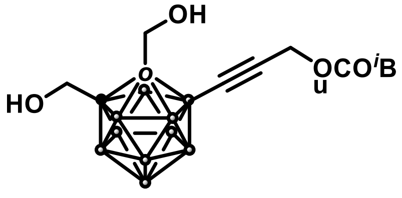

16b

To a solution of **13b** (70 mg, 0.121 mmol) in CH_2Cl_2 (1 mL), was added 4N HCl in Dioxane (1 mL). The resulting mixture was stirred at room temperature until the full conversion of a starting material was observed. After that, the reaction mixture was concentrated under pressure. The crude material was purified by column chromatography on silica gel (25% to 50% EtOAc in Hexane) to afford **16b** (36.7 mg, 0.106 mmol, 87%) as colorless oil.
^{1}H NMR (500 MHz; $CDCl_3$): δ 4.67 (d, J = 15.7 Hz, 1H), 4.60 (d, J = 15.7 Hz, 1H), 4.44 (d, J = 13.8 Hz, 1H), 4.29 (d, J = 13.8 Hz, 1H), 4.18 (d, J = 2.7 Hz, 2H), 3.00-1.50 (m, 9H), 2.24 (t, J = 3.9 Hz, 2H) 2.14-2.08 (m, 1H), 0.97 (d, J = 6.7 Hz, 6H); ^{13}C NMR (125 MHz; $CDCl_3$): δ 173.9, 92.9, 79.1, 78.8, 64.2, 63.3, 53.0, 43.3, 26.0, 22.4; ^{11}B NMR (128 MHz; $CDCl_3$): δ -3.24, -9.97, -10.9, 11.6; HRMS (ESI, positive) for $C_{12}H_{26}B_{10}O_4$ (m/z): calculated 367.2675 $(M+Na)^+$, found 367.2683.

Synthesis of 1-((3-methylbutanoyl)oxymethyl)-2-((3-methylbutanoyl)oxymethyl)-4-(2-phenylacetoxy)prop-1-yn-1-yl)-1,2-dicarba-*closo*-dodecaborane (IIa)

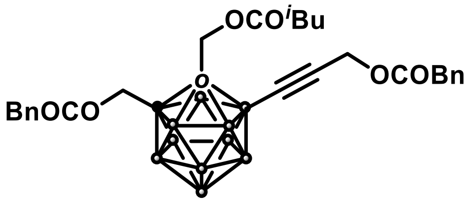

IIa

To a solution of **15a** (27 mg, 44.3 μmol) in CH_2Cl_2 (500 μL), was added 4N HCl in Dioxane (500 μL). The resulting mixture was stirred at room temperature until the full conversion of a starting material was observed. After that, the reaction mixture was concentrated under pressure to use in the next reaction without further purification.

To a solution of the crude material and triethylamine (18 μL, 133 μmol) in CH_2Cl_2 (500 μL), was slowly added isovaleryl chloride (8.1 μL, 66.5 μmol) in CH_2Cl_2 at 0 °C. After the resulting mixture was stirred at room temperature for 9 h, the resulting mixture was concentrated under vacuum. The crude materials were filtered through basic Al_2O_3 pad and purified by column chromatography on silica gel (25% Et_2O in hexane) afforded **IIa** (15.6 mg, 27.0 μmol, 61% 2 steps) as colorless oil.

^{1}H NMR (500 MHz; $CDCl_3$): δ 7.33-7.26 (m, 10H), 4.75-4.67 (m, 6H), 3.68 (s, 2H), 3.67 (s, 2H), 2.20 (d, *J* = 7.1 Hz, 1H), 2.11-2.06 (m, 1H), 3.00-1.50 (m, 9H), 0.96 (d, *J* = 6.6 Hz, 6H); ^{13}C NMR (125 MHz; $CDCl_3$): δ 171.2, 170.8, 169.8, 133.7, 132.7, 129.5, 129.4, 129.0, 128.8, 127.8, 127.4, 75.8, 75.3, 63.1, 61.4, 53.0, 42.9, 41.1, 41.0, 25.6, 22.5; ^{11}B NMR (160 MHz; $CDCl_3$): δ -3.1, -11.0; HRMS (ESI, positive) for $C_{28}H_{38}B_{10}O_6$ (m/z): calculated 603.3532 $(M+Na)^+$, found 603.3532.

Synthesis of 1-((3-methylbutanoyl)oxymethyl)-2-(2-phenylacetoxymethyl)-4-((3-methylbutanoyl)prop-1-yn-1-yl)-1,2-dicarba-*closo*-dodecaborane (IIb)

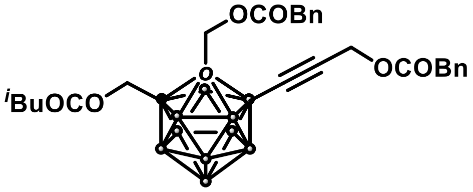

IIb

This compound was prepared from **15b** (39.7 mg, 69.1 μmol) and benzyl chloride using the procedure described before for **IIa** in 43% yield as colorless oil.

^{1}H NMR (500 MHz; $CDCl_3$): δ 7.35-7.27 (m, 10H), 4.85 (m, 6H), 3.68 (d, *J* = 13.7 Hz, 1H), 4.80 (d, *J* = 13.7 Hz, 1H), 4.68 (s, 2H), 4.51 (d, *J* = 1.0 Hz, 1H), 3.67 (s, 2H), 3.66 (s, 2H), 3.00-1.50 (m, 9H), 2.20 (d, *J* = 7.0 Hz, 2H), 2.11-2.07 (m, 1H), 0.96 (d, *J* = 6.6 Hz, 6H); ^{13}C NMR (125 MHz; $CDCl_3$): δ 171.2, 170.9, 169.8, 133.7, 132.9, 129.5, 129.4, 128.9, 128.8, 127.7, 127.4, 75.61, 75.57, 62.4, 62.0, 53.0, 42.8, 41.1, 41.0, 25.6, 22.5; ^{11}B NMR (160 MHz; $CDCl_3$): δ -3.1, -11.1; HRMS (ESI, positive) for $C_{28}H_{38}B_{10}O_6$ (m/z): calculated 603.3532 $(M+Na)^+$, found 603.3529.

Synthesis of 1-(2-phenylacetoxymethyl)-2-((3-methylbutanoyl)oxymethyl)-4-((3-methylbutanoyl)prop-1-yn-1-yl)-1,2-dicarba-*closo*-dodecaborane (IIc)

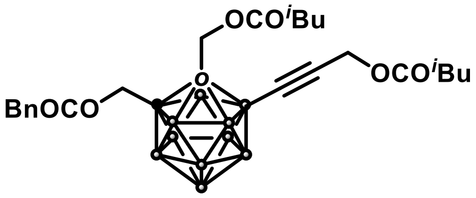

IIc

This compound was prepared from **15c** (29.8 mg, 51.8 μmol) and isovaleryl chloride using the procedure described before for **IIa** in 89% yield as colorless oil.

^{1}H NMR (500 MHz; $CDCl_3$):δ 7.37-7.25 (m, 5H), 4.73 (d, *J* = 3.8 Hz, 2H), 4.68 (m, 4H), 3.67 (s, 2H), 2.90-1.60 (m, 9H), 2.24-2.20 (m, 4H), 2.14-2.07 (m, 2H), 0.98-0.96 (m, 12H); ^{13}C NMR (125 MHz; $CDCl_3$): δ 172.3, 171.2, 169.8, 132.8, 129.5, 129.0, 127.8, 94.0, 75.9, 75.4, 63.1, 61.5, 52.3, 43.2, 42.9, 41.1, 25.9, 25.6, 22.5; ^{11}B NMR (160 MHz; $CDCl_3$): δ -3.1, -11.0; HRMS (ESI, positive) for $C_{25}H_{40}B_{10}O_6$ (m/z): calculated 569.3685 $(M+Na)^+$, found 569.3680.

Synthesis of 1-(3-methylbutanoyl)-2-(2-phenylacetoxymethyl)-4-((3-methylbutanoyl)prop-1-yn-1-yl)-1,2-dicarba-*closo*-dodecaborane (IId)

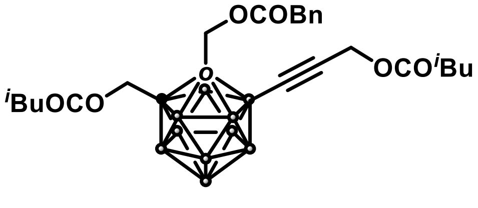

IId

This compound was prepared from **15d** (32.3 mg, 59.7 μmol) and benzyl chloride using the procedure described before for **IIa** in 71% yield as colorless oil.
^{1}H NMR (500 MHz; $CDCl_3$):δ 7.36-7.27 (m, 5H), 4.85 (d, J = 3.1 Hz, 1H), 4.66 (s, 2H), 4.52 (d, J = 0.9 Hz, 1H), 3.69 (s, 2H), 2.90-1.60 (m, 9H), 2.29-2.18 (m, 4H), 2.15-2.05 (m, 2H), 0.96 (d, J = 6.6 Hz, 12H); ^{13}C NMR (125 MHz; $CDCl_3$): δ 172.4, 171.2, 169.8, 132.9, 129.5, 128.9, 127.8, 75.6, 75.5, 62.4, 62.1, 52.4, 43.2, 42.8, 41.0, 25.9, 25.6, 22.49, 22.48; ^{11}B NMR (160 MHz; $CDCl_3$): δ -2.9, -11.1; HRMS (ESI, positive) for $C_{25}H_{40}B_{10}O_6$ (m/z): calculated 569.3685 $(M+Na)^+$, found 569.3681.

Synthesis of 1,2-bis(phenylacetoxymethyl)-4-(2-phenylacetoxy)prop-1-yn-1-yl)-1,2-dicarba-*closo*-dodecaborane (IIe)

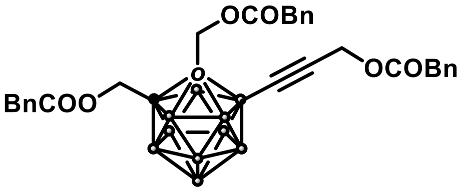

IIe

This compound was prepared from **16a** (11.9 mg, 31.6 μmol) and 2.0 equivalent of phenylacetyl chloride using the procedure described before for **13a** in 57% yield as colorless oil.
^{1}H NMR (500 MHz; $CDCl_3$):δ 7.33-7.21 (m, 15H), 4.68 (d, J = 4.3 Hz, 4H), 4.49 (s, 2H), 3.66 (s, 2H), 3.61 (s, 4H), 2.90-1.40 (m, 9H); ^{13}C NMR (125 MHz; $CDCl_3$): δ 170.8, 169.7, 169.7, 133.7, 132.9, 132.7, 129.51, 129.49, 129.41, 129.0, 128.9, 128.8, 127.7, 127.4, 93.5, 75.4, 75.1, 63.0, 62.1, 53.0, 41.1, 41.0; ^{11}B NMR (160 MHz; $CDCl_3$): δ -3.10, -11.4; HRMS (ESI, positive) for $C_{31}H_{36}B_{10}O_6$ (m/z): calculated 637.3378 $(M+Na)^+$, found 637.3386.

Synthesis of 1,2-bis((3-methylbutanoyl)oxymethyl)-4-(2-phenylacetoxy)prop-1-yn-1-yl)-1,2-dicarba-*closo*-dodecaborane (IIf)

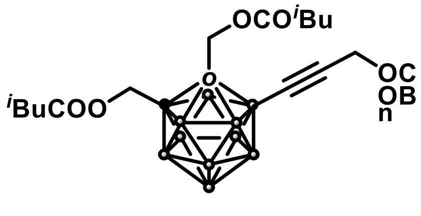

IIf

This compound was prepared from **16a** (12.6 mg, 33.5 μmol) and 2.0 equivalent of isovaleryl chloride using the procedure described before for **13b** in 23% yield as colorless oil.
^{1}H NMR (500 MHz; $CDCl_3$):δ 7.35-7.27 (m, 5H), 4.87 (d, J = 13.8 Hz, 1H), 4.79 (d, J = 13.8 Hz, 1H), 4.71 (s, 2H), 4.70-4.65 (m, 2H), 3.68 (s, 2H), 2.90-1.60 (m, 9H), 2.25 (d, J = 7.1 Hz, 4H) 2.16-2.07 (m, 2H), 0.99-0.97 (m, 12H); ^{13}C NMR (125 MHz; $CDCl_3$): δ 171.3, 171.3, 170.8, 133.7, 129.4, 128.8, 127.4, 75.9, 75.7, 62.5, 61.4, 53.0, 42.92, 42.89, 41.1, 25.62, 25.60, 22.5; ^{11}B NMR (160 MHz; $CDCl_3$): δ -3.15, -11.1; HRMS (ESI, positive) for $C_{25}H_{40}B_{10}O_6$ (m/z): calculated 569.3685 $(M+Na)^+$, found 569.3684.

Synthesis of 1,2-bis(2-phenylacetoxymethyl)-4-((3-methylbutanoyl)prop-1-yn-1-yl)-1,2-dicarba-*closo*-dodecaborane (IIg)

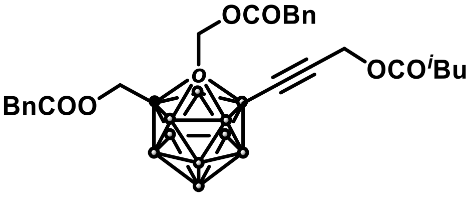

IIg

This compound was prepared from **16b** (18.6 mg, 54.3 μmol) and 2.0 equivalent of phenylacetyl chloride using the procedure described before for **13a** in 72% yield as colorless oil.
^{1}H NMR (500 MHz; $CDCl_3$):δ 7.36-7.28 (m, 6H), 7.25-7.22 (m, 4H), 4.71 (s, 2H), 4.66 (s, 2H), 4.50 (s, 2H), 3.63 (s, 2H), 3.61 (s, 2H), 2.90-1.40 (m, 9H), 2.23 (d, *J* = 7.1 Hz, 2H), 2.16-2.09 (m, 1H), 0.97 (d, *J* = 6.6 Hz, 6H); ^{13}C NMR (125 MHz; $CDCl_3$): δ 172.3, 169.70, 169.69, 132.9, 129.50, 125.49, 128.93, 128.89, 127.8, 127.7, 93.9, 75.4, 75.2, 63.0, 62.1, 52.3, 43.1, 41.0, 25.9, 22.5; ^{11}B NMR (160 MHz; $CDCl_3$): δ -2.9, -10.3, -11.0; HRMS (ESI, positive) for $C_{28}H_{38}B_{10}O_6$ (m/z): calculated 603.3532 $(M+Na)^+$, found 603.3521.

Synthesis of 1,2-bis((3-methylbutanoyl)oxymethyl)-4-((3-methylbutanoyl)prop-1-yn-1-yl)-1,2-dicarba-*closo*-dodecaborane (IIh)

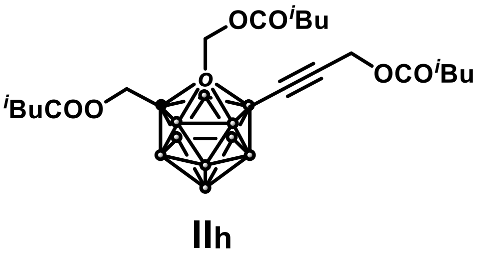

IIh

This compound was prepared from **16b** (19.4 mg, 56.7 μmol) and 2.0 equivalent of isovaleryl chloride using the procedure described before for **13b** in 88% yield as colorless oil.
^{1}H NMR (500 MHz; $CDCl_3$):δ 4.88 (d, *J* = 13.7 Hz, 1H), 4.82 (d, *J* = 13.7 Hz, 1H), 4.68-4.67 (m, 4H), 2.90-1.60 (m, 9H), 2.26-2.22 (m, 6H) 2.16-2.08 (m, 3H), 0.98-0.95 (m, 18H); ^{13}C NMR (125 MHz; $CDCl_3$): δ 172.3, 171.3, 94.0, 75.8, 75.7, 62.5, 43.1, 42.92, 42.88, 25.9, 25.61, 25.60, 22.51, 22.50, 22.47; ^{11}B NMR (160 MHz; $CDCl_3$): δ -3.0, -10.3, -11.0; HRMS (ESI, positive) for $C_{25}H_{40}B_{10}O_6$ (m/z): calculated 535.3839 $(M+Na)^+$, found 535.3839.

➢ Synthesis of scaffold III-type compounds

Synthesis of 1-hydroxymethyl-9-((trimethylsilyl)ethynyl)-1,7-dicarba-*closo*-dodecaborane (S5)

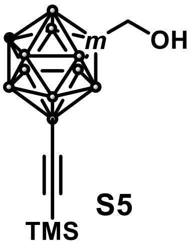

S5

To a solution of 9-((trimethylsilyl)ethynyl)-1,7-dicarba-*closo*-dodecaborane (238 mg, 0.989 mmol) prepared according to the reported literature[3] in THF (4 mL), was slowly added *n*-BuLi 1.6 M solution in hexane (622 μL, 0.989 mmol) at -78 °C. After the resulting mixture was stirred at -78 °C under argon atmosphere for 2 h, paraformaldehyde (44 mg, 1.48 mmol) was added at -78 °C. Then, the resulting mixture was warmed up to room temperature and stirred for 12 h under argon atmosphere. The reaction mixture was quenched with aq. 1N HCl. The resulting mixture was partitioned between the aqueous and organic layers. The aqueous layer was washed with EtOAc, and the mixture was extracted with EtOAc, washed with brine, dried over sodium sulfate, and concentrated under vacuum. The residue was purified by column chromatography on silica gel (20% EtOAc in Hexane) to afford **S5** (122 mg, 0.452 mmol, 46%) as colorless oil.
^{1}H NMR (500 MHz; $CDCl_3$): δ 3.81 (s, 2H), 2.93 (s, 1H), 3.40-1.60 (m, 9H), 0.15 (s, 9H); ^{13}C NMR (125 MHz; $CDCl_3$): δ 105.1, 76.2, 65.3, 53.6, 21.2, 0.21; ^{11}B NMR (160 MHz; $CDCl_3$): δ -4.3, -8.6, -10.0, -11.5, -12.8, -14.1, -16.7, -18.3; HRMS (ESI, positive) for $C_8H_{22}B_{10}OSi$ (m/z): calculated 295.2281 $(M+Na)^+$, found 295.2273.

Synthesis of 1-((*tert*-butyl)dimethylsilyl)hydroxymethyl)-9-((trimethylsilyl)ethynyl)-1,7-dicarba-*closo*-dodecaborane (17)

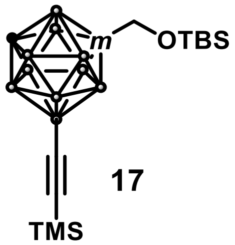

17

To a solution of **S5** (132 mg, 0.487 mmol) and 2,6-lutidine (85 µL, 0.730 mmol) in DCM (2 mL) was slowly added TBSOTf (132 µL, 0.584 mmol) at 0 °C under argon atmosphere. The resulting mixture was stirred at room temperature until the starting material disappeared as monitored by TLC analysis. Then, the reaction mixture was quenched with a small amount of aq. $NaHCO_3$ and filtered on celite pad. The filtrate was concentrated under vacuum. The crude material was purified by column chromatography on silica gel (10% EtOAc in Hexane) to afford **17** (185 mg, 0.480 mmol, 99%) as colorless oil. ^{1}H NMR (500 MHz; $CDCl_3$): δ 3.81 (s, 2H), 2.93 (s, 1H), 3.40-1.60 (m, 9H), 0.15 (s, 9H); ^{13}C NMR (125 MHz; $CDCl_3$): δ 105.1, 76.2, 65.3, 53.6, 21.2, 0.21; ^{11}B NMR (160 MHz; $CDCl_3$): δ -4.3, -8.6, -10.0, -11.5, -12.8, -14.1, -16.7, -18.3; HRMS (ESI, positive) for $C_{14}H_{36}B_{10}OSi_2$ (m/z): calculated 409.3156 $(M+Na)^+$, found 409.3153.

Synthesis of 7-((*tert*-butyl)dimethylsilyl)hydroxymethyl-9-(trimethylsilyl)ethynyl-1,7-dicarba-*closo*-dodecaboranylcarboxylic acid (18)

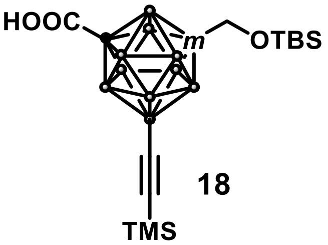

18

To a solution of **17** (184 mg, 0.478 mmol) in THF (1.8 mL), was slowly added *n*-BuLi 1.6 M solution in hexane (316 µL, 0.502 mmol) at -78 °C. After the resulting mixture was stirred at -78 °C under argon atmosphere for 2 h, CO_2 balloon was attached. Then, the resulting mixture was warmed up to room temperature and stirred for 12 h under CO_2 atmosphere. After that, the reaction mixture was concentrated under pressure. Hexane was added after which the product was partitioned between the aqueous and organic layers. The aqueous layer was washed with hexane. Then, to the aqueous layer, were added 6 M HCl and Et_2O after which the product was partitioned between the aqueous and organic layers. The aqueous layer was washed with hexane and combined organic layers were dried over sodium sulfate and concentrated under vacuum to afford **18** (188 mg, 92%) as a white solid.
^{1}H NMR (500 MHz; $CDCl_3$): δ 3.75 (s, 2H), 3.10-1.80 (m, 9H), 0.89 (s, 9H), 0.16 (s, 9H), 0.04 (s, 6H); ^{13}C NMR (125 MHz; $CDCl_3$): δ 165.7, 105.9, 79.3, 69.2, 65.5, 25.8, 18.4, 0.18, -5.5; ^{11}B NMR (160 MHz; $CDCl_3$): δ -3.9, -6.8, -10.4, -12.7, -17.1; HRMS (ESI, positive) for $C_{15}H_{36}B_{10}O_3Si_2$ (m/z): calculated 453.3056 $(M+Na)^+$, found 453.3051; m.p. 119-120 °C.

Synthesis of 1-((*tert*-butyl)dimethylsilyl)hydroxymethyl)-7-benzylaminocarboxyl-9-ethynyl-1,7-dicarba-*closo*-dodecaborane (19a)

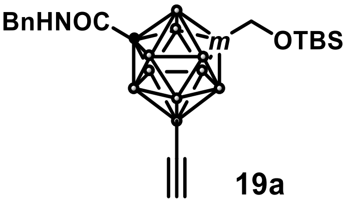

19a

To a solution of **18** (87.8 mg, 0.281 mmol) in THF (1 mL), benzylamine (37 µL, 0.337 mmol), EDCI (65 mg, 0.337 mmol), HOBt (65 mg, 0.422 mmol) and DIEA (143 µL, 0.843 mmol) were slowly added. The resulting mixture was stirred at room temperature under argon atmosphere until the full conversion of a starting material was observed. The resulting mixture was added aq. $NaHCO_3$ and EtOAc after which the product was partitioned between the aqueous and organic layers. The aqueous layer was washed with EtOAc and combined organic layers were washed with brine, dried over sodium sulfate and concentrated under vacuum. The crude material was used to the next reaction without further purification.
To a solution of the crude material in MeOH (500 µL), potassium carbonate (40 mg, 0.444 mmol) was added. The resulting mixture was stirred at room temperature until the full conversion of a starting material was observed. After that, the reaction mixture was concentrated under pressure. To the resulting mixture was added water and EtOAc after which

the product was partitioned between the aqueous and organic layers. The aqueous layer was washed with EtOAc and combined organic layers were washed with brine, dried over sodium sulfate and concentrated under vacuum. The crude material was purified by column chromatography on silica gel (10% EtOAc in Hexane) to afford **19a** (20.5 mg, 0.0460 mmol, 16% 2 steps) as colorless oil. ^{1}H NMR (500 MHz; $CDCl_3$): δ 7.36-7.17 (m, 4H), 7.18 (d, J = 7.0 Hz, 2H), 6.09 (s, 1H), 4.38 (d, J = 5.5 Hz, 2H), 3.78 (s, 2H), 3.40-1.60 (m, 9H), 2.27 (s, 1H), 0.89 (s, 9H), 0.04 (s, 6H); ^{13}C NMR (125 MHz; $CDCl_3$): δ 160.0, 136.8, 129.1, 128.1, 127.6, 86.4, 73.6, 65.5, 45.0, 43.9, 25.8, 18.3, -5.5; ^{11}B NMR (160 MHz; $CDCl_3$): δ -4.6, -7.9, -10.9, -12.5, -15.3, -16.6; HRMS (ESI, positive) for $C_{19}H_{35}B_{10}O_2NSi$ (m/z): calculated 470.3291 $(M+Na)^+$, found 470.3285.

Synthesis of 1-((*tert*-butyl)dimethylsilyl)hydroxymethyl-7-isobutylcarbamoyl-9-ethynyl-1,7-dicarba-*closo*-dodecaborane (19b)

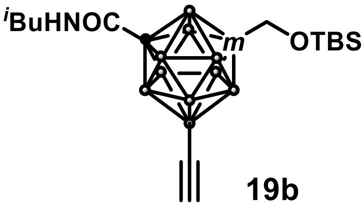

19b

This compound was prepared from **18** (102 mg, 0.327 mmol) using the procedure described for **19a** in 56% yield as a white solid. ^{1}H NMR (500 MHz; $CDCl_3$): δ 5.83 (s, 1H), 3.76 (s, 2H), 3.02 (t, J = 6.4 Hz, 2H), 3.40-1.80 (m, 9H), 2.27 (s, 1H), 1.79-1.70 (m, 1H), 0.88 (s, 9H), 0.87 (d, J = 6.8 Hz, 6H), 0.03 (s, 6H); ^{13}C NMR (125 MHz; $CDCl_3$): δ 159.9, 86.4, 76.5, 74.0, 65.5, 48.1, 28.5, 25.8, 19.9, 18.3, -5.5; ^{11}B NMR (160 MHz; $CDCl_3$): δ -4.9, -7.9, -11.0, -12.5, -15.5, -16.6; HRMS (ESI, positive) for $C_{16}H_{37}B_{10}NO_2Si$ (m/z): calculated 436.3444 $(M+Na)^+$, found 436.3423; m.p. 69-70 °C.

Synthesis of 1-((*tert*-butyl)dimethylsilyl)hydroxymethyl)-7-benzylaminocarboxyl-9-(1*H*-1,2,3-triazol-4-yl)benzylcarbamoyl-1,7-dicarba-*closo*-dodecaborane (20a)

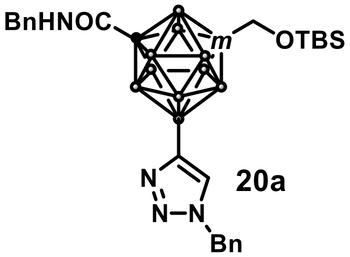

20a

To a solution of **19a** (17 mg, 38.4 μmol) in DMF:H_2O (1:1, v/v, 500 μL), were added CuI (0.7 mg, 3.84 μmol), sodium ascorbate (1.5 mg, 7.67 μmol), and benzylazide (5.1 μL, 38.4 μmol). The mixture was stirred vigorously at room temperature for 12 h. The reaction was quenched with water and the mixture was extracted with EtOAc, washed with brine, dried over sodium sulfate, and concentrated under vacuum. The residue was purified by column chromatography on silica gel (40% EtOAc in Hexane) to afford **20a** (15.5 mg, 26.8 μmol, 70%) as a white solid.

^{1}H NMR (500 MHz; $CDCl_3$):δ 7.37-7.26 (m, 10H), 7.18 (d, J = 7.0 Hz, 2H), 6.25 (s, 1H), 5.50 (s, 2H), 4.38 (d, J = 5.5 Hz, 2H), 3.81 (s, 2H), 3.50-1.80 (m, 9H), 0.88 (s, 9H), 0.04 (s, 6H); ^{13}C NMR (125 MHz; $CDCl_3$): δ 160.3, 136.9, 134.9, 129.2, 129.0, 128.7, 128.3, 128.0, 127.6, 74.1, 65.5, 53.8, 44.9, 29.8, 25.8, 18.3, -5.2; ^{11}B NMR (160 MHz; $CDCl_3$): δ -5.2, -8.1, -10.6, -12.2, -16.6; HRMS (ESI, positive) for $C_{26}H_{42}B_{10}N_4O_2Si$ (m/z): calculated 603.3938 $(M+Na)^+$, found 603.3939; m.p. 66-68 °C.

Synthesis of 1-((*tert*-butyl)dimethylsilyl)hydroxymethyl)-7-benzylaminocarboxyl-9-((1*H*-1,2,3-triazol-4-yl)-3-methyl-butanoyl)-1,7-dicarba-*closo*-dodecaborane (20b)

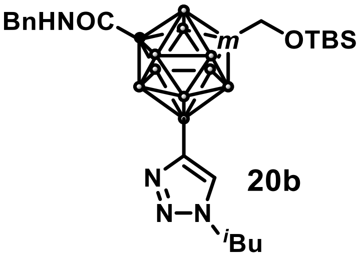

20b

This compound was prepared from **19a** (38.0 mg, 85.3 µmol) and isovalerylazide using the procedure described before for **20a** in 84% yield as a white solid; m.p. 49-51 °C; ^{1}H NMR (500 MHz; $CDCl_3$):δ 7.40 (s, 1H), 7.33-7.26 (m, 3H), 7.18 (d, *J* = 7.0 Hz, 2H), 6.24 (s, 1H), 4.39 (s, 2H), 4.12 (d, *J* = 5.7 Hz, 2H), 3.83 (s, 2H), 3.20-2.00 (m, 9H), 2.19 (m, 1H), 0.92 (d, *J* = 6.4 Hz, 6H), 0.89 (s, 9H), 0.05 (s, 6H); ^{13}C NMR (125 MHz; $CDCl_3$): δ 160.3, 136.9, 129.0, 128.0, 127.6, 74.0, 65.5, 57.2, 44.9, 29.8, 25.8, 20.1, 18.3, -5.5; ^{11}B NMR (160 MHz; $CDCl_3$): δ -5.1, -8.1, -12.9, -15.3; HRMS (ESI, positive) for $C_{23}H_{44}B_{10}N_4O_2Si$ (m/z): calculated 569.4092 $(M+Na)^+$, found 569.4075.

Synthesis of 1-((*tert*-butyl)dimethylsilyl)hydroxymethyl)-7-isobutylcarbamoyl-9-(1*H*-1,2,3-triazol-4-yl)benzylcarbamoyl-1,7-dicarba-*closo*-dodecaborane (20c)

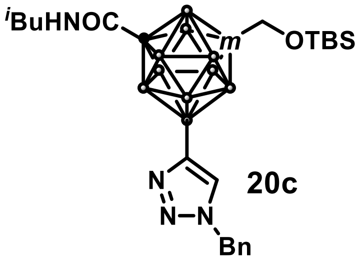

20c

This compound was prepared from **19b** (34.6 mg, 84.1 µmol) using the procedure described before for **20a** in 93% yield as colorless oil. ^{1}H NMR (500 MHz; $CDCl_3$):δ 7.37-7.23 (m, 5H), 5.94 (s, 1H), 5.50 (s, 2H), 3.80 (s, 2H), 3.02 (m, 2H), 3.00-1.80 (m, 9H), 0.87 (m, 15H), 0.03 (s, 6H); ^{13}C NMR (125 MHz; $CDCl_3$): δ 160.2, 135.0, 129.1, 128.7, 128.2, 74.4, 65.5, 48.2, 25.8, 20.0, 18.3, -5.5 ^{11}B NMR (160 MHz; $CDCl_3$): δ –5.1, -8.1, -12.2, -15.2; HRMS (ESI, positive) for $C_{23}H_{44}B_{10}N_4O_2Si$ (m/z): calculated 569.4092 $(M+Na)^+$, found 569.4078.

Synthesis of 1-((*tert*-butyl)dimethylsilyl)hydroxymethyl)-7-isobutylcarbamoyl-9-((1*H*-1,2,3-triazol-4-yl)-3-methyl-butanoyl)-1,7-dicarba-*closo*-dodecaborane (20d)

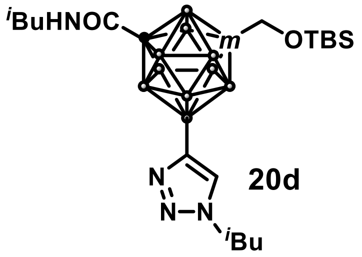

20d

This compound was prepared from **19b** (35.8 mg, 87.0 µmol) and isovalerylazide using the procedure described before for **20a** in 14% yield as colorless oil. ^{1}H NMR (500 MHz; $CDCl_3$):δ 7.43 (s, 1H), 6.04 (s, 1H), 4.14 (d, *J* = 7.1 Hz, 2H), 3.82 (s, 2H), 3.04 (m, 2H), 2.24-2.19 (m, 1H), 1.79-1.77 (m, 1H), 0.94 (d, *J* = 6.6 Hz, 6H), 0.89 (s, 9H), 0.87 (d, *J* = 6.7 Hz, 6H), 0.05 (s, 6H); ^{13}C NMR (125 MHz; $CDCl_3$): δ 160.3, 74.6, 65.6, 57.5, 48.2, 29.9, 28.5, 20.1, 20.0, 18.4, -5.4; ^{11}B NMR (160 MHz; $CDCl_3$): δ -5.4, -8.2, -10.6, -12.5, 16.4; HRMS (ESI, positive) for $C_{20}H_{46}B_{10}N_4O_2Si$ (m/z): calculated 535.4245 $(M+Na)^+$, found 535.4230.

Synthesis of 1-phenylacetoxymethyl-7-benzylaminocarboxyl-9-(1*H*-1,2,3-triazol-4-yl)benzylcarbamoyl-1,7-dicarba-*closo*-dodecaborane (IIIa)

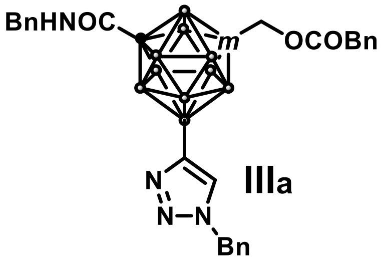

To a solution of **20a** (11.9 mg, 26.8 μmol) in CH_2Cl_2 (500 μL), was added 4N HCl in Dioxane (500 μL). The resulting mixture was stirred at room temperature until the full conversion of a starting material was observed. After that, the reaction mixture was concentrated under pressure to use in the next reaction without further purification.

To a solution of the crude material and triethylamine (16.7 μL, 0.121 mmol) in CH_2Cl_2 (500 μL), was slowly added phenylacetyl chloride (5.3 μL, 40.2 μmol) at 0 °C. After the resulting mixture was stirred at room temperature for 9 h, the resulting mixture was concentrated under vacuum. The crude materials were filtered through basic Al_2O_3 pad and purified by column chromatography on silica gel (20% to 40% EtOAc in hexane) to afford **IIIa** (12.6 mg, 21.6 μmol, 81% 2 steps) as colorless oil.

^1H NMR (500 MHz; $CDCl_3$):δ 7.40-7.17 (m, 15H), 6.15 (s, 1H), 5.51 (s, 2H), 4.38 (d, *J* = 5.8 Hz, 2H), 4.34 (s, 2H),3.65 (s, 2H), 3.40-1.80 (m, 9H); ^{13}C NMR (125 MHz; $CDCl_3$): δ 170.1, 159.8, 136.7, 135.0, 133.1, 129.5, 129.2, 129.1, 128.8, 128.7, 128.3, 128.1, 127.6, 127.5, 127.0, 74.5, 71.8, 64.6, 53.8, 45.0, 41.1; ^{11}B NMR (160 MHz; $CDCl_3$): δ –4.7, -7.5, -10.3, -12.0, -15.0; HRMS (ESI, positive) for $C_{28}H_{34}B_{10}N_4O_3$ (m/z): calculated 619.3301 $(M+Na)^+$, found 619.3329.

Synthesis of 1-(3-methylbutanoyl)oxymethyl-7-benzylaminocarboxyl-9-(1*H*-1,2,3-triazol-4-yl)benzylcarbamoyl-1,7-dicarba-*closo*-dodecaborane (IIIb)

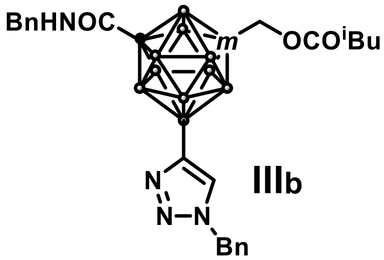

This compound was prepared from **20a** (7.1 mg, 12.3 μmol) and isovaleryl chloride using the procedure described before for **IIIa** in 76% yield as colorless oil.

^1H NMR (500 MHz; $CDCl_3$):δ 7.33-7.26 (m, 9H), 7.18 (d, *J* = 8.2 Hz, 2H), 6.15 (s, 1H), 5.51 (s, 2H), 4.38 (d, *J* = 5.7 Hz, 2H), 4.33 (s, 2H), 3.40-1.80 (m, 9H), 2.23 (d, *J* = 7.2 Hz, 2H), 2.14-2.07 (m, 1H), 0.96 (d, *J* = 6.7 Hz, 6H); ^{13}C NMR (125 MHz; $CDCl_3$): δ 171.6, 159.9, 136.7, 135.0, 129.2, 129.1, 128.7, 128.3, 128.1, 127.6, 127.0, 74.5, 72.1, 64.2, 53.8, 45.0, 43.0, 25.6, 22.5; ^{11}B NMR (160 MHz; $CDCl_3$): δ –4.7, -7.5, -10.2, -11.8, -15.3; HRMS (ESI, positive) for $C_{25}H_{36}B_{10}N_4O_3$ (m/z): calculated 585.3455 $(M+Na)^+$, found 585.3460.

Synthesis of 1-phenylacetoxymethyl-7-benzylaminocarboxyl-9-((1*H*-1,2,3-triazol-4-yl)-3-methyl-butanoyl)-1,7-dicarba-*closo*-dodecaborane (IIIc)

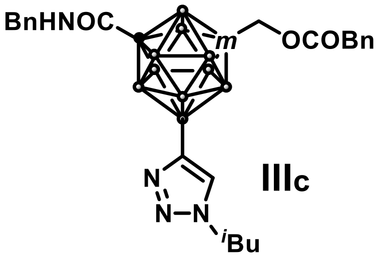

This compound was prepared from **20b** (17.6 mg, 32.3 μmol) using the procedure described before for **IIIa** in 68% yield as colorless oil. ^1H NMR (500 MHz; $CDCl_3$):δ 7.39 (s, 1H), 7.36-7.27 (m, 8H), 7.19 (d, *J* = 6.8 Hz, 2H), 6.13 (s, 1H), 4.39 (d, *J* = 5.7 Hz, 2H), 4.36 (s, 2H), 4.13 (d, *J* = 7.2 Hz, 2H), 3.67 (s, 2H), 3.40-1.80 (m, 9H), 2.23-2.17 (m, 1H), 0.94 (d, *J* = 6.7 Hz, 6H); ^{13}C NMR (125 MHz; $CDCl_3$): δ 170.1, 159.8, 136.7, 133.1, 129.5, 129.1, 128.8, 128.1, 127.6, 127.5, 127.5, 74.5, 71.8, 64.6, 57.2, 45.0, 41.0, 29.8, 20.1; ^{11}B NMR (160 MHz; $CDCl_3$): δ -4.5, -7.1, -10.0, -11.6, -15.3; HRMS (ESI, positive) for $C_{25}H_{36}B_{10}N_4O_3$ (m/z): calculated 585.3455 $(M+Na)^+$, found 585.3470.

Synthesis of 1-(3-methylbutanoyl)oxymethyl-7-benzylaminocarboxyl-9-((1*H*-1,2,3-triazol-4-yl)-3-methyl-butanoyl)-1,7-dicarba-*closo*-dodecaborane (IIId)

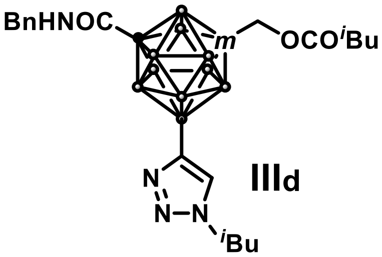

IIId

This compound was prepared from **20b** (20.2 mg, 37.1 μmol) and isovaleryl chloride using the procedure described before for **IIIa** in quantitative yield as colorless oil.

^{1}H NMR (500 MHz; $CDCl_3$):δ 7.41 (s, 1H), 7.35-7.28 (m, 3H), 7.19-7.18 (m, 2H), 6.20 (s, 1H), 4.39 (d, *J* = 5.7 Hz, 2H), 4.35 (s, 2H), 4.13 (d, *J* = 7.2 Hz, 2H), 3.20-1.80 (m, 9H), 2.24 (d, *J* = 7.1 Hz, 2H), 2.22-2.17 (m, 1H), 2.15-2.09 (m, 1H), 0.97 (d, *J* = 6.6 Hz, 6H), 0.93 (d, *J* = 6.7 Hz, 6H); ^{13}C NMR (125 MHz; $CDCl_3$): δ 171.6, 159.9, 136.7, 129.0, 128.1, 127.6, 127.5, 74.5, 72.0, 64.2, 57.2, 45.0, 43.0, 29.8, 25.6, 22.5, 20.1; ^{11}B NMR (160 MHz; $CDCl_3$): δ -4.8, -7.63, -10.4, -12.1, -15.0; HRMS (ESI, positive) for $C_{22}H_{38}B_{10}N_4O_3$ (m/z): calculated 551.3609 $(M+Na)^+$, found 551.3639.

Synthesis of 1-phenylacetoxymethyl-7-isobutylcarbamoyl-9-(1*H*-1,2,3-triazol-4-yl)benzylcarbamoyl-1,7-dicarba-*closo*-dodecaborane (IIIe)

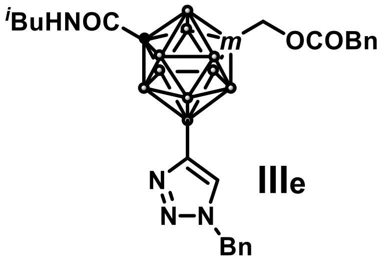

IIIe

This compound was prepared from **20c** (20.8 mg, 38.1 μmol) using the procedure described before for **IIIa** in 61% yield as colorless oil.

^{1}H NMR (500 MHz; $CDCl_3$):δ 7.40-7.23 (m, 10H), 5.89 (s, 1H), 5.52 (s, 2H), 4.34 (s, 2H), 3.65 (s, 2H), 3.02 (d, *J* = 6.0 Hz, 2H), 1.78-1.71 (m, 1H), 0.87 (d, *J* = 6.7 Hz, 6H); ^{13}C NMR (125 MHz; $CDCl_3$): δ 171.1, 159.8, 135.1, 133.1, 129.5, 129.2, 128.8, 128.7, 128.3, 127.5, 127.0, 74.9, 71.8, 64.6, 53.8, 48.2, 41.0, 28.5, 20.0; ^{11}B NMR (160 MHz; $CDCl_3$): δ -5.0, -7.5, -10.4, -12.2, -15.3, -16.7; HRMS (ESI, positive) for $C_{25}H_{36}B_{10}N_4O_3$ (m/z): calculated 585.3455 $(M+Na)^+$, found 585.3456.

Synthesis of 1-(3-methylbutanoyl)oxymethyl-7-isobutylcarbamoyl-9-(1*H*-1,2,3-triazol-4-yl)benzylcarbamoyl-1,7-dicarba-*closo*-dodecaborane (IIIf)

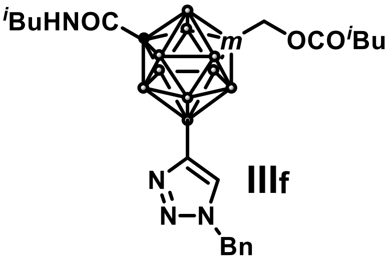

IIIf

This compound was prepared from **20c** (22.0 mg, 40.3 μmol) and isovaleryl chloride using the procedure described before for **IIIa** in 77% yield as colorless oil.

^{1}H NMR (500 MHz; $CDCl_3$):δ 7.40-7.26 (m, 5H), 5.92 (s, 1H), 5.52 (s, 2H), 4.33 (s, 2H), 3.02 (t, *J* = 6.4 Hz, 2H), 2.23 (d, *J* = 7.1 Hz, 2H), 2.14-2.07 (m, 1H), 3.00-1.80 (m, 9H), 1.78-1.72 (m, 1H), 0.96 (d, *J* = 6.7 Hz, 6H), 0.86 (d, *J* = 6.7 Hz, 6H); ^{13}C NMR (125 MHz; $CDCl_3$): δ 171.6, 159.8, 135.0, 129.2, 128.7, 128.3, 127.0, 74.9, 72.0, 64.2, 53.8, 48.2, 43.0, 28.5, 25.6, 22.5, 20.1, 20.0; ^{11}B NMR (160 MHz; $CDCl_3$): δ -4.9, -7.7, -10.4, -12.2, -15.4, -16.8; HRMS (ESI, positive) for $C_{22}H_{38}B_{10}N_4O_3$ (m/z): calculated 551.3609 $(M+Na)^+$, found 551.3611.

Synthesis of 1-phenylacetoxymethyl-7-benzylaminocarboxyl-9-((1*H*-1,2,3-triazol-4-yl)-3-methyl-butanoyl)-1,7-dicarba-*closo*-dodecaborane (IIIg)

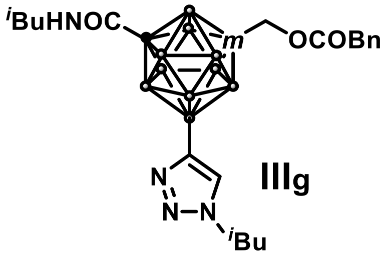

IIIg

This compound was prepared from **20d** (3.9 mg, 7.64 μmol) using the procedure described before for **IIIa** in quantitative yield as colorless oil. ^{1}H NMR (500 MHz; $CDCl_3$):δ 7.40 (s, 1H), 7.35-7.32 (m, 2H), 7.29-7.27 (m, 2H), 5.88 (s, 1H), 4.36 (s, 2H), 4.14 (d, *J* = 7.2 Hz, 2H), 3.66 (s, 2H), 3.03 (t, *J* = 6.4 Hz, 2H), 3.20-1.80 (m, 9H), 2.24-2.19 (m, 1H), 1.78-1.73 (m, 1H), 0.95 (d, *J* = 6.7 Hz, 6H), 0.88 (d, *J* = 6.7 Hz, 6H); ^{13}C NMR (125 MHz; $CDCl_3$): δ 170.1, 159.8, 133.1, 129.6, 128.8, 127.54, 127.48, 74.9, 71.8, 64.6, 57..3, 48.2, 41.1, 29.9, 28.5, 20.1, 20.0; ^{11}B NMR (160 MHz; $CDCl_3$): δ -4.8, -7.1, -10.4, -12.1; HRMS (ESI, positive) for $C_{22}H_{38}B_{10}N_4O_3$ (m/z): calculated 551.3609 $(M+Na)^+$, found 551.3633.

Synthesis of 1-(3-methylbutanoyl)oxymethyl-7-benzylaminocarboxyl-9-((1*H*-1,2,3-triazol-4-yl)-3-methyl-butanoyl)-1,7-dicarba-*closo*-dodecaborane (IIIh)

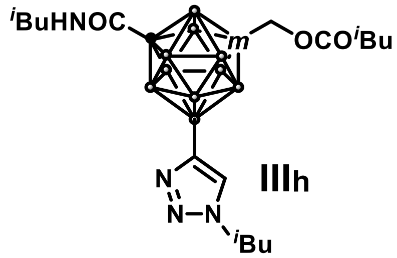

IIIh

This compound was prepared from **20d** (16.5 mg, 32.3 μmol) and isovaleryl chloride using the procedure described before for **IIIa** in quantitative yield as colorless oil.
^{1}H NMR (500 MHz; $CDCl_3$):δ 7.42 (s, 1H), 5.93 (s, 1H), 4.35 (s, 2H), 4.14 (d, *J* = 7.3 Hz, 2H), 3.04 (d, *J* = 7.3 Hz, 2H), 3.00-1.80 (m, 9H), 2.24 (d, *J* = 7.1 Hz, 2H), 2.23-2.17 (m, 1H), 2.15-2.07 (m, 1H), 1.79-1.73 (m, 1H), 0.97 (d, *J* = 6.7 Hz, 6H), 0.95 (d, *J* = 6.7 Hz, 6H), 0.87 (d, *J* = 6.7 Hz, 6H); ^{13}C NMR (125 MHz; $CDCl_3$): δ 171.7, 159.9, 127.5, 74.9, 72.0, 64.2, 57.3, 48.2, 43.0, 29.9, 28.5, 25.6, 22.5, 20.1, 20.0; ^{11}B NMR (160 MHz; $CDCl_3$): δ -4.8, -7.3, -10.2, -12.2, -15.0, -16.7; HRMS (ESI, positive) for $C_{19}H_{40}B_{10}N_4O_3$ (m/z): calculated 517.3752 $(M+Na)^+$, found 517.3733.

➢ Synthesis of scaffold IV-type compounds

Synthesis of 9,10-diiodo-1,7-dicarba-*closo*-dodecaborane (21) and 4,9,10-triiodo-1,7-dicarba-*closo*-dodecaborane (22)

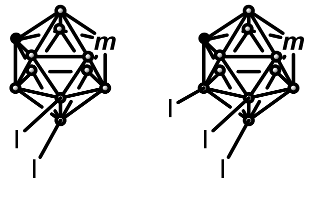

21 22

To a solution of *m*-carborane (72.1 mg, 0.500 mmol) and I_2 (133.1 mg, 0.525 mmol) in acetic acid (1.5 mL) was added a 1:1 mixture of concentrated H_2SO_4 and concentrated HNO_3 (0.375 mL). The resulting mixture was stirred at 100 °C for 40 min with microwave. Then, the colorless orange mixture was cooled to room temperature and poured into ice water. The white precipitate was filtered, washed with water, and the resulting solid was dissolved in ether and recrystallized with hexane to give **21**. The remaining mixture was purified by column chromatography on silica gel (15% EtOAc in hexane) to give white solids **21** (166.7 mg, 0.42 mmol, 84%) and **22** (26.6 mg, 0.051 mmol, 10%).
(21)^{1}H NMR (500 MHz; $CDCl_3$): δ 3.18 (s, 2H).
The NMR spectra of this compound is consistent with previous report.[3]
(22)^{1}H NMR (500 MHz; $CDCl_3$): δ 3.61 (s, 1H), 3.48 (s, 1H), 4.00-2.50 (m, 7H); ^{13}C NMR (125 MHz; $CDCl_3$): 58.9, 54.0; δ ;^{11}B NMR (160 MHz; $CDCl_3$): δ -4.1, -9.9, -11.8, -15.7, -18.0; HRMS (ESI, positive) for $C_2H_9B_{10}I_3$ (m/z): calculated 546.8665 $(M+Na)^+$, found 546.8665.

Synthesis of 9,10-di((trimethylsilyl)ethynyl)-1,7-dicarba-*closo*-dodecaborane (23)

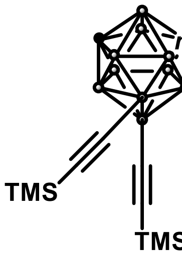

23

To a solution of TMS acetylene (1.46 ml, 10.6 mmol) in THF (6 ml), add ethyl magnesium bromide 3.0 M in THF (3.51 ml, 10.6 mmol) under argon; reflux at 60°C for 2 hours.
To a solution of **21** (837.7 mg, 2.11 mmol) in THF (14 ml), add dichlorobis(triphenylphosphine)-palladium (147.3 mg, 0.21 mmol) under argon and degas. Add the previously adjusted TMS acetylene magnesium bromide and reflux at 60°C overnight. The reaction was quenched with NH_4Cl and the brown mixture was extracted with EtOAc, washed with brine, dried over sodium sulfate, and concentrated under vacuum. The crude material was purified by column chromatography on silica gel (10% EtOAc in Hexane) afforded **23** (666.7 mg, 1.98 mmol, 94%) as a white solid.
^{1}H NMR (500 MHz; $CDCl_3$): δ 2.83(s, 2H), 0.17 (s, 18H).
The NMR spectra of this compound is consistent with previous report.[3]

Synthesis of 1-benzylaminocarboxyl-9,10-di((trimethylsilyl)ethynyl)-1,7-dicarba-*closo*-dodecaborane (24a)

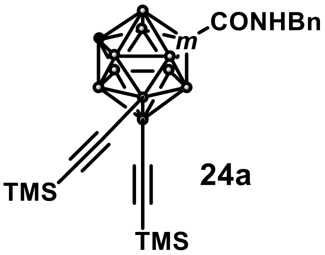

24a

To a solution of **23** (0.952 g, 2.50 mmol) in THF (10 mL), was slowly added *n*-BuLi 1.6 M solution in hexane (1.75 mL, 2.75 mmol) at -78 °C. After the resulting mixture was stirred at -78 °C under argon atmosphere for 2 h, CO_2 balloon was attached. Then, the resulting mixture was stirred at 0 °C for 2 h under CO_2 atmosphere. The reaction was quenched with 1M HCl and the mixture was extracted with Et_2O, washed with brine, dried over sodium sulfate, and concentrated under vacuum. The crude materials were used to the next reaction without further purification.
To a solution of the crude material in THF (4 mL), benzylamine (328 μL, 3.00 mmol), COMU (11.18 g, 2.75 mmol) and DIEA (871 μL, 5.00 mmol) were added. The resulting mixture was stirred at room temperature under argon atmosphere for 25 min until complete conversion of the starting material was observed. The resulting mixture was added aq. $NaHCO_3$ and EtOAc after which the product was partitioned between the aqueous and organic layers. The aqueous layer was extracted with EtOAc and combined organic layers were washed with brine, dried over sodium sulfate, and concentrated under vacuum. The crude material was purified by column chromatography on silica gel (10% EtOAc) to afford **24a** (484.4 mg, 1.03 mmol, 41% 2 steps) as a white solid; m.p. 127-128 °C; ^{1}H NMR (400 MHz; $CDCl_3$): δ 7.35-7.16 (m, 5H), 6.15 (t, *J*=5.3 Hz, 1H), 4.35(d, *J* = 5.7 Hz, 2H), 2.96(s, 1H), 0.18 (s, 18H) ^{13}C NMR (125 MHz; $CDCl_3$): δ 159.6, 136.7, 129.1, 128,1, 127.6, 107.2, 72.0, 51.4, 45.0 0.22 ^{11}B NMR (160 MHz; $CDCl_3$): δ -6.32, -9.63, -13.63, -18.77; HRMS (ESI, positive) for $C_{20}H_{35}B_{10}NOSi_2$ (m/z): calculated 494.3114 $(M+Na)^+$, found 494.3117.

Synthesis of 1-isobutylaminocarboxyl-9,10-di((trimethylsilyl)ethynyl)-1,7-dicarba-*closo*-dodecaborane (24b)

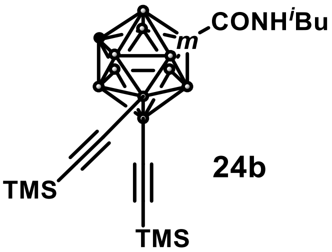

24b

This compound was prepared from **23** (1.01 g, 2.65 mmol) and isobutylamine using the procedure described before for **24a** (572.4 mg, 1.31 mmol, 48% 2 steps) as a white solid; m.p. 166-167 °C; ^{1}H NMR (400 MHz; $CDCl_3$): δ 5.85 (t, *J* = 5.5 Hz, 1H), 3.00 (t, *J* = 6.5 Hz, 2H), 2.93 (s, 1H), 1.78-1.68 (m, *J* = 6.7 Hz, 1H), 0.86 (d, *J* = 6.7 Hz, 6H), 0.16 (s, 18H); ^{13}C NMR (125 MHz; $CDCl_3$): δ 159.6, 107.0, 72.4, 51.4, 48.2, 28.5, 20.0, 0.24; ^{11}B NMR (160 MHz; $CDCl_3$): δ -6.52, -9.73, -13.75, -18.90; HRMS (ESI, positive) for $C_{17}H_{37}B_{10}NOSi_2$ (m/z): calculated 460.3268 $(M+Na)^+$, found 460.3265.

Synthesis of 1-benzylaminocarboxyl-9,10-di((trimethylsilyl)ethynyl)-1,7-dicarba-*closo*-dodecaborane (25)

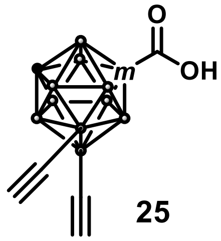

25

To a solution of **23** (460 mg, 1.37 mmol) in THF (11 mL), was slowly added *n*-BuLi 1.6 M solution in hexane (957 µL, 1.50 mmol) at -78 °C. After the resulting mixture was stirred at -78 °C under argon atmosphere for 2 h, CO_2 balloon was attached. Then, the resulting mixture was stirred at 0 °C for 2 h under CO_2 atmosphere. The reaction was quenched with 1M HCl and the mixture was extracted with Et_2O, washed with brine, dried over sodium sulfate, and concentrated under vacuum. The crude materials were used to the next reaction without further purification.

To a solution of the crude material in MeOH (10 mL), potassium carbonate (568 mg, 4.11 mmol) was added. The resulting mixture was stirred at room temperature for 30 min until the full conversion of a starting material was observed. After that, the reaction mixture was concentrated under pressure. Hexane was added after which the product was partitioned between the aqueous and organic layers. The aqueous layer was washed with hexane. Then, to the aqueous layer, were added 6 M HCl and Et_2O after which the product was partitioned between the aqueous and organic layers. The aqueous layer was washed with hexane and combined organic layers were dried over sodium sulfate and concentrated under vacuum to afford **25** (97 mg, 0.411 mmol, 30% 2 steps) as a white solid; m.p. 166-167 °C; ^{1}H NMR (400 MHz; $CDCl_3$): δ 5.85 (t, *J* = 5.5 Hz, 1H), 3.00 (t, *J* = 6.5 Hz, 2H), 2.93 (s, 1H), 1.78-1.68 (m, *J* = 6.7 Hz, 1H), 0.86 (d, *J* = 6.7 Hz, 6H), 0.16 (s, 18H); ^{13}C NMR (125 MHz; $CDCl_3$): δ 159.6, 107.0, 72.4, 51.4, 48.2, 28.5, 20.0, 0.24; ^{11}B NMR (160 MHz; $CDCl_3$): δ -6.52, -9.73, -13.75, -18.90; HRMS (ESI, positive) for $C_{17}H_{37}B_{10}NOSi_2$ (m/z): calculated 460.3268 $(M+Na)^+$, found 460.3265.

Synthesis of 1-benzylaminocarboxyl-9,10-diethynyl-1,7-dicarba-*closo*-dodecaborane (S6a)

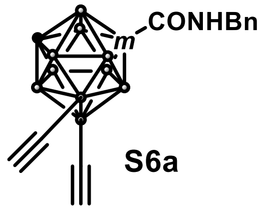

S6a

To a solution of **24a** (484.4 mg, 1.03 mmol) in MeOH (10 mL), potassium carbonate (1.40 g, 10.3 mmol) was added. The resulting mixture was stirred at room temperature for 30 min until the full conversion of a starting material was observed. To the resulting mixture was added DCM after which the product was filtered with celite. The material was purified by short column chromatography on silica gel (DCM 100%) to afford **S6a** (309.1 mg, 0.95 mmol, 92%) as a white solid; m.p. 141-142 °C; ^{1}H NMR (400 MHz; $CDCl_3$): δ 7.28-7.08 (m, 5H), 6.09 (t, *J* = 4.8 Hz, 1H), 4.29(d, *J* = 5.7 Hz, 2H), 2.97(s, 1H), 2.33 (s, 2H); ^{13}C NMR (125 MHz; $CDCl_3$): δ159.2, 136.6, 129.0, 128.128.1, 128.0, 87.7, 72.6, 52.0, 45.0; ^{11}B NMR (160 MHz; $CDCl_3$): δ -5.33, -6.62, -9.88, -12.02, -13.42, -18.39; HRMS (ESI, positive) for $C_{14}H_{19}B_{10}NO$ (m/z): calculated 350.2312 $(M+Na)^+$, found 350.2318.

Synthesis of 1-isobutylaminocarboxyl-9,10-diethynyl-1,7-dicarba-*closo*-dodecaborane (S6b)

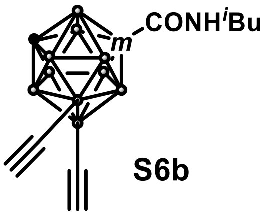

S6b

This compound was prepared from **24b** (572.4 mg, 1.31 mmol) using the procedure described before for **S6a** (383.0 mg, 1.31 mmol, quant.) as a white solid; m.p. 148-149 °C; ^{1}H NMR (400 MHz; $CDCl_3$): δ 5.90 (t, *J* = 4.9 Hz, 1H), 3.06 (s, 1H), 3.02 (t, *J* = 6.4 Hz, 2H), 2.40 (s, 2H), 1.80-1.70 (m, *J* = 6.7 Hz, 1H), 0.86 (d, *J* = 6.7 Hz, 6H); ^{13}C NMR (125 MHz; $CDCl_3$): δ 159.1, 87.7, 77.4, 77.1, 76.8, 72.9, 51.9, 48.1, 28.3, 19.8; ^{11}B NMR (160 MHz; $CDCl_3$): δ -5.28, -6.81, -9.95, -12.03, -13.47, -18.47; HRMS (ESI, positive) for $C_{11}H_{21}B_{10}NO$ (m/z): calculated 316.2464 $(M+Na)^+$, found 316.2465.

Synthesis of 1-benzylaminocarboxyl-9,10-bis((1*H*-1,2,3-triazol-4-yl)benzylcarbamoyl)-1,7-dicarba-*closo*-dodecaborane (IVa)

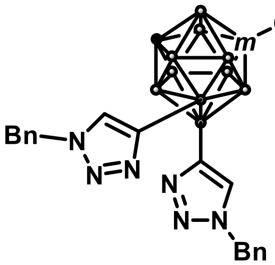

IVa

To a solution of **S6a** (148 mg, 0.455 mmol) in DMF:H_2O (1.6 mL, 4:1, v/v), were added CuI (8.6 mg, 45 µmol), sodium ascorbate (18 mg, 91 µmol) and azide (114 µL, 0.910 mmol). The mixture was stirred at room temperature for 24 h under argon atmosphere. The reaction was quenched with water and the mixture was extracted with EtOAc, washed with brine, dried over sodium sulfate, and concentrated under vacuum. The residue was purified by column chromatography on silica gel (80% EtOAc) to afford **IVa** (220 mg, 0.372 mmol, 82%) as a white solid; m.p. 70-72 °C; ^{1}H NMR (400 MHz; $CDCl_3$): δ 7.33 (s, 2H), 7,28-7.10 (m, 15H), 6.66 (t, *J* = 5.7 Hz, 1H), 5.31 (s, 4H), 4.34 (d, *J* = 5.8 Hz, 2H), 3.18 (s, 1H); ^{13}C NMR (125 MHz; $CDCl_3$): δ 159.7, 142.5, 136.8, 134.9, 128.9, 128.4, 127.8, 127.7, 127.6, 127.4, 72.9, 53.3, 52.2, 44.7; ^{11}B NMR (160 MHz; $CDCl_3$): δ -4.01, -11.27; HRMS (ESI, positive) for $C_{28}H_{33}B_{10}N_7O$ (m/z): calculated 616.3607 $(M+Na)^+$, found 616.3596.

Synthesis of 1-benzylaminocarboxyl-9,10-bis((1*H*-1,2,3-triazol-4-yl)isobutylcarbamoyl)-1,7-dicarba-*closo*-dodecaborane (IVb)

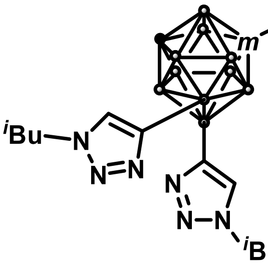

IVb

This compound was prepared from **S6a** (161 mg, 0.494 mmol) using the procedure described before for **IVa** (75 mg, 0.143 mmol, 29%) as a white solid; m.p. 76-77 °C; ^{1}H NMR (500 MHz; $CDCl_3$): δ 7.60 (s, 2H), 7.28-7.22 (m, 5H), 6.88 (t, *J* = 5.0 Hz, 1H), 4.38 (d, *J* = 5.9 Hz, 2H), 4.04 (d, *J* = 7.2 Hz, 4H), 3.28 (s, 1H), 2.17-2.09 (m, 2H), 0.85 (d, *J* = 6.7 Hz, 12H); ^{13}C NMR (125 MHz; $CDCl_3$): δ159.6, 137.1, 128.8, 128.7, 127.7, 127.6, 73.3, 57.4, 52.5, 44.7, 29.5, 19.9; ^{11}B NMR (160 MHz; $CDCl_3$): δ -5.07, -12.35, -17.16; HRMS (ESI, positive) for $C_{22}H_{37}B_{10}N_7O$ (m/z): calculated 548.3914 $(M+Na)^+$, found 548.3916.

Synthesis of 1-isobutylaminocarboxyl-9,10-bis((1*H*-1,2,3-triazol-4-yl)benzylcarbamoyl)-1,7-dicarba-*closo*-dodecaborane (IVc)

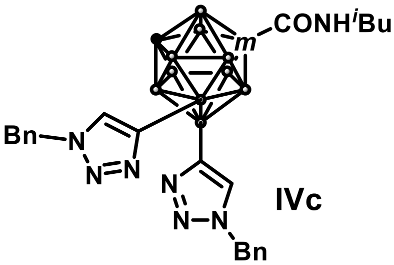

IVc

This compound was prepared from **S6b** (192 mg, 0.660 mmol) using the procedure described before for **IVa** (277 mg, 0.497 mmol, 75%) as a white solid; m.p. 176-177 °C; ^{1}H NMR (500 MHz; $CDCl_3$): δ 7.34 (s, 2H), 7.30-7.13 (m, 10H), 6.24 (t, *J* = 5.7 Hz, 1H), 5.35 (s, 4H), 3.22 (s, 1H), 3.01 (t, *J* = 6.5 Hz, 2H), 1.78-1.70 (m, 1H), 0.83 (d, *J* = 6.7 Hz, 6H); ^{13}C NMR (125 MHz; $CDCl_3$): δ159.6, 128.7, 128.2, 127.7, 127.6, 73.1, 53.2, 52.1, 47.9, 28.1, 19.7; ^{11}B NMR (160 MHz; $CDCl_3$): δ -4.70, -13.10, -17.48; HRMS (ESI, positive) for $C_{25}H_{35}B_{10}N_7O$ (m/z): calculated 582.3761 $(M+Na)^+$, found 582.3759.

Synthesis of 1-isobutylaminocarboxyl-9,10-bis((1*H*-1,2,3-triazol-4-yl)isobutylcarbamoyl)-1,7-dicarba-*closo*-dodecaborane (IVd)

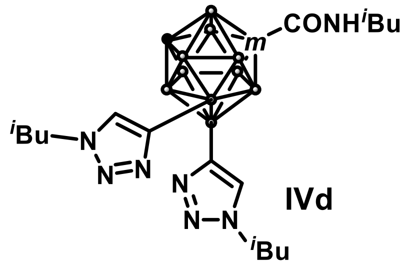

IVd

This compound was prepared from **S6b** (191 mg, 0.654 mmol) using the procedure described before for **IVa** (123 mg, 0.251 mmol, 38%) as a white solid; m.p. 103-105 °C; ^{1}H NMR (500 MHz; $CDCl_3$): δ 7.51 (s, 2H), 6.20 (t, *J* = 5.3 Hz, 1H), 4.10 (d, *J* = 7.5 Hz, 4H), 3.22 (s, 1H), 3.05 (t, *J* = 6.5 Hz, 2H), 2.22-2.13 (m, 2H), 1.85-1.77 (m, 1H), 0.89 (d, *J* = 6.7 Hz, 12H), 0.88 (d, *J* = 6.7 Hz, 6H); ^{13}C NMR (125 MHz; $CDCl_3$): δ159.5, 128.5, 73.81, 57.5, 52.0, 48.2, 29.5, 28.4, 20.0, 19.9; ^{11}B NMR (160 MHz; $CDCl_3$): δ -4.81, -11.24, -17.19; HRMS (ESI, positive) for $C_{19}H_{39}B_{10}N_7O$ (m/z): calculated 514.4067 $(M+Na)^+$, found 514.4064.

➢ Synthesis of scaffold V-type compounds

Synthesis of 2-(3-((4-methoxybenzyl)oxy)prop-1-yn-1-yl)-1,12-dicarba-*closo*-dodecaborane (26)

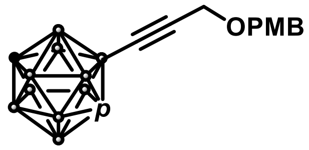

26

To a solution of 1-methoxy-4-((prop-2-yn-1-yloxy)methyl)benzene (1.59 g, 9.00 mmol) in toluene:piperidine (13.5 mL, 2:1, v/v), were added CuI (57.0 mg, 0.300 mmol), $PdCl_2(PPh_3)_2$ (210 mg, 0.300 mmol) and 2-iodo-1,12-dicarba-*closo*-dodecaborane (810 mg, 3.00 mmol) prepared according to the reported literature.[3] The mixt99%ure was stirred at 80 °C under argon atmosphere for 40 min with microwave synthesizer. The reaction was quenched with 1N HCl aq. and Et_2O. The resulting mixture was partitioned between the aqueous and organic layers. The aqueous layer was washed with Et_2O, and the mixture was extracted with Et_2O, washed with brine, dried over sodium sulfate, and concentrated under vacuum. The residue was purified by column chromatography on silica gel (5% to 10% EtOAc in Hexane) to afford **26** as a colorless oil (50 mg, 0.157 mmol, 49%).

^{1}H NMR (500 MHz; $CDCl_3$): δ 7.28 (d, *J* = 8.6 Hz, 2H), 6.88 (d, *J* = 8.6 Hz, 2H), 4.53 (s, 2H), 4.13 (s, 2H), 3.80 (s, 3H), 3.01 (s, 1H), 2.78 (s, 1H), 2.90-1.40 (m, 9H); ^{13}C NMR (125 MHz; $CDCl_3$): δ 159.5, 130.0, 129.6, 93.56, 71.4, 66.3, 63.2, 57.6, 55.4; ^{11}B NMR (160 MHz; $CDCl_3$): δ -13.3, -14.3, -15.1, -16.8; HRMS (ESI, positive) for $C_{13}H_{22}B_{10}O_2$ (m/z): calculated 343.2464 $(M+Na)^+$, found 343.2472.

Synthesis of 12-((*tert*-butyl)dimethylsilyl)hydroxymethyl)-7-(3-((4-methoxybenzyl)oxy)prop-1-yn-1-yl)-1,12-dicarba-*closo*-dodecaboranyl-1-carboxylic acid (27) and 12-((*tert*-butyl)dimethylsilyl)hydroxymethyl)-2-(3-((4-methoxybenzyl)oxy)prop-1-yn-1-yl)-1,12-dicarba-*closo*-dodecaboranyl-1-carboxylic acid (28)

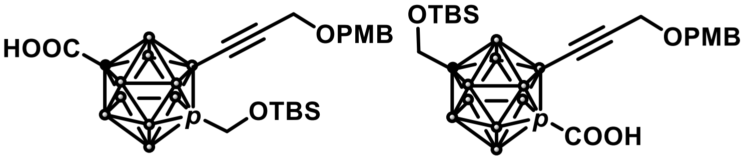

27 **28**

To a solution of **26** (202 mg, 0.634 mmol) in THF (2.5 mL), was slowly added *n*-BuLi 1.6 M solution in hexane (400 µL, 0.634 mmol) at -78 °C. After the resulting mixture was stirred at -78 °C under argon atmosphere for 2 h, paraformaldehyde (23 mg, 0.761 mmol) was added at -78 °C. Then, the resulting mixture was warmed up to room temperature and stirred for 12 h under argon atmosphere. The reaction mixture was quenched with aq. 1N HCl. The resulting mixture was partitioned between the aqueous and organic layers. The aqueous layer was washed with EtOAc, and the mixture was extracted with EtOAc, washed with brine, dried over sodium sulfate and concentrated under vacuum. The residue was purified by column chromatography on silica gel (10% to 20% EtOAc in Hexane) to afford the mixture of regioisomer to use in the next step without further purification.

To a solution of the mixture and 2,6-lutidine (56 µL, 0.482 mmol) in DCM (1.2 mL) was slowly added TBSOTf (89 µL,

0.386 mmol) at 0 °C under argon atmosphere. The resulting mixture was stirred at room temperature until the starting material disappeared as monitored by TLC analysis. Then, the reaction mixture was quenched with a small amount of aq. $NaHCO_3$ and filtered on celite pad. The filtrate was concentrated under vacuum. The crude material was purified by column chromatography on silica gel (10% EtOAc in Hexane) to afford the mixture of regioisomer to use in the next step without further purification.

To a solution of the mixture in THF (1 mL), was slowly added *n*-BuLi 1.6 M solution in hexane (185 μL, 0.288 mmol) at -78 °C. After the resulting mixture was stirred at -78 °C under argon atmosphere for 2 h, CO_2 balloon was attached. Then, the resulting mixture was warmed up to room temperature and stirred for 12 h under CO_2 atmosphere. After that, the reaction mixture was concentrated under pressure. Hexane was added after which the product was partitioned between the aqueous and organic layers. The aqueous layer was washed with hexane. Then, to the aqueous layer, were added 6 M HCl and Et_2O after which the product was partitioned between the aqueous and organic layers. The aqueous layer was washed with hexane and combined organic layers were dried over sodium sulfate and concentrated under vacuum. The crude material was purified by reverse-phase HPLC (40% to 100% MeCN in H_2O) to afford 60.5 mg of faster eluting isomer **27** as colorless oil, 43.6 mg of slower eluting isomer **28** as a white solid, and 10.5 mg of the mixture (total yield 114.6 mg, 0.226 mmol, 37%, 3 steps).

27: ^1H NMR (400 MHz; $CDCl_3$): major isomer δ 7.28 (d, *J* = 8.6 Hz, 2H), 6.88 (d, *J* = 8.6 Hz, 2H), 4.53 (s, 2H), 4.15 (s, 2H), 3.81 (s, 3H), 3.63 (s, 2H), 3.00-1.70 (br, 9H), 0.85 (s, 9H), -0.01 (s, 6H); ^{13}C NMR (100 MHz; $CDCl_3$): δ 166.3, 159.7, 130.0, 129.4, 114.1, 96.3, 85.2, 74.8, 71.3, 65.0, 57.5, 55.5, 25.8, 18.3, -5.4; ^{11}B NMR (128 MHz; $CDCl_3$): δ -13.2; HRMS (ESI, positive) for $C_{26}H_{36}B_{10}N_2O_4$ (m/z): calculated 531.3346 $(M+Na)^+$, found 531.3342.

28: ^1H NMR (400 MHz; $CDCl_3$): δ 7.28 (d, *J* = 8.6 Hz, 2H), 6.88 (d, *J* = 8.6 Hz, 2H), 4.50 (s, 2H), 4.10 (s, 2H), 3.81 (s, 3H), 3.44 (s, 2H), 3.00-1.70 (br, 9H), 0.85 (s, 9H), -0.03 (s, 6H); ^{13}C NMR (100 MHz; $CDCl_3$): δ 164.1, 159.6, 130.2, 129.5, 114.1, 71.0, 66.5, 57.4, 55.5, 25.8, 18.3, -5.5; ^{11}B NMR (128 MHz; $CDCl_3$): δ -13.2; HRMS (ESI, positive) for $C_{26}H_{36}B_{10}N_2O_4$ (m/z): calculated 531.3346 $(M+Na)^+$, found 531.3345.

Synthesis of 1-((*tert*-butyl)dimethylsilyl)hydroxymethyl)-2-(3-((4-methoxybenzyl)oxy)prop-1-yn-1-yl)-12-benzylaminocarboxyl-1,12-dicarba-*closo*-dodecaborane (29a)

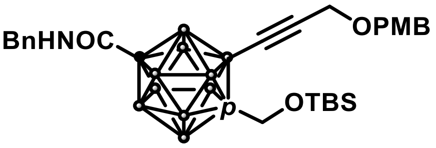

29a

To a solution of **27** (25.2 mg, 49.7 μmol) in THF (1.0 mL), benzyllamine (6.5 μL, 59.7 μmol), EDCI (11 mg, 59.7 μmol), HOBt (11 mg, 74.6 μmol) and DIEA (25 μL, 0.149 mmol) were slowly added. The resulting mixture was stirred at room temperature under argon atmosphere until the full conversion of a starting material was observed. To the resulting mixture were added aq. $NaHCO_3$ and EtOAc after which the product was partitioned between the aqueous and organic layers. The aqueous layer was washed with EtOAc and combined organic layers were washed with brine, dried over sodium sulfate and concentrated under vacuum. The crude material was purified by column chromatography on silica gel (20% EtOAc in Hexane) to afford **29a** (20.2 mg, 33.9 μmol, 68%) as a colorless oil. ^1H NMR (400 MHz; $CDCl_3$): δ 7.33-7.27 (m, 5H), 7.15-7.13 (m, 2H), 6.88 (d, *J* = 8.7 Hz, 2H), 5.88 (s, 1H), 4.53 (s, 2H), 4.30 (d, *J* = 5.7 Hz, 2H), 4.15 (s, 2H), 3.81 (s, 3H), 3.65 (s, 2H), 3.10-1.70 (br, 9H), 0.85 (s, 9H), -0.01 (s, 6H); ^{13}C NMR (100 MHz; $CDCl_3$): δ 160.8, 159.6, 137.1, 129.9, 129.6, 129.0, 128.0, 127.5, 114.1, 71.4, 64.9, 57.6, 55.4, 44.8, 25.8, 18.3, -5.4; ^{11}B NMR (128 MHz; $CDCl_3$): δ -13.1; HRMS (ESI, positive) for $C_{28}H_{45}B_{10}NO_4Si$ (m/z): calculated 620.3982 $(M+Na)^+$, found 620.3990.

Synthesis of 1-((*tert*-butyl)dimethylsilyl)hydroxymethyl)-2-(3-((4-methoxybenzyl)oxy)prop-1-yn-1-yl)-12-isobutylcarbamoyl-1,12-dicarba-*closo*-dodecaborane (29b)

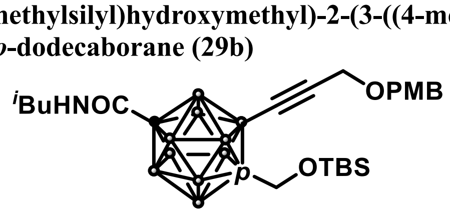

29b

To a solution of **27** (35.3 mg, 69.7 μmol) in THF (1.0 mL), isobutylamine (8.4 μL, 83.6 μmol), EDCI (16 mg, 83.6 μmol), HOBt (16 mg, 0.105 mmol) and DIEA (36 μL, 0.209 mmol) were slowly added. The resulting mixture was stirred at room temperature under argon atmosphere until the full conversion of a starting material was observed. To the resulting mixture were added aq. $NaHCO_3$ and EtOAc after which the product was partitioned between the aqueous and organic layers. The aqueous layer was washed with EtOAc and combined organic layers were washed with brine, dried over sodium sulfate and concentrated under vacuum. The crude material was purified by column chromatography on silica gel (20% EtOAc in Hexane) to afford **29b** (18.3 mg, 32.6 μmol, 47%) as a colorless oil. ^1H NMR (400 MHz; $CDCl_3$): δ

7.28 (d, *J* = 8.6 Hz, 2H), 6.88 (d, *J* = 8.6 Hz, 2H), 5.64 (s, 1H), 4.53 (s, 2H), 4.15 (d, *J* = 5.7 Hz, 2H), 3.81 (s, 3H), 3.65 (s, 2H), 2.94 (t, *J* = 6.4 Hz, 2H), 3.10-1.70 (br, 9H), 1.75-1.65 (m, 1H), 0.85 (s, 9H), 0.84 (d, *J* = 6.7 Hz, 6H), -0.01 (s, 6H); ^{13}C NMR (100 MHz; $CDCl_3$): δ 160.7, 159.6, 129.9, 129.6, 114.0, 83.9, 79.4, 71.3, 64.9, 57.6, 55.4, 48.0, 29.8, 28.4, 25.8, 19.9, 18.3, -5.4; ^{11}B NMR (128 MHz; $CDCl_3$): δ -13.3; HRMS (ESI, positive) for $C_{25}H_{47}B_{10}NO_4Si$ (m/z): calculated 586.4136 $(M+Na)^+$, found 586.4136.

Synthesis of 12-((*tert*-butyl)dimethylsilyl)hydroxymethyl)-2-(3-((4-methoxybenzyl)oxy)prop-1-yn-1-yl)-1-benzylaminocarboxyl-1,12-dicarba-*closo*-dodecaborane (33a)

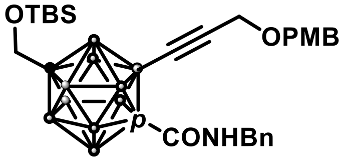

33a

This compound was prepared from **28** (18.4 mg, 36.3 µmol) using the procedure described before for **29a** in 73% yield as colorless oil. ^{1}H NMR (500 MHz; $CDCl_3$): δ 7.28-7.26 (m, 5H), 7.19-7.17 (m, 2H), 6.89 (d, *J* = 8.7 Hz, 2H), 6.42 (s, 1H), 4.46 (s, 2H), 4.34 (d, *J* = 5.6 Hz, 2H), 3.94 (s, 2H), 3.82 (s, 3H), 3.48 (s, 2H), 3.00-1.70 (br, 9H), 0.87 (s, 9H), -0.01 (s, 6H); ^{13}C NMR (125 MHz; $CDCl_3$): δ 160.3, 159.6, 137.1, 130.0, 129.4, 128.9, 127.8, 127.7, 114.0, 83.8, 79.7, 71.4, 66.3, 57.4, 55.4, 44.8, 29.8, 25.8, -5.5; ^{11}B NMR (160 MHz; $CDCl_3$): δ -13.6; HRMS (ESI, positive) for $C_{28}H_{45}B_{10}NO_4Si$ (m/z): calculated 620.3982 $(M+Na)^+$, found 620.3987.

Synthesis of 12-((*tert*-butyl)dimethylsilyl)hydroxymethyl)-2-(3-((4-methoxybenzyl)oxy)prop-1-yn-1-yl)-1-isobutylcarbamoyl-1,12-dicarba-*closo*-dodecaborane (33b)

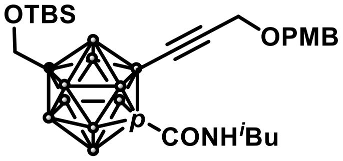

33b

This compound was prepared from **28** (23.1 mg, 45.6 µmol) using the procedure described before for **29b** in 83% yield as colorless oil. ^{1}H NMR (500 MHz; $CDCl_3$): δ 7.28 (d, *J* = 8.6 Hz, 2H), 6.88 (d, *J* = 8.6 Hz, 2H), 6.10 (s, 1H), 4.52 (s, 2H), 4.12 (s, 2H), 3.81 (s, 3H), 3.46 (s, 2H), 2.96 (t, *J* = 6.4 Hz, 2H), 3.00-1.80 (br, 9H), 1.72-1.64 (m, 1H), 0.85 (s, 9H), 0.82 (d, *J* = 6.7 Hz, 6H), -0.03 (s, 6H); ^{13}C NMR (125 MHz; $CDCl_3$): δ 160.2, 159.6, 130.0, 129.4, 114.0, 96.9, 83.6, 80.2, 71.4, 66.3, 57.5, 55.4, 48.1, 28.4, 25.8, 20.0, 18.3, -5.5; ^{11}B NMR (160 MHz; $CDCl_3$): δ -13.6; HRMS (ESI, positive) for $C_{25}H_{47}B_{10}NO_4Si$ (m/z): calculated 586.4136 $(M+Na)^+$, found 586.4136.

Synthesis of 1-((*tert*-butyl)dimethylsilyl)hydroxymethyl)-2-(3-hydroxyprop-1-yn-1-yl)-12-benzylaminocarboxyl-1,12-dicarba-*closo*-dodecaborane (30a)

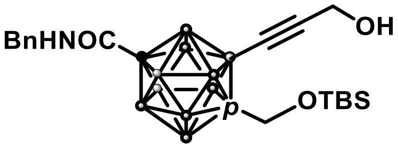

30a

To a solution of **29a** (20 mg, 33.6 µmol) and NaH_2PO_4 (8.1 mg, 67.1 µmol) in DCM/H_2O (500 µL, 10:1, v/v) was added DDQ (15 mg, 67.1 mmol) at 0 °C under argon atmosphere. After the resulting mixture was stirred at room temperature until the starting material disappeared as monitored by TLC analysis. Then, the reaction mixture was concentrated under pressure. Then, the reaction mixture was quenched with a small amount of aq. $NaHCO_3$ and filtered on celite pad. The filtrate was concentrated under vacuum. The crude material was purified by column chromatography on silica gel (20% to 40% EtOAc in Hexane) to afford **30a** (14 mg, 29.4 mmol, 88%) as white solid. m.p. 113-114 °C; ^{1}H NMR (500 MHz; $CDCl_3$): δ 7.34-7.28 (m, 3H), 7.13 (d, *J* = 7.0 Hz, 2H), 5.88 (s, 1H), 4.30 (d, *J* = 5.8 Hz, 2H), 4.28 (s, 2H), 3.63 (s, 2H), 3.00-1.80 (br, 9H), 0.86 (s, 9H), -0.00 (s, 6H); ^{13}C NMR (125 MHz; $CDCl_3$): δ 160.7, 137.0, 129.0, 128.0, 127.5, 84.1, 78.9, 64.8, 51.8, 44.8, 25.8, 18.3, -5.4; ^{11}B NMR (160 MHz; $CDCl_3$): δ -13.6; HRMS (ESI, positive) for $C_{20}H_{37}B_{10}NO_3Si$ (m/z): calculated 500.3398 $(M+Na)^+$, found 500.3399.

Synthesis of 1-((*tert*-butyl)dimethylsilyl)hydroxymethyl)-2-(3-hydroxyprop-1-yn-1-yl)-12-isobutylcarbamoyl-1,12-dicarba-*closo*-dodecaborane (30b)

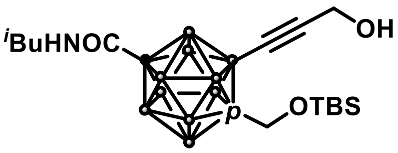

30b

This compound was prepared from **29b** (18.3 mg, 32.6 μmol) using the procedure described before for **30a** in 89% yield as white solid. m.p. 122-123 °C; ^{1}H NMR (500 MHz; $CDCl_3$): δ 5.62 (s, 1H), 4.29 (s, 2H), 3.63 (s, 2H), 2.94 (t, *J* = 6.4 Hz, 2H), 2.90-1.70 (br, 9H), 1.72-1.66 (m, 1H), 0.86 (s, 9H), 0.83 (d, *J* = 6.7 Hz, 6H), 0.00 (s, 6H); ^{13}C NMR (125 MHz; $CDCl_3$): δ 160.7, 83.8, 79.3, 64.8, 51.8, 48.0, 28.4, 25.8, 19.9, 18.3, -5.4; ^{11}B NMR (160 MHz; $CDCl_3$): δ -13.1, -13.6, -14.5; HRMS (ESI, positive) for $C_{17}H_{39}B_{10}NO_3Si$ (m/z): calculated 466.3552 $(M+Na)^+$, found 466.3552.

Synthesis of 12-((*tert*-butyl)dimethylsilyl)hydroxymethyl)-2-(3-hydroxyprop-1-yn-1-yl)-1-benzylaminocarboxyl-1,12-dicarba-*closo*-dodecaborane (34a)

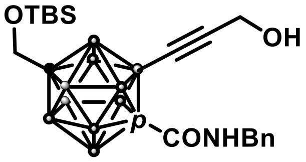

34a

This compound was prepared from **33a** (15.0 mg, 25.2 μmol) using the procedure described before for **30a** in 73% yield as white solid. m.p. 141-142 °C; ^{1}H NMR (500 MHz; $CDCl_3$): δ 7.35-7.28 (m, 3H), 7.24-7.22 (m, 2H), 6.42 (s, 1H), 4.35 (d, *J* = 5.6 Hz, 2H), 4.00 (s, 2H), 3.45 (s, 2H), 3.00-1.70 (br, 9H), 0.84 (s, 9H), -0.03 (s, 6H); ^{13}C NMR (125 MHz; $CDCl_3$): δ 160.3, 137.3, 128.9, 128.00, 127.97, 83.9, 79.6, 66.3, 51.5, 44.9, 25.8, 18.3, -5.5; ^{11}B NMR (160 MHz; $CDCl_3$): δ -13.5; HRMS (ESI, positive) for $C_{20}H_{37}B_{10}NO_3Si$ (m/z): calculated 500.3398 $(M+Na)^+$, found 500.3392.

Synthesis of 12-((*tert*-butyl)dimethylsilyl)hydroxymethyl)-2-(3-hydroxyprop-1-yn-1-yl)-1-isobutylcarbamoyl-1,12-dicarba-*closo*-dodecaborane (34b)

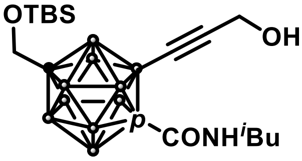

34b

This compound was prepared from **33b** (20.0 mg, 35.6 μmol) using the procedure described before for **30a** in 92% yield as white solid. m.p. 111-112 °C; ^{1}H NMR (500 MHz; $CDCl_3$): δ 6.07 (s, 1H), 4.26 (s, 2H), 3.45 (s, 2H), 2.99 (t, *J* = 6.4 Hz, 2H), 3.00-1.80 (br, 9H), 1.76-1.70 (m, 1H), 0.87 (d, *J* = 6.7 Hz, 6H), 0.84 (s, 9H), -0.03 (s, 6H); ^{13}C NMR (125 MHz; $CDCl_3$): δ 160.3, 83.6, 66.3, 51.7, 48.1, 28.5, 25.8, 20.0, 18.3, -5.5; ^{11}B NMR (160 MHz; $CDCl_3$): δ -13.5; HRMS (ESI, positive) for $C_{17}H_{39}B_{10}NO_3Si$ (m/z): calculated 466.3552 $(M+Na)^+$, found 466.3550.

Synthesis of 1-((*tert*-butyl)dimethylsilyl)hydroxymethyl)-2-(3-oxoprop-1-yn-1-yl)-12-benzylaminocarboxyl-1,12-dicarba-*closo*-dodecaborane (31a)

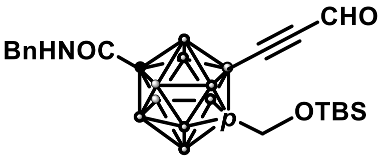

31a

To a solution of **30a** (12.0 mg, 25.2 μmol) in DCM (1 mL) was added Dess-Martin periodinane (21.0 mg, 50.5 μmol) at 0 °C under argon atmosphere. After the resulting mixture was stirred at room temperature until the starting material disappeared as monitored by TLC analysis. Then, the reaction mixture was concentrated under pressure. To the resulting mixture were added $NaHCO_3$ aqueous and Et_2O after which the product was partitioned between the aqueous and organic layers. The aqueous layer was washed with Et_2O and combined organic layers were dried over sodium sulfate and concentrated under vacuum. The crude material was purified by column chromatography on silica gel (20% EtOAc in

Hexane) to afford **31a** (9.6 mg, 20.3 μmol, 62%) as colorless oil. ^{1}H NMR (500 MHz; $CDCl_3$): δ 9.19 (s, 1H), 7.34-7.28 (m, 3H), 7.14 (d, *J* = 7.2 Hz, 2H), 5.88 (s, 1H), 4.30 (d, *J* = 5.6 Hz, 2H), 3.62 (s, 2H), 3.10-1.80 (br, 9H), 0.86 (s, 9H), -0.00 (s, 6H); ^{13}C NMR (125 MHz; $CDCl_3$): δ 176.4, 160.3, 136.9, 129.0, 128.0, 127.5, 84.4, 79.5, 64.8, 44.9, 25.7, 18.3, -5.5; ^{11}B NMR (160 MHz; $CDCl_3$): δ -13.7, -14.1; HRMS (ESI, positive) for $C_{20}H_{35}B_{10}NO_3Si$ (m/z): calculated 498.3241 $(M+Na)^+$, found 498.3249.

Synthesis of 1-((*tert*-butyl)dimethylsilyl)hydroxymethyl)-2-(3-oxoprop-1-yn-1-yl)-12-isobutylcarbamoyl-1,12-dicarba-*closo*-dodecaborane (31b)

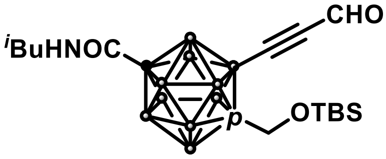

31b

This compound was prepared from **30b** (10.0 mg, 22.6 μmol) using the procedure described before for **31a** in quantitative yield as colorless oil. ^{1}H NMR (500 MHz; $CDCl_3$): δ 9.20 (s, 1H), 5.62 (s, 1H), 3.61 (s, 2H), 2.95 (t, *J* = 6.4 Hz, 2H), 3.00-1.80 (br, 9H), 1.73-1.68 (m, 1H), 0.86 (s, 9H), 0.84 (d, *J* = 6.7 Hz, 6H), 0.00 (s, 6H); ^{13}C NMR (125 MHz; $CDCl_3$): δ 176.4, 160.2, 84.2, 79.9, 64.8, 48.0, 28.4, 25.7, 19.9, 18.3, -5.5; ^{11}B NMR (160 MHz; $CDCl_3$): δ -13.6, -14.2; HRMS (ESI, positive) for $C_{17}H_{39}B_{10}NO_3Si$ (m/z): calculated 466.3552 $(M+Na)^+$, found 466.3552.

Synthesis of 12-((*tert*-butyl)dimethylsilyl)hydroxymethyl)-2-(3-oxoprop-1-yn-1-yl)-1-benzylaminocarboxyl-1,12-dicarba-*closo*-dodecaborane (35a)

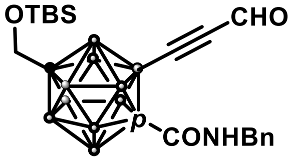

35a

This compound was prepared from **34a** (8.0 mg, 16.8 μmol) using the procedure described before for **31a** in 29% yield as colorless oil. ^{1}H NMR (500 MHz; $CDCl_3$): δ 8.95 (s, 1H), 7.32-7.27 (m, 3H), 7.21-7.19 (m, 2H), 6.03 (s, 1H), 4.35 (d, *J* = 5.7 Hz, 2H), 3.46 (s, 2H), 3.00-1.80 (br, 9H), 0.85 (s, 9H), -0.03 (s, 6H); ^{13}C NMR (125 MHz; $CDCl_3$): δ 176.4, 159.4, 136.9, 129.0, 128.1, 127.8, 84.5, 80.0, 66.2, 45.0, 25.8, 18.3, -5.5; ^{11}B NMR (160 MHz; $CDCl_3$): δ -13.4; HRMS (ESI, positive) for $C_{20}H_{35}B_{10}NO_3Si$ (m/z): calculated 498.3241 $(M+Na)^+$, found 498.3242.

Synthesis of 12-((*tert*-butyl)dimethylsilyl)hydroxymethyl)-2-(3-oxoprop-1-yn-1-yl)-1-isobutylcarbamoyl-1,12-dicarba-*closo*-dodecaborane (35b)

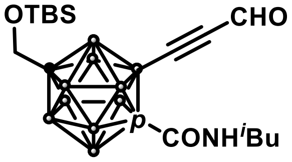

35b

This compound was prepared from **34b** (12.0 mg, 27.2 μmol) using the procedure described before for **31a** in 98% yield as colorless oil. ^{1}H NMR (500 MHz; $CDCl_3$): δ 9.15 (s, 1H), 5.78 (s, 1H), 3.46 (s, 2H), 3.00 (t, *J* = 6.4 Hz, 2H), 3.00-1.80 (br, 9H), 1.76-1.70 (m, 1H), 0.86 (d, *J* = 6.8 Hz, 6H), 0.85 (s, 9H), -0.03 (s, 6H); ^{13}C NMR (125 MHz; $CDCl_3$): δ 176.4, 159.4, 84.3, 80.5, 66.2, 48.2, 28.5, 25.8, 20.0, 18.3, -5.5; ^{11}B NMR (160 MHz; $CDCl_3$): δ -13.4; HRMS (ESI, positive) for $C_{17}H_{37}B_{10}NO_3Si$ (m/z): calculated 464.3395 $(M+Na)^+$, found 466.3401.

Synthesis of 1-(hydroxymethyl)-2-(3-(benzylamino)-3-oxoprop-1-yn-1-yl)-12-benzylaminocarboxyl-1,12-dicarba-*closo*-dodecaborane (32a)

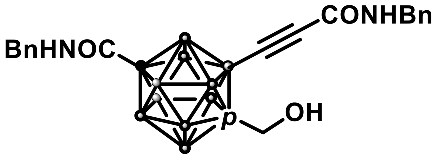

32a

To a solution of **31a** (63.3 mg, 0.134 mmol) and 2-methyl-2-butene (283 μL, 2.68 mmol) in acetone (1 mL) was slowly added a freshly prepared solution of NaH_2PO_4 (250 mg, 1.60 mmol) and $NaClO_2$ (121 mg, 1.33 mmol) in water (1 mL) at 0 °C. After the resulting mixture was stirred at room temperature until the starting material disappeared as monitored by TLC analysis. Then, the reaction mixture was concentrated under pressure. Then, the reaction mixture was quenched with brine, which was partitioned between the aqueous and organic layers. The aqueous layer was washed with DCM and combined organic layers were dried over sodium sulfate and concentrated under vacuum to use in the next step without further purification.

To a solution of the crude material in THF (1 mL), benzylamine (18 μL, 0.161 mmol), EDCI (30.9 mg, 0.161 mmol), HOBt (30.8 mg, 0.201 mmol) and DIEA (68 μL, 0.402 mmol) were slowly added. The resulting mixture was stirred at room temperature under argon atmosphere until the full conversion of a starting material was observed. To the resulting mixture were added aq. $NaHCO_3$ and EtOAc after which the product was partitioned between the aqueous and organic layers. The aqueous layer was washed with EtOAc and combined organic layers were dried over sodium sulfate and concentrated under vacuum to use in the next step without further purification.

To a solution of the crude material in CH_2Cl_2 (500 μL), was added 4N HCl in Dioxane (500 μL). The resulting mixture was stirred at room temperature until the full conversion of a starting material was observed. After the reaction mixture was concentrated under pressure, the crude material was purified by column chromatography on silica gel (20% EtOAc in Hexane) to afford **32a** (50.6 mg, 0.109 mmol, 81%) as white solid. m.p. 64-66 °C; ^{1}H NMR (500 MHz; $CDCl_3$): δ 7.38-7.29 (m, 8H), 7.13 (d, *J* = 7.0 Hz, 2H), 6.26 (bs, 1H), 5.84 (bs, 1H), 4.47 (d, *J* = 5.8 Hz, 2H), 4.29 (d, *J* = 5.6 Hz, 2H), 3.66 (s, 2H), 3.00-1.50 (br, 9H); ^{13}C NMR (125 MHz; $CDCl_3$): δ 160.2, 152.5, 137.0, 136.9, 129.1, 129.1, 129.0, 128.2, 128.1, 128.06, 127.5, 84.6, 79.4, 64.7, 44.9, 44.2; ^{11}B NMR (160 MHz; $CDCl_3$): δ -14.1; HRMS (ESI, positive) for $C_{21}H_{28}B_{10}N_2O_3$ (m/z): calculated 489.2954 $(M+Na)^+$, found 489.2951.

Synthesis of 1-(hydroxymethyl)-2-(3-(isobutylamino)-3-oxoprop-1-yn-1-yl)-12-benzylaminocarboxyl-1,12-dicarba-*closo*-dodecaborane (32b)

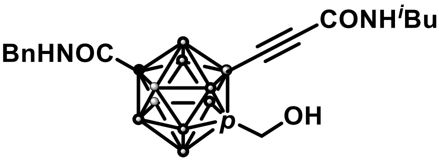

32b

This compound was prepared from **31a** (63.3 mg, 0.134 mmol) and isobutylamine instead of benzylamine using the procedure described before for **32a** in 83% yield as amorphous. ^{1}H NMR (500 MHz; $CDCl_3$): δ 7.35-7.29 (m, 3H), 7.13 (d, *J* = 6.8 Hz, 2H), 6.03 (s, 2H), 5.86 (s, 1H), 4.30 (d, *J* = 5.6 Hz, 2H), 3.13 (t, *J* = 6.5 Hz, 2H), 3.00-1.80 (br, 9H), 1.84-1.79 (m, 1H), 0.94 (d, *J* = 6.7 Hz, 6H); ^{13}C NMR (125 MHz; $CDCl_3$): δ 160.3, 152.7, 136.9, 129.1, 128.1, 127.5, 84.6, 79.4, 64.8, 47.4, 44.9, 28.5, 20.2; ^{11}B NMR (160 MHz; $CDCl_3$): δ -14.1; HRMS (ESI, positive) for $C_{18}H_{30}B_{10}N_2O_3$ (m/z): calculated 455.3107 $(M+Na)^+$, found 455.3107.

Synthesis of 1-(hydroxymethyl)-2-(3-(benzylamino)-3-oxoprop-1-yn-1-yl)-12-isobutylcarbamoyl-1,12-dicarba-*closo*-dodecaborane (32c)

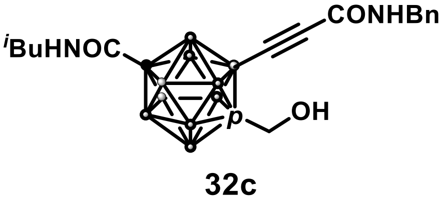

32c

This compound was prepared from **31b** (54.5 mg, 0.124 mmol) using the procedure described before for **32a** in 85% yield as white solid. m.p. 135-136 °C; ^{1}H NMR (500 MHz; $CDCl_3$): δ 7.37-7.29 (m, 5H), 6.42 (s, 1H), 5.61 (s, 1H), 4.47 (d, *J* = 5.8 Hz, 2H), 3.64 (s, 2H), 3.00-1.80 (br, 9H), 2.93 (t, *J* = 6.4, 2H), 1.72-1.66 (m, 1H), 0.83 (d, *J* = 6.7 Hz, 6H); ^{13}C NMR (125 MHz; $CDCl_3$): δ 160.2, 152.7, 137.0, 129.0, 128.2, 128.1, 79.8, 64.6, 48.0, 44.1, 29.8, 28.4, 19.9; ^{11}B

NMR (160 MHz; $CDCl_3$): δ -13.6; HRMS (ESI, positive) for $C_{18}H_{30}B_{10}N_2O_3$ (m/z): calculated 455.3107 $(M+Na)^+$, found 455.3092.

Synthesis of 1-(hydroxymethyl)-2-(3-(isobutylamino)-3-oxoprop-1-yn-1-yl)-12-isobutylcarbamoyl-1,12-dicarba-*closo*-dodecaborane (32d)

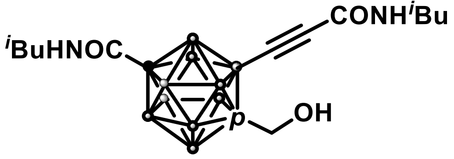

32d

This compound was prepared from **31b** (54.5 mg, 0.124 mmol) and isobutylamine instead of benzylamine using the procedure described before for **32a** in 82% yield as white solid. m.p. 128-129 °C; ^{1}H NMR (500 MHz; $CDCl_3$): δ 6.16 (s, 1H), 5.62 (s, 1H), 3.67 (s, 2H), 3.13 (t, J = 6.5, 2H), 2.94 (t, J = 6.4, 2H), 3.00-1.80 (br, 9H), 1.84-1.79 (m, 1H), 1.73-1.67 (m, 1H), 0.94 (d, J = 6.7 Hz, 6H), 0.84 (d, J = 6.7 Hz, 6H); ^{13}C NMR (125 MHz; $CDCl_3$): δ 160.3, 152.9, 84.5, 79.8, 64.6, 48.1, 47.4, 28.5, 28.4, 20.2, 19.9; ^{11}B NMR (160 MHz; $CDCl_3$): δ -14.0; HRMS (ESI, positive) for $C_{15}H_{32}B_{10}N_2O_3$ (m/z): calculated 421.3260 $(M+Na)^+$, found 421.3261.

Synthesis of 12-(hydroxymethyl)-2-(3-(benzylamino)-3-oxoprop-1-yn-1-yl)-1-benzylaminocarboxyl-1,12-dicarba-*closo*-dodecaborane (36a)

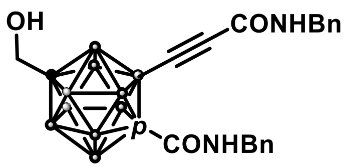

36a

This compound was prepared from **35a** (50.8 mg, 0.107 mmol) using the procedure described before for **32a** in 55% yield as amorphous. ^{1}H NMR (500 MHz; $CDCl_3$): δ 7.35-7.16 (m, 10H), 6.18 (s, 1H), 6.04 (s, 1H), 4.38 (d, J = 5.9 Hz, 2H), 4.32 (d, J = 5.8 Hz, 2H), 3.49 (s, 2H), 3.00-1.60 (br, 9H); ^{13}C NMR (125 MHz; $CDCl_3$): δ 159.8, 152.6, 137.0, 129.0 127.9, 127.5, 127.49, 84.3, 80.1, 65.9, 47.3, 44.9, 28.5, 20.2; ^{11}B NMR (160 MHz; $CDCl_3$): δ -13.8; HRMS (ESI, positive) for $C_{18}H_{30}B_{10}N_2O_3$ (m/z): calculated 489.2954 $(M+Na)^+$, found 489.2947.

Synthesis of 12-(hydroxymethyl)-2-(3-(isobutylamino)-3-oxoprop-1-yn-1-yl)-1-benzylaminocarboxyl-1,12-dicarba-*closo*-dodecaborane (36b)

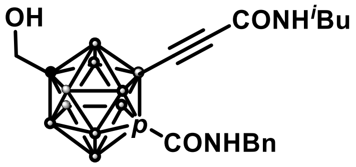

36b

This compound was prepared from **35a** (50.8 mg, 0.107 mmol) and isobutylamine instead of benzylamine using the procedure described before for **32a** in 63% yield as amorphous.

^{1}H NMR (500 MHz; $CDCl_3$): δ 7.30-7.25 (m, 3H), 7.19 (d, J = 7.1 Hz, 2H), 6.24 (d, J = 5.4 Hz, 2H), 5.88 (d, J = 5.2 Hz, 2H), 4.35 (d, J = 5.4 Hz, 2H), 3.50 (s, 2H), 3.03 (t, J = 6.6 Hz, 2H), 3.00-2.00 (br, 9H), 1.76-1.71 (m, 1H), 0.89 (d, J = 6.7 Hz, 6H); ^{13}C NMR (125 MHz; $CDCl_3$): δ 159.8, 152.6, 137.0, 129.0, 127.9, 127.5, 127.49, 84.3, 80.1, 65.9, 47.3, 44.9, 28.5, 20.2; ^{11}B NMR (160 MHz; $CDCl_3$): δ -13.8; HRMS (ESI, positive) for $C_{18}H_{30}B_{10}N_2O_3$ (m/z): calculated 455.3107 $(M+Na)^+$, found 455.3106.

Synthesis of 12-(hydroxymethyl)-2-(3-(benzylamino)-3-oxoprop-1-yn-1-yl)-1-isobutylcarbamoyl-1,12-dicarba-*closo*-dodecaborane (36c)

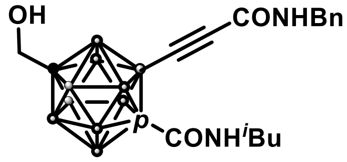

36c

This compound was prepared from **35b** (43.6 mg, 0.094 mmol) using the procedure described before for **32a** in 68% yield as white solid. m.p. 134-135 °C; ^{1}H NMR (500 MHz; $CDCl_3$): δ 7.35-7.27 (m, 5H), 6.23 (s, 1H), 5.84 (s, 1H), 4.45 (d, *J* = 5.9 Hz, 2H), 3.51 (d, *J* = 7.3 Hz, 2H), 2.97 (t, *J* = 4.3 Hz, 2H), 3.00-1.90 (br, 9H), 1.73-1.67 (m, 1H), 0.82 (d, *J* = 6.7 Hz, 6H); ^{13}C NMR (125 MHz; $CDCl_3$): δ 159.5, 152.2, 137.1, 129.0, 128.2, 128.1, 83.8, 66.0, 48.3, 44.1, 28.5, 20.0; ^{11}B NMR (160 MHz; $CDCl_3$): δ -13.4, -14.1; HRMS (ESI, positive) for $C_{18}H_{30}B_{10}N_2O_3$ (m/z): calculated 455.3107 $(M+Na)^+$, found 455.3099.

Synthesis of 12-(hydroxymethyl)-2-(3-(isobutylamino)-3-oxoprop-1-yn-1-yl)-1-isobutylcarbamoyl-1,12-dicarba-*closo*-dodecaborane (36d)

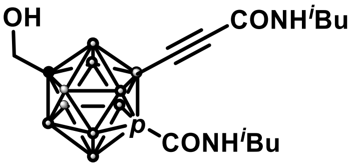

36d

This compound was prepared from **35b** (43.6 mg, 0.094 mmol) and isobutylamine instead of benzylamine using the procedure described before for **32a** in 78% yield as white solid. m.p. 157-158 °C; ^{1}H NMR (500 MHz; $CDCl_3$): δ 5.92 (s, 1H), 5.87 (s, 1H), 3.52 (s, 2H), 3.11 (t, *J* = 6.5 Hz, 2H), 3.00 (t, *J* = 6.4 Hz, 2H), 3.00-1.90 (br, 9H), 1.82-1.71 (m, 2H), 0.93 (d, *J* = 6.7 Hz, 6H), 0.86 (d, *J* = 6.7 Hz, 6H); ^{13}C NMR (125 MHz; $CDCl_3$): δ 159.5, 152.2, 137.1, 129.0, 128.2, 128.1, 83.8, 66.0, 48.3, 44.1, 28.5, 20.0; ^{11}B NMR (160 MHz; $CDCl_3$): δ -13.4, -14.1; HRMS (ESI, positive) for $C_{15}H_{32}B_{10}N_2O_3$ (m/z): calculated 421.3260 $(M+Na)^+$, found 421.3264.

Synthesis of 1-phenylacetoxymethyl-2-(3-(benzylamino)-3-oxoprop-1-yn-1-yl)-12- benzylaminocarboxyl-1,12-dicarba-*closo*-dodecaborane (Va)

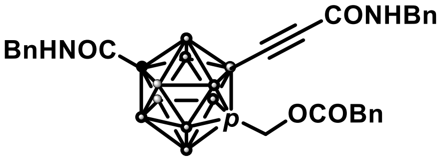

Va

To a solution of **32a** (25.3 mg, 54.5 µmol) and triethylamine (22 µL, 164 µmol) in CH_2Cl_2 (200 µL), was slowly added phenylacetyl chloride (8.6 µL, 65.4 µmol) in CH_2Cl_2 at 0 °C. After the resulting mixture was stirred at room temperature for 9 h, the resulting mixture was concentrated under vacuum. The crude materials were filtered through basic Al_2O_3 pad and purified by column chromatography on silica gel (20% to 40% EtOAc in hexane) afforded **Va** (22.1 mg, 37.9 µmol, 70%) as amorphous. ^{1}H NMR (500 MHz; $CDCl_3$): δ 7.34-7.24 (m, 11H), 7.19 (d, *J* = 6.6 Hz, 2H), 7.12 (d, *J* = 6.8 Hz, 2H), 6.47 (s, 1H), 5.86 (s, 1H), 4.39 (d, *J* = 5.9 Hz, 2H), 4.28 (d, *J* = 5.7 Hz, 2H), 3.58 (s, 2H), 3.10-1.60 (m, 9H); ^{13}C NMR (125 MHz; $CDCl_3$): δ 170.4, 159.9, 152.2, 137.2, 136.8, 133.0, 129.5, 129.0, 128.9, 128.8, 128.1, 128.0, 127.8, 127.52, 127.46, 92,9, 80.1, 79.9, 63.6, 44.8, 43.9, 40.9; ^{11}B NMR (160 MHz; $CDCl_3$): δ -13.6; HRMS (ESI, negative) for $C_{29}H_{34}B_{10}N_2O_4$ (m/z): calculated 619.3178 $(M+Cl)^-$, found 619.3187.

Synthesis of 1-((3-methylbutanoyl)oxy)methyl-2-(3-(benzylamino)-3-oxoprop-1-yn-1-yl)-12-benzylaminocarboxyl-1,12-dicarba-*closo*-dodecaborane (Vb)

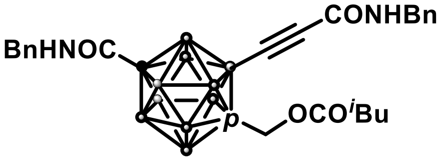

Vb

This compound was prepared from **32a** (25.3 mg, 54.5 µmol) and isovaleryl chloride instead of phenylacetyl chloride using the procedure described above for **Va** in 74% yield as amorphous. ^{1}H NMR (500 MHz; $CDCl_3$): δ 7.36-7.28 (m, 8H), 7.12 (d, *J* = 7.1 Hz, 2H), 6.62 (s, 1H), 5.88 (s, 1H), 4.47 (d, *J* = 5.9 Hz, 2H), 4.29 (d, *J* = 5.7 Hz, 2H), 4.21 (s, 2H), 2.13 (d, *J* = 7.2 Hz, 2H), 1.97-1.92 (m, 1H), 3.00-1.80 (m, 9H), 0.88 (d, *J* = 6.7 Hz, 6H); ^{13}C NMR (125 MHz; $CDCl_3$): δ 171.9, 160.0, 152.3, 137.3, 136.8, 129.0, 128.9, 128.2, 128.1, 127.9, 127.5, 127.3, 93.0, 80.2, 80.1, 63.1, 44.9, 44.0, 42.9, 25.5, 22.5; ^{11}B NMR (160 MHz; $CDCl_3$): δ -13.6; HRMS (ESI, negative) for $C_{26}H_{36}B_{10}N_2O_4$ (m/z): calculated 585.3333 $(M+Cl)^-$, found 585.3344.

Synthesis of 1-phenylacetoxymethyl-2-(3-(isobutylamino)-3-oxoprop-1-yn-1-yl)-12- benzylaminocarboxyl-1,12-dicarba-*closo*-dodecaborane (Vc)

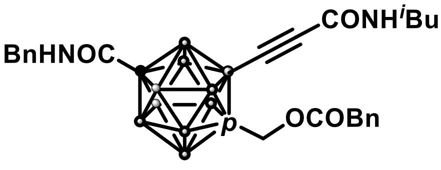

Vc

This compound was prepared from **32b** (23.9 mg, 55.5 µmol) using the procedure described above for **Va** in 55% yield as amorphous. ^{1}H NMR (500 MHz; $CDCl_3$): δ 7.35-7.26 (m, 6H), 7.23 (d, *J* = 6.7 Hz, 2H), 7.13 (d, *J* = 6.7 Hz, 2H), 6.23 (s, 1H), 5.86 (s, 1H), 4.29 (d, *J* = 5.7 Hz, 2H), 4.25 (s, 2H), 3.62 (s, 2H), 3.04 (t, *J* = 6.5 Hz, 2H), 1.73-1.68 (m, 1H), 3.00-1.80 (m, 9H), 0.88 (d, *J* = 6.7 Hz, 6H); ^{13}C NMR (125 MHz; $CDCl_3$): δ 170.5, 160.0, 152.5, 136.8, 133.0, 129.6, 129.1, 128.8, 128.1, 127.6, 127.5, 93.4, 80.1, 90.0, 63.6, 47.2, 44.9, 41.0, 18.4, 20.2; ^{11}B NMR (160 MHz; $CDCl_3$): δ -13.6; HRMS (ESI, negative) for $C_{26}H_{36}B_{10}N_2O_4$ (m/z): calculated 549.3559 $(M+Cl)^-$, found 549.3566.

Synthesis of 1-((3-methylbutanoyl)oxy)methyl-2-(3-(isobutylamino)-3-oxoprop-1-yn-1-yl)-12-benzylaminocarboxyl-1,12-dicarba-*closo*-dodecaborane (Vd)

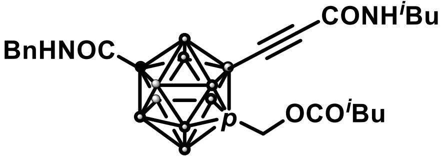

Vd

This compound was prepared from **32b** (23.9 mg, 55.5 µmol) and isovaleryl chloride instead of phenylacetyl chloride using the procedure described above for **Va** in 55% yield as amorphous. ^{1}H NMR (500 MHz; $CDCl_3$): δ 7.33-7.26 (m, 3H), 7.23 (d, *J* = 6.7 Hz, 2H), 7.13 (d, *J* = 6.9 Hz, 2H), 6.36 (s, 1H), 5.88 (s, 1H), 4.30 (d, *J* = 5.6 Hz, 2H), 4.24 (s, 2H), 3.12 (t, *J* = 6.5 Hz, 2H), 2.19 (t, *J* = 7.2 Hz, 2H), 2.07-2.03 (m, 1H), 1.83-1.78 (m, 1H), 3.00-1.80 (m, 9H), 0.945 (d, *J* = 6.7 Hz, 6H), 0.939 (d, *J* = 6.7 Hz, 6H); ^{13}C NMR (125 MHz; $CDCl_3$): δ 171.8, 160.0, 152.6, 136.8, 129.1, 128.1, 127.5, 93.3, 80.2, 80.1, 63.1, 47.3, 44.9, 43.0, 28.5, 25.6, 22.5, 20.2; ^{11}B NMR (160 MHz; $CDCl_3$): δ -13.5; HRMS (ESI, negative) for $C_{23}H_{38}B_{10}N_2O_4$ (m/z): calculated 551.3487 $(M+Cl)^-$, found 551.3502.

Synthesis of 1-phenylacetoxymethyl-2-(3-(benzylamino)-3-oxoprop-1-yn-1-yl)-12-isobutylcarbamoyl-1,12-dicarba-*closo*-dodecaborane (Ve)

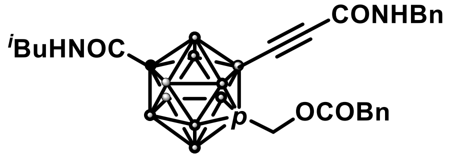

Ve

This compound was prepared from **32c** (24.1 mg, 56.0 μmol) using the procedure described above for **Va** in 71% yield as amorphous. ^{1}H NMR (500 MHz; $CDCl_3$): δ 7.34-7.18 (m, 10H), 6.49 (s, 1H), 5.61 (s, 1H), 4.40 (d, J = 5.9 Hz, 2H), 4.21 (s, 2H), 3.58 (s, 2H), 2.93 (t, J = 6.4 Hz, 2H), 3.05-1.60 (m, 9H), 1.72-1.66 (m, 1H), 0.83 (d, J = 6.7 Hz, 6H); ^{13}C NMR (125 MHz; $CDCl_3$): δ 170.4, 160.0, 152.2, 137.3, 133.0, 129.5, 128.9, 128.8, 128.1, 127.9, 127.6, 92.9, 80.5, 79.7, 63.6, 48.0, 43.9, 41.0, 28.4, 19.9; ^{11}B NMR (160 MHz; $CDCl_3$): δ -13.7; HRMS (ESI, negative) for $C_{26}H_{36}B_{10}N_2O_4$ (m/z): calculated 585.3344 $(M+Cl)^-$, found 585.3334.

Synthesis of 1-((3-methylbutanoyl)oxy)methyl-2-(3-(benzylamino)-3-oxoprop-1-yn-1-yl)-12-isobutylcarbamoyl-1,12-dicarba-*closo*-dodecaborane (Vf)

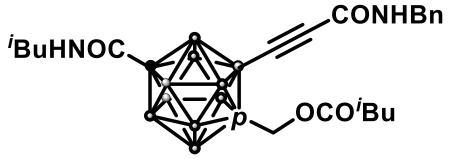

Vf

This compound was prepared from **32c** (24.1 mg, 56.0 μmol) and isovaleryl chloride instead of phenylacetyl chloride using the procedure described above for **Va** in 62% yield as white solid. m.p. 84-86 °C; ^{1}H NMR (500 MHz; $CDCl_3$): δ 7.33-7.28 (m, 5H), 6.64 (s, 1H), 5.62 (s, 1H), 4.48 (d, J = 5.9 Hz, 2H), 4.21 (s, 2H), 2.94 (t, J = 6.4 Hz, 2H), 2.13 (t, J = 7.2 Hz, 2H), 1.97-1.92 (m, 1H), 1.72-1.67 (m, 1H), 3.05-1.60 (m, 9H), 1.72-1.66 (m, 1H), 0.89 (d, J = 6.7 Hz, 6H), 0.83 (d, J = 6.7 Hz, 6H); ^{13}C NMR (125 MHz; $CDCl_3$): δ 171.9, 160.0, 152.3, 137.3, 128.9, 128.2, 127.9, 92.9, 80.5, 80.0, 63.1, 48.0, 44.0, 42.9, 28.4, 25.5, 22.4, 19.9; ^{11}B NMR (160 MHz; $CDCl_3$): δ -13.6; HRMS (ESI, negative) for $C_{26}H_{36}B_{10}N_2O_4$ (m/z): calculated 551.3487 $(M+Cl)^-$, found 551.3510.

Synthesis of 1-phenylacetoxymethyl-2-(3-(isobutylamino)-3-oxoprop-1-yn-1-yl)-12-isobutylcarbamoyl-1,12-dicarba-*closo*-dodecaborane (Vg)

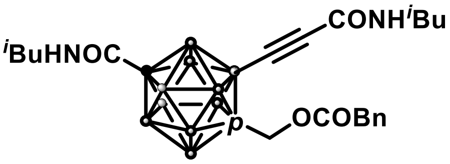

Vg

This compound was prepared from **32d** (21.2 mg, 53.5 μmol) using the procedure described above for **Va** in 54% yield as amorphous. ^{1}H NMR (500 MHz; $CDCl_3$): δ 7.29-7.22 (m, 5H), 6.26 (s, 1H), 5.62 (s, 1H), 4.25 (s, 2H), 3.62 (s, 2H), 3.04 (t, J = 6.5 Hz, 2H), 2.94 (t, J = 6.4 Hz, 2H), 1.74-1.66 (m, 2H), 3.05-1.60 (m, 9H), 0.88 (d, J = 6.7 Hz, 6H), 0.88 (d, J = 6.7 Hz, 6H); ^{13}C NMR (125 MHz; $CDCl_3$): δ 170.5, 160.0, 152.5, 133.0, 129.6, 128.8, 127.6, 93.3, 80.5, 79.8, 63.6, 48.0, 47.2, 41.0, 28.40, 28.36, 20.2, 19.9, ; ^{11}B NMR (160 MHz; $CDCl_3$): δ -13.7; HRMS (ESI, negative) for $C_{23}H_{38}B_{10}N_2O_4$ (m/z): calculated 551.3498 $(M+Cl)^-$, found 551.3485.

Synthesis of 1-((3-methylbutanoyl)oxy)methyl-2-(3-(isobutylamino)-3-oxoprop-1-yn-1-yl)-12-isobutylcarbamoyl-1,12-dicarba-*closo*-dodecaborane (Vh)

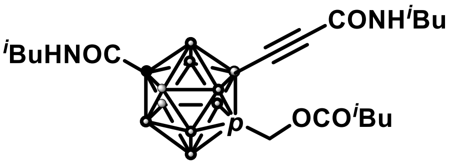

Vh

This compound was prepared from **32d** (21.2 mg, 53.5 μmol) and isovaleryl chloride instead of phenylacetyl chloride using the procedure described above for **Va** in 48% yield as amorphous.

^{1}H NMR (500 MHz; $CDCl_3$): δ 6.37 (s, 1H), 5.61 (s, 1H), 4.24 (s, 2H), 3.13 (t, J = 6.5 Hz, 2H), 2.94 (t, J = 6.4 Hz, 2H),

2.20 (d, J = 7.2 Hz, 2H), 2.09-2.03 (m, 1H), 1.84-1.79 (m, 1H), 1.73-1.68 (m, 1H), 3.00-1.50 (m, 9H), 0.95 (d, J = 6.7 Hz, 6H), 0.94 (d, J = 6.7 Hz, 6H), 0.84 (d, J = 6.8 Hz, 6H); ^{13}C NMR (125 MHz; $CDCl_3$): δ 171.9, 160.0, 152.6, 93.3, 80.5, 80.0, 63.1, 48.1, 47.3, 43.0, 28.5, 28.4, 25.6, 22.5, 20.2, 19.9; ^{11}B NMR (160 MHz; $CDCl_3$): δ -13.6; HRMS (ESI, negative) for $C_{20}H_{40}B_{10}N_2O_4$ (m/z): calculated 517.3641 $(M+Cl)^-$, found 517.3640.

Synthesis of 12-phenylacetoxymethyl-2-(3-(benzylamino)-3-oxoprop-1-yn-1-yl)-1- benzylaminocarboxyl-1,12-dicarba-*closo*-dodecaborane (Vi)

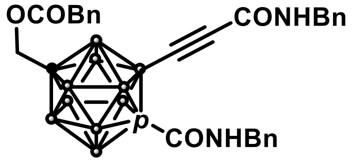

Vi

This compound was prepared from **36a** (13.8 mg, 29.6 μmol) using the procedure described above for **Va** in quantitative yield as white solid. m.p. 126-128 °C; ^{1}H NMR (500 MHz; $CDCl_3$): δ 7.34-7.15 (m, 10), 6.20 (s, 1H), 6.15 (s, 1H), 4.38 (d, J = 6.0 Hz, 2H), 4.30 (d, J = 5.8 Hz, 2H), 3.98 (s, 2H), 3.57 (s, 2H), 3.00-1.80 (m, 9H); ^{13}C NMR (125 MHz; $CDCl_3$): δ 170.0, 159.4, 152.1, 137.1, 136.9, 133.0, 129.5, 129.0, 128.9, 128.8, 128.1, 128.0, 127.9, 127.63, 127.57, 127.5, 92.7, 80.7, 79.3, 65.1, 60.5, 44.9, 44.0, 41.0; ^{11}B NMR (160 MHz; $CDCl_3$): δ -13.9; HRMS (ESI, negative) for $C_{29}H_{34}B_{10}N_2O_4$ (m/z): calculated 619.3178 $(M+Cl)^-$, found 619.3186.

Synthesis of 12-((3-methylbutanoyl)oxy)methyl-2-(3-(benzylamino)-3-oxoprop-1-yn-1-yl)-1-benzylaminocarboxyl-1,12-dicarba-*closo*-dodecaborane (Vj)

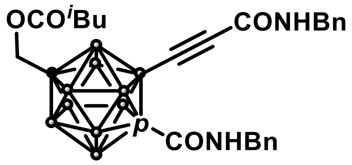

Vj

This compound was prepared from **36a** (13.8 mg, 29.6 μmol) and isovaleryl chloride instead of phenylacetyl chloride using the procedure described above for **Va** in 49% yield as white solid. m.p. 133-135 °C; ^{1}H NMR (500 MHz; $CDCl_3$): δ 7.28-7.17 (m, 10), 6.12 (s, 1H), 5.90 (s, 1H), 4.40 (d, J = 6.0 Hz, 2H), 4.33 (d, J = 5.8 Hz, 2H), 3.98 (s, 2H), 2.16 (d, J = 7.1 Hz, 2H), 2.08-2.03 (m, 1H), 3.00-1.50 (m, 9H), 0.94 (d, J = 6.6 Hz, 6H); ^{13}C NMR (125 MHz; $CDCl_3$): δ 171.5, 159.4, 152.1, 137.1, 137.0, 129.01, 128.96, 128.1, 128.01, 127.95, 127.7, 127.5, 92.8, 80.7, 79.5, 64.8, 44.9, 44.0, 42.9, 25.6, 22.5; ^{11}B NMR (160 MHz; $CDCl_3$): δ -13.8; HRMS (ESI, negative) for $C_{26}H_{36}B_{10}N_2O_4$ (m/z): calculated 549.3559 $(M-H)^-$, found 549.3561.

Synthesis of 12-phenylacetoxymethyl-2-(3-(isobutylamino)-3-oxoprop-1-yn-1-yl)-1- benzylaminocarboxyl-1,12-dicarba-*closo*-dodecaborane (Vk)

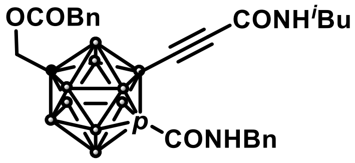

Vk

This compound was prepared from **36b** (14.5 mg, 33.6 μmol) using the procedure described above for **Va** in 83% yield as amorphous. ^{1}H NMR (500 MHz; $CDCl_3$): δ 7.29-7.18 (m, 10H), 6.15 (s, 1H), 5.74 (s, 1H), 4.35 (d, J = 5.7 Hz, 2H), 3.99 (s, 2H), 3.59 (s, 2H), 3.05 (t, J = 6.6 Hz, 2H), 1.78-1.73 (m, 1H), 3.00-1.50 (m, 9H), 0.90 (d, J = 6.7 Hz, 6H); ^{13}C NMR (125 MHz; $CDCl_3$): δ 170.0, 159.4, 152.3, 136.9, 133.0, 129.5, 129.0, 128.8, 127.9, 127.6, 127.5, 93.1, 80.8, 79.3, 65.1, 47.3, 44.9, 41.0, 28.5, 20.2; ^{11}B NMR (160 MHz; $CDCl_3$): δ -13.5; HRMS (ESI, negative) for $C_{26}H_{36}B_{10}N_2O_4$ (m/z): calculated 585.3333 $(M+Cl)^-$, found 585.3342.

Synthesis of 12-((3-methylbutanoyl)oxy)methyl-2-(3-(isobutylamino)-3-oxoprop-1-yn-1-yl)-1-benzylaminocarboxyl-1,12-dicarba-*closo*-dodecaborane (Vl)

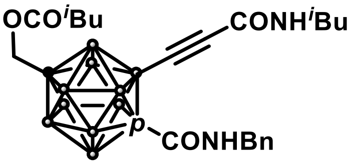

Vl

This compound was prepared from **36c** (14.5 mg, 33.6 μmol) and isovaleryl chloride instead of phenylacetyl chloride using the procedure described above for **Va** in 44% yield as amorphous. ^{1}H NMR (500 MHz; $CDCl_3$): δ 7.33-7.20 (m, 3H), 7.20 (d, *J* = 7.1 Hz, 2H), 6.14 (s, 1H), 5.71 (s, 1H), 4.36 (d, *J* = 5.8 Hz, 2H), 3.99 (s, 2H), 3.05 (t, *J* = 6.6 Hz, 2H), 2.17 (d, *J* = 7.2 Hz, 2H), 2.10-2.03 (m, 1H), 1.78-1.72 (m, 1H), 3.00-1.50 (m, 9H), 0.94 (d, *J* = 6.7 Hz, 6H), 0.90 (d, *J* = 6.7 Hz, 6H); ^{13}C NMR (125 MHz; $CDCl_3$): δ 171.5, 159.5, 152.3, 136.9, 129.0, 127.9, 127.6, 93.1, 80.7, 79.5, 64.8, 47.3, 44.9, 42.9, 28.5, 25.6, 22.5, 20.2; ^{11}B NMR (160 MHz; $CDCl_3$): δ -13.5; HRMS (ESI, negative) for $C_{23}H_{38}B_{10}N_2O_4$ (m/z): calculated 551.3487 $(M+Cl)^-$, found 551.3506.

Synthesis of 12-phenylacetoxymethyl-2-(3-(benzylamino)-3-oxoprop-1-yn-1-yl)-1-isobutylcarbamoyl-1,12-dicarba-*closo*-dodecaborane (Vm)

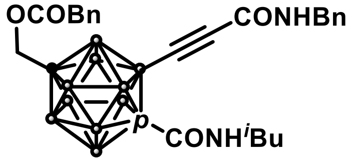

Vm

This compound was prepared from **36c** (13.8 mg, 32.1 μmol) using the procedure described above for **Va** in 51% yield as white solid. m.p. 112-115 °C; ^{1}H NMR (500 MHz; $CDCl_3$): δ 7.37-7.23 (m, 10H), 6.16 (s, 1H), 5.82 (s, 1H), 4.46 (d, *J* = 5.9 Hz, 2H), 3.99 (s, 2H), 3.58 (s, 2H), 2.96 (t, *J* = 6.4 Hz, 2H), 1.74-1.67 (m, 1H), 3.00-1.80 (m, 9H), 0.81 (d, *J* = 6.7 Hz, 6H); ^{13}C NMR (125 MHz; $CDCl_3$): δ 170.0, 159.2, 152.1, 137.1, 133.0, 129.5, 129.0, 128.8, 128.2, 128.1, 127.6, 92.5, 81.3, 79.1, 48.2, 44.1, 41.0, 28.4, 20.0; ^{11}B NMR (160 MHz; $CDCl_3$): δ -13.8; HRMS (ESI, negative) for $C_{26}H_{36}B_{10}N_2O_4$ (m/z): calculated 585.3333 $(M+Cl)^-$, found 585.3339.

Synthesis of 12-((3-methylbutanoyl)oxy)methyl-2-(3-(benzylamino)-3-oxoprop-1-yn-1-yl)-1-isobutylcarbamoyl-1,12-dicarba-*closo*-dodecaborane (Vn)

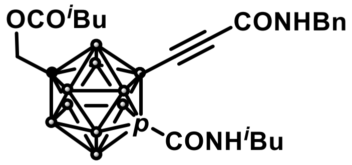

Vn

This compound was prepared from **36d** (13.8 mg, 32.1 μmol) and isovaleryl chloride instead of phenylacetyl chloride using the procedure described above for **Va** in 42% yield as white solid. m.p. 116-118 °C; ^{1}H NMR (500 MHz; $CDCl_3$): δ 7.36-7.27 (m, 5H), 6.16 (s, 1H), 5.83 (s, 1H), 4.46 (d, *J* = 5.9 Hz, 2H), 3.98 (s, 2H), 3.58 (s, 2H), 2.96 (t, *J* = 6.4 Hz, 2H), 2.16 (d, *J* = 7.1 Hz, 2H), 2.08-2.03 (m, 1H), 1.73-1.67 (m, 1H), 3.00-1.80 (m, 9H), 0.94 (d, *J* = 6.6 Hz, 6H), 0.82 (d, *J* = 6.7 Hz, 6H); ^{13}C NMR (125 MHz; $CDCl_3$): δ 171.5, 159.3, 152.1, 137.1, 129.0, 128.2, 128.1, 92.5, 81.2, 79.3, 64.7, 48.2, 44.1, 42.9, 28.5, 25.6, 22.5, 20.0; ^{11}B NMR (160 MHz; $CDCl_3$): δ -13.3; HRMS (ESI, negative) for $C_{23}H_{38}B_{10}N_2O_4$ (m/z): calculated 551.3487 $(M+Cl)^-$, found 551.3498.

Synthesis of 12-phenylacetoxymethyl-2-(3-(isobutylamino)-3-oxoprop-1-yn-1-yl)-1-isobutylcarbamoyl-1,12-dicarba-*closo*-dodecaborane (Vo)

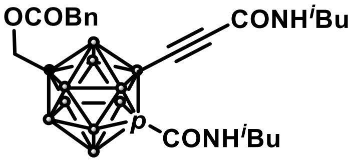

Vo

This compound was prepared from **36d** (14.6 mg, 36.8 μmol) using the procedure described above for **Va** in 67% yield as white solid. m.p. 115-119 °C; ^{1}H NMR (500 MHz; $CDCl_3$): δ 7.36-7.23 (m, 5H), 5.93 (s, 1H), 5.86 (s, 1H), 3.99 (s, 2H), 3.59 (s, 2H), 3.11 (t, J = 6.6 Hz, 2H), 2.99 (t, J = 6.4 Hz, 2H), 1.82-1.72 (m, 2H), 3.00-1.80 (m, 9H), 0.93 (d, J = 6.7 Hz, 6H), 0.85 (d, J = 6.7 Hz, 6H); ^{13}C NMR (125 MHz; $CDCl_3$): δ 170.0, 159.3, 152.4, 133.0, 129.5, 128.8, 127.6, 92.9, 81.3, 79.1, 65.1, 48.3, 47.3, 41.0, 28.51, 28.46, 21.2, 20.0; ^{11}B NMR (160 MHz; $CDCl_3$): δ -13.9; HRMS (ESI, negative) for $C_{23}H_{38}B_{10}N_2O_4$ (m/z): calculated 551.3487 $(M+Cl)^-$, found 551.3495.

Synthesis of 12-((3-methylbutanoyl)oxy)methyl-2-(3-(isobutylamino)-3-oxoprop-1-yn-1-yl)-1-isobutylcarbamoyl-1,12-dicarba-*closo*-dodecaborane (Vp)

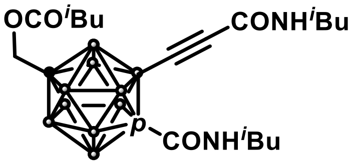

Vp

This compound was prepared from **36d** (14.6 mg, 36.8 μmol) and isovaleryl chloride instead of phenylacetyl chloride using the procedure described above for **VaS** in 25% yield as white solid. m.p. 93-94 °C; ^{1}H NMR (500 MHz; $CDCl_3$): δ 5.91 (s, 1H), 5.86 (s, 1H), 3.99 (s, 2H), 3.59 (s, 2H), 3.11 (t, J = 6.6 Hz, 2H), 3.00 (t, J = 6.4 Hz, 2H), 2.17 (d, J = 7.0 Hz, 2H), 2.10-2.04 (m, 1H), 1.82-1.71 (m, 2H), 3.00-1.80 (m, 9H), 0.94 (d, J = 6.7 Hz, 6H), 0.92 (d, J = 6.8 Hz, 6H), 0.86 (d, J = 6.7 Hz, 6H); ^{13}C NMR (125 MHz; $CDCl_3$): δ 171.5, 159.3, 152.4, 92.8, 81.2, 79.3, 64.8, 48.3, 47.3, 42.9, 28.51, 28.47, 25.6, 22.5, 20.2, 20.0; ^{11}B NMR (160 MHz; $CDCl_3$): δ -13.9; HRMS (ESI, negative) for $C_{20}H_{40}B_{10}N_2O_4$ (m/z): calculated 517.3641 $(M+Cl)^-$, found 517.3649.

2. X-ray Crystallography

For each scaffold, the absolute structures were determined by X-ray structural analysis as described in the synthesis section.

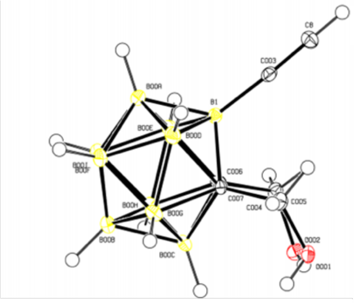

Scaffold I

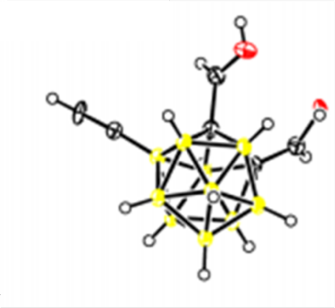

Scaffold II

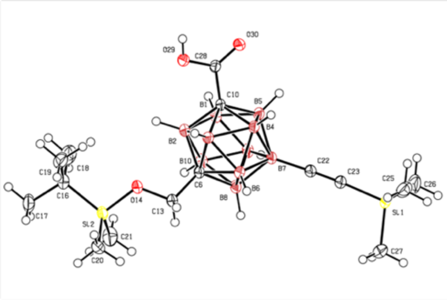

Scaffold III

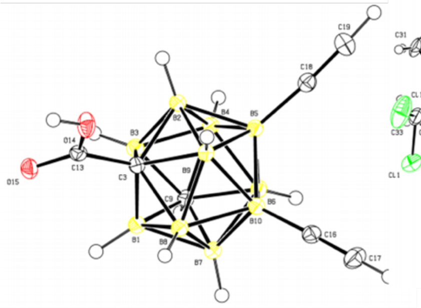

Scaffold IV

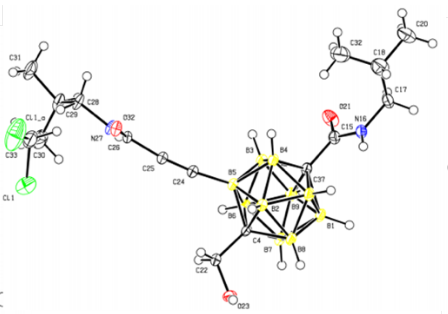

Scaffold V-1

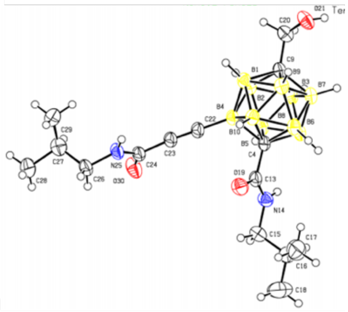

Scaffold V-2

Table S1 Crystal data and structure refinement

Compound	**4** (Scaffold I)	**14** (Scaffold II)
Identification code	CRL-26-1263	CRL261328-2_autored
Empirical formula	$C_6H_{16}B_{10}O_2$	$C_6H_{16}B_{10}O_2$
Formula weight	228.29	228.29
Temperature/K	90	90
Crystal system	orthorhombic	monoclinic
Space group	$Pna2_1$	I2/a
a/Å	12.0547(4)	24.7677(3)
b/Å	13.7982(4)	15.2606(2)
c/Å	7.4135(2)	26.7524(3)
α/°	90	90
β/°	90	100.1710(10)
γ/°	90	90
Volume/Å^3	1233.11(6)	9952.7(2)
Z	4	32
ρ_{calc}g/cm^3	1.230	1.219
μ/mm^{-1}	0.510	0.506
F(000)	472.0	3776.0
Crystal size/mm^3	0.163 × 0.074 × 0.053	0.27 × 0.078 × 0.041
Radiation	Cu Kα (λ = 1.54184)	Cu Kα (λ = 1.54184)
2Θ range for data collection/°	9.742 to 153.358	6.694 to 153.06
Index ranges	$-14 \leq h \leq 14$, $-15 \leq k \leq 17$, $-8 \leq l \leq 3$	$-31 \leq h \leq 31$, $-18 \leq k \leq 18$, $-33 \leq l \leq 33$
Reflections collected	4182	72811
Independent reflections	1774 [R_{int} = 0.0262, R_{sigma} = 0.0301]	10199 [R_{int} = 0.0453, R_{sigma} = 0.0202]
Data/restraints/parameters	1774/326/165	10199/2105/822
Goodness-of-fit on F^2	1.081	1.133
Final R indexes [I>=2σ (I)]	R_1 = 0.0598, wR_2 = 0.1571	R_1 = 0.2344, wR_2 = 0.6794
Final R indexes [all data]	R_1 = 0.0615, wR_2 = 0.1586	R_1 = 0.2370, wR_2 = 0.6856
Largest diff. peak/hole / e Å^{-3}	1.00/-0.91	1.97/-1.92

Table S1 Crystal data and structure refinement (continued)

Compound	**18** (Scaffold III)	**25** (Scaffold IV)
Identification code	CRL-26-1114_3	CRL-26-1021
Empirical formula	$C_{15}H_{36}B_{10}O_3Si_2$	$C_7H_{12}B_{10}O_2$
Formula weight	428.72	236.27
Temperature/K	90	293(2)
Crystal system	monoclinic	triclinic
Space group	I2/a	P-1
a/Å	11.4845(4)	6.9823(4)
b/Å	12.7847(5)	7.3010(4)
c/Å	36.4855(13)	13.2381(8)
α/°	90	76.236(5)
β/°	95.462(3)	83.750(5)
γ/°	90	89.704(4)
Volume/$Å^3$	5332.7(3)	651.42(7)
Z	8	2
$\rho_{calc}g/cm^3$	1.068	1.205
μ/mm^{-1}	1.296	0.510
F(000)	1824.0	240.0
Crystal size/mm^3	0.297 × 0.053 × 0.046	0.121 × 0.119 × 0.071
Radiation	Cu Kα (λ = 1.54184)	CuKα (λ = 1.54184)
2Θ range for data collection/°	7.33 to 149.398	6.918 to 152.73
Index ranges	-14 ≤ h ≤ 14, -15 ≤ k ≤ 12, -44 ≤ l ≤ 43	-8 ≤ h ≤ 8, -9 ≤ k ≤ 8, -10 ≤ l ≤ 16
Reflections collected	16339	6996
Independent reflections	5168 [R_{int} = 0.0217, R_{sigma} = 0.0236]	2599 [R_{int} = 0.0265, R_{sigma} = 0.0309]
Data/restraints/parameters	5168/0/280	2599/0/217
Goodness-of-fit on F^2	1.052	1.069
Final R indexes [I>=2σ (I)]	R_1 = 0.0397, wR_2 = 0.1047	R_1 = 0.0360, wR_2 = 0.0957
Final R indexes [all data]	R_1 = 0.0435, wR_2 = 0.1073	R_1 = 0.0397, wR_2 = 0.0988
Largest diff. peak/hole / e $Å^{-3}$	0.59/-0.32	0.24/-0.32

Table S1 Crystal data and structure refinement (continued)

Compound	**32d** (Scaffold V-1)	**36d** (Scaffold V-2)
Identification code	CRL-26-1132_2	CRL-26-1134_4
Empirical formula	$C_{31}H_{66}B_{20}Cl_2N_4O_6$	$C_{15}H_{32}B_{10}N_2O_3$
Formula weight	877.97	396.52
Temperature/K	90	90
Crystal system	monoclinic	triclinic
Space group	C2/c	P-1
a/Å	29.1281(13)	9.1672(5)
b/Å	8.7234(4)	11.8780(5)
c/Å	20.1283(9)	12.9840(5)
α/°	90	105.803(4)
β/°	108.963(5)	102.443(4)
γ/°	90	111.614(4)
Volume/$Å^3$	4836.9(4)	1183.29(10)
Z	4	2
$\rho_{calc}g/cm^3$	1.206	1.113
μ/mm^{-1}	1.543	0.514
F(000)	1848.0	420.0
Crystal size/mm^3	0.186 × 0.177 × 0.124	0.342 × 0.1 × 0.055
Radiation	Cu Kα (λ = 1.54184)	Cu Kα (λ = 1.54184)
2Θ range for data collection/°	6.418 to 149.99	7.564 to 149.972
Index ranges	-36 ≤ h ≤ 34, -10 ≤ k ≤ 10, -25 ≤ l ≤ 22	-11 ≤ h ≤ 11, -14 ≤ k ≤ 13, -15 ≤ l ≤ 15
Reflections collected	14464	13384
Independent reflections	4740 [R_{int} = 0.0472, R_{sigma} = 0.0388]	4674 [R_{int} = 0.0308, R_{sigma} = 0.0373]
Data/restraints/parameters	4740/486/290	4674/0/285
Goodness-of-fit on F^2	1.041	1.075
Final R indexes [I>=2σ (I)]	R_1 = 0.0906, wR_2 = 0.2566	R_1 = 0.0552, wR_2 = 0.1535
Final R indexes [all data]	R_1 = 0.0939, wR_2 = 0.2612	R_1 = 0.0642, wR_2 = 0.1617
Largest diff. peak/hole / e $Å^{-3}$	1.71/-1.37	1.03/-0.32

Table S1 Crystal data and structure refinement (continued)

Compound	**22**
Identification code	CRL64-1-2
Empirical formula	$C_2H_9B_{10}I_3$
Formula weight	521.89
Temperature/K	293(2)
Crystal system	monoclinic
Space group	$P2_1/c$
a/Å	8.6488(2)
b/Å	12.1509(2)
c/Å	12.6613(2)
α/°	90
β/°	89.949(2)
γ/°	90
Volume/Å^3	1330.58(4)
Z	4
ρ_{calc}g/cm^3	2.605
μ/mm^{-1}	54.923
F(000)	920.0
Crystal size/mm^3	? × ? × ?
Radiation	Cu Kα (λ = 1.54184)
2Θ range for data collection/°	10.09 to 151.506
Index ranges	-10 ≤ h ≤ 10, -9 ≤ k ≤ 15, -15 ≤ l ≤ 14
Reflections collected	4669
Independent reflections	2191 [R_{int} = 0.0321, R_{sigma} = 0.0331]
Data/restraints/parameters	2191/270/136
Goodness-of-fit on F^2	1.184
Final R indexes [I>=2σ (I)]	R_1 = 0.0560, wR_2 = 0.1538
Final R indexes [all data]	R_1 = 0.0702, wR_2 = 0.1998
Largest diff. peak/hole / e Å^{-3}	2.22/-3.33

3. Pharmacophore fitting toward RABV inhibitors

To predict conformation among RABV inhibitors, a pharmacophore model was constructed from compounds **Va-h** (Scaffold V-1) using Ligand-Based Pharmacophores option of LigandScout 4.4 (InteLigand GmbH). Then, three pharmacophore features were used including hydrophobic (Hy) moity, a hydrogen bond acceptor (HA), donor (HD). For pharmacophore evaluation, the scoring function was set to 'Relative Pharmacophore-Fit'. For alll other parameters, default values were used. Conformations of compounds generated by 'iCon best' option of LigandScout 4.4 were fitted to the pharmacophore and visualized.

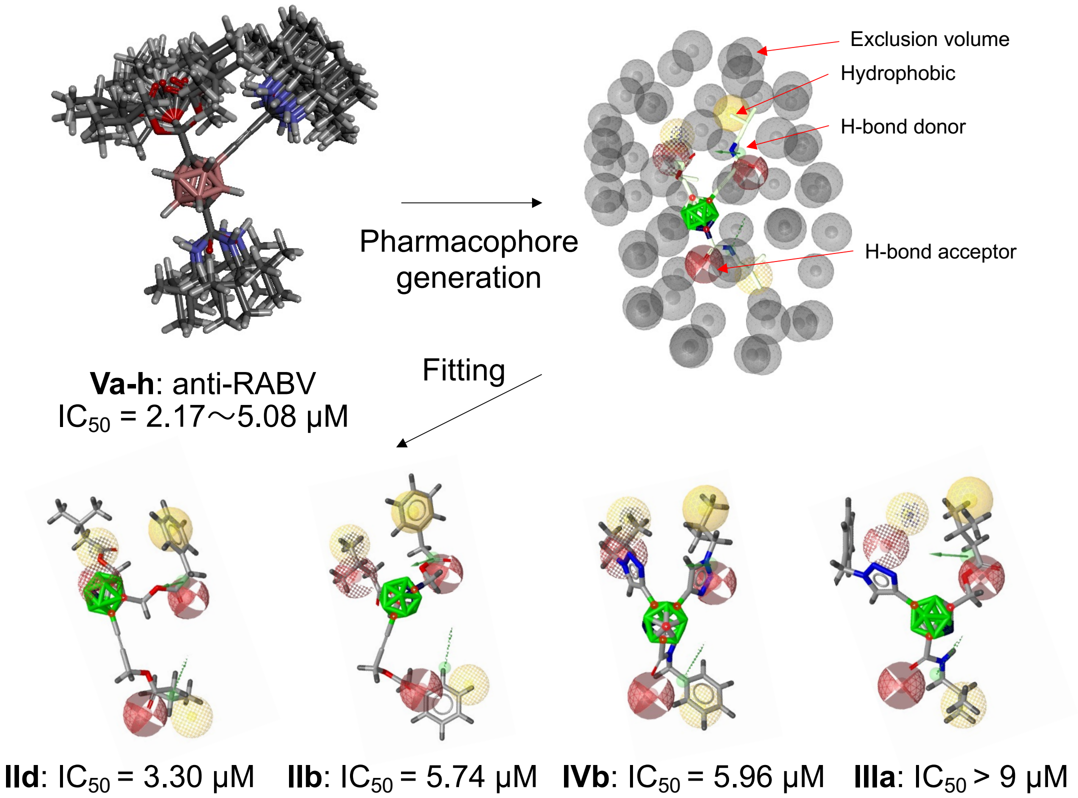

Figure S1. Comparison of conformation of RABV inhibitors.

4. Inhibitory activity of Vj against luciferase

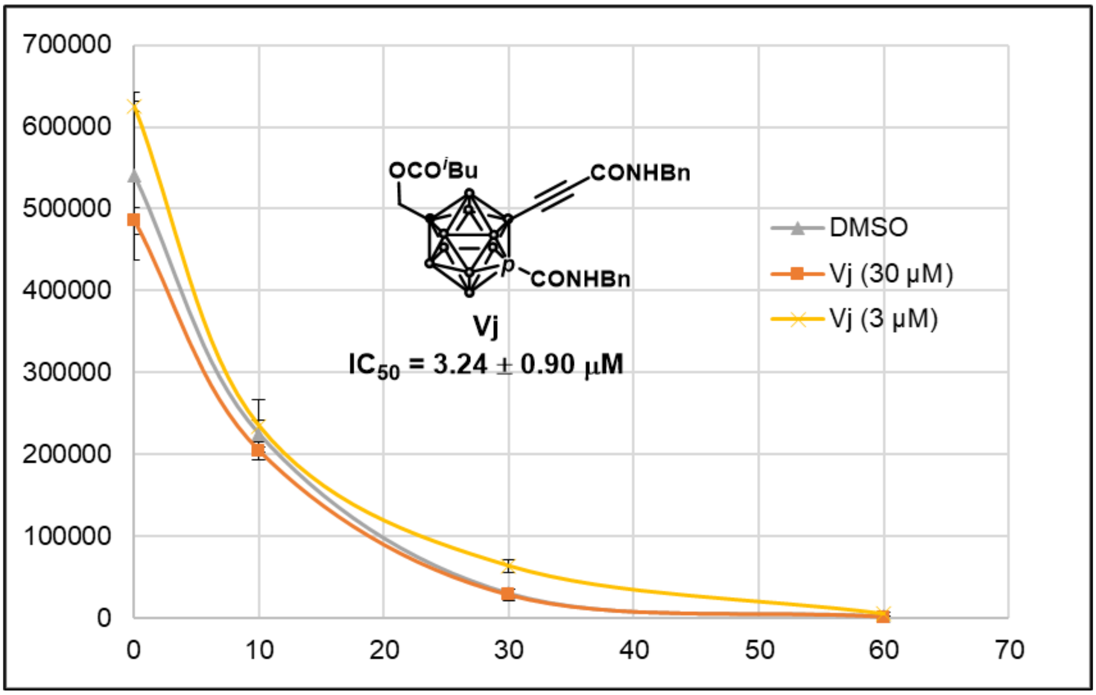

Figure S2. Inhibitory activity of compound **Vj** against luciferase. After luciferase was incubated at 37 °C for indicated time with (or without) the treatment of 30 μM or 3 μM **Vj**, luciferase assay reagent was added, and the emission intensity was measured.[5]

5. Western Blot and RT-PCR with HIF inhibitors

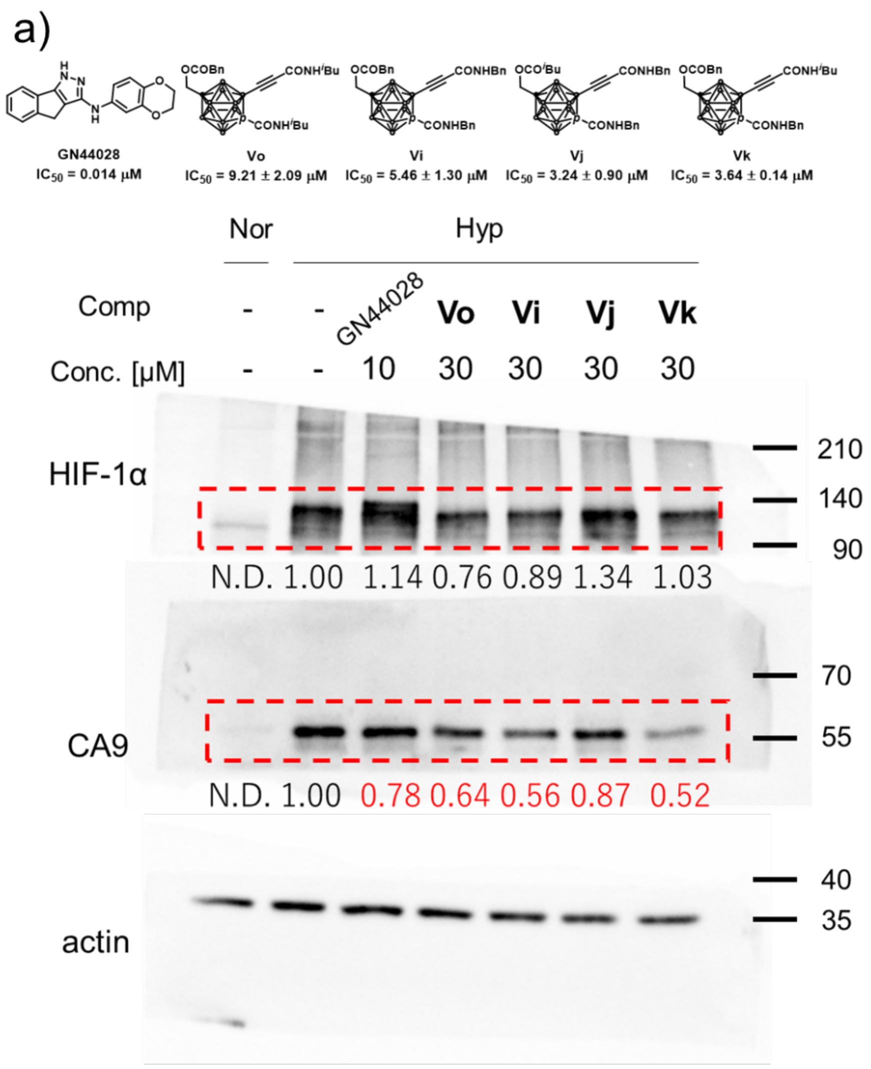

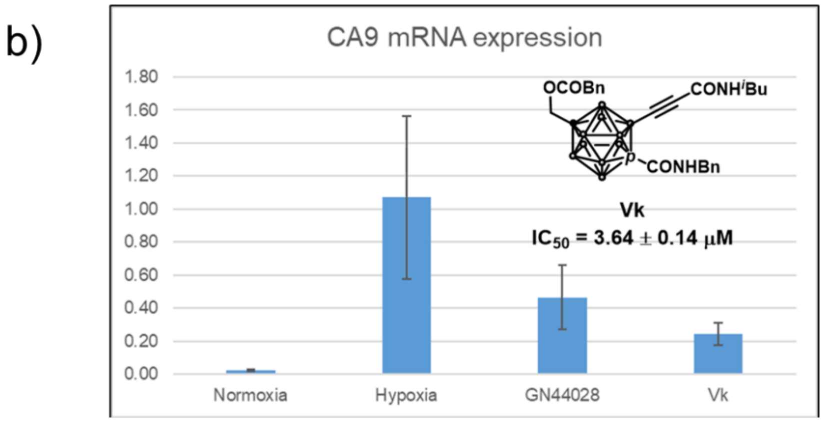

Figure S3. Reduction of HIF-1α regulating protein CA9 by HIF inhibitors; a) Effects of HIF inhibitors on HIF-1α and CA9 protein accumulation. Protein accumulation was detected by immunoblot analysis with the specific antibodies after HeLa cells treated with 1.0 μM compounds for 12 h. The ratio was calculated based on vehicle intensity. GN44028 (**GN**)[4] was used as a positive control; b) mRNA level of CA9 was detected by RT-PCR (n = 3).

6. Rabies viral protein accumulation induced by compound Vp

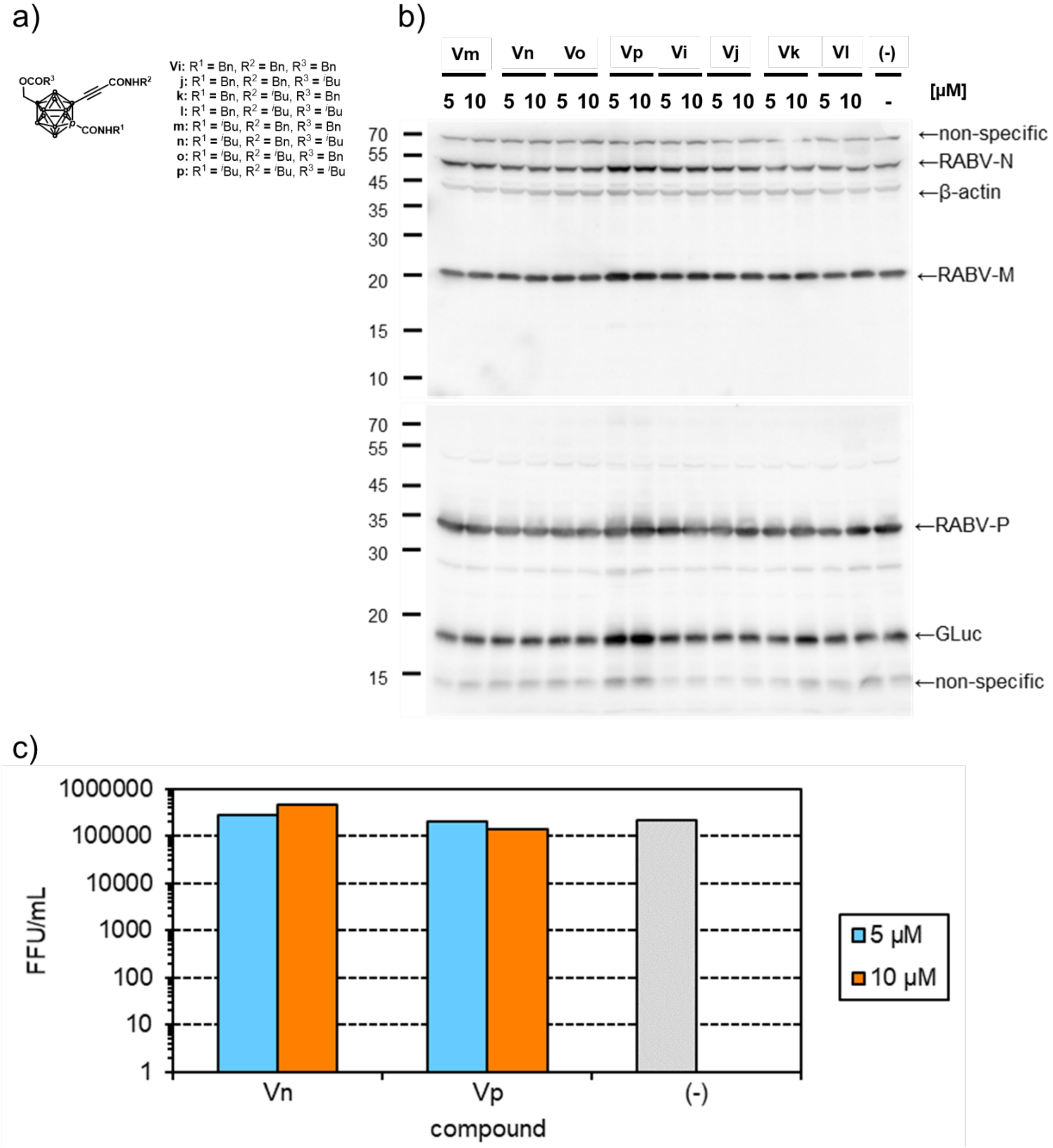

Figure S4. Immunoblotting analyses of rabies virus protein and Gaussia luciferase (GLuc) in N2a cells pre-incubated with Rabies virus RNA fused with GLuc reporter. Protein accumulation was detected with the specific antibodies after cells treated with or without indicated concentration of compounds **Vi-p** for 48 h; c) virus titer after treatment of compounds.

7. PMI analysis

To validate that the synthesized compounds cover the unique three-dimensional chemical space, the possible conformations of trisubstituted carborane were calculated using the 'iCon fast' option of LigandScout 4.4. Also, a normalized PMI analysis of them was performed.

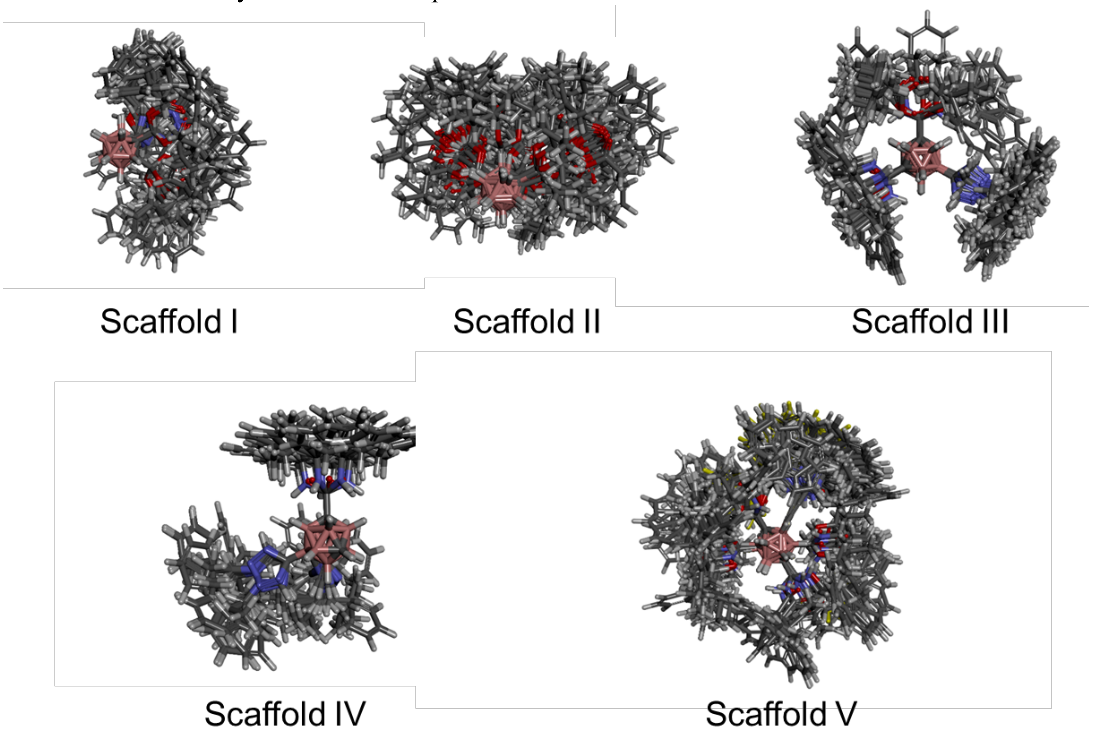

Figure S5. Generated conformations of each scaffold.

8. Reference

(1) Yamazaki, H.; Ohta, K.; Endo, Y. Regioselective Synthesis of Triiodo-o-Carboranes and Tetraiodo-o-Carborane. *Tetrahedron Lett.* **2005**, *46* (17), 3119–3122.

(2) Kramer, R.; Berkenbusch, T.; Brückner, R. Stereocomplementary Desymmetrizations of Divinylcarbinols by Zirconium(IV)- vs. Titanium(IV)-Mediated Asymmetric Epoxidations. *Adv. Synth. Catal.* **2008**, *350* (7–8), 1131–1148.

(3) Himmelspach, A.; Finze, M. Dicarba-*Closo*-Dodecaboranes with One and Two Ethynyl Groups Bonded to Boron. *Eur. J. Inorg. Chem.* **2010**, *10* (13), 2012–2024.

(4) Minegishi, H.; Fukashiro, S.; Ban, H. S.; Nakamura, H. Discovery of Indenopyrazoles as a New Class of Hypoxia Inducible Factor (HIF)-1 Inhibitors. *ACS Med. Chem. Lett.* 2013, pp 297–301.

(5) Fuse, S.; Suzuki, K.; Kuchimaru, T.; Kadonosono, T.; Ueda, H.; Sato, S.; Kizaka-Kondoh, S.; Nakamura, H. Design, Synthesis, and Evaluation of Indeno[2,1-c]Pyrazolones for Use as Inhibitors against Hypoxia-Inducible Factor (HIF)-1 Transcriptional Activity. *Bioorg. Med. Chem.* **2020**, *28* (1), 115207.

9. NMR Spectra

3-(2-(trimethylsilyl)prop-1-yn-1-yl)-1,2-dicarba-*closo*-dodecaborane (1)

^{1}H NMR (500 MHz; $CDCl_3$)

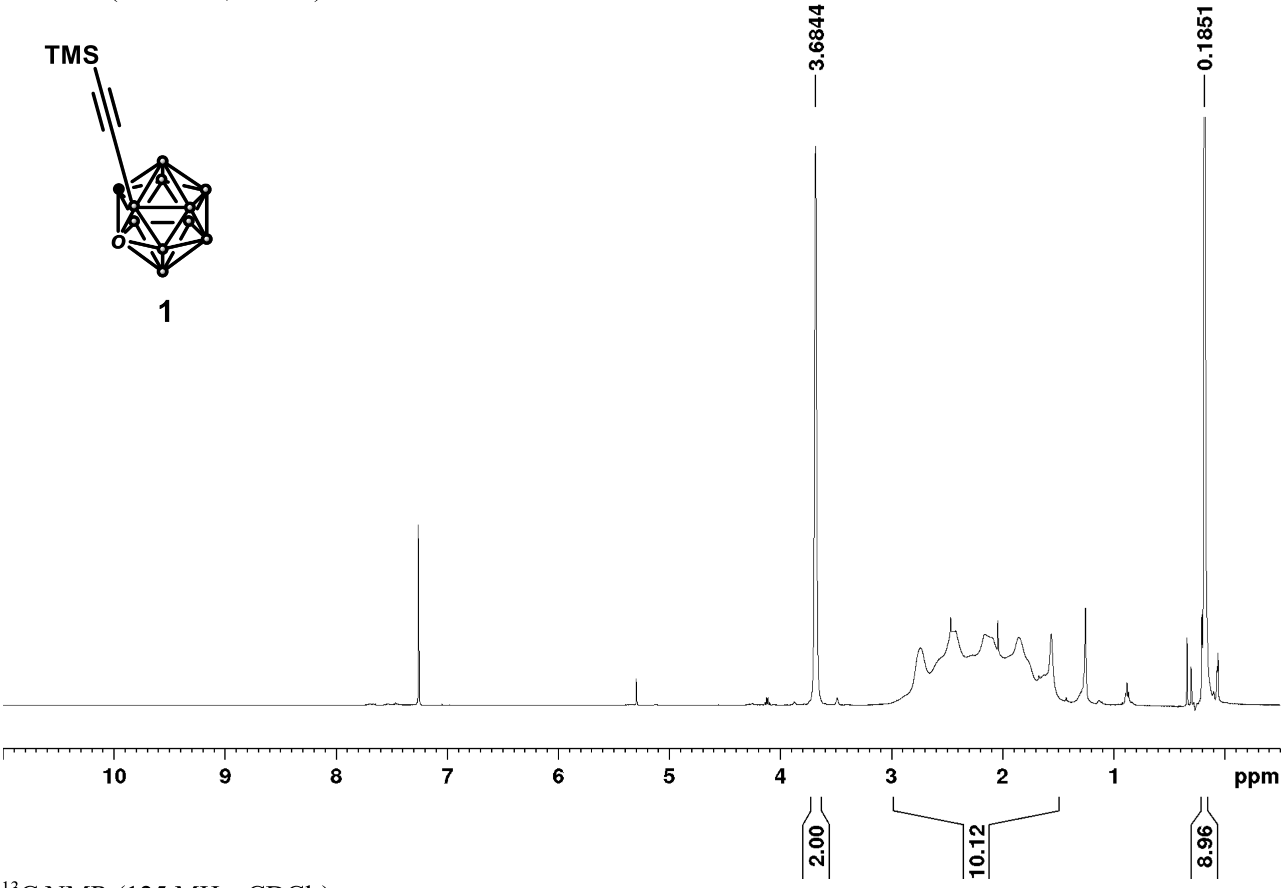

^{13}C NMR (125 MHz; $CDCl_3$)

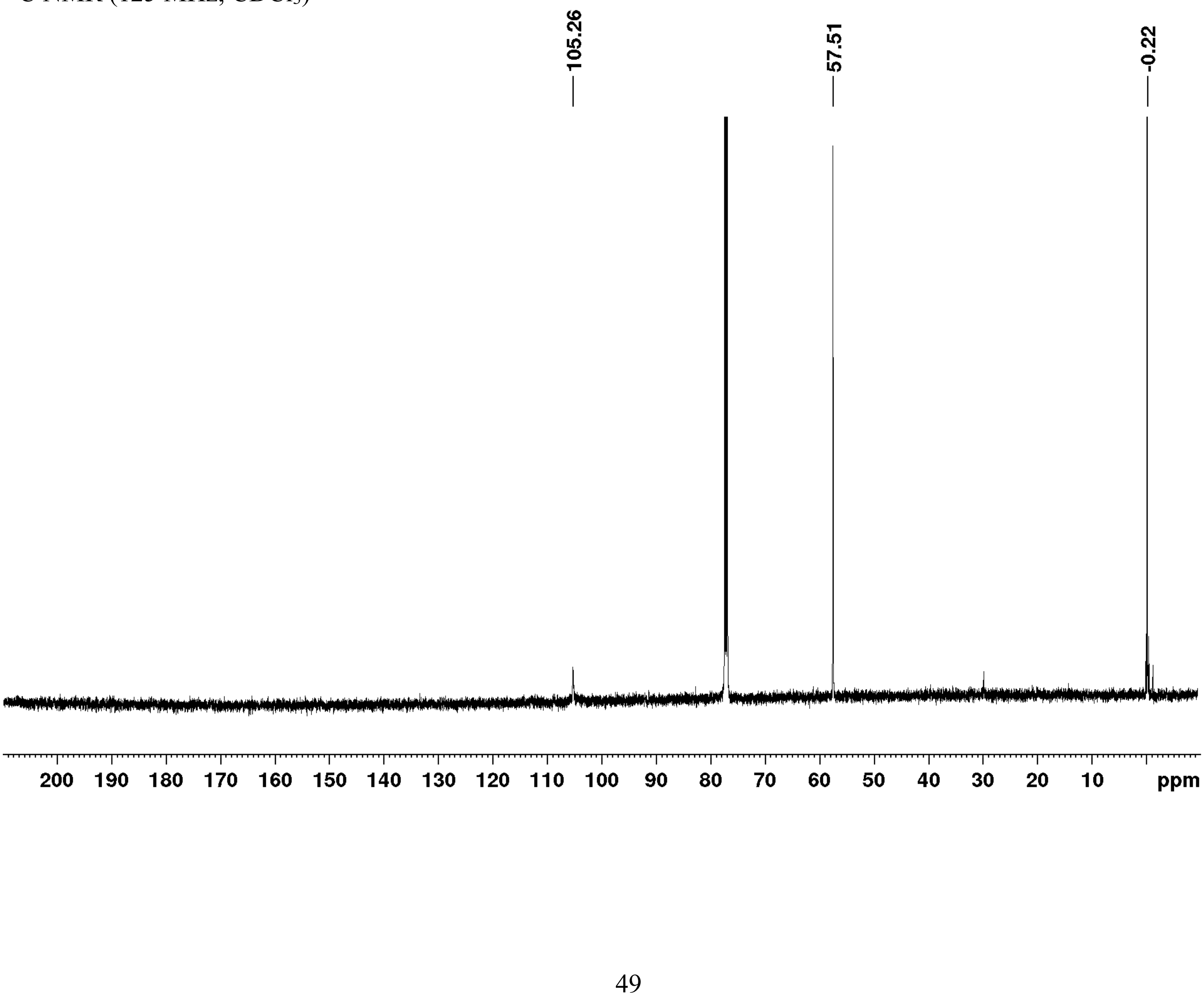

^{11}B NMR (160 MHz; CDCl$_3$)

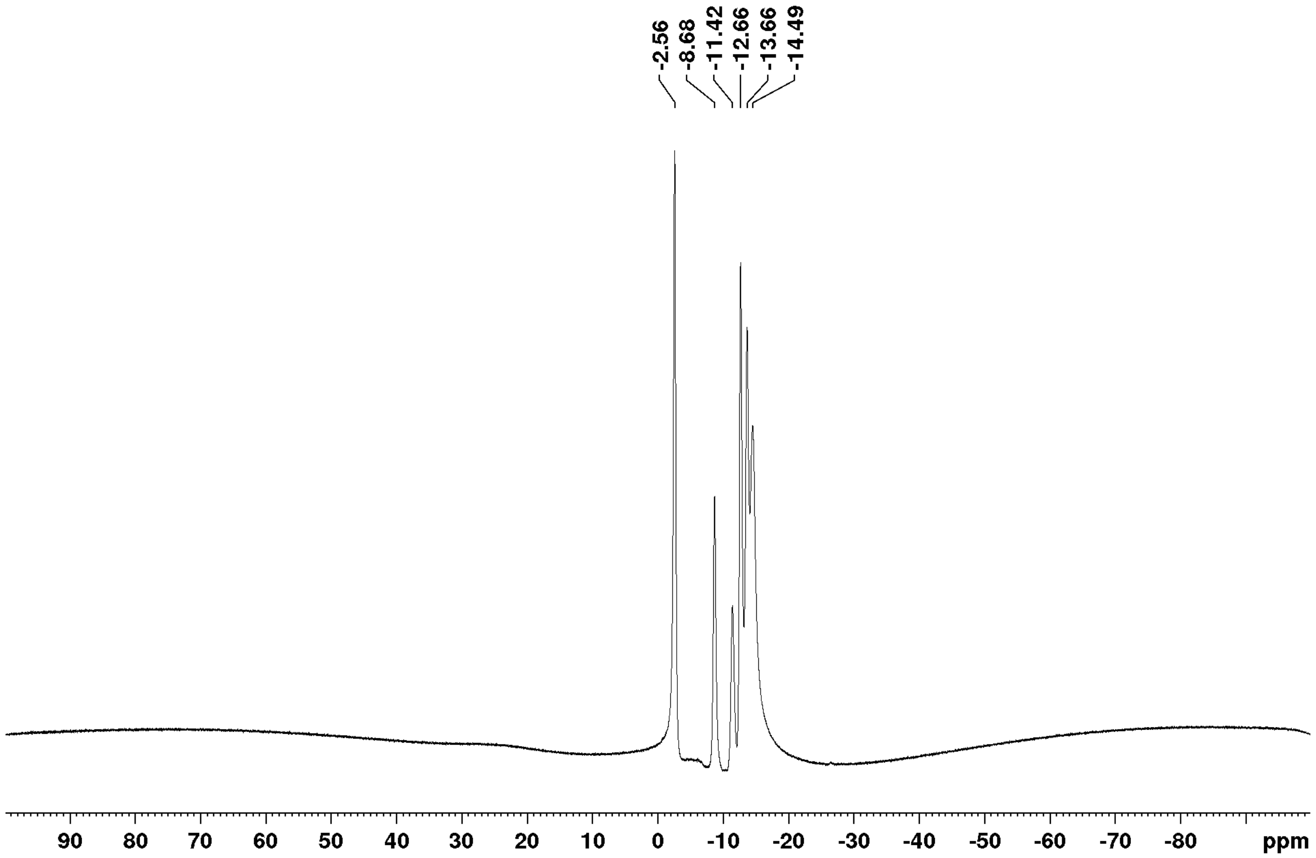

1-((*tert*-butyl)dimethylsilyl)hydroxymethyl)-3-((trimethylsilyl)ethynyl)-1,2-dicarba-*closo*-dodecaborane (S1)
^{1}H NMR (500 MHz; CDCl$_3$)

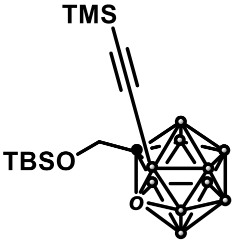

S1

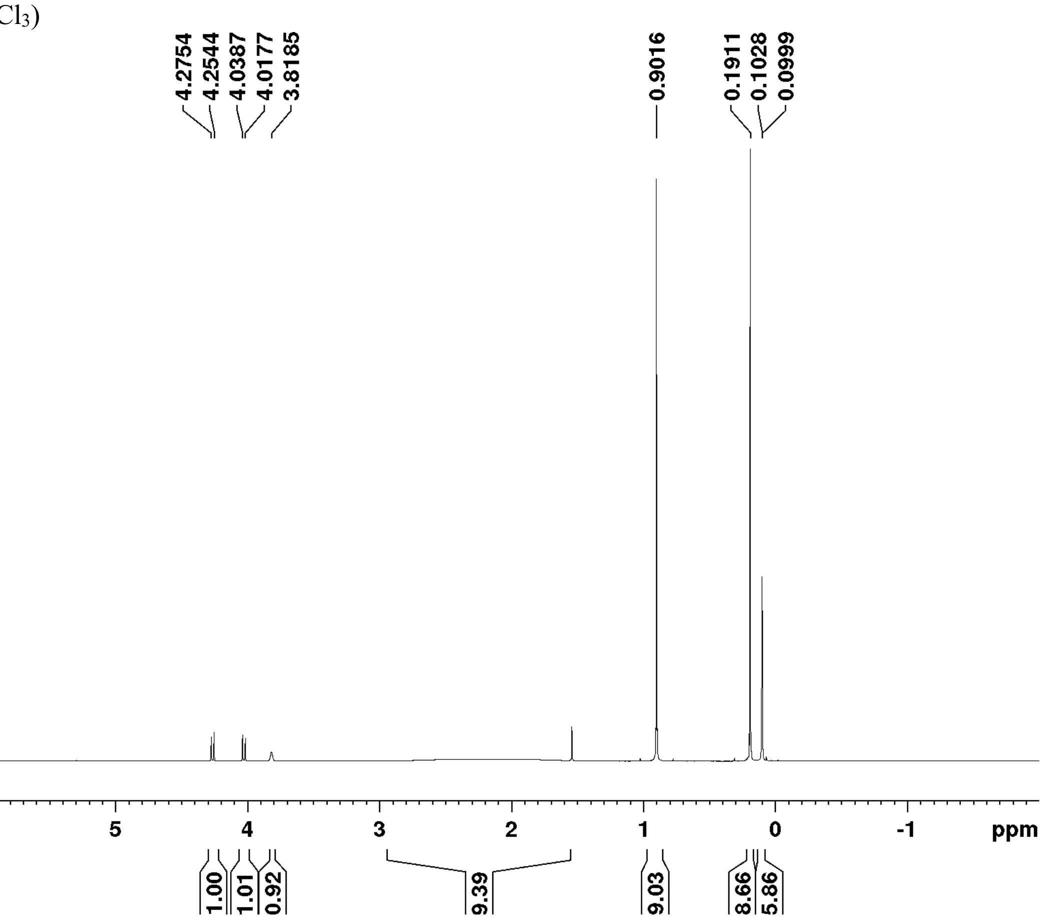

^{13}C NMR (125 MHz; $CDCl_3$)

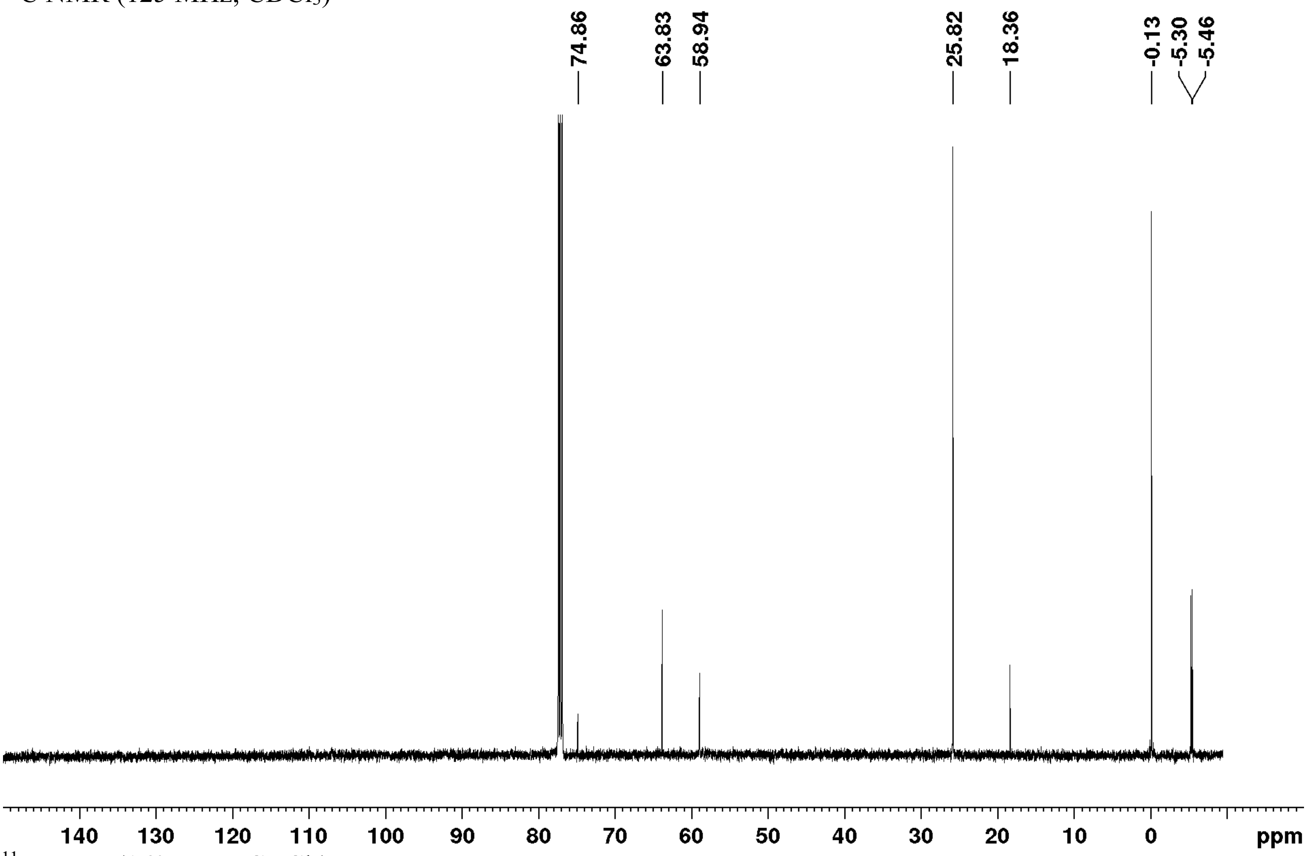

^{11}B NMR (160 MHz; $CDCl_3$)

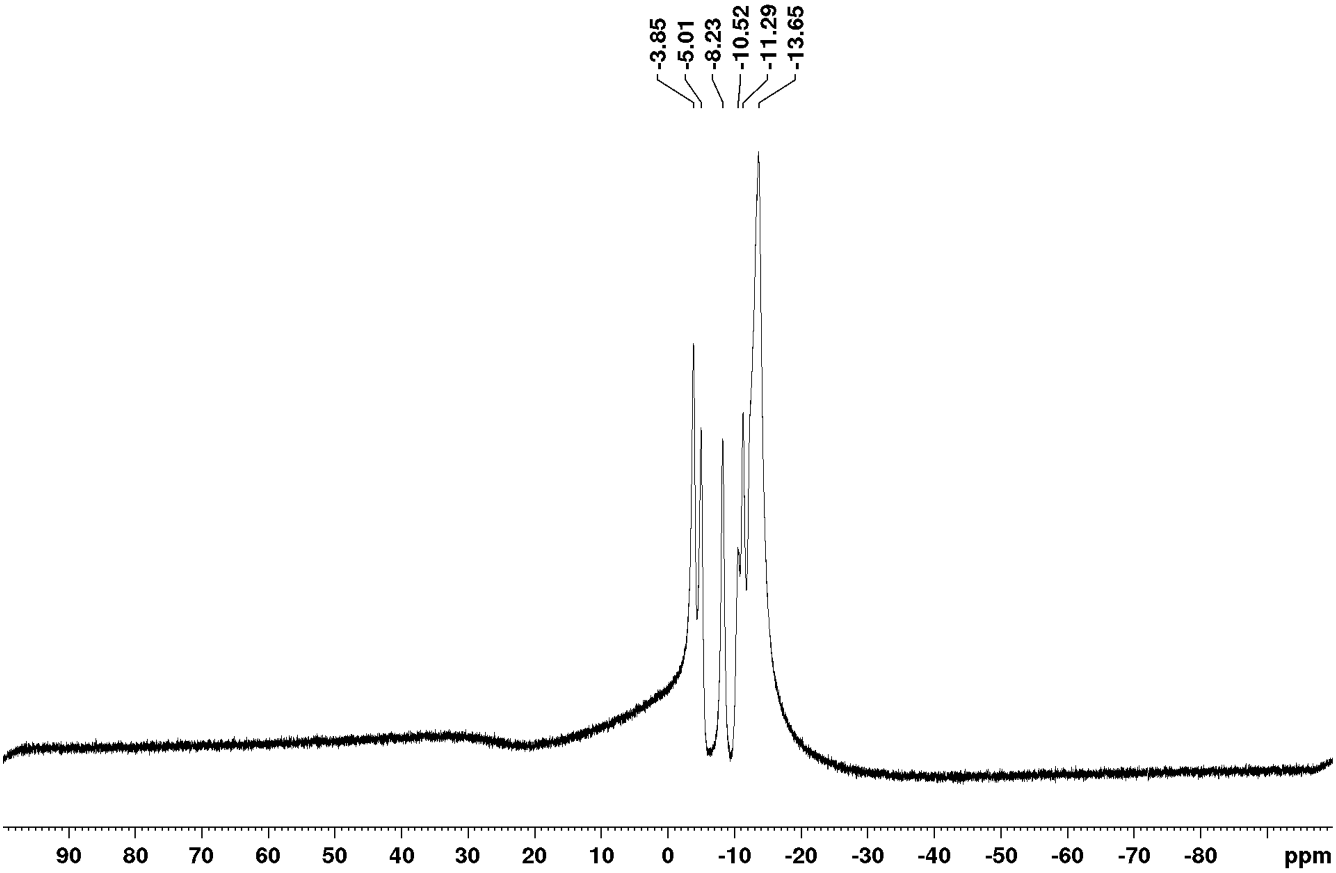

1-((*tert*-butyl)dimethylsilyl)hydroxymethyl)-2-hydroxymethyl-3-((trimethylsilyl)ethynyl)-1,2-dicarba-*closo*-dodecaborane (2)

^{1}H NMR (500 MHz; CDCl$_3$)

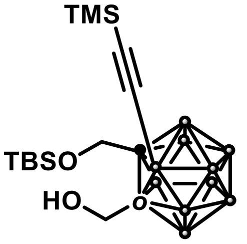

2

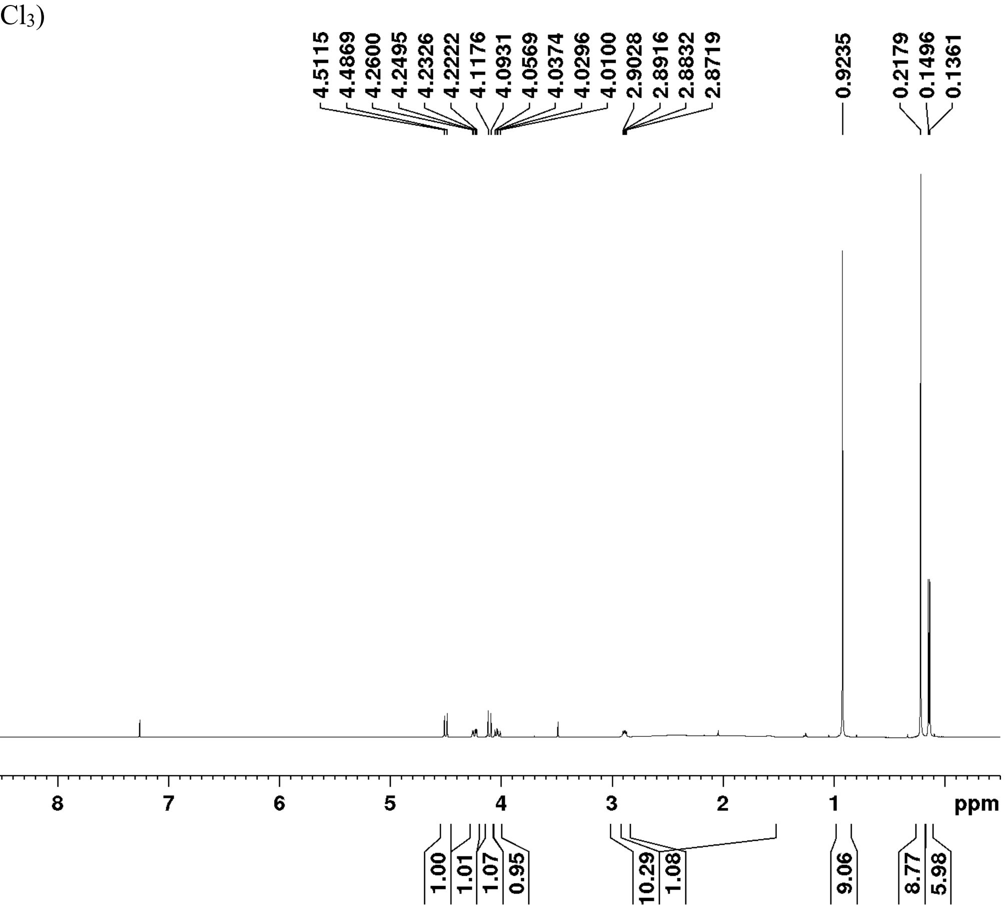

^{13}C NMR (125 MHz; CDCl$_3$)

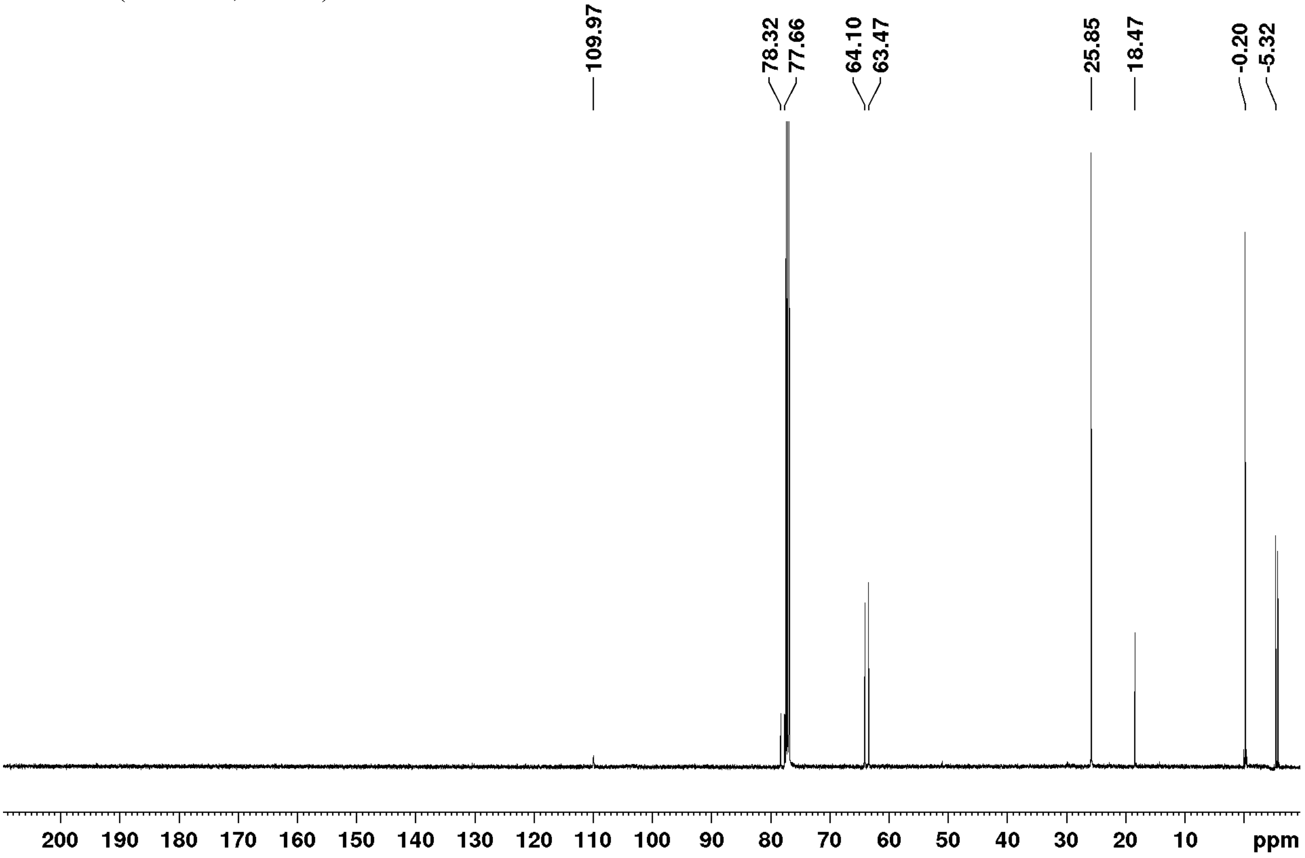

^{11}B NMR (160 MHz; $CDCl_3$)

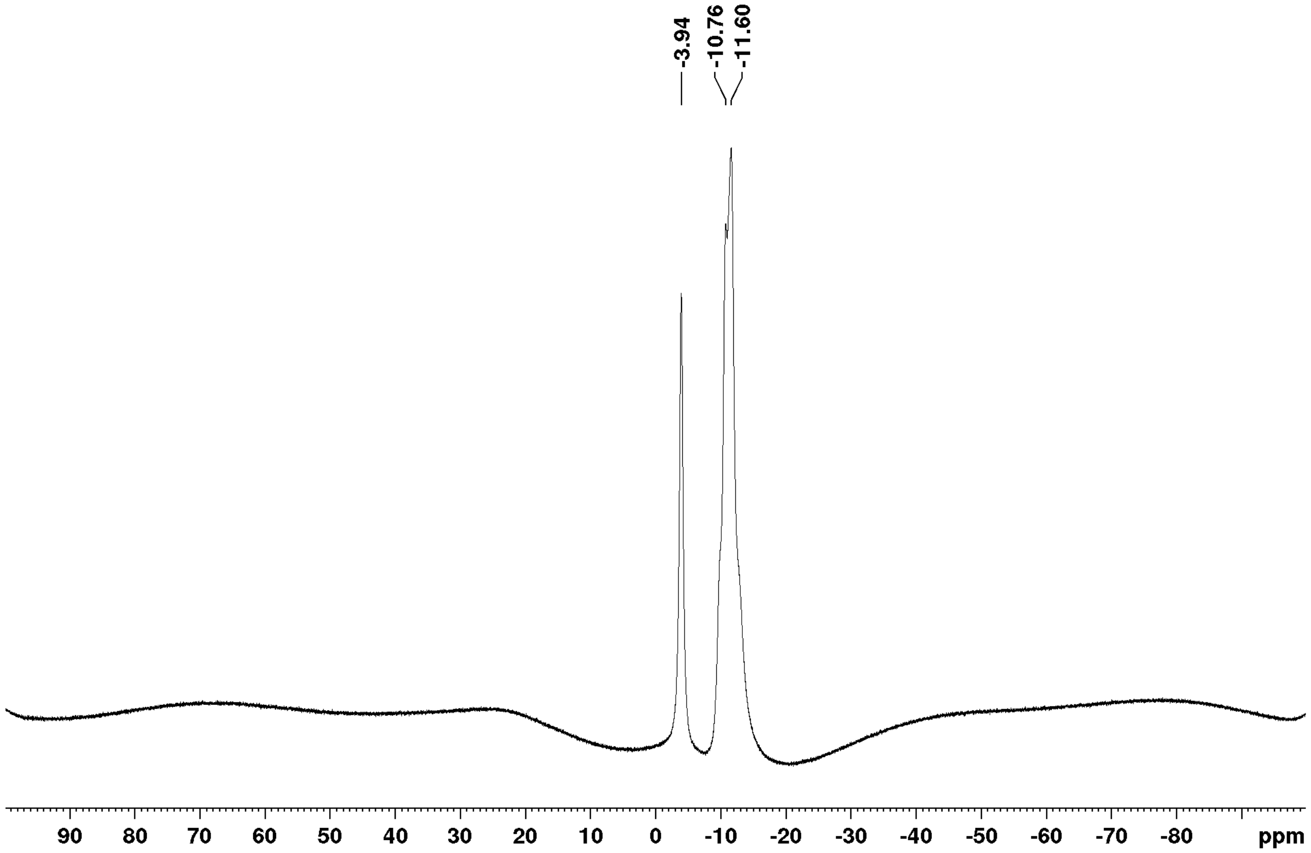

Synthesis of 1-((*tert*-butyl)dimethylsilyl)hydroxymethyl)-2-hydroxymethyl-3-ethynyl-1,2-dicarba-*closo*-dodecaborane (3)

^1H NMR (400 MHz; $CDCl_3$)

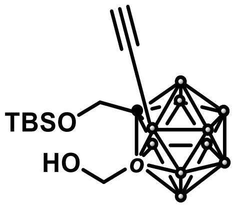

3

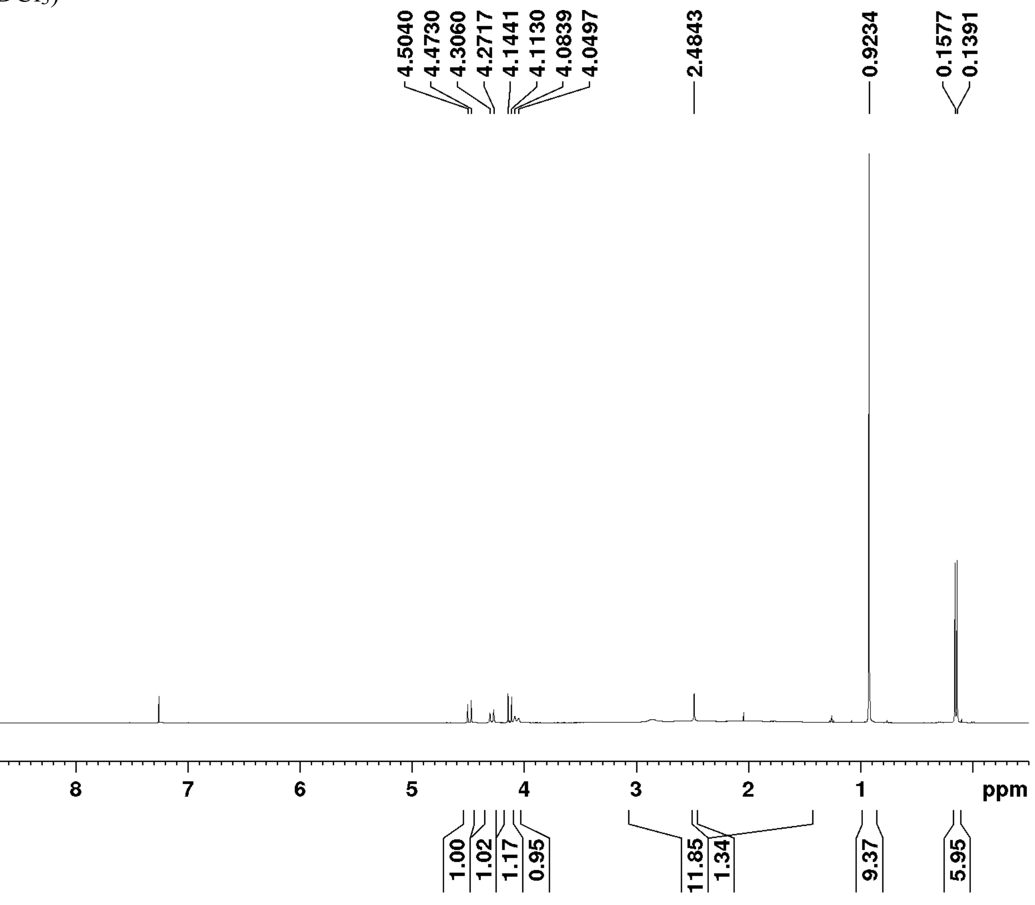

^{13}C NMR (100 MHz; $CDCl_3$)

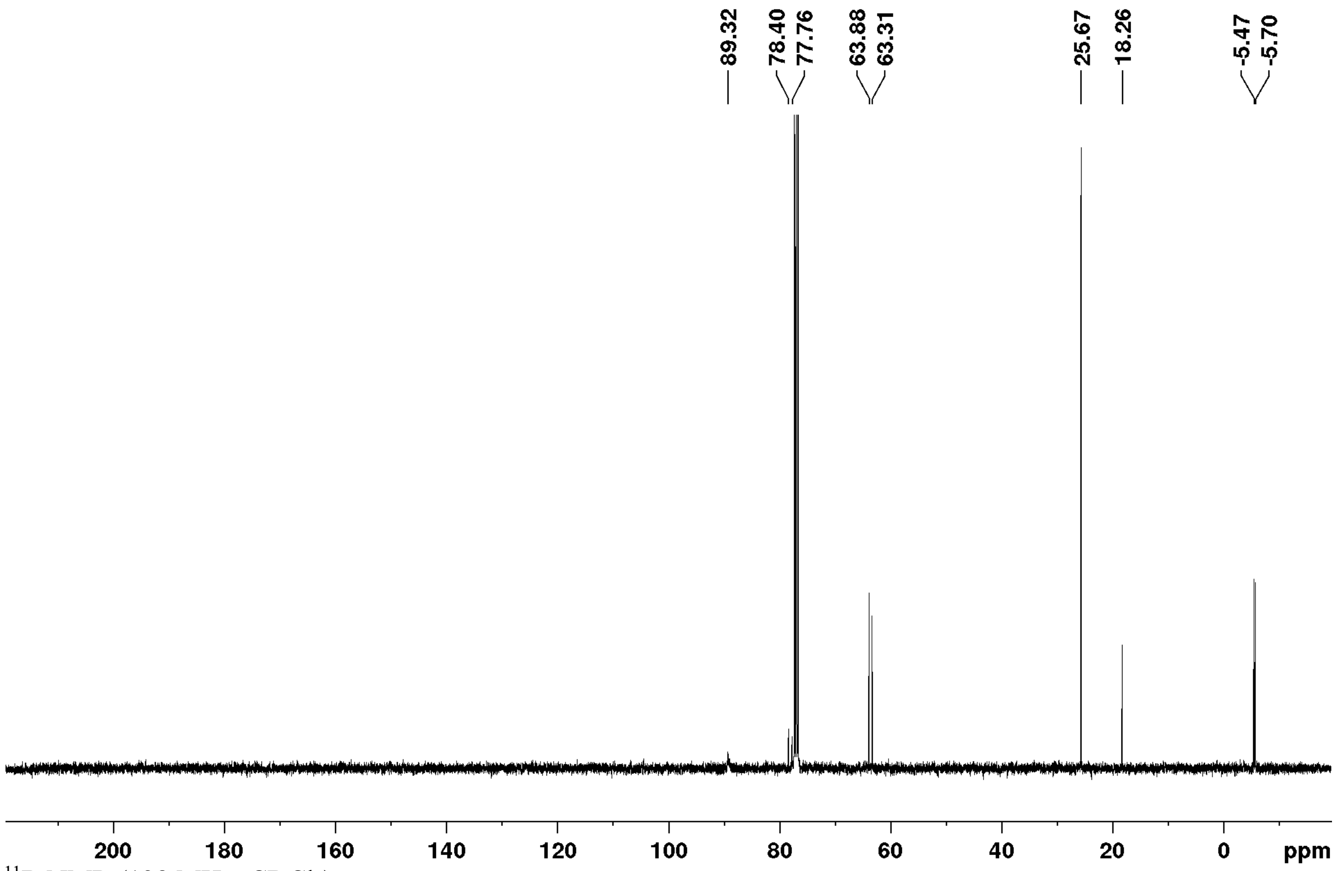

^{11}B NMR (128 MHz; $CDCl_3$)

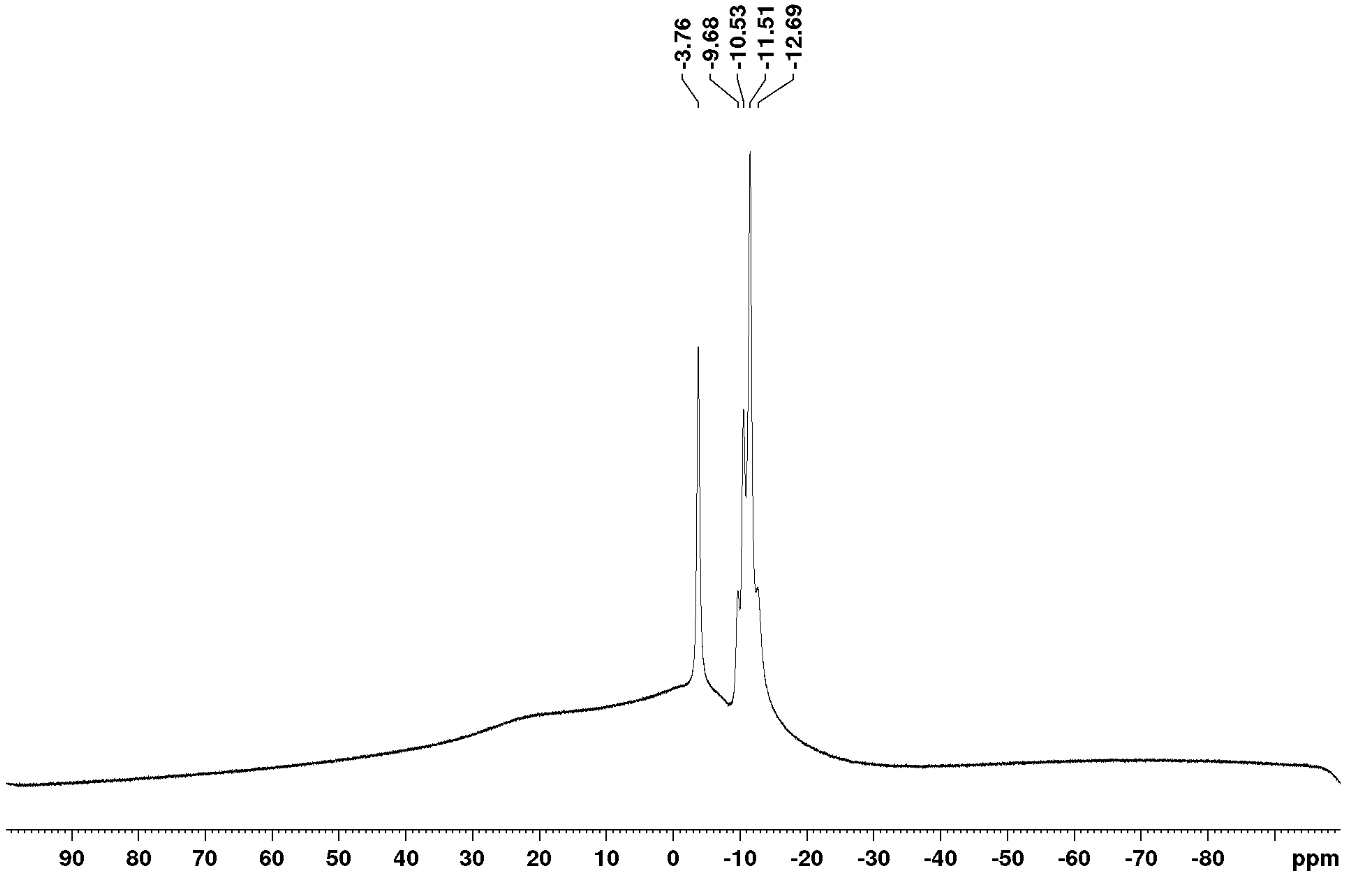

1,2-bis(hydroxymethyl)-3-ethynyl-1,2-dicarba-*closo*-dodecaborane (4)

^{1}H NMR (500 MHz; $CDCl_3$)

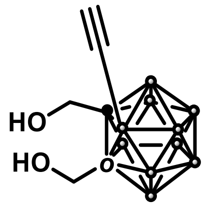

4

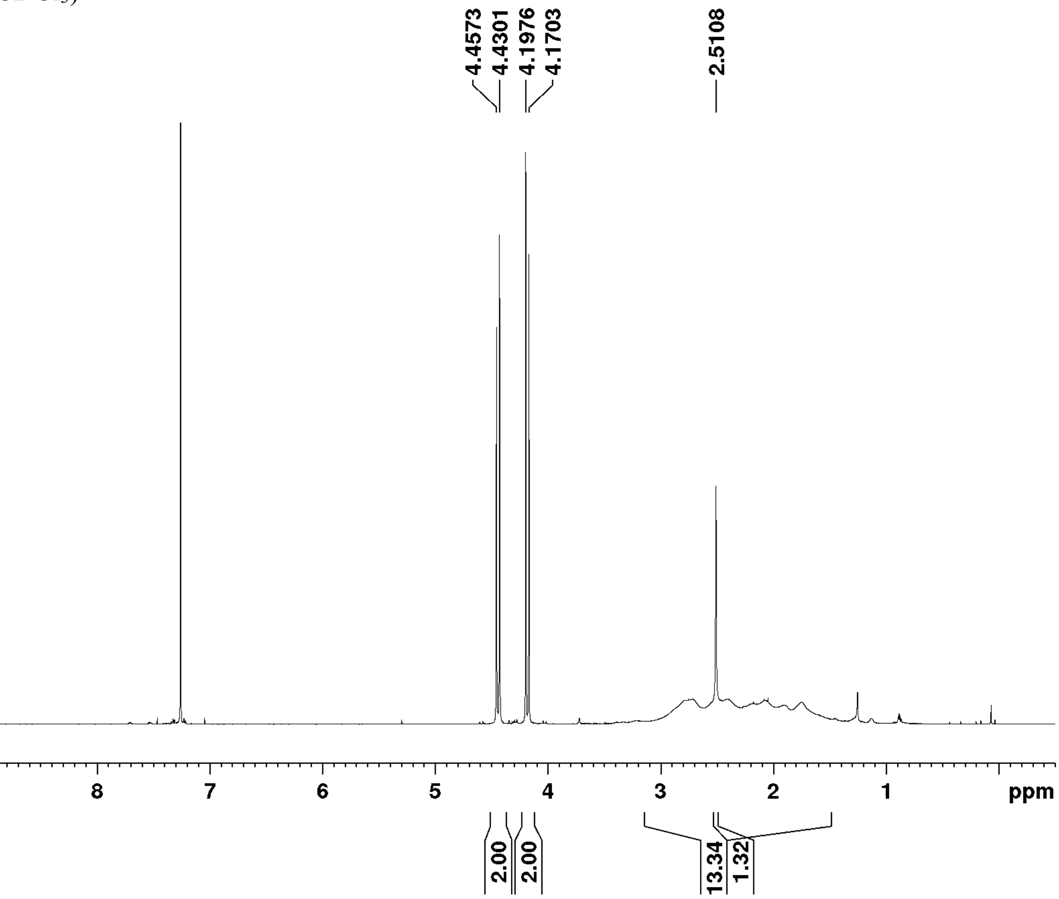

^{13}C NMR (125 MHz; $CDCl_3$)

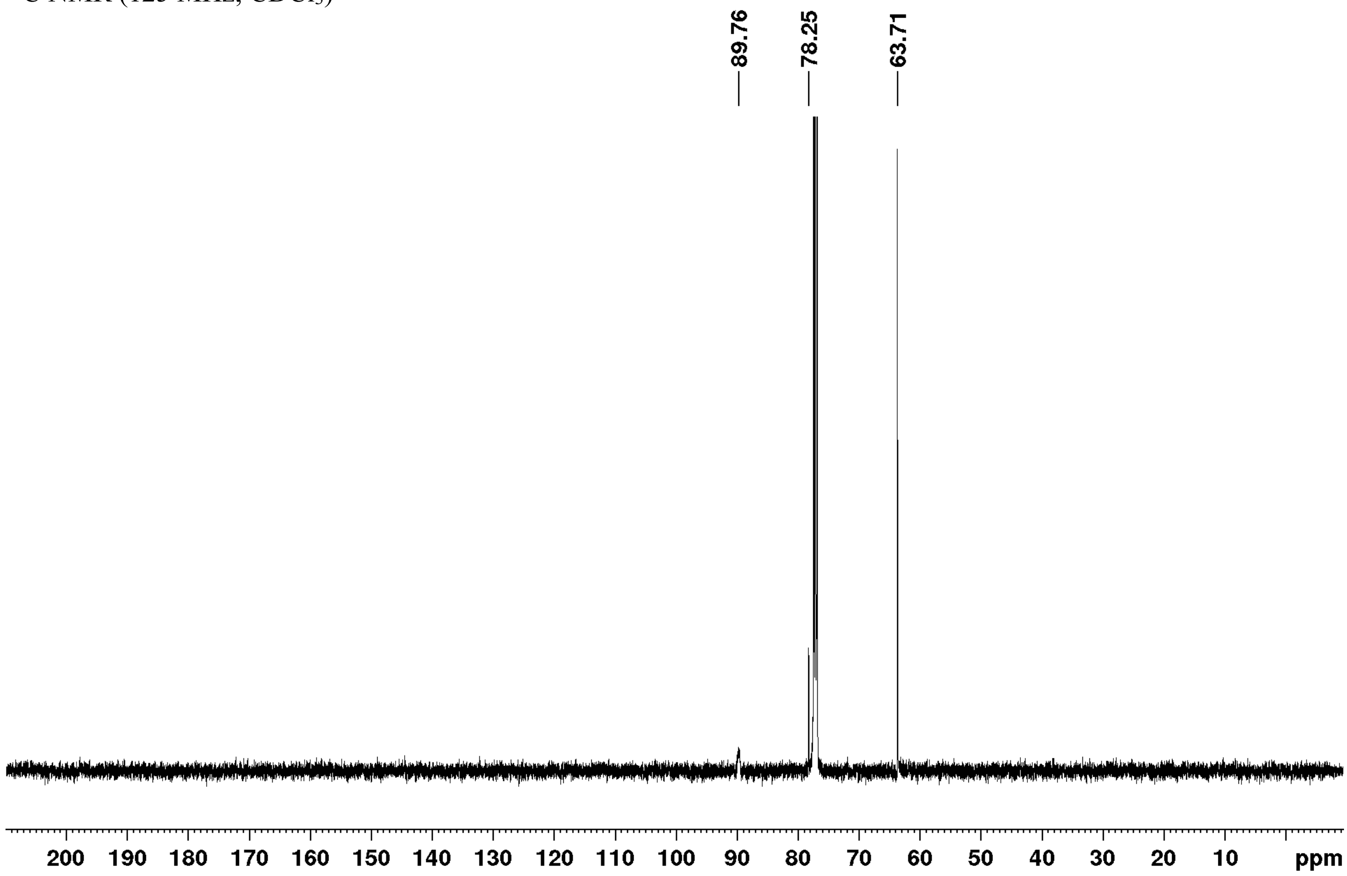

^{11}B NMR (160 MHz; CDCl$_3$)

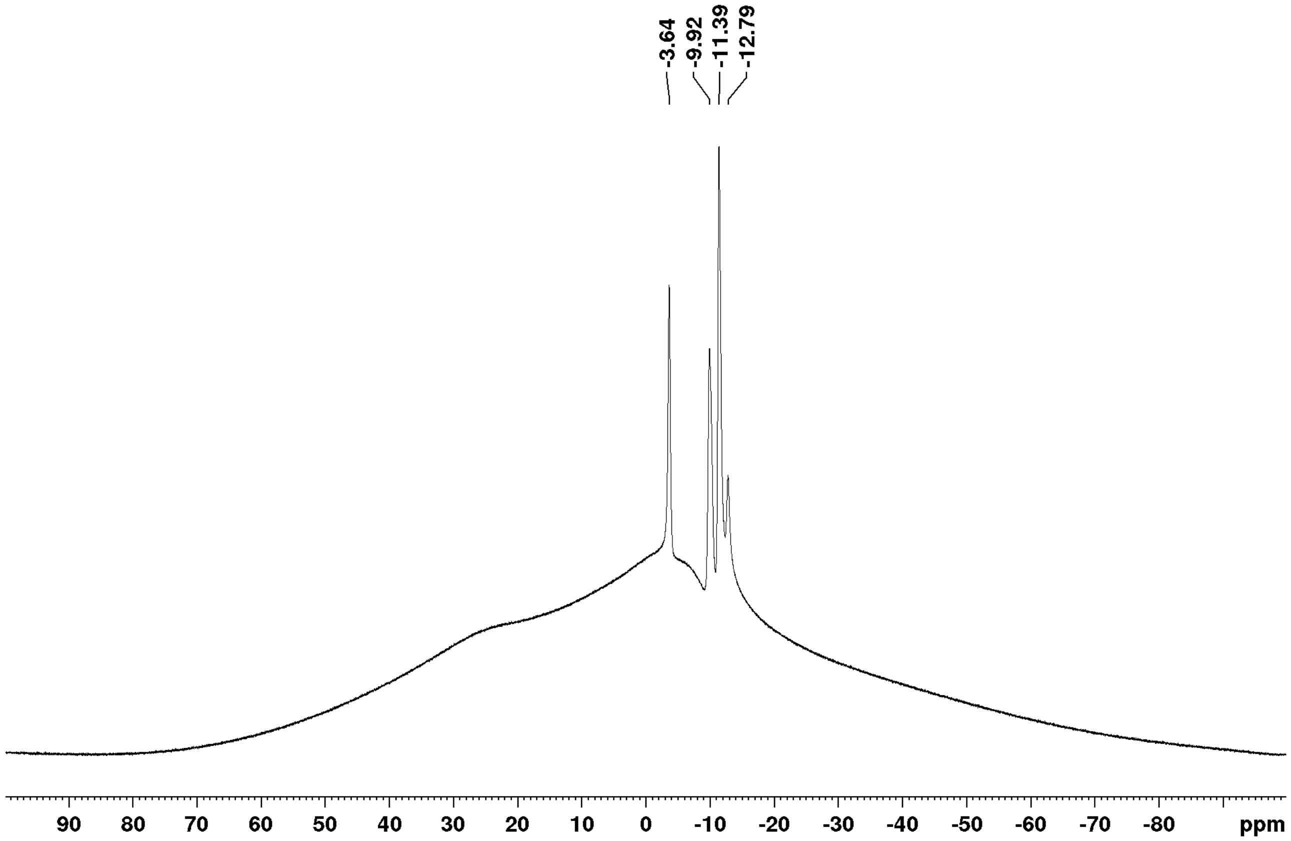

1-((*tert*-butyl)dimethylsilyl)hydroxymethyl)-2-phenylacetoxymethyl-3-ethynyl-1,2-dicarba-*closo*-dodecaborane (5)

^{1}H NMR (400 MHz; CDCl$_3$)

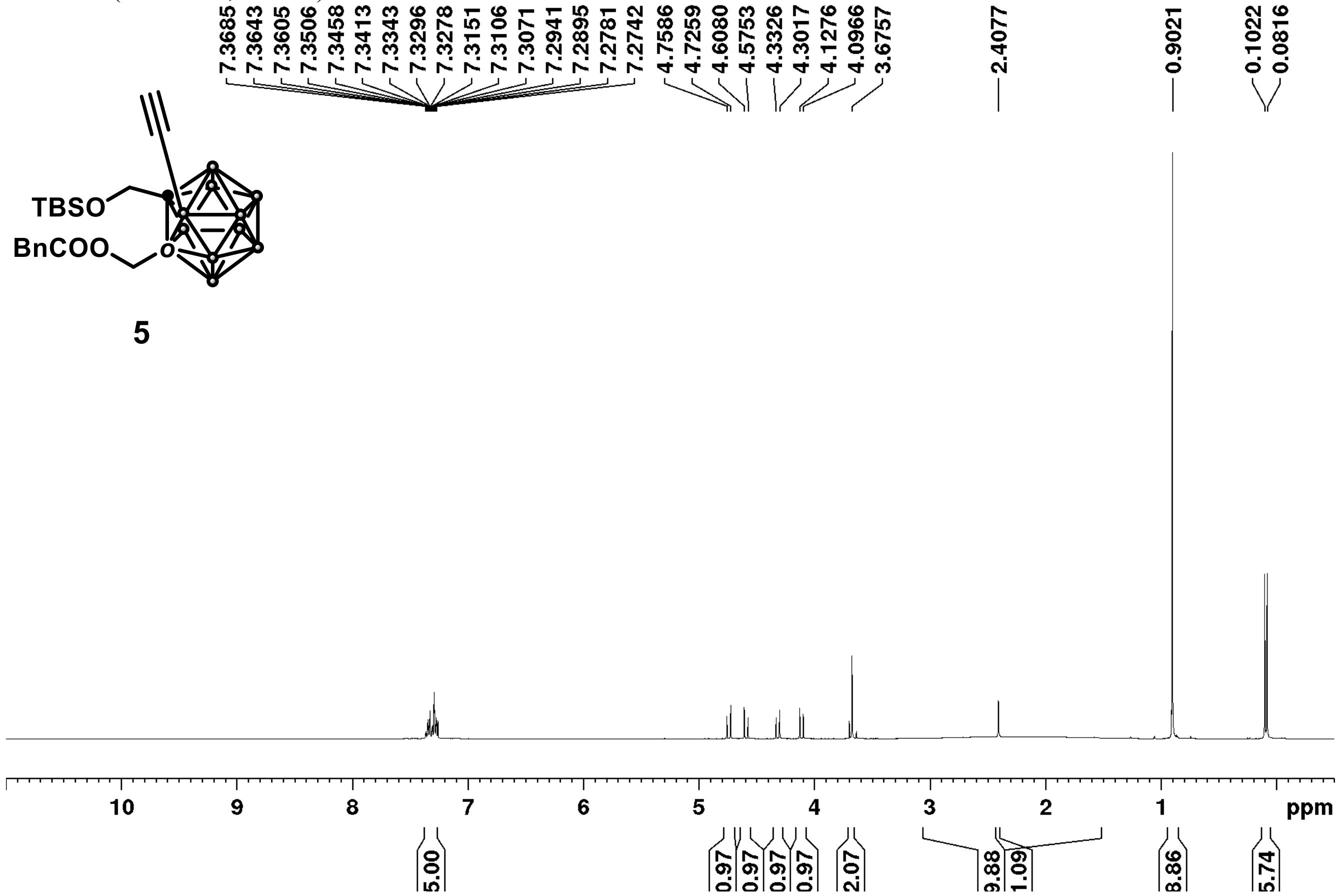

^{13}C NMR (100 MHz; $CDCl_3$)

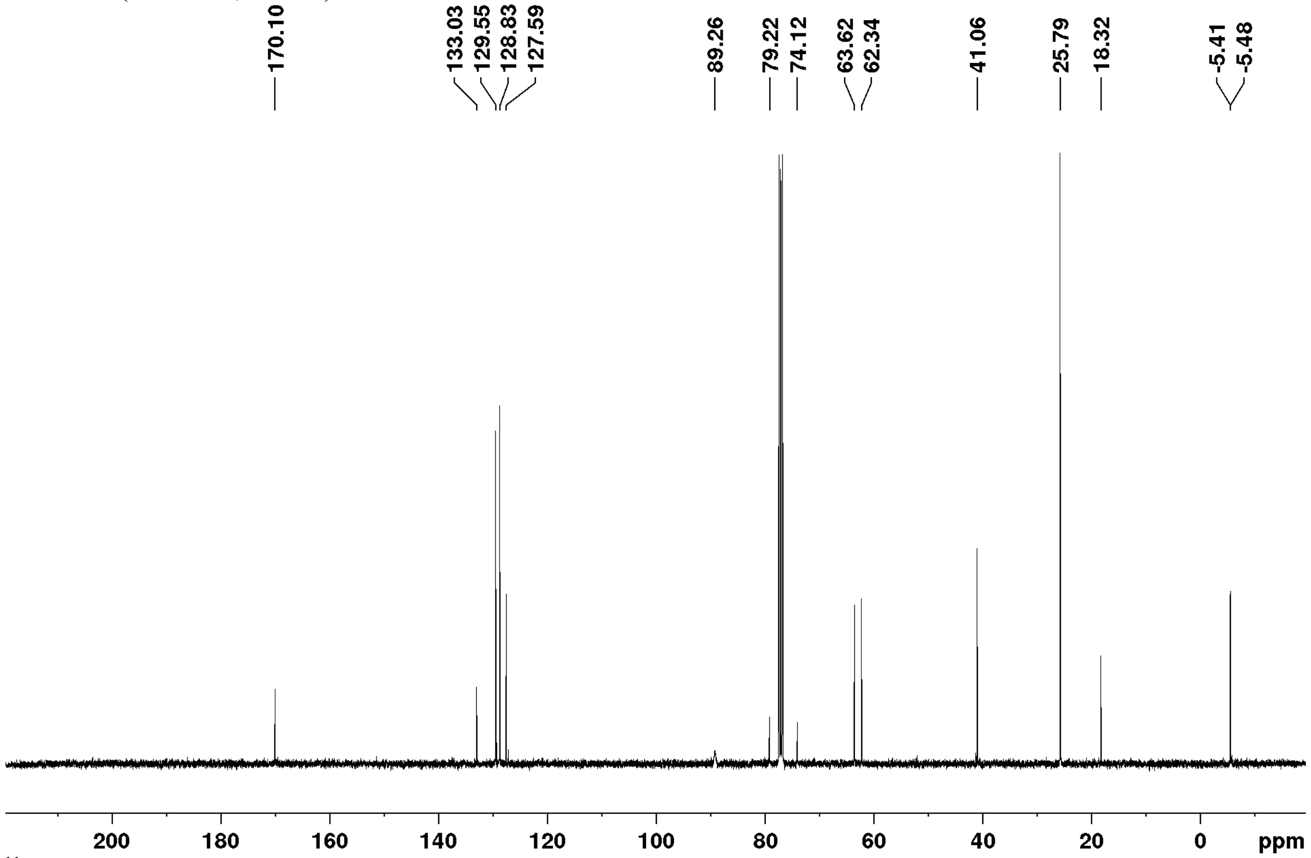

^{11}B NMR (128 MHz; $CDCl_3$)

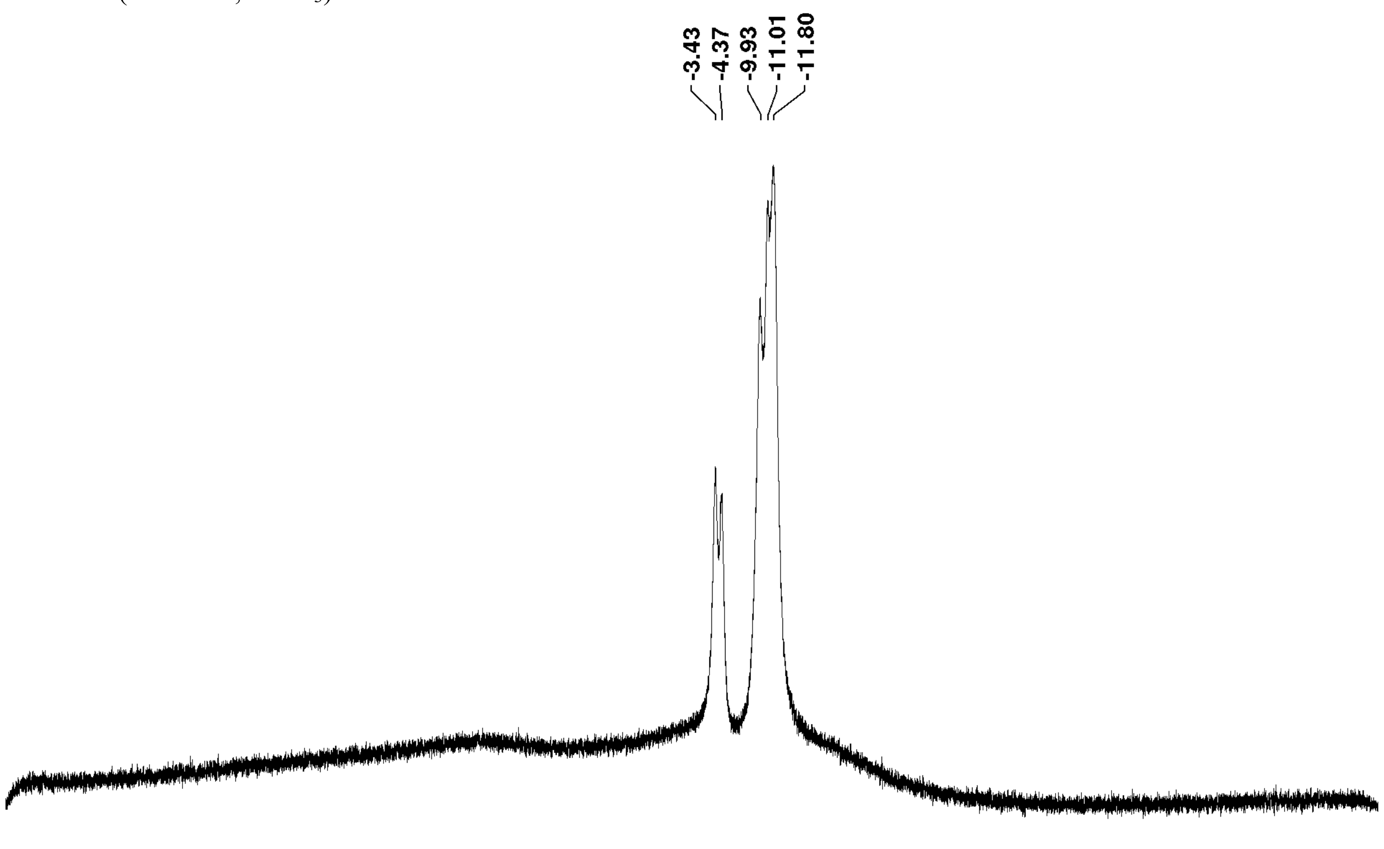

1-hydroxymethyl-2-phenylacetoxymethyl-3-ethynyl-1,2-dicarba-*closo*-dodecaborane (S2)

^{1}H NMR (400 MHz; $CDCl_3$)

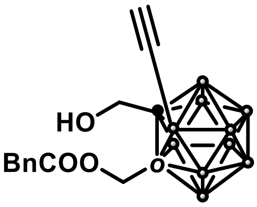

S2

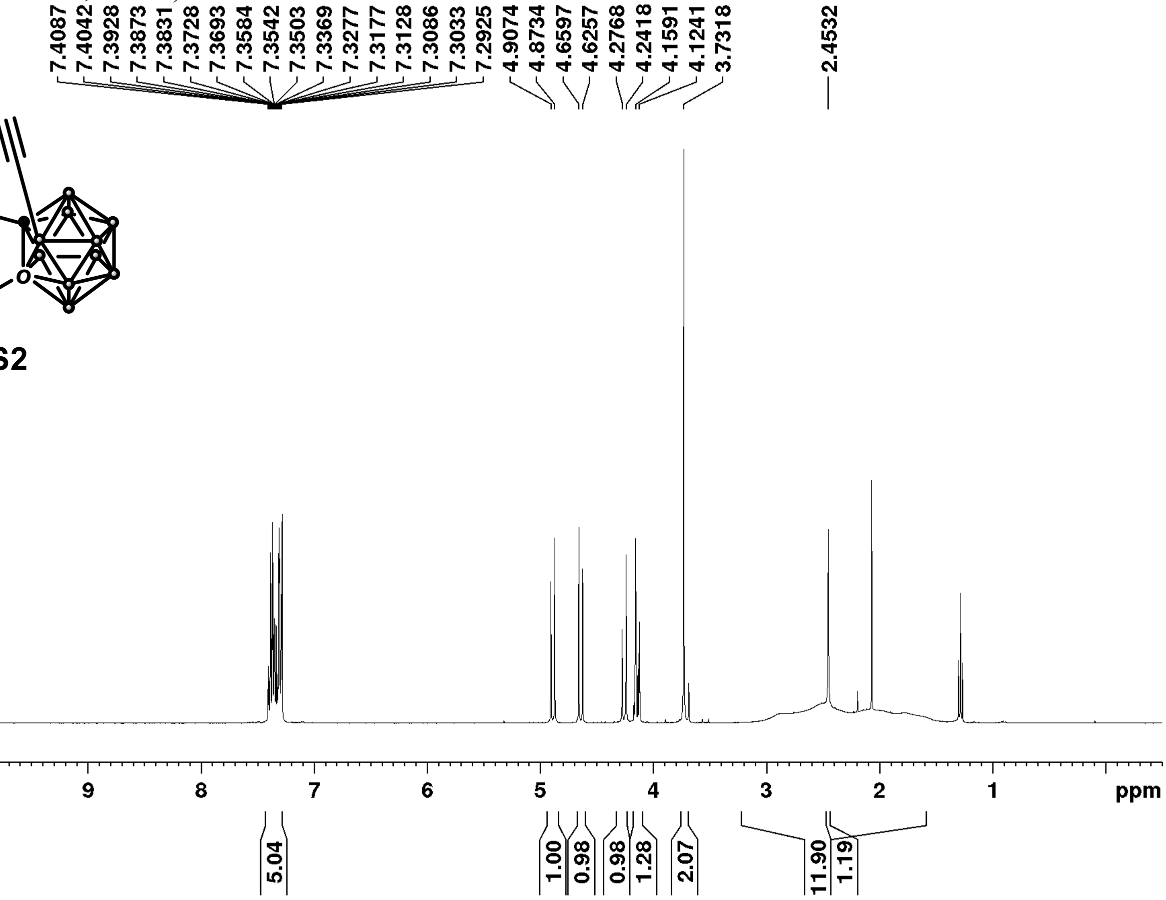

^{13}C NMR (100 MHz; $CDCl_3$)

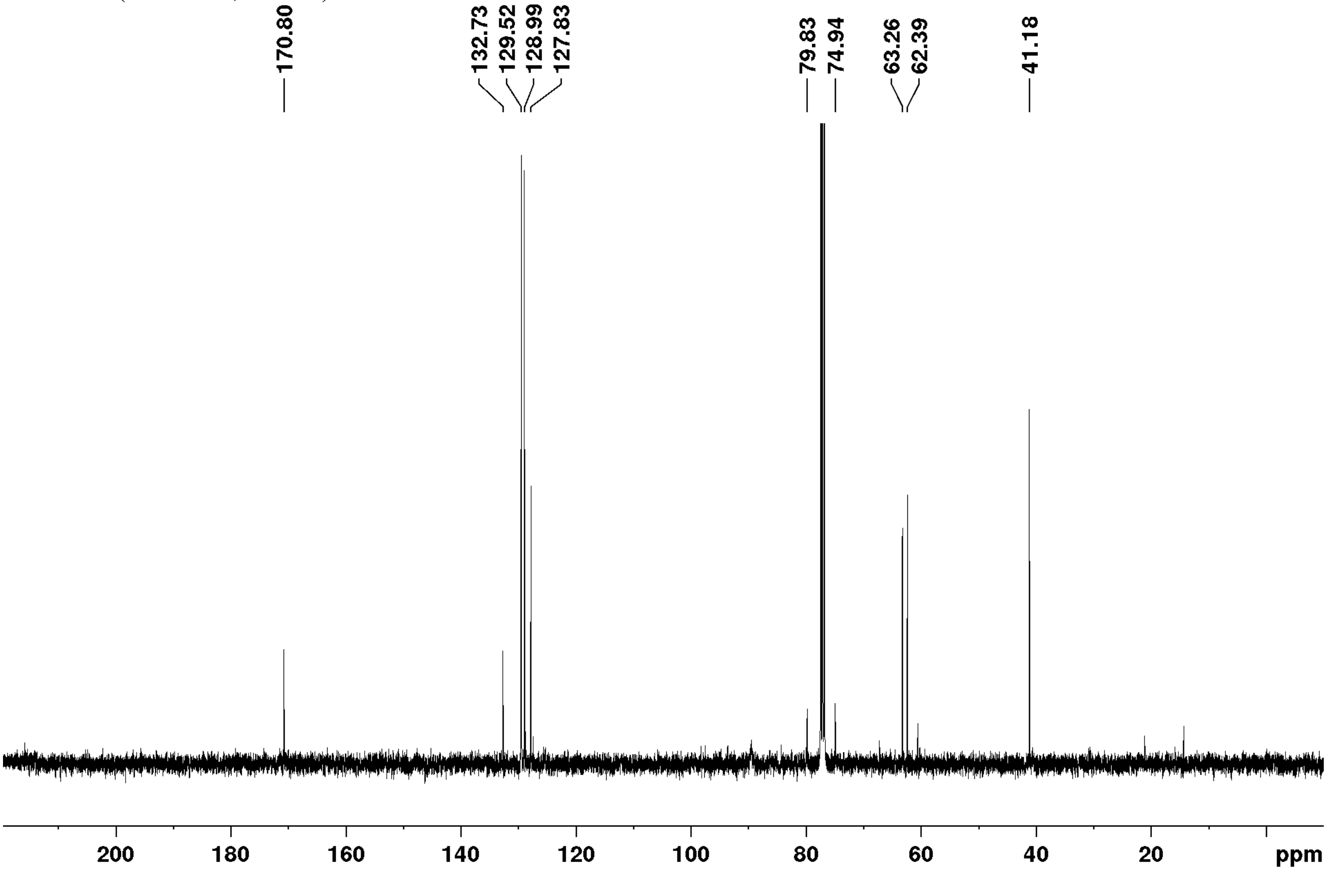

^{11}B NMR (128 MHz; $CDCl_3$)

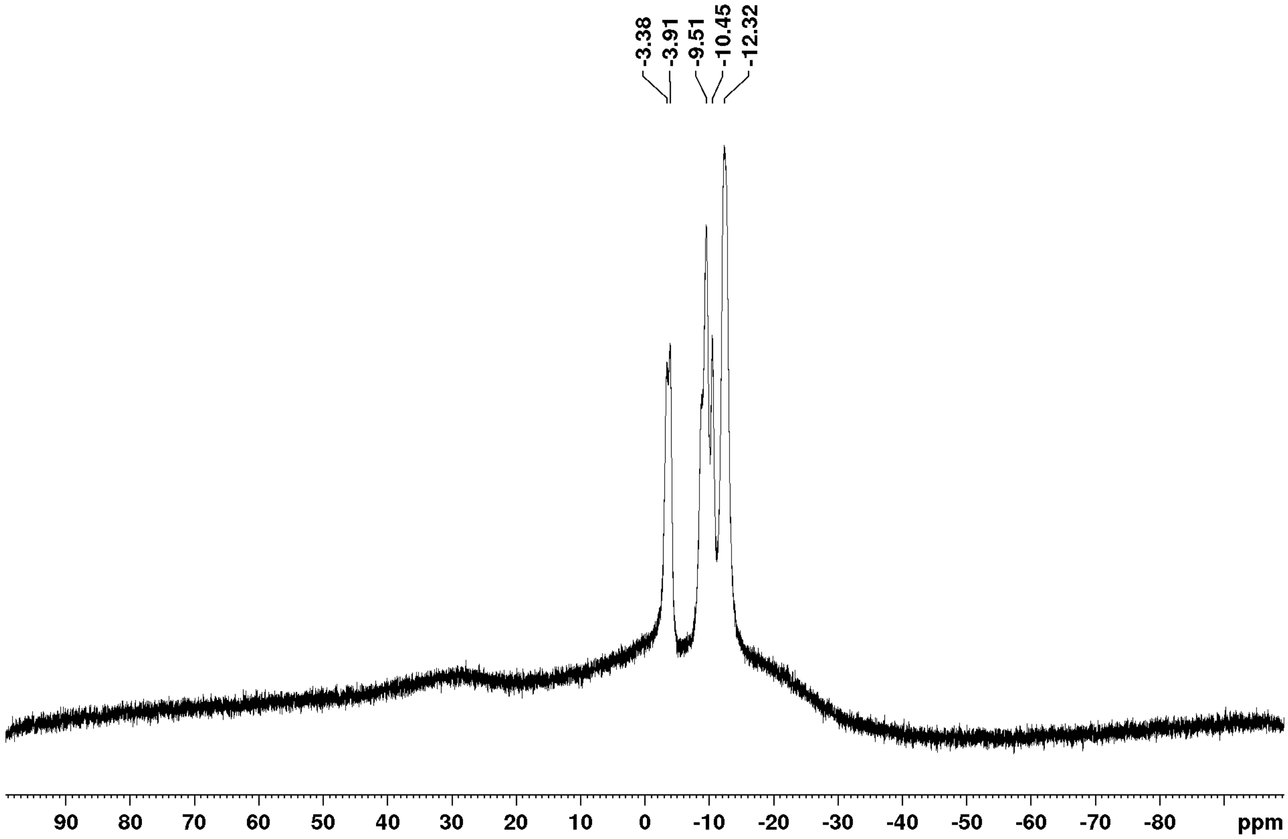

1-(3-methylbutanoyl)oxymethyl-2-phenylacetoxymethyl-3-ethynyl-1,2-dicarba-*closo*-dodecaborane (6)

^{1}H NMR (500 MHz; $CDCl_3$)

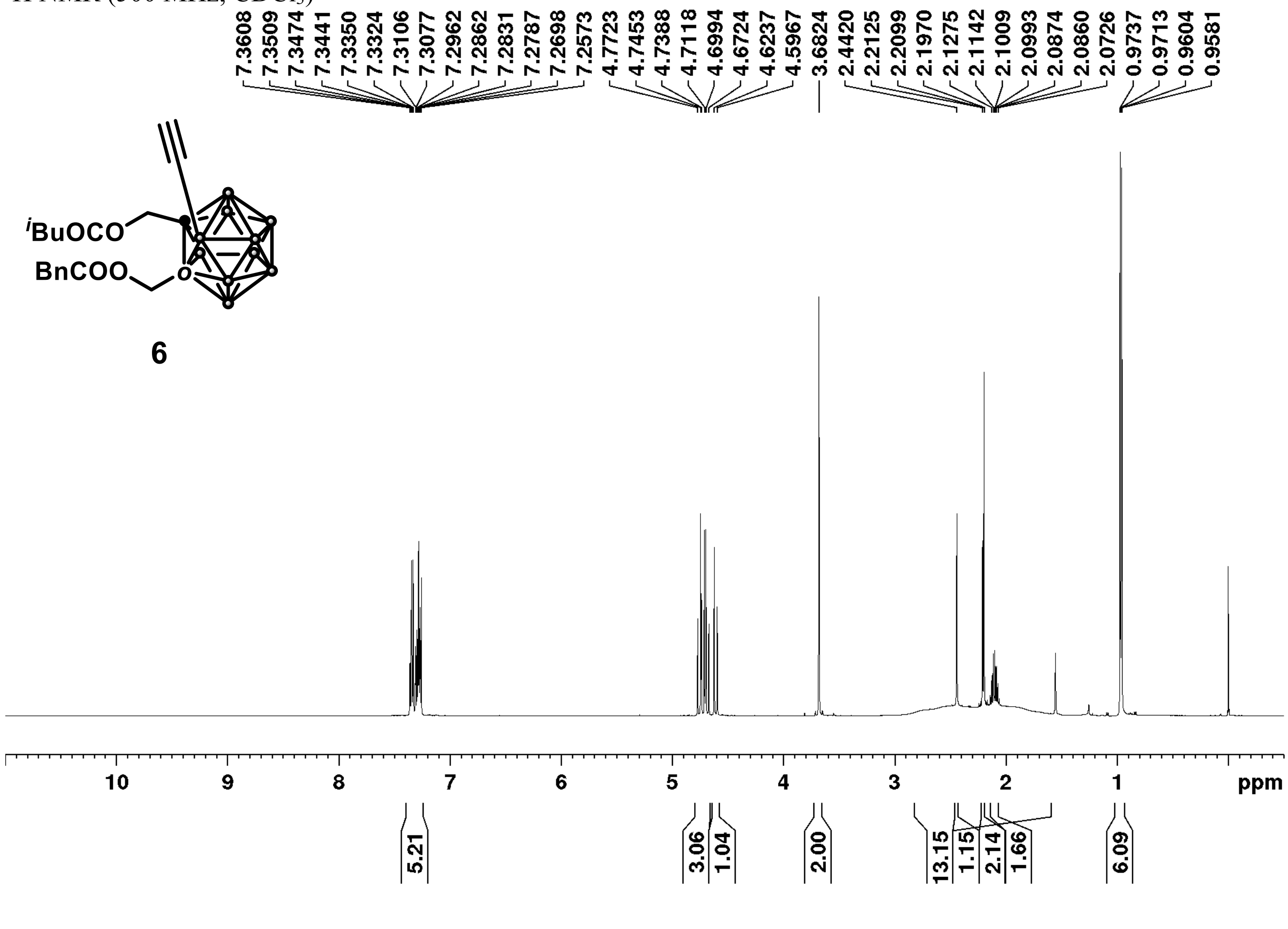

^{13}C NMR (125 MHz; $CDCl_3$)

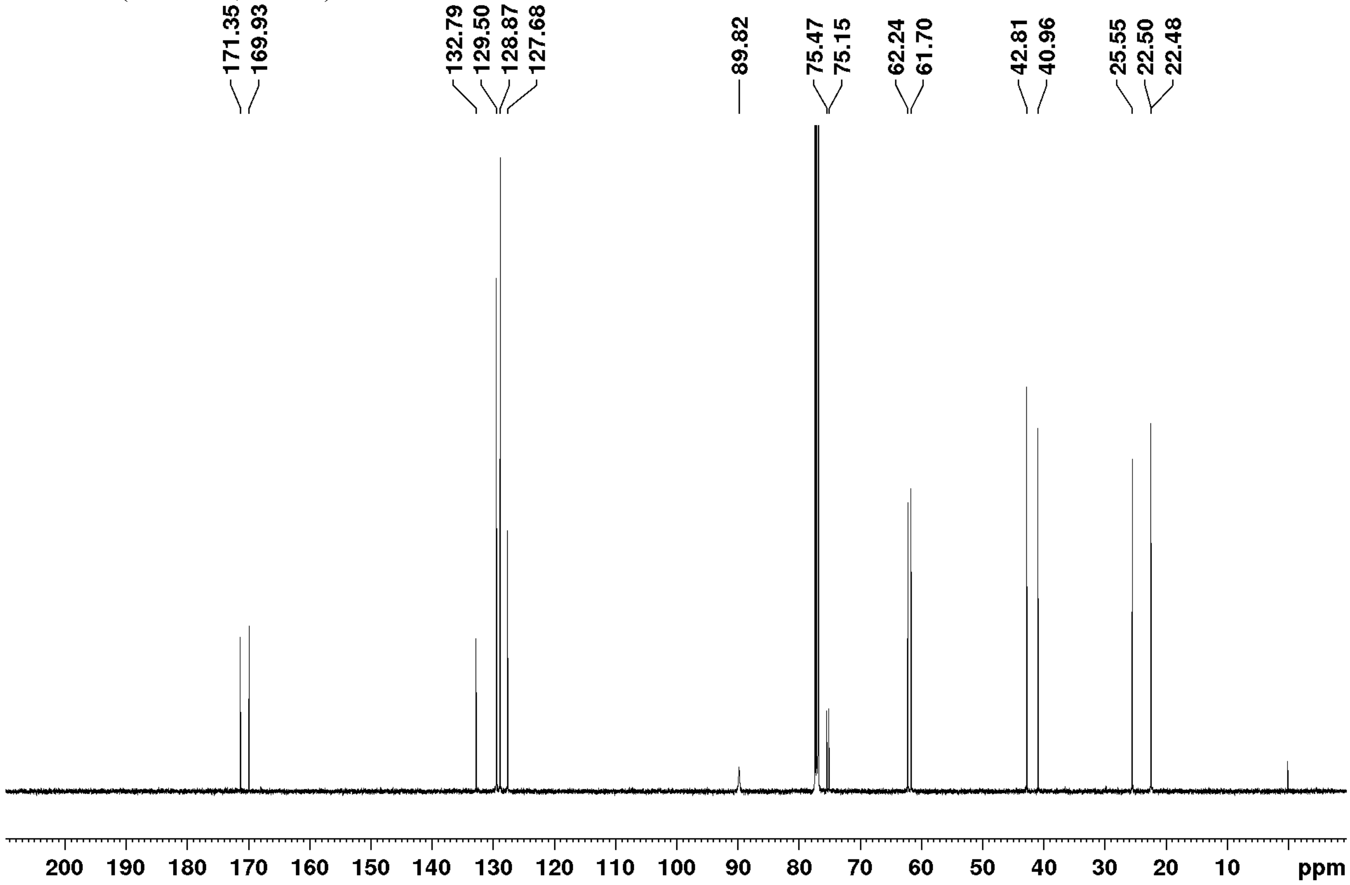

^{11}B NMR (160 MHz; $CDCl_3$)

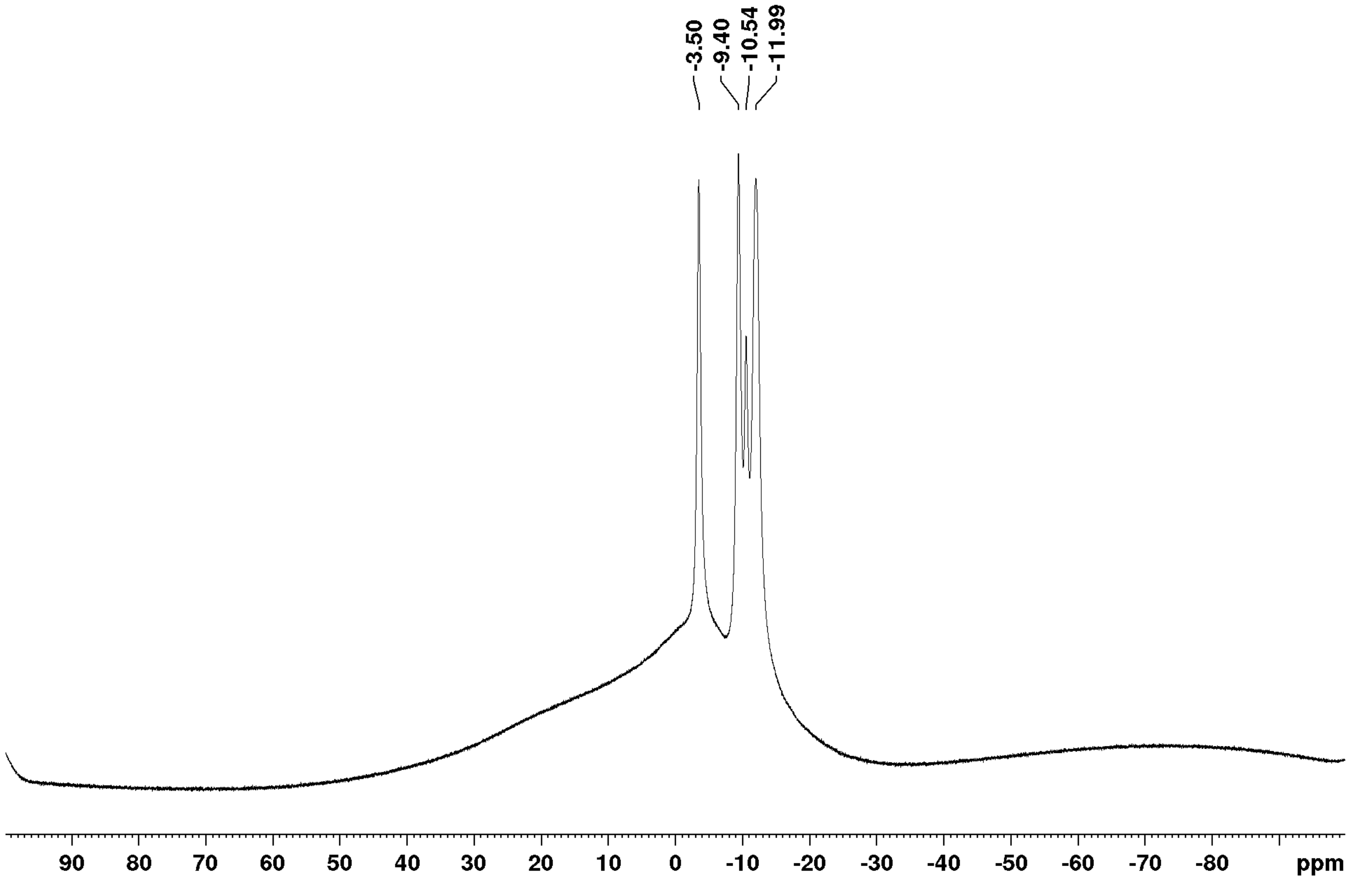

1,2-bis(phenylacetoxymethyl)-3-ethynyl-1,2-dicarba-*closo*-dodecaborane (7a)

^{1}H NMR (500 MHz; $CDCl_3$)

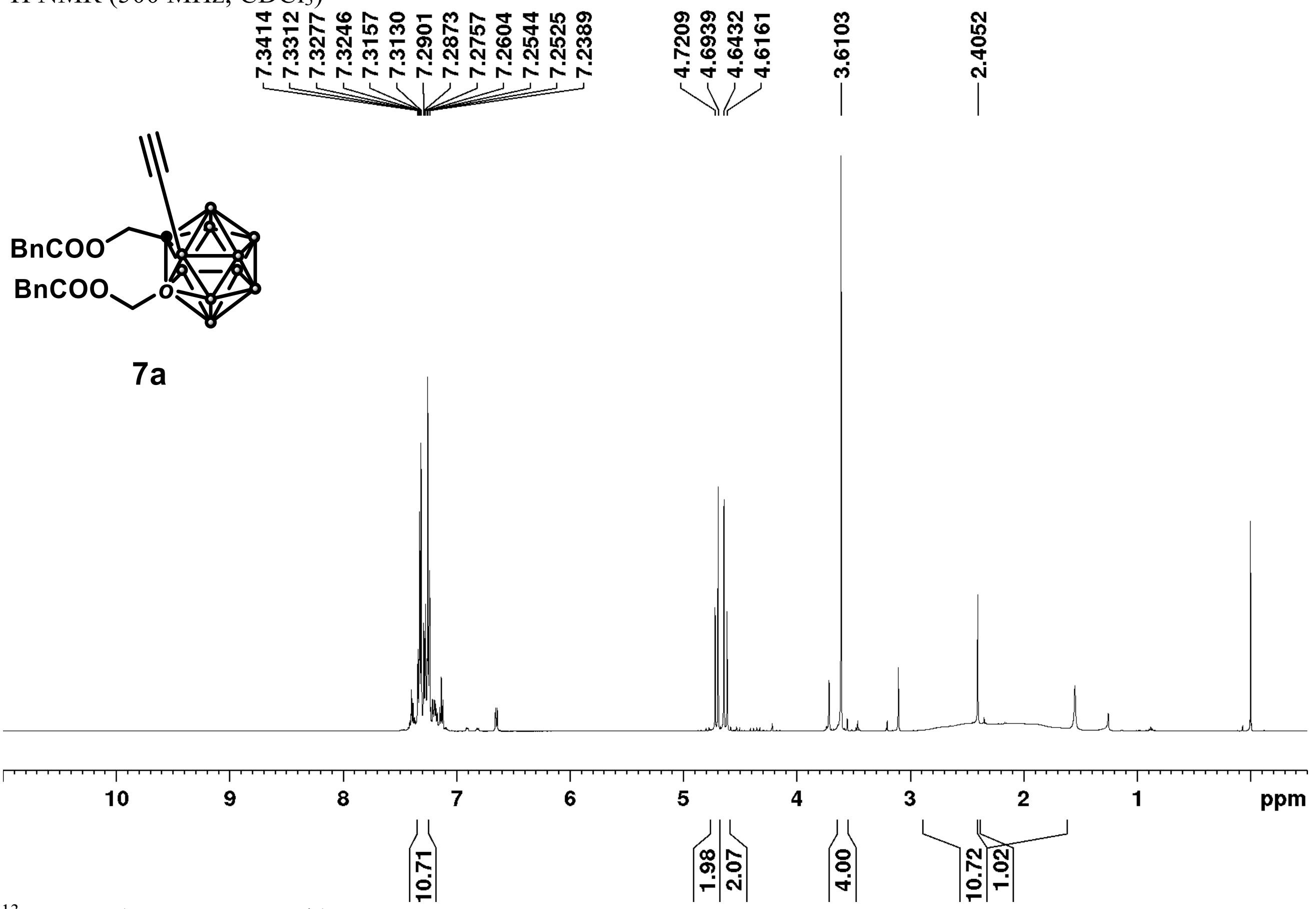

^{13}C NMR (125 MHz; $CDCl_3$)

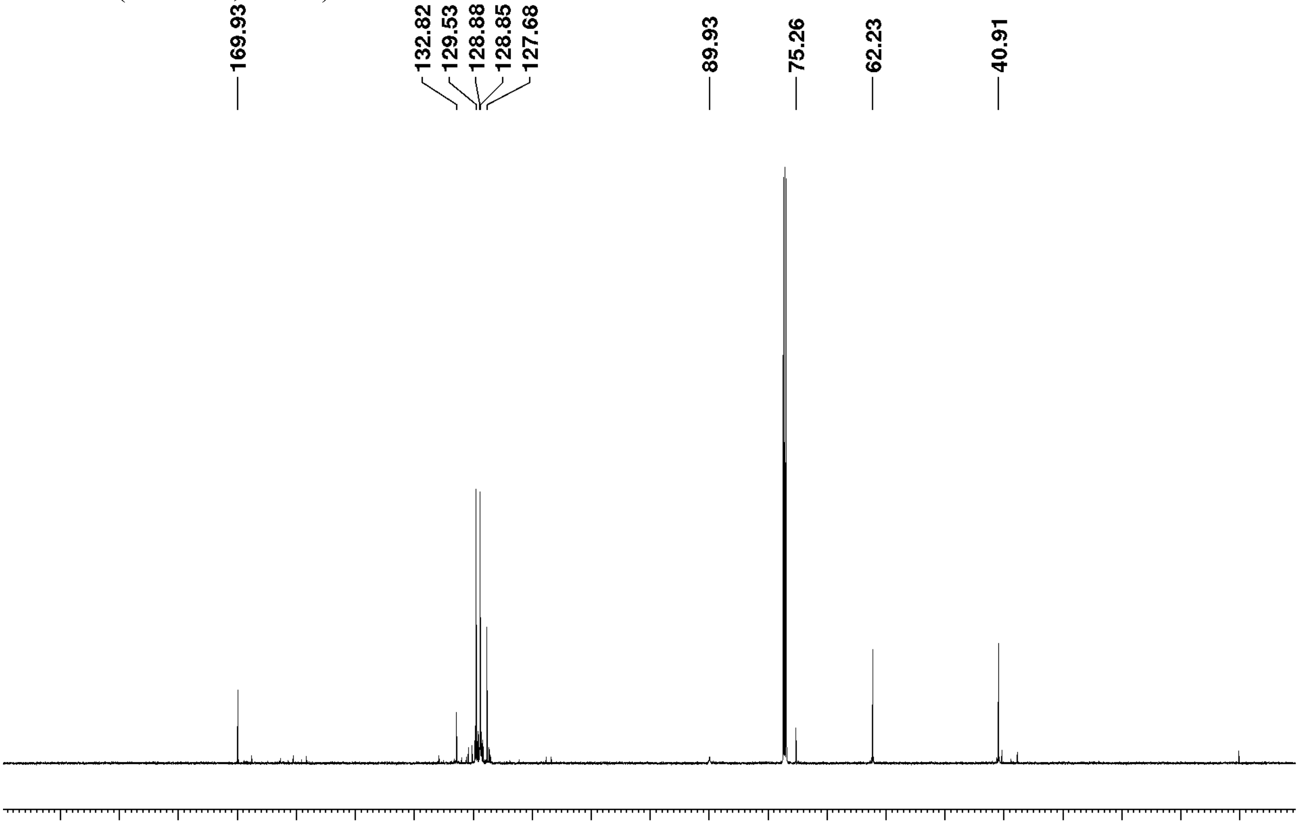

^{11}B NMR (160 MHz; $CDCl_3$)

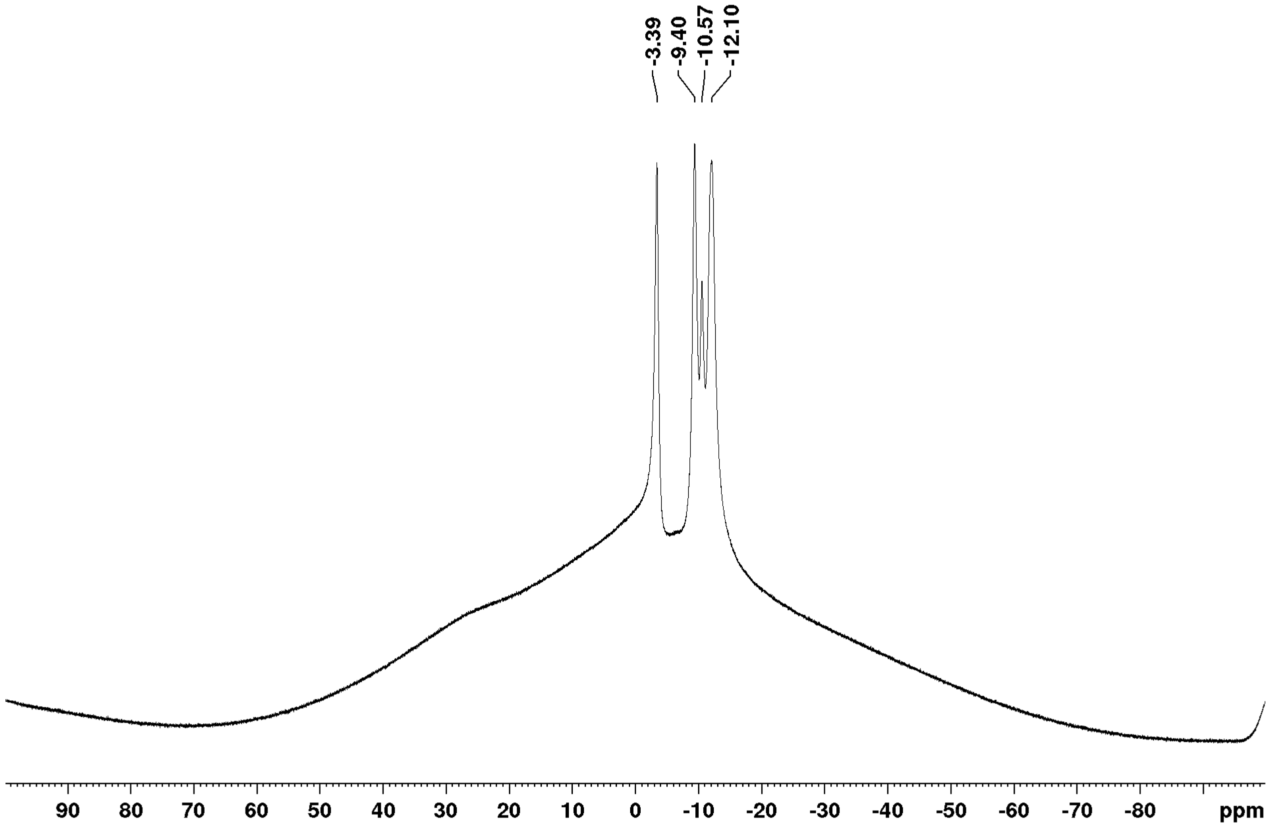

1,2-bis((3-methylbutanoyl)oxymethyl)-3-ethynyl-1,2-dicarba-*closo*-dodecaborane (7b)

^{1}H NMR (500 MHz; $CDCl_3$)

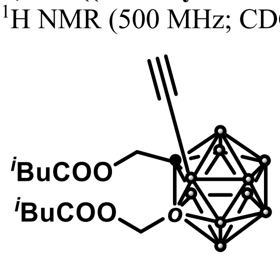

7b

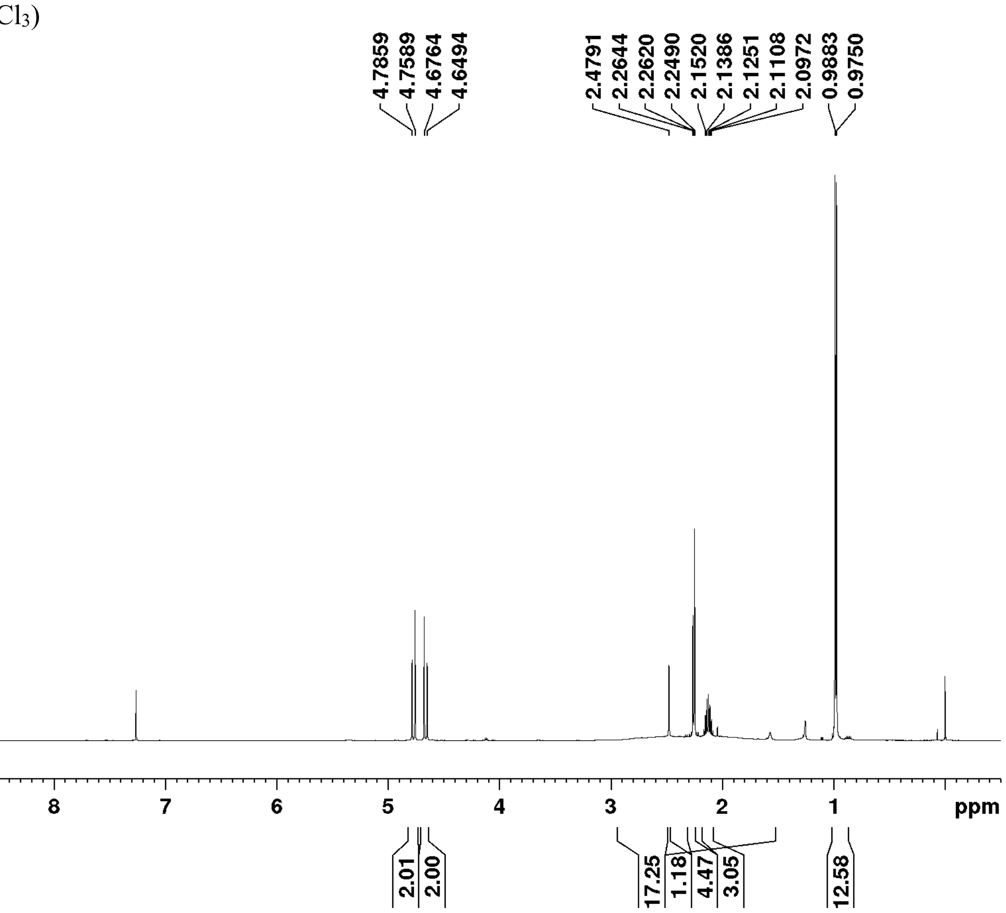

^{13}C NMR (125 MHz; $CDCl_3$)

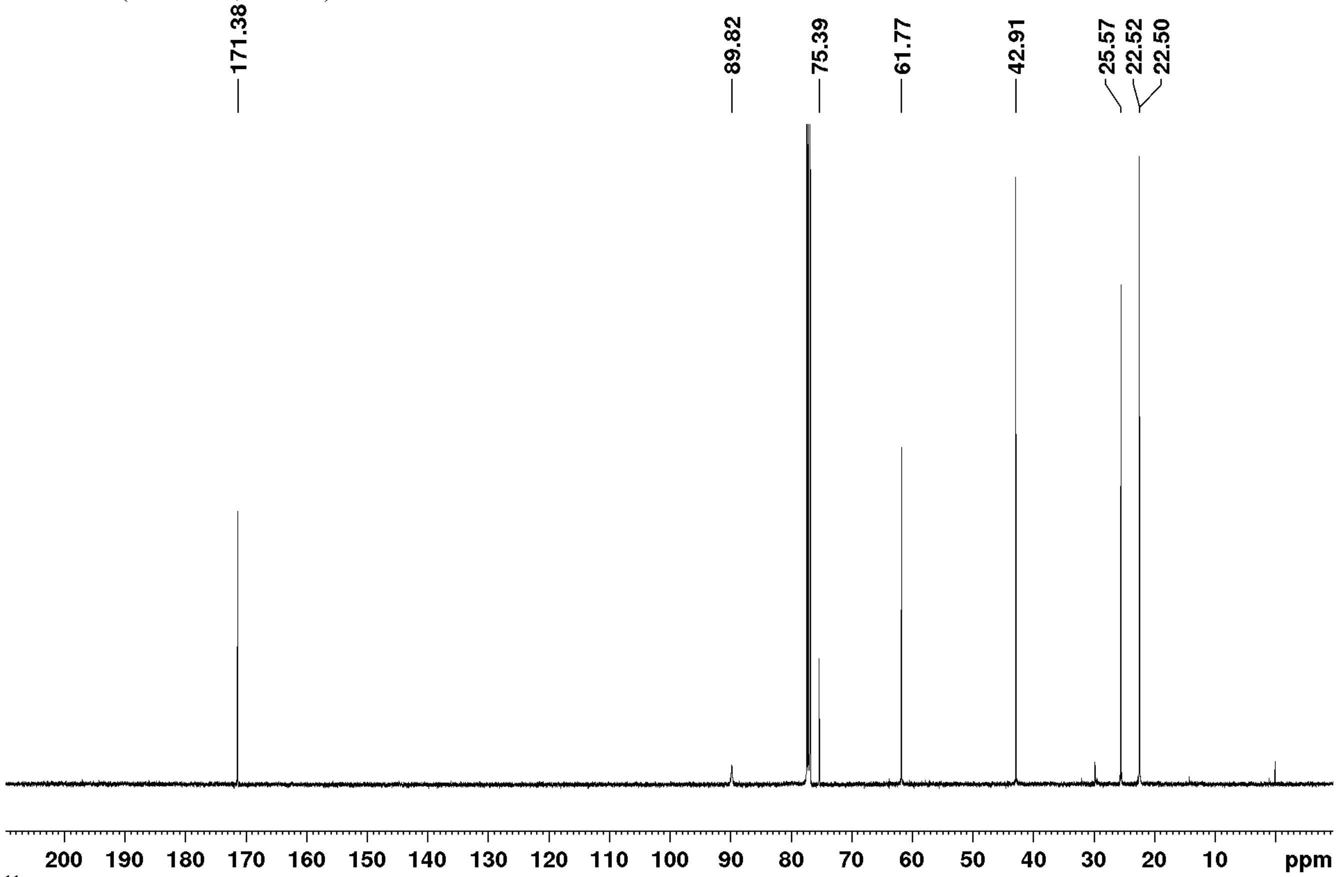

^{11}B NMR (160 MHz; $CDCl_3$)

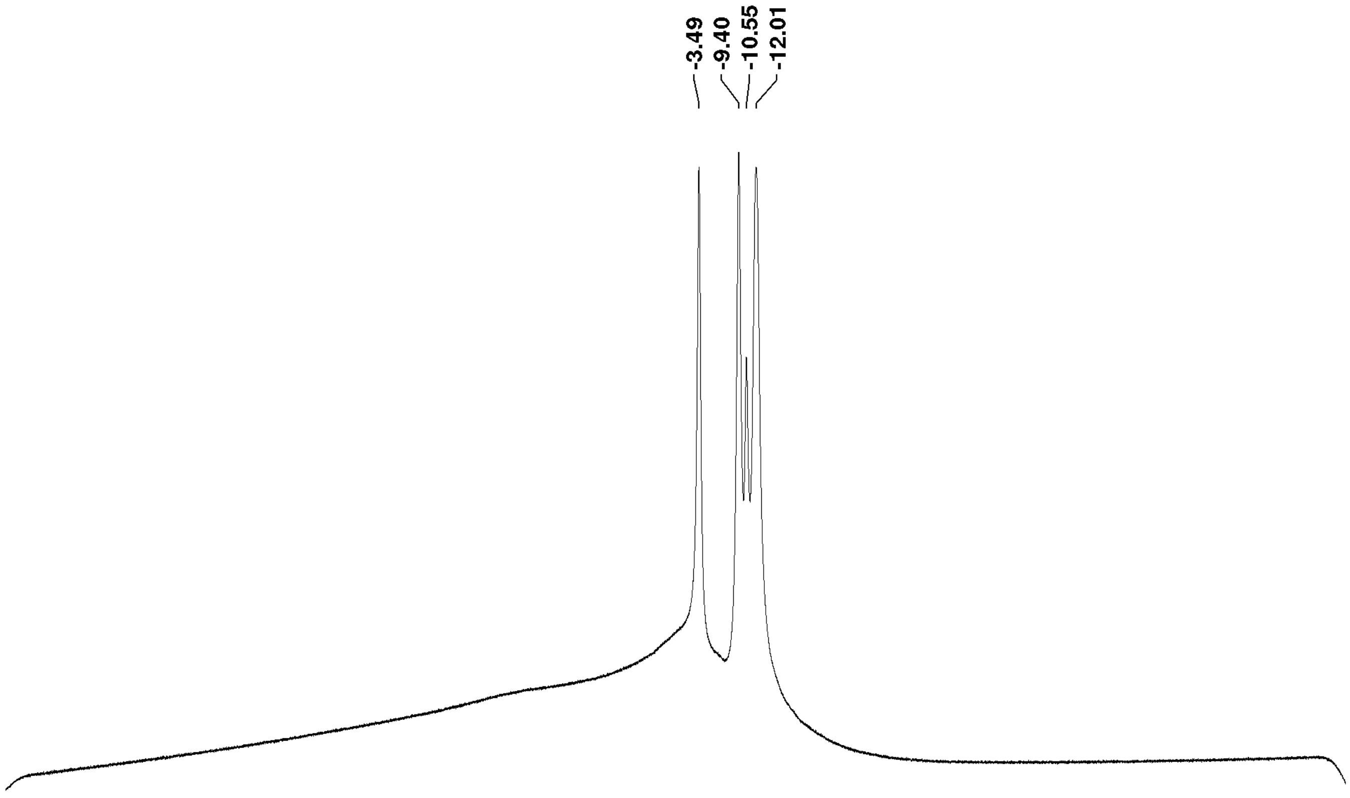

1-(3-methylbutanoyl)oxymethyl-2-phenylacetoxymethyl-3-(1*H*-1,2,3-triazol-4-yl)benzylcarbamoyl-1,2-dicarba-*closo*-dodecaborane (Ia)

^{1}H NMR (500 MHz; $CDCl_3$)

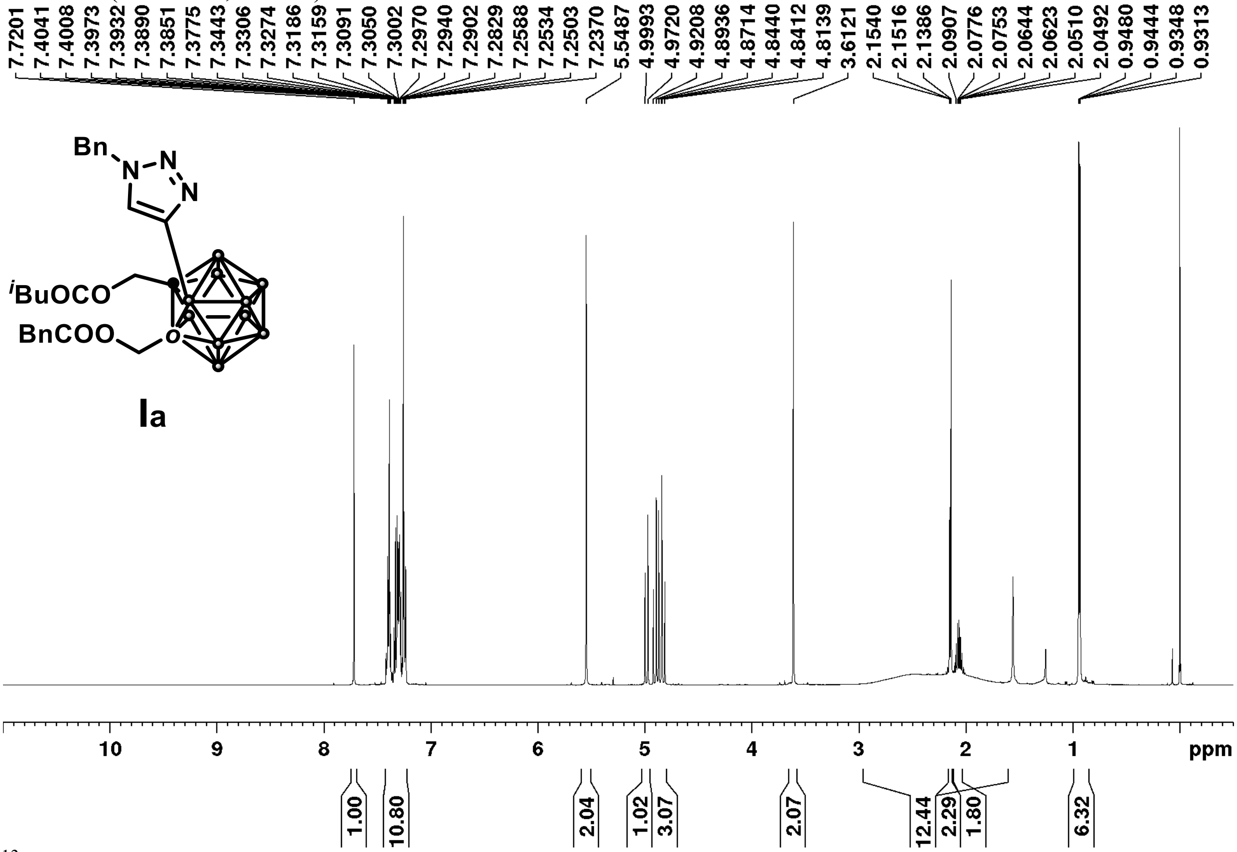

^{13}C NMR (125 MHz; $CDCl_3$)

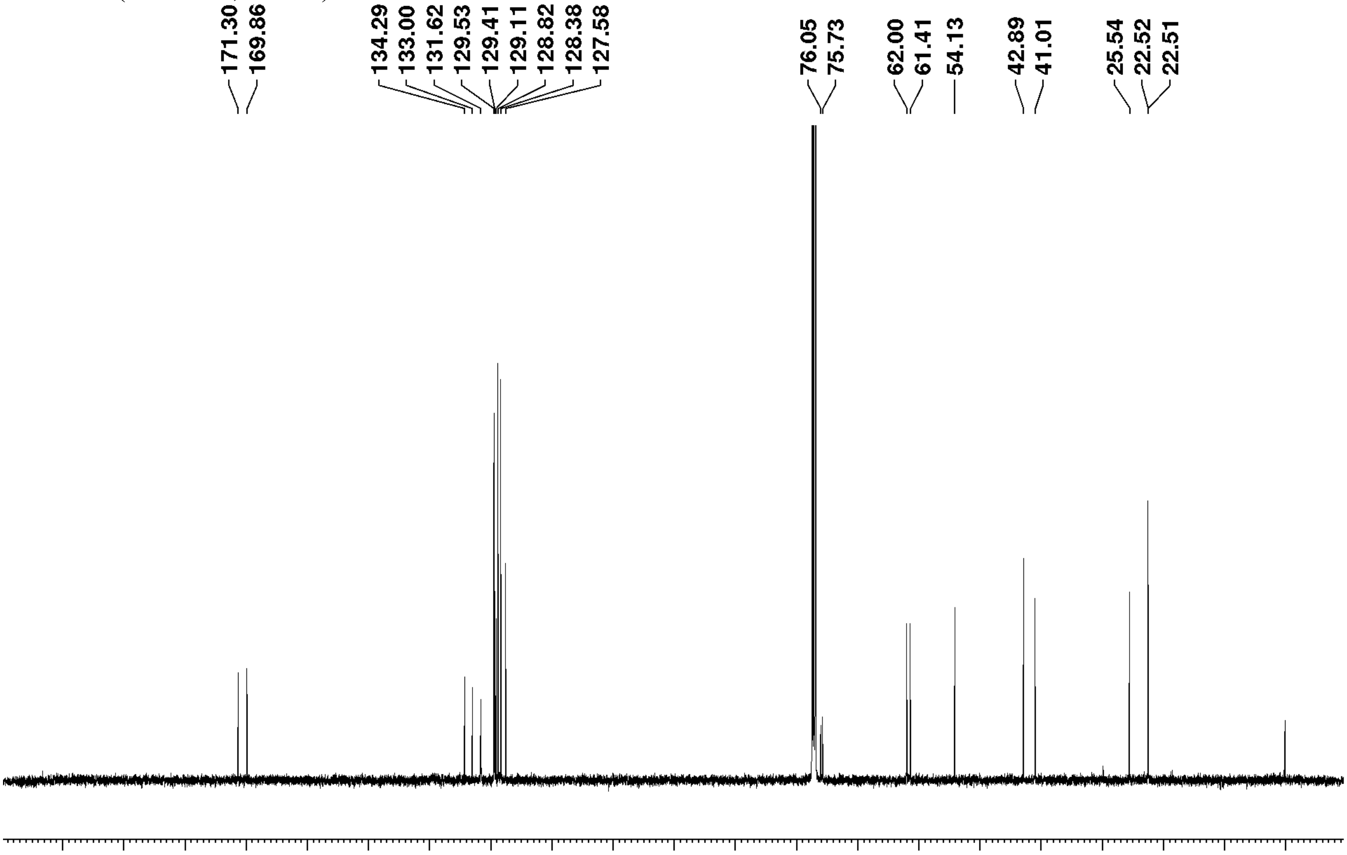

^{11}B NMR (160 MHz; $CDCl_3$)

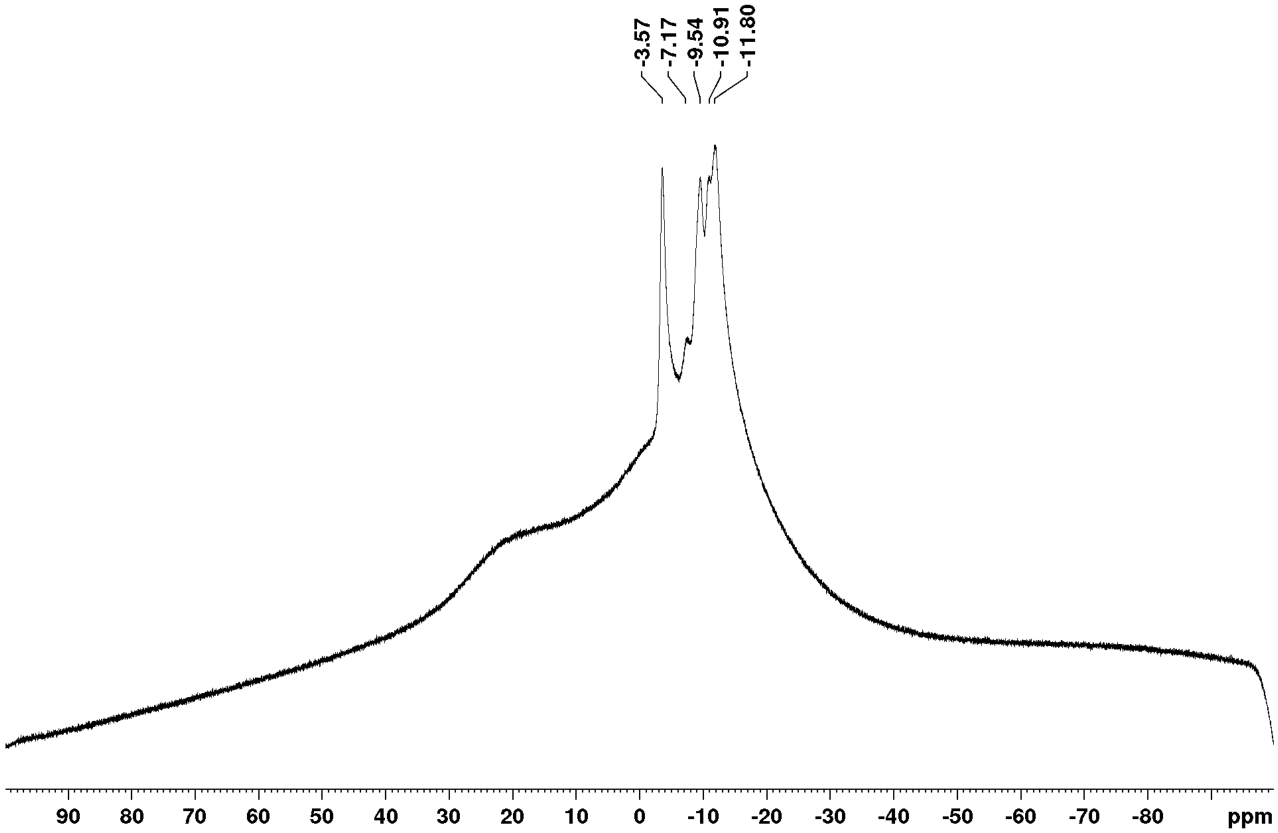

1-(3-methylbutanoyl)oxymethyl-2-phenylacetoxymethyl-3-((1*H*-1,2,3-triazol-4-yl)-3-methyl-butanoyl)-1,2-dicarba-*closo*-dodecaborane (Ib)

^{1}H NMR (500 MHz; $CDCl_3$)

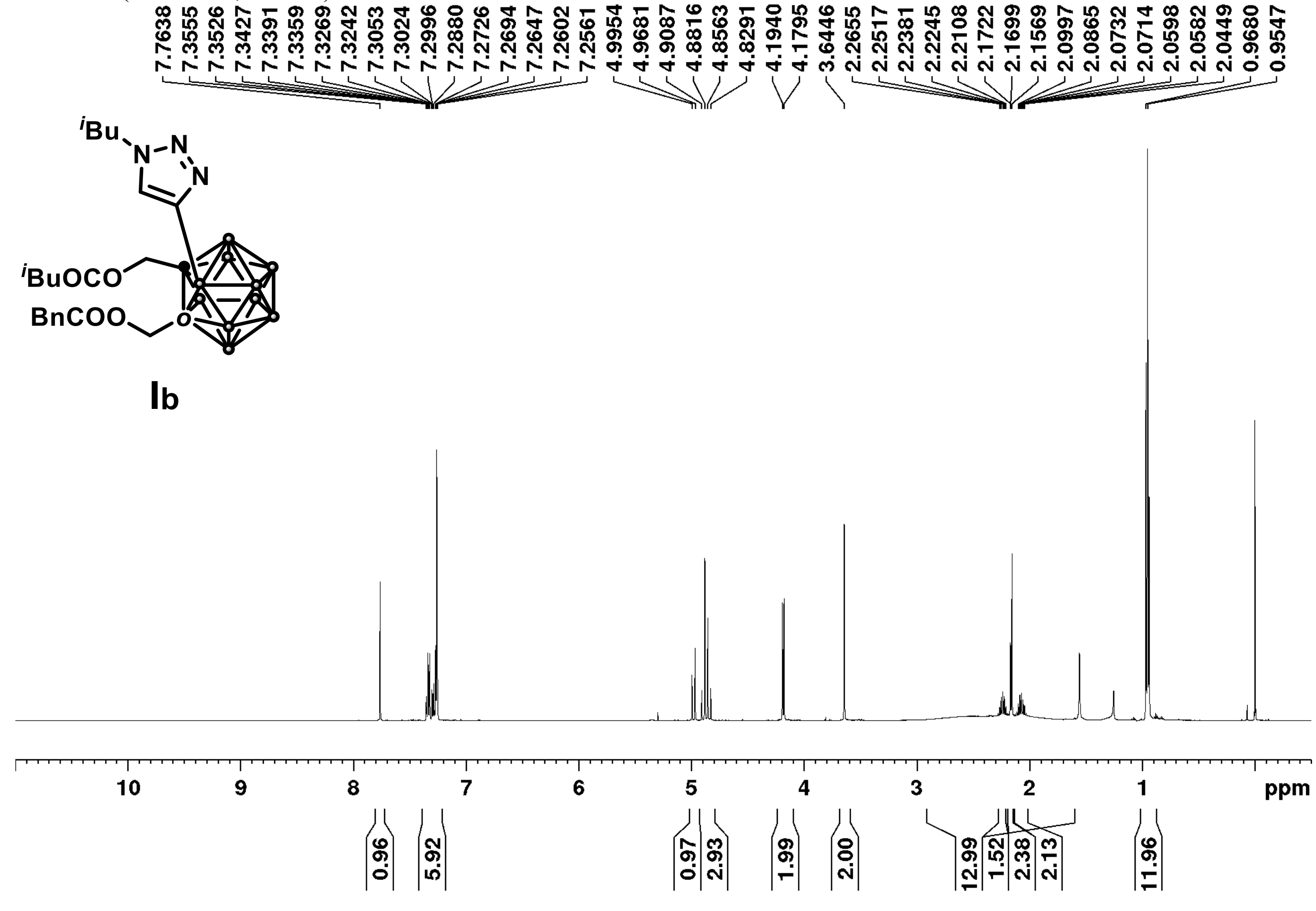

^{13}C NMR (125 MHz; $CDCl_3$)

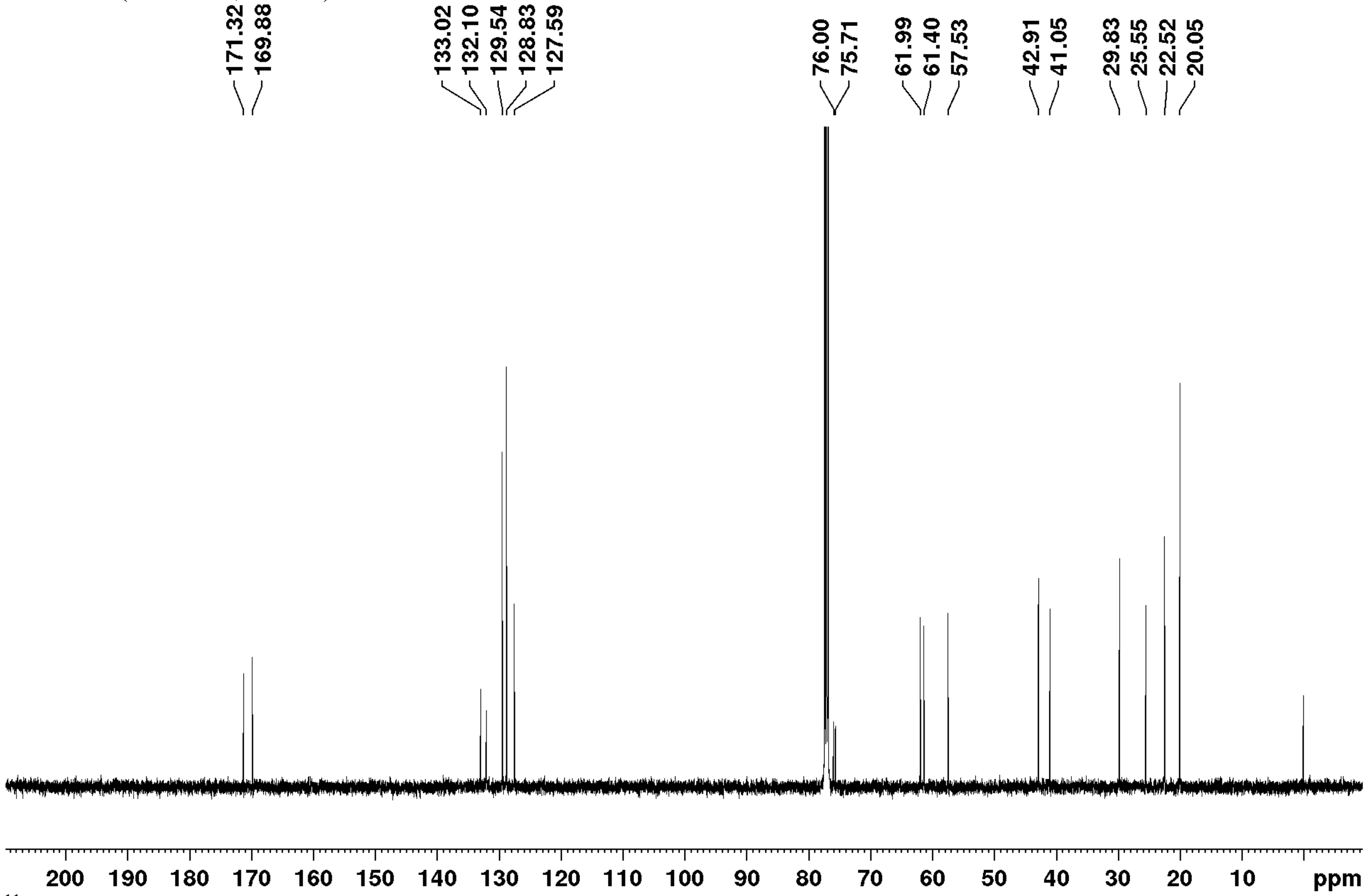

^{11}B NMR (160 MHz; $CDCl_3$)

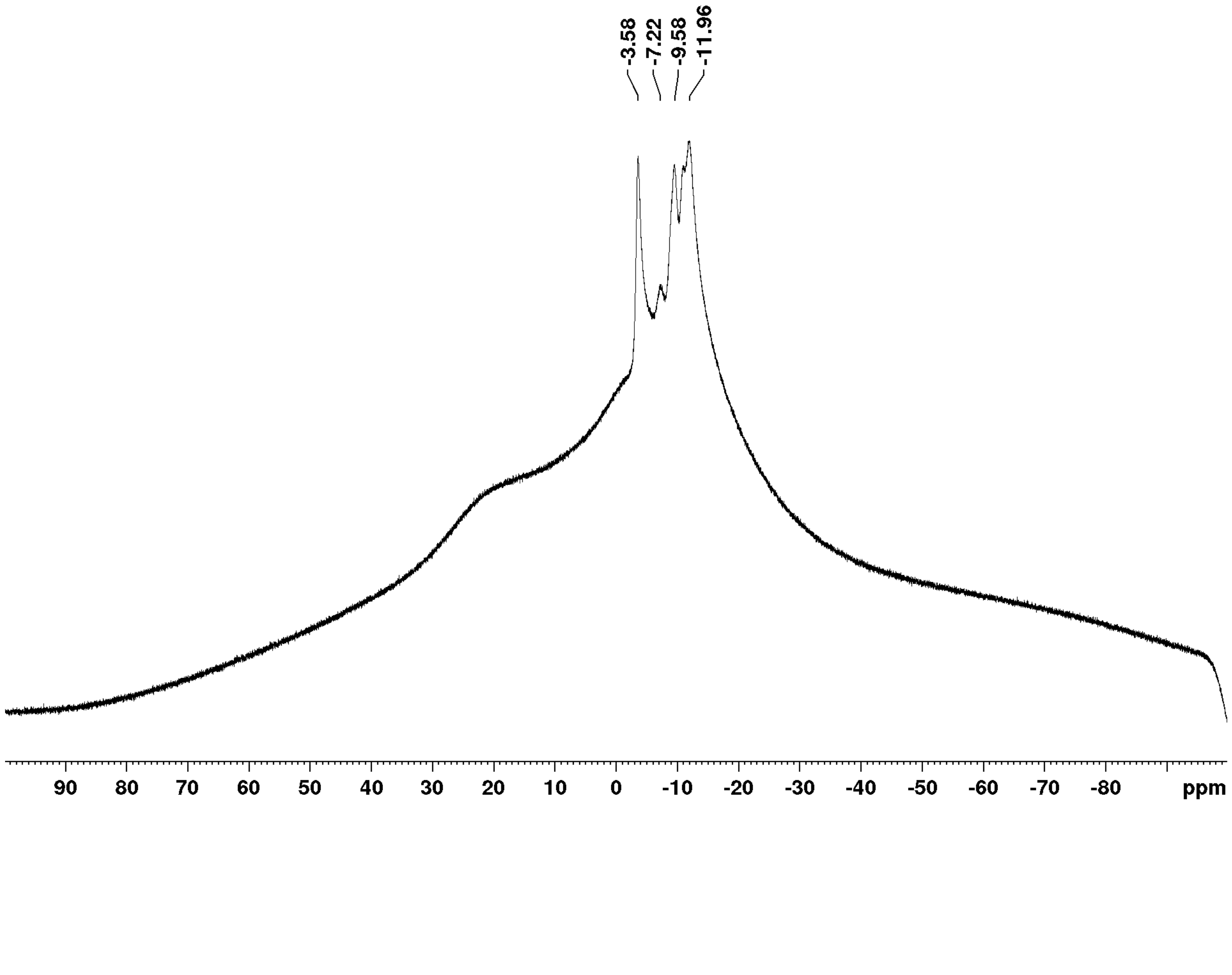

1,2-bis(phenylacetoxymethyl)-3-(1*H*-1,2,3-triazol-4-yl)benzylcarbamoyl-1,2-dicarba-*closo*-dodecaborane (Ic)

^{1}H NMR (500 MHz; $CDCl_3$)

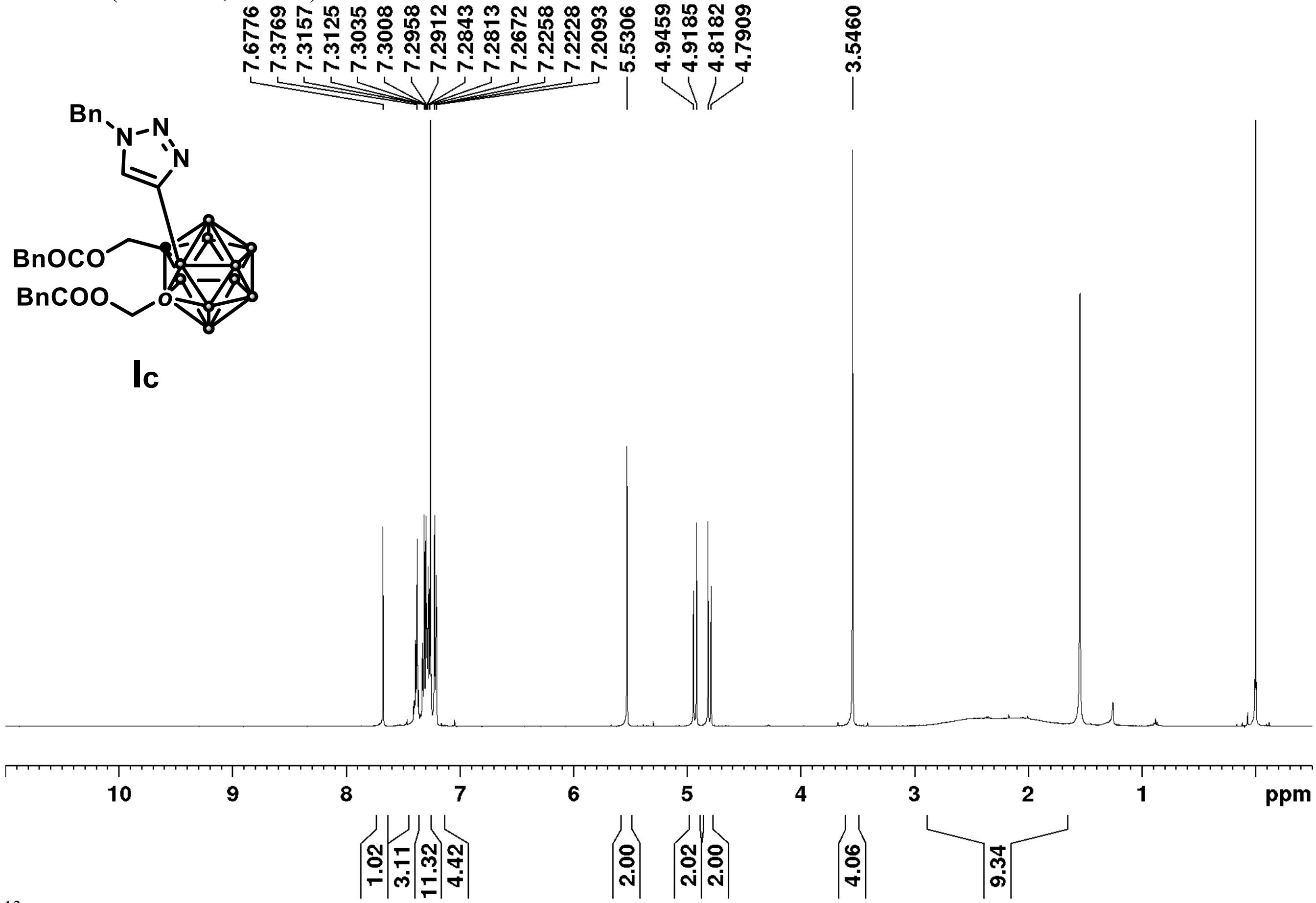

Ic

^{13}C NMR (125 MHz; $CDCl_3$)

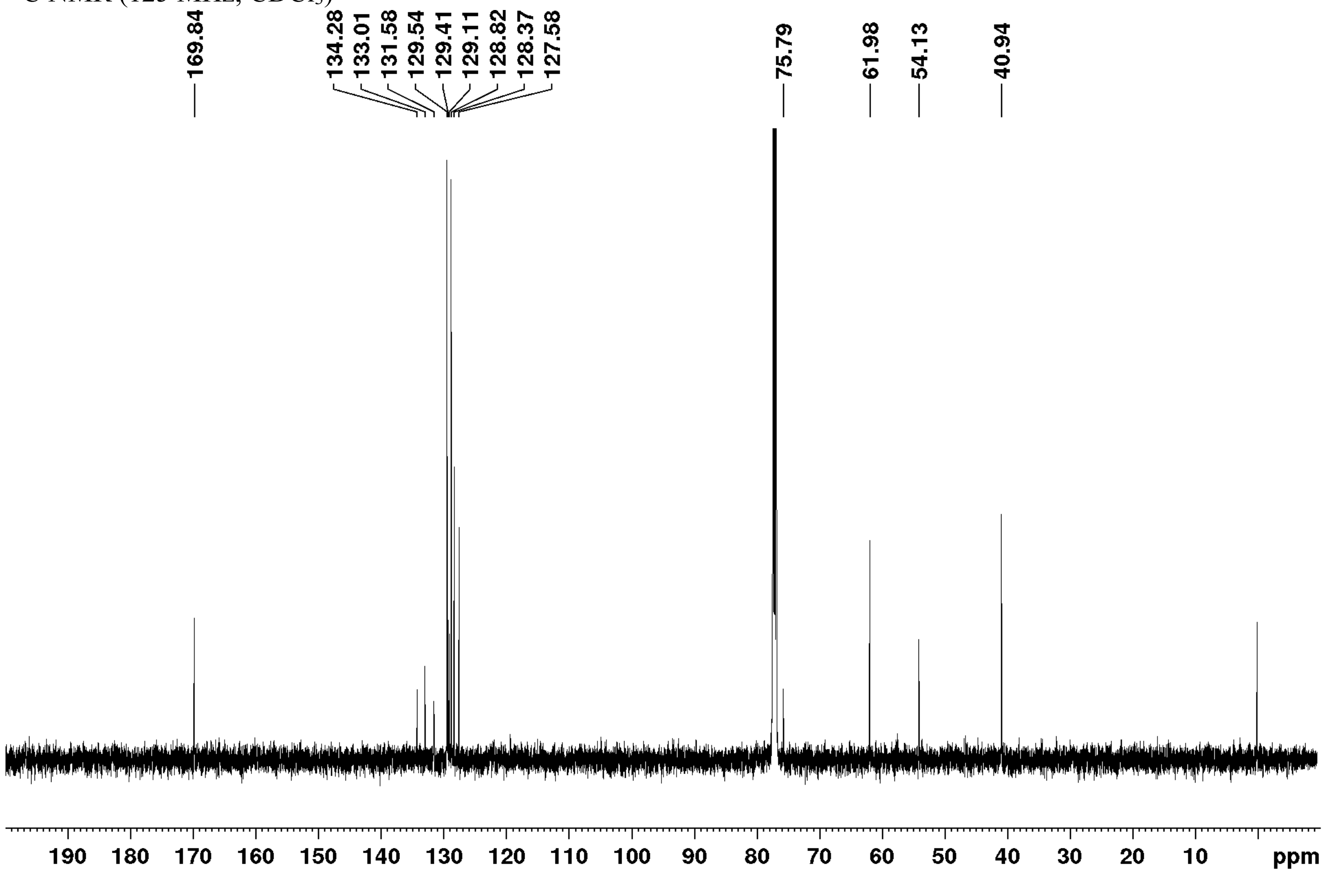

^{11}B NMR (160 MHz; $CDCl_3$)

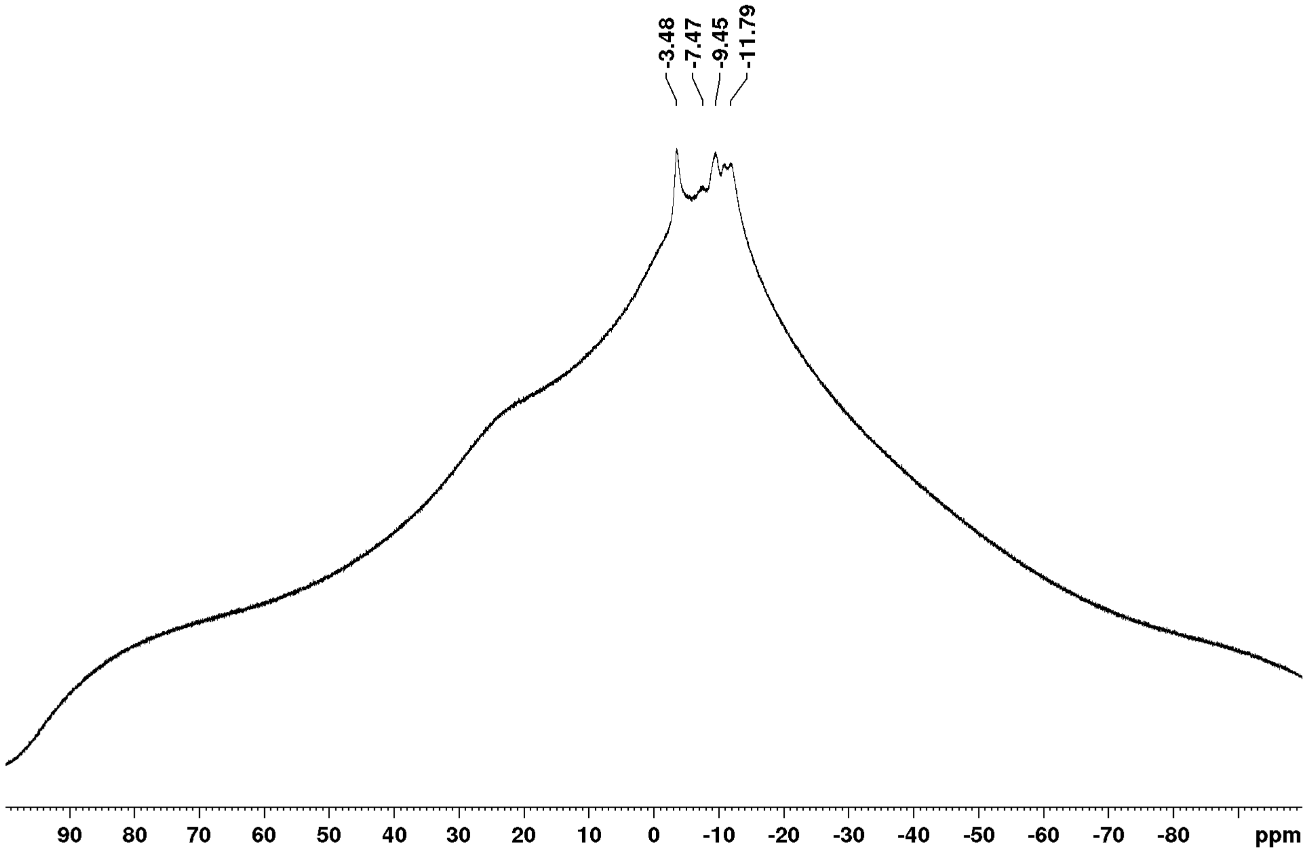

1,2-bis(phenylacetoxymethyl)-3-((1*H*-1,2,3-triazol-4-yl)-3-methyl-butanoyl)-1,2-dicarba-*closo*-dodecaborane (Id)

^{1}H NMR (500 MHz; $CDCl_3$)

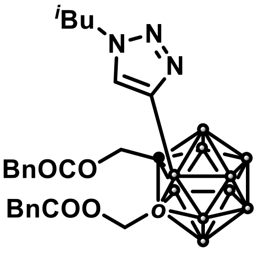

Id

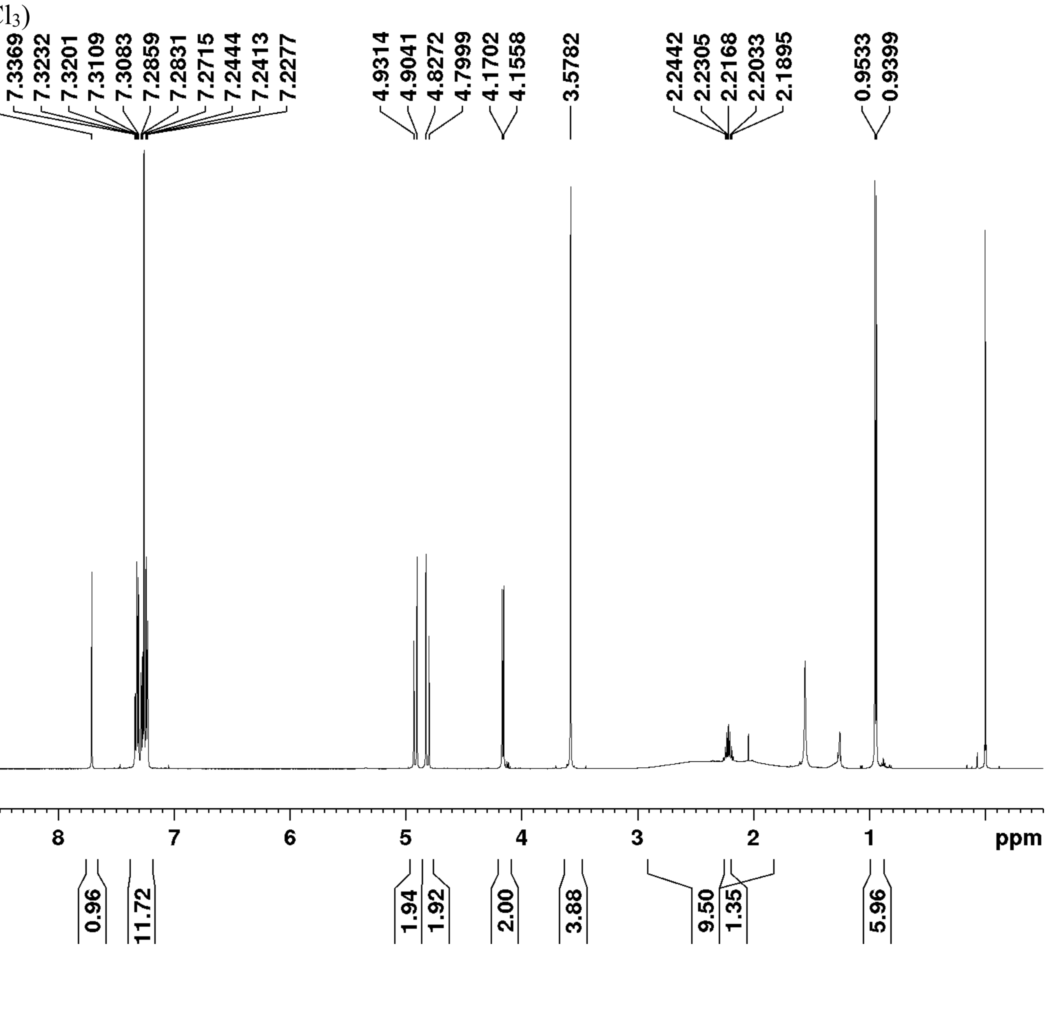

^{13}C NMR (125 MHz; $CDCl_3$)

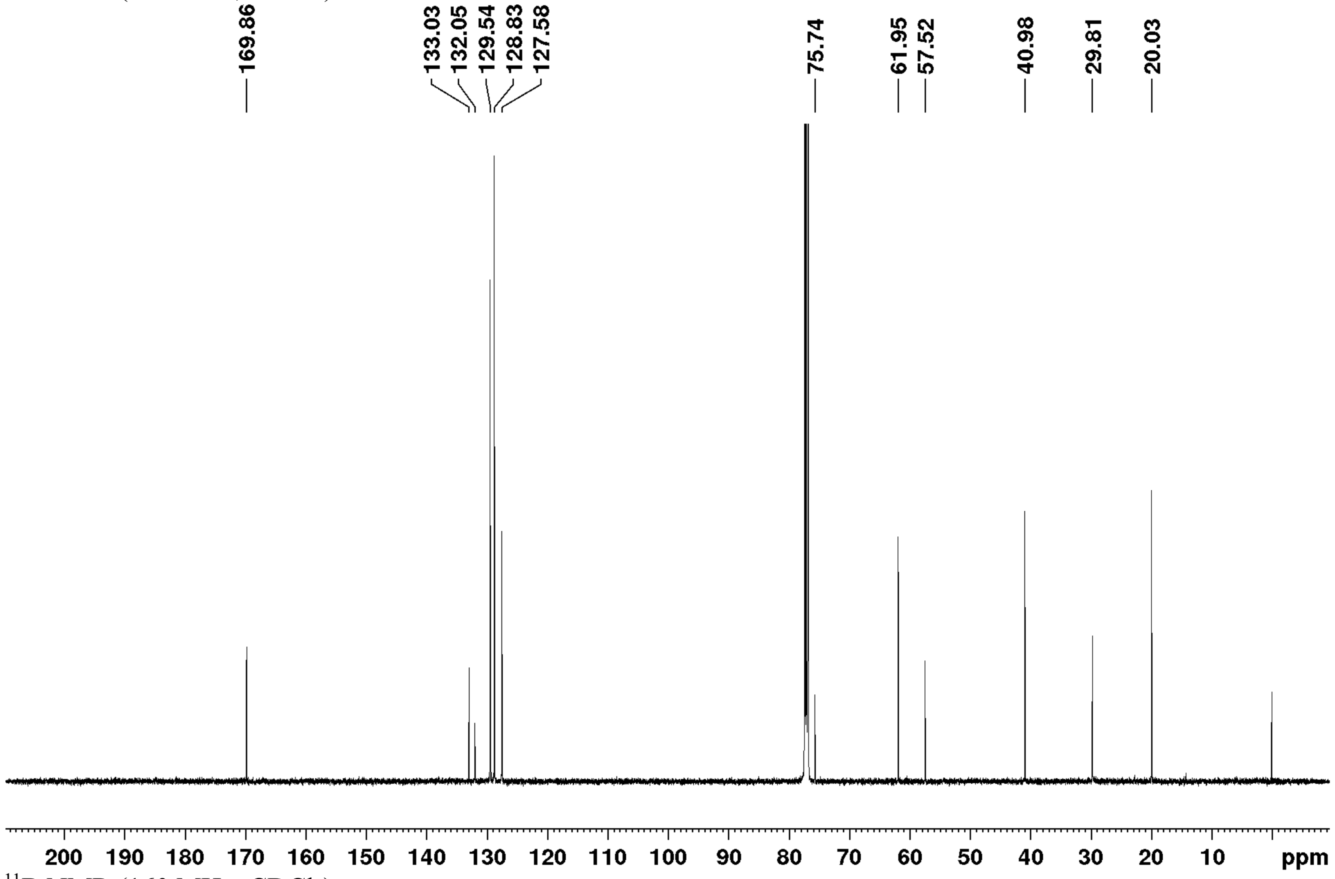

^{11}B NMR (160 MHz; $CDCl_3$)

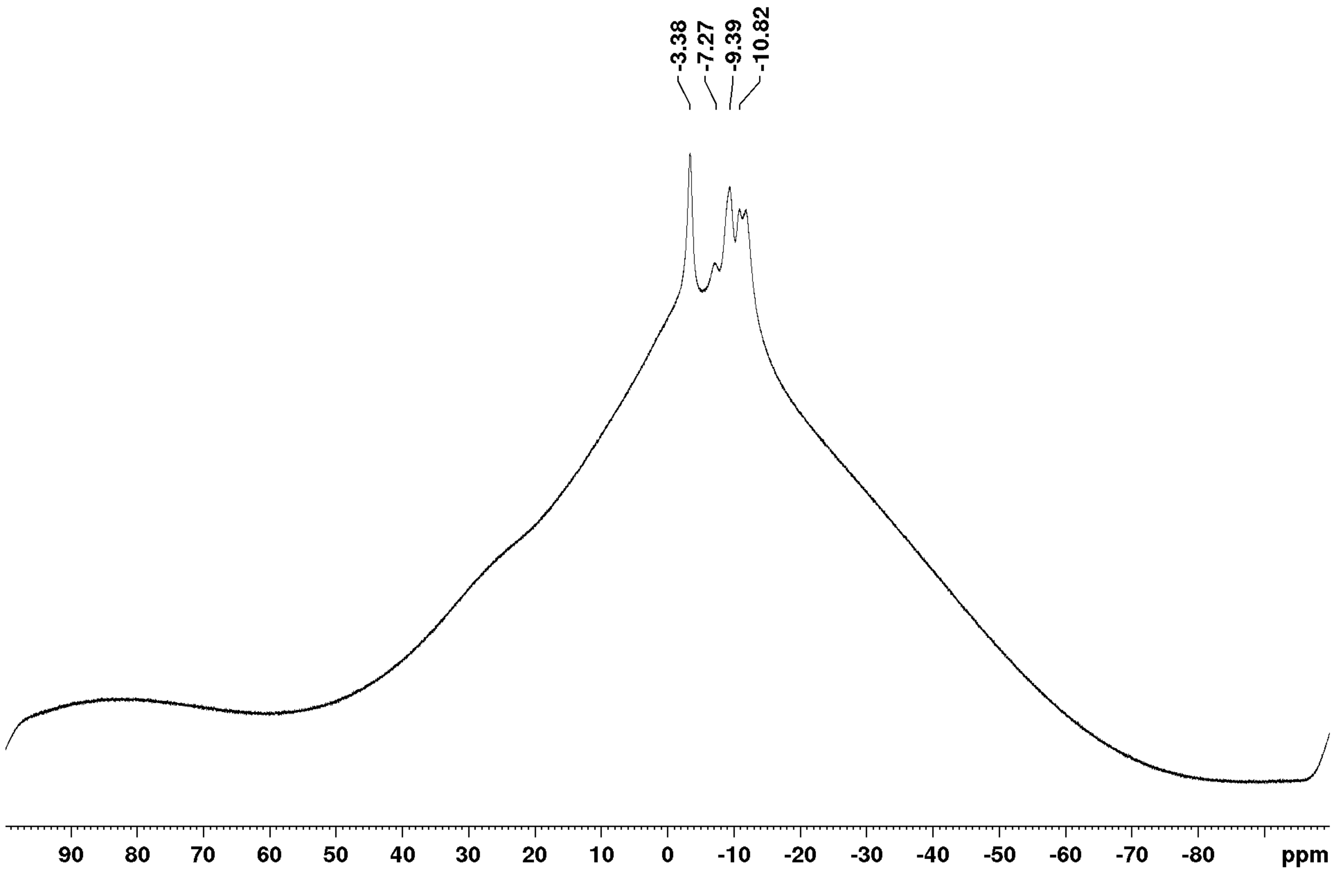

1,2-bis((3-methylbutanoyl)oxymethyl)-3-(1*H*-1,2,3-triazol-4-yl)benzylcarbamoyl-1,2-dicarba-*closo*-dodecaborane (Ie)

^{1}H NMR (500 MHz; $CDCl_3$)

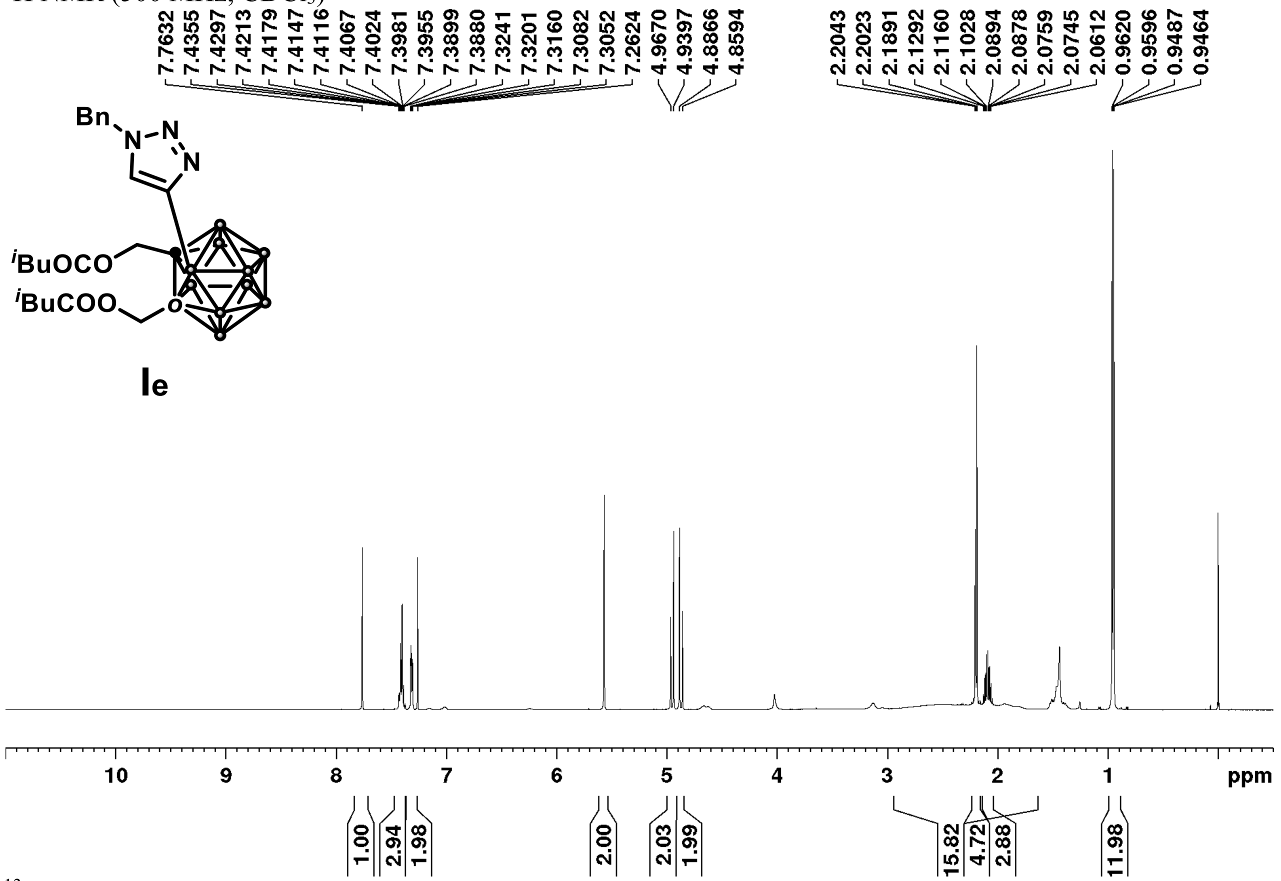

^{13}C NMR (125 MHz; $CDCl_3$)

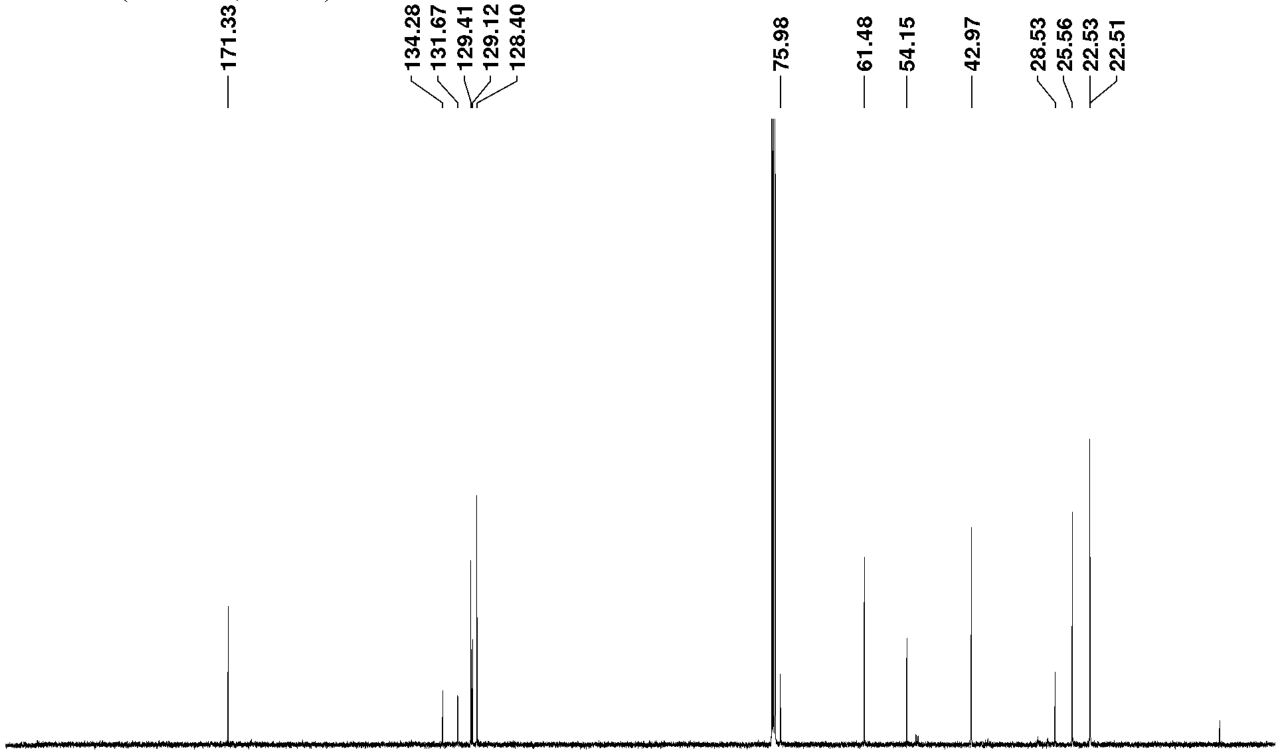

^{11}B NMR (160 MHz; $CDCl_3$)

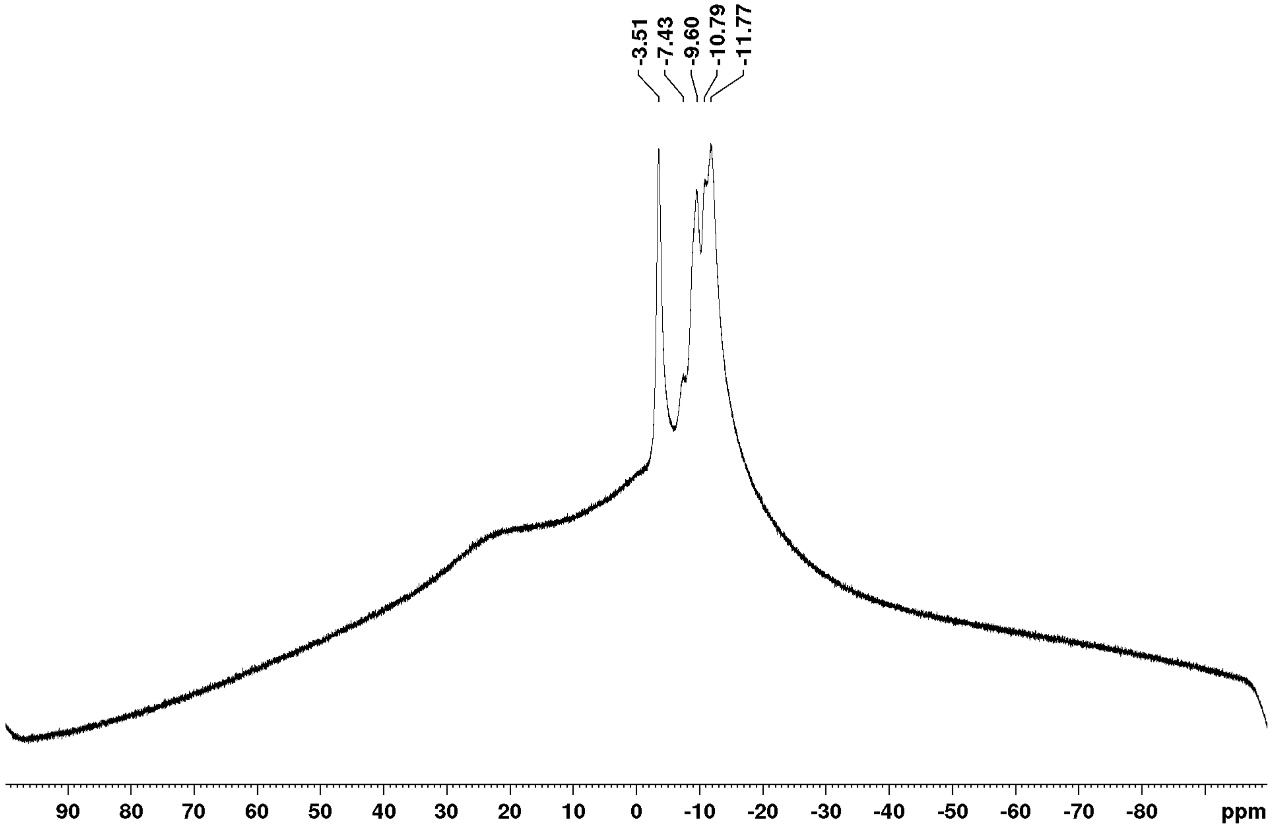

1,2-bis((3-methylbutanoyl)oxymethyl)-3-((1*H*-1,2,3-triazol-4-yl)-3-methyl-butanoyl)-1,2-dicarba-*closo*-dodecaborane (If)

^{1}H NMR (500 MHz; $CDCl_3$)

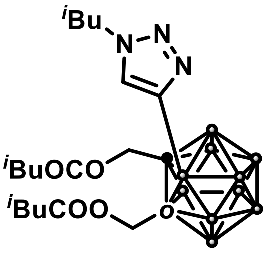

If

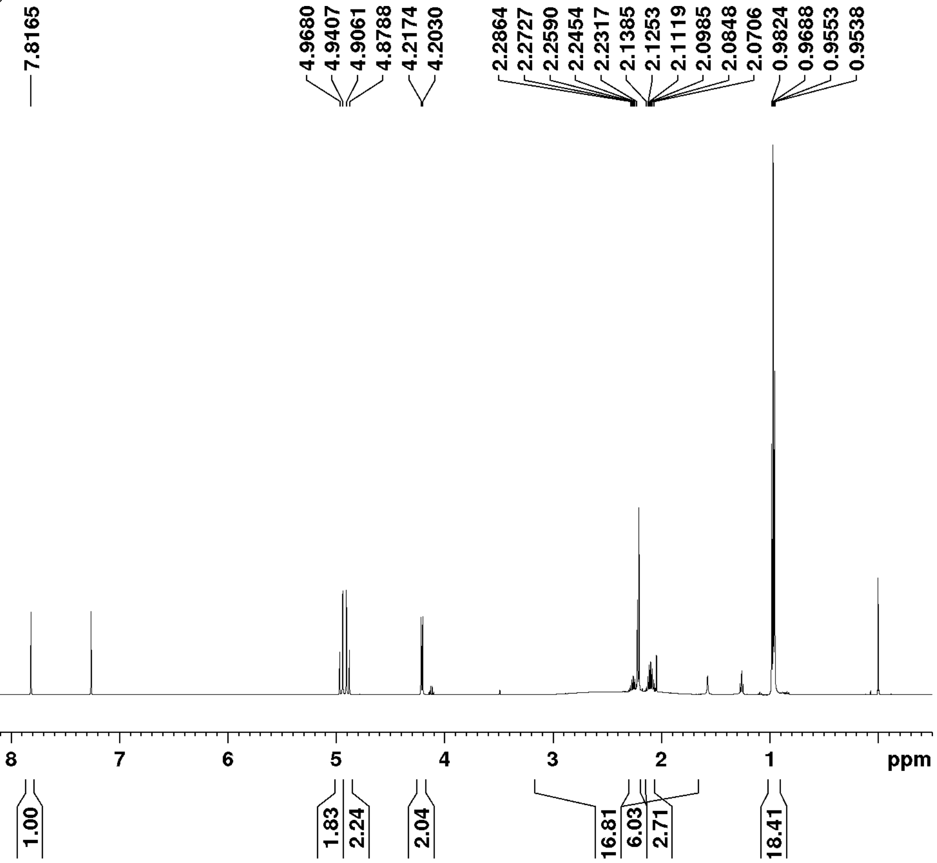

^{13}C NMR (125 MHz; $CDCl_3$)

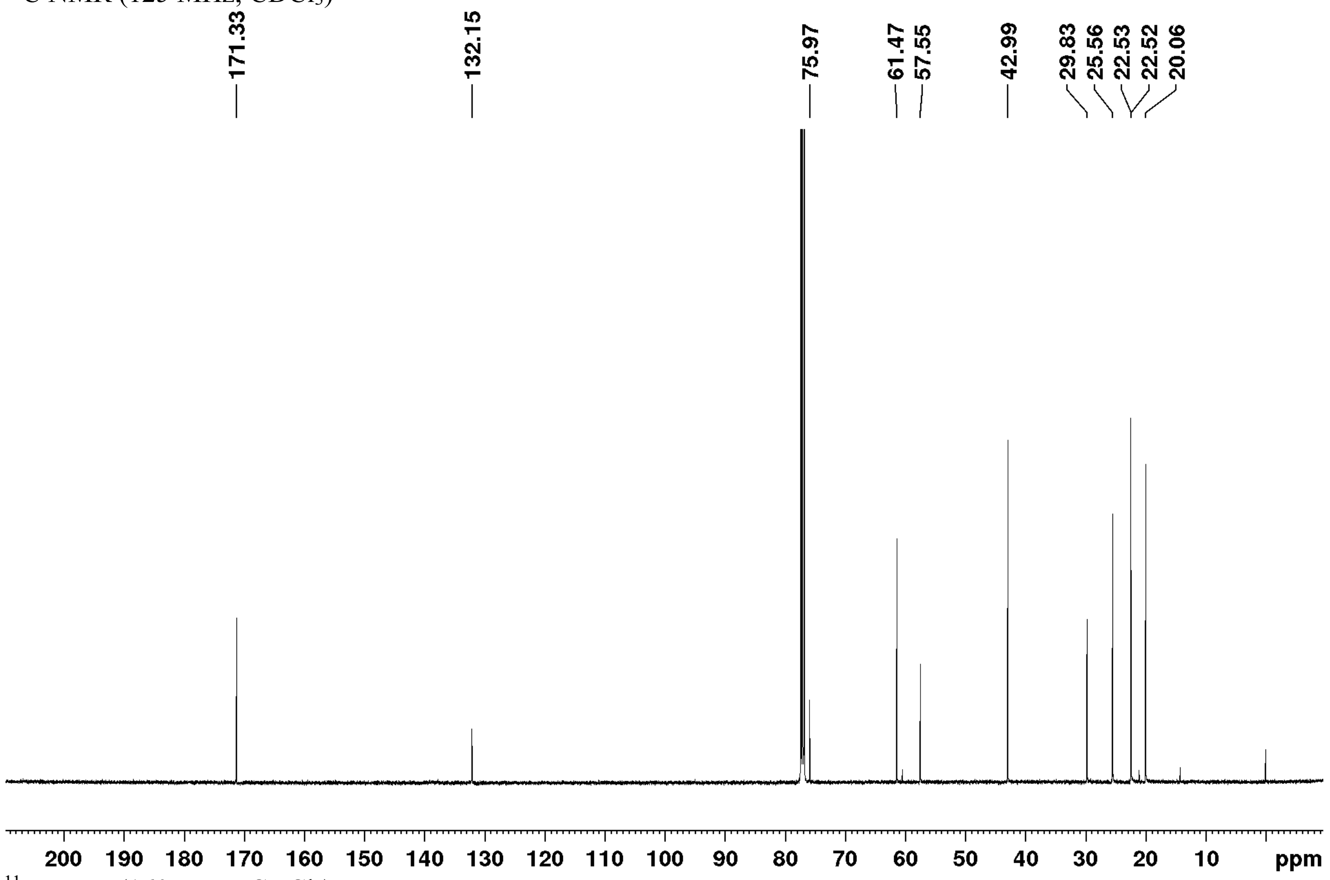

^{11}B NMR (160 MHz; $CDCl_3$)

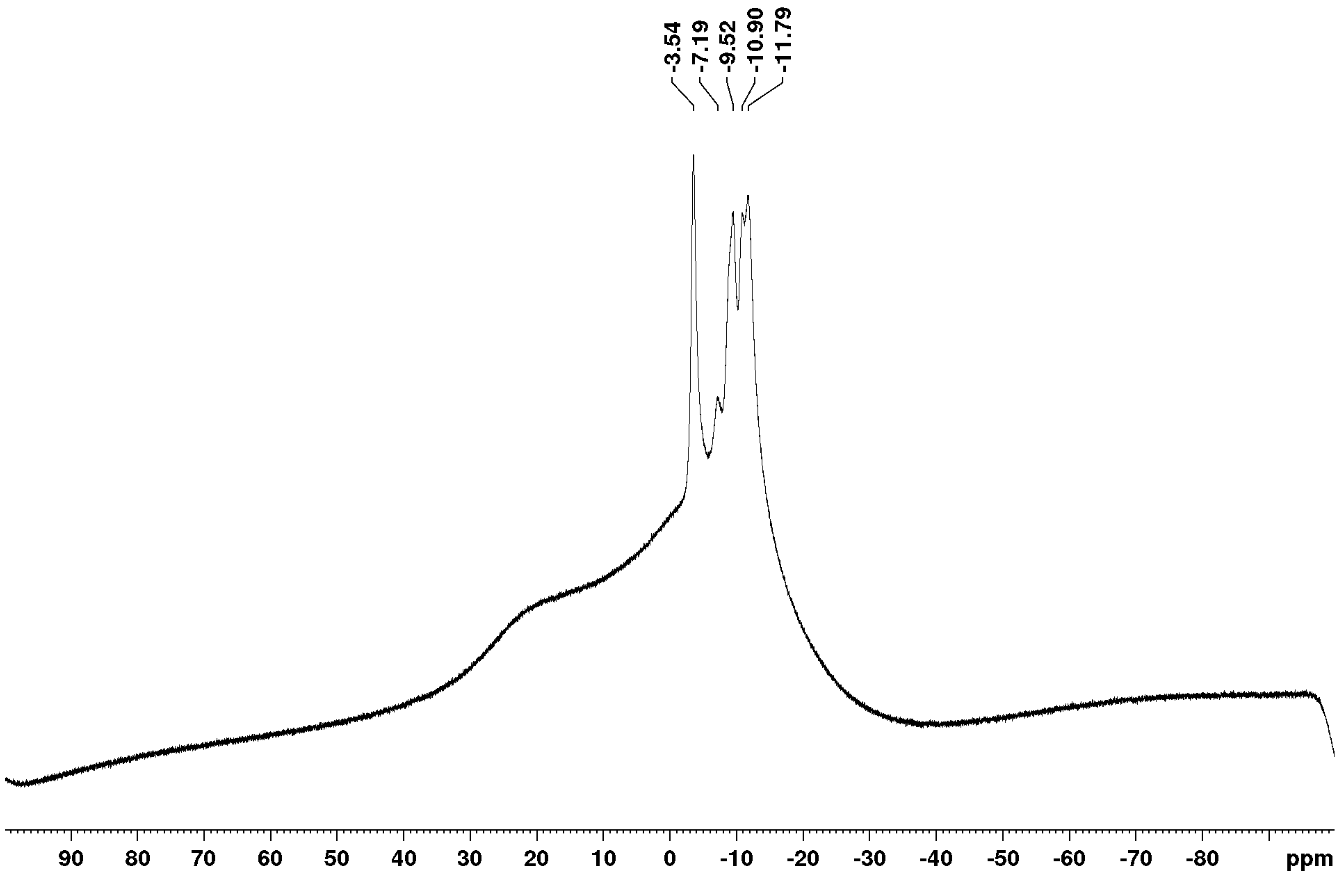

1-(3-((4-methoxybenzyl)oxy)prop-1-yn-1-yl)-1,2-dicarba-*closo*-dodecaborane (9)

^{1}H NMR (500 MHz; $CDCl_3$)

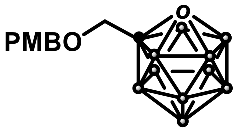

9

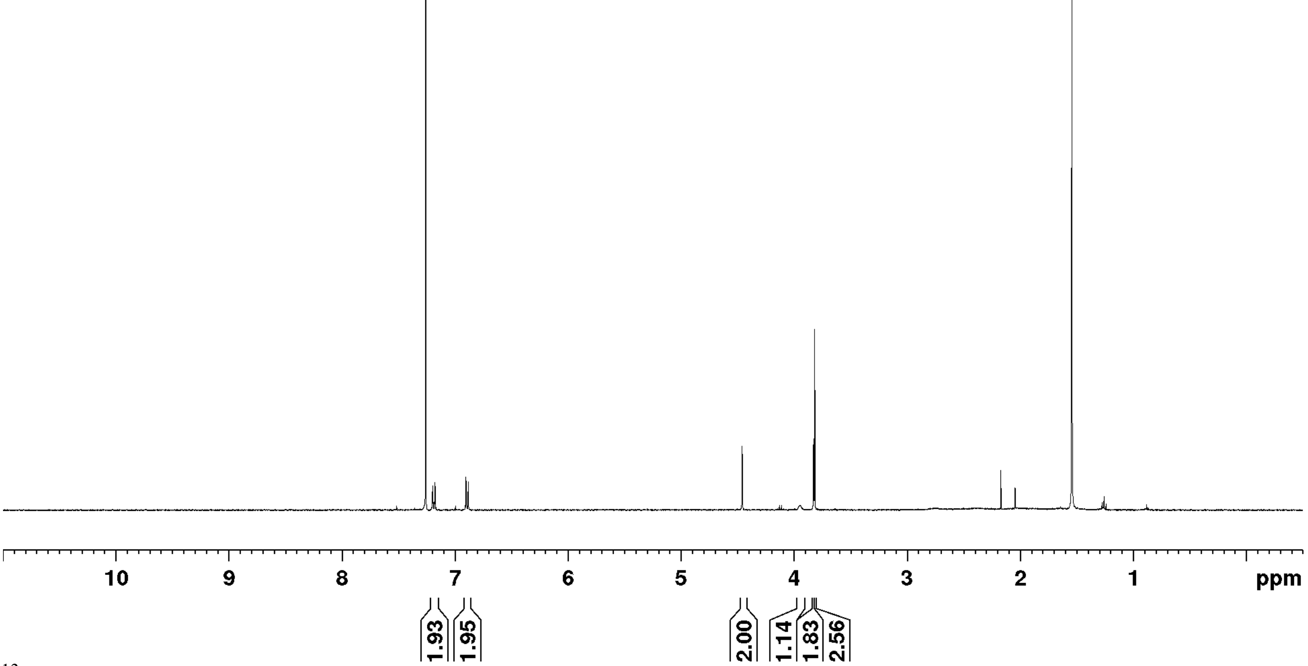

^{13}C NMR (125 MHz; $CDCl_3$)

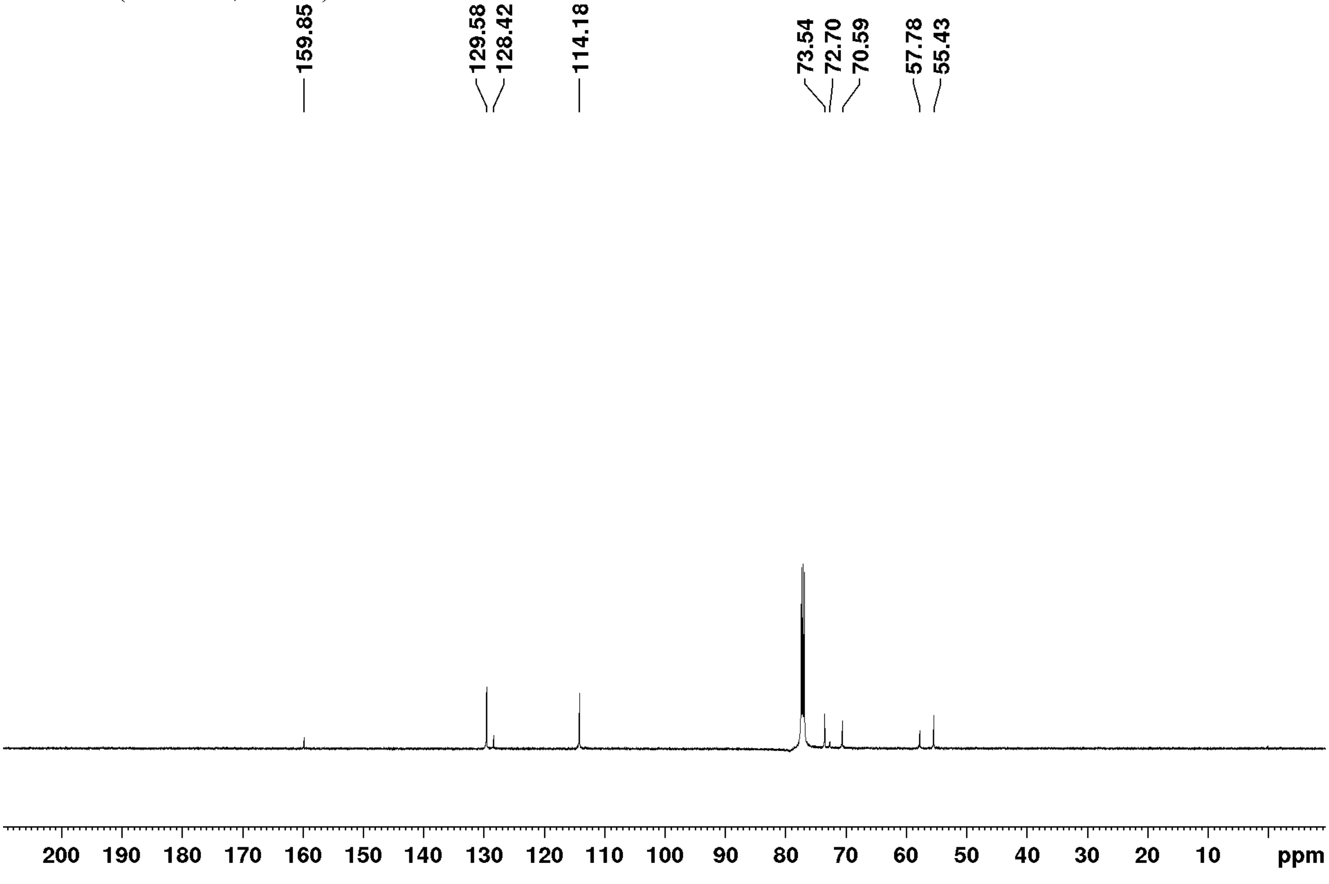

^{11}B NMR (160 MHz; $CDCl_3$)

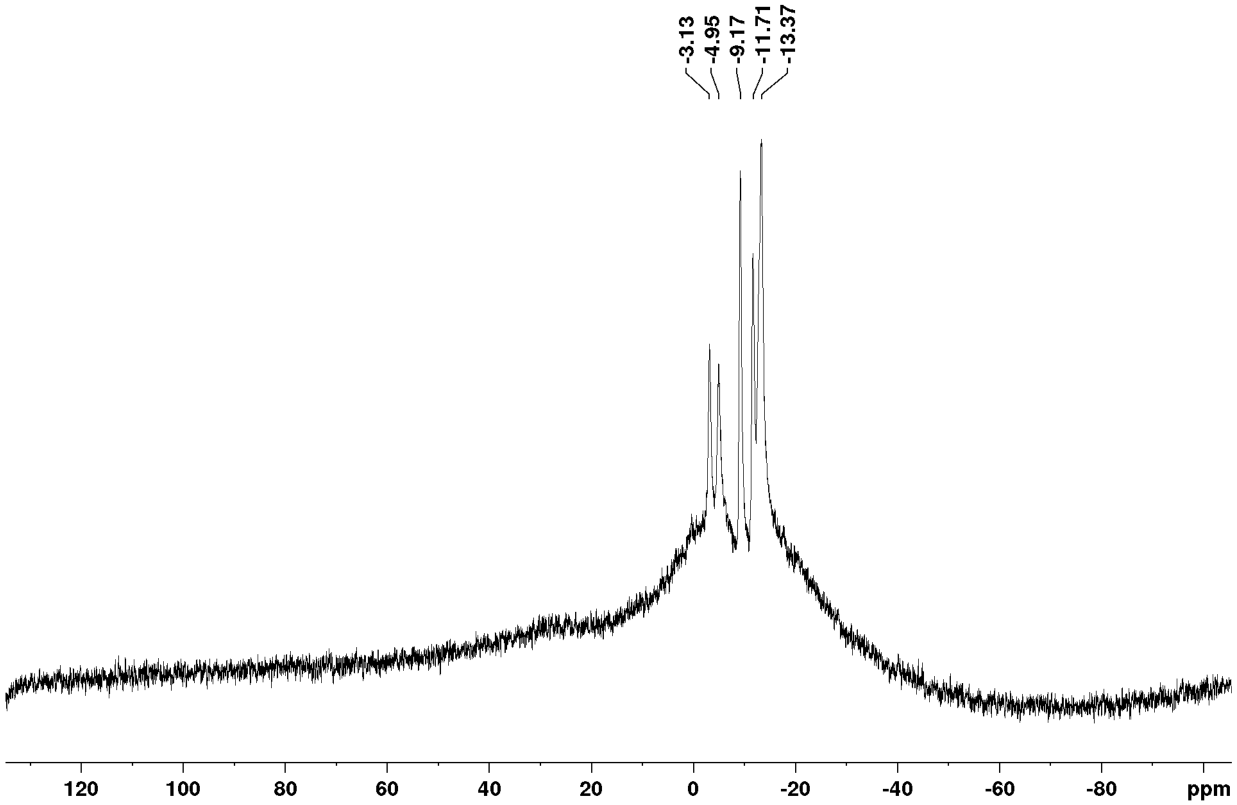

1-(3-(4-methoxybenzyl)hydroxymethyl)-4-((triisopropylsilyl)ethynyl)-1,2-dicarba-*closo*-dodecaborane (10)

^{1}H NMR (500 MHz; $CDCl_3$)

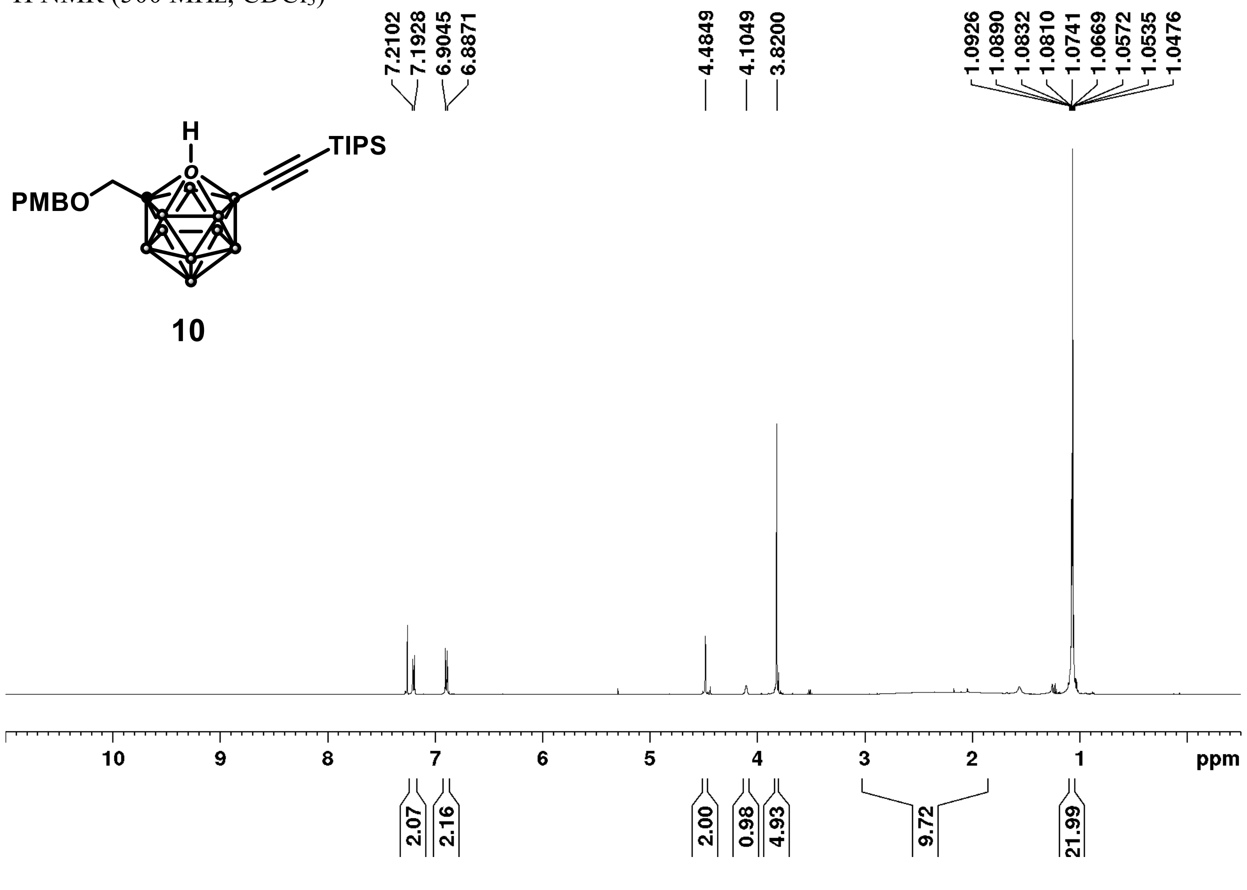

^{13}C NMR (125 MHz; $CDCl_3$)

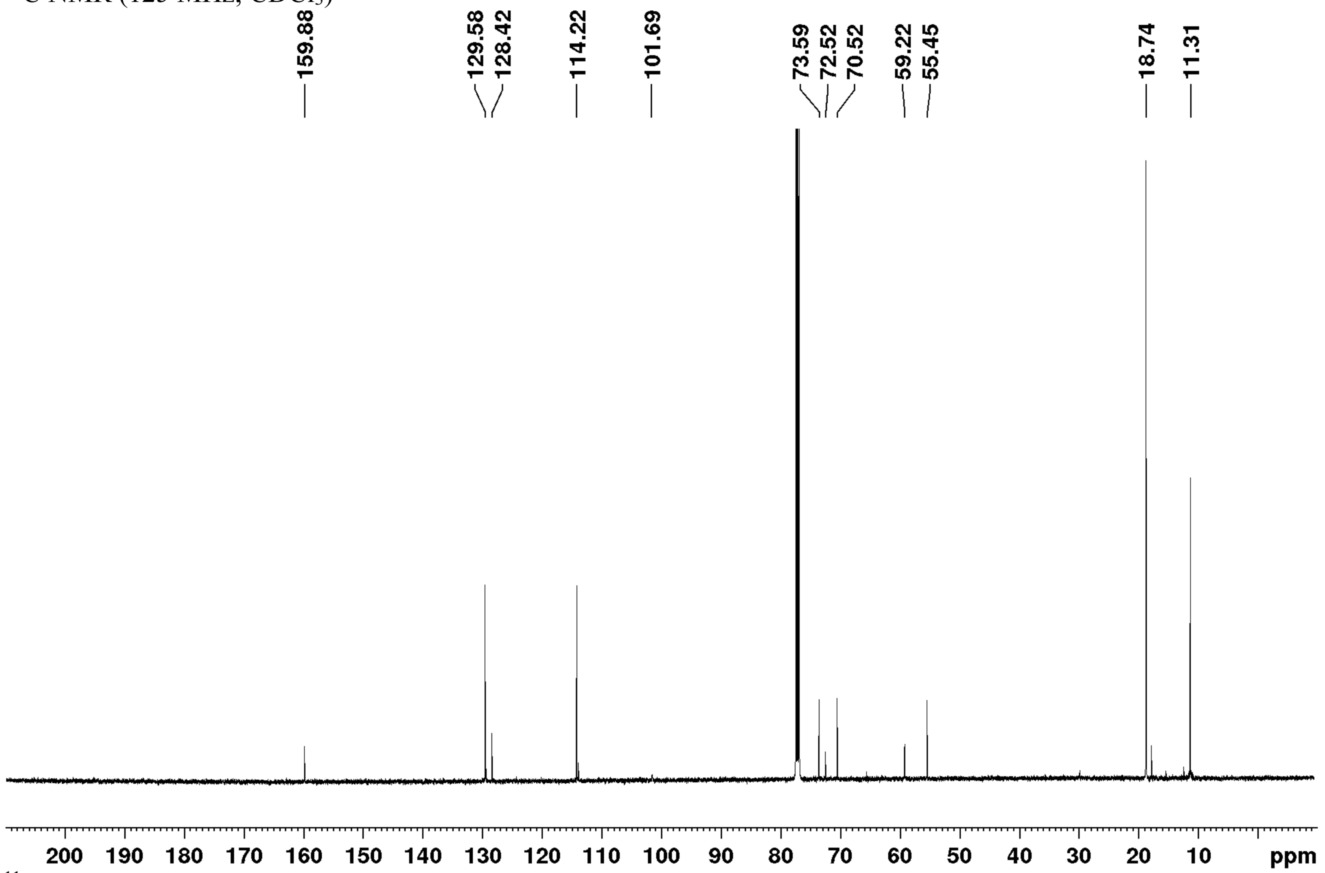

^{11}B NMR (160 MHz; $CDCl_3$)

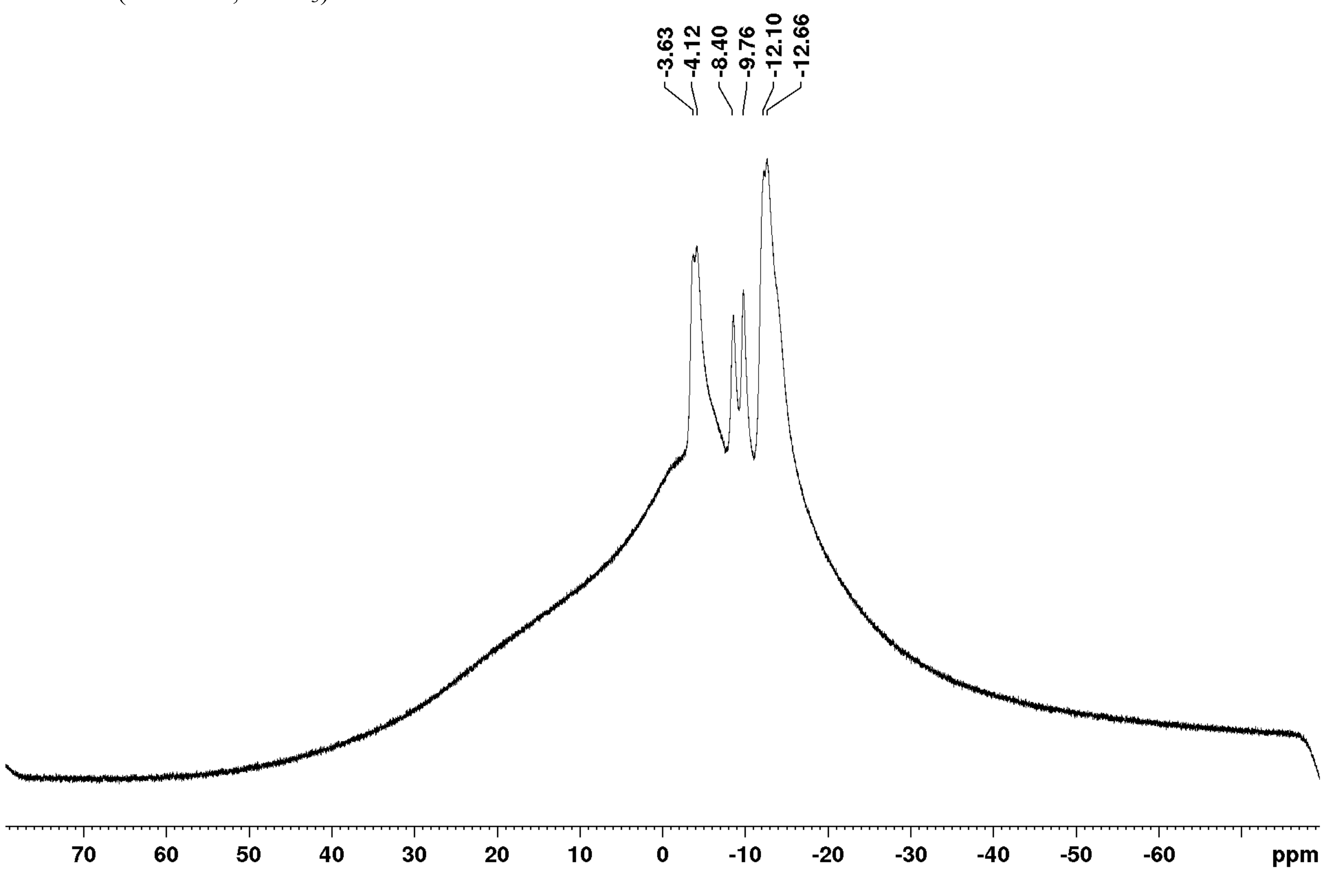

1-(3-(4-methoxybenzyl)hydroxymethyl)-2-(hydroxymethyl)-4-((triisopropylsilyl)ethynyl)-1,2-dicarba-*closo*-dodecaborane (S3)

^{1}H NMR (500 MHz; CDCl$_3$)

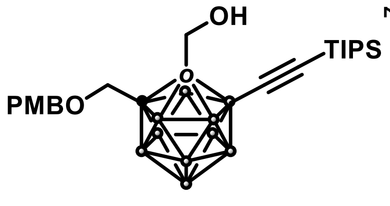

S3

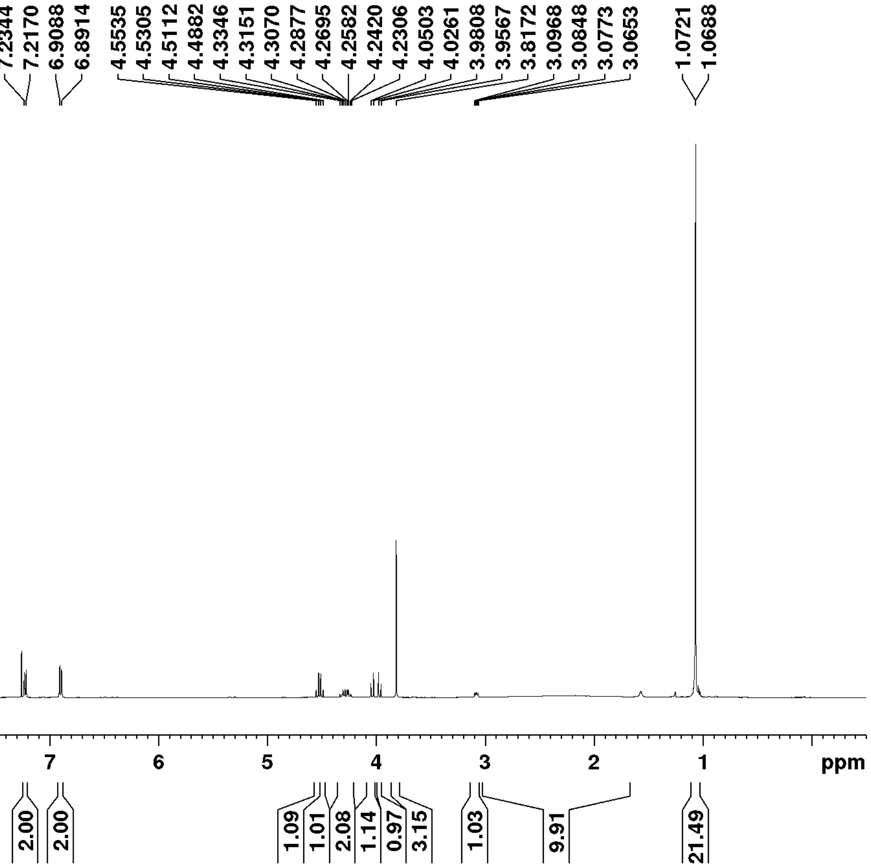

^{13}C NMR (125 MHz; CDCl$_3$)

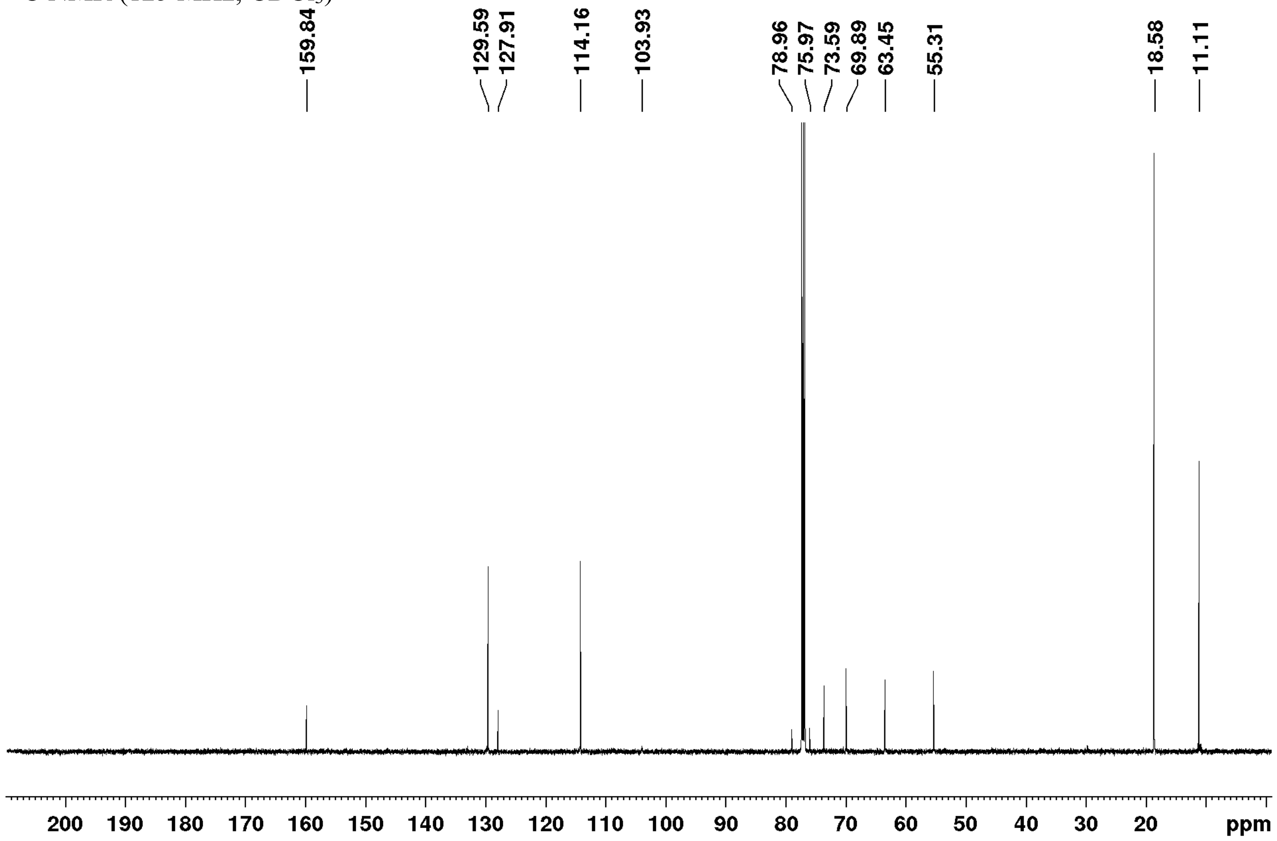

^{11}B NMR (160 MHz; $CDCl_3$)

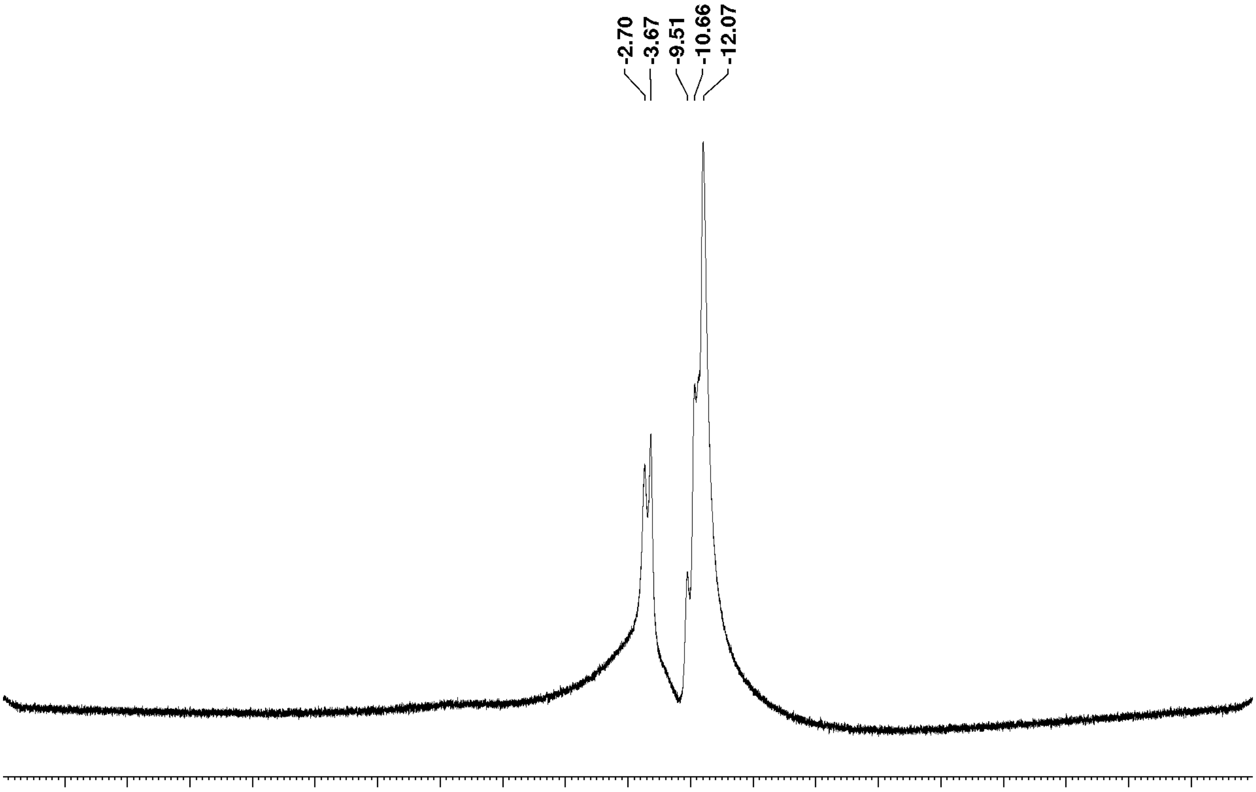

Synthesis of 1-(3-(4-methoxybenzyl)hydroxymethyl)-2-(hydroxymethyl)-4-ethynyl-1,2-dicarba-*closo*-dodecaborane (11)

^1H NMR (500 MHz; $CDCl_3$)

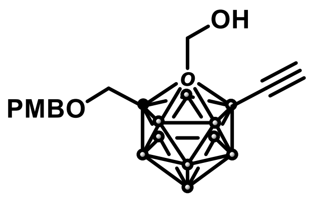

11

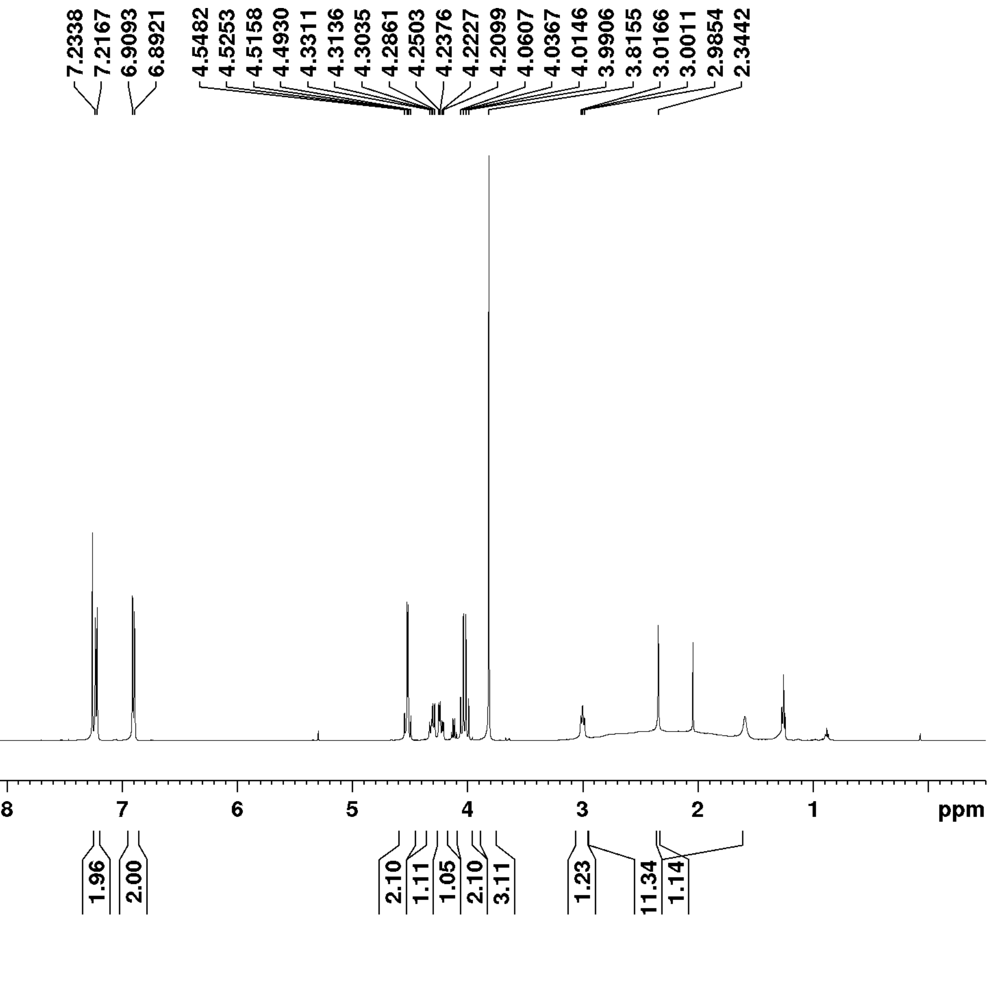

^{13}C NMR (125 MHz; $CDCl_3$)

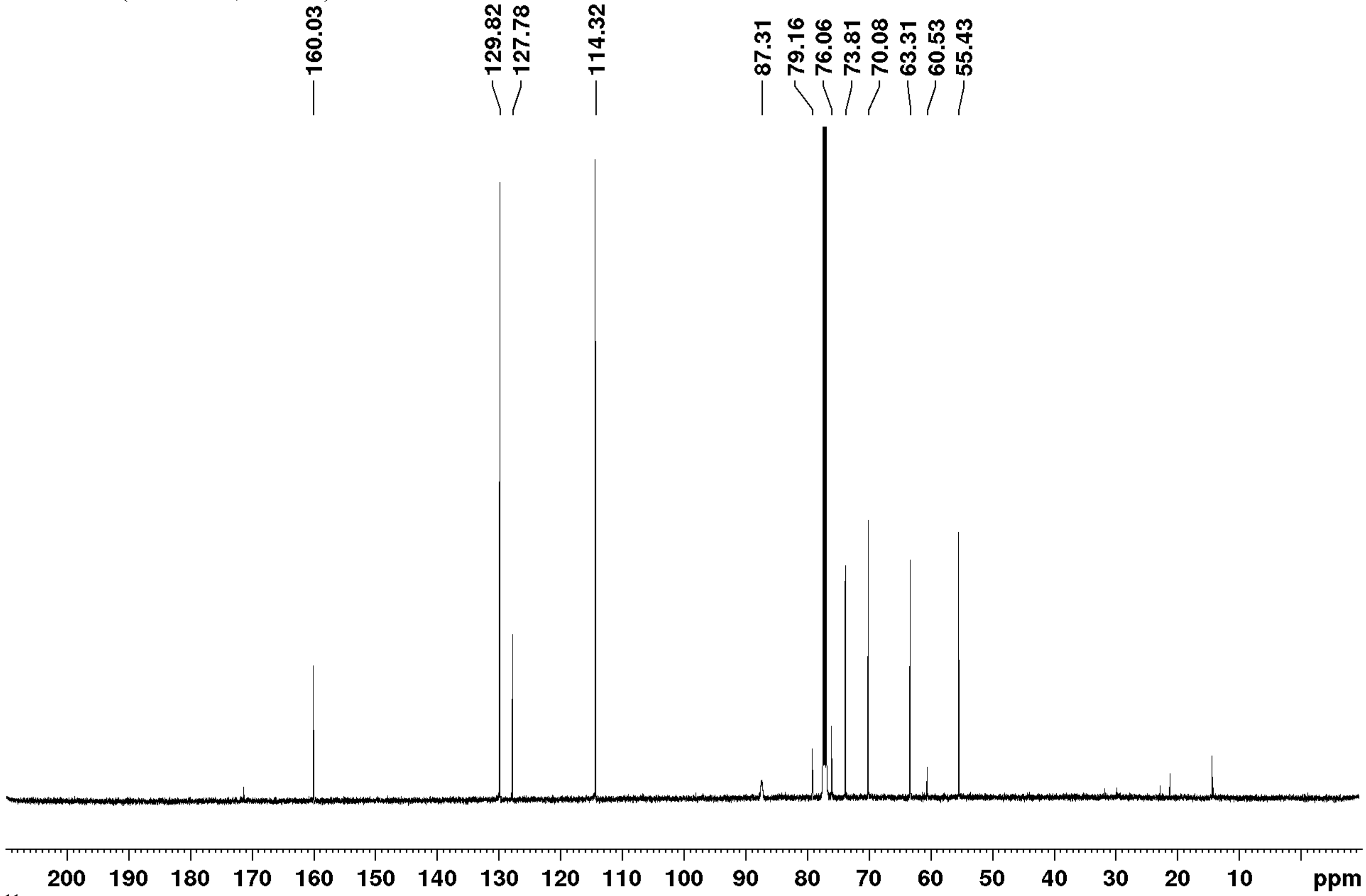

^{11}B NMR (160 MHz; $CDCl_3$)

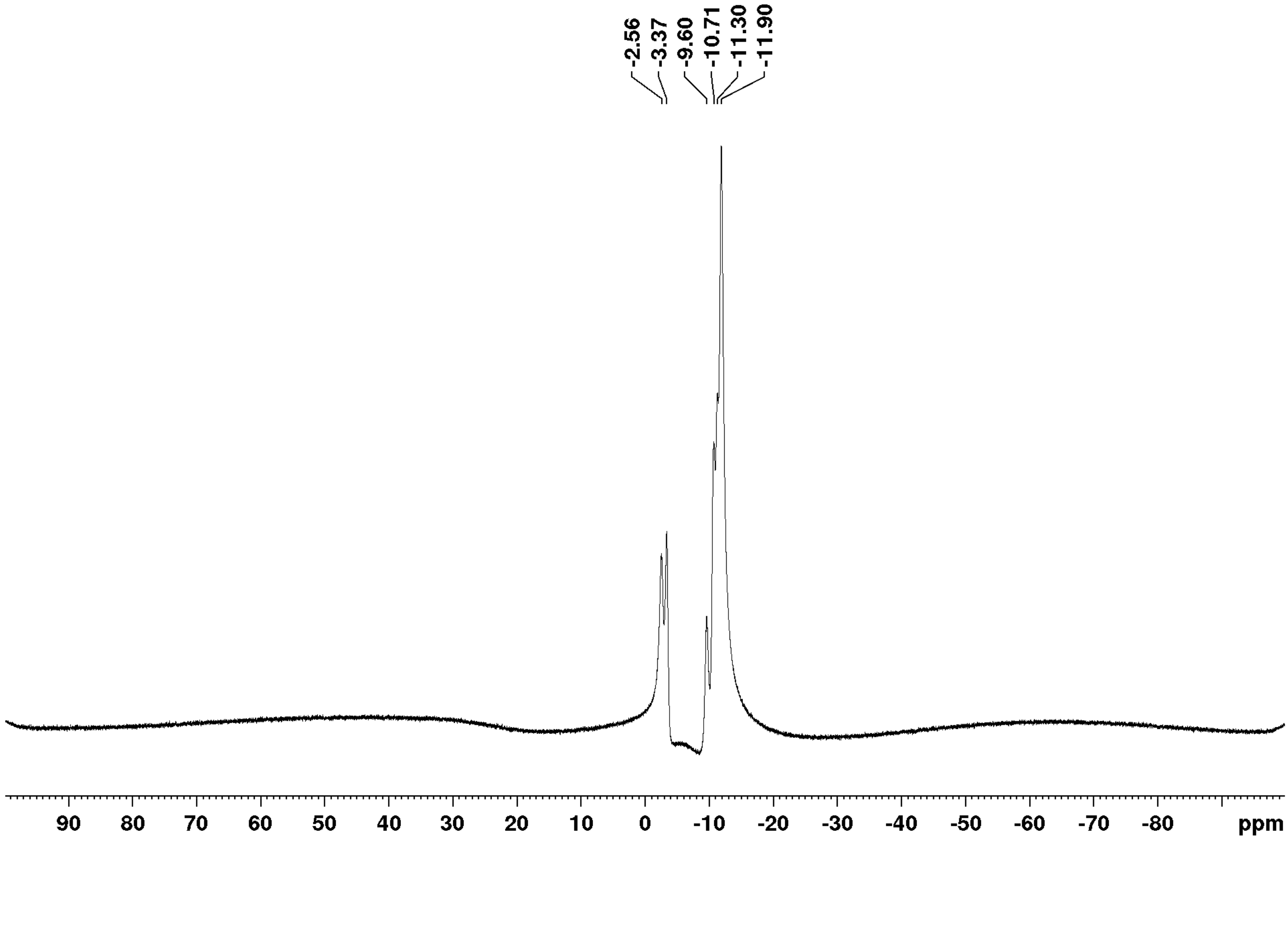

1-(3-(4-methoxybenzyl)hydroxymethyl)-2-((*tert*-butyl)dimethylsilyl)-hydroxymethyl)-4-ethynyl-1,2-dicarba-*closo*-dodecaborane (S4)

^{1}H NMR (500 MHz; $CDCl_3$)

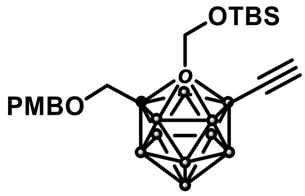

S4

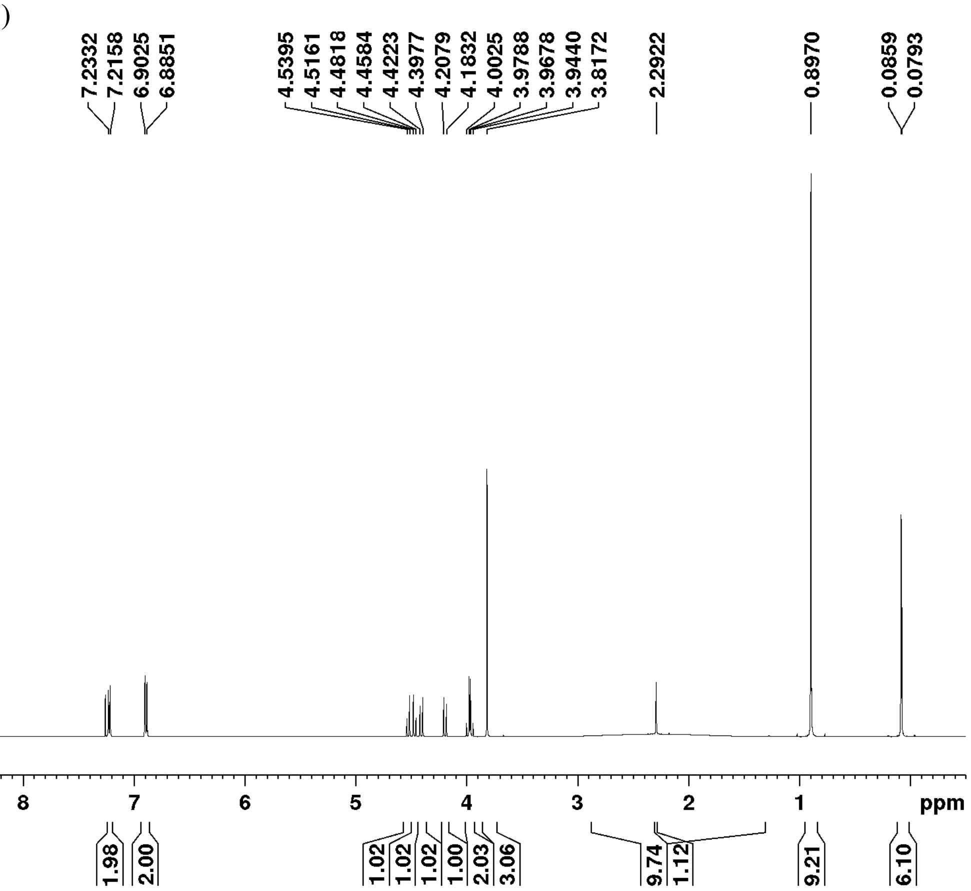

^{13}C NMR (125 MHz; $CDCl_3$)

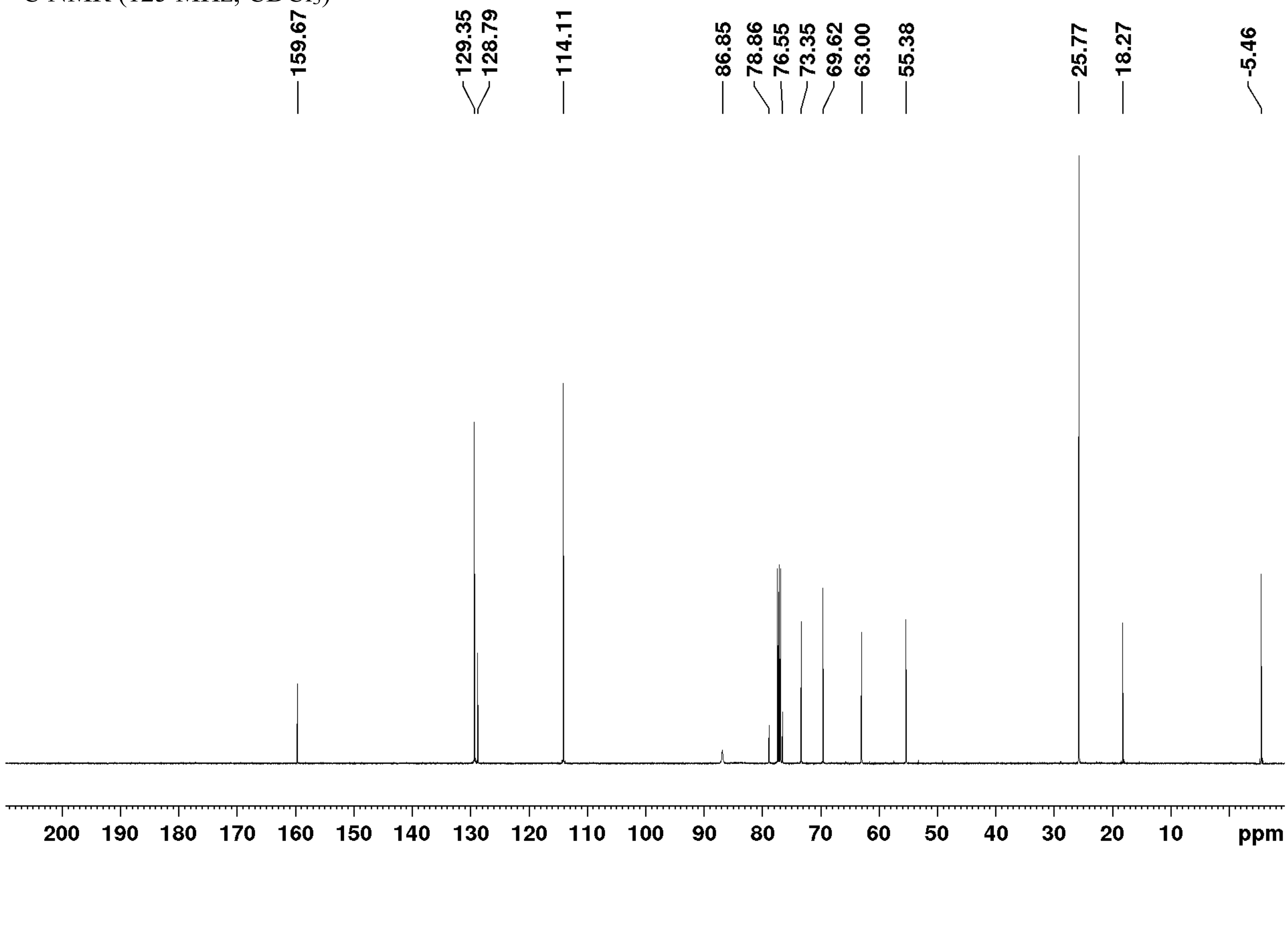

^{11}B NMR (160 MHz; $CDCl_3$)

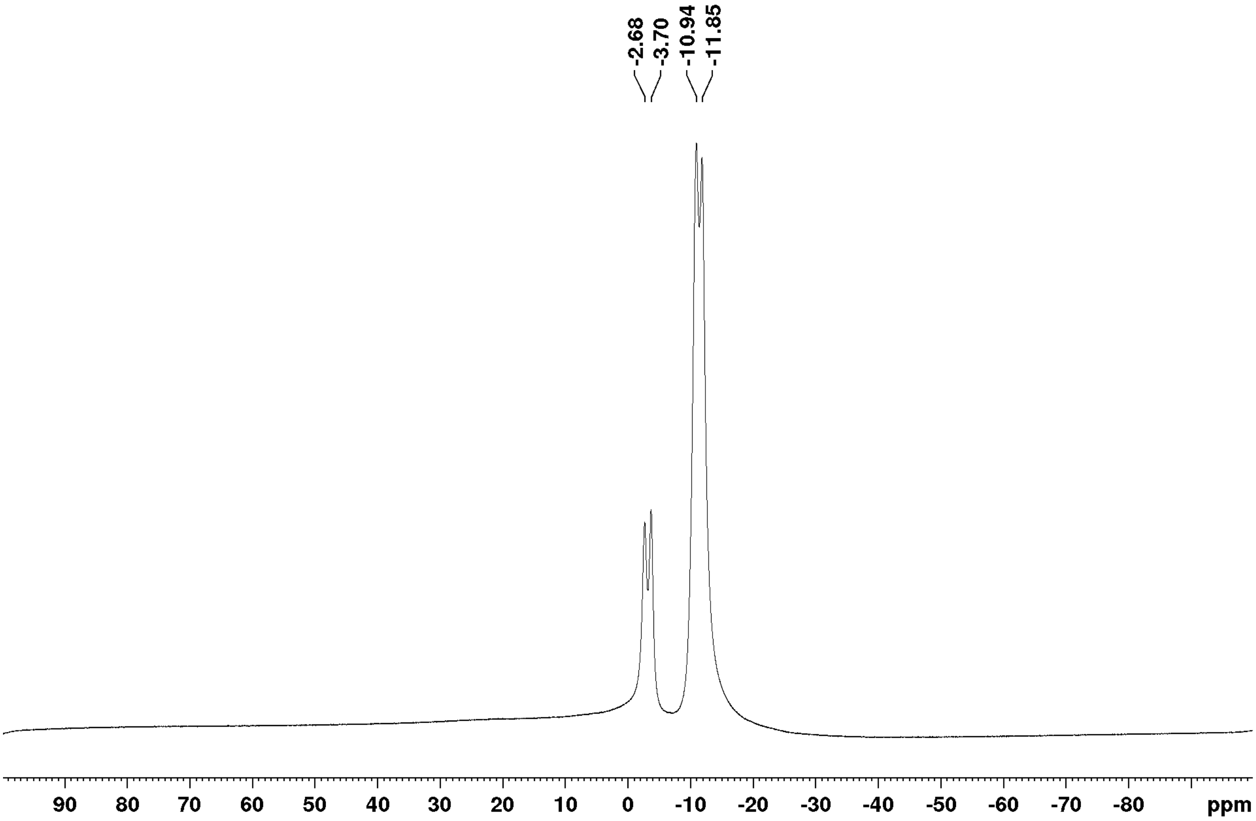

1-(3-(4-methoxybenzyl)hydroxymethyl)-2-((*tert*-butyl)dimethylsilyl)-hydroxymethyl)-4-(3-oxoprop-1-yn-1-yl)-1,2-dicarba-*closo*-dodecaborane (12)

^{1}H NMR (500 MHz; $CDCl_3$)

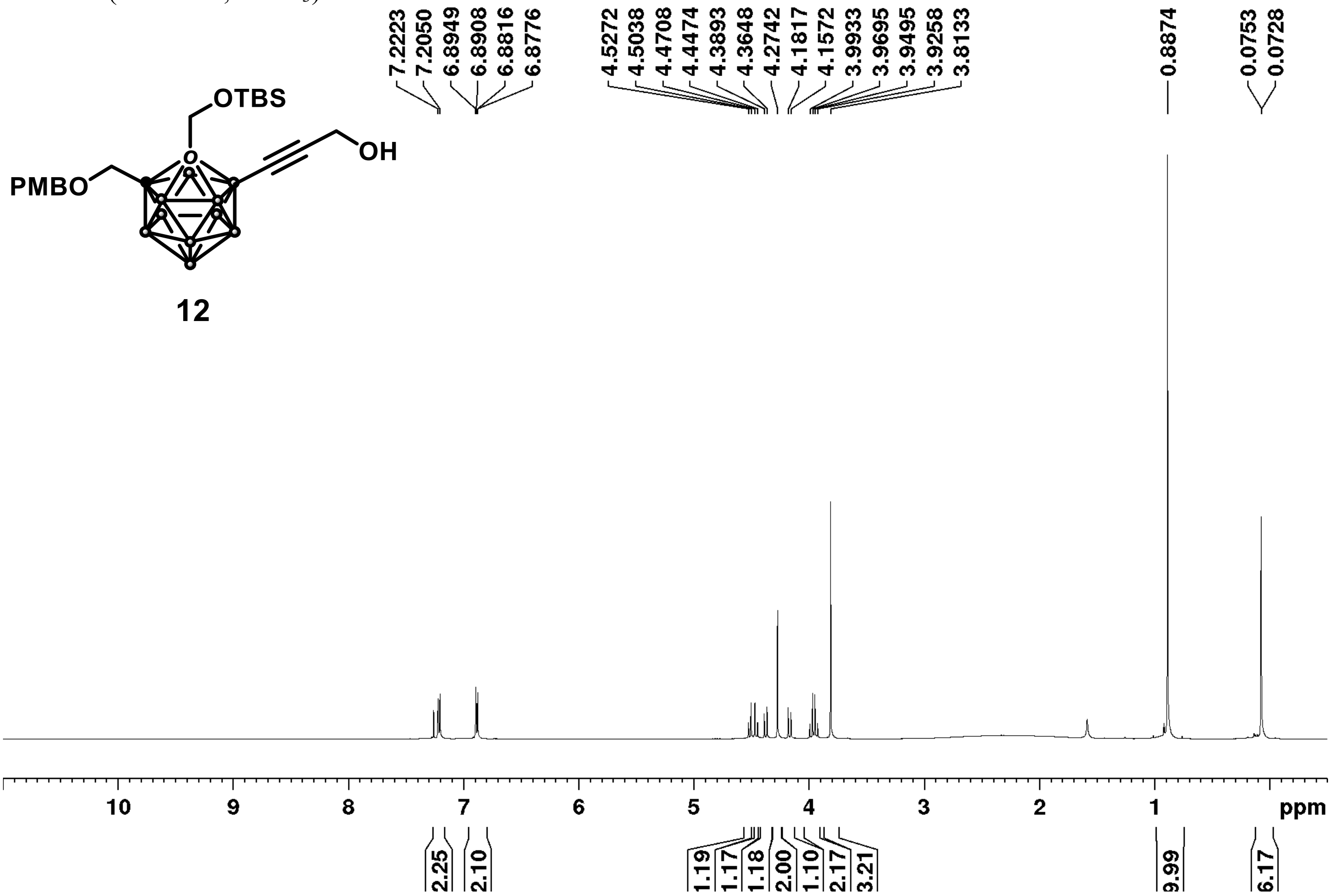

^{13}C NMR (125 MHz; $CDCl_3$)

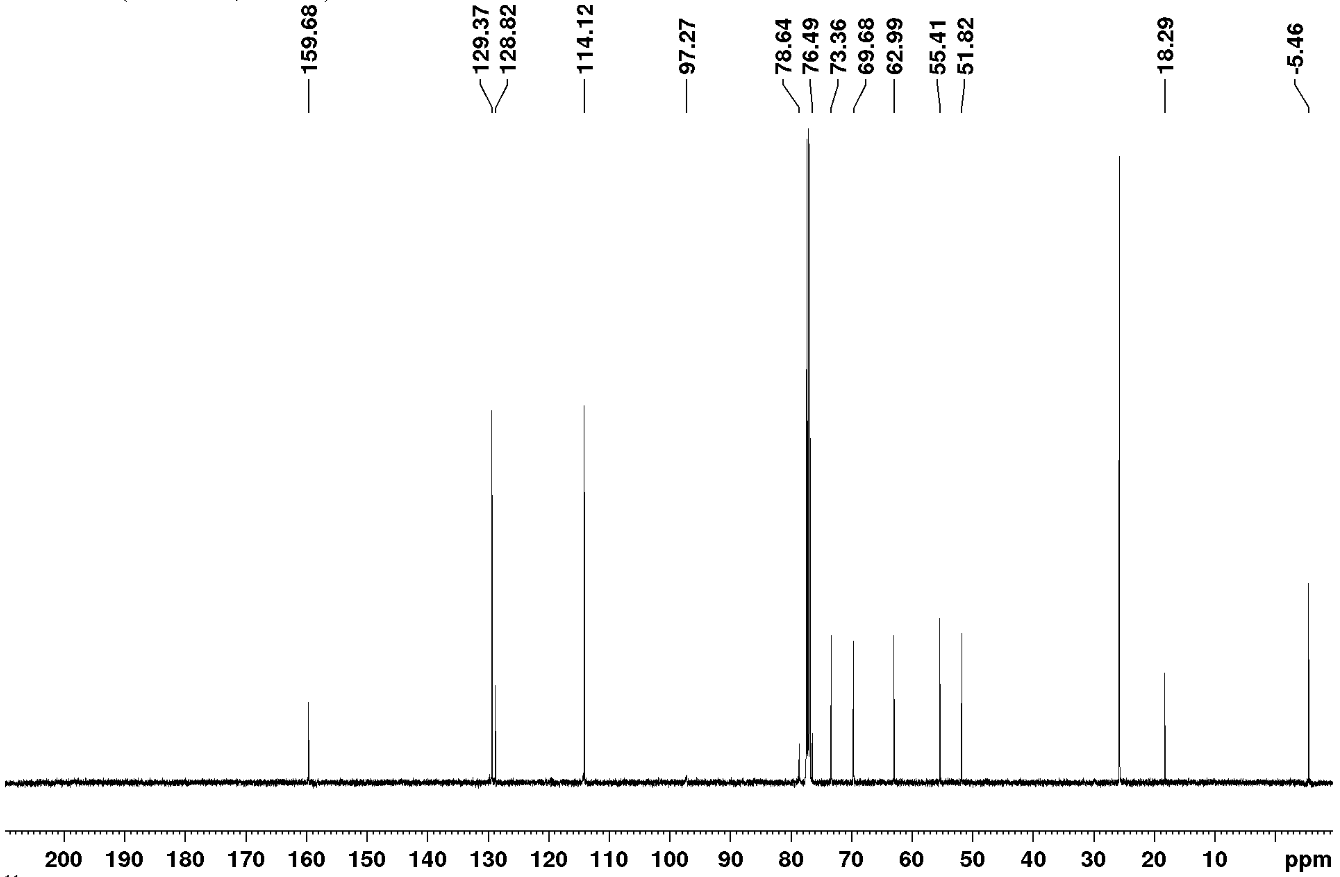

^{11}B NMR (160 MHz; $CDCl_3$)

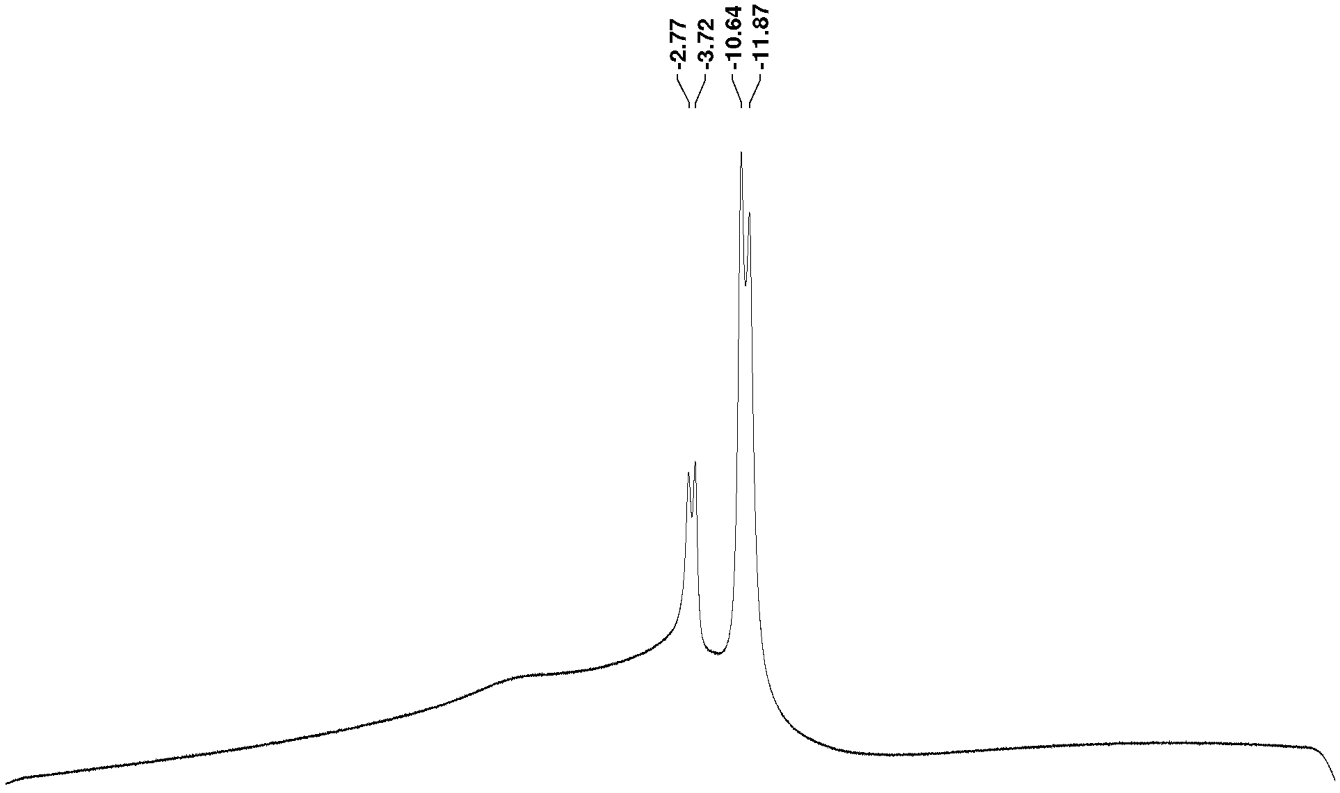

1-(3-(4-methoxybenzyl)hydroxymethyl)-2-((*tert*-butyl)dimethylsilyl)-hydroxymethyl)-4-(2-phenylacetoxy)prop-1-yn-1-yl)-1,2-dicarba-*closo*-dodecaborane (13a)

^{1}H NMR (500 MHz; $CDCl_3$)

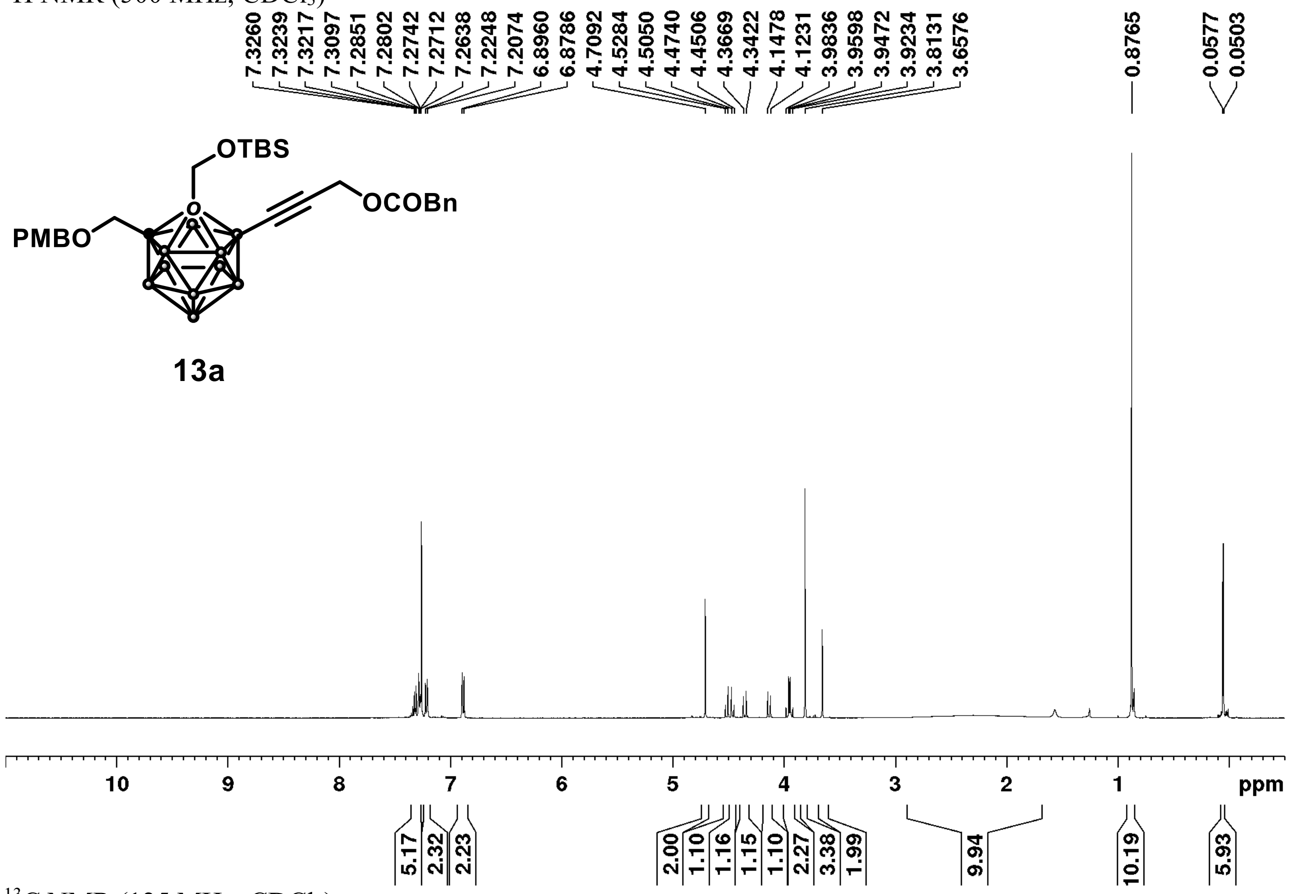

13a

^{13}C NMR (125 MHz; $CDCl_3$)

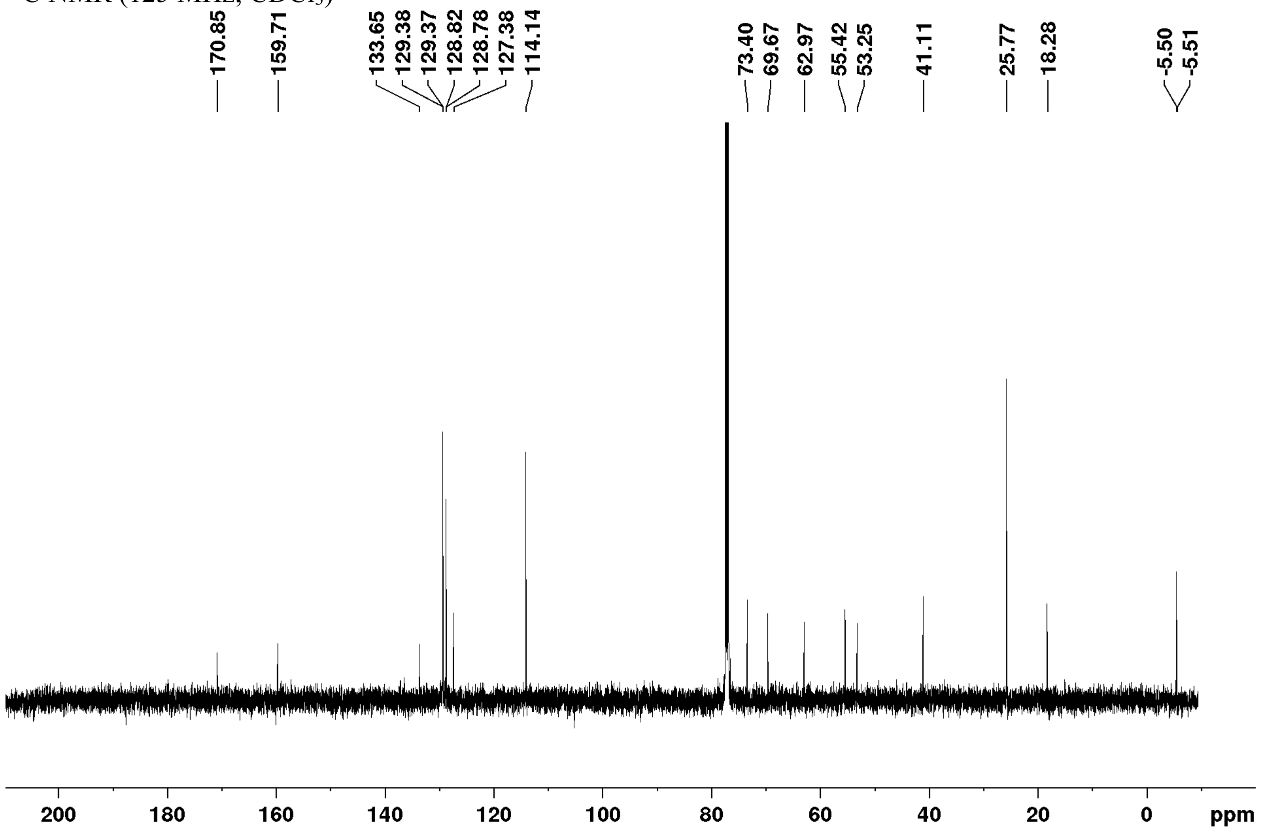

^{11}B NMR (160 MHz; $CDCl_3$)

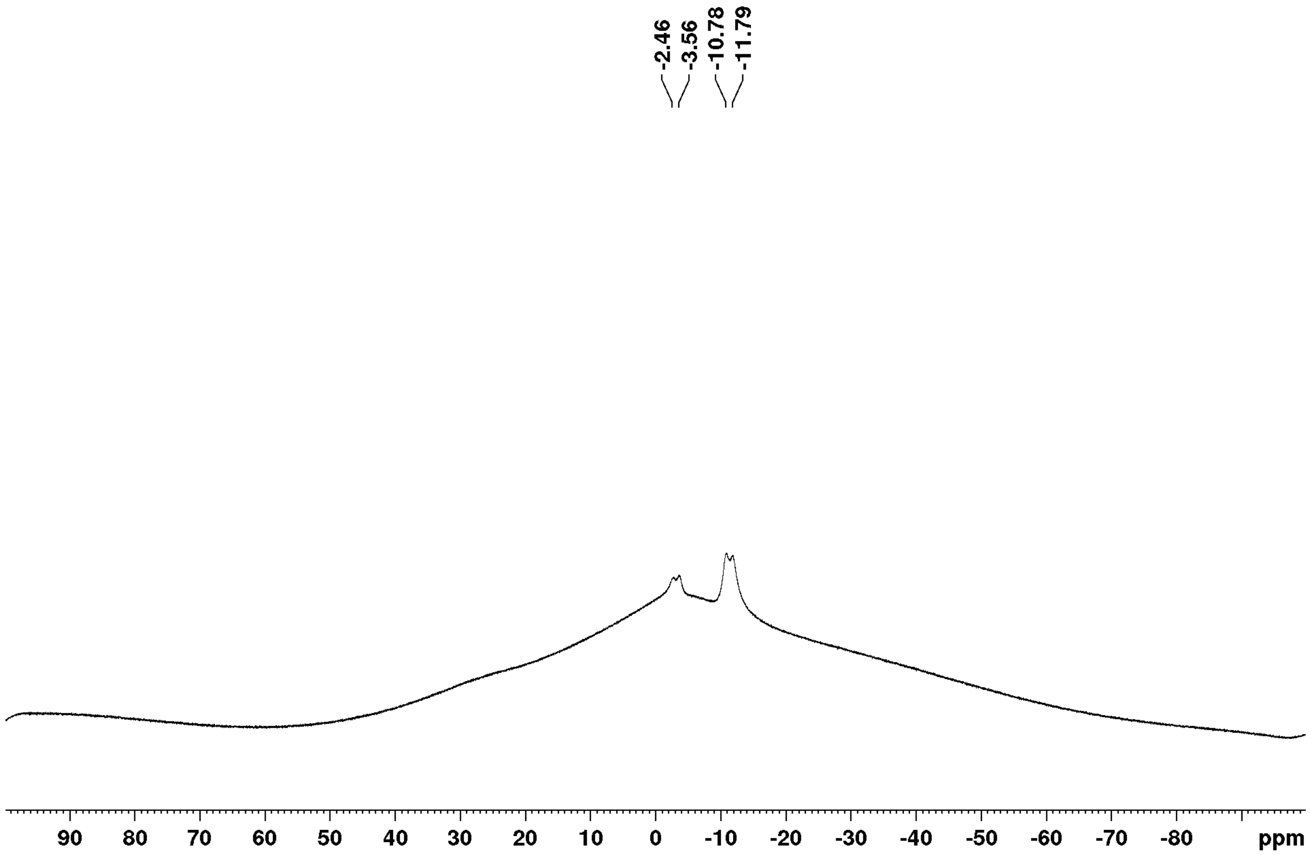

1-(3-(4-methoxybenzyl)hydroxymethyl)-2-((*tert*-butyl)dimethylsilyl)-hydroxymethyl)-4-(3-((3-methylbutanoyl)oxy)prop-1-yn-1-yl)-1,2-dicarba-*closo*-dodecaborane (13b)

^{1}H NMR (400 MHz; $CDCl_3$)

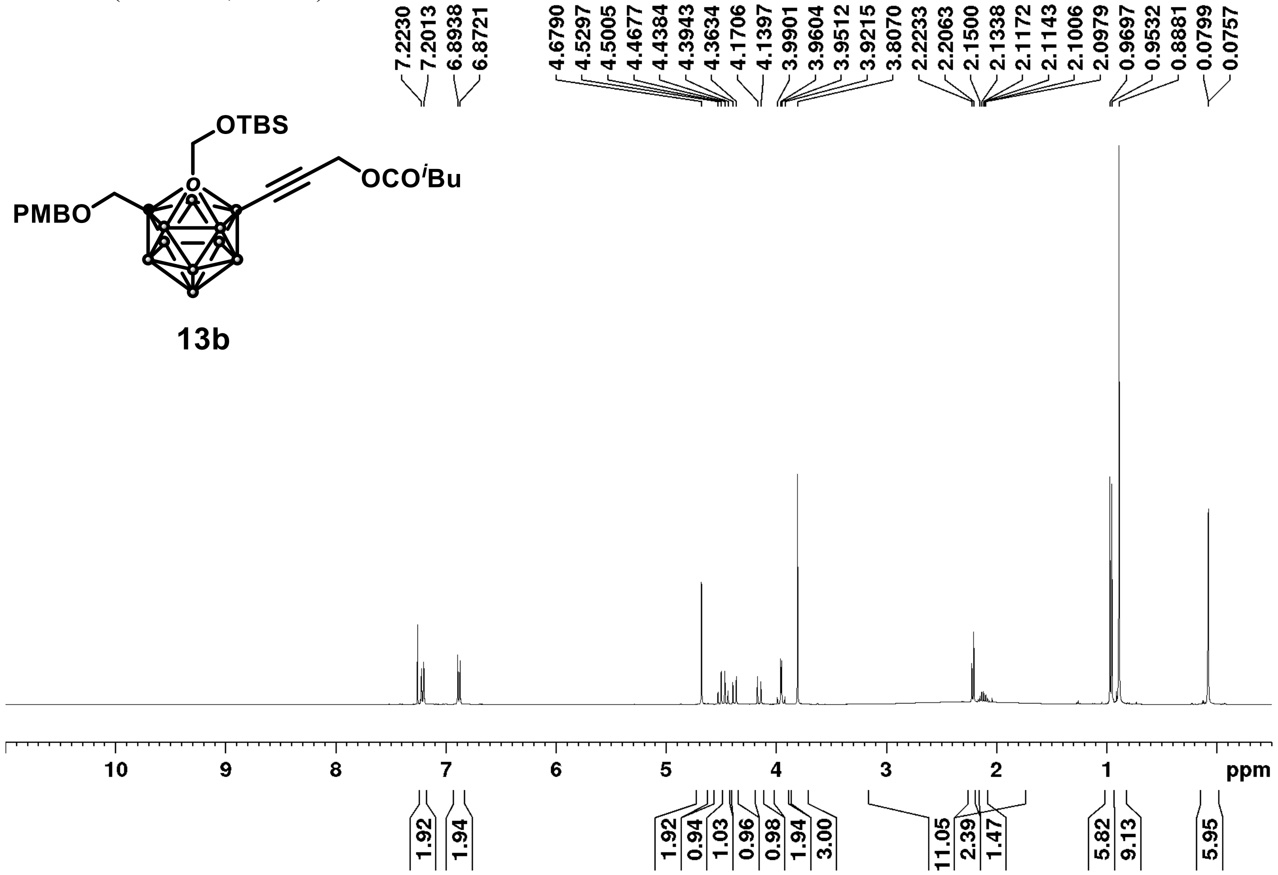

^{13}C NMR (100 MHz; $CDCl_3$)

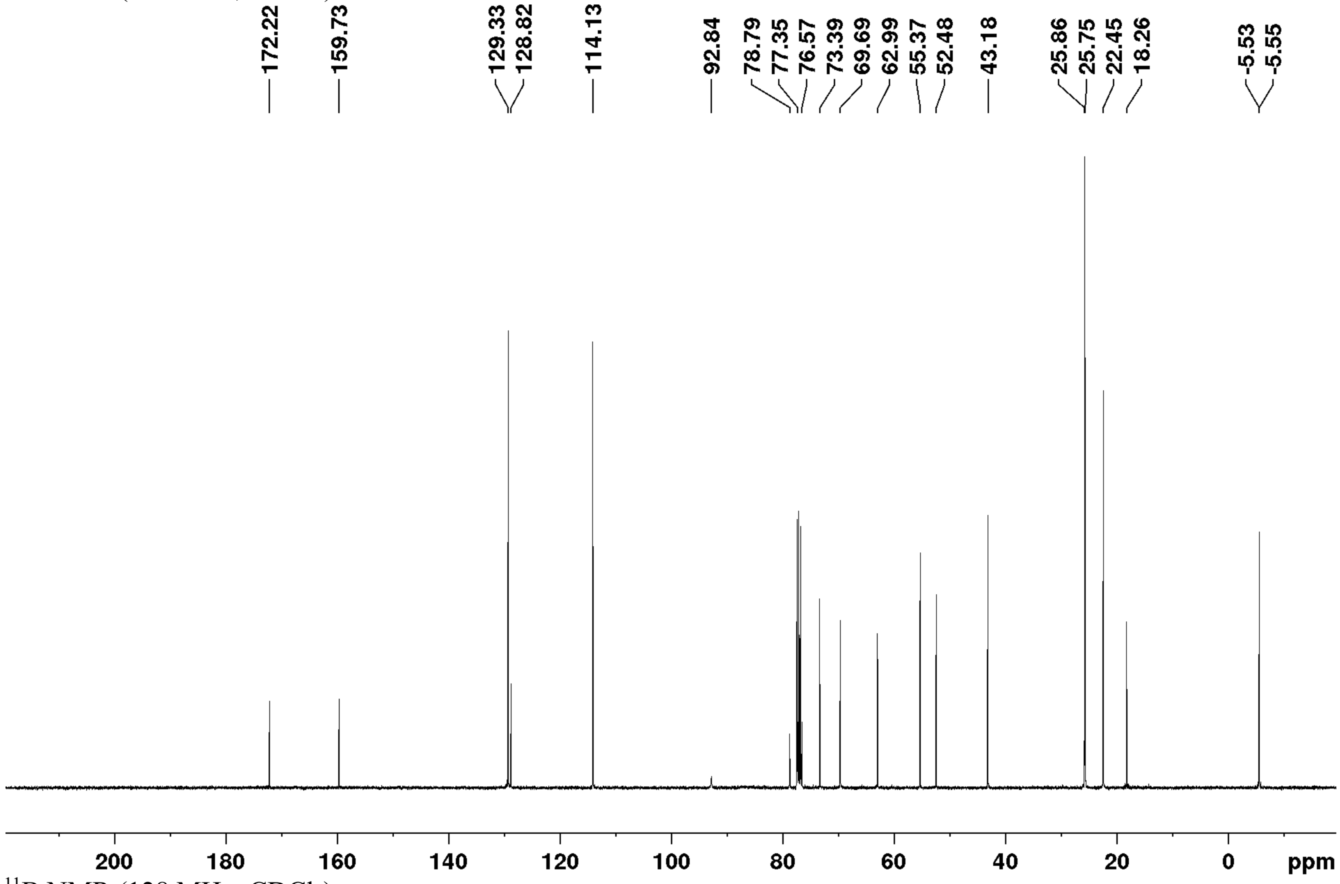

^{11}B NMR (128 MHz; $CDCl_3$)

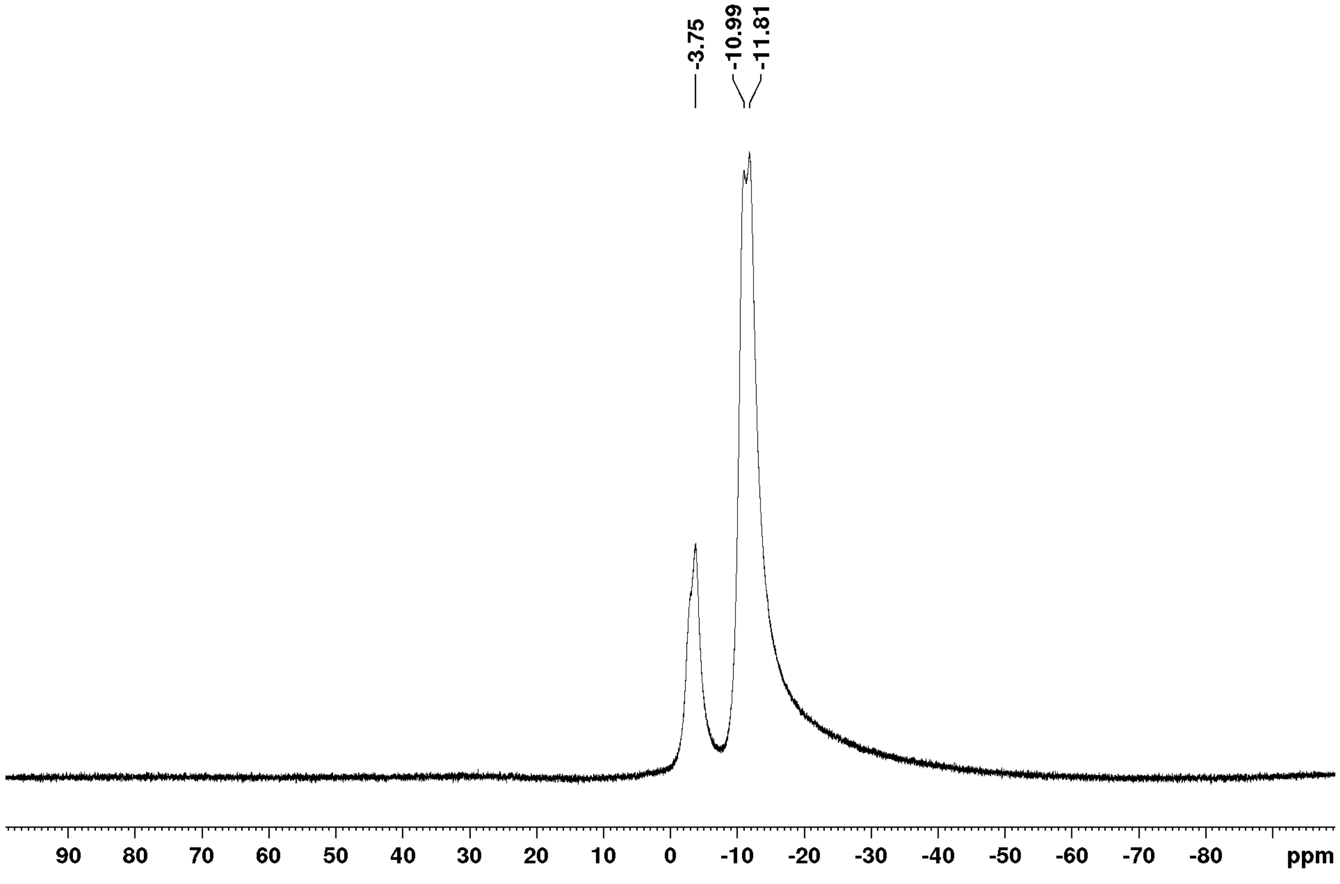

1,2-bis(hydroxymethyl)-4-ethynyl-1,2-dicarba-*closo*-dodecaborane (14)

^{1}H NMR (500 MHz; $CDCl_3$)

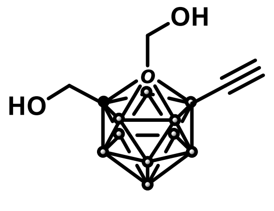

14

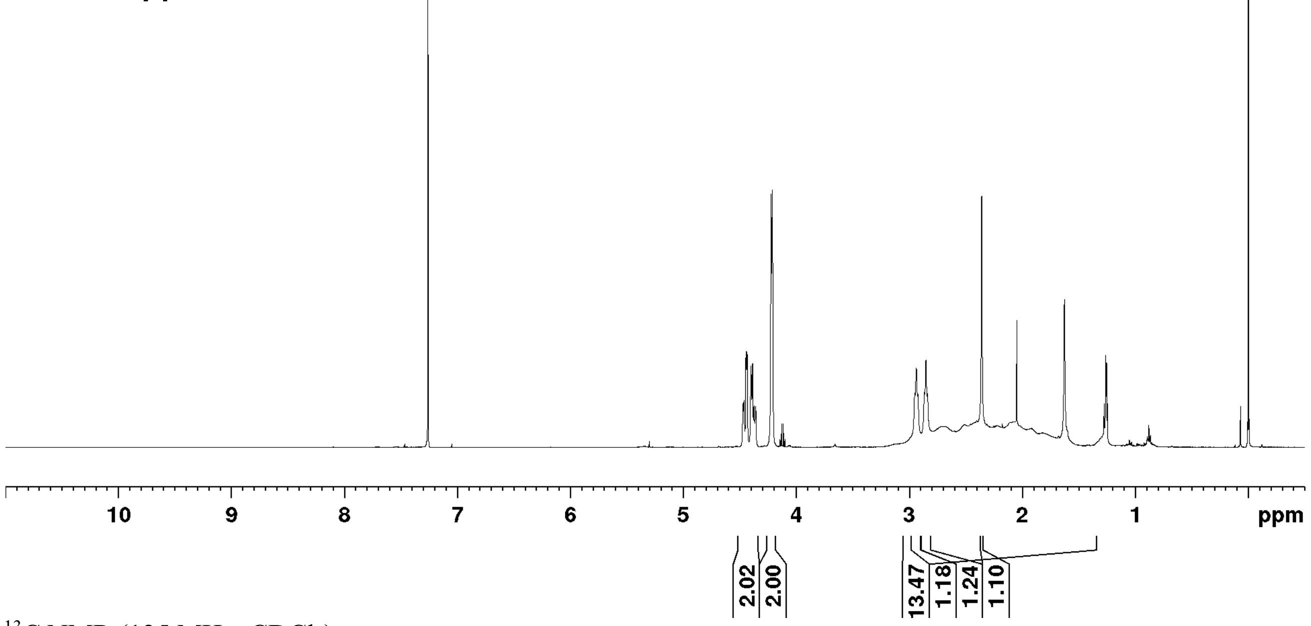

^{13}C NMR (125 MHz; $CDCl_3$)

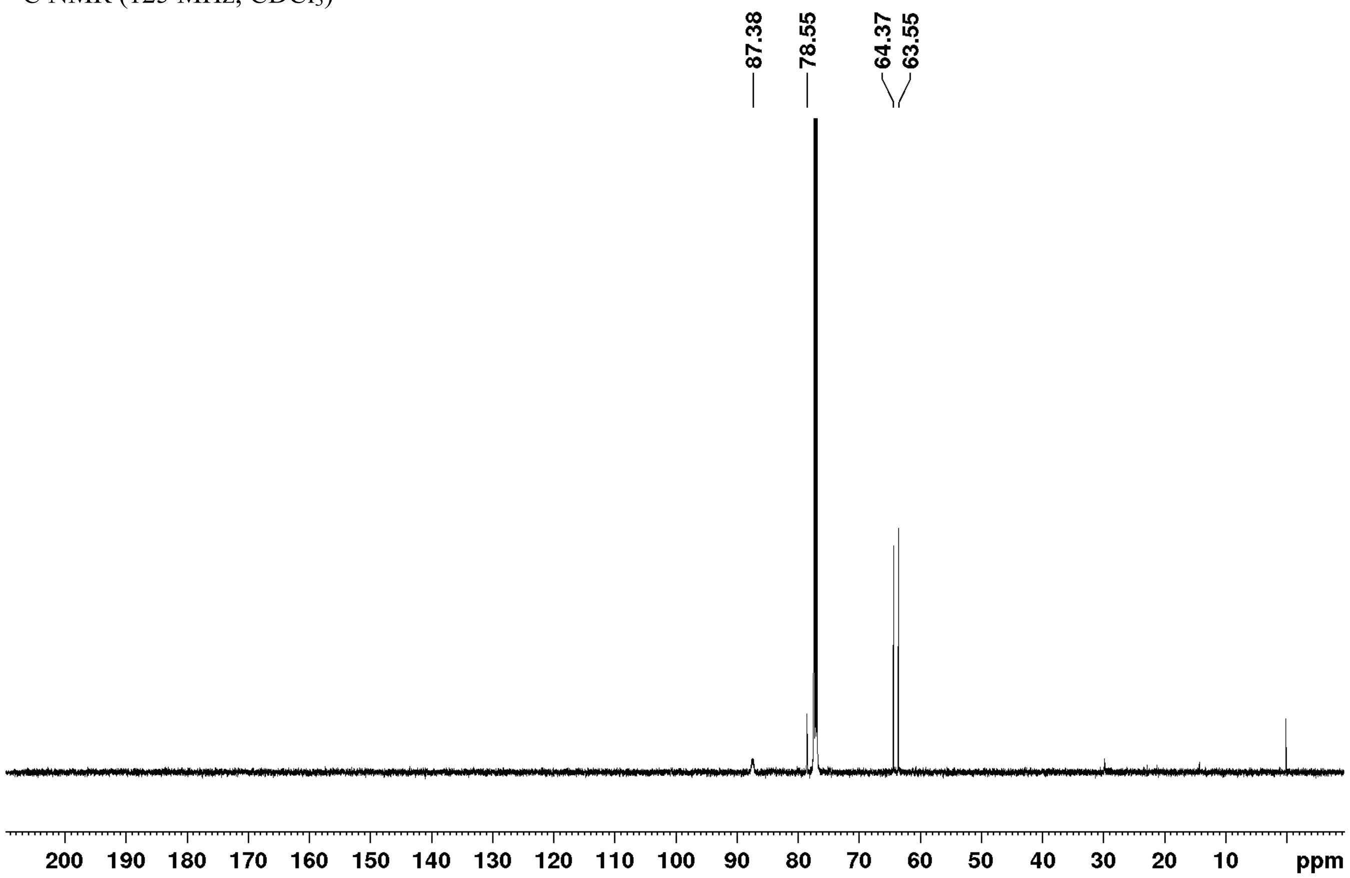

^{11}B NMR (160 MHz; CDCl$_3$)

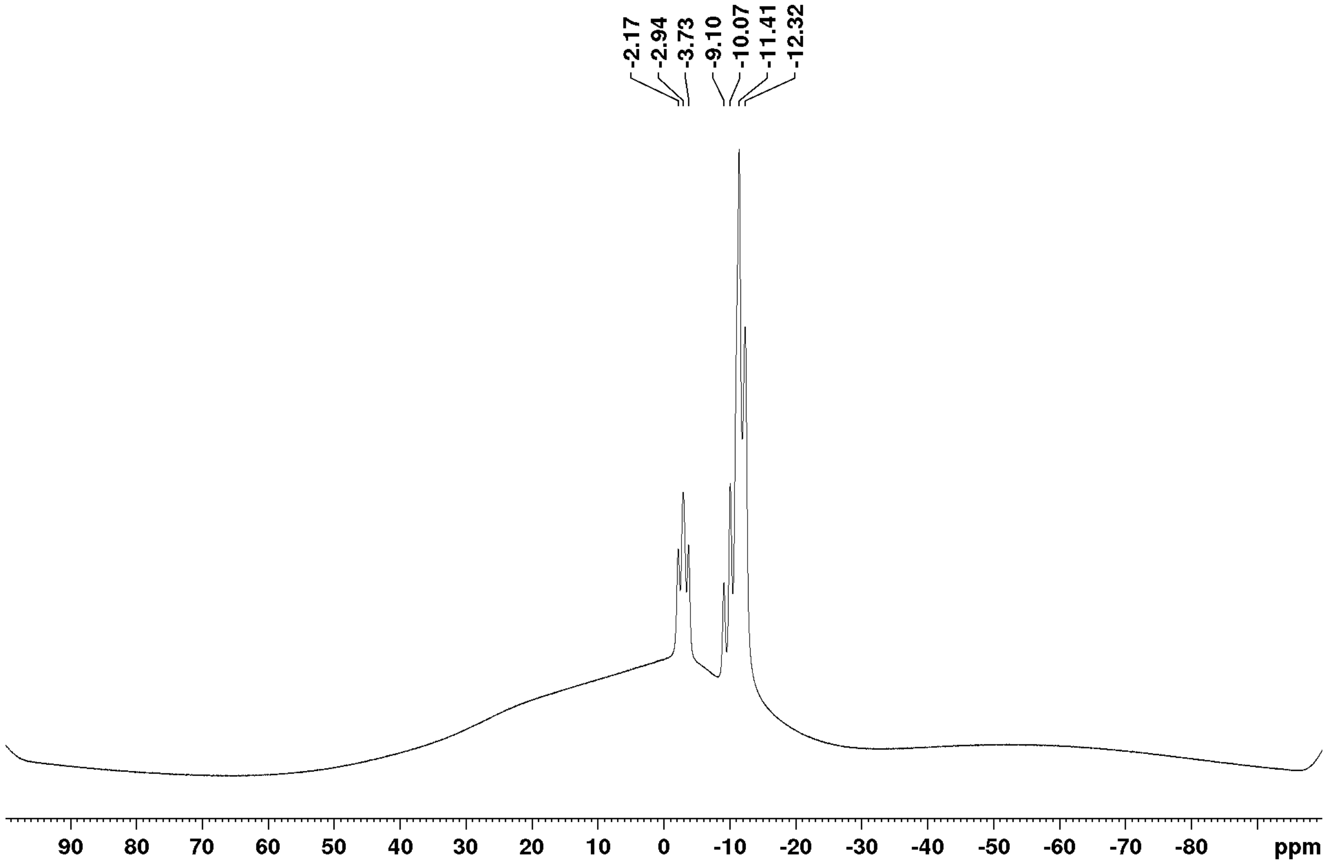

1-(2-phenylacetoxymethyl)-2-((*tert*-butyl)dimethylsilyl)-hydroxymethyl)-4-(2-phenylacetoxy)prop-1-yn-1-yl)-1,2-dicarba-*closo*-dodecaborane (15a)

^{1}H NMR (500 MHz; CDCl$_3$)

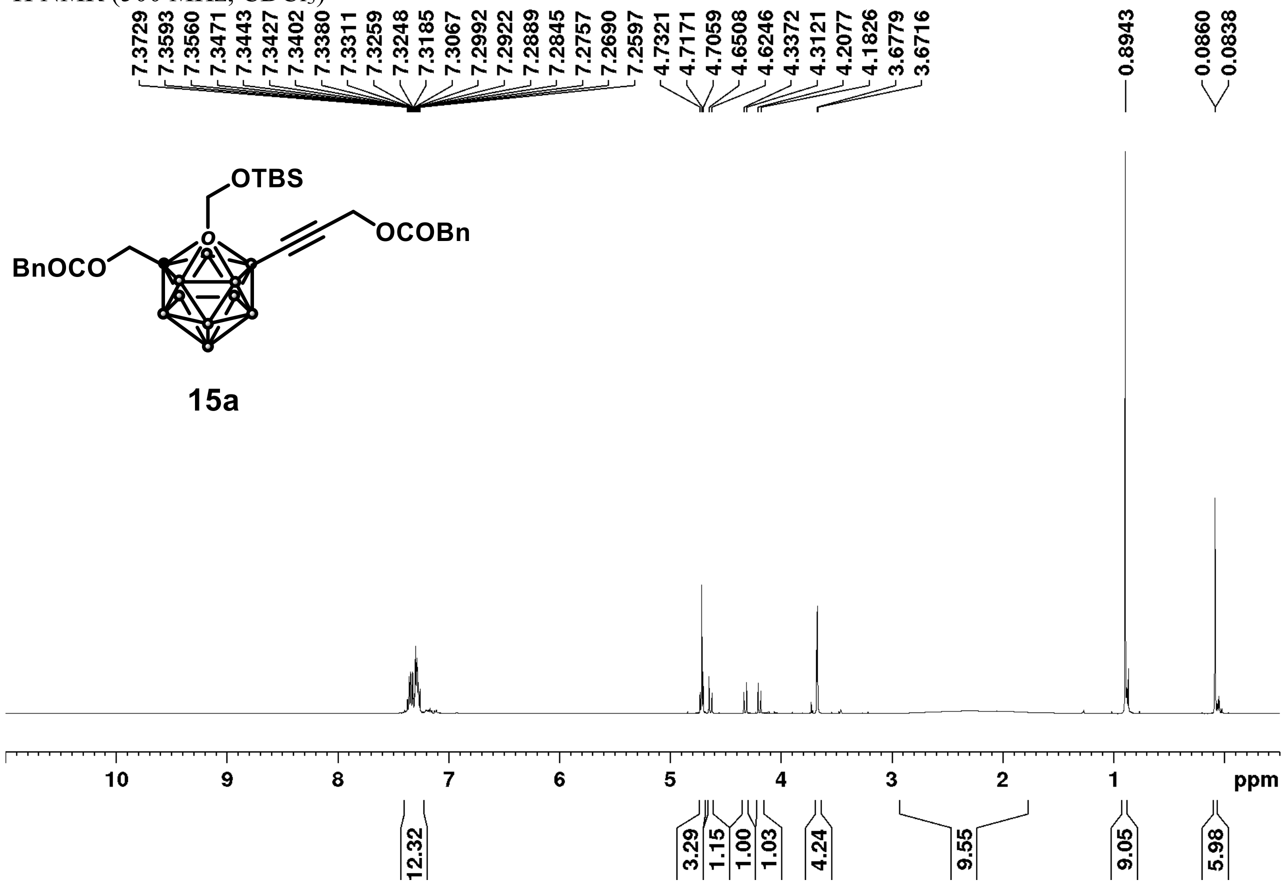

^{13}C NMR (125 MHz; $CDCl_3$)

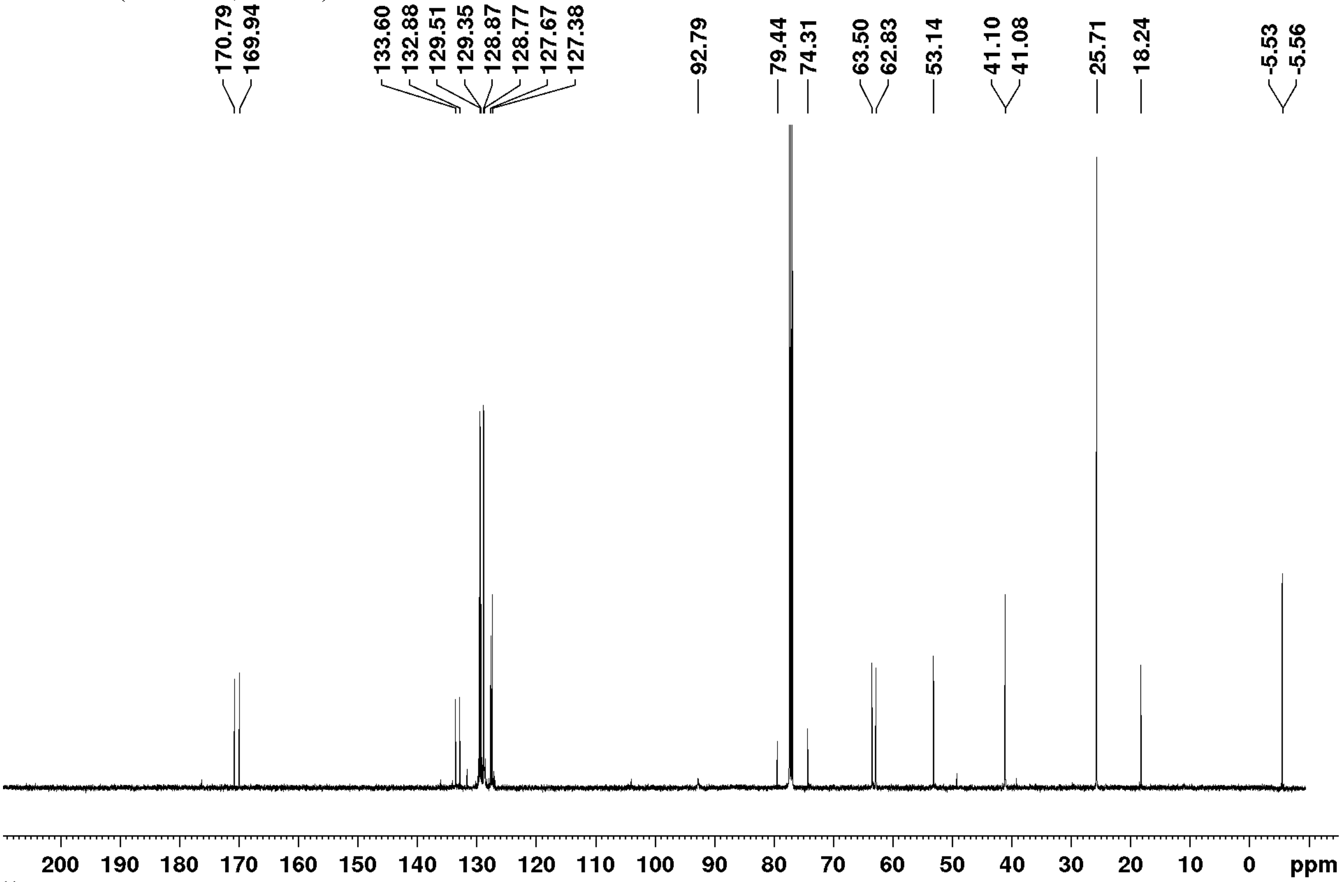

^{11}B NMR (160 MHz; $CDCl_3$)

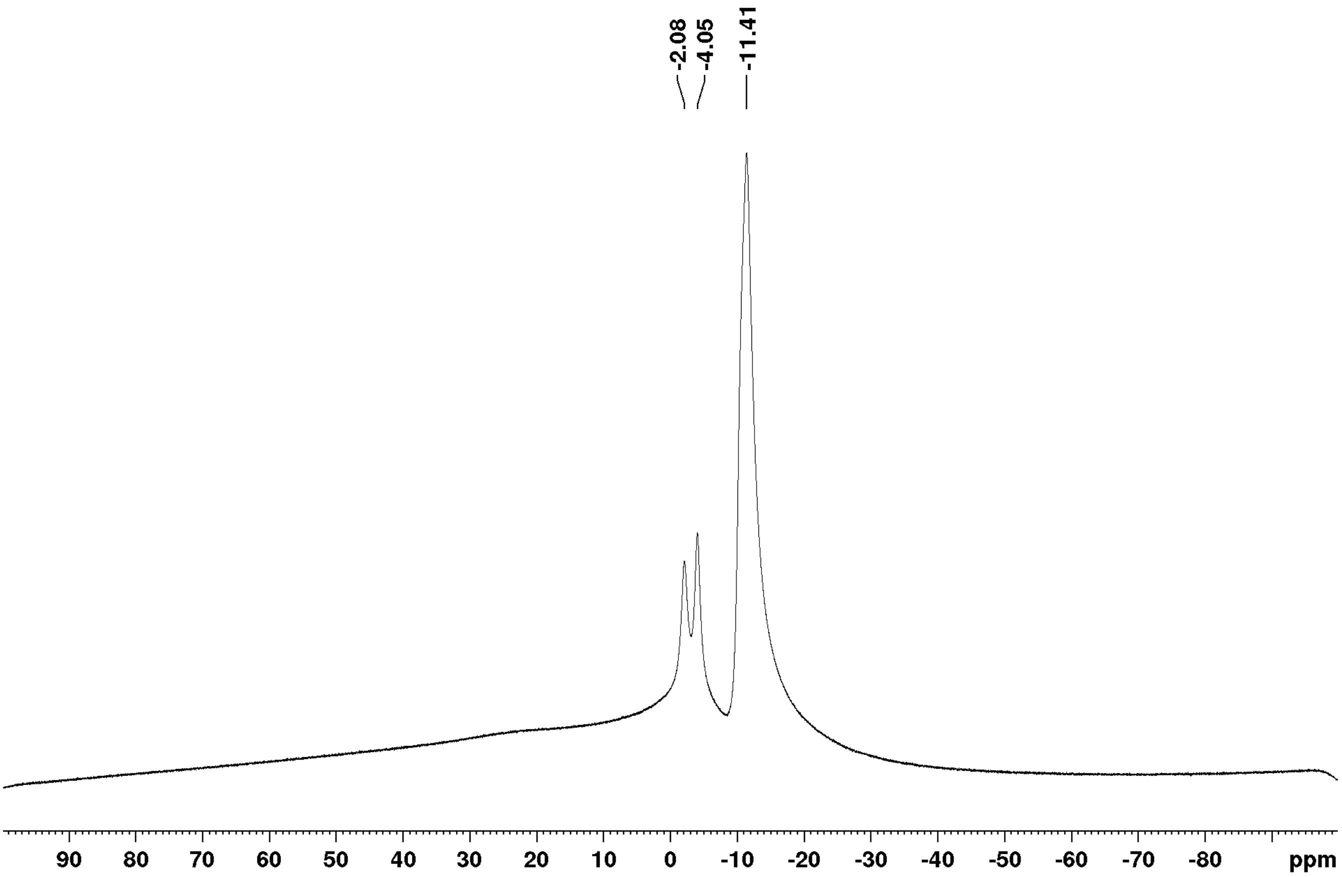

1-((3-methylbutanoyl)oxymethyl)-2-((*tert*-butyl)dimethylsilyl)-hydroxymethyl)-4-(2-phenylacetoxy)prop-1-yn-1-yl)-1,2-dicarba-*closo*-dodecaborane (15b)

^{1}H NMR (500 MHz; $CDCl_3$)

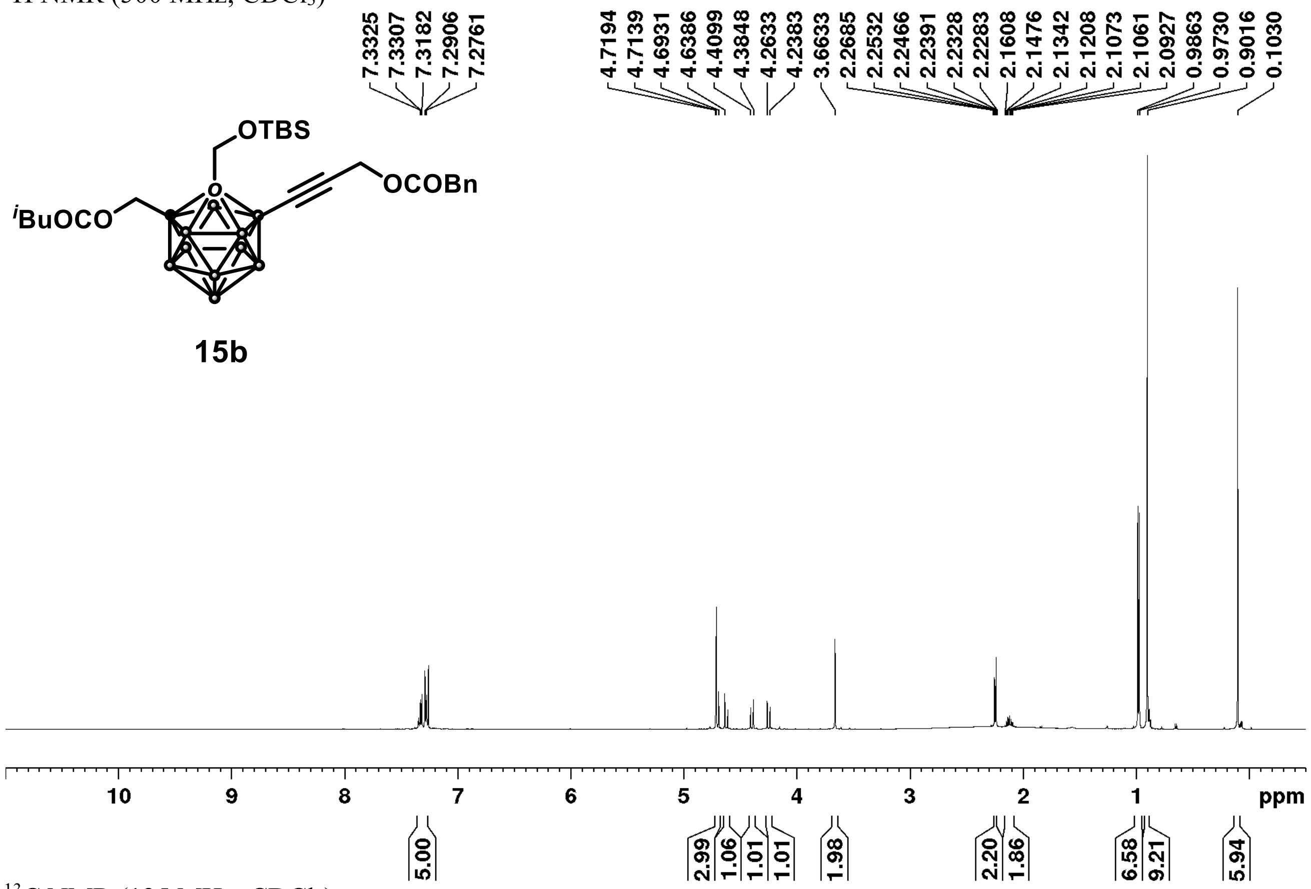

^{13}C NMR (125 MHz; $CDCl_3$)

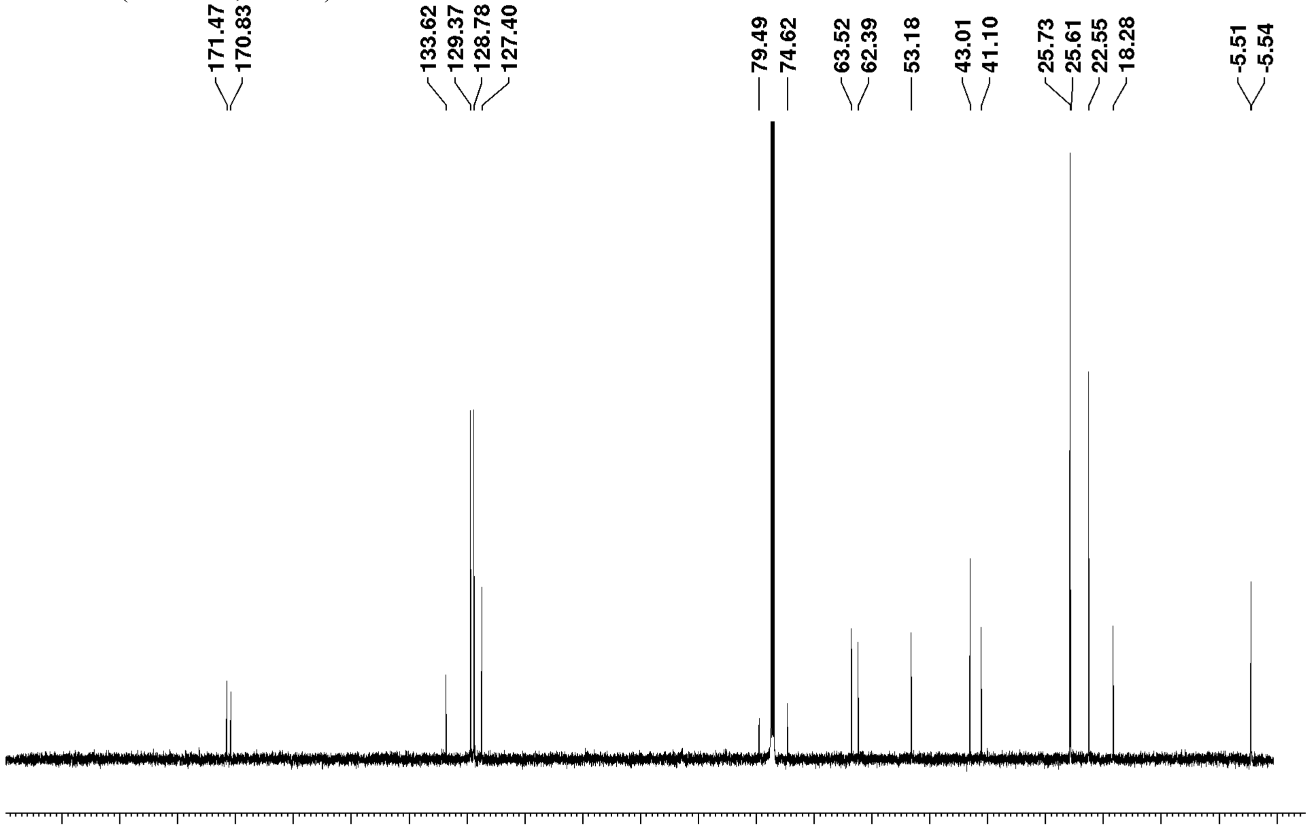

^{11}B NMR (160 MHz; CDCl$_3$)

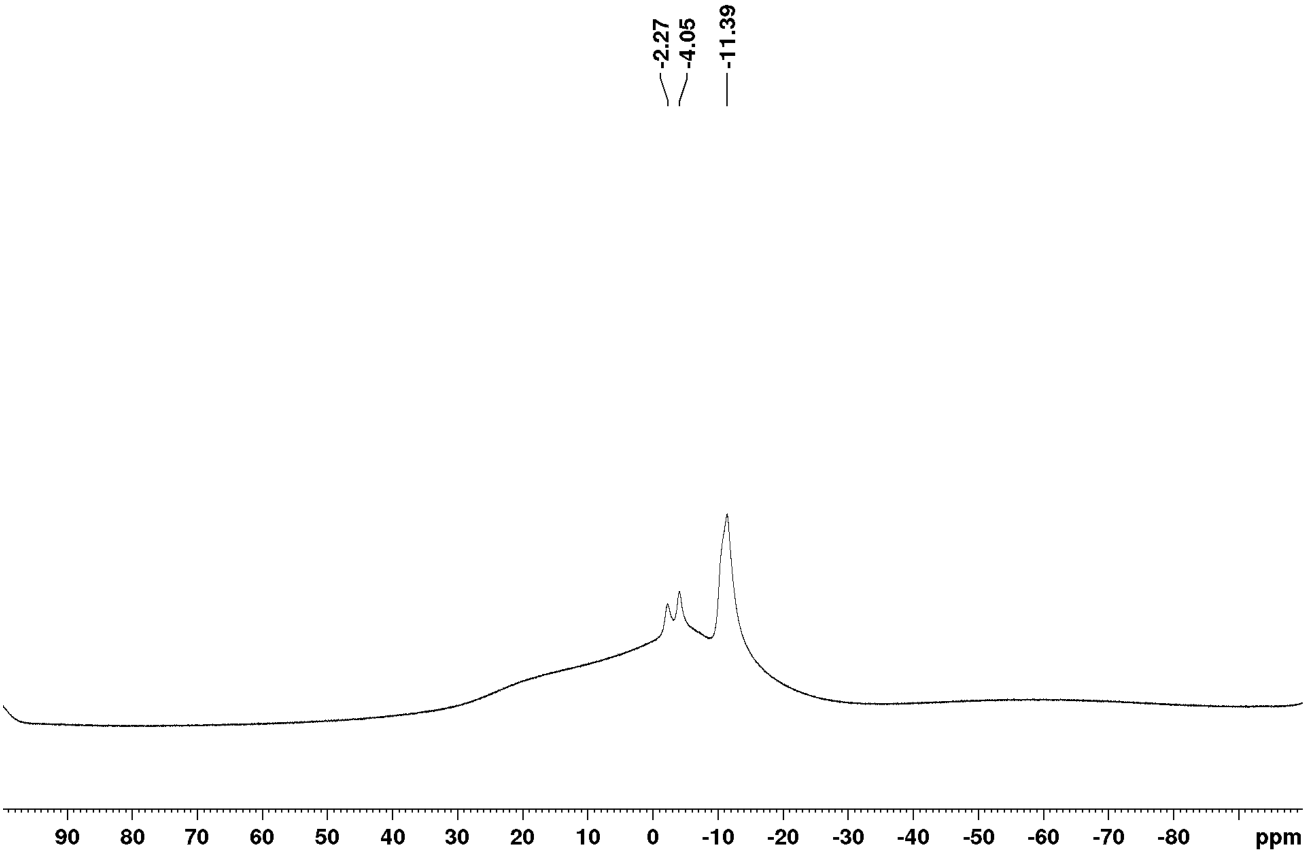

1-(2-phenylacetoxymethyl)-2-((*tert*-butyl)dimethylsilyl)-hydroxymethyl)-4-((3-methylbutanoyl)prop-1-yn-1-yl)-1,2-dicarba-*closo*-dodecaborane (15c)

^{1}H NMR (500 MHz; CDCl$_3$)

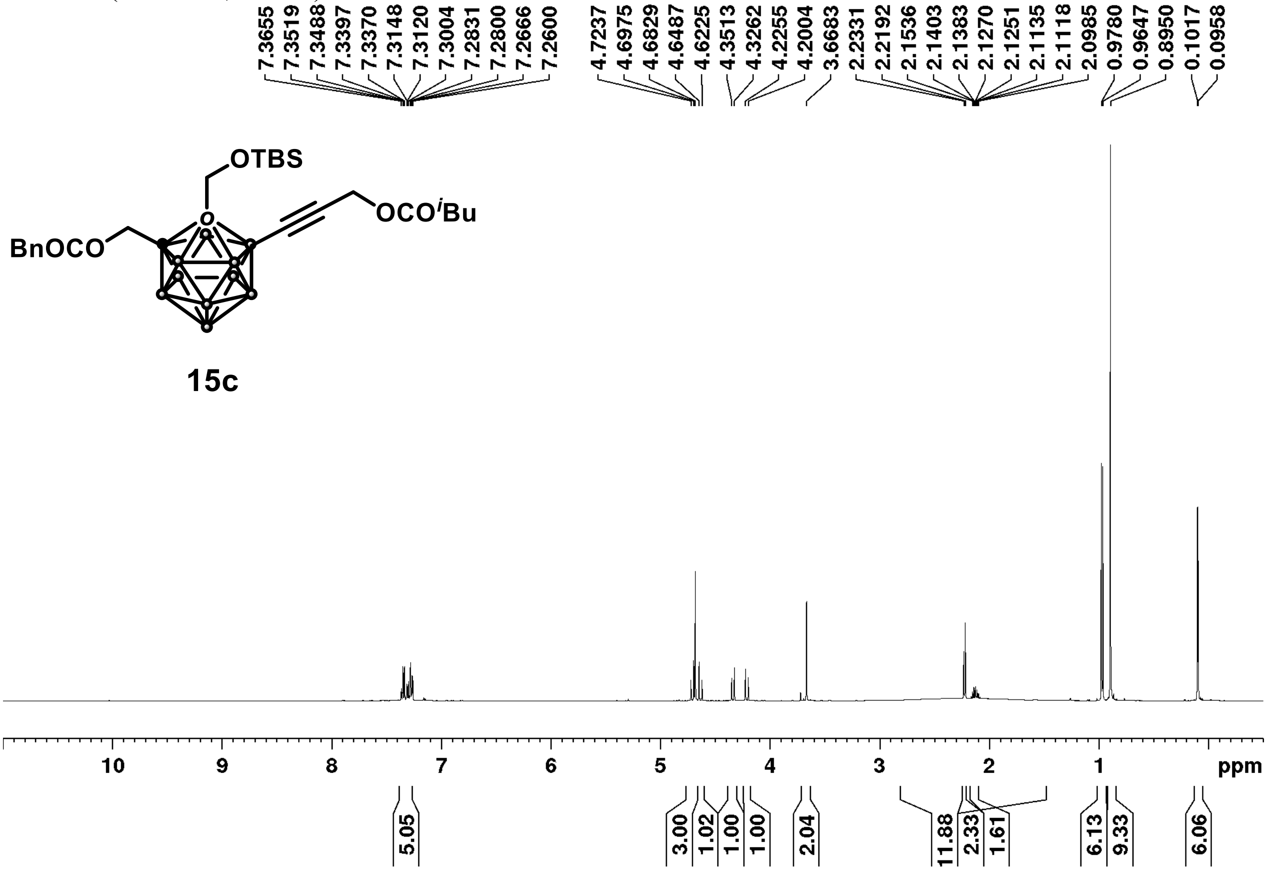

^{13}C NMR (125 MHz; $CDCl_3$)

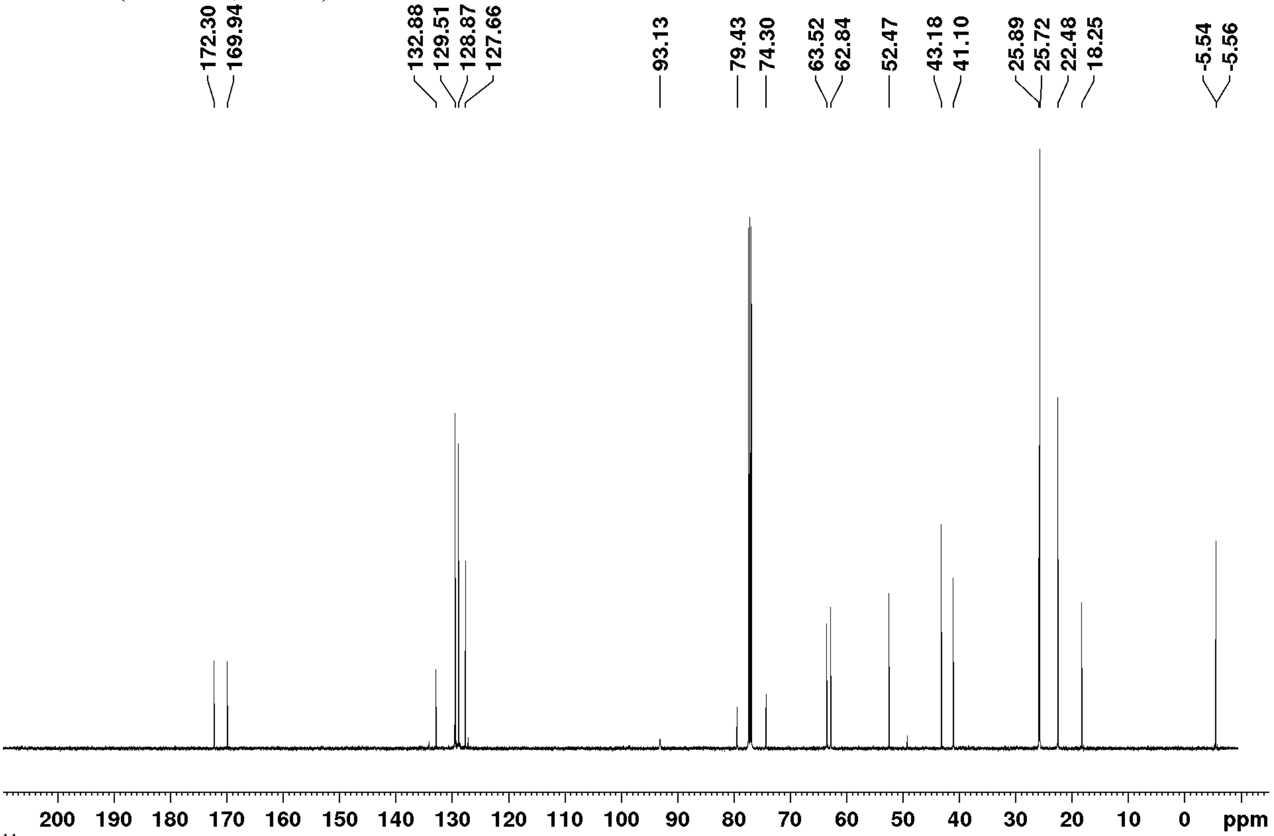

^{11}B NMR (160 MHz; $CDCl_3$)

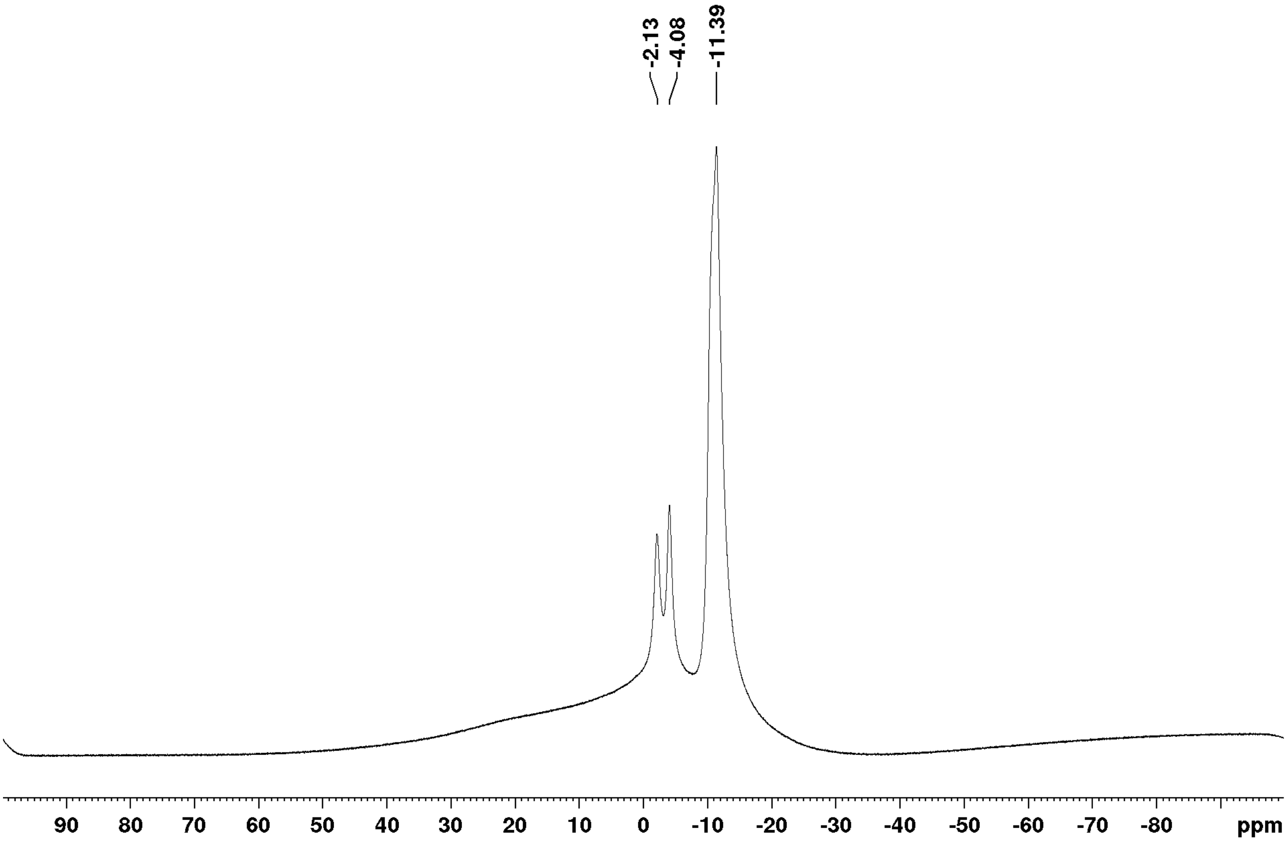

1-(3-methylbutanoyl)-2-((*tert*-butyl)dimethylsilyl)-hydroxymethyl)-4-((3-methylbutanoyl)prop-1-yn-1-yl)-1,2-dicarba-*closo*-dodecaborane (15d)

^{1}H NMR (500 MHz; $CDCl_3$)

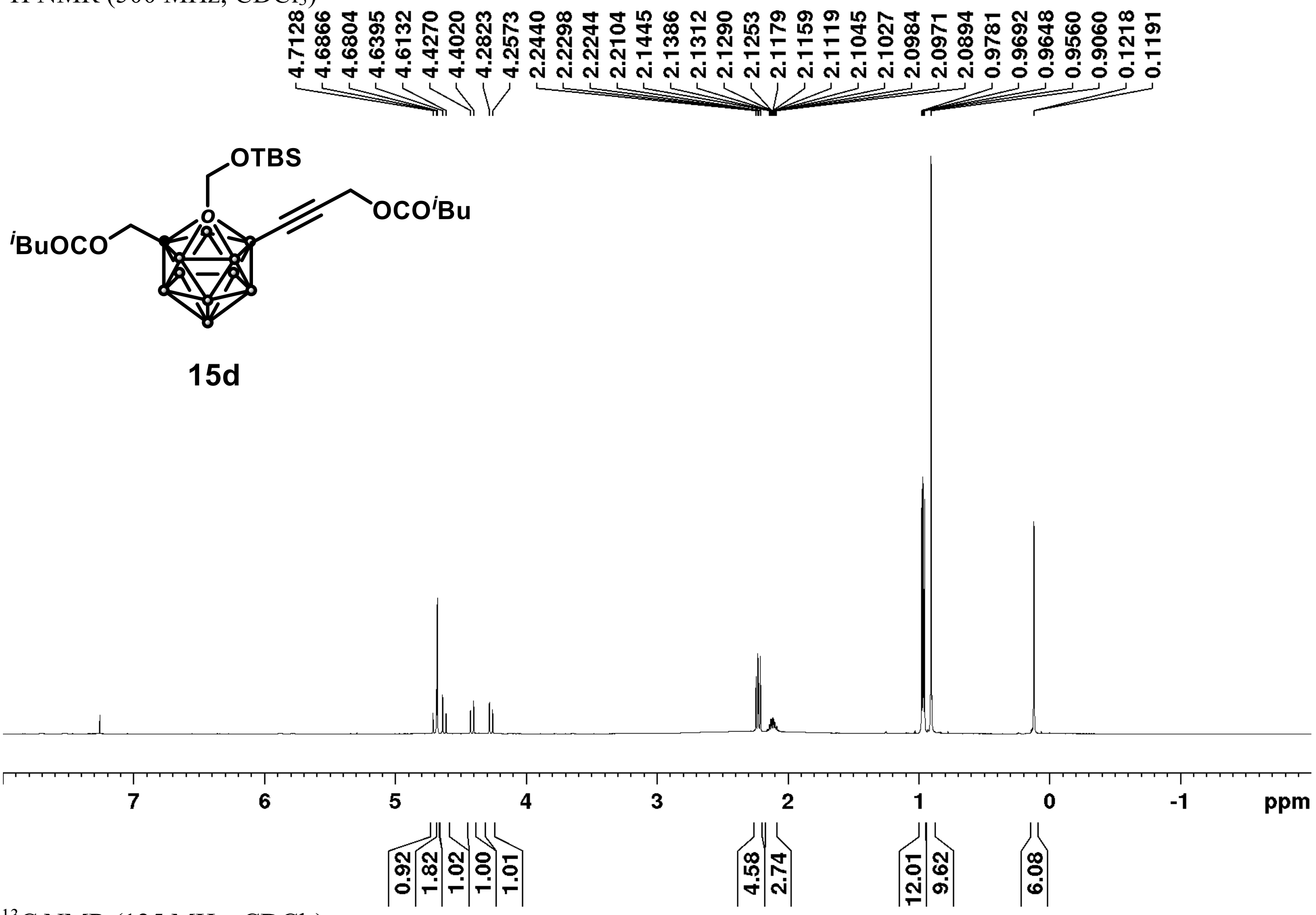

15d

^{13}C NMR (125 MHz; $CDCl_3$)

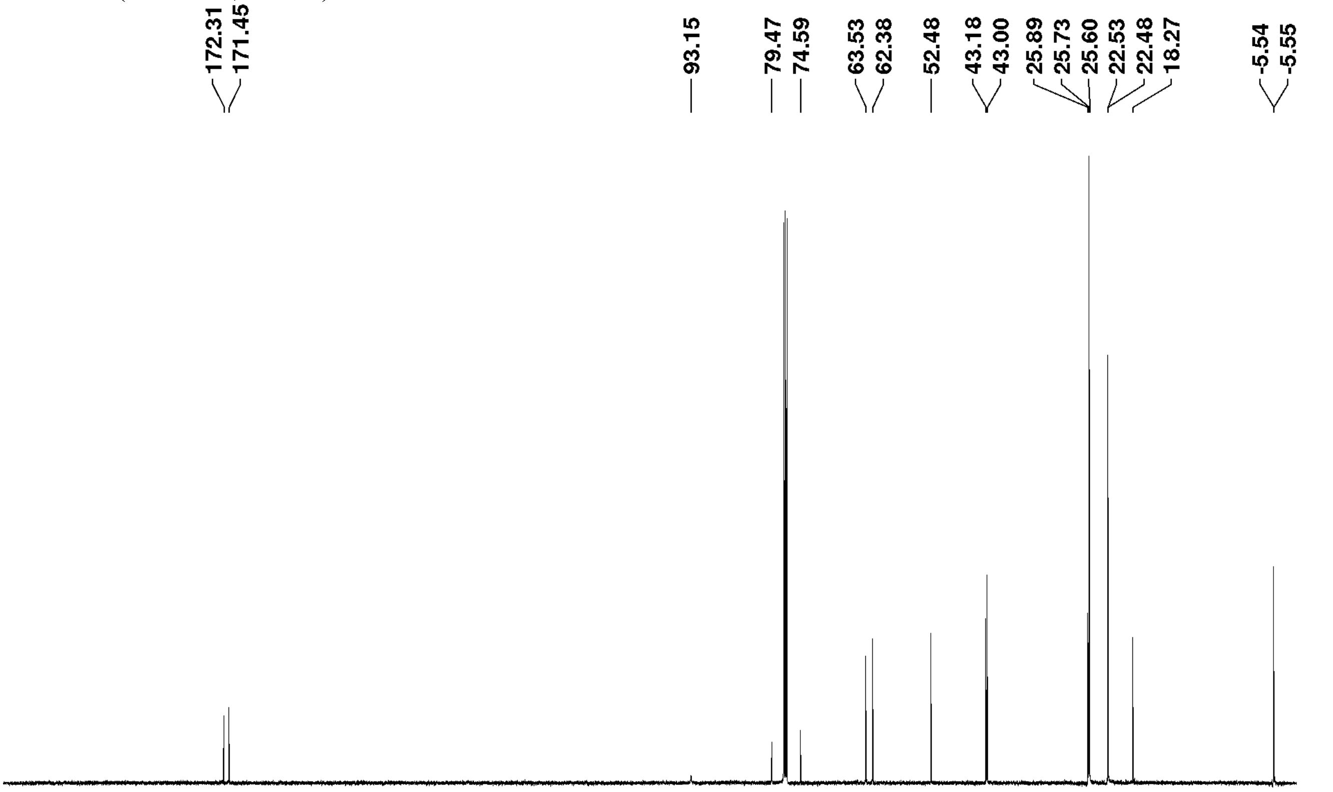

^{11}B NMR (160 MHz; $CDCl_3$)

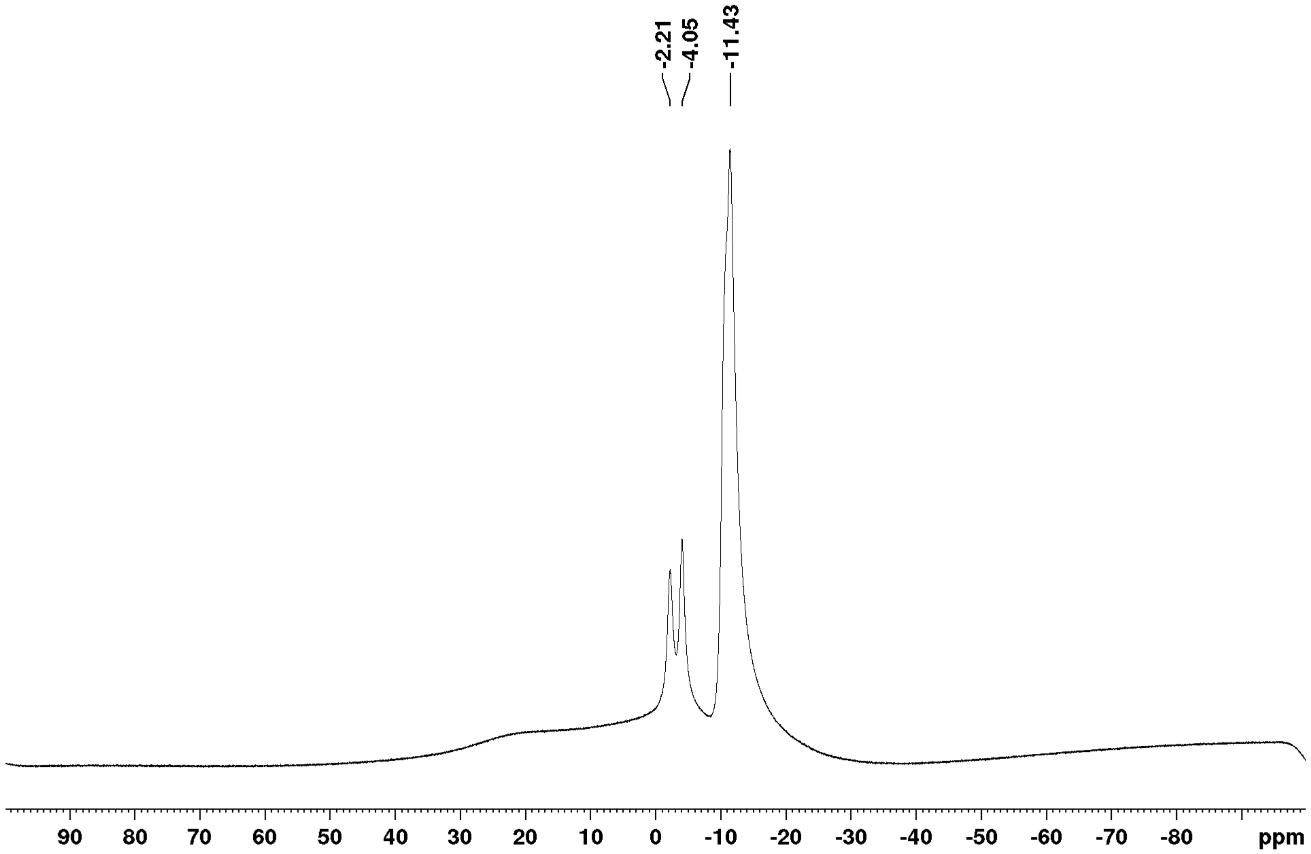

1,2-bis(hydroxymethyl)-4-(2-phenylacetoxy)prop-1-yn-1-yl)-1,2-dicarba-*closo*-dodecaborane (16a)
^{1}H NMR (500 MHz; $CDCl_3$)

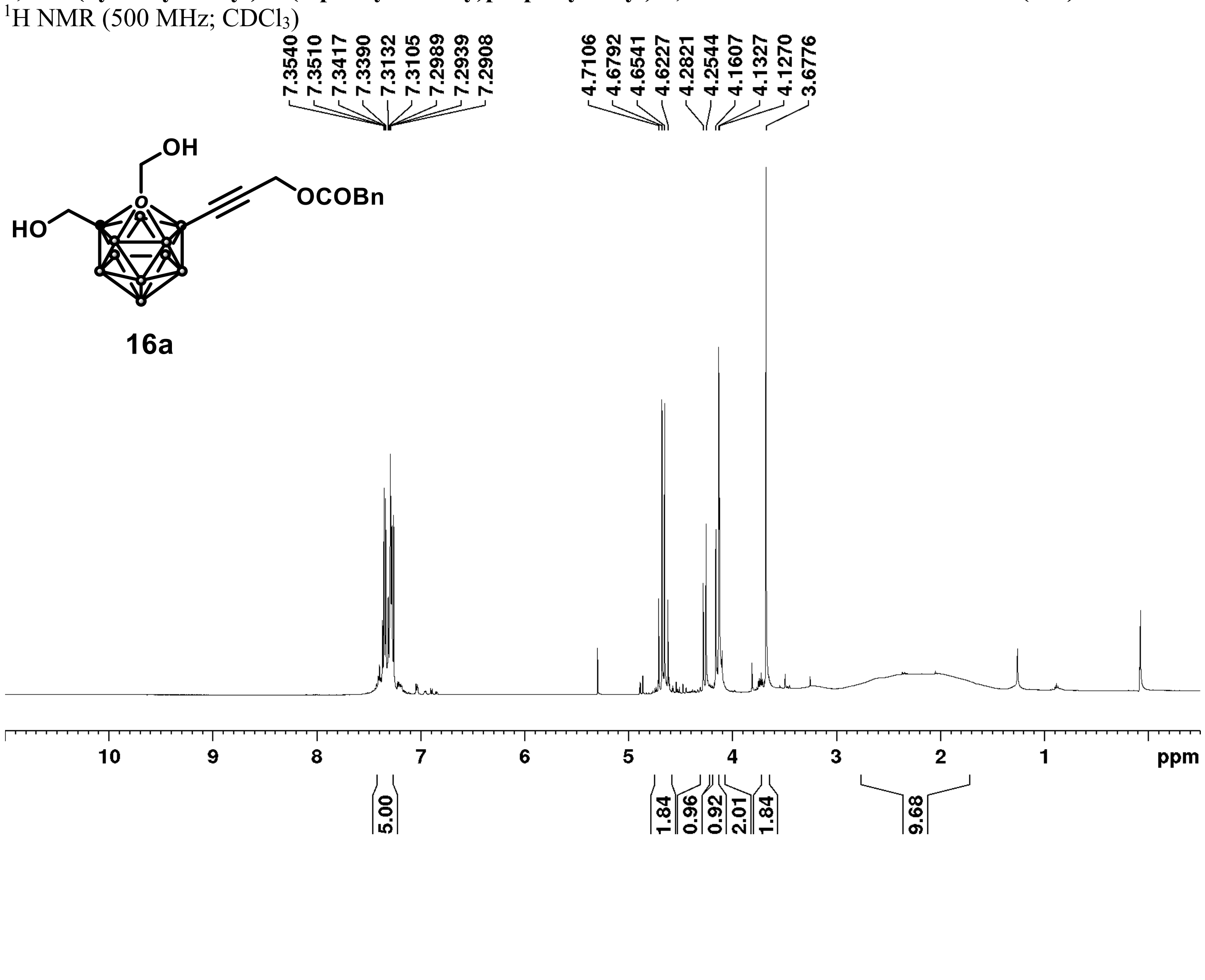

^{13}C NMR (125 MHz; $CDCl_3$)

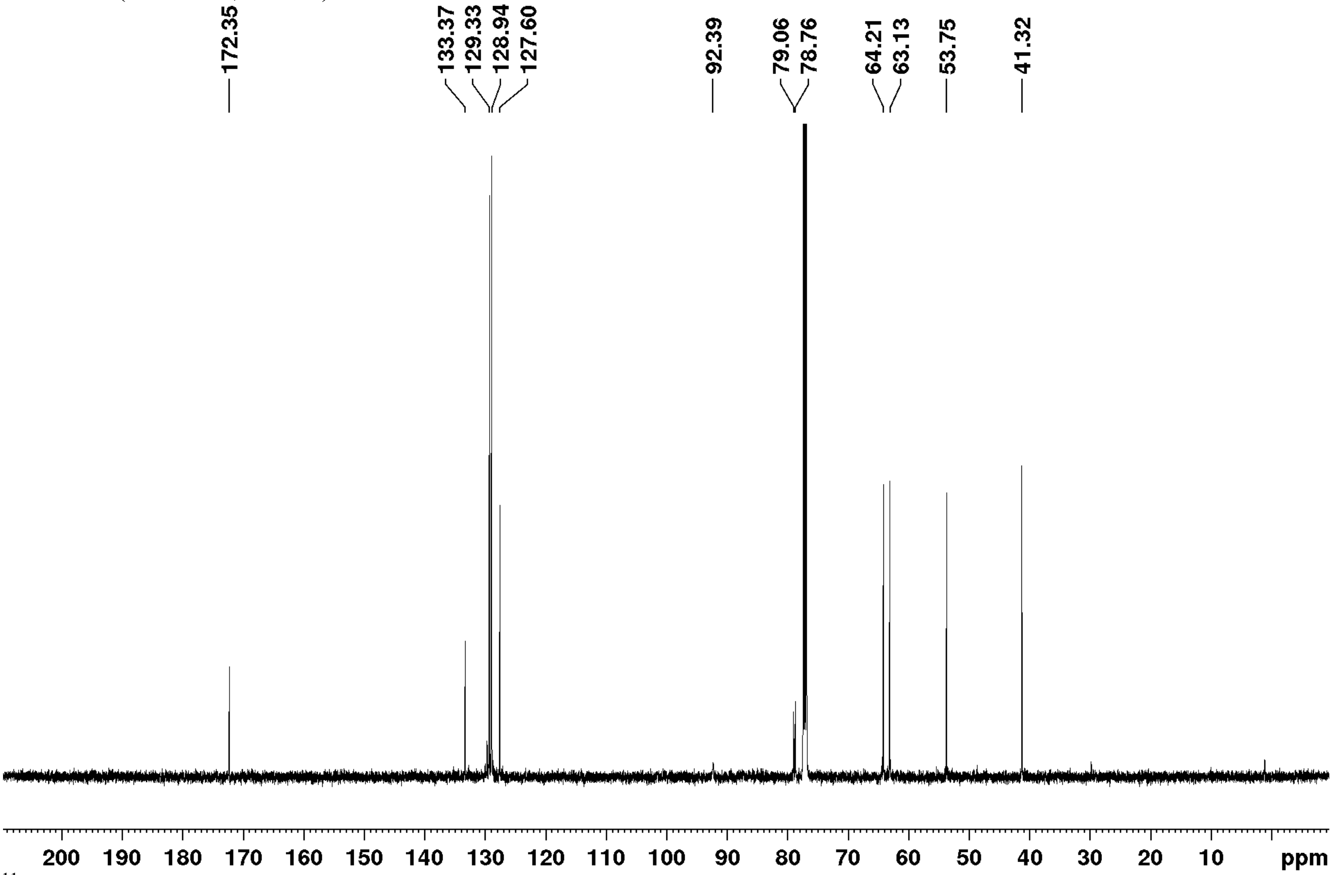

^{11}B NMR (160 MHz; $CDCl_3$)

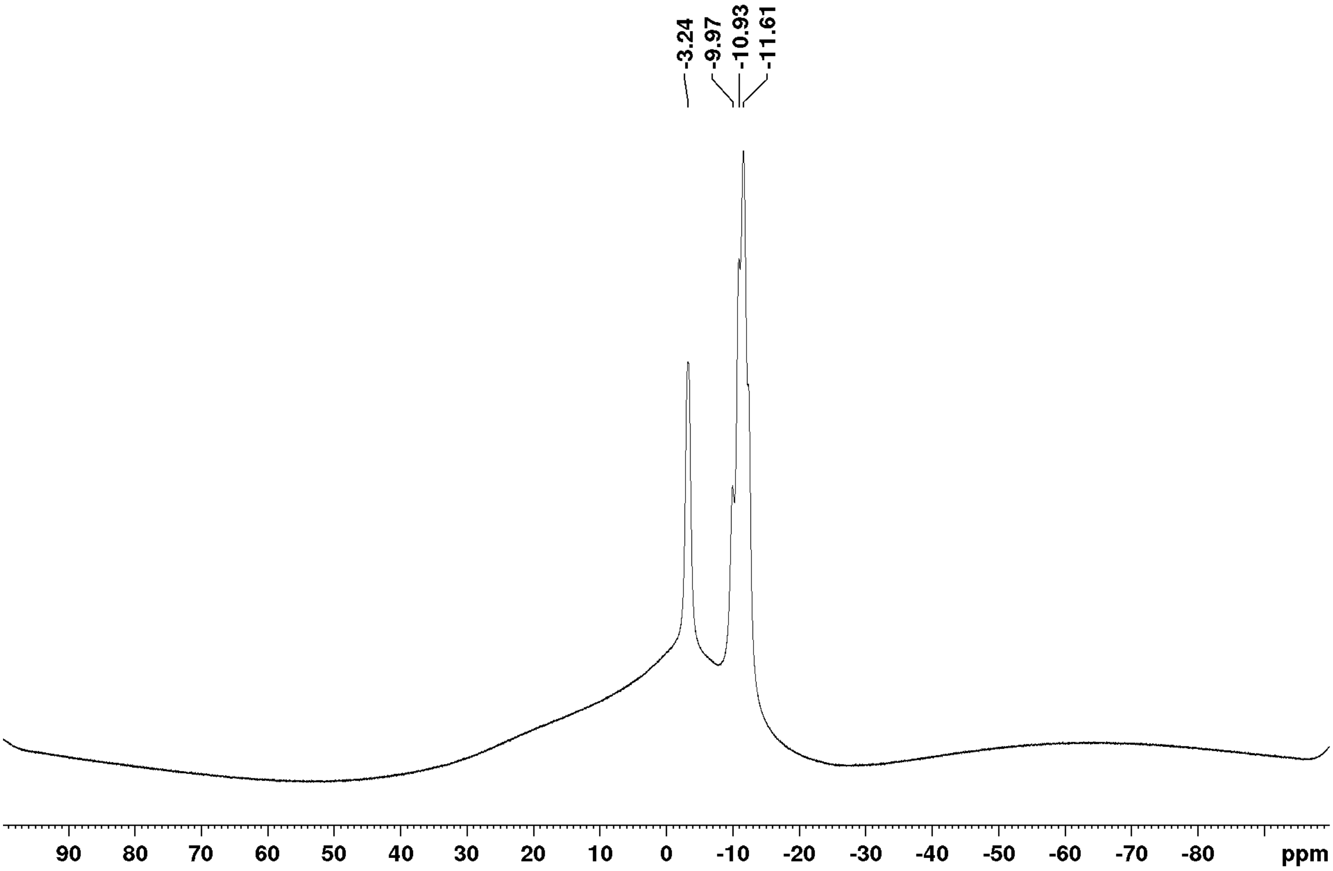

1,2-bis(hydroxymethyl)-4-(3-((3-methylbutanoyl)oxy)prop-1-yn-1-yl)-1,2-dicarba-*closo*-dodecaborane (16b)
^{1}H NMR (500 MHz; $CDCl_3$)

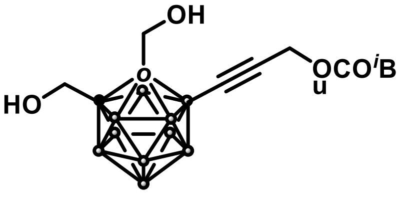

16b

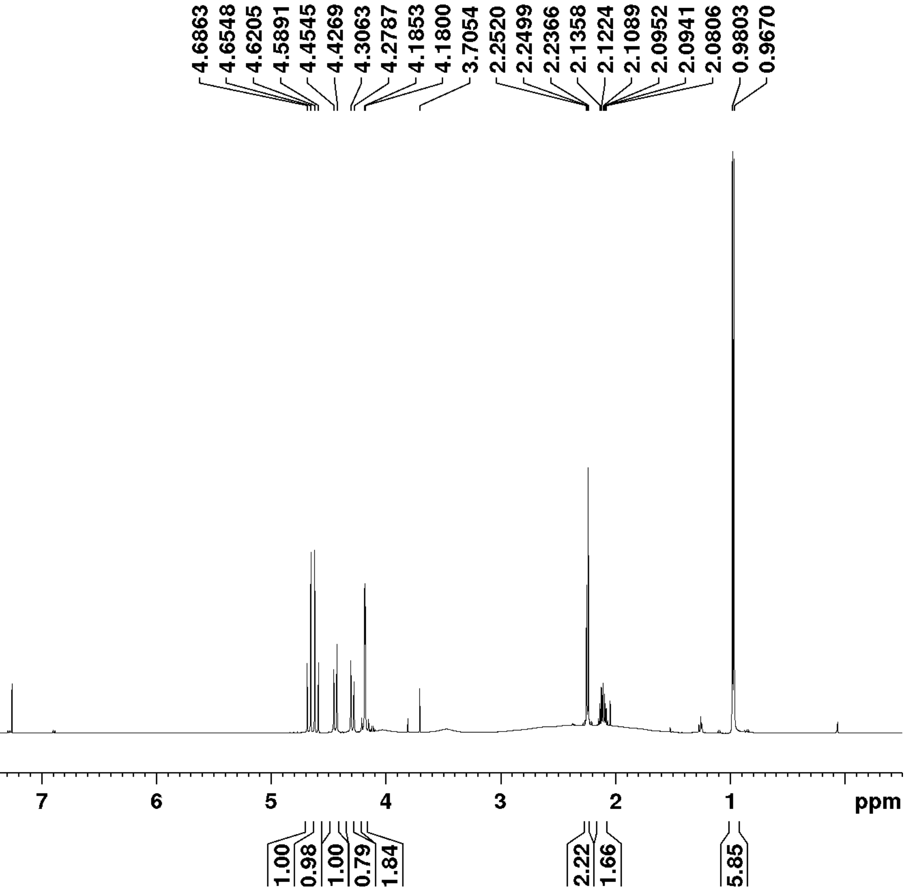

^{13}C NMR (125 MHz; $CDCl_3$)

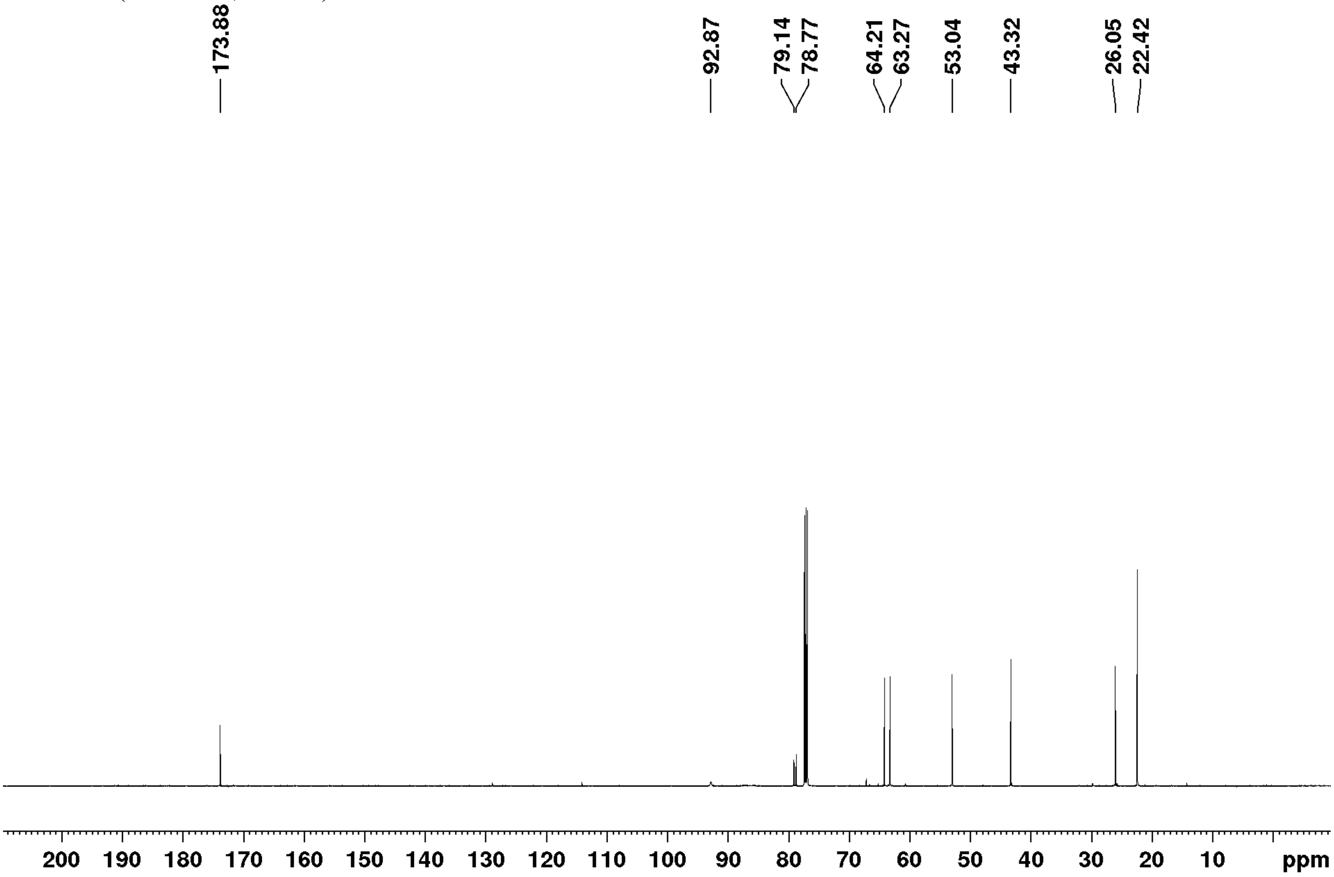

^{11}B NMR (160 MHz; $CDCl_3$)

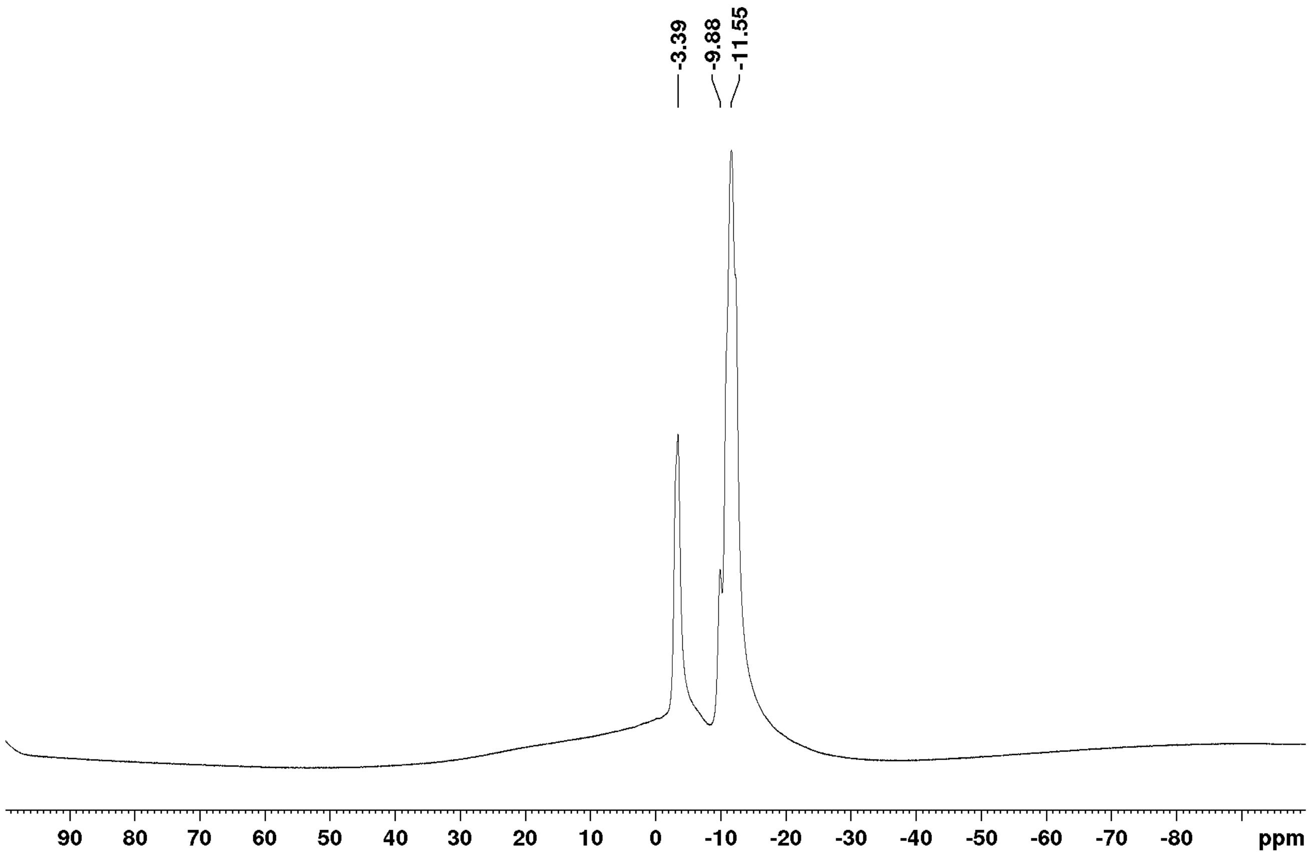

1-((3-methylbutanoyl)oxymethyl)-2-((*tert*-butyl)dimethylsilyl)-hydroxymethyl)-4-(2-phenylacetoxy)prop-1-yn-1-yl)-1,2-dicarba-*closo*-dodecaborane (IIa)

^{1}H NMR (500 MHz; $CDCl_3$)

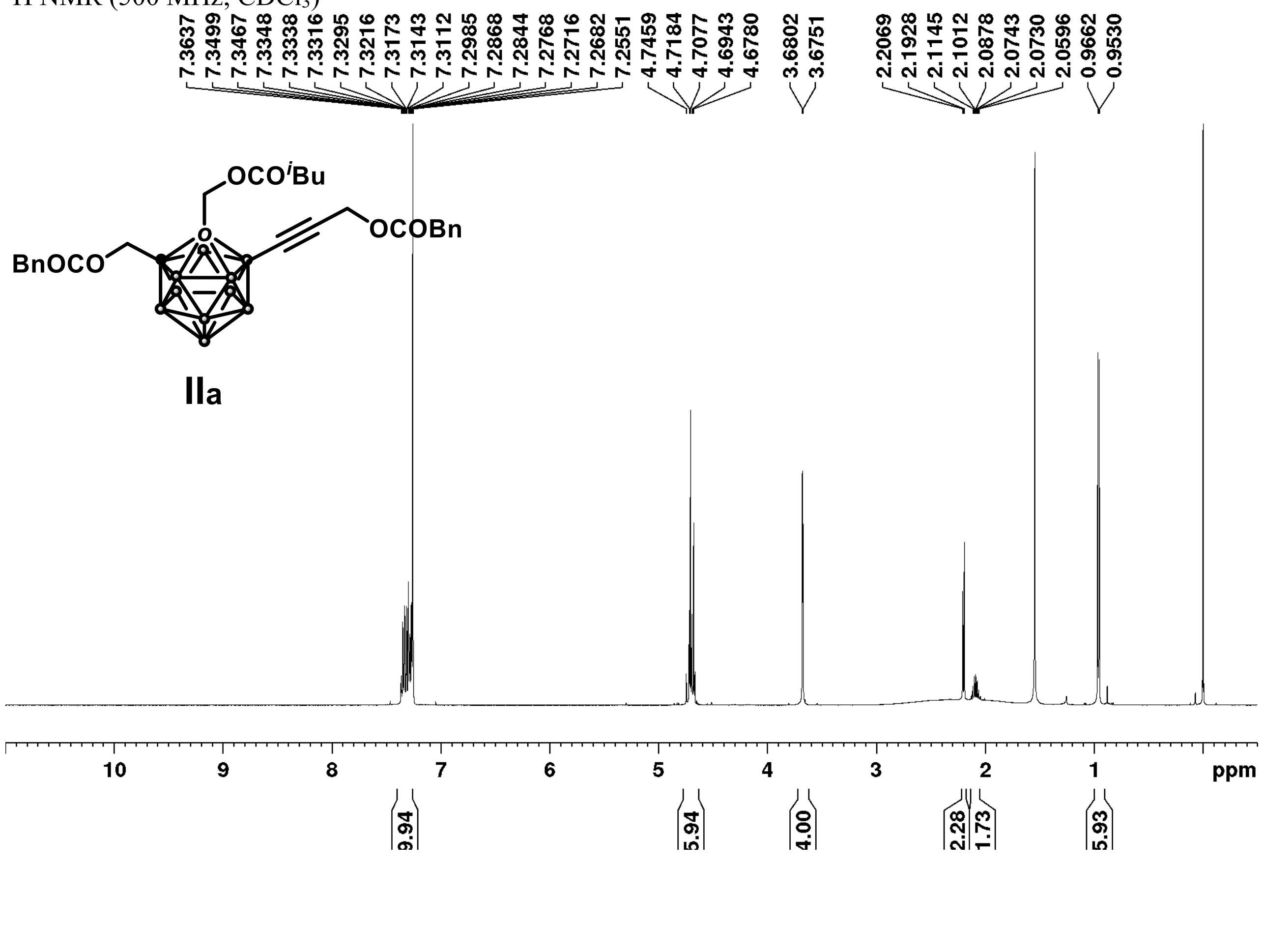

^{13}C NMR (125 MHz; $CDCl_3$)

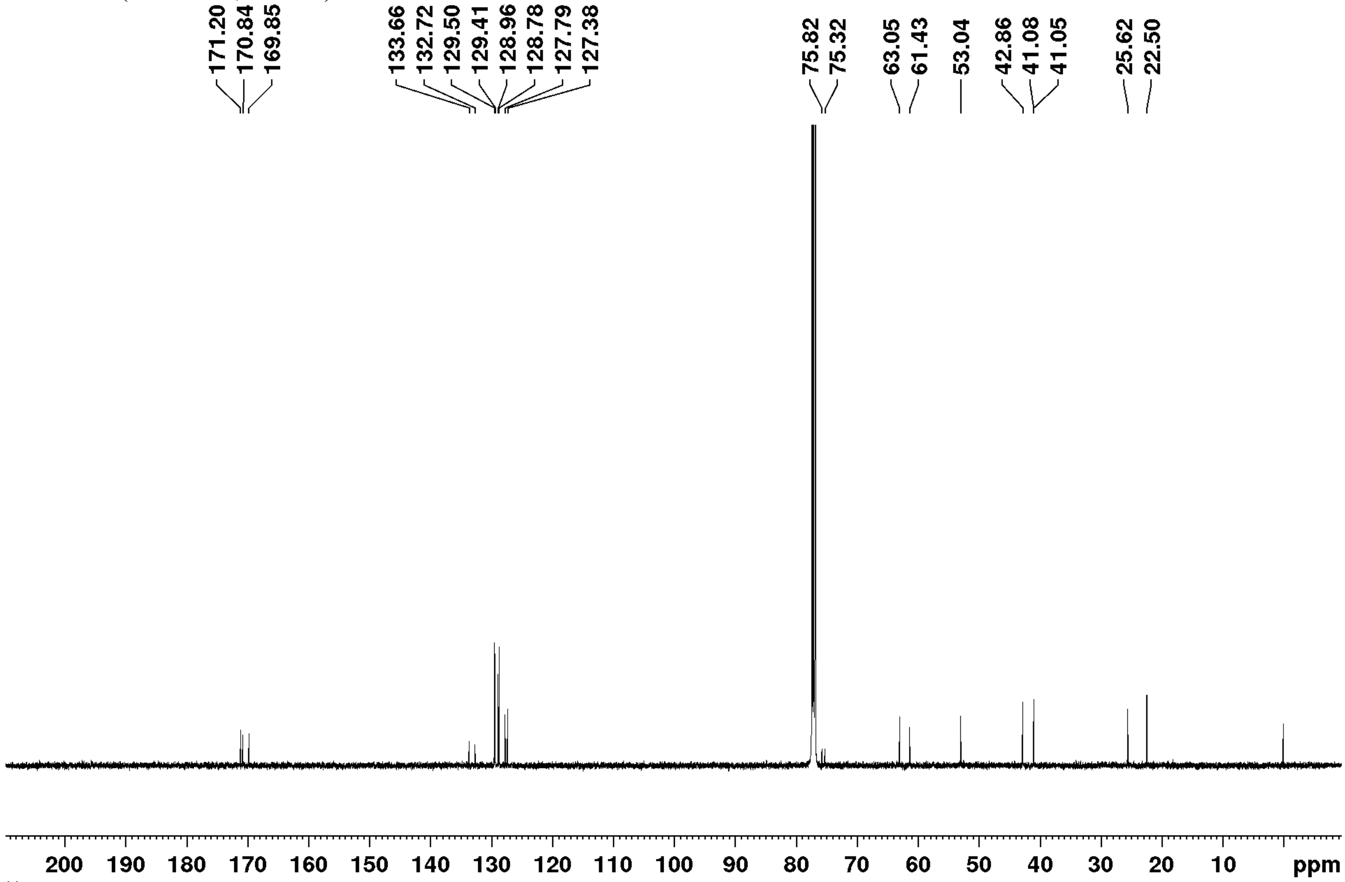

^{11}B NMR (160 MHz; $CDCl_3$)

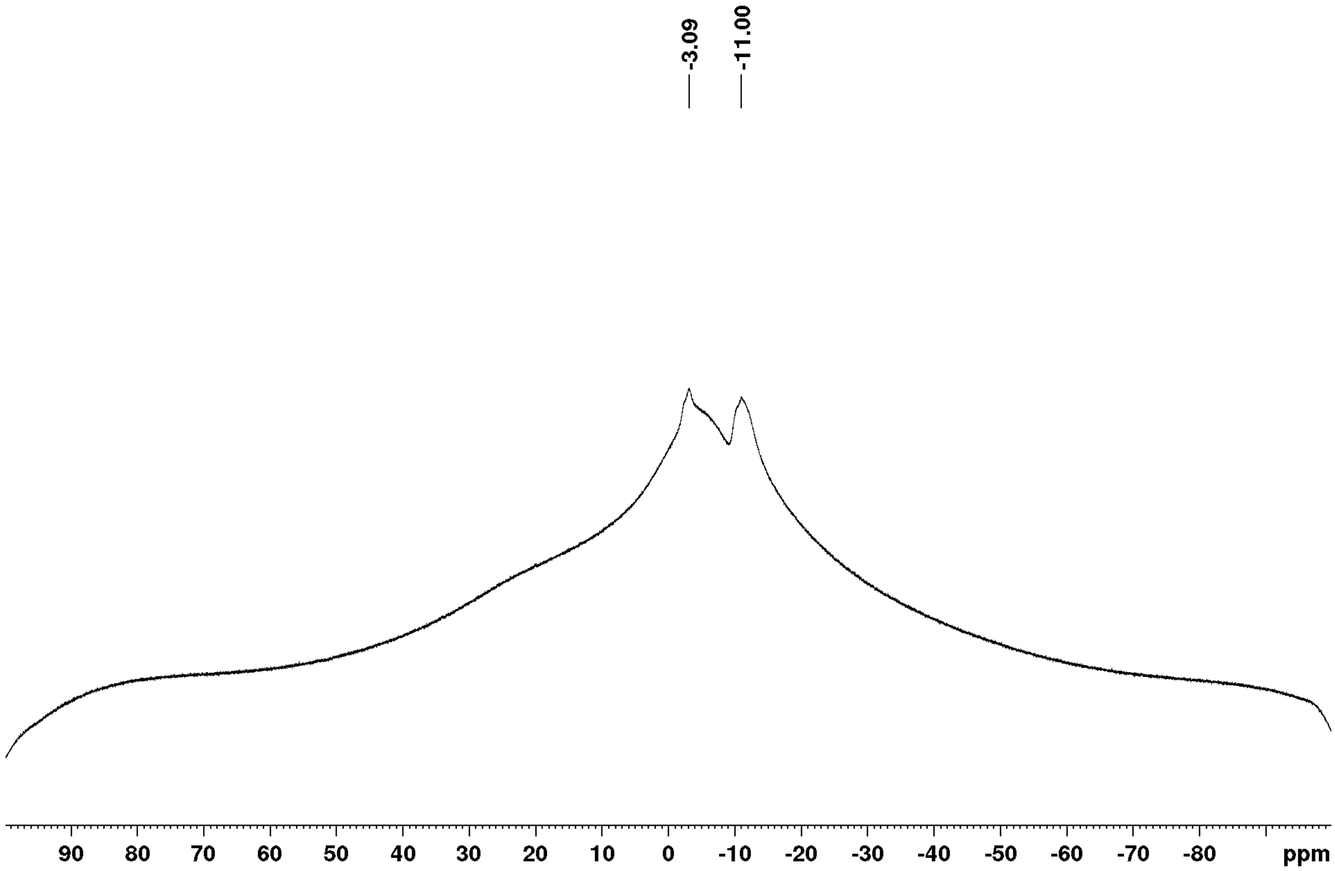

1-((3-methylbutanoyl)oxymethyl)-2-(2-phenylacetoxymethyl)-4-((3-methylbutanoyl)prop-1-yn-1-yl)-1,2-dicarba-*closo*-dodecaborane (IIb)

^{1}H NMR (500 MHz; $CDCl_3$)

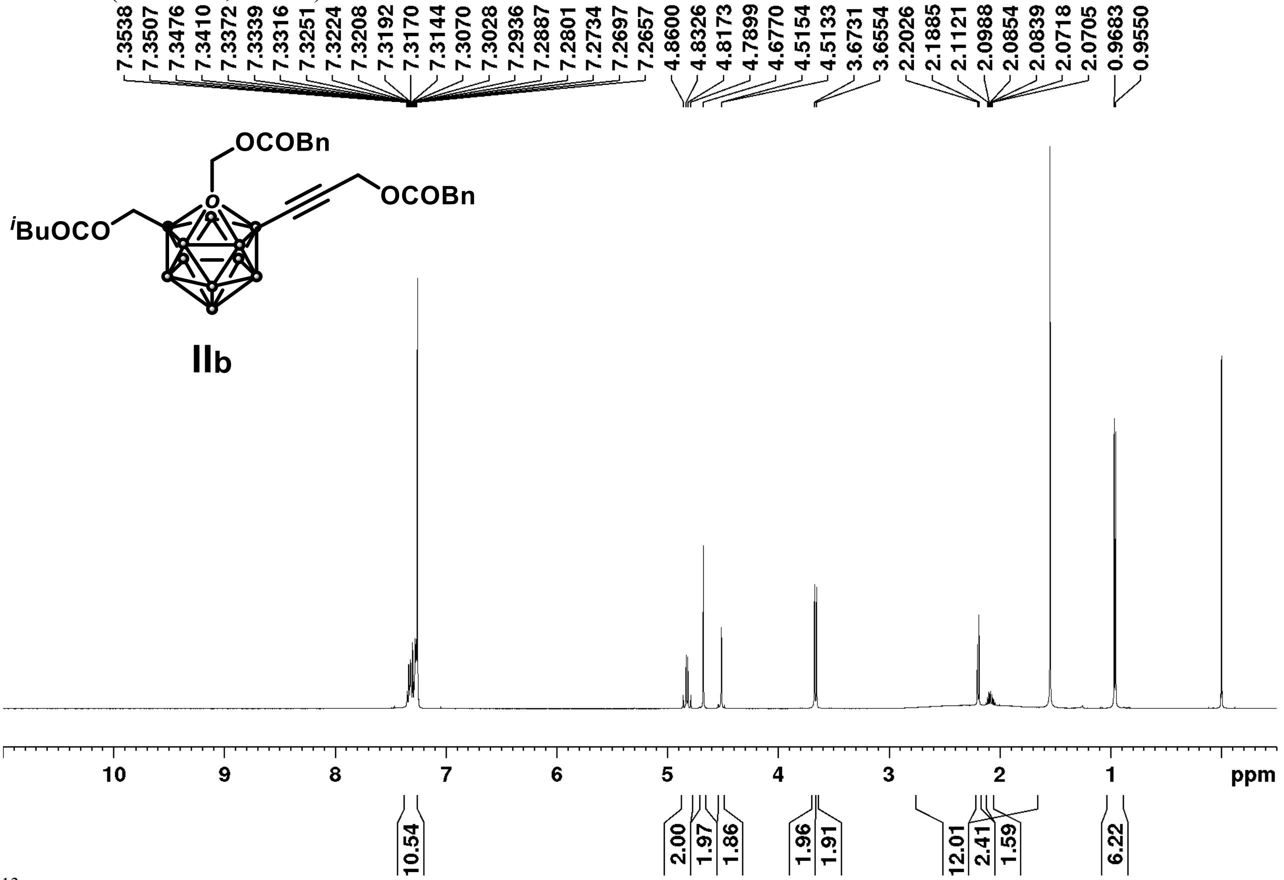

^{13}C NMR (125 MHz; $CDCl_3$)

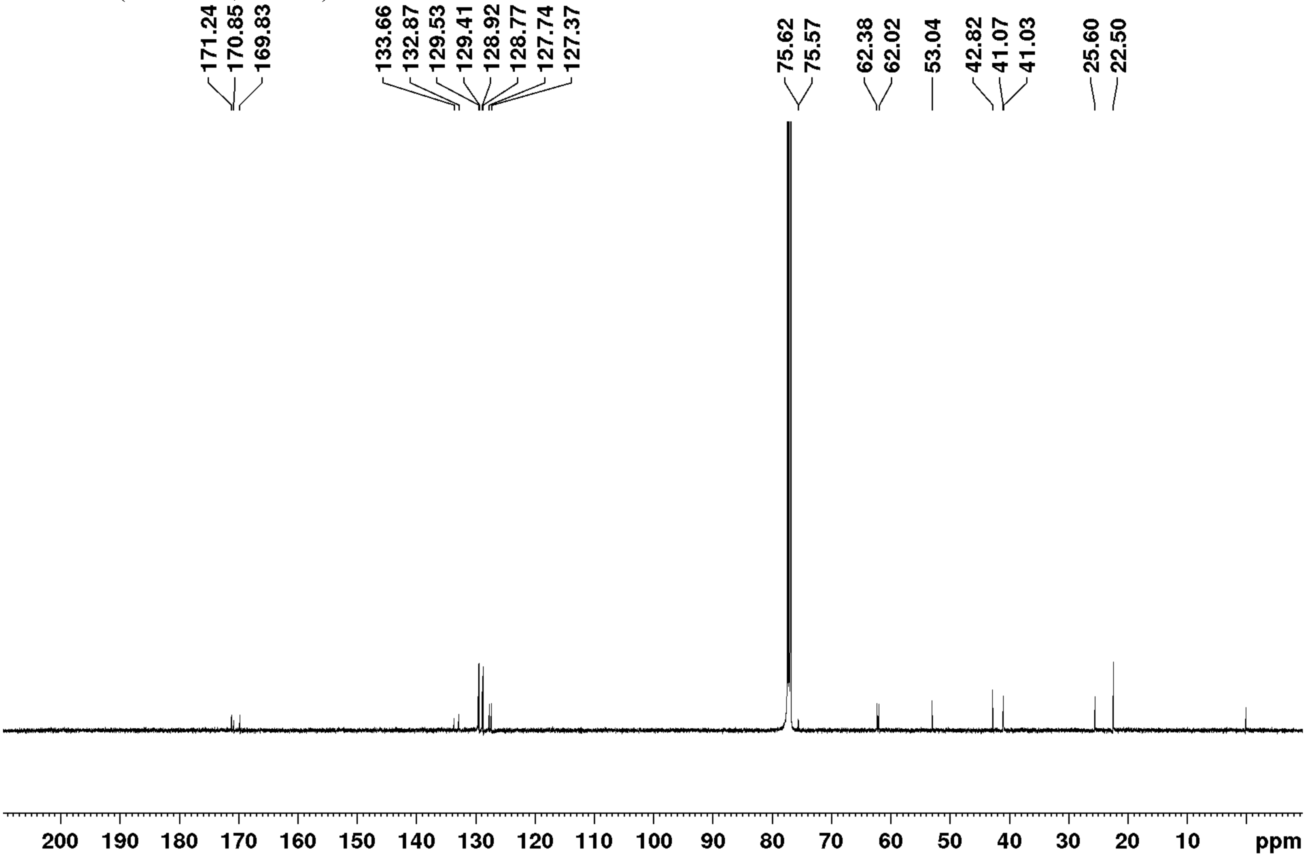

^{11}B NMR (160 MHz; $CDCl_3$)

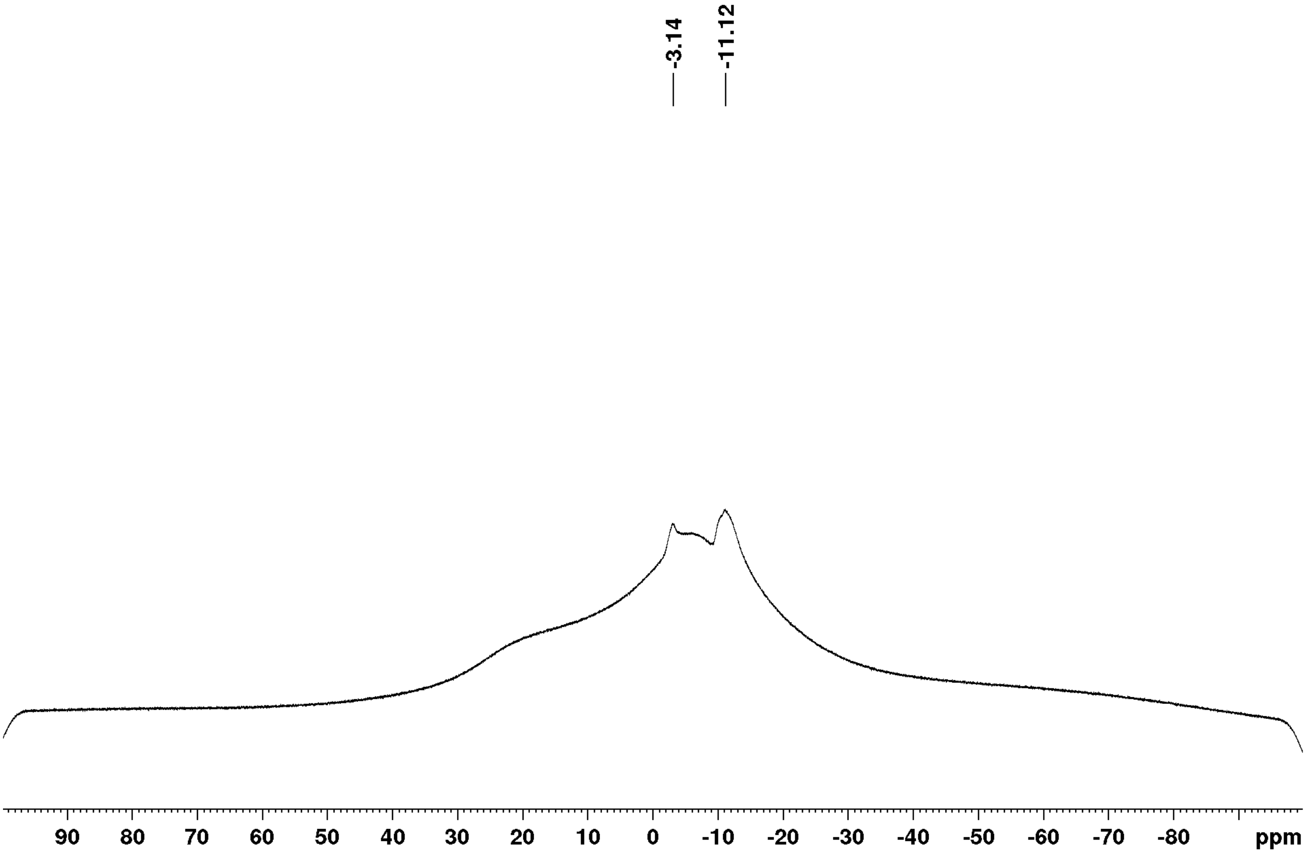

1-(2-phenylacetoxymethyl)-2-((3-methylbutanoyl)oxymethyl)-4-((3-methylbutanoyl)prop-1-yn-1-yl)-1,2-dicarba-*closo*-dodecaborane (IIc)

^{1}H NMR (500 MHz; $CDCl_3$)

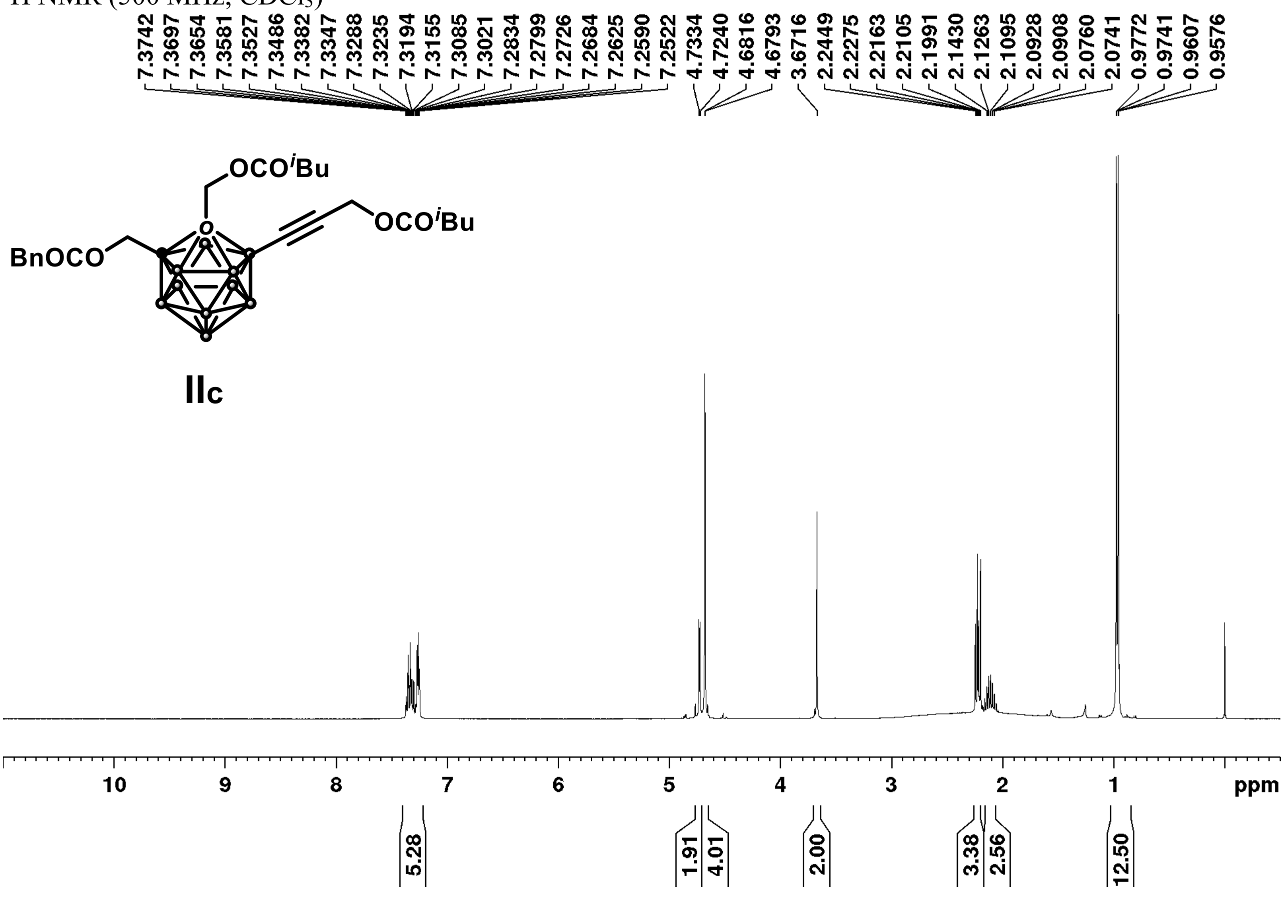

^{13}C NMR (125 MHz; CDCl$_3$)

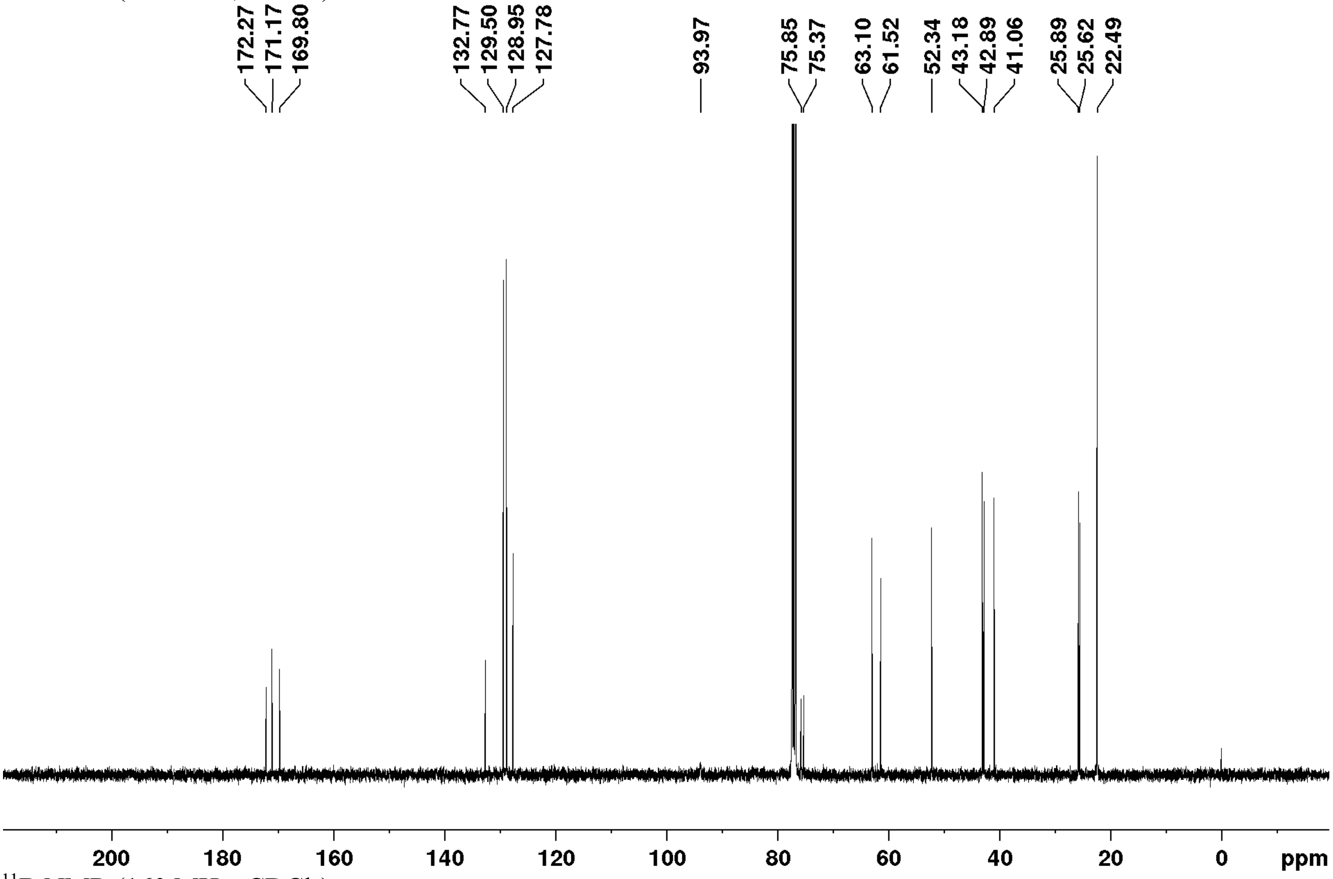

^{11}B NMR (160 MHz; CDCl$_3$)

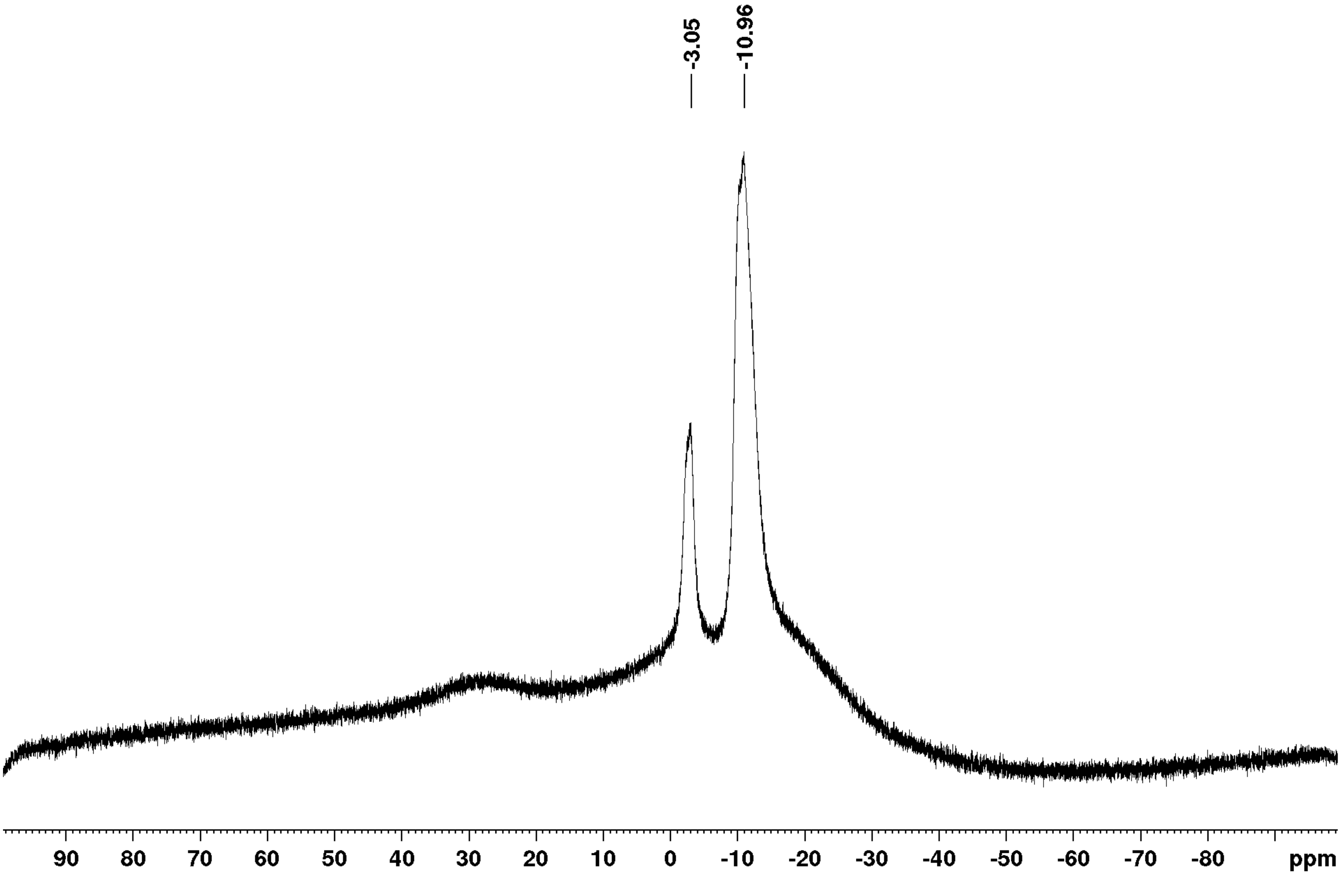

1-(3-methylbutanoyl)-2-(2-phenylacetoxymethyl)-4-((3-methylbutanoyl)prop-1-yn-1-yl)-1,2-dicarba-*closo*-dodecaborane (IId)

^{1}H NMR (500 MHz; $CDCl_3$)

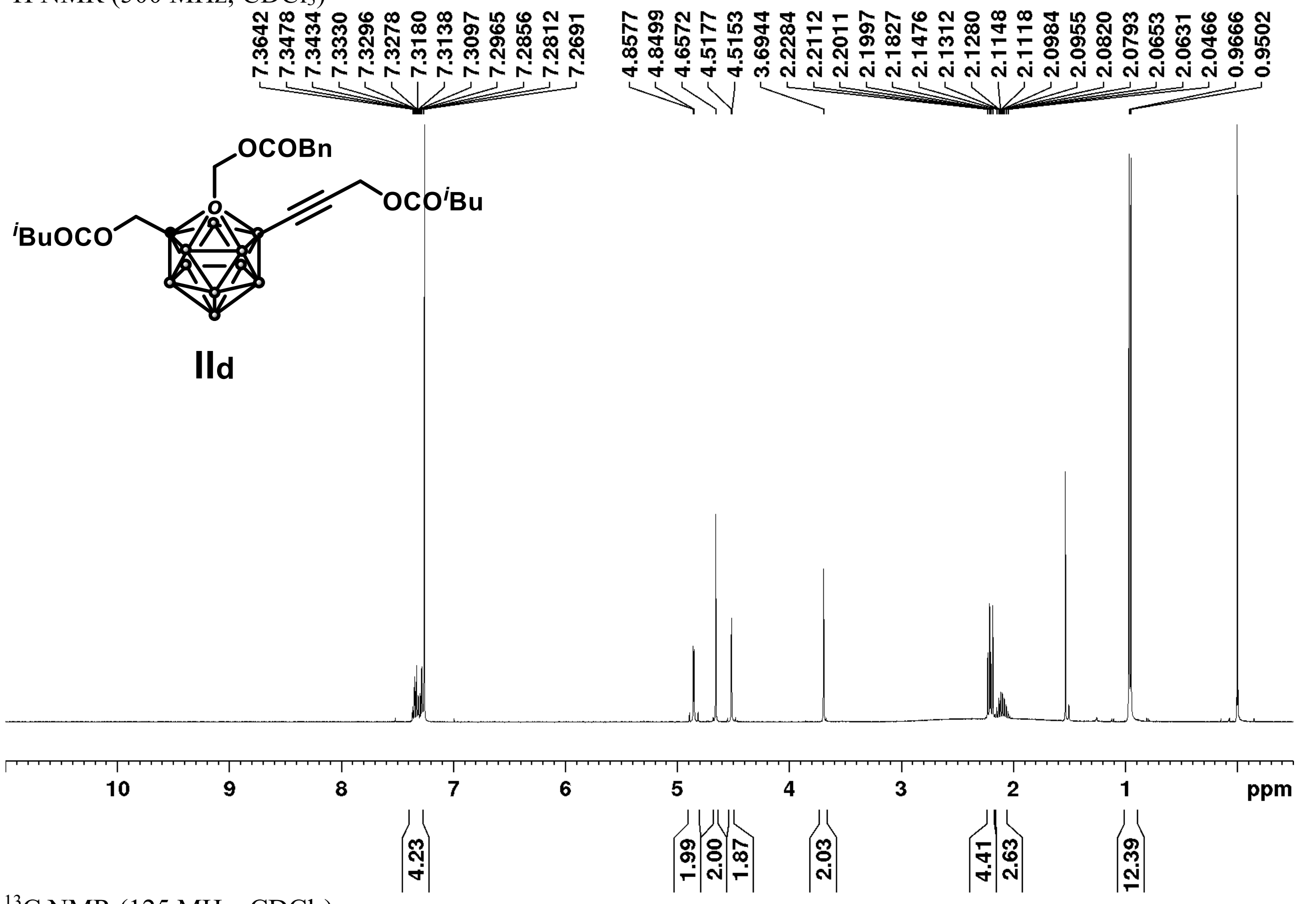

^{13}C NMR (125 MHz; $CDCl_3$)

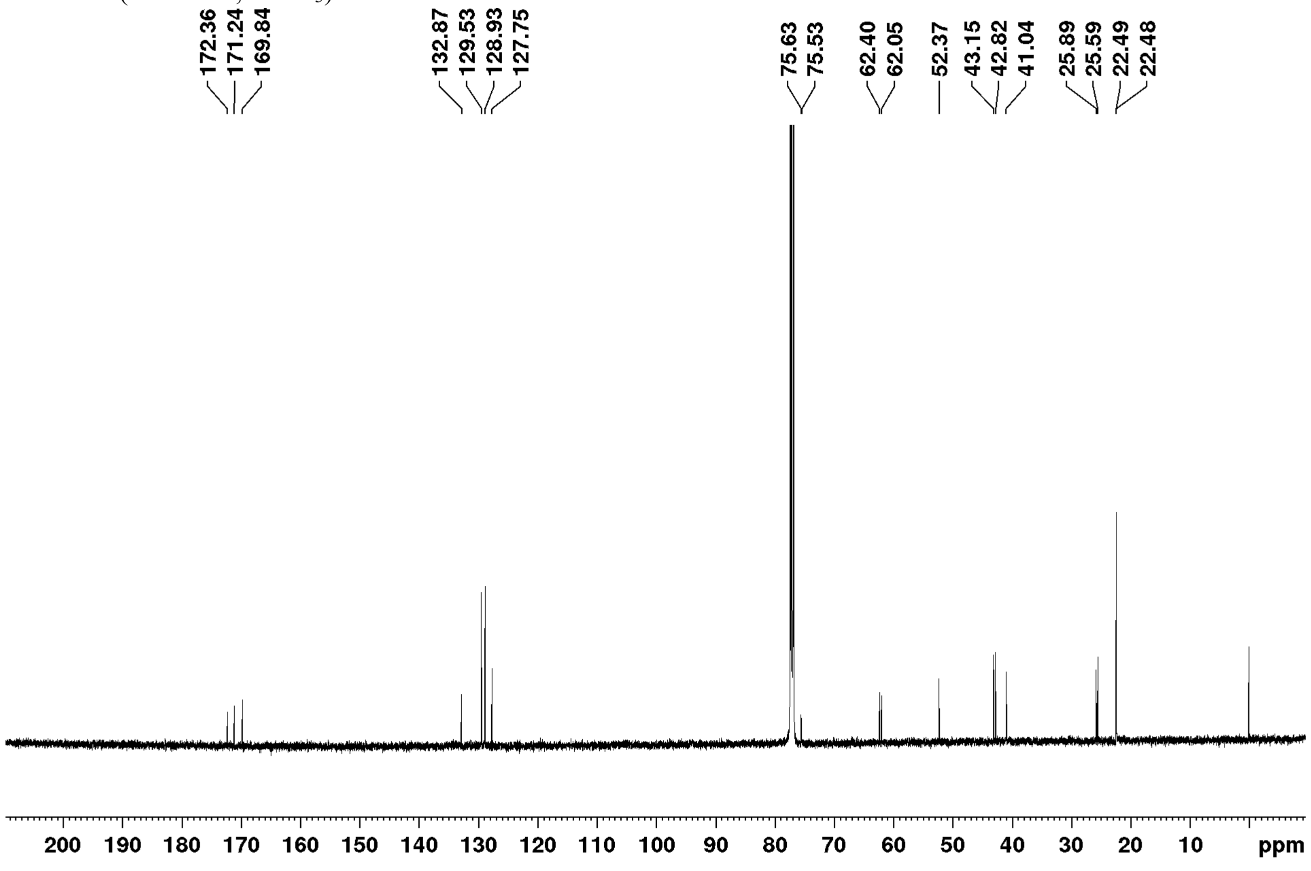

^{11}B NMR (160 MHz; CDCl$_3$)

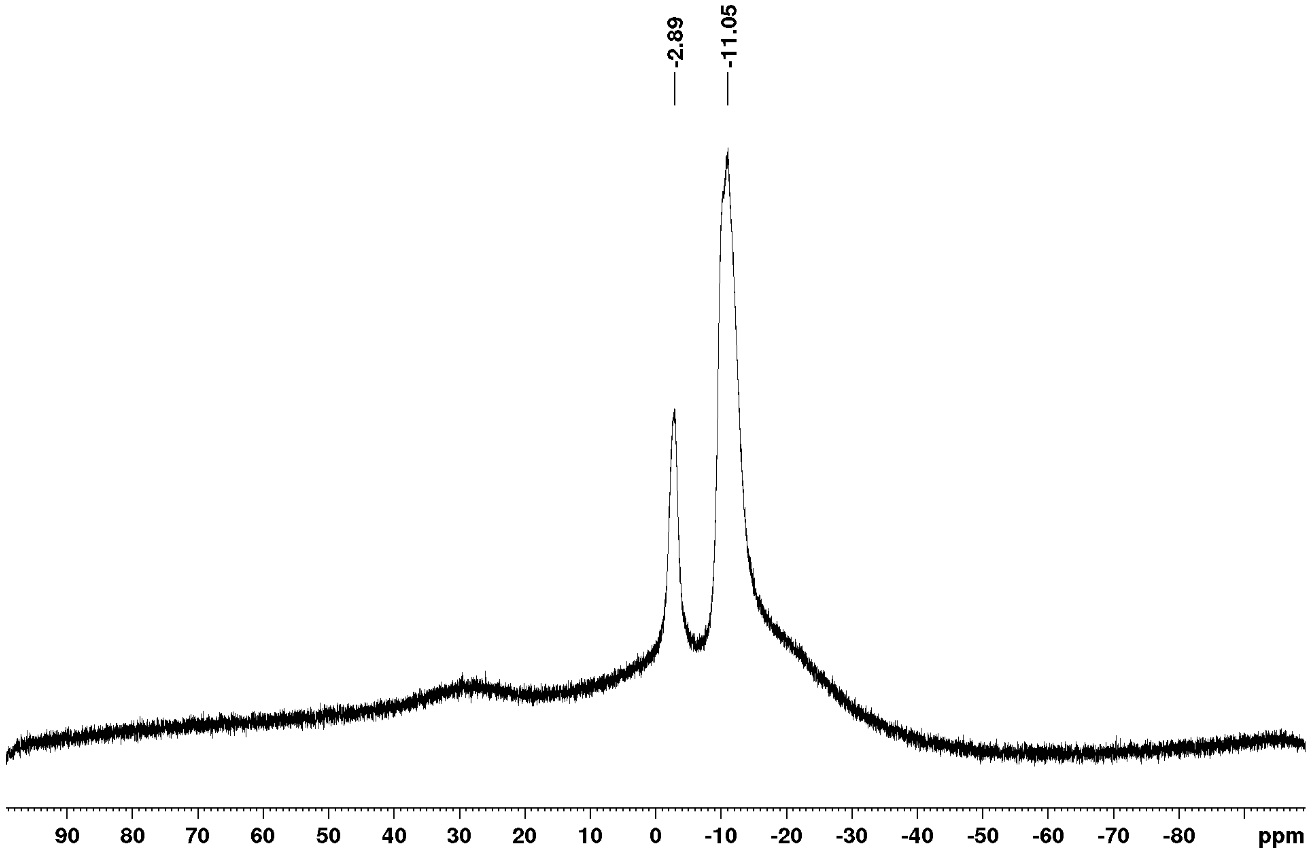

1,2-bis(phenylacetoxymethyl)-4-(2-phenylacetoxy)prop-1-yn-1-yl)-1,2-dicarba-*closo*-dodecaborane (IIe)

^{1}H NMR (500 MHz; CDCl$_3$)

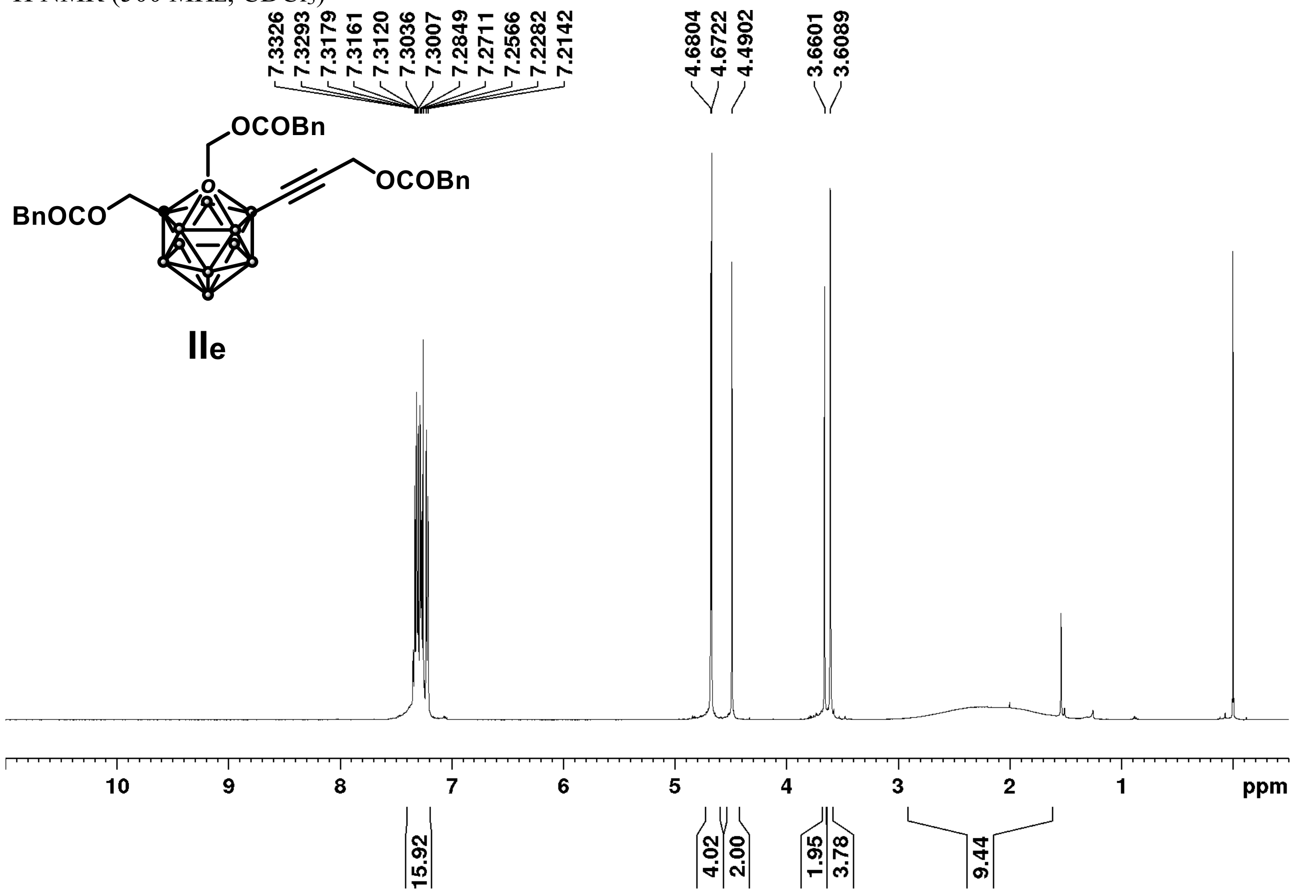

^{13}C NMR (125 MHz; $CDCl_3$)

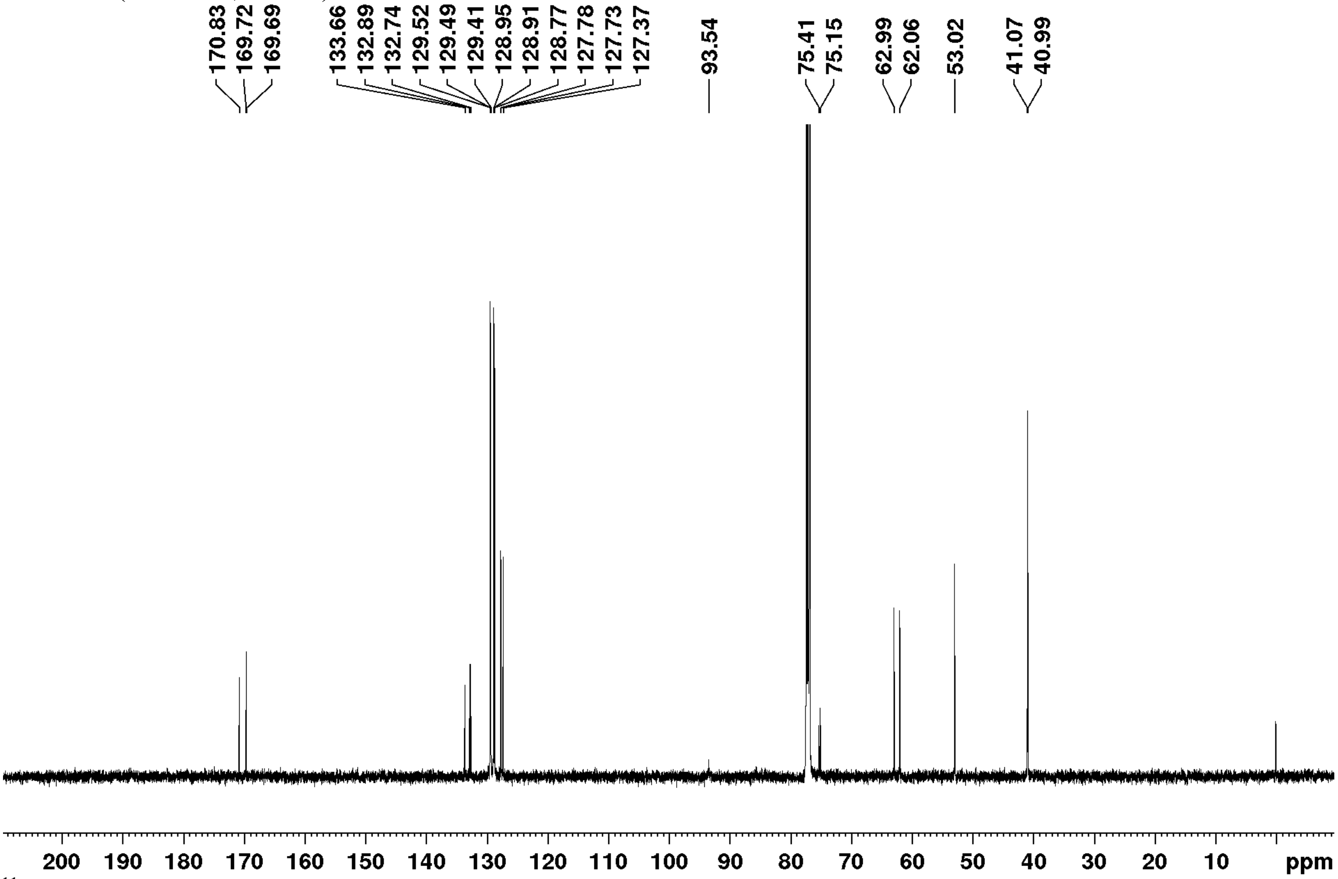

^{11}B NMR (160 MHz; $CDCl_3$)

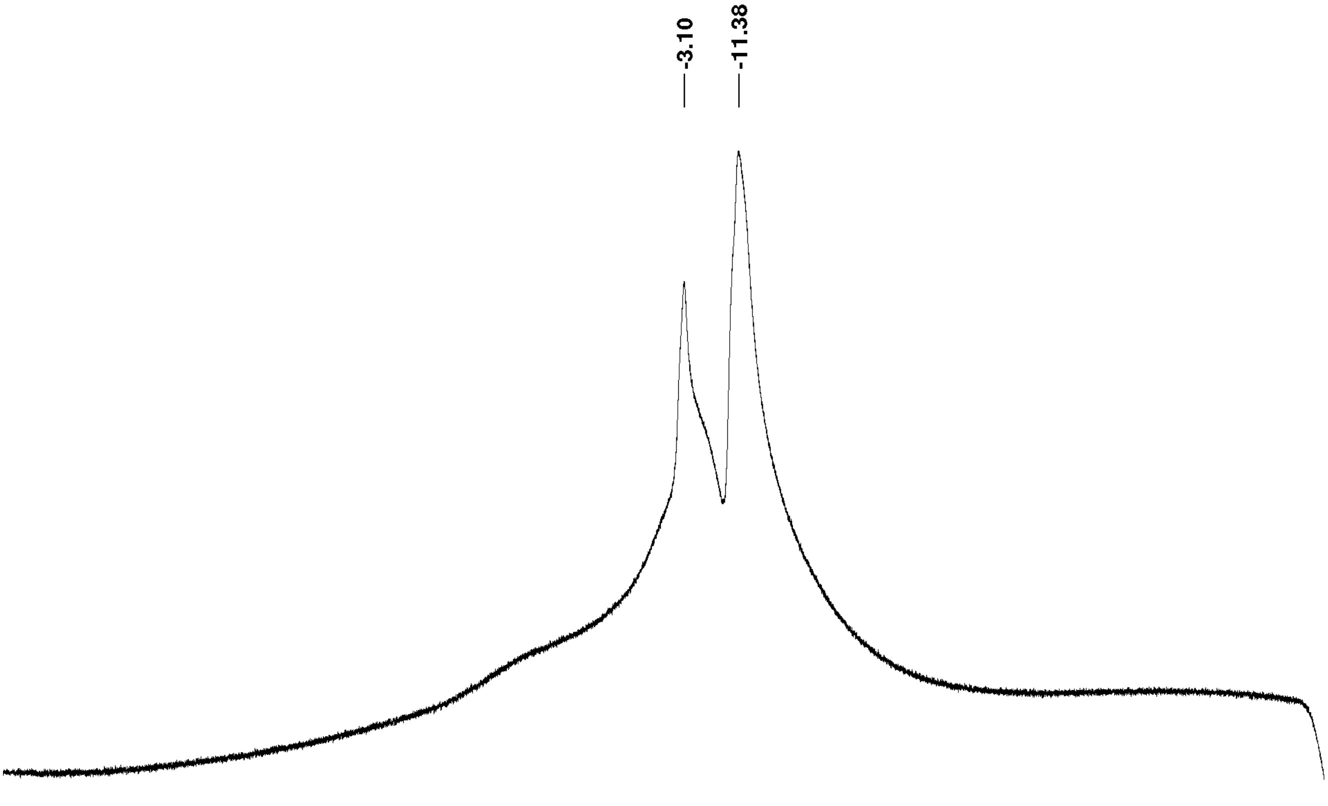

1,2-bis((3-methylbutanoyl)oxymethyl)-4-(2-phenylacetoxy)prop-1-yn-1-yl)-1,2-dicarba-*closo*-dodecaborane (IIf)

^{1}H NMR (500 MHz; $CDCl_3$)

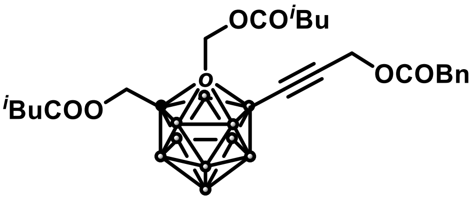

IIf

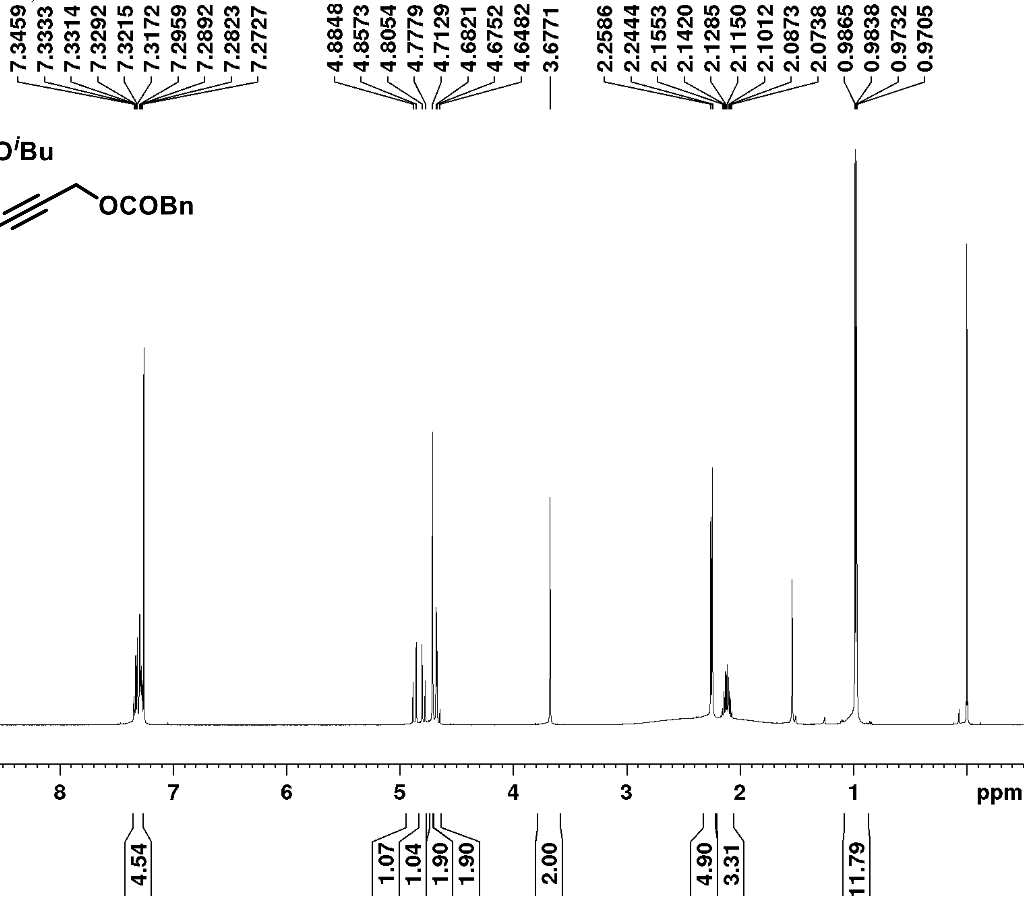

^{13}C NMR (125 MHz; $CDCl_3$)

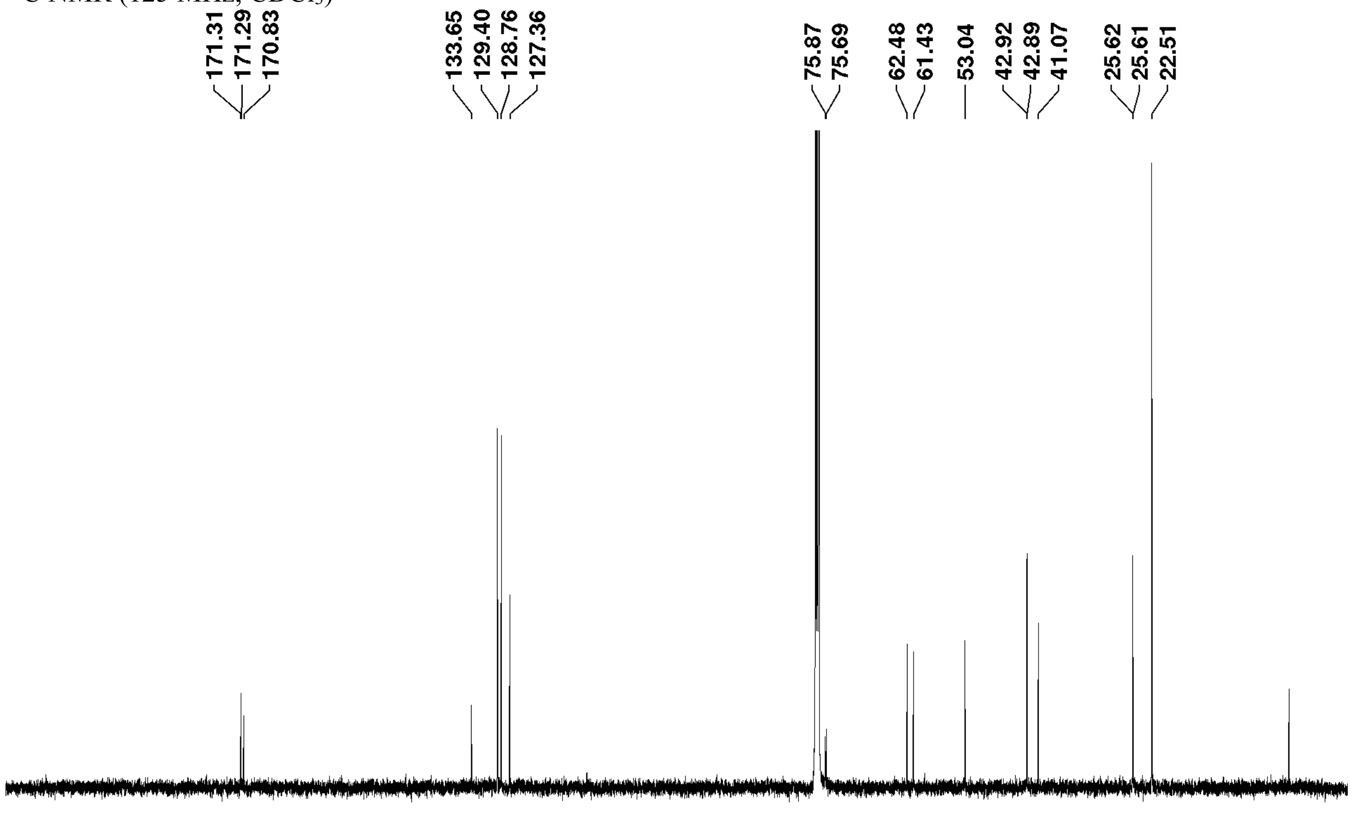

^{11}B NMR (160 MHz; $CDCl_3$)

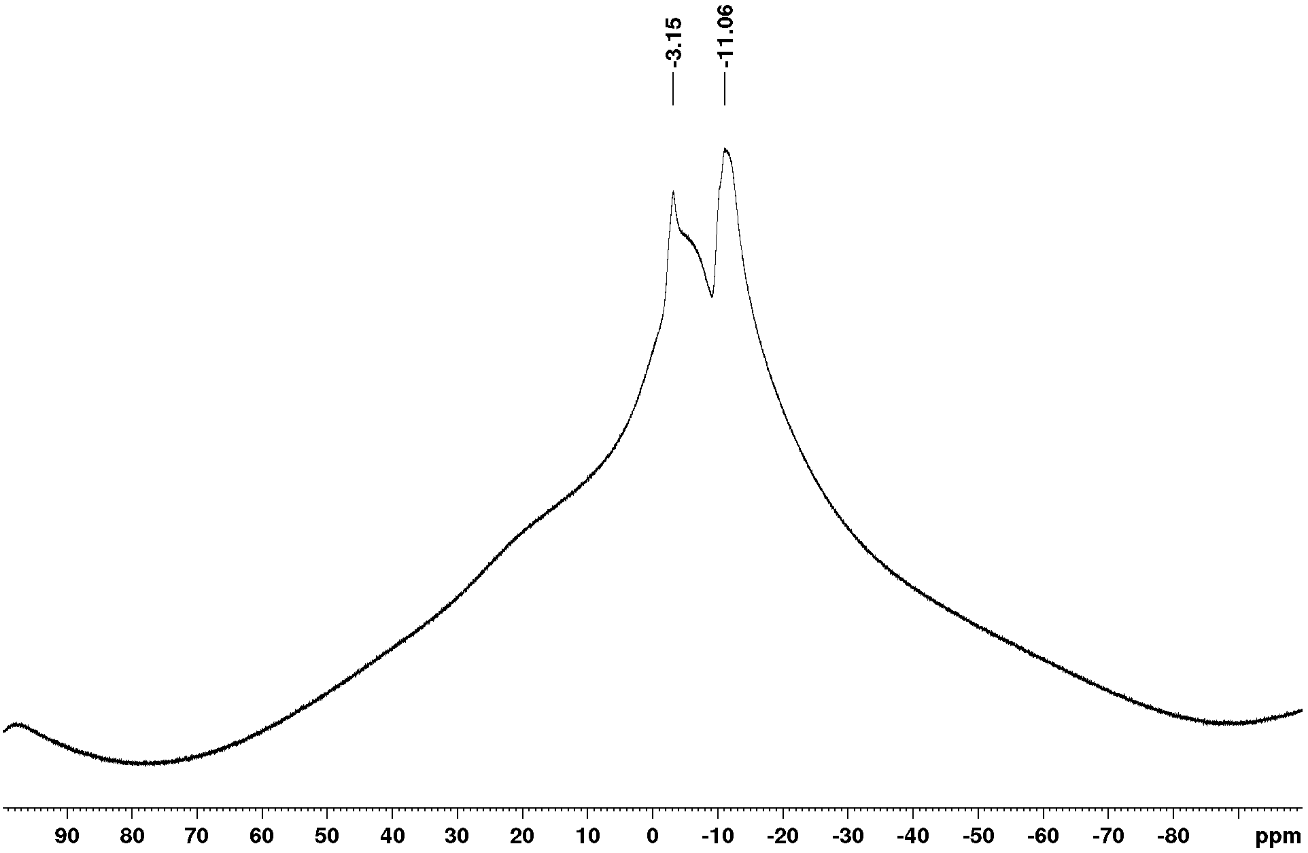

1,2-bis(phenylacetoxymethyl)-4-(2-phenylacetoxy)prop-1-yn-1-yl)-1,2-dicarba-*closo*-dodecaborane (IIg)

^{1}H NMR (500 MHz; $CDCl_3$)

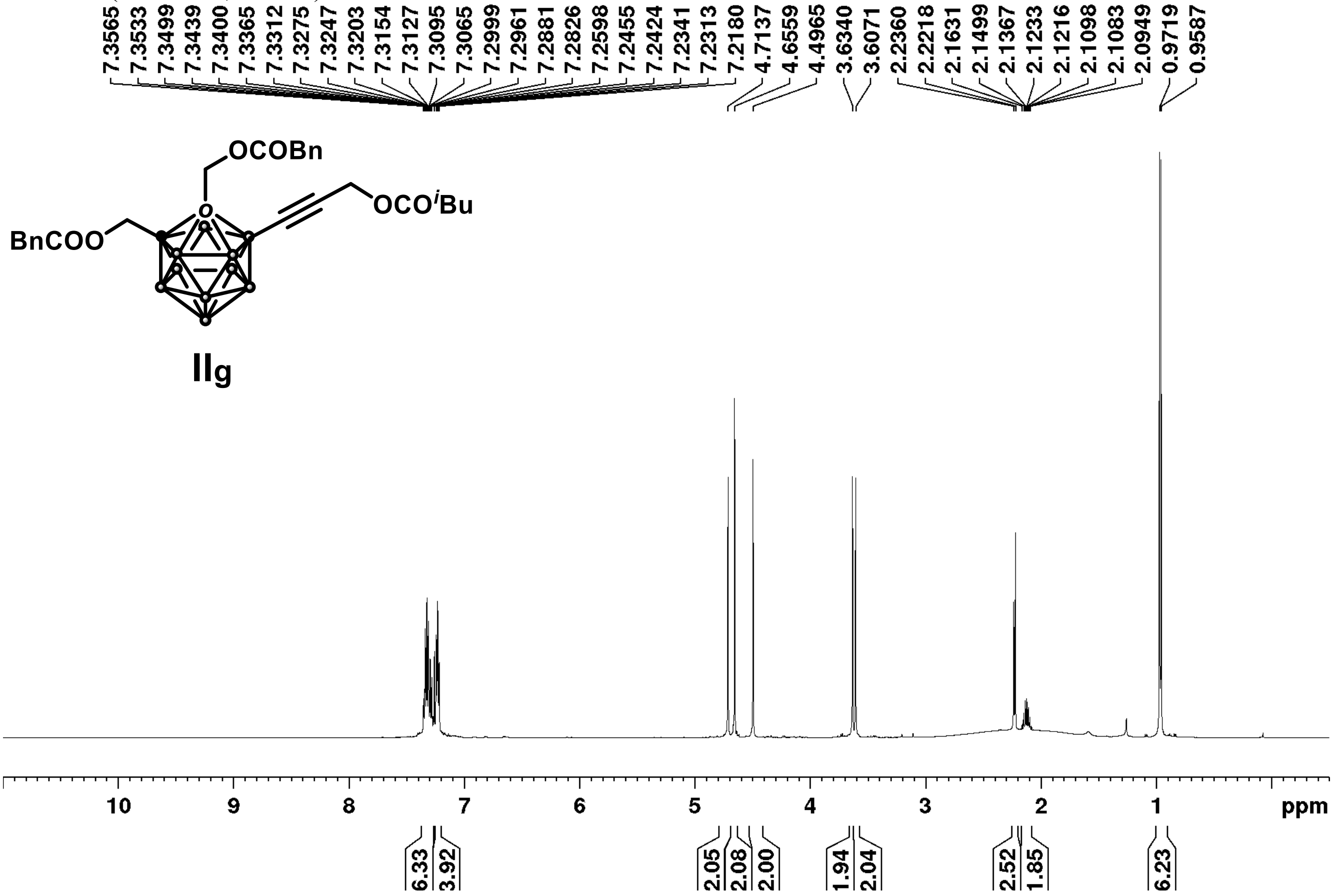

^{13}C NMR (125 MHz; $CDCl_3$)

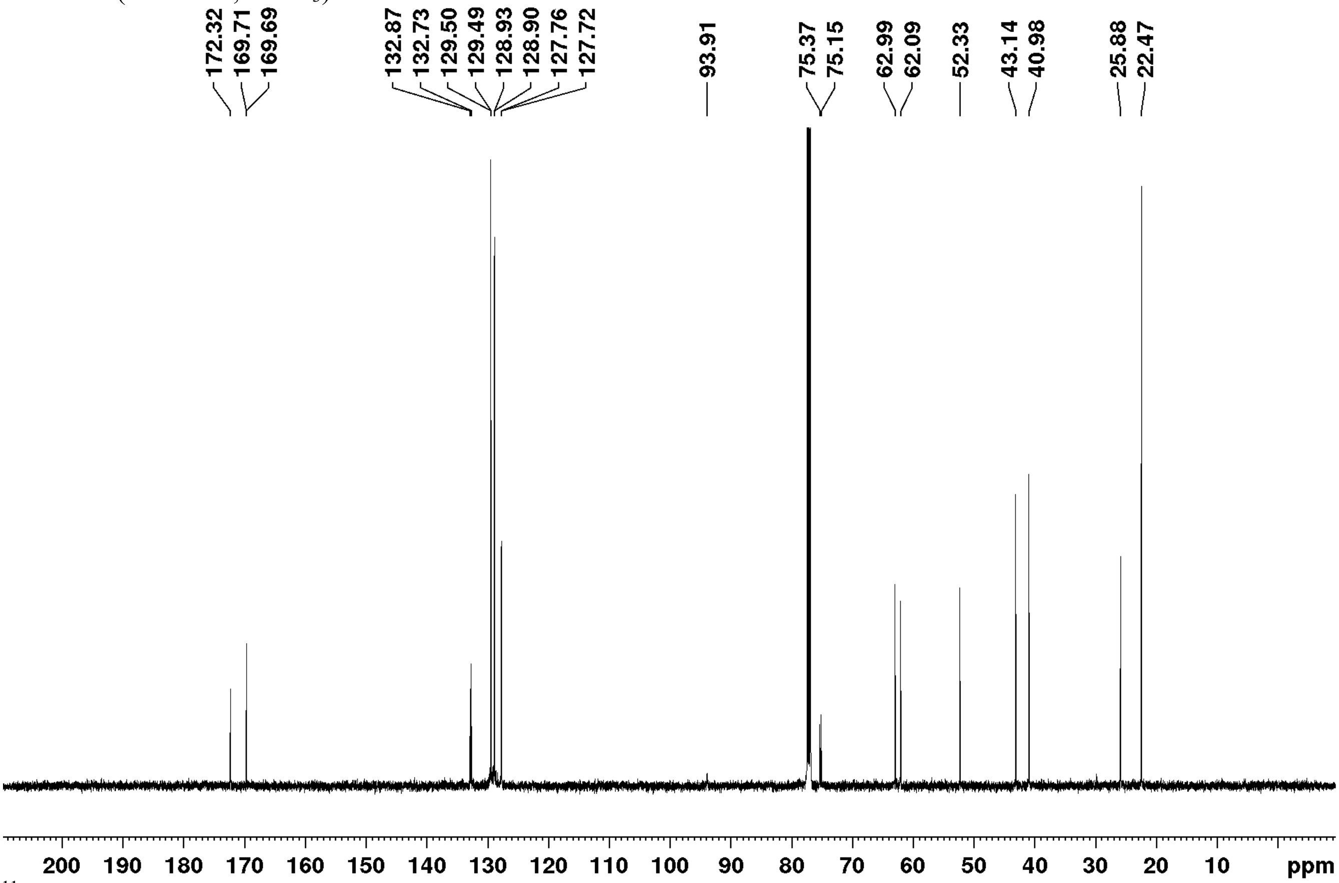

^{11}B NMR (160 MHz; $CDCl_3$)

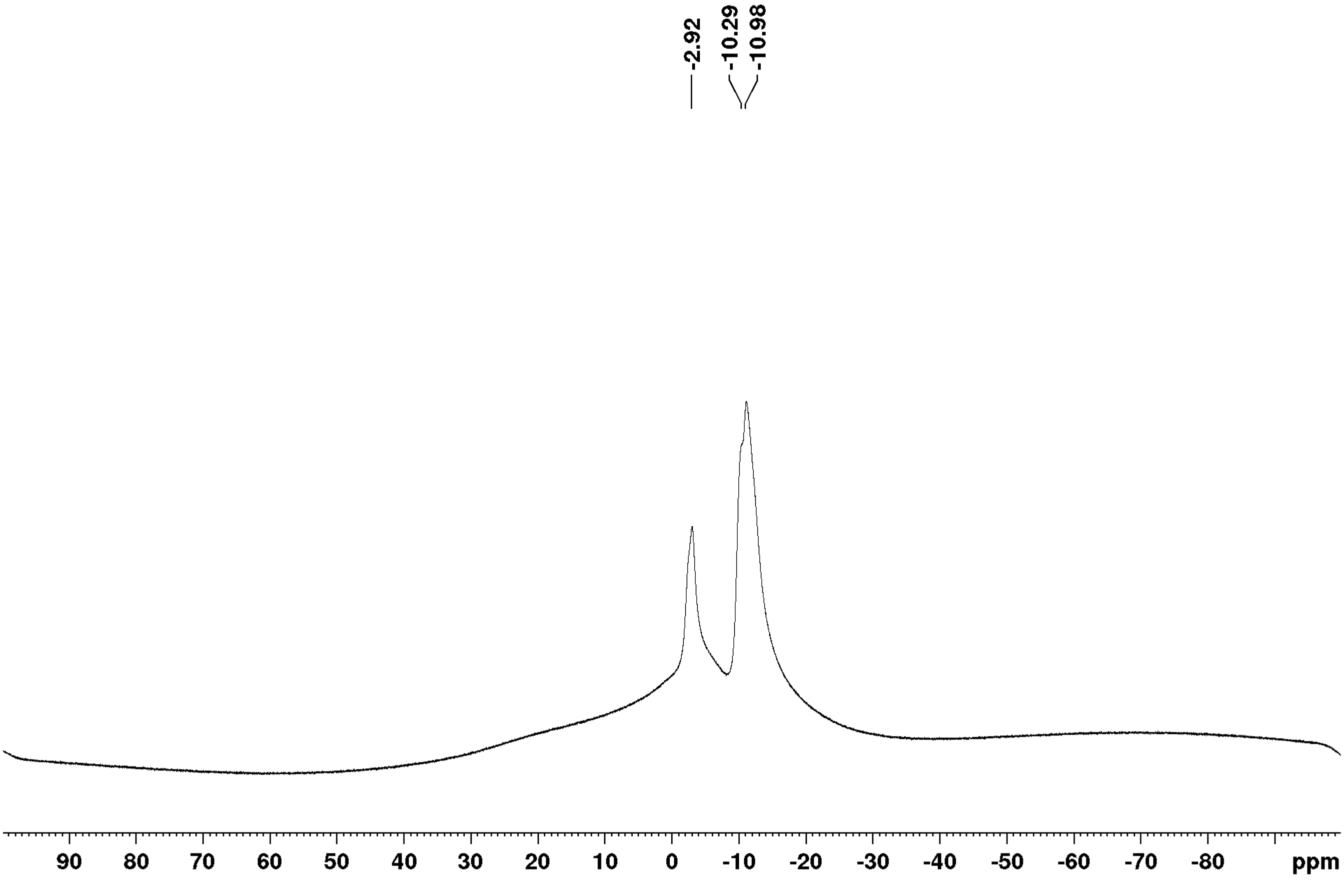

1,2-bis((3-methylbutanoyl)oxymethyl)-4-((3-methylbutanoyl)prop-1-yn-1-yl)-1,2-dicarba-*closo*-dodecaborane (IIh)

^{1}H NMR (500 MHz; CDCl$_3$)

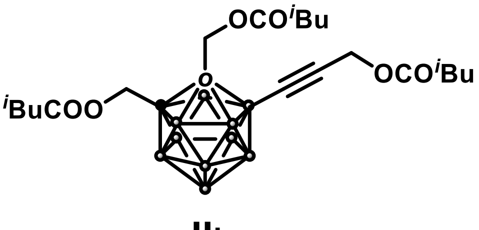

IIh

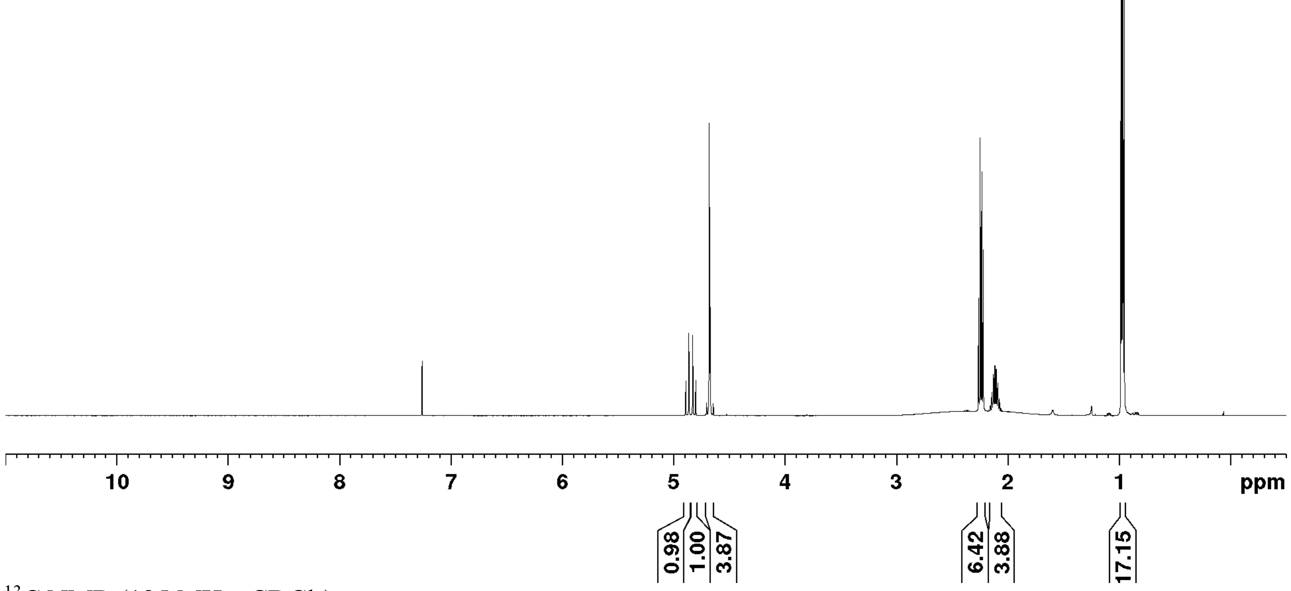

^{13}C NMR (125 MHz; CDCl$_3$)

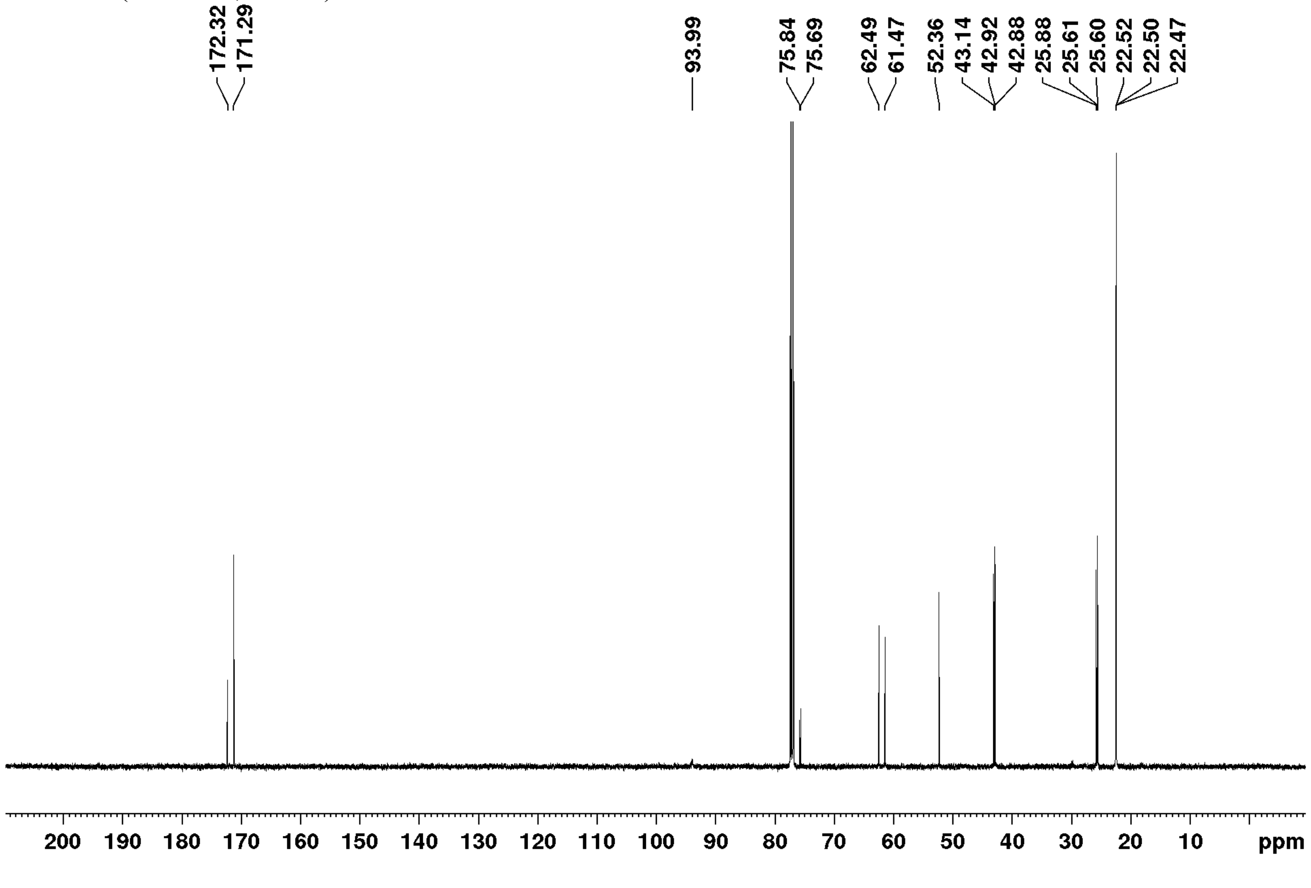

^{11}B NMR (160 MHz; $CDCl_3$)

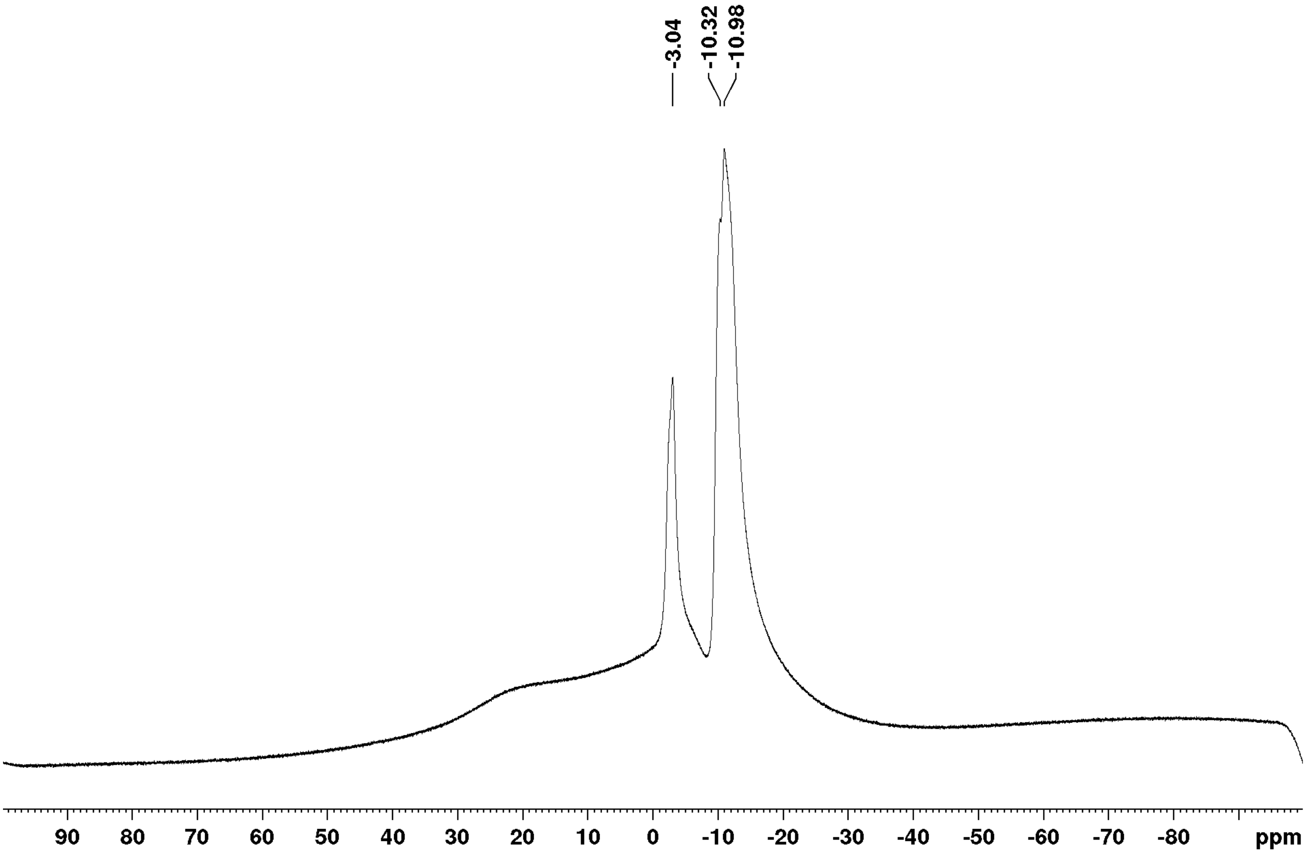

1-hydroxymethyl-9-((trimethylsilyl)ethynyl)-1,7-dicarba-*closo*-dodecaborane (S5)

^{1}H NMR (500 MHz; $CDCl_3$)

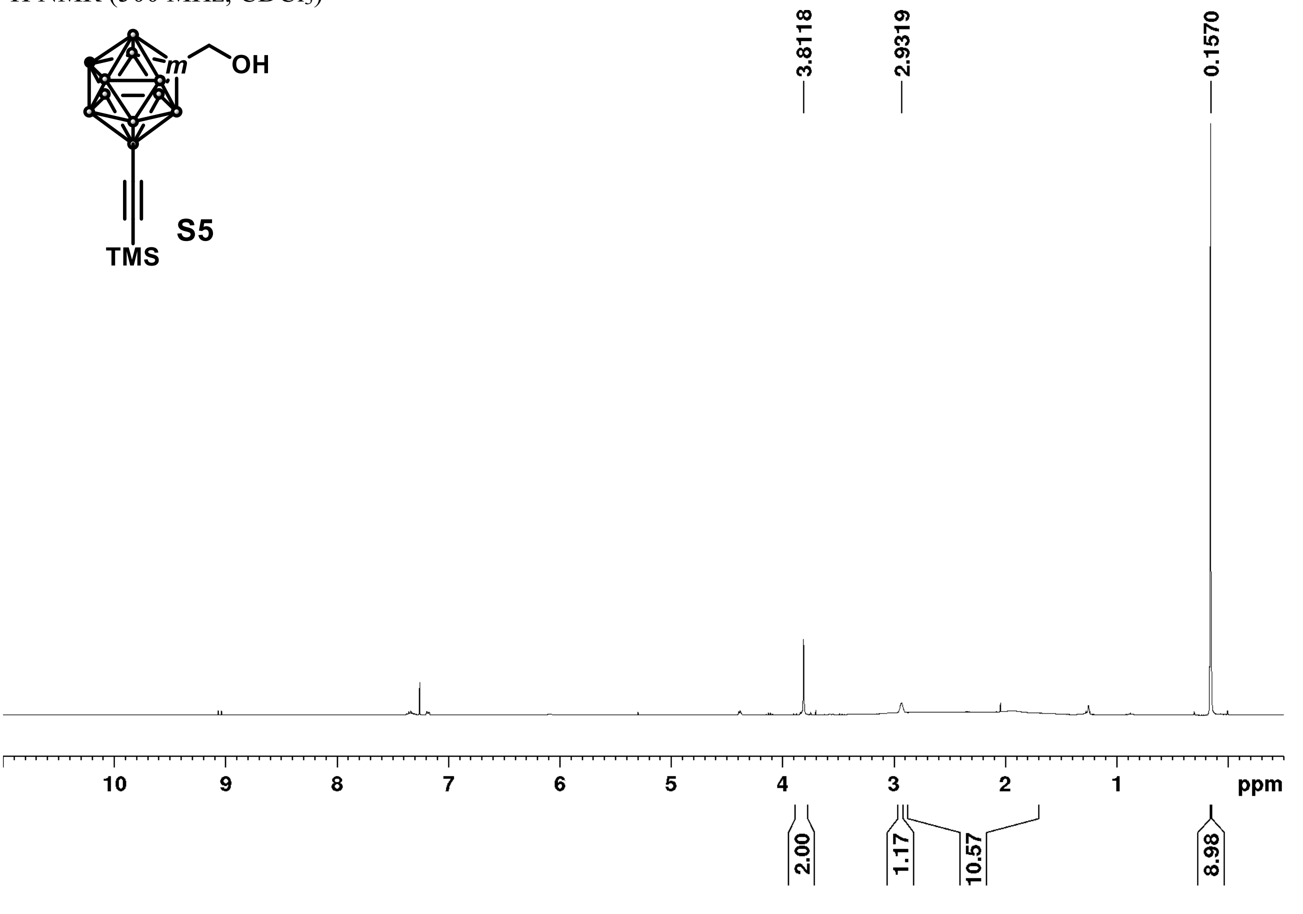

^{13}C NMR (125 MHz; $CDCl_3$)

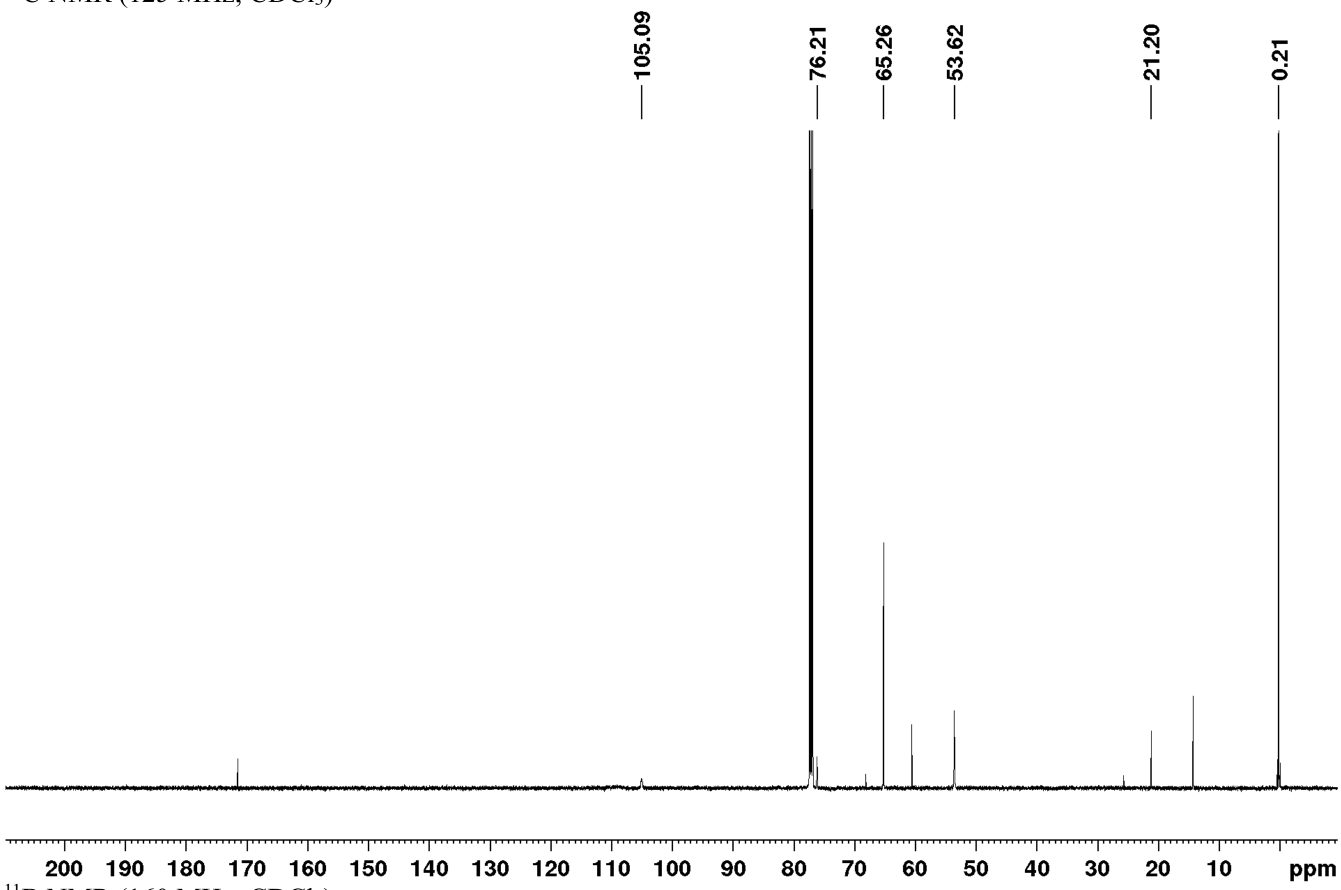

^{11}B NMR (160 MHz; $CDCl_3$)

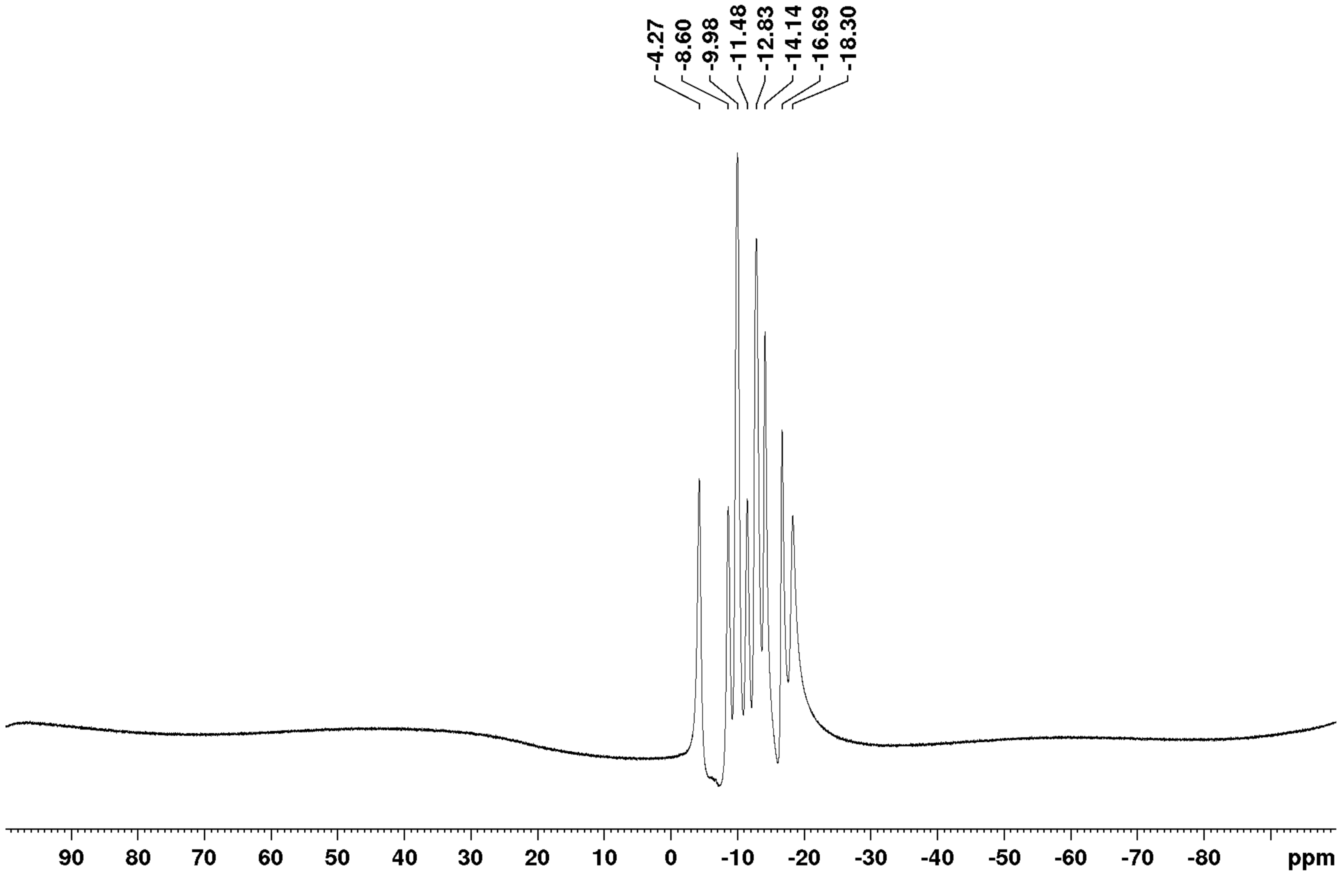

1-((*tert*-butyl)dimethylsilyl)hydroxymethyl)-9-((trimethylsilyl)ethynyl)-1,7-dicarba-*closo*-dodecaborane (17)

^{1}H NMR (500 MHz; $CDCl_3$)

17

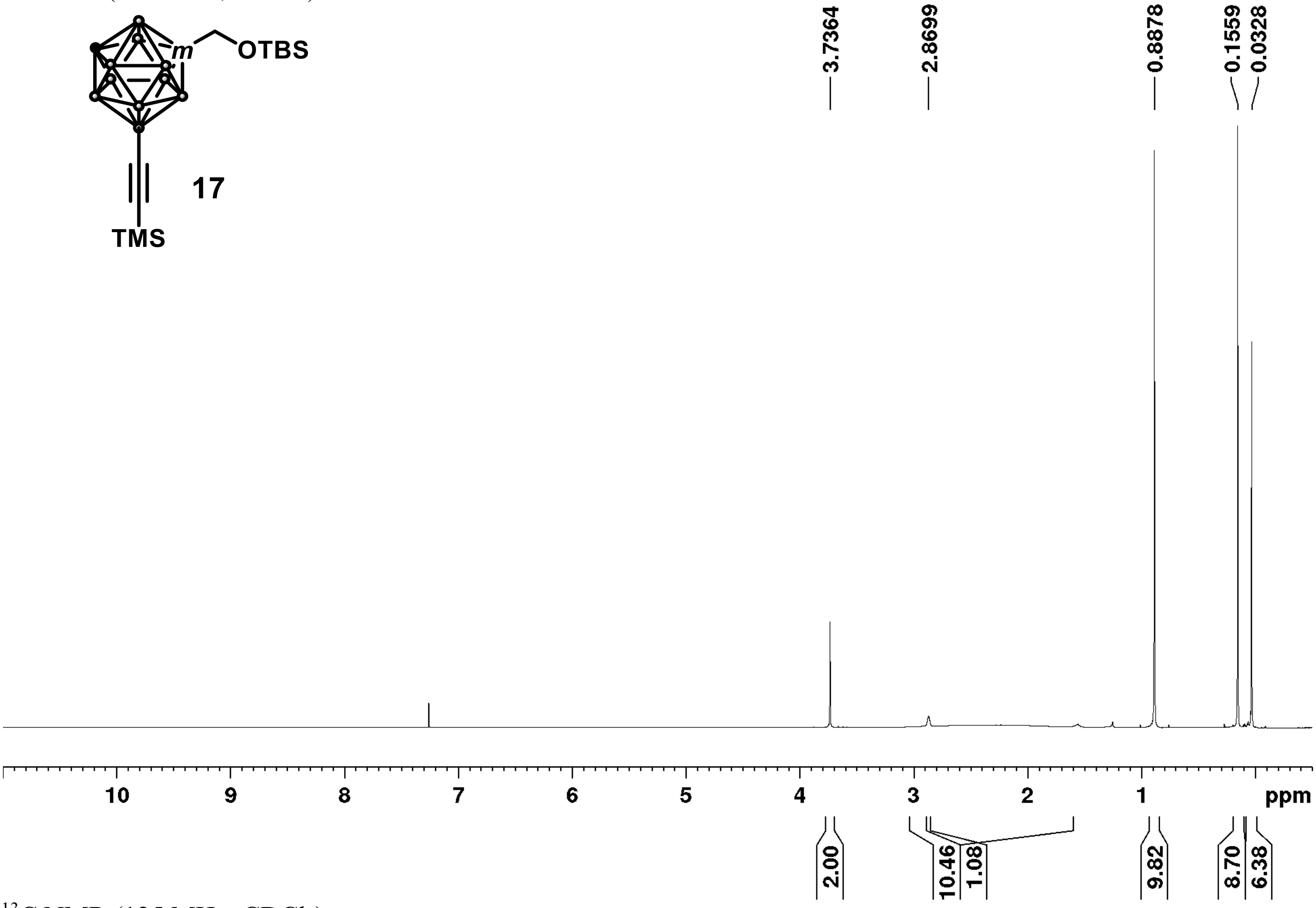

^{13}C NMR (125 MHz; $CDCl_3$)

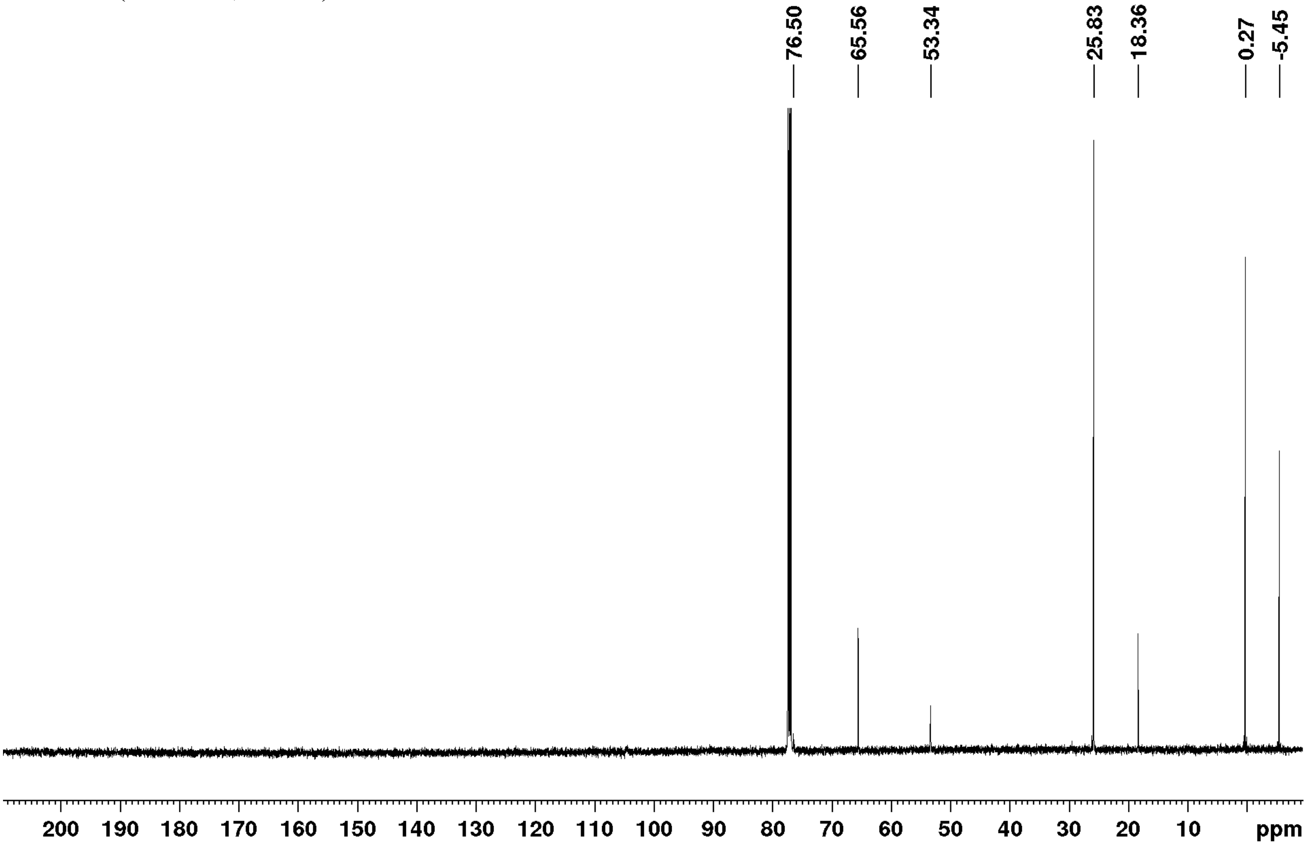

^{11}B NMR (160 MHz; $CDCl_3$)

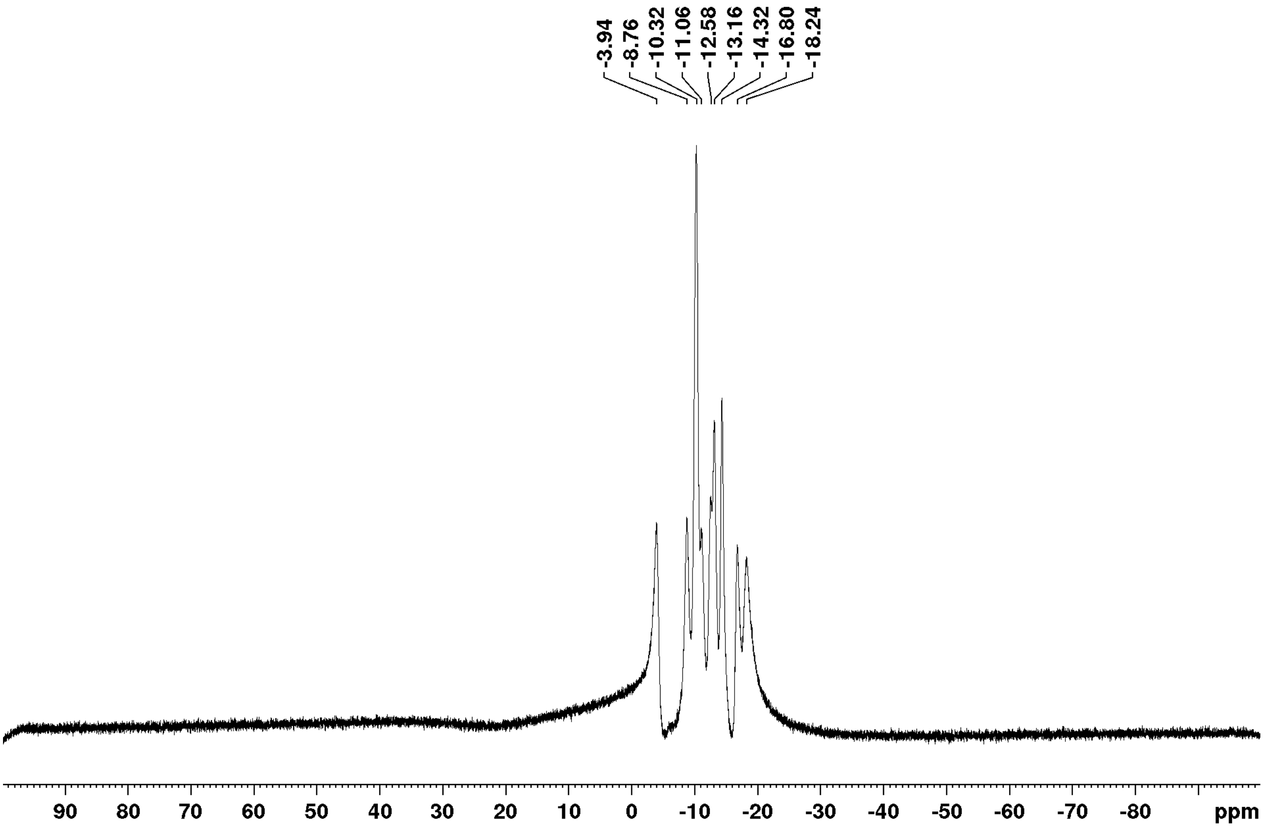

7-((*tert*-butyl)dimethylsilyl)hydroxymethyl-9-(trimethylsilyl)ethynyl-1,7-dicarba-*closo*-dodecaboranylcarboxylic acid (18)

^{1}H NMR (500 MHz; $CDCl_3$)

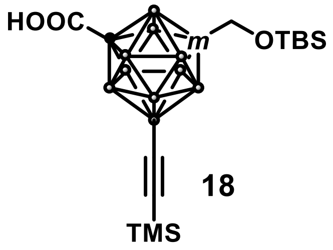

18

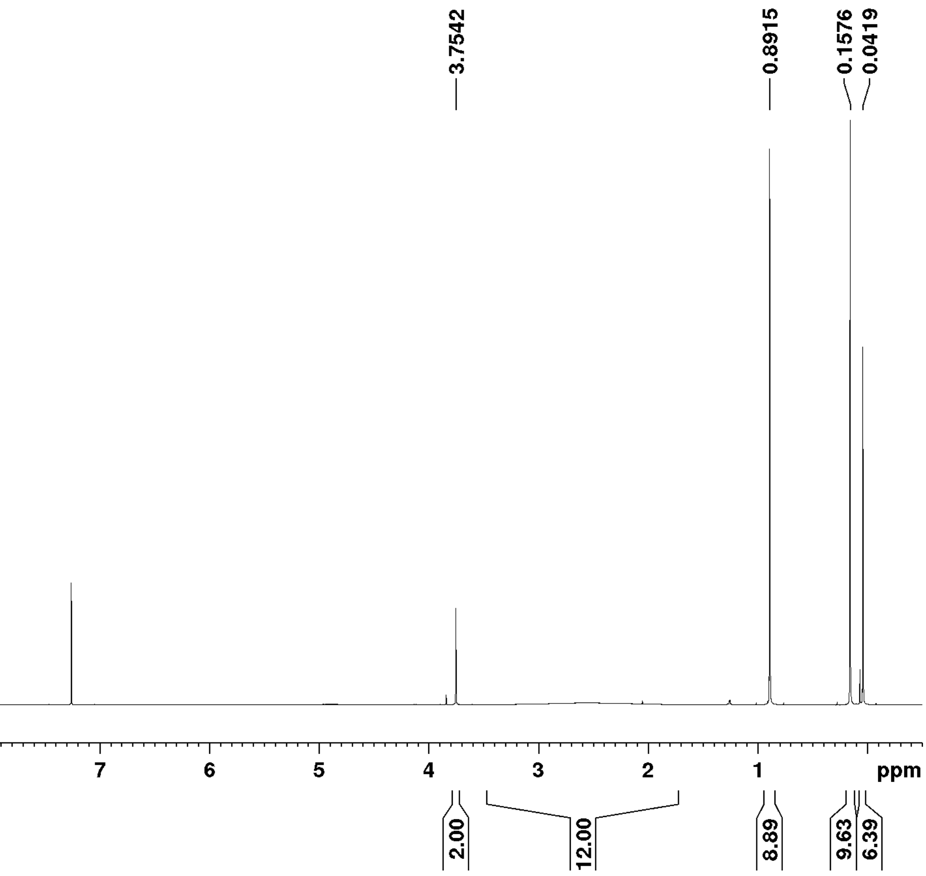

^{13}C NMR (125 MHz; $CDCl_3$)

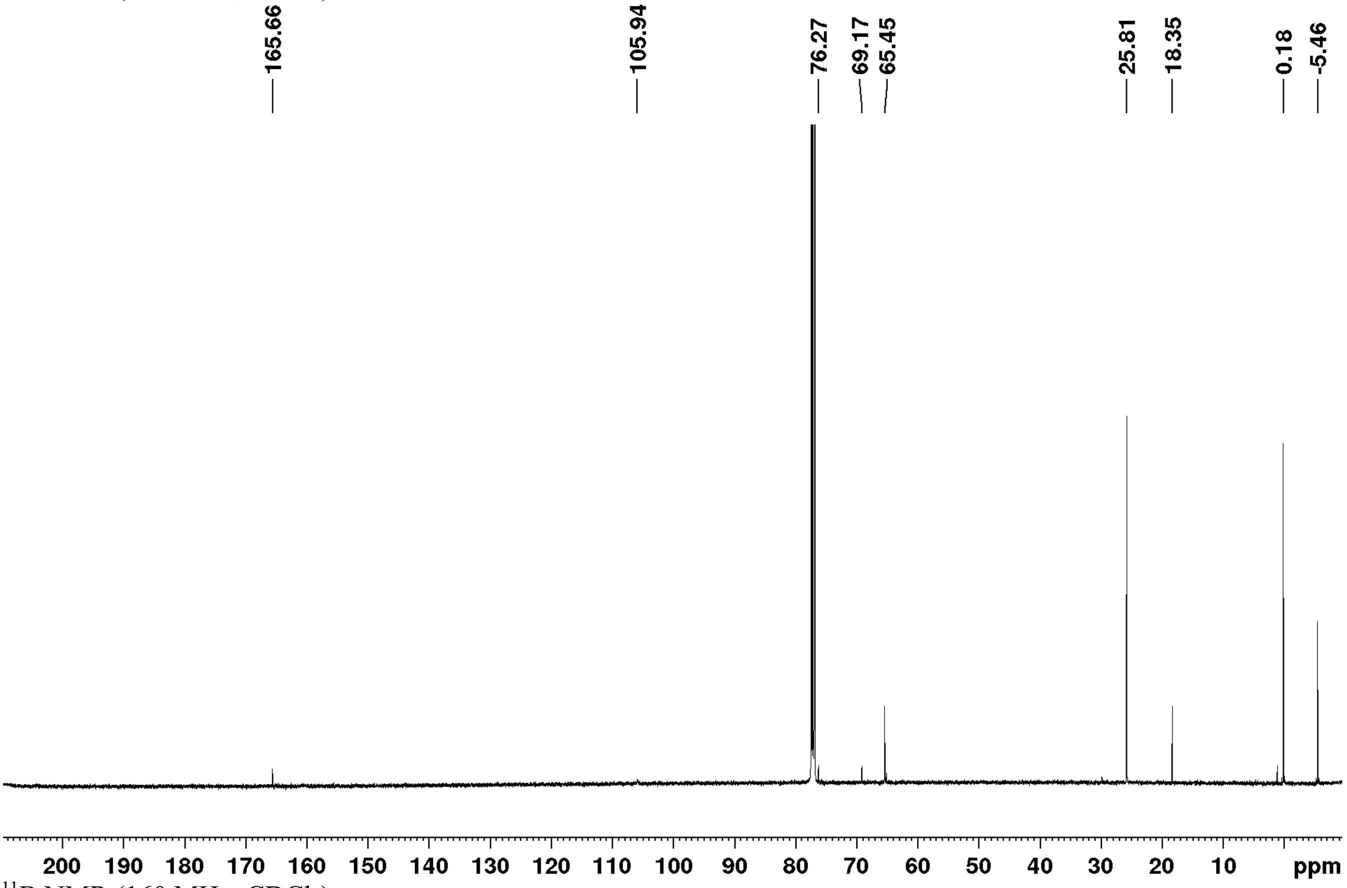

^{11}B NMR (160 MHz; $CDCl_3$)

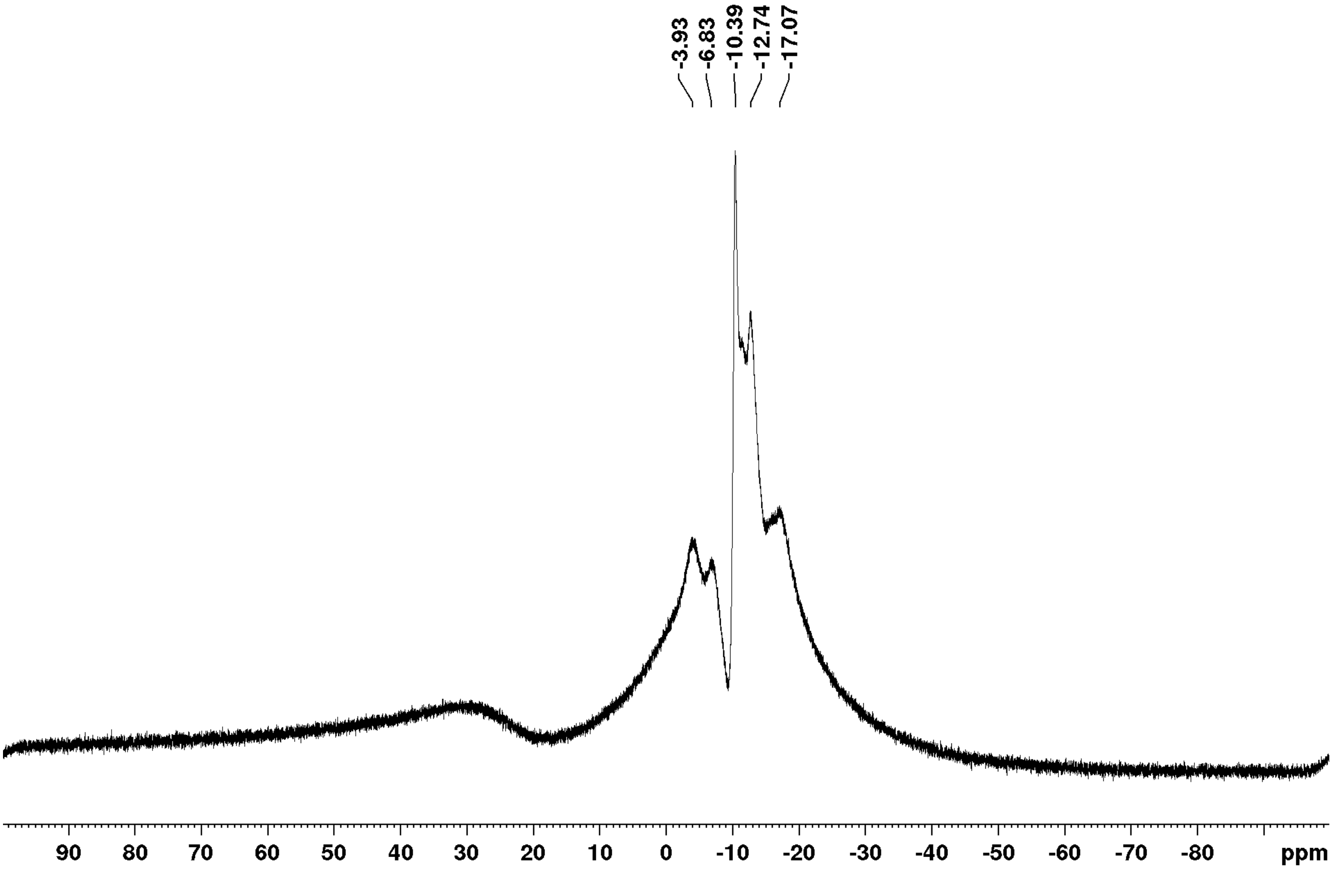

1-((*tert*-butyl)dimethylsilyl)hydroxymethyl)-7-benzylaminocarboxyl-9-ethynyl-1,7-dicarba-*closo*-dodecaborane (19a)

^{1}H NMR (500 MHz; $CDCl_3$)

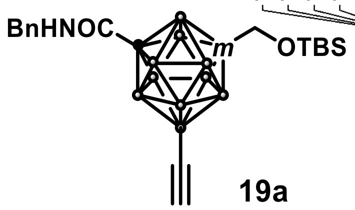

19a

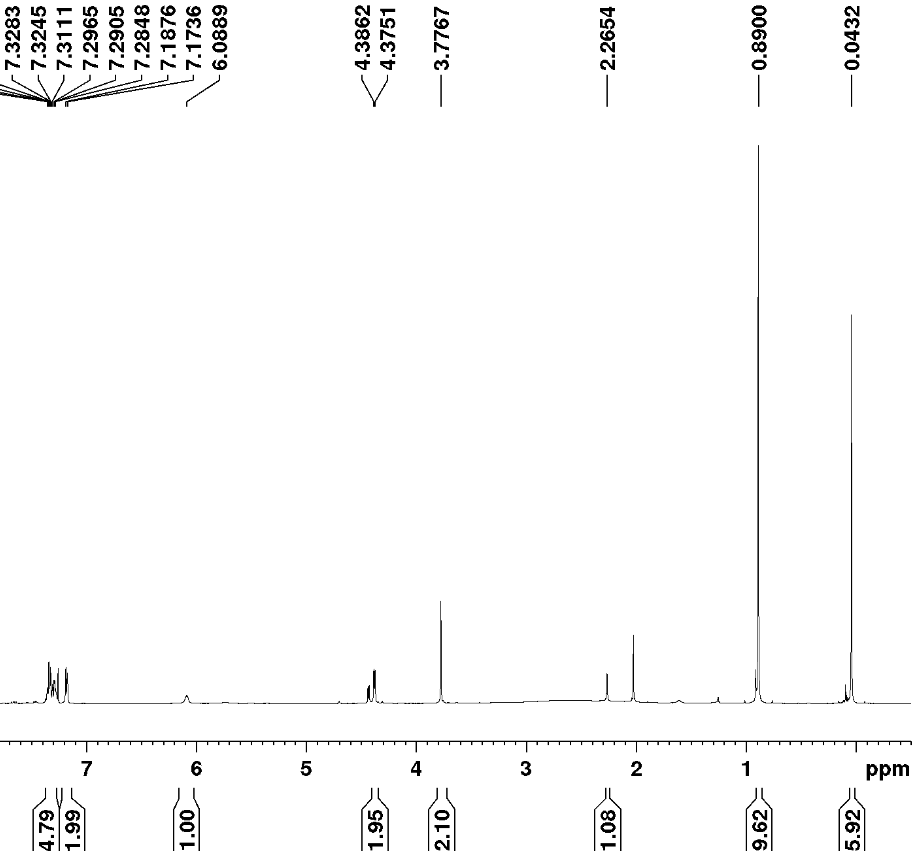

^{13}C NMR (125 MHz; $CDCl_3$)

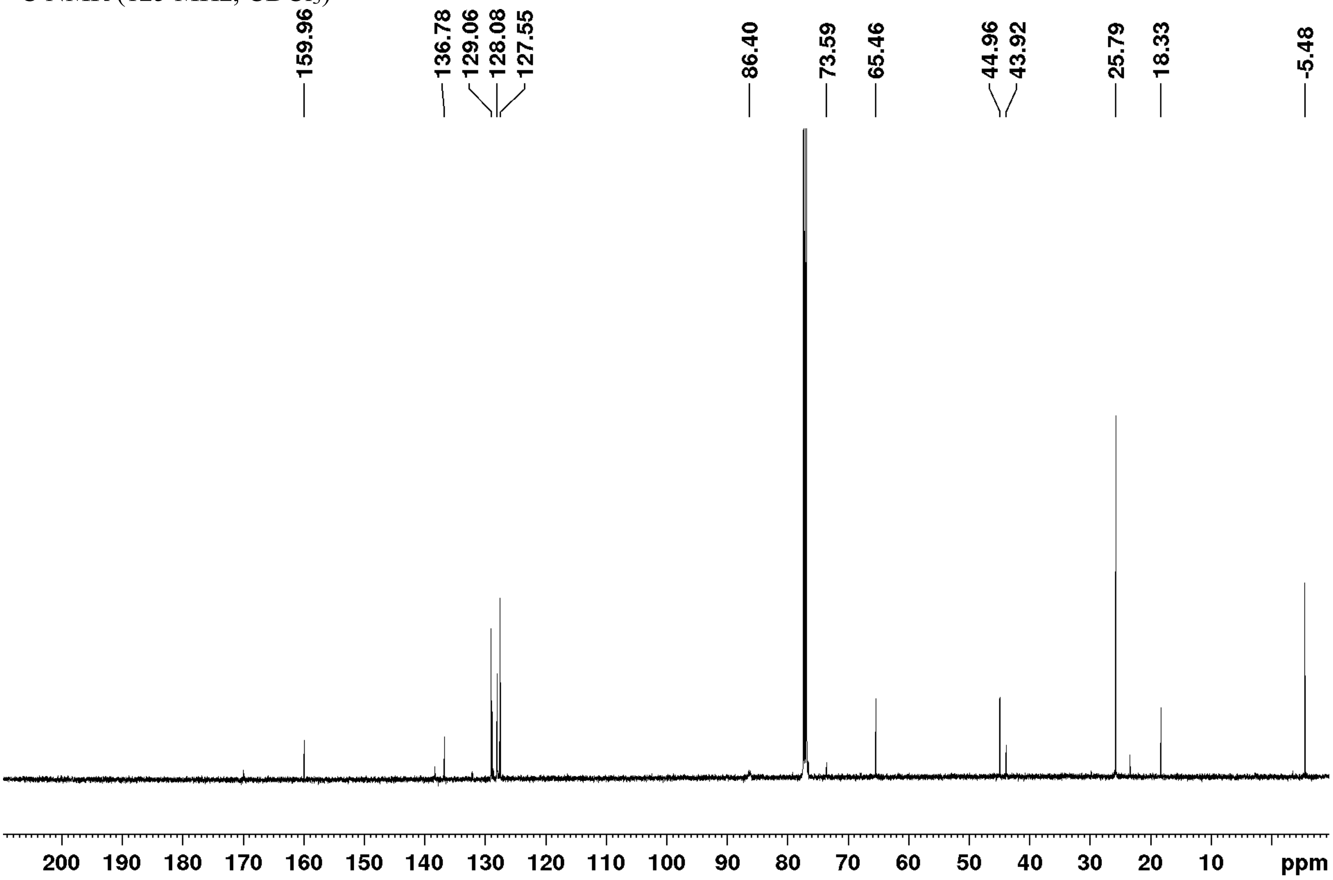

^{11}B NMR (160 MHz; $CDCl_3$)

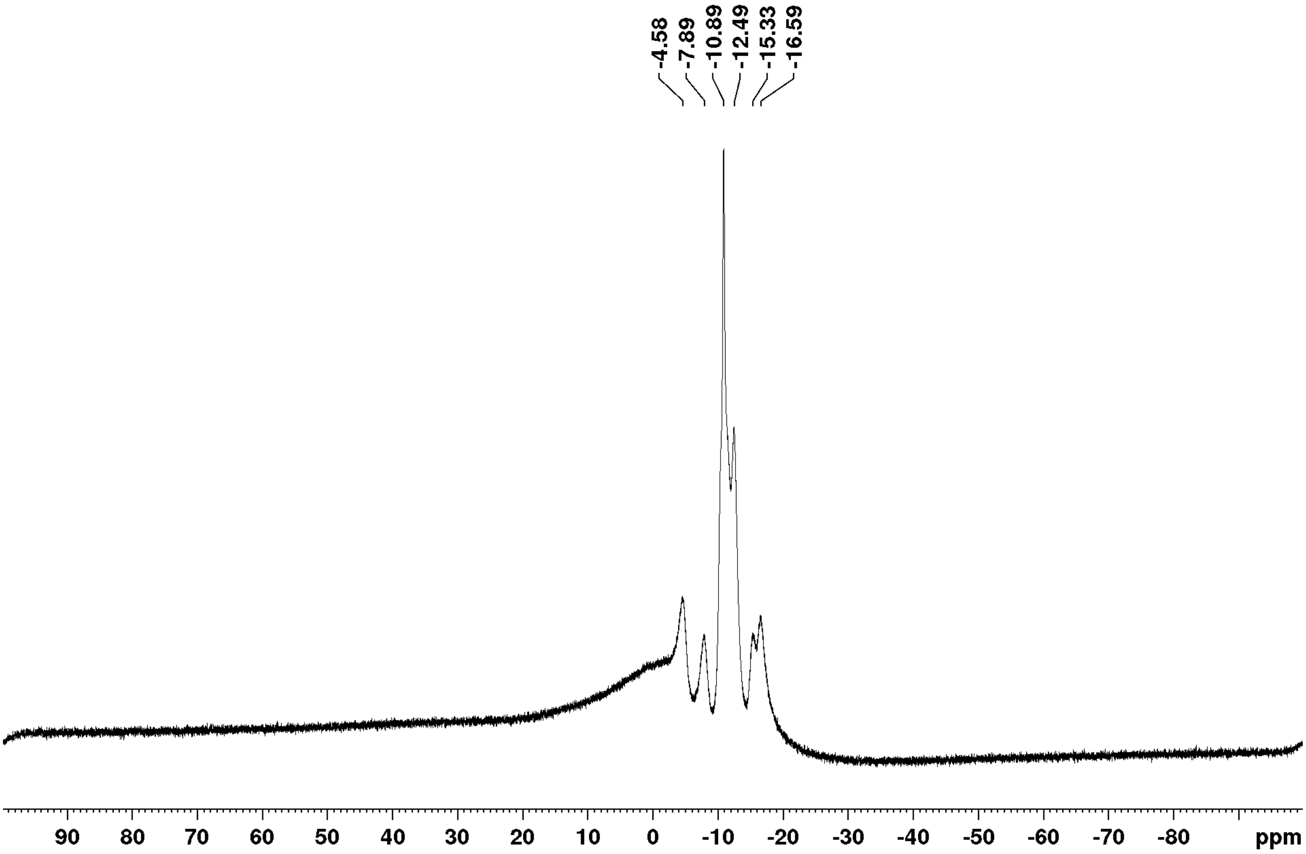

Synthesis of 1-((*tert*-butyl)dimethylsilyl)hydroxymethyl-7-isobutylcarbamoyl-9-ethynyl-1,7-dicarba-*closo*-dodecaborane (19b)

^{1}H NMR (500 MHz; $CDCl_3$)

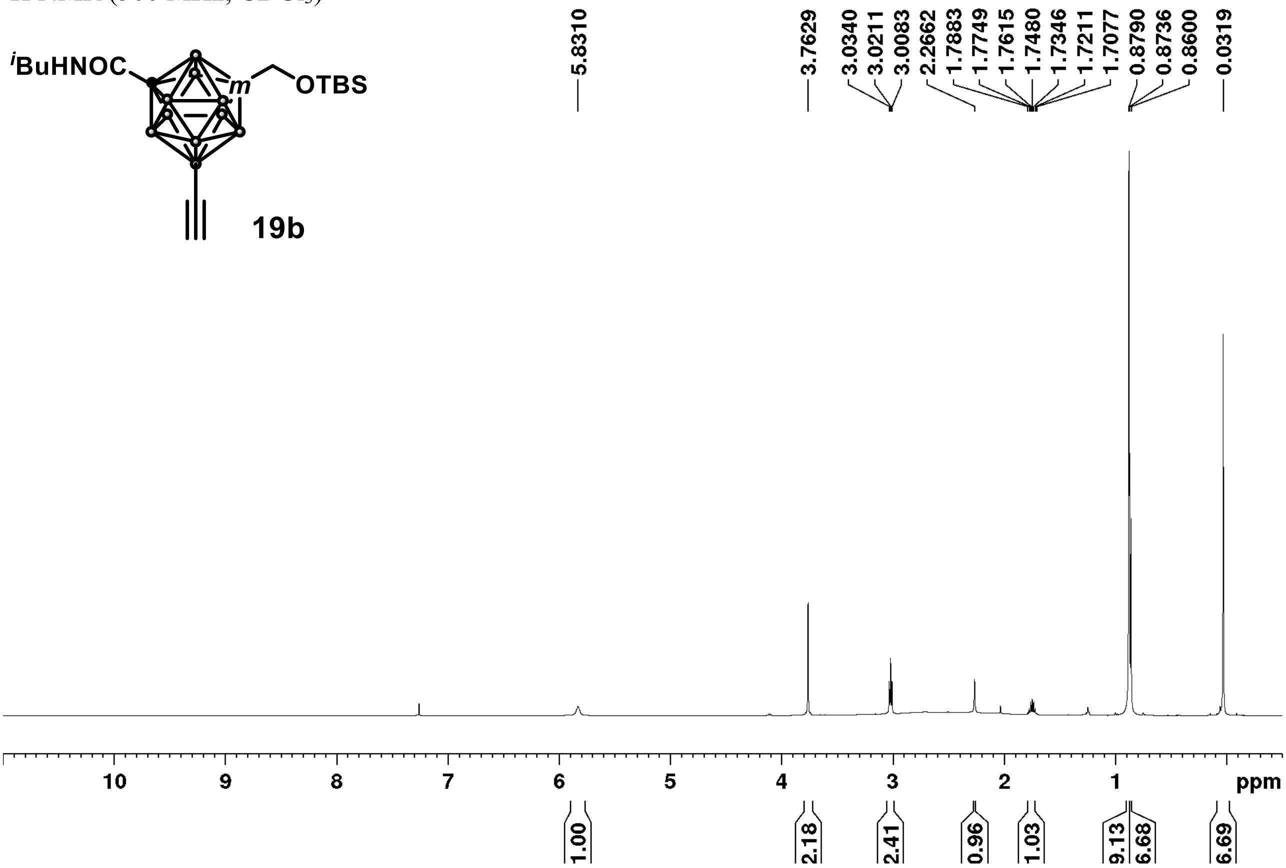

^{13}C NMR (125 MHz; $CDCl_3$)

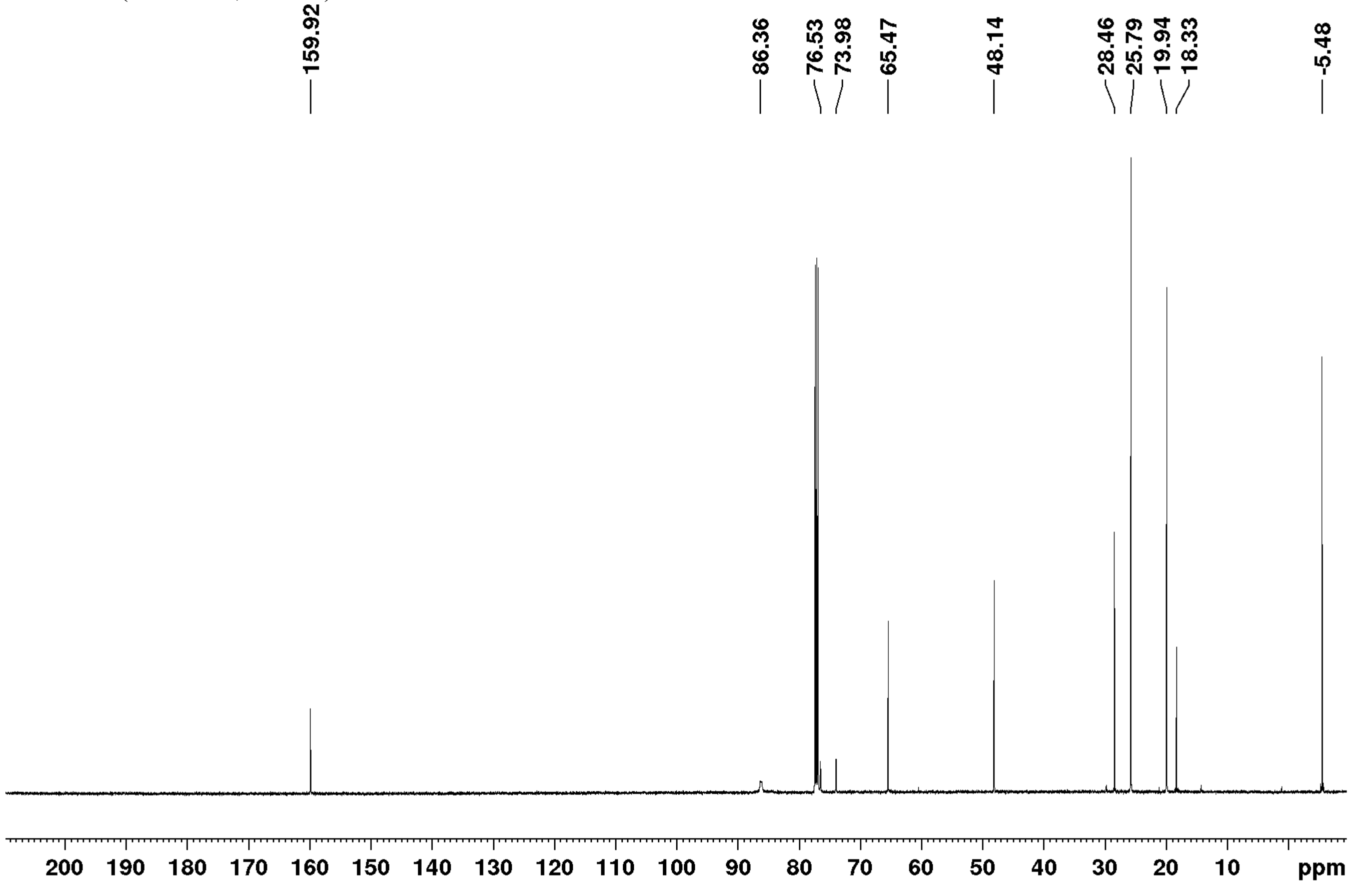

^{11}B NMR (160 MHz; $CDCl_3$)

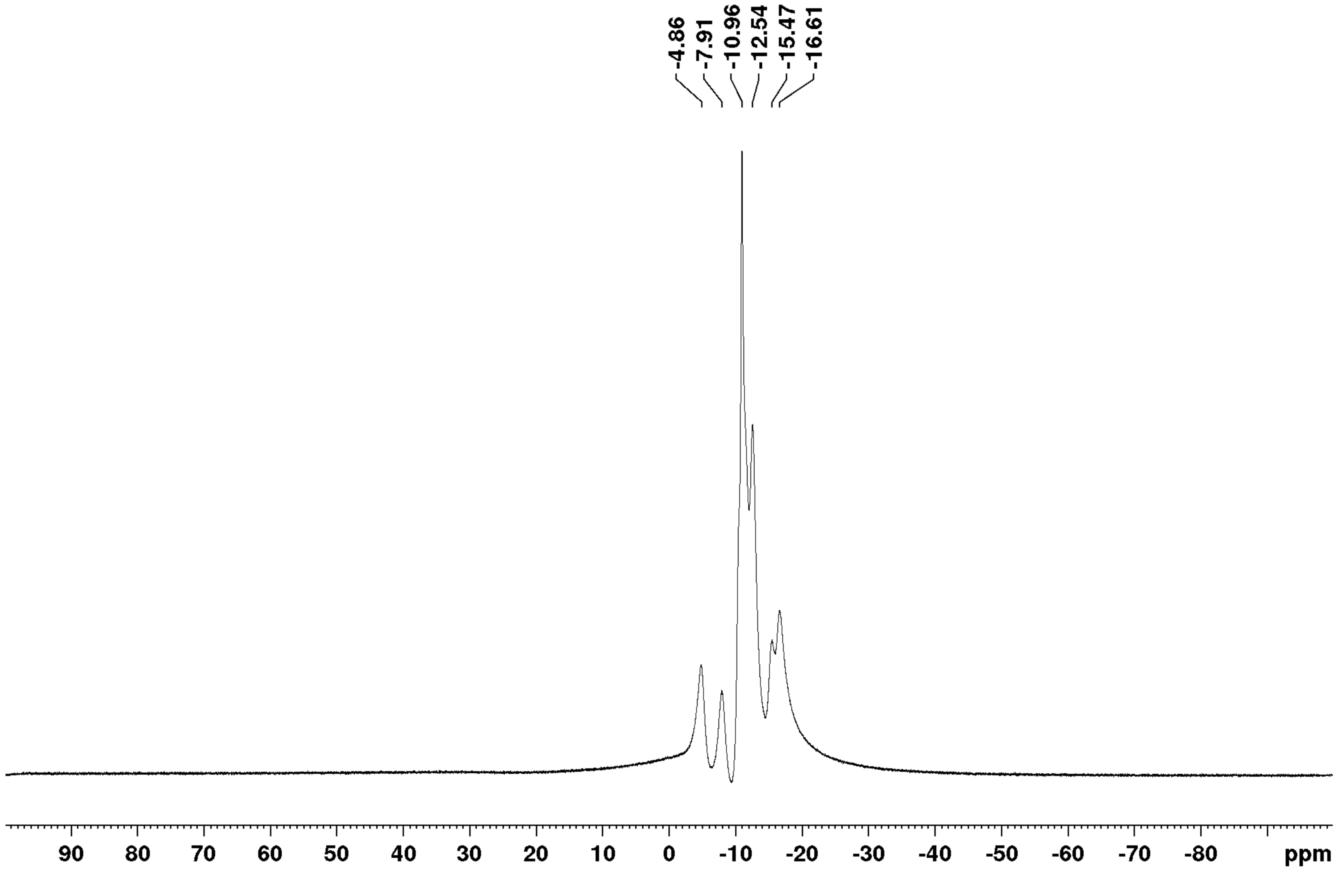

1-((*tert*-butyl)dimethylsilyl)hydroxymethyl)-7-benzylaminocarboxyl-9-(1*H*-1,2,3-triazol-4-yl)benzylcarbamoyl-1,7-dicarba-*closo*-dodecaborane (20a)

^{1}H NMR (500 MHz; $CDCl_3$)

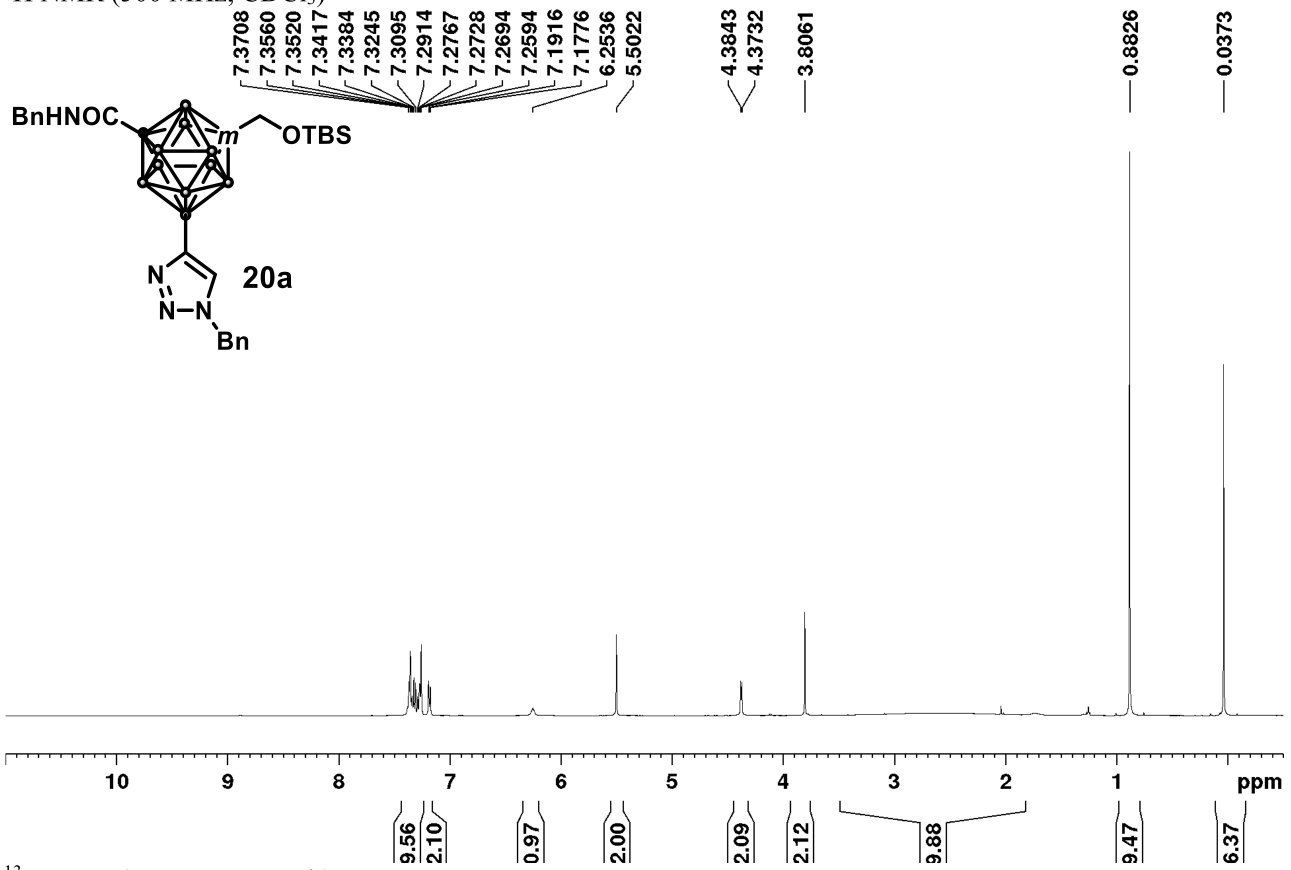

^{13}C NMR (125 MHz; $CDCl_3$)

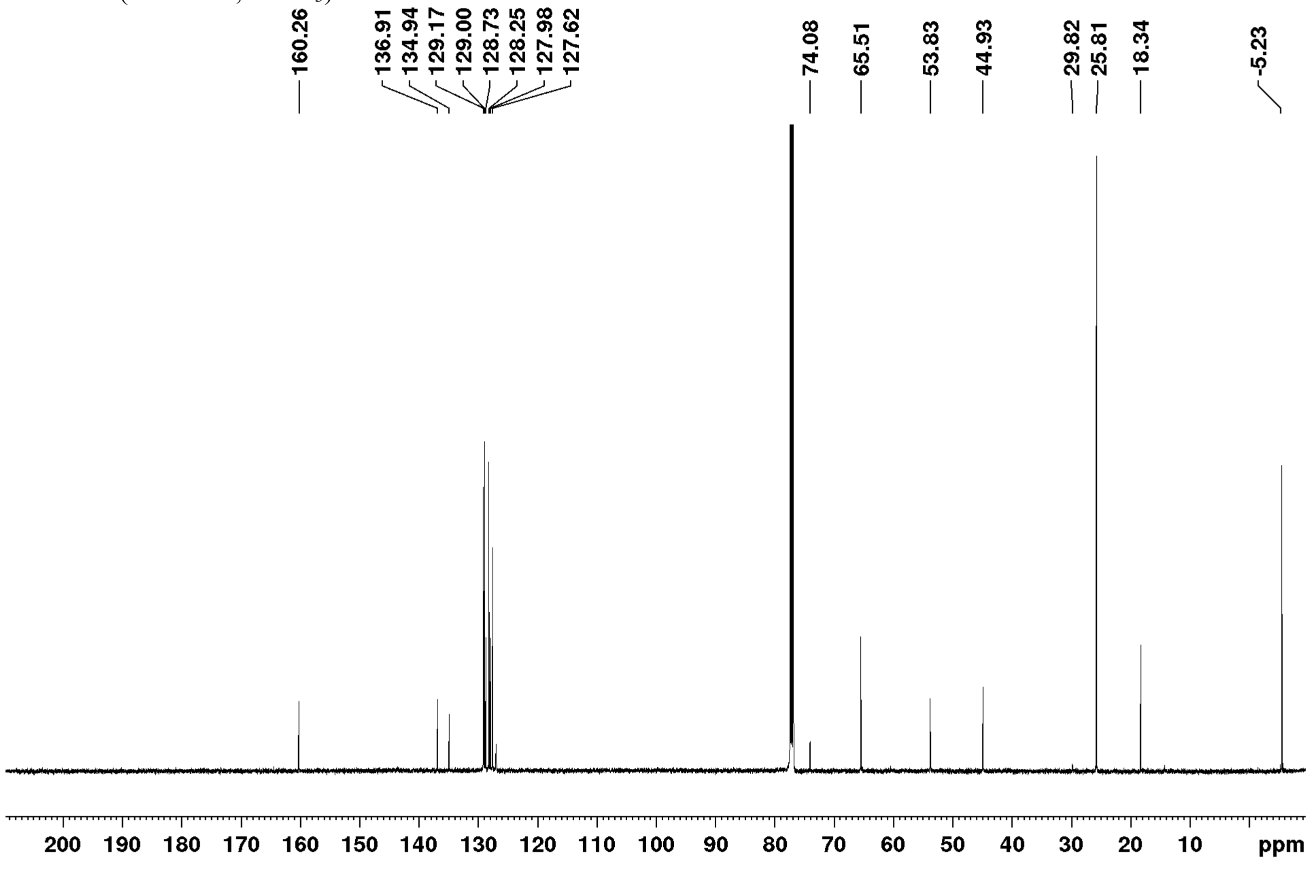

^{11}B NMR (160 MHz; $CDCl_3$)

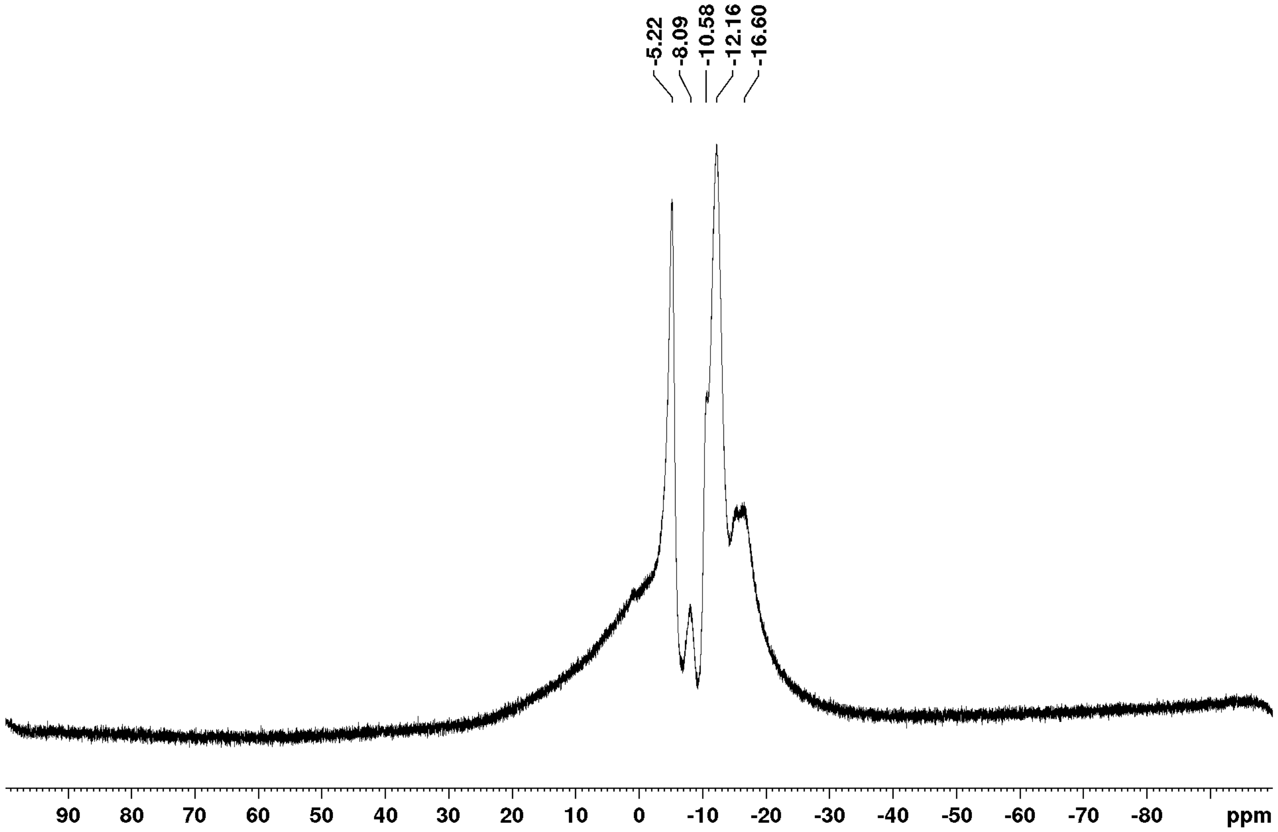

1-((*tert*-butyl)dimethylsilyl)hydroxymethyl)-7-benzylaminocarboxyl-9-((1*H*-1,2,3-triazol-4-yl)-3-methyl-butanoyl)-1,7-dicarba-*closo*-dodecaborane (20b)

^{1}H NMR (500 MHz; $CDCl_3$)

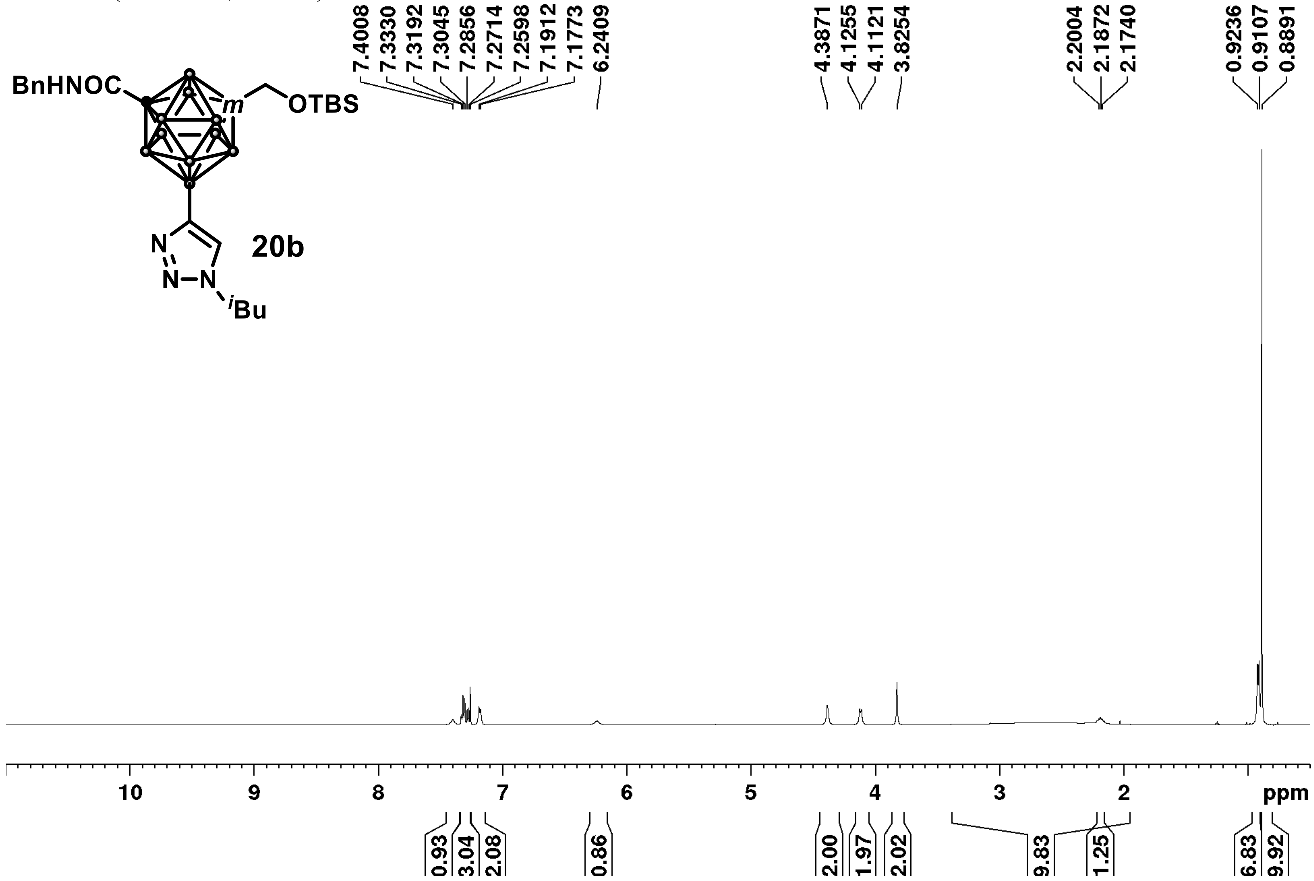

^{13}C NMR (125 MHz; $CDCl_3$)

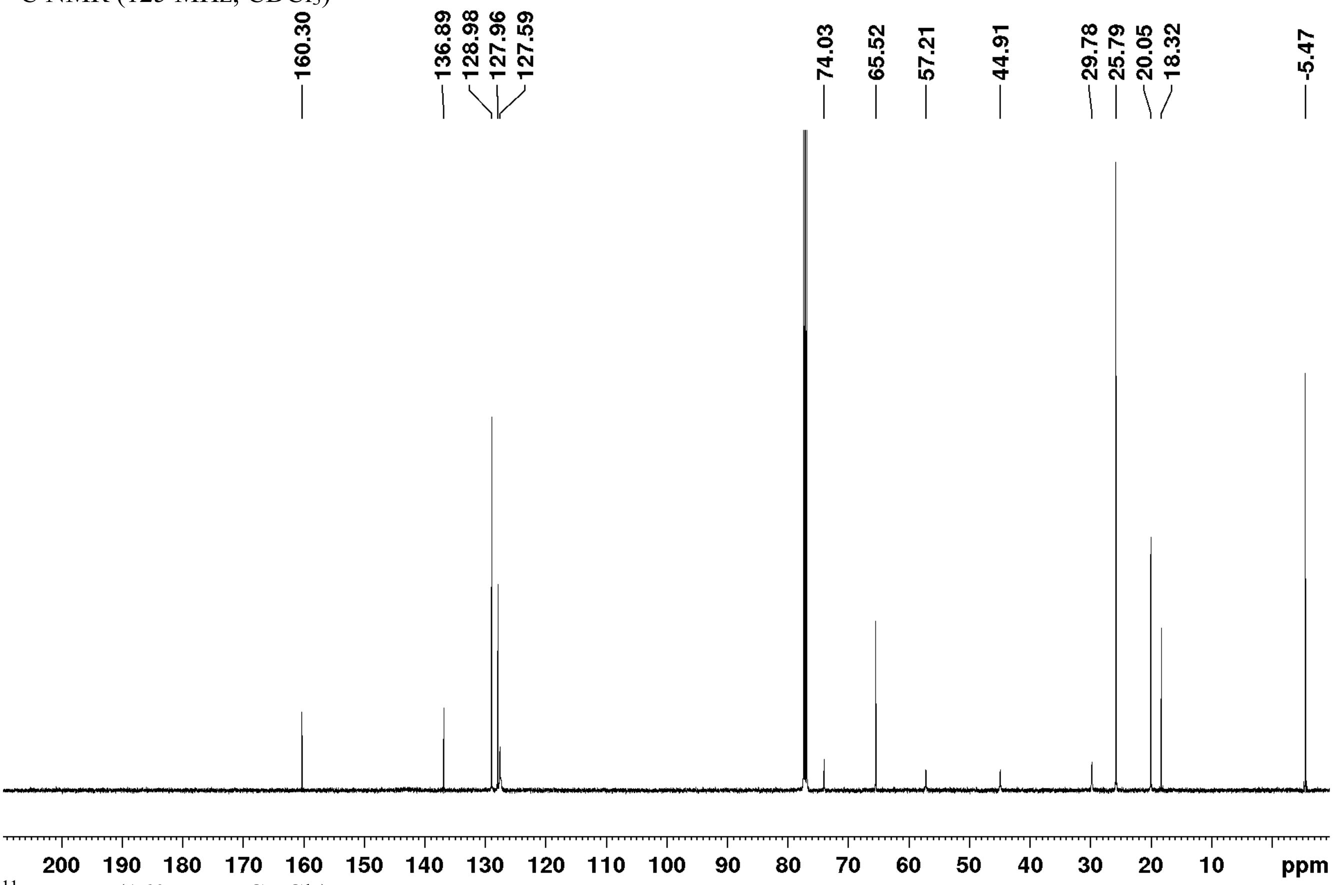

^{11}B NMR (160 MHz; $CDCl_3$)

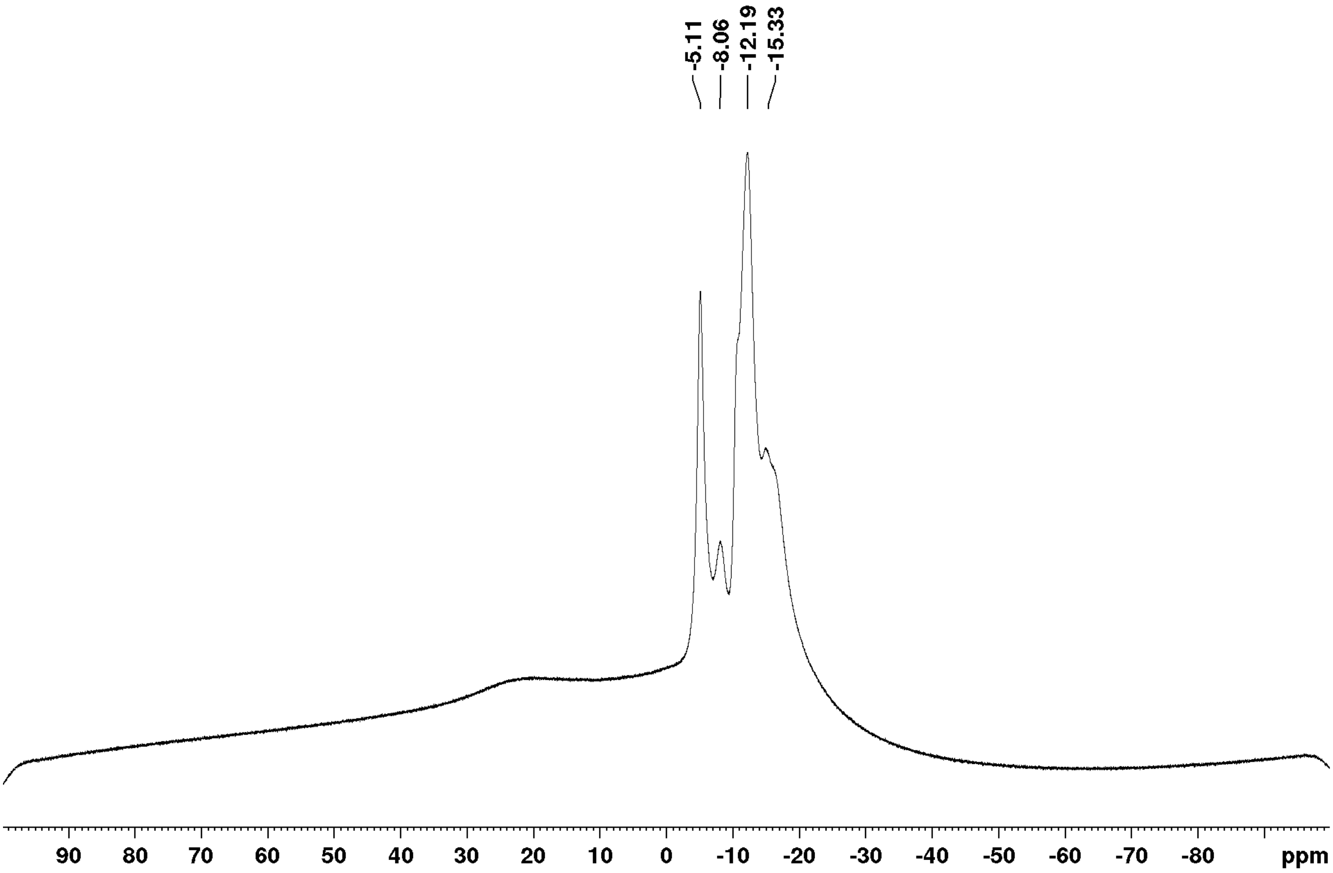

1-((*tert*-butyl)dimethylsilyl)hydroxymethyl)-7-isobutylcarbamoyl-9-(1*H*-1,2,3-triazol-4-yl)benzylcarbamoyl-1,7-dicarba-*closo*-dodecaborane (20c)

^{1}H NMR (500 MHz; CDCl$_3$)

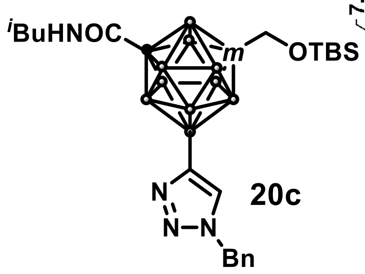

20c

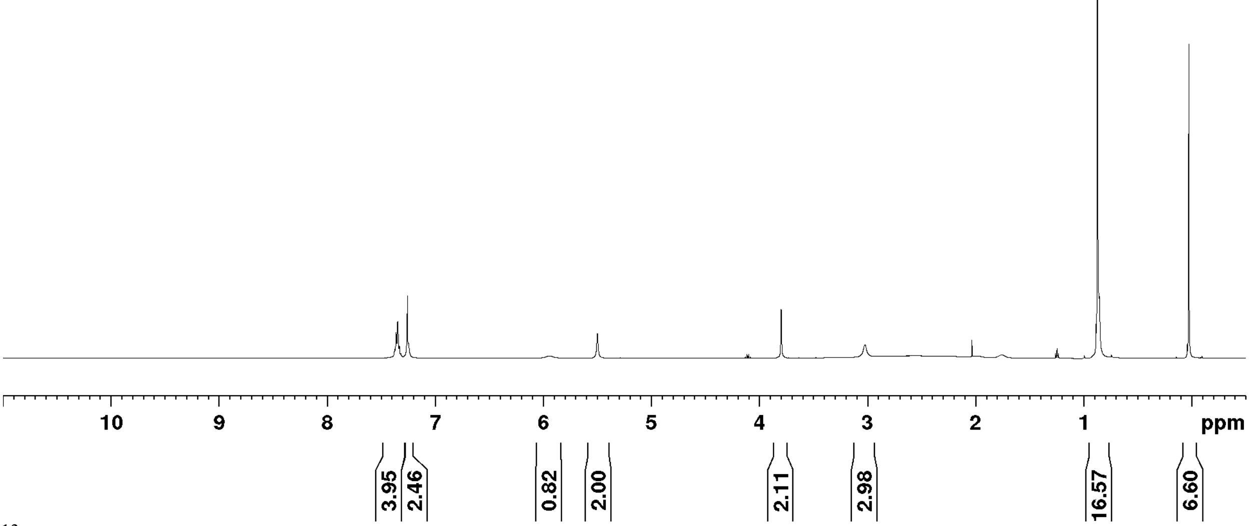

^{13}C NMR (125 MHz; CDCl$_3$)

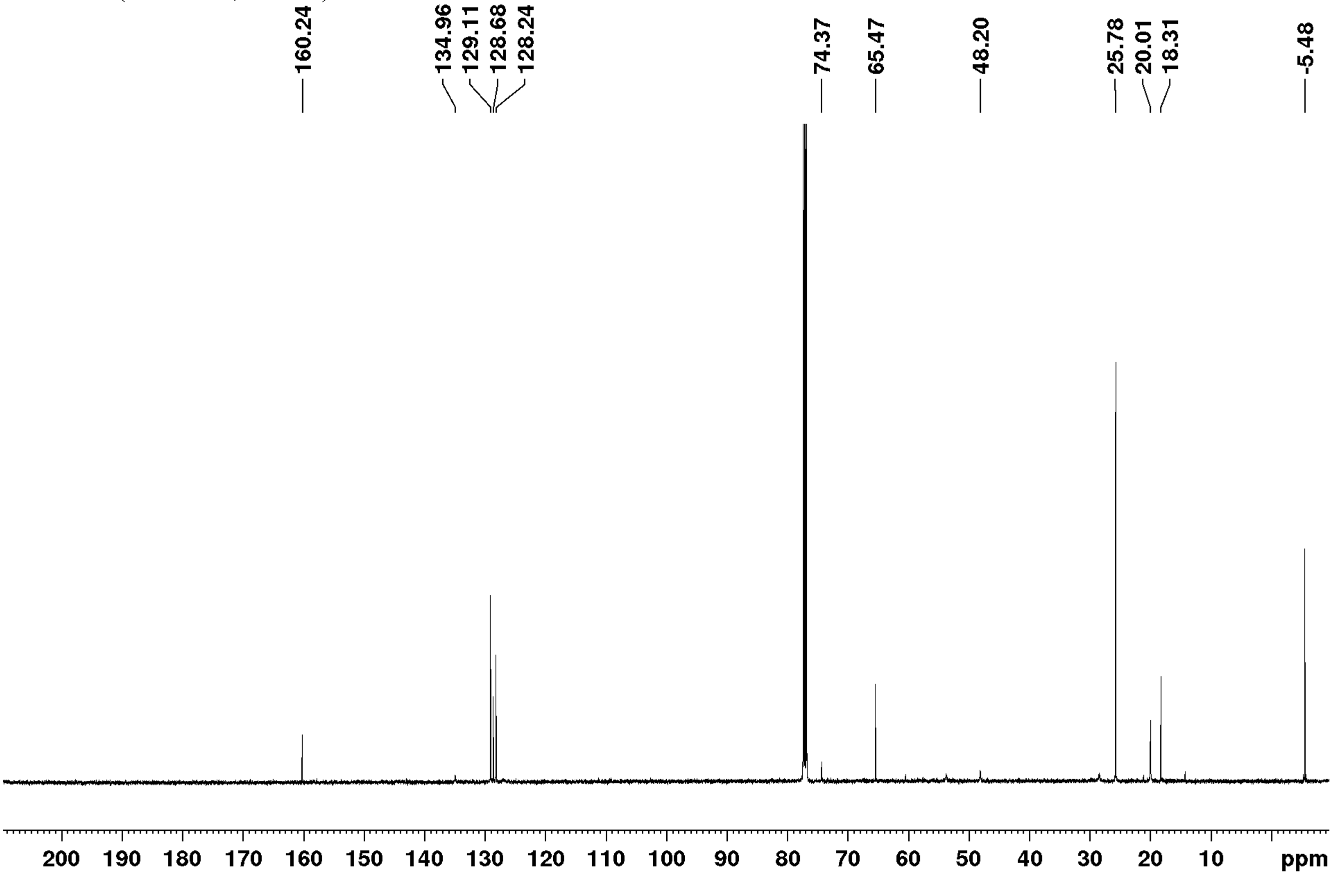

^{11}B NMR (160 MHz; $CDCl_3$)

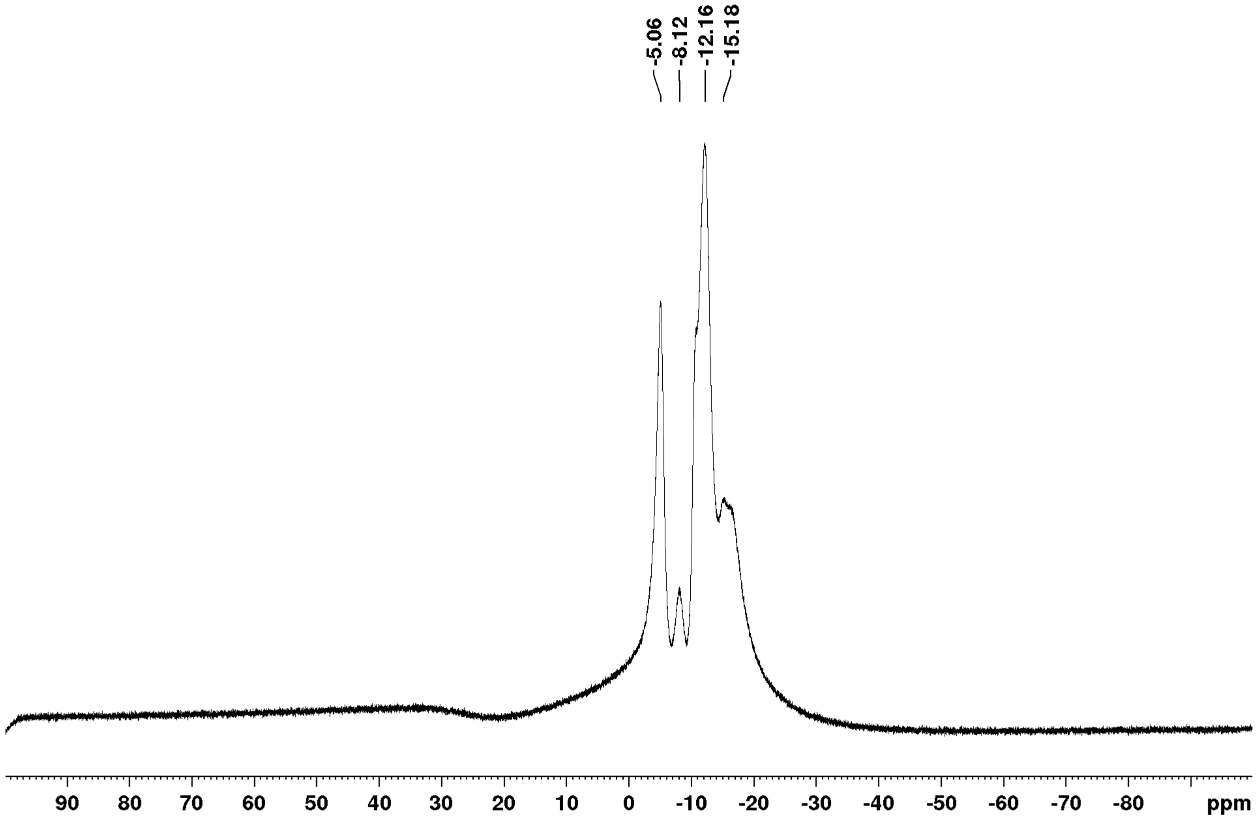

1-((*tert*-butyl)dimethylsilyl)hydroxymethyl)-7-isobutylcarbamoyl-9-((1*H*-1,2,3-triazol-4-yl)-3-methyl-butanoyl)-1,7-dicarba-*closo*-dodecaborane (20d)

^{1}H NMR (500 MHz; $CDCl_3$)

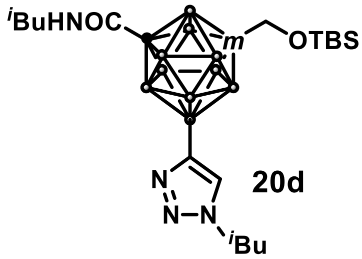

20d

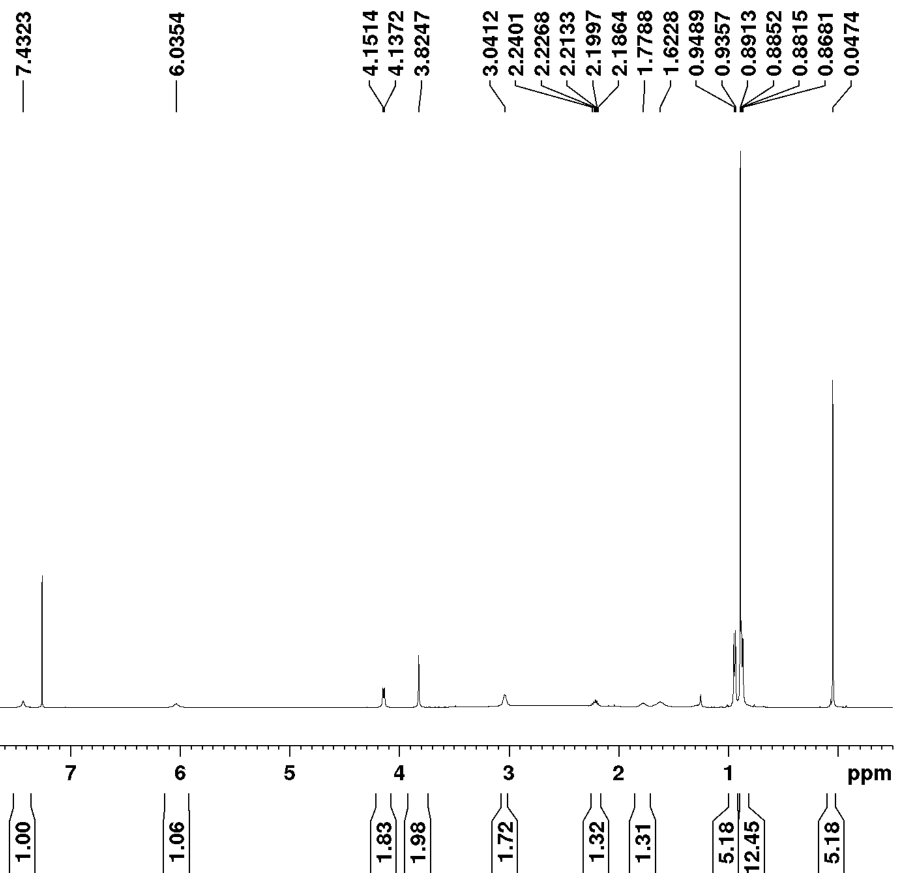

^{13}C NMR (125 MHz; CDCl$_3$)

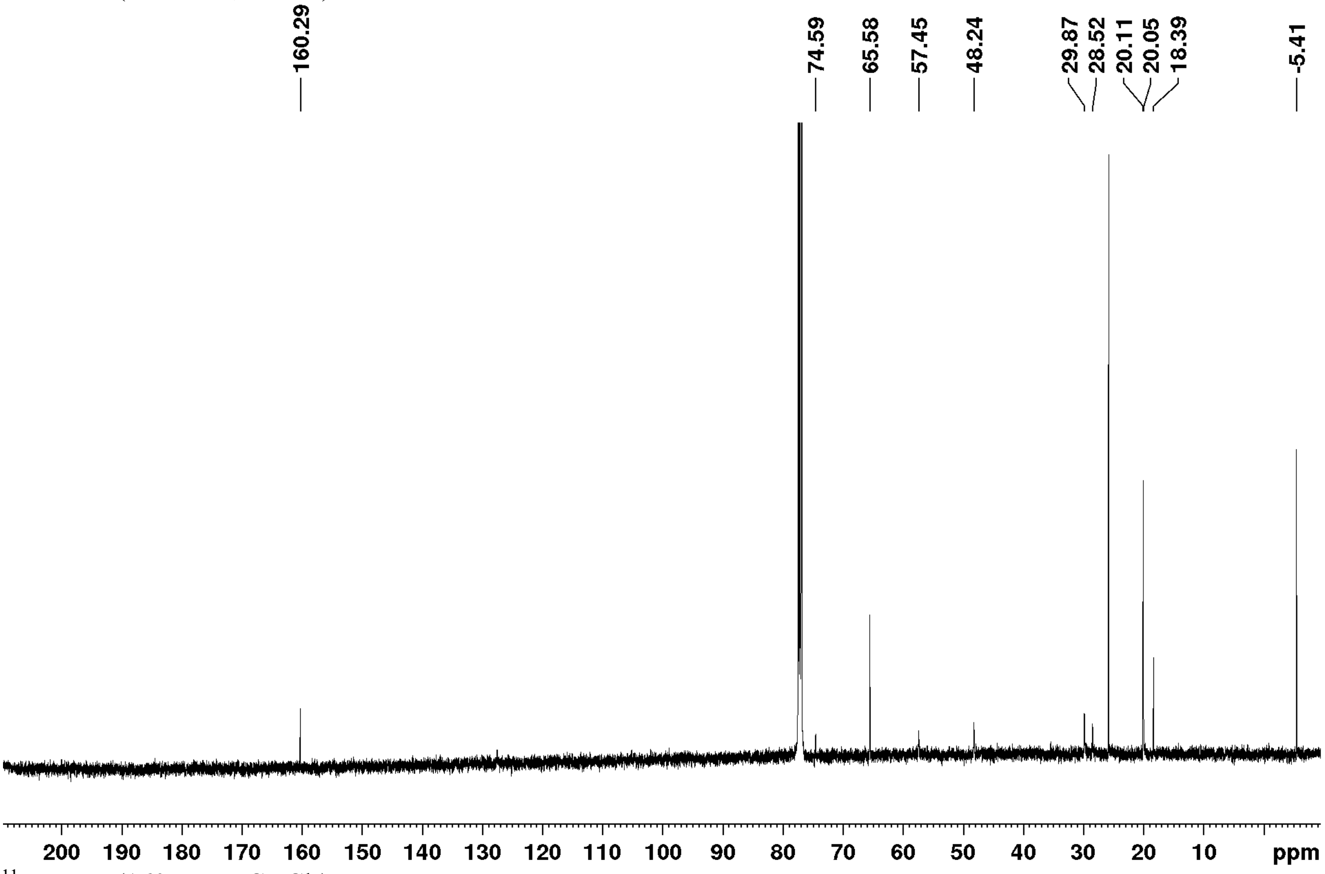

^{11}B NMR (160 MHz; CDCl$_3$)

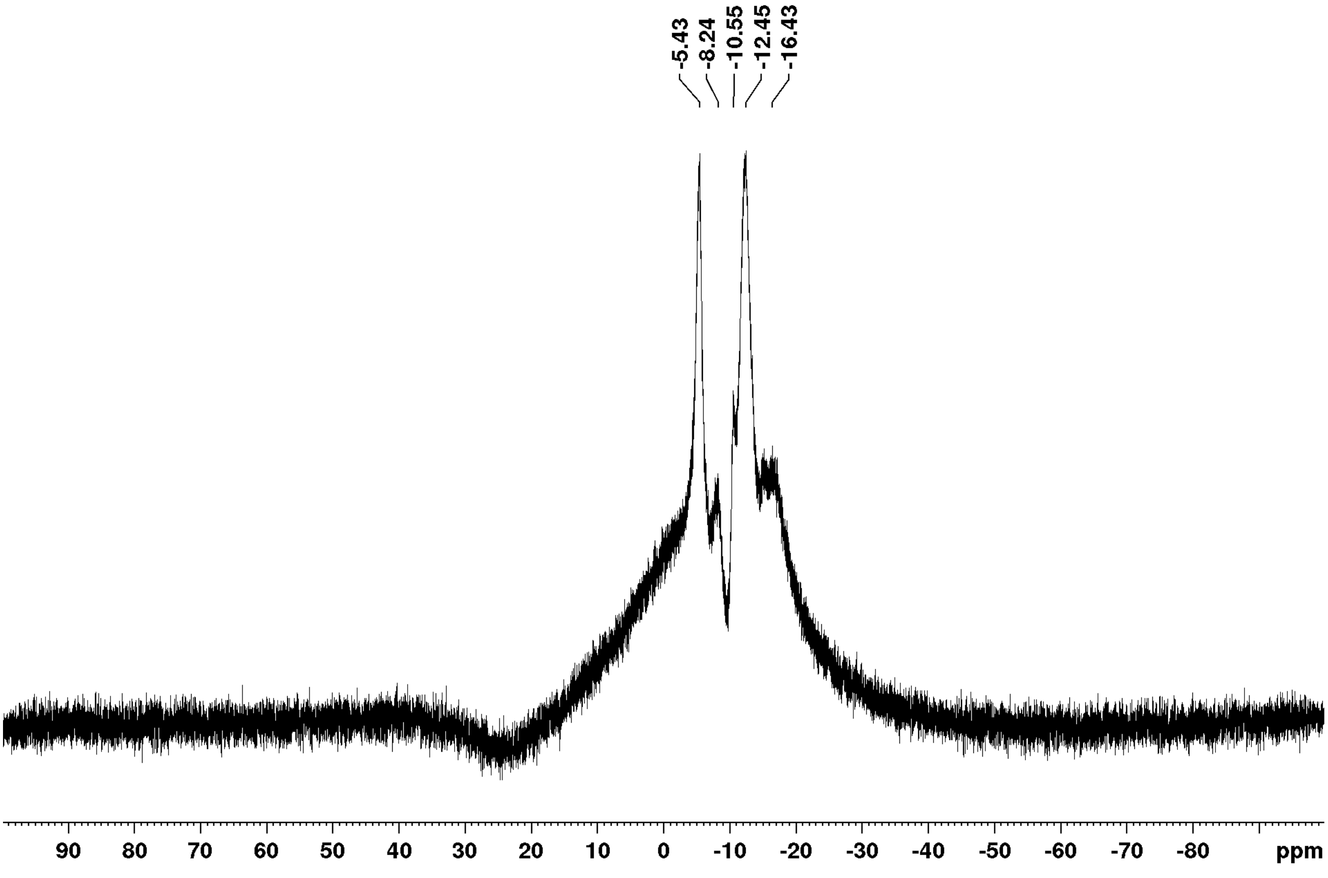

1-phenylacetoxymethyl-7-benzylaminocarboxyl-9-(1*H*-1,2,3-triazol-4-yl)benzylcarbamoyl-1,7-dicarba-*closo*-dodecaborane (IIIa)

^{1}H NMR (500 MHz; $CDCl_3$)

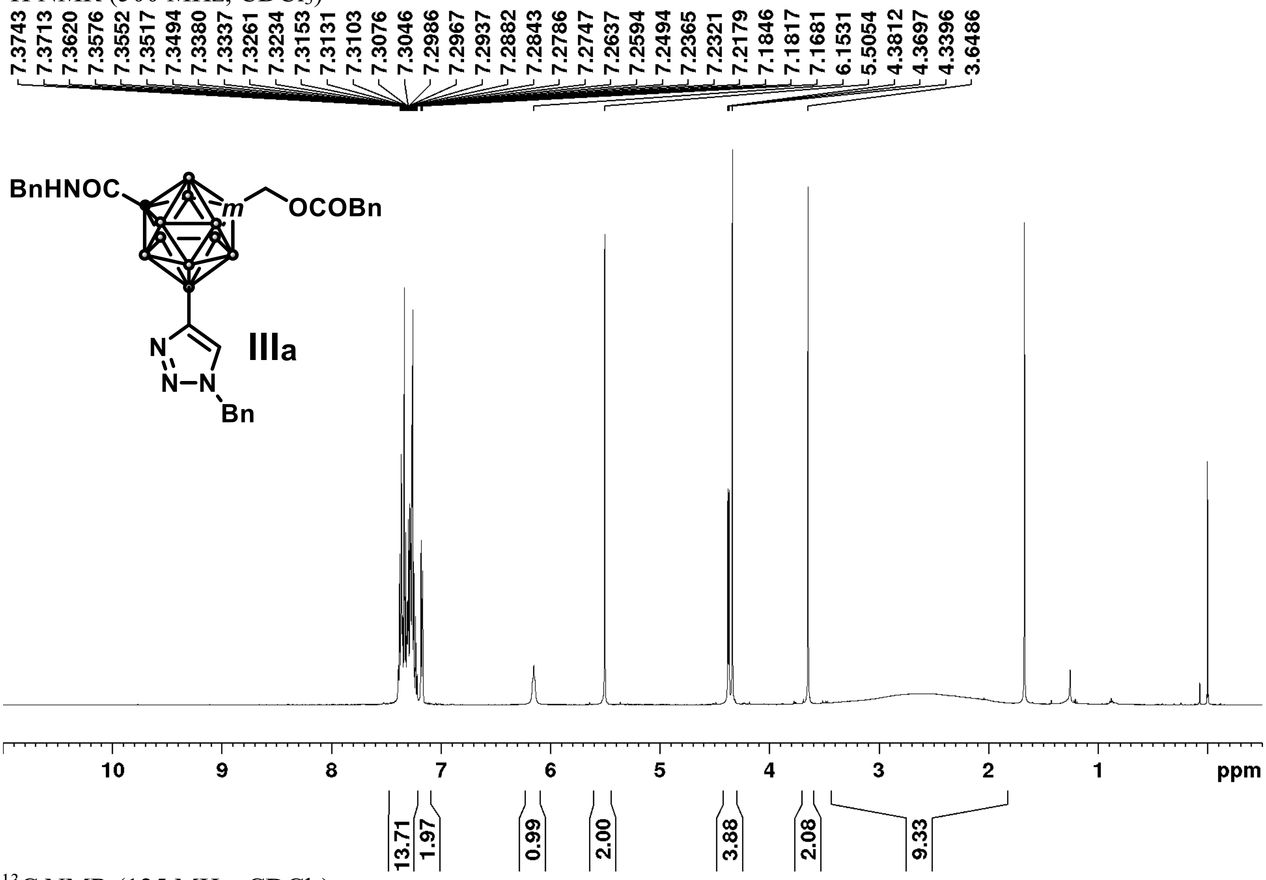

^{13}C NMR (125 MHz; $CDCl_3$)

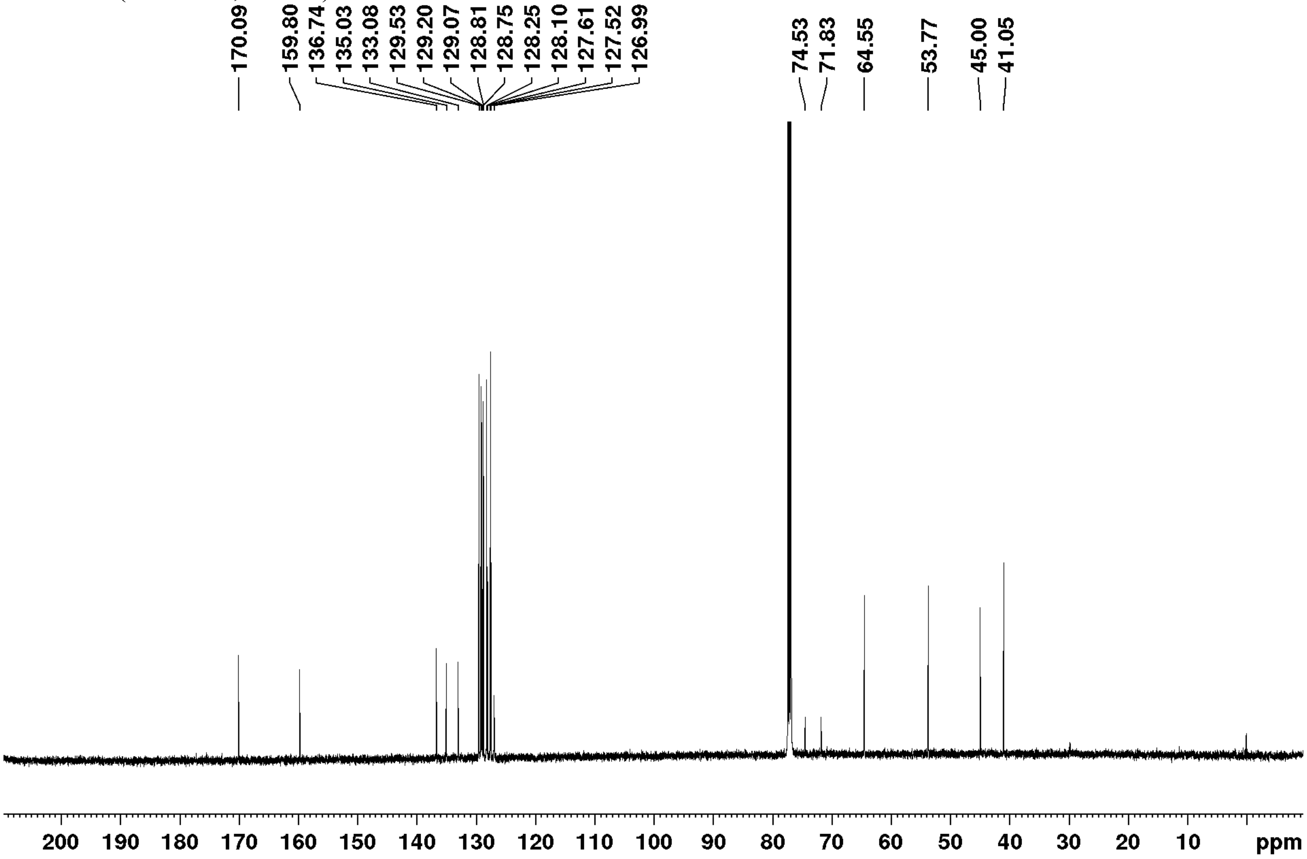

^{11}B NMR (160 MHz; $CDCl_3$)

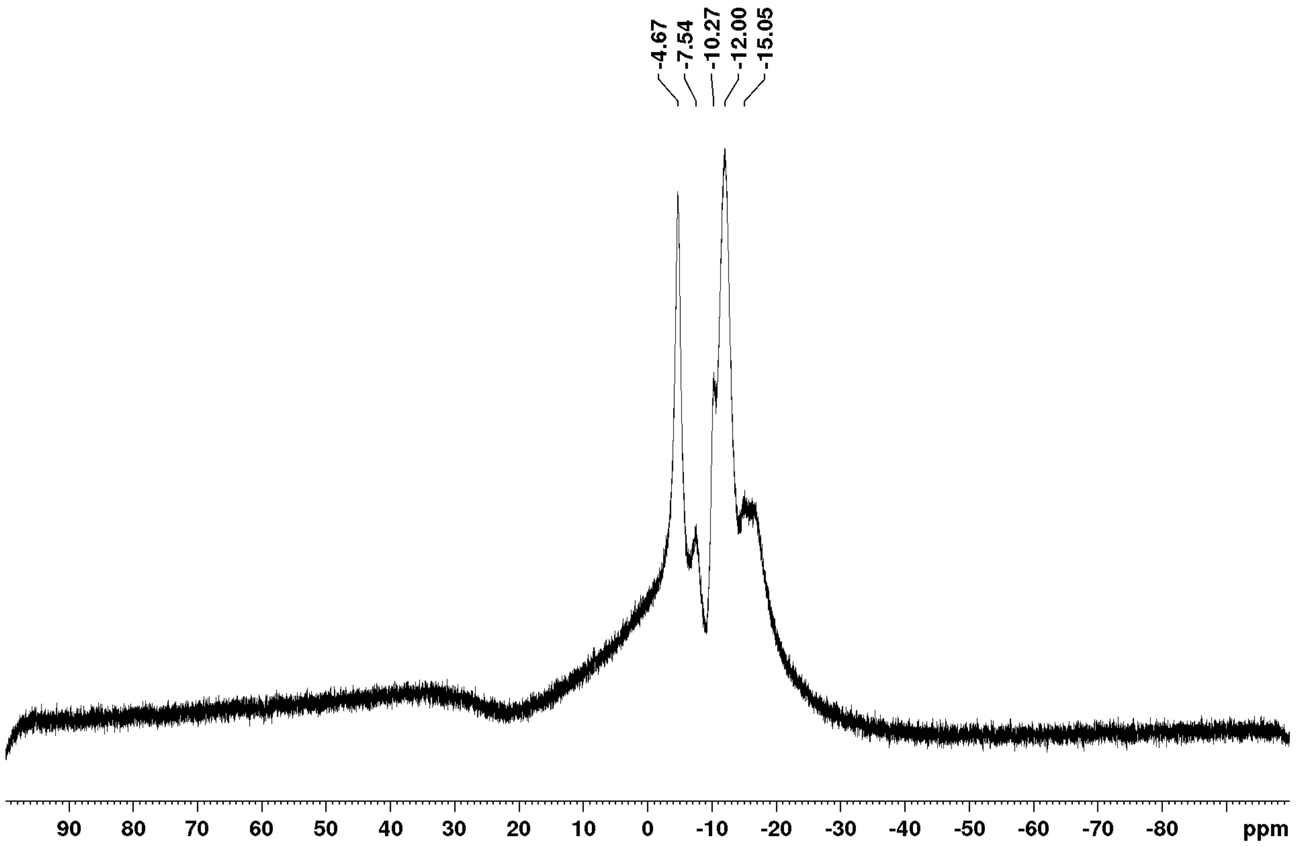

1-(3-methylbutanoyl)oxymethyl-7-benzylaminocarboxyl-9-(1*H*-1,2,3-triazol-4-yl)benzylcarbamoyl-1,7-dicarba-*closo*-dodecaborane (IIIb)

^1H NMR (500 MHz; $CDCl_3$)

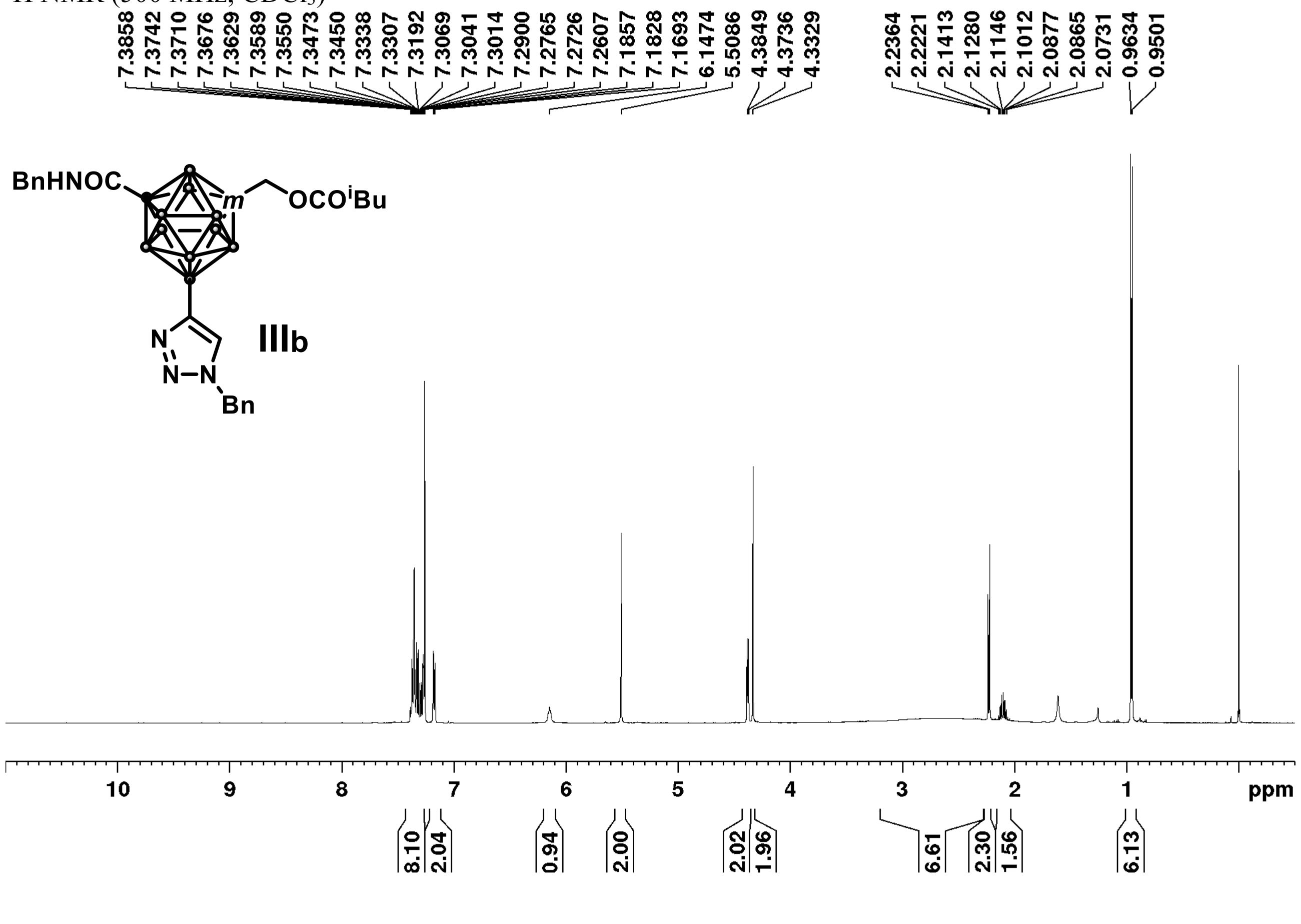

^{13}C NMR (125 MHz; $CDCl_3$)

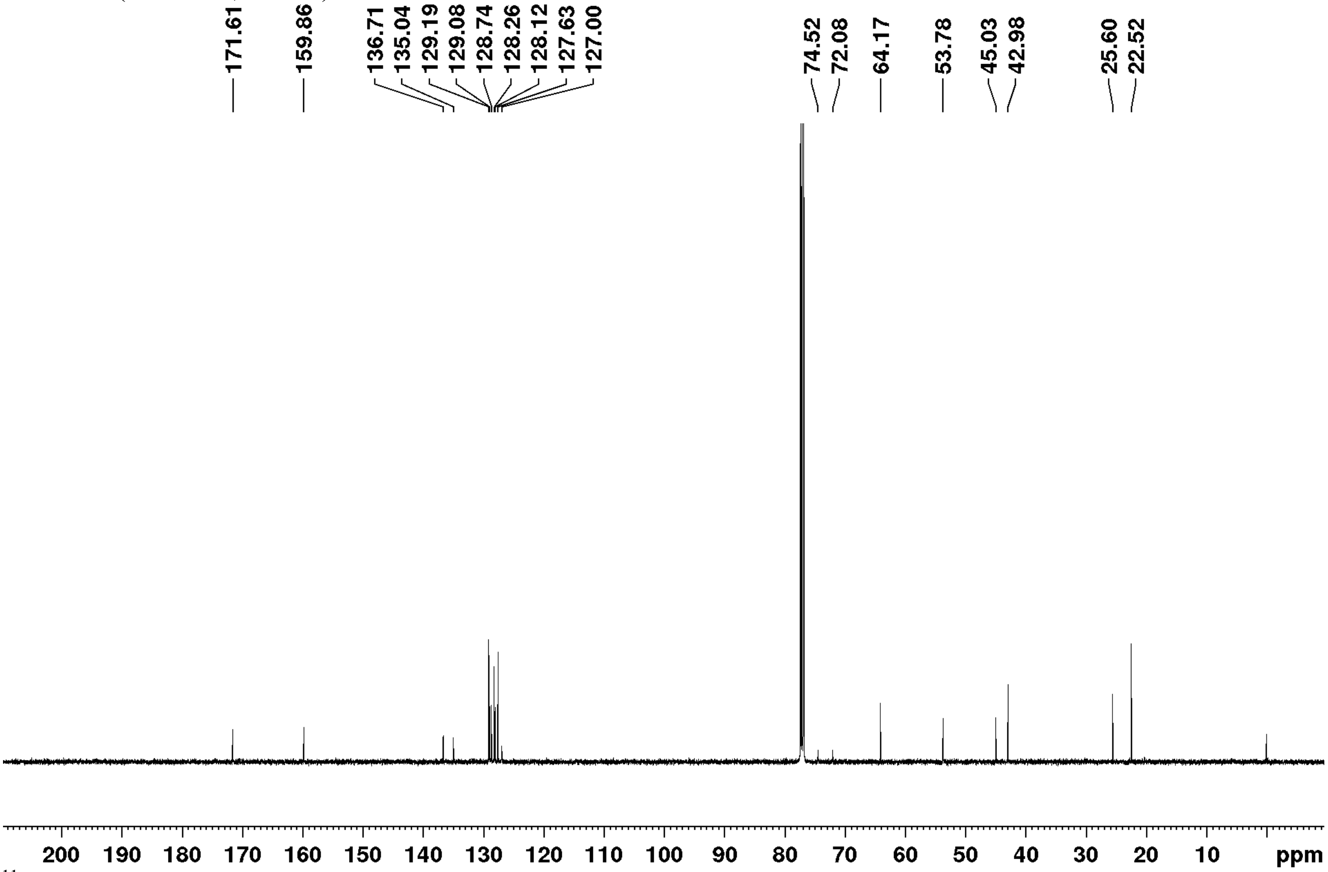

^{11}B NMR (160 MHz; $CDCl_3$)

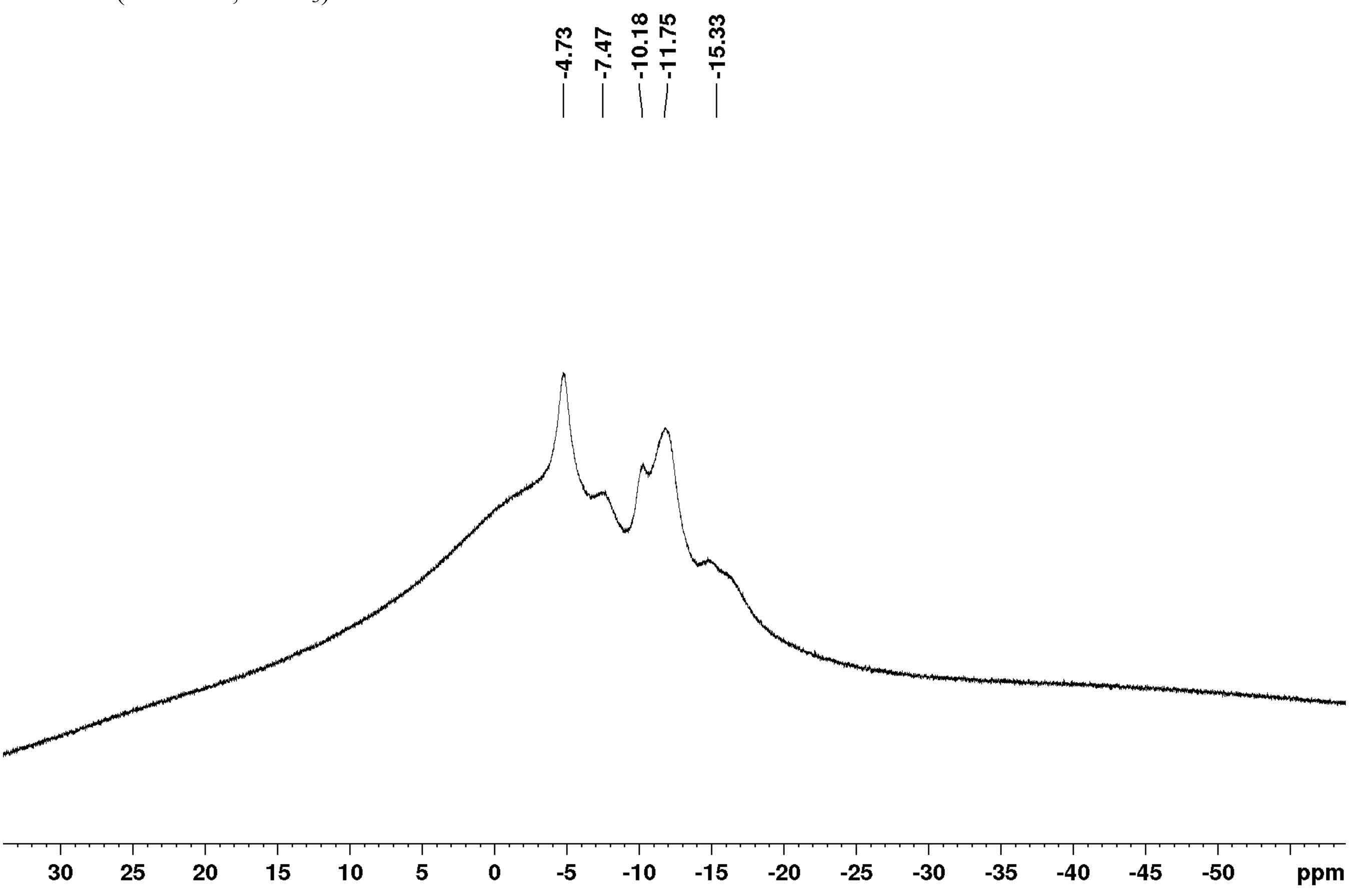

1-phenylacetoxymethyl-7-benzylaminocarboxyl-9-((1*H*-1,2,3-triazol-4-yl)-3-methyl-butanoyl)-1,7-dicarba-*closo*-dodecaborane (IIIc)

^{1}H NMR (500 MHz; $CDCl_3$)

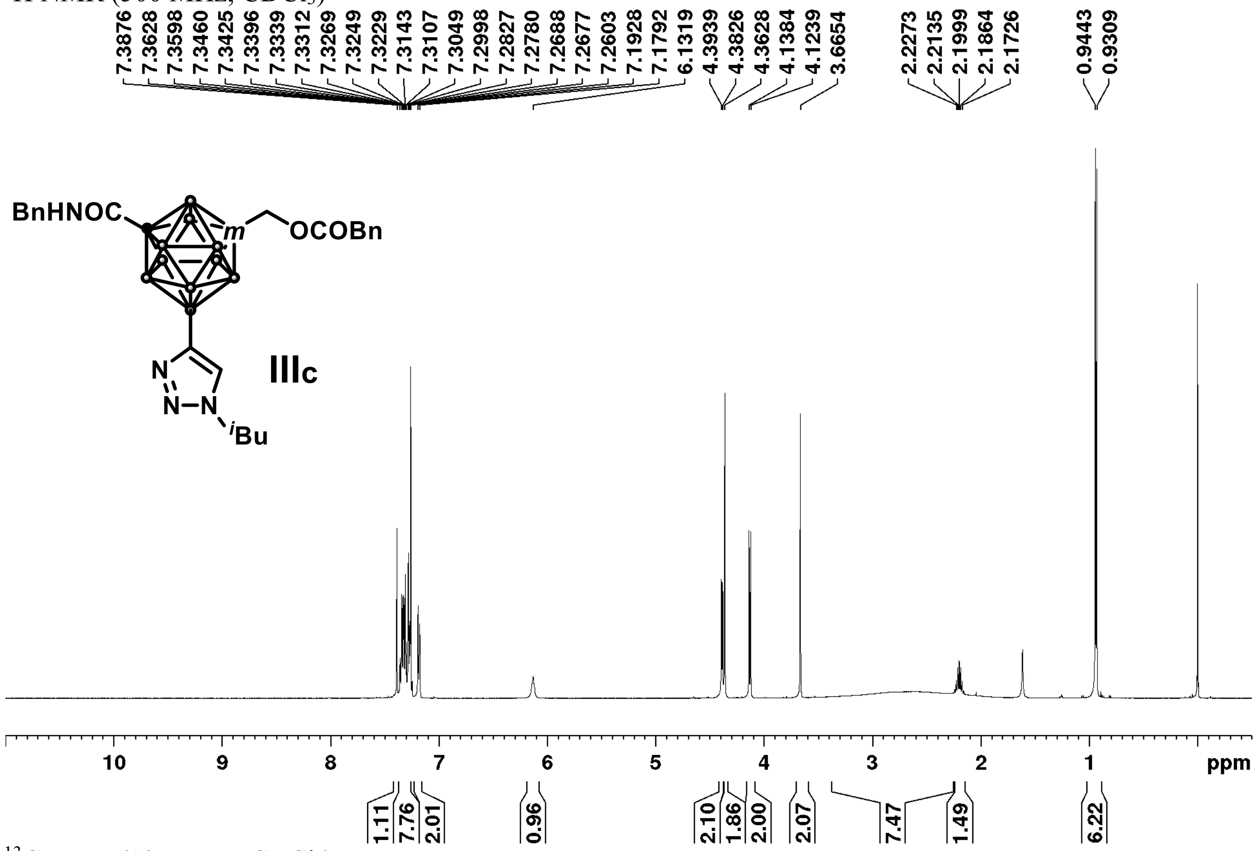

^{13}C NMR (125 MHz; $CDCl_3$)

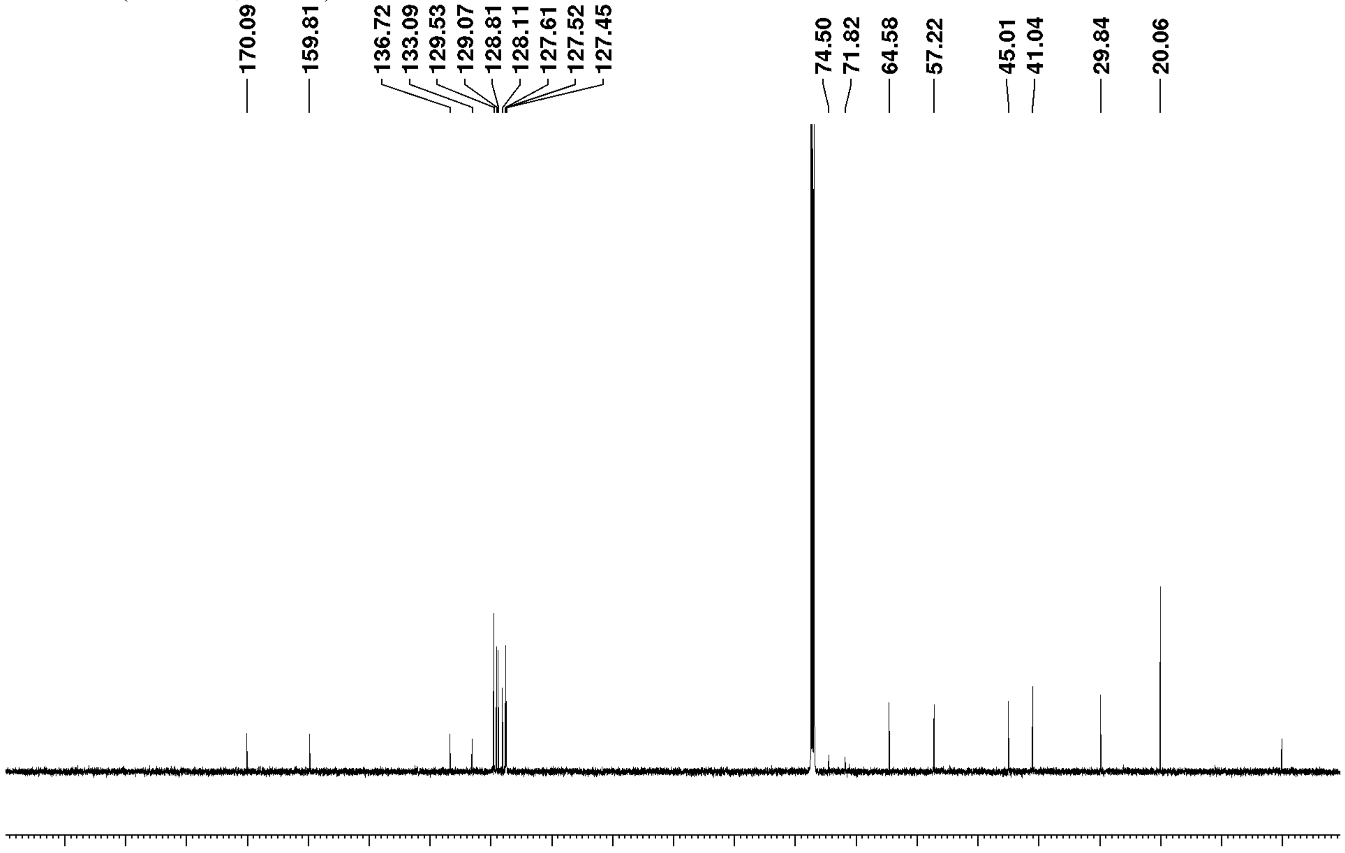

^{11}B NMR (160 MHz; $CDCl_3$)

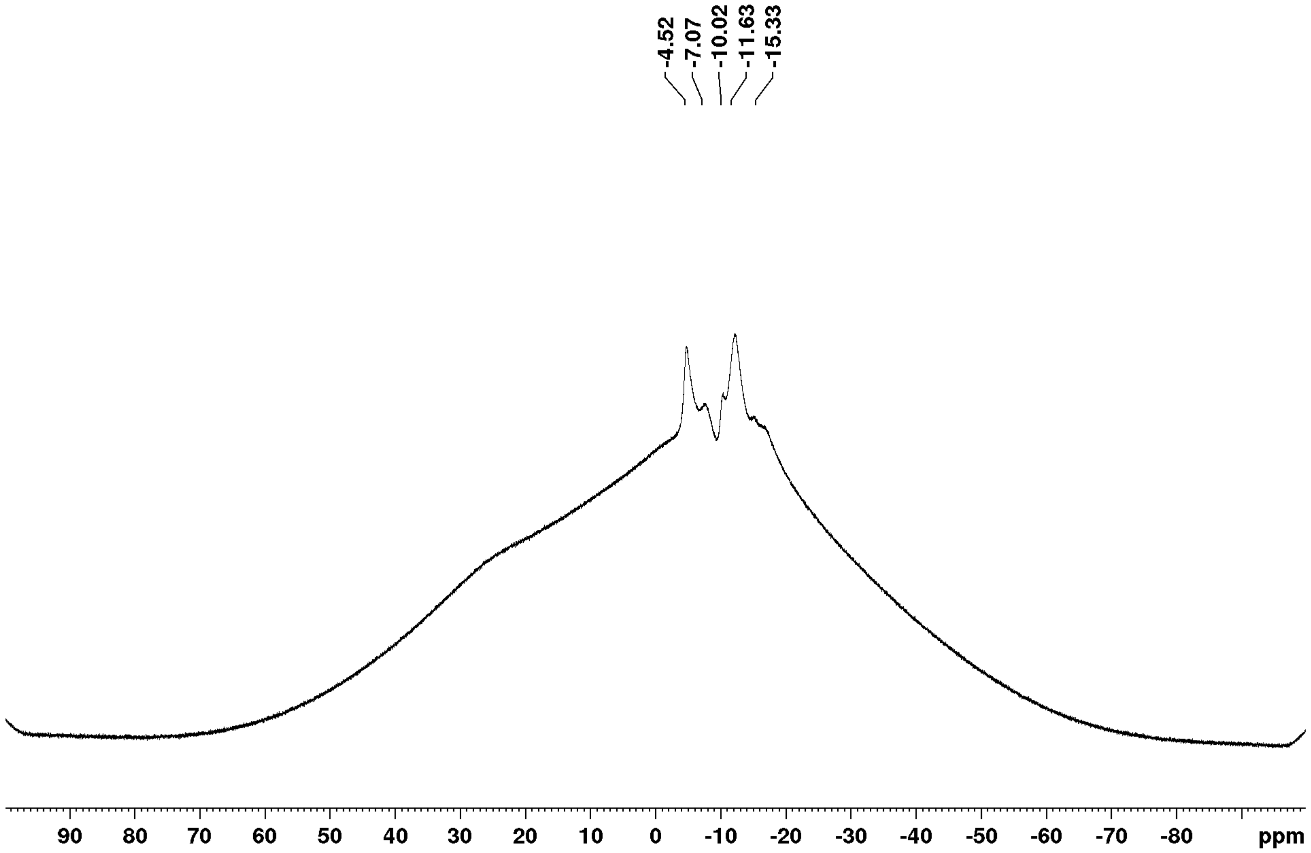

1-(3-methylbutanoyl)oxymethyl-7-benzylaminocarboxyl-9-((1*H*-1,2,3-triazol-4-yl)-3-methyl-butanoyl)-1,7-dicarba-*closo*-dodecaborane (IIId)

^{1}H NMR (500 MHz; $CDCl_3$)

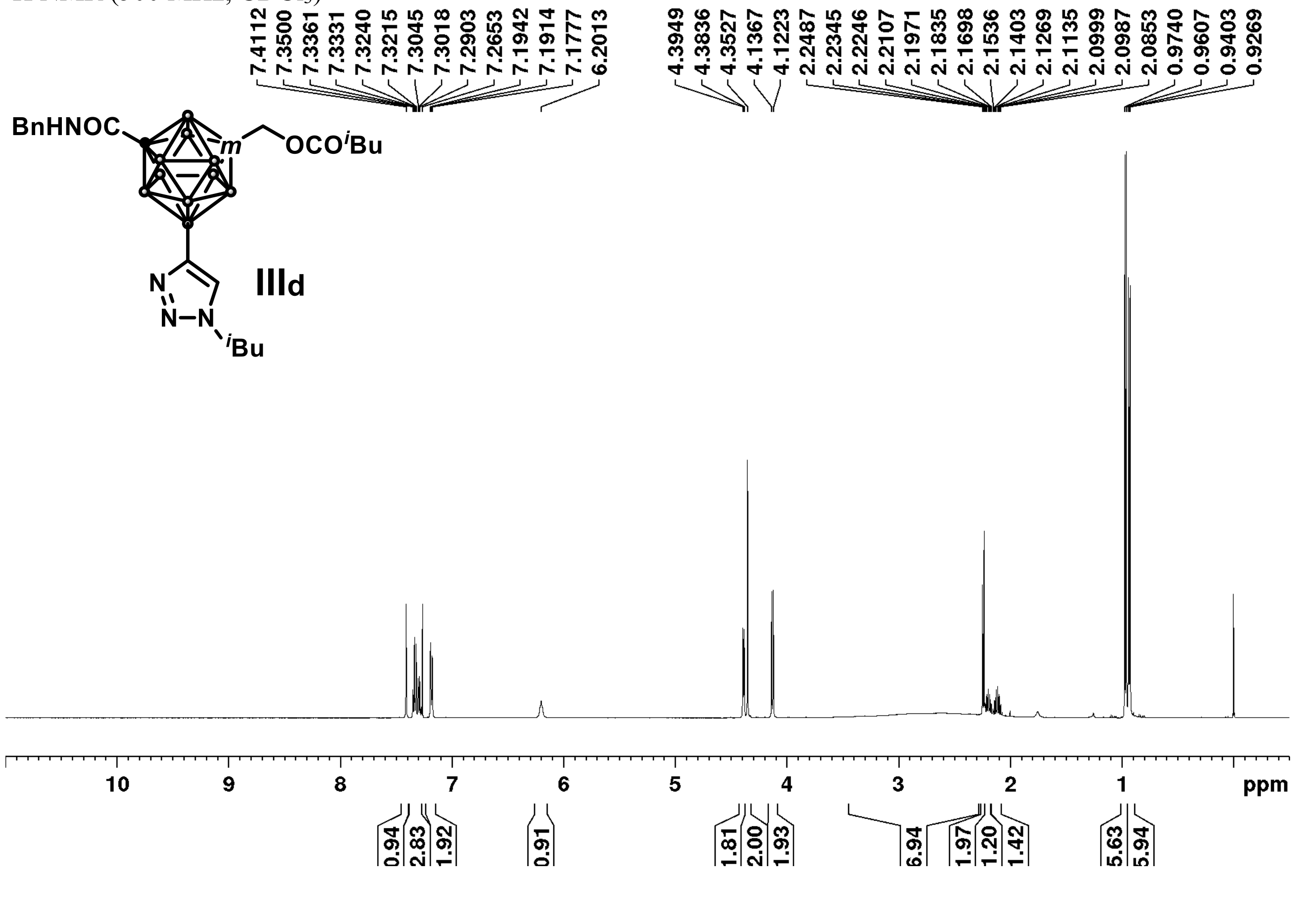

^{13}C NMR (125 MHz; $CDCl_3$)

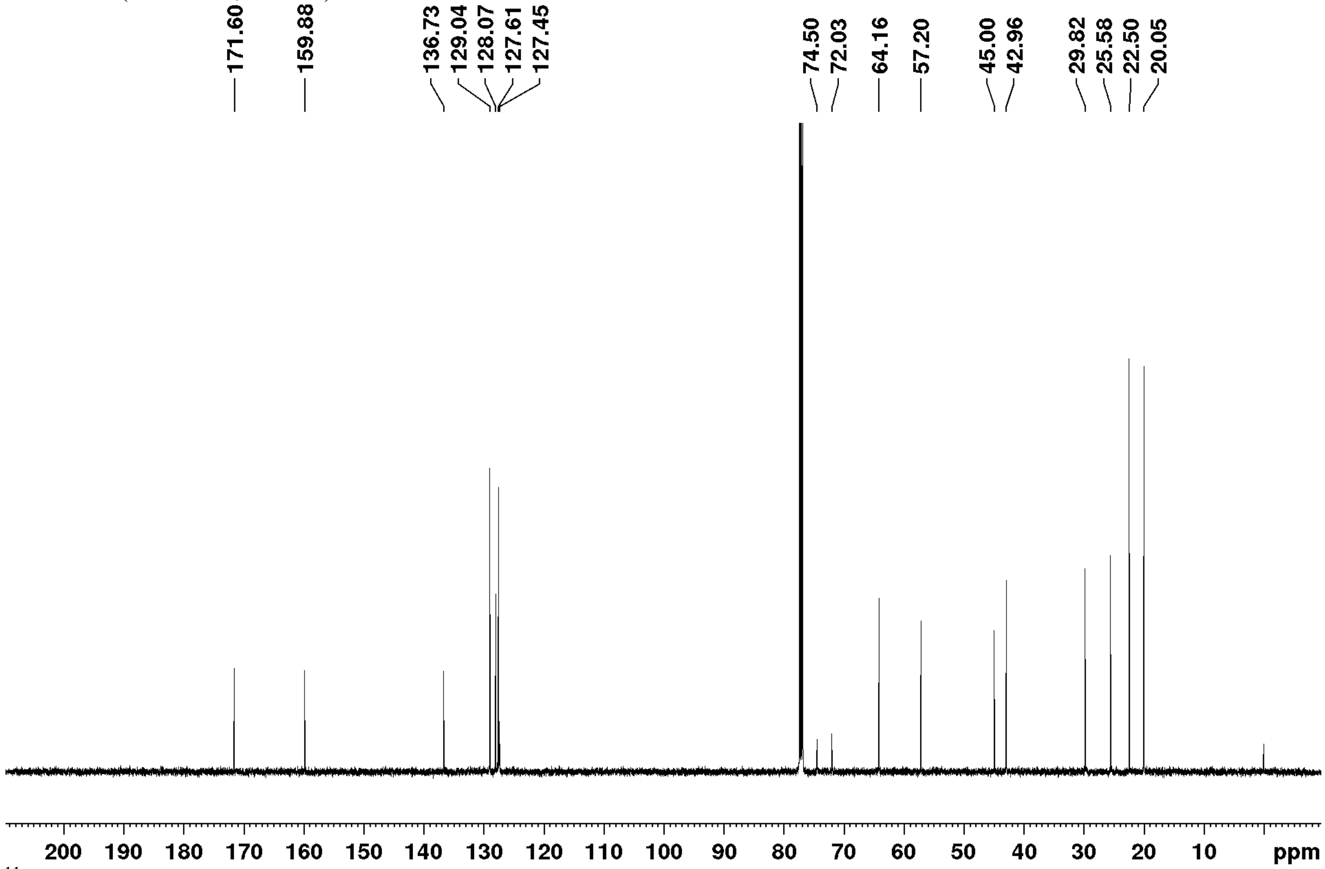

^{11}B NMR (160 MHz; $CDCl_3$)

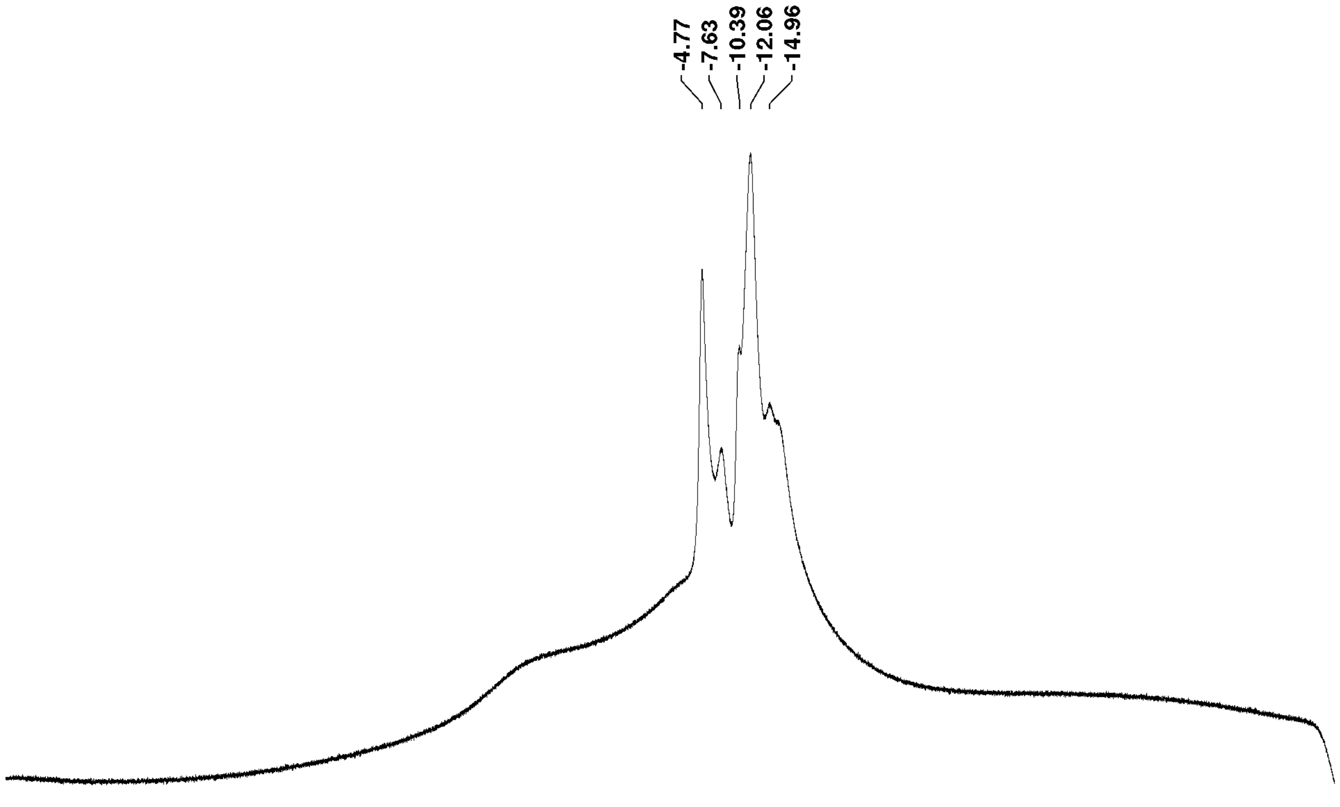

1-phenylacetoxymethyl-7-isobutylcarbamoyl-9-(1*H*-1,2,3-triazol-4-yl)benzylcarbamoyl-1,7-dicarba-*closo*-dodecaborane (IIIe)

^{1}H NMR (500 MHz; $CDCl_3$)

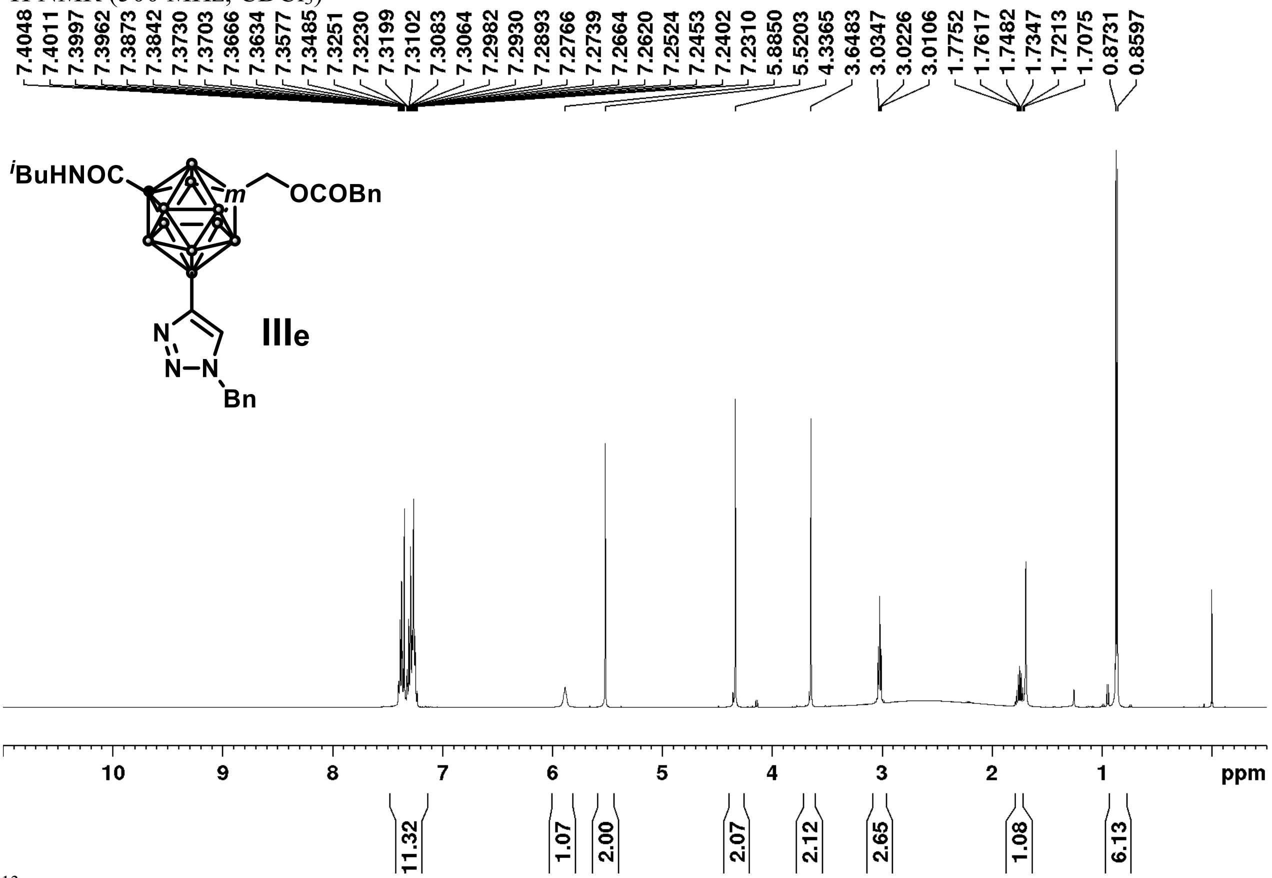

^{13}C NMR (125 MHz; $CDCl_3$)

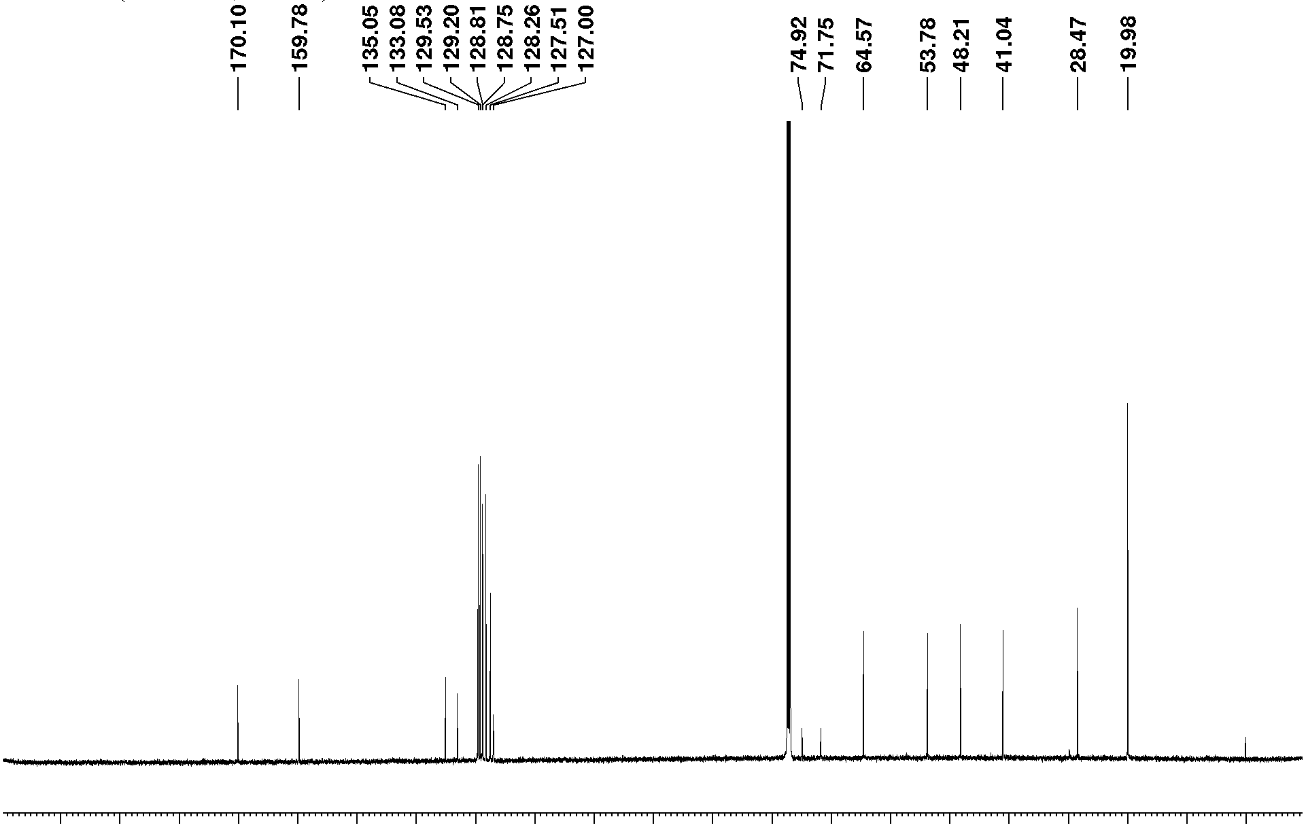

^{11}B NMR (160 MHz; $CDCl_3$)

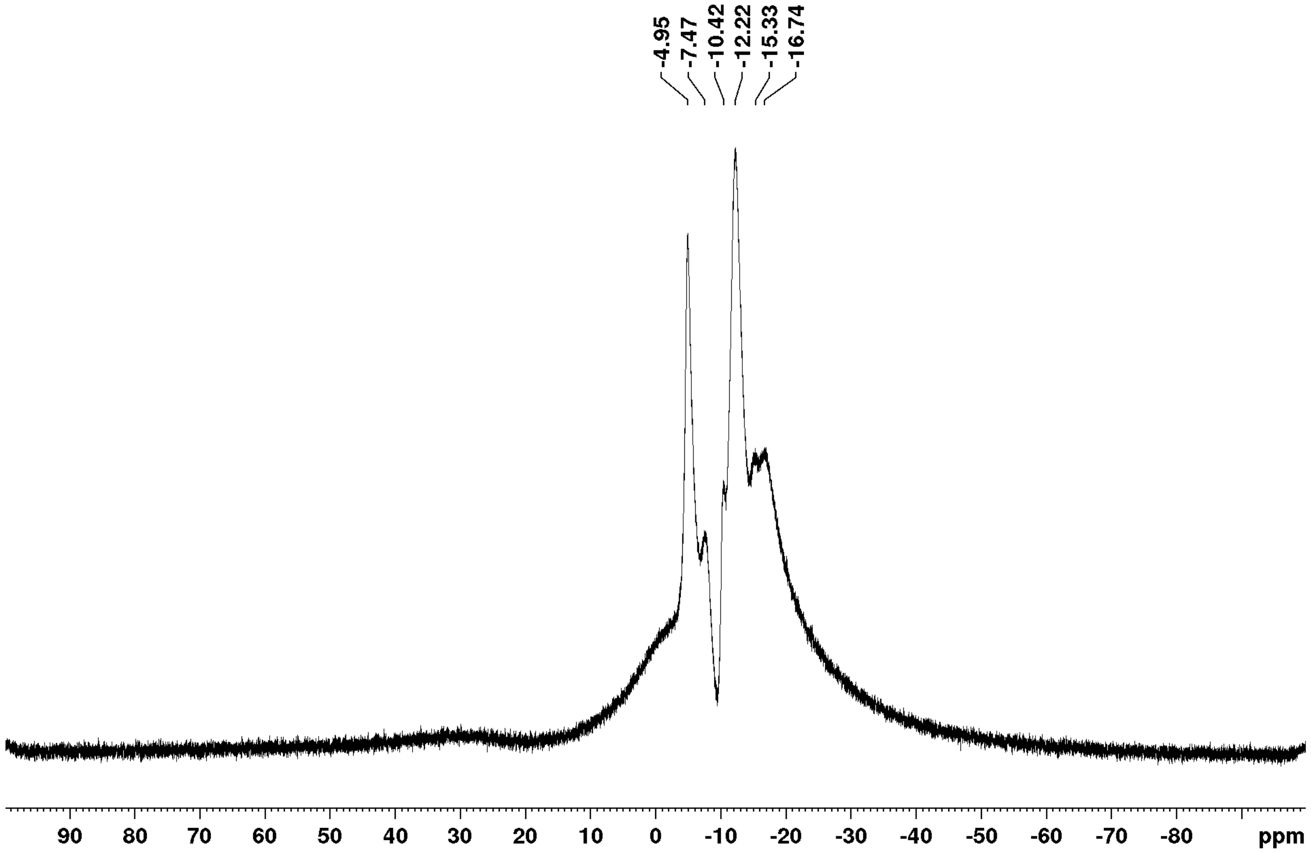

1-(3-methylbutanoyl)oxymethyl-7-isobutylcarbamoyl-9-(1*H*-1,2,3-triazol-4-yl)benzylcarbamoyl-1,7-dicarba-*closo*-dodecaborane (IIIf)

^{1}H NMR (500 MHz; $CDCl_3$)

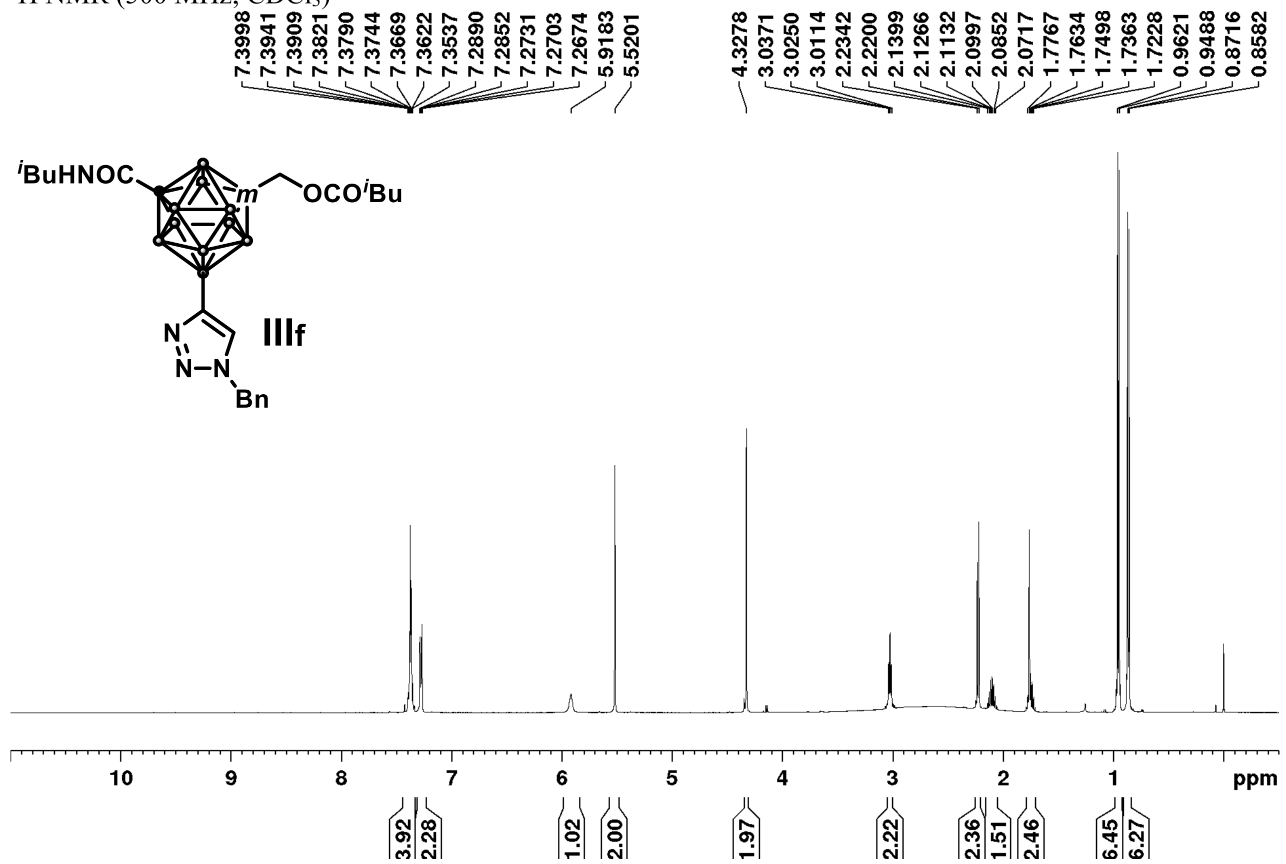

^{13}C NMR (125 MHz; $CDCl_3$)

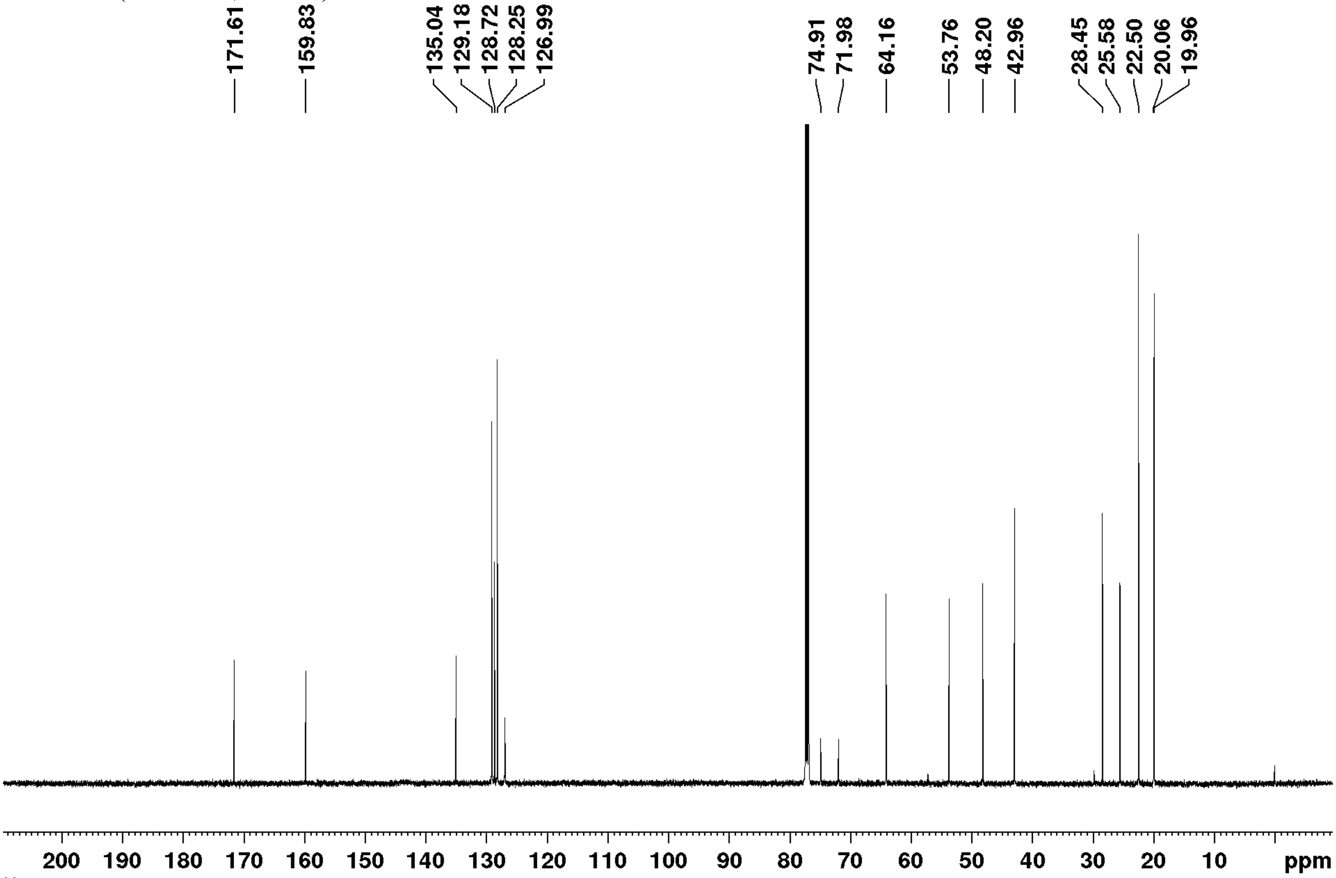

^{11}B NMR (160 MHz; $CDCl_3$)

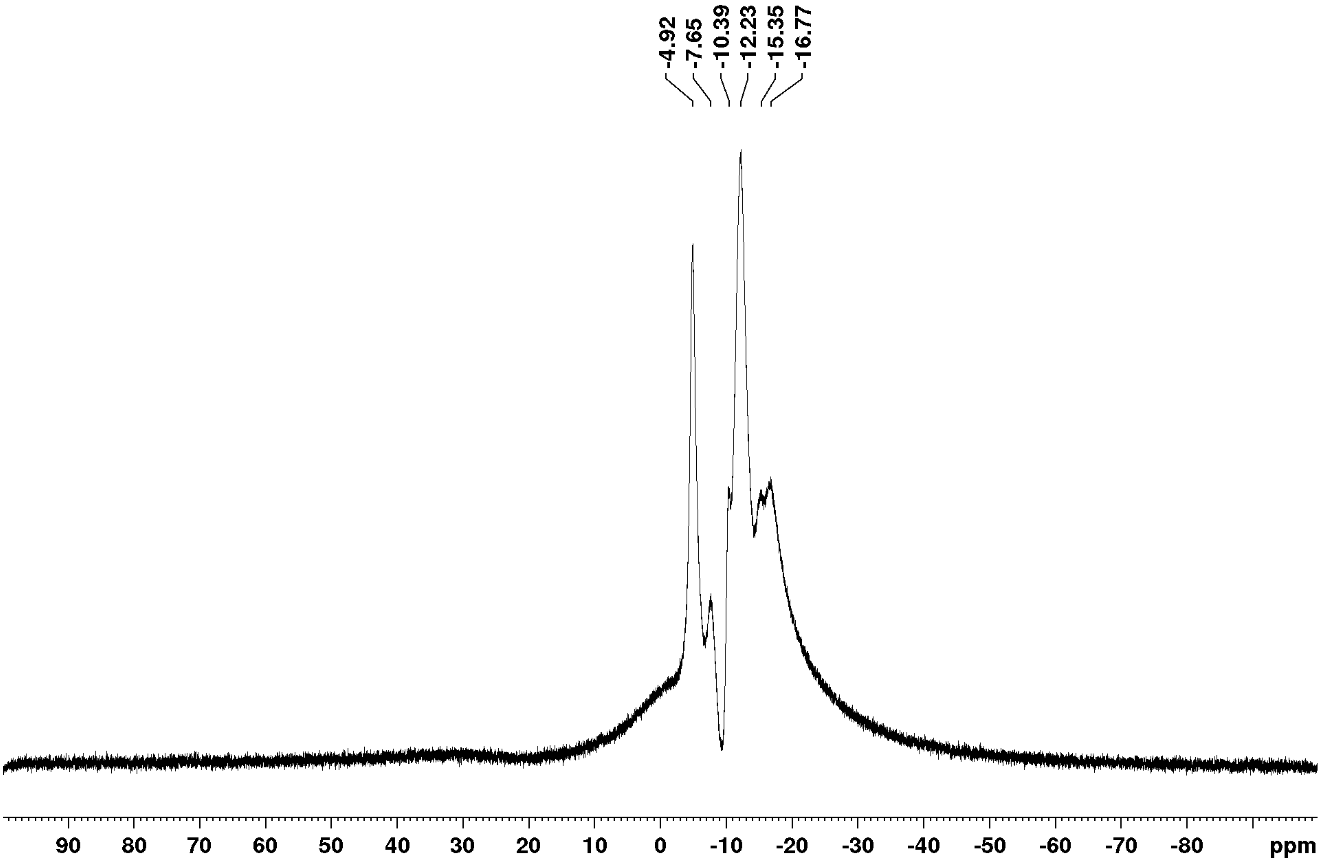

1-phenylacetoxymethyl-7-benzylaminocarboxyl-9-((1*H*-1,2,3-triazol-4-yl)-3-methyl-butanoyl)-1,7-dicarba-*closo*-dodecaborane (IIIg)

^{1}H NMR (500 MHz; $CDCl_3$)

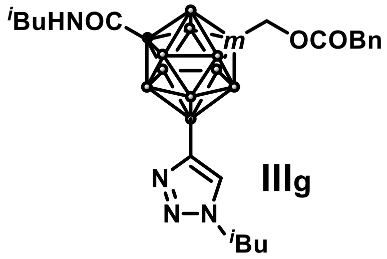

IIIg

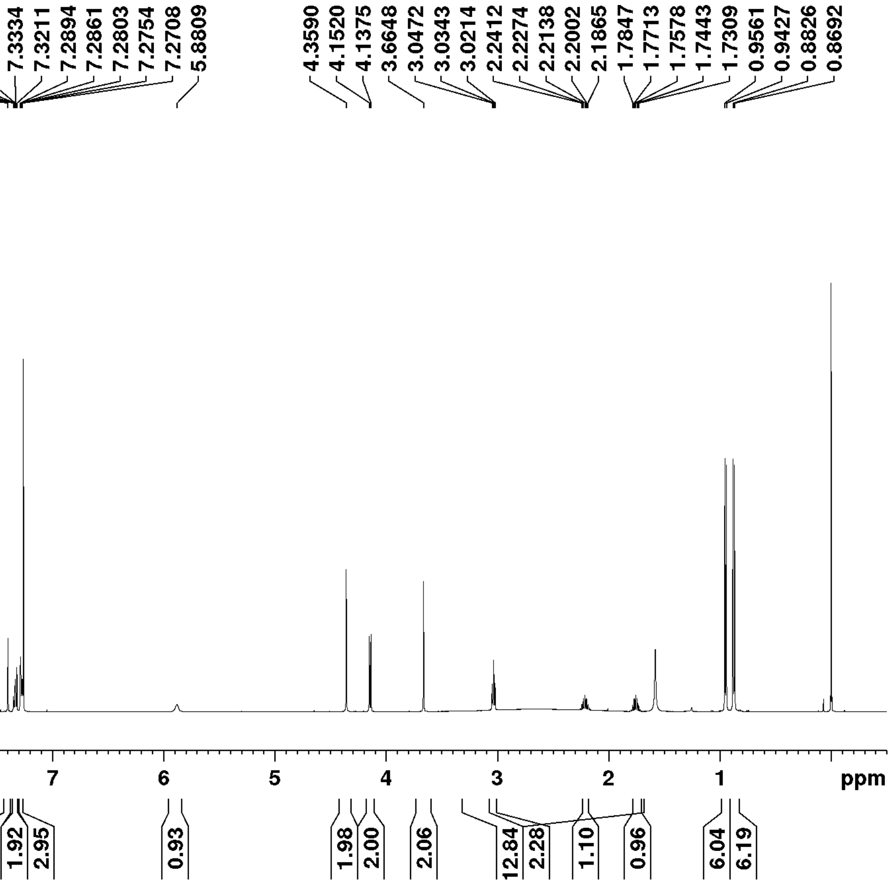

^{13}C NMR (125 MHz; $CDCl_3$)

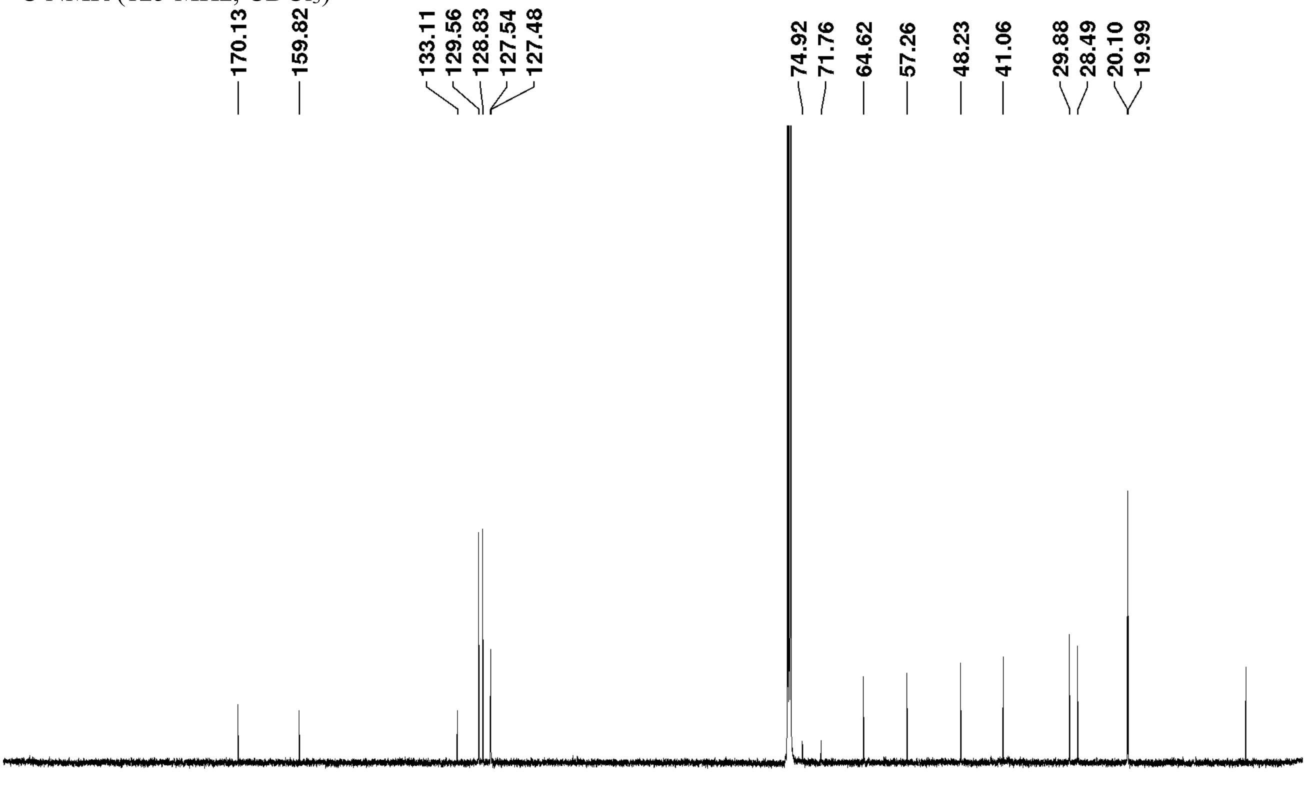

^{11}B NMR (160 MHz; $CDCl_3$)

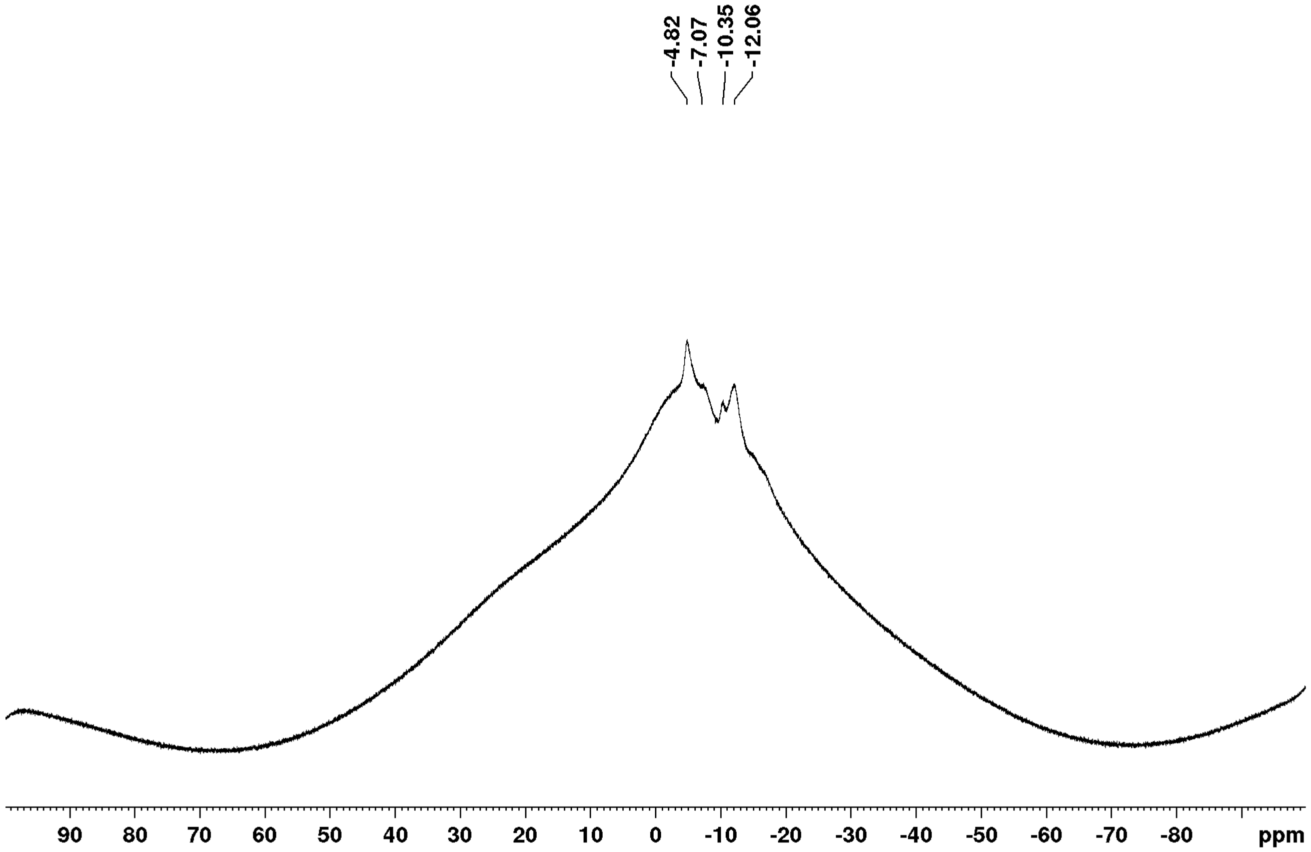

1-(3-methylbutanoyl)oxymethyl-7-isobutylcarbamoyl-9-((1*H*-1,2,3-triazol-4-yl)-3-methyl-butanoyl)-1,7-dicarba-*closo*-dodecaborane (IIIh)

^{1}H NMR (500 MHz; $CDCl_3$)

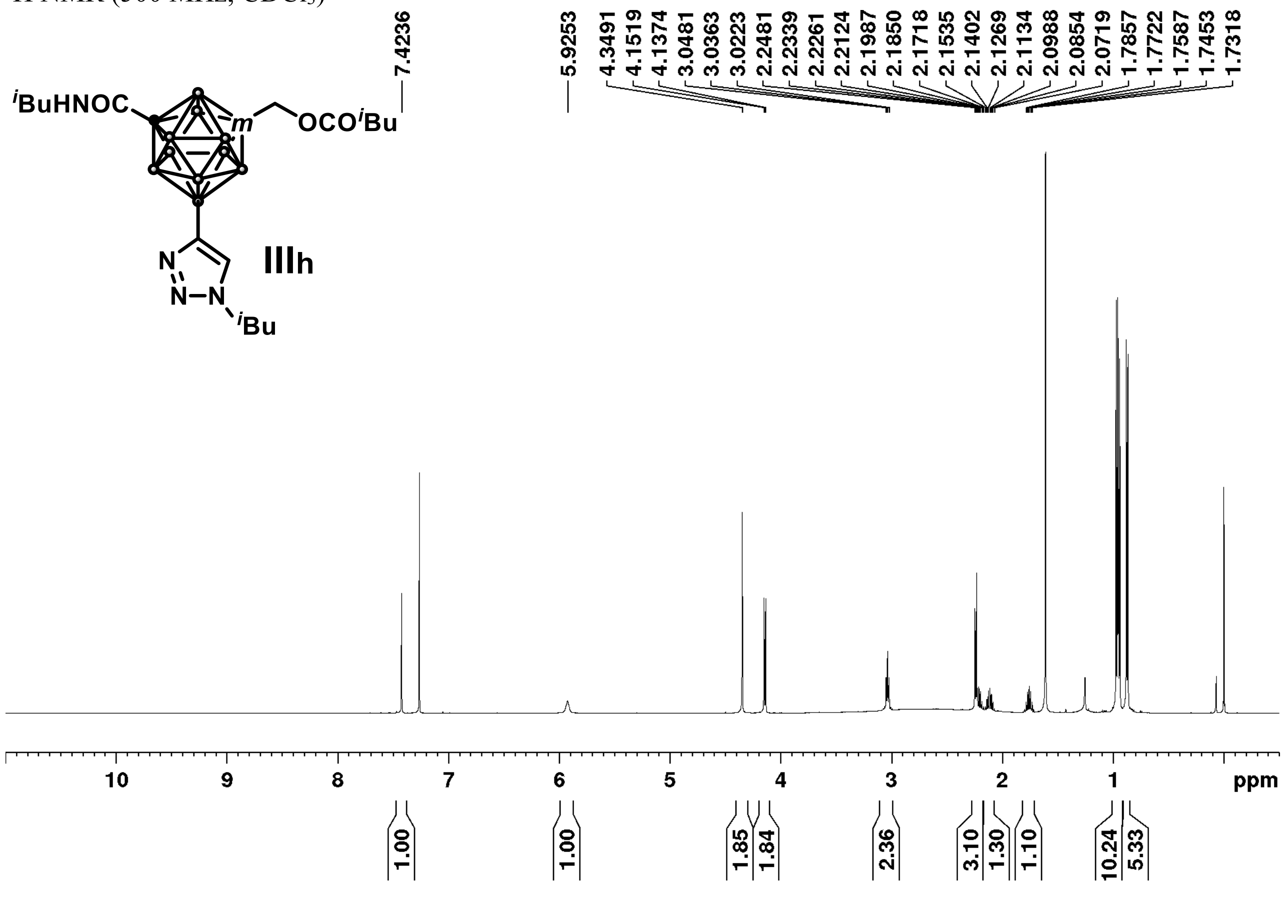

^{13}C NMR (125 MHz; $CDCl_3$)

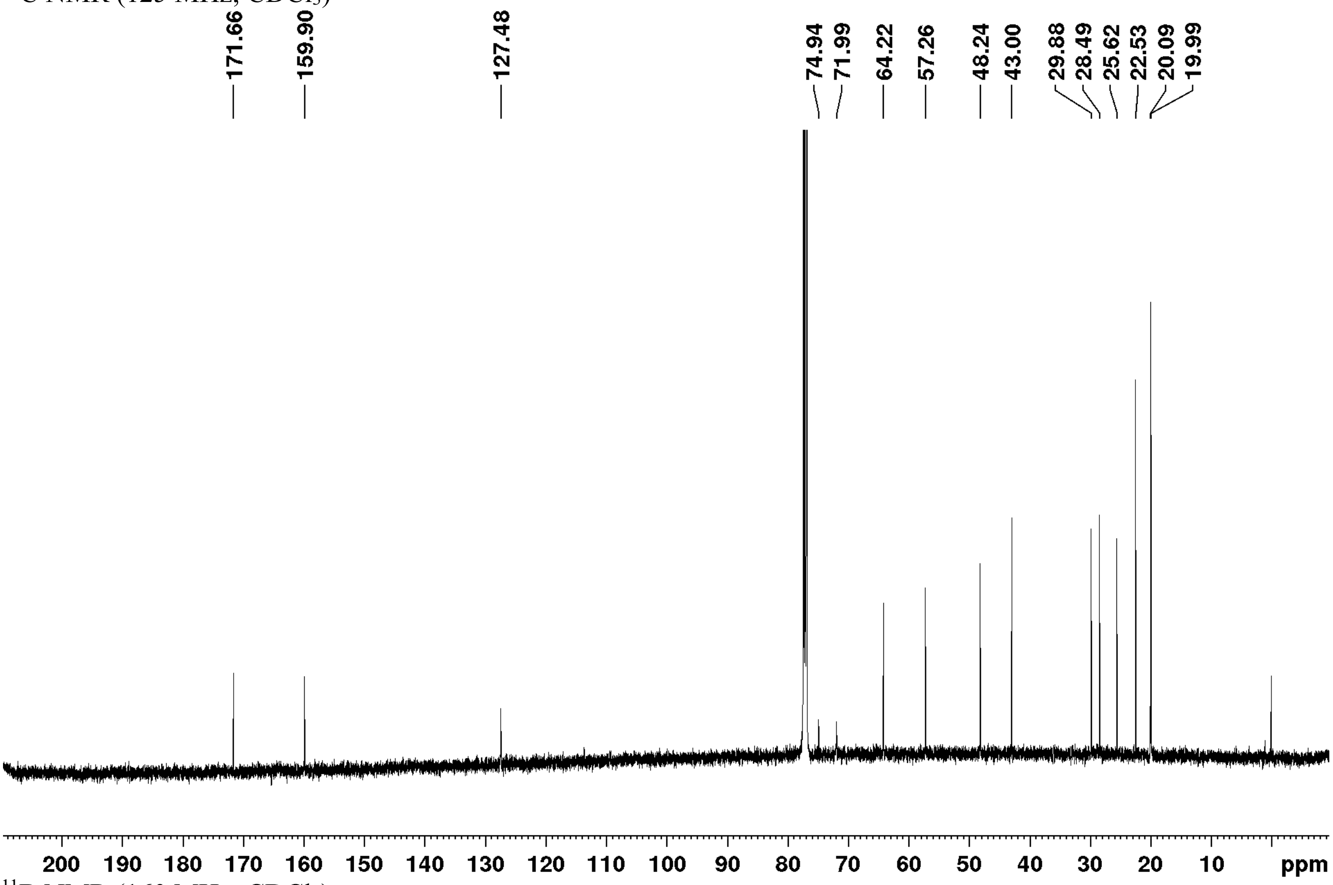

^{11}B NMR (160 MHz; $CDCl_3$)

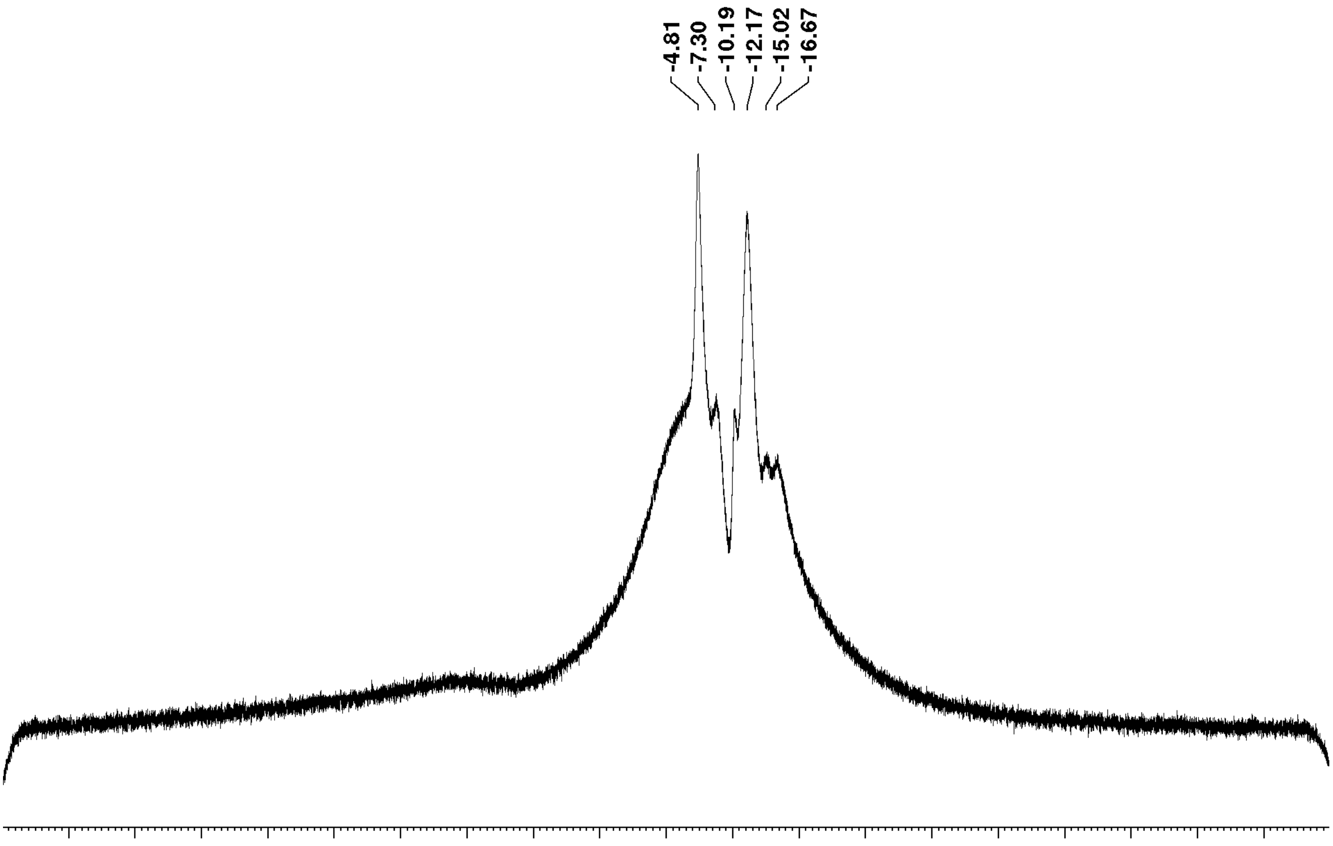

4,9,10-triiodo-1,7-dicarba-*closo*-dodecaborane (22)
^{1}H NMR (400 MHz; $CDCl_3$)

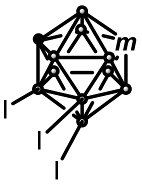

22

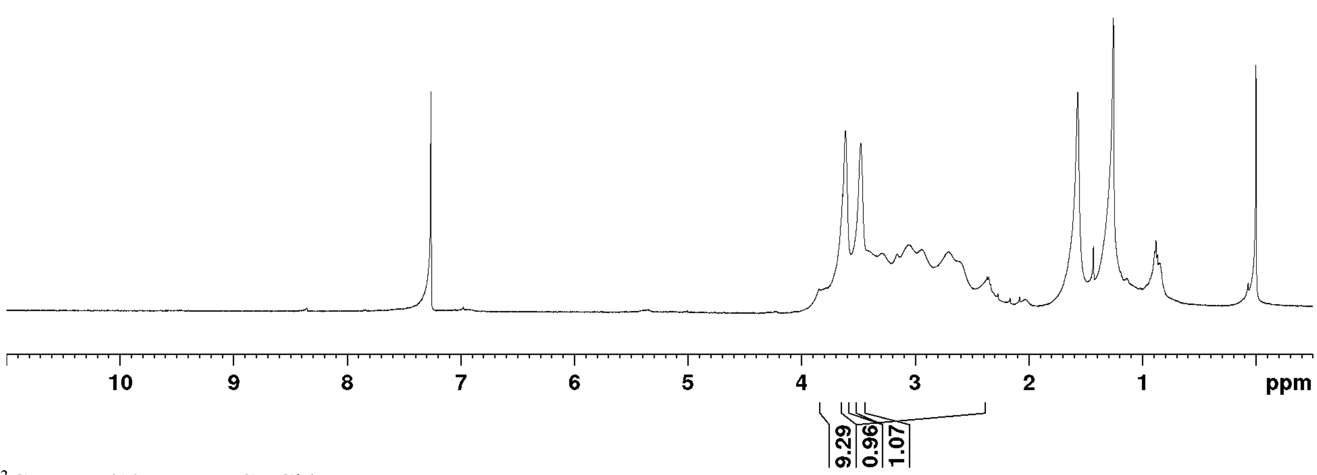

^{13}C NMR (125 MHz; $CDCl_3$)

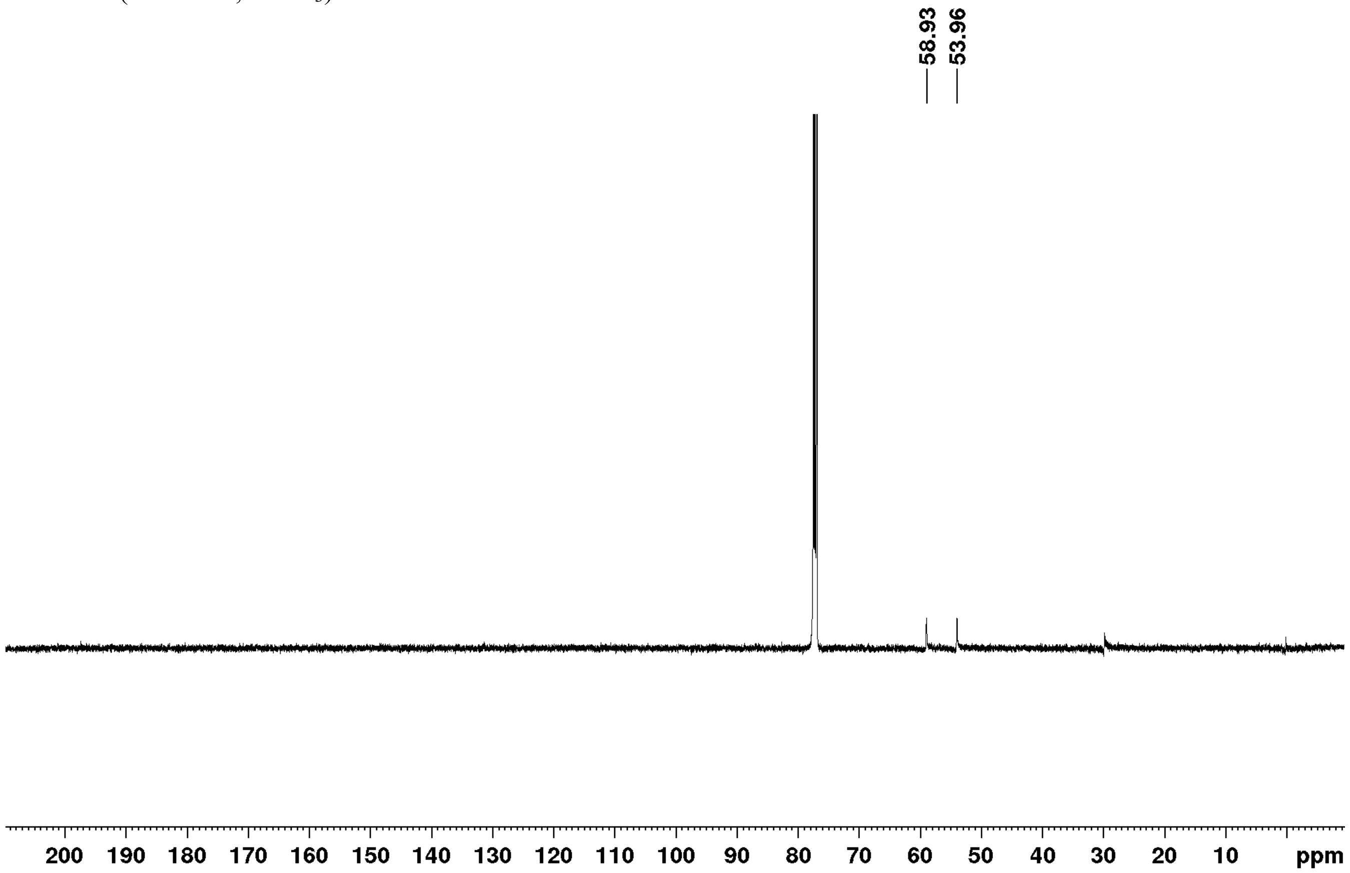

^{11}B NMR (160 MHz; CDCl3)

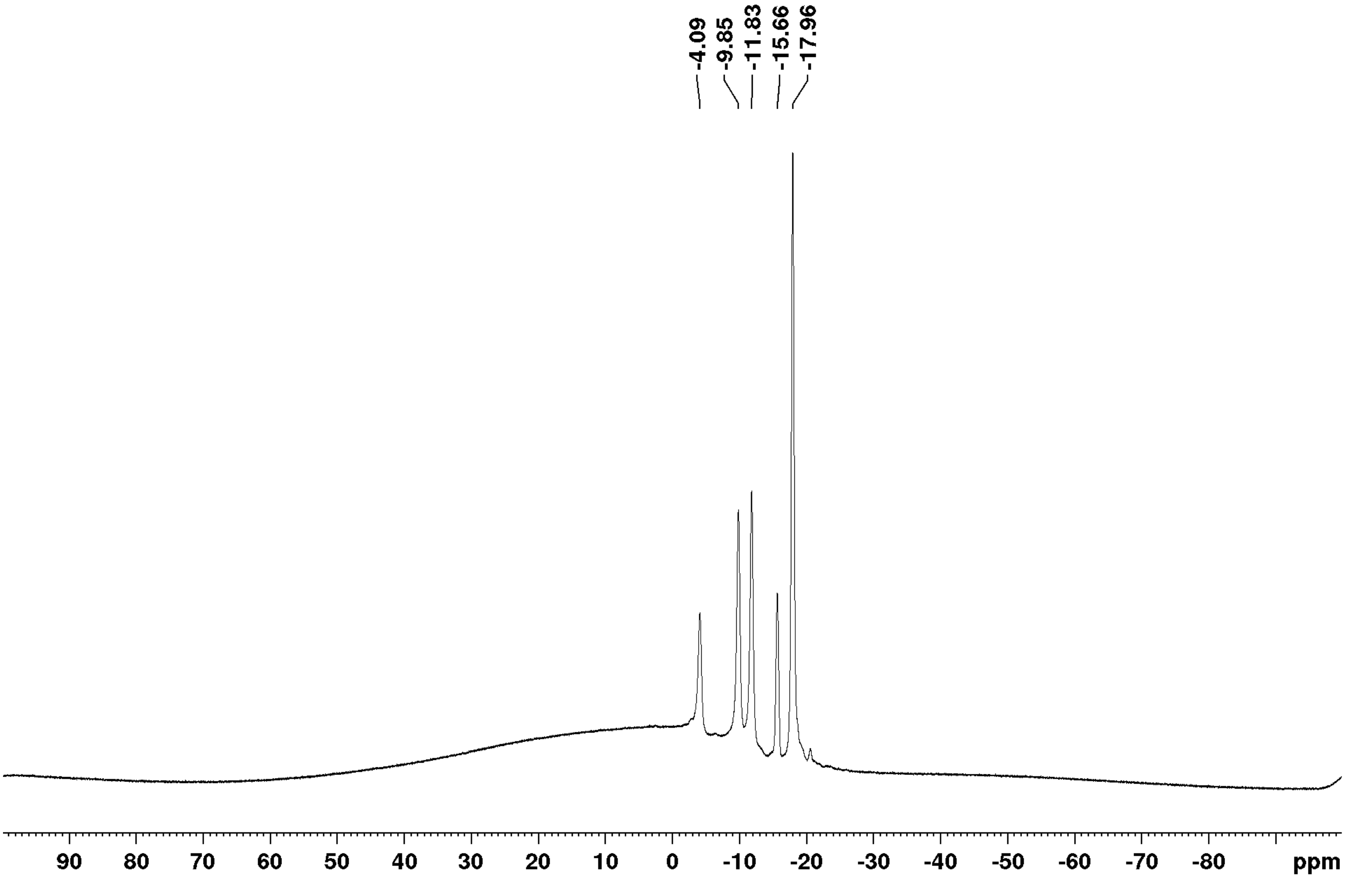

1-benzylaminocarboxyl-9,10-bis((trimethylsilyl)ethynyl)-1,7-dicarba-*closo*-dodecaborane (24a)

^{1}H NMR (400 MHz; CDCl3)

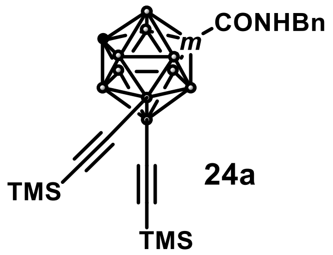

24a

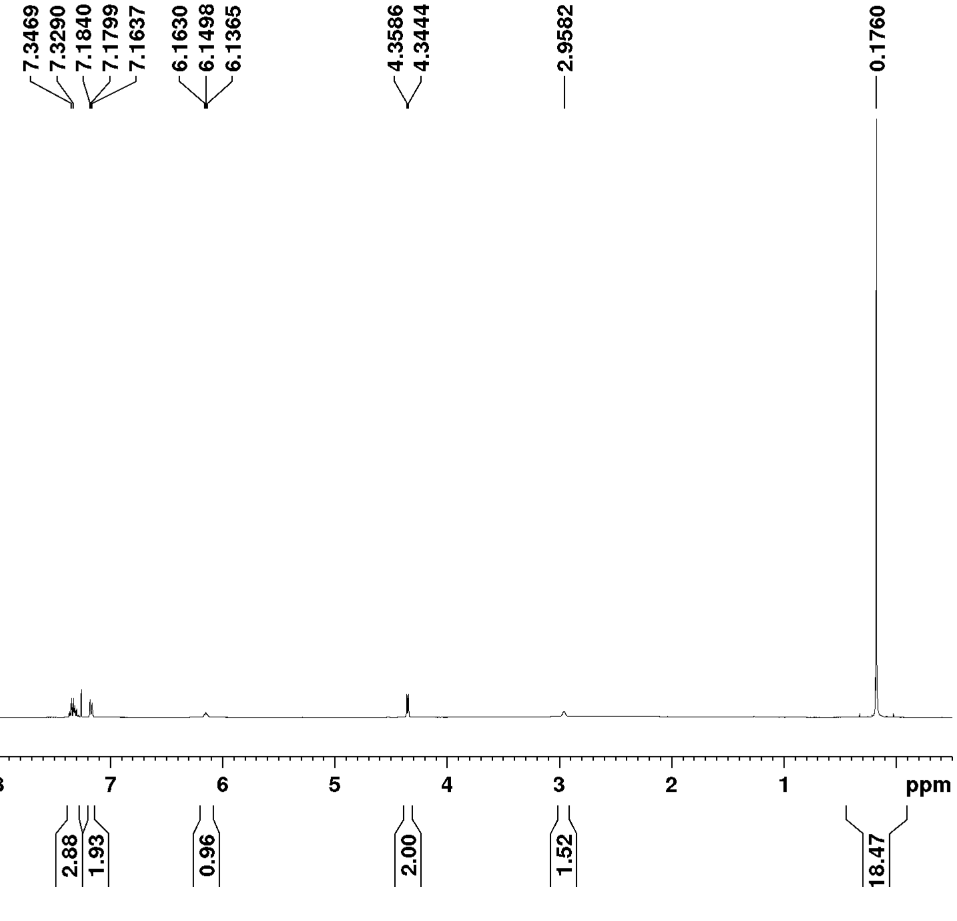

^{13}C NMR (125 MHz; $CDCl_3$)

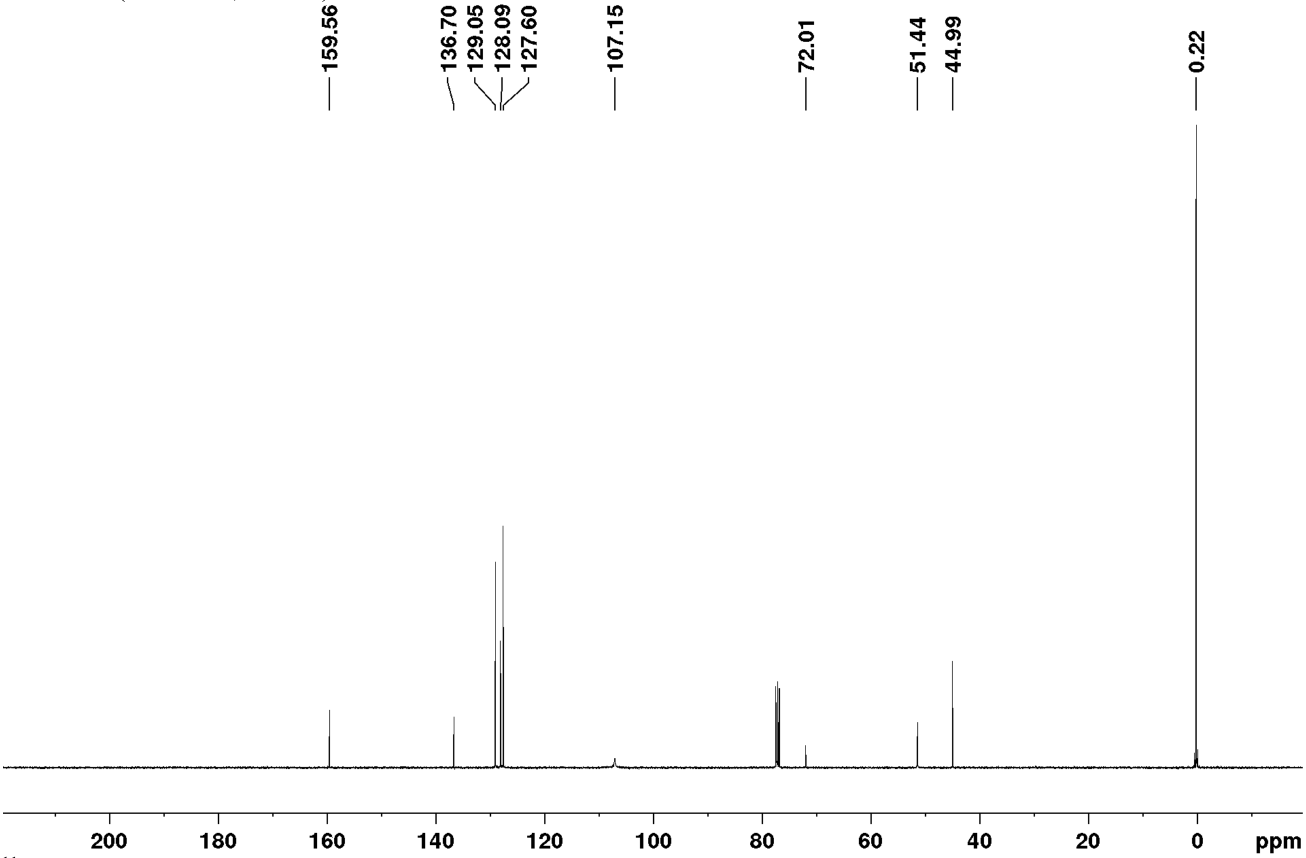

^{11}B NMR (160 MHz; $CDCl_3$)

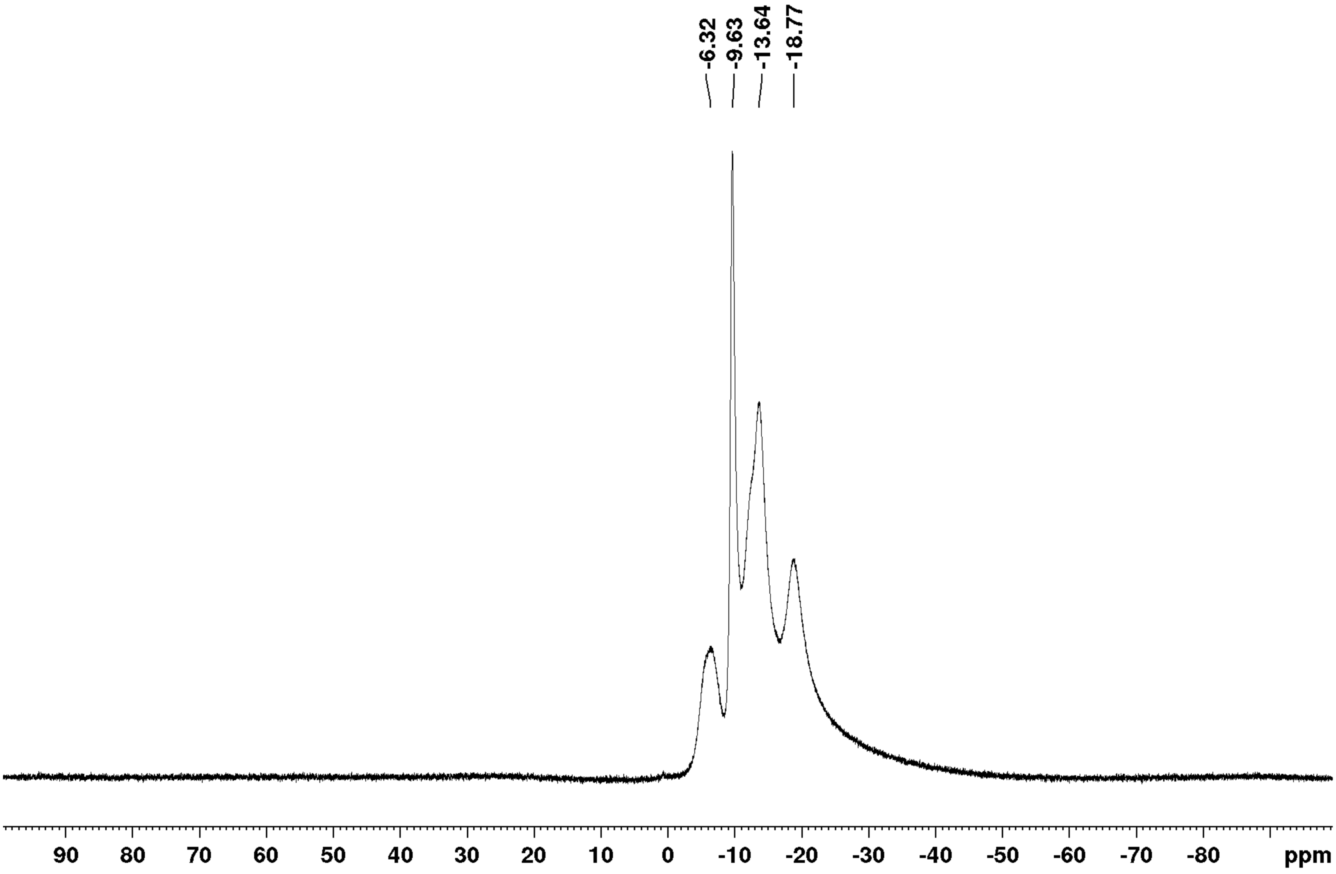

1-isobutylaminocarboxyl-9,10-bis((trimethylsilyl)ethynyl)-1,7-dicarba-*closo*-dodecaborane (24b)

^{1}H NMR (400 MHz; $CDCl_3$)

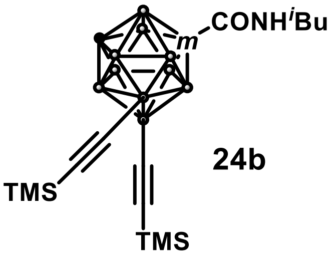

24b

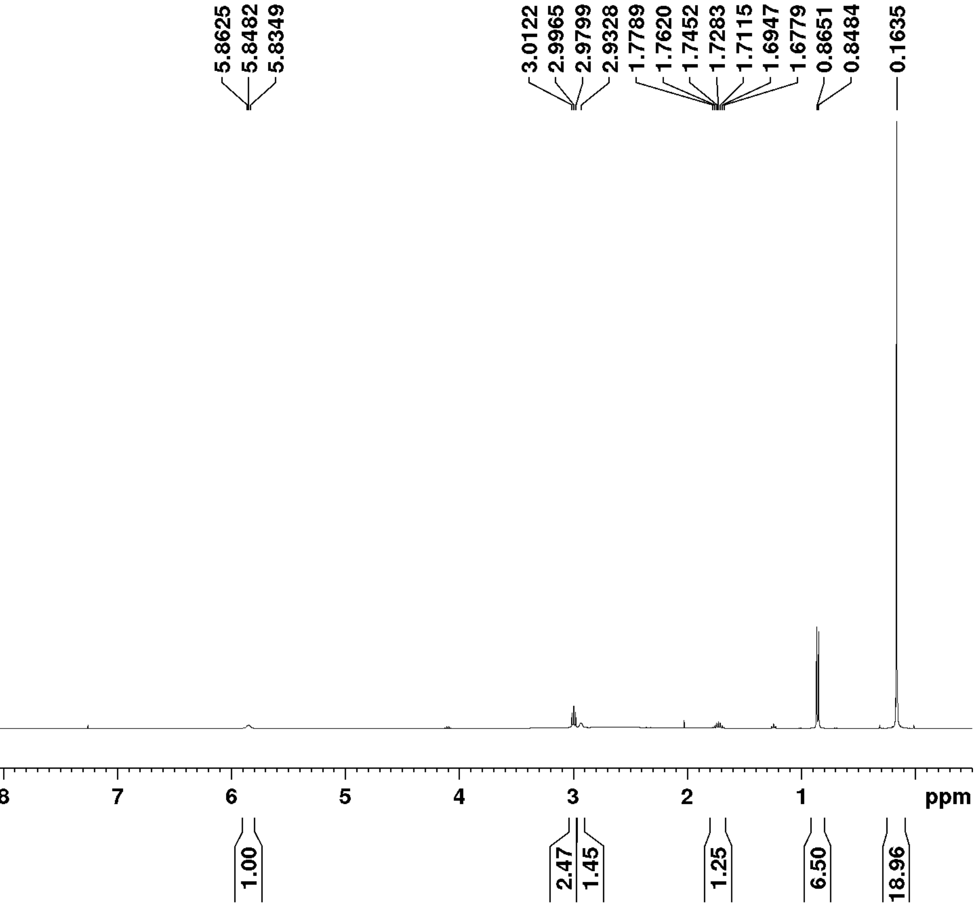

^{13}C NMR (125 MHz; $CDCl_3$)

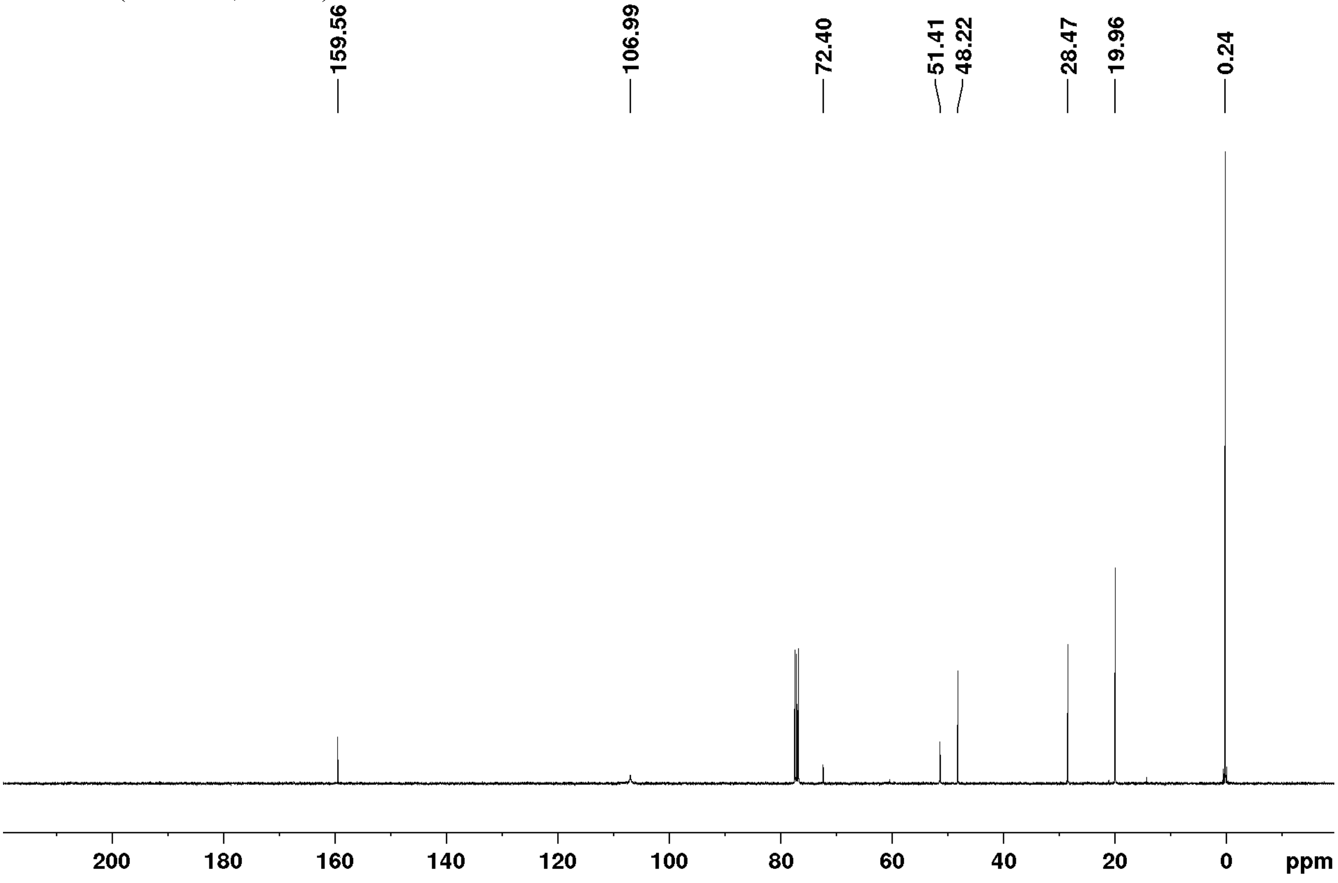

^{11}B NMR (160 MHz; $CDCl_3$)

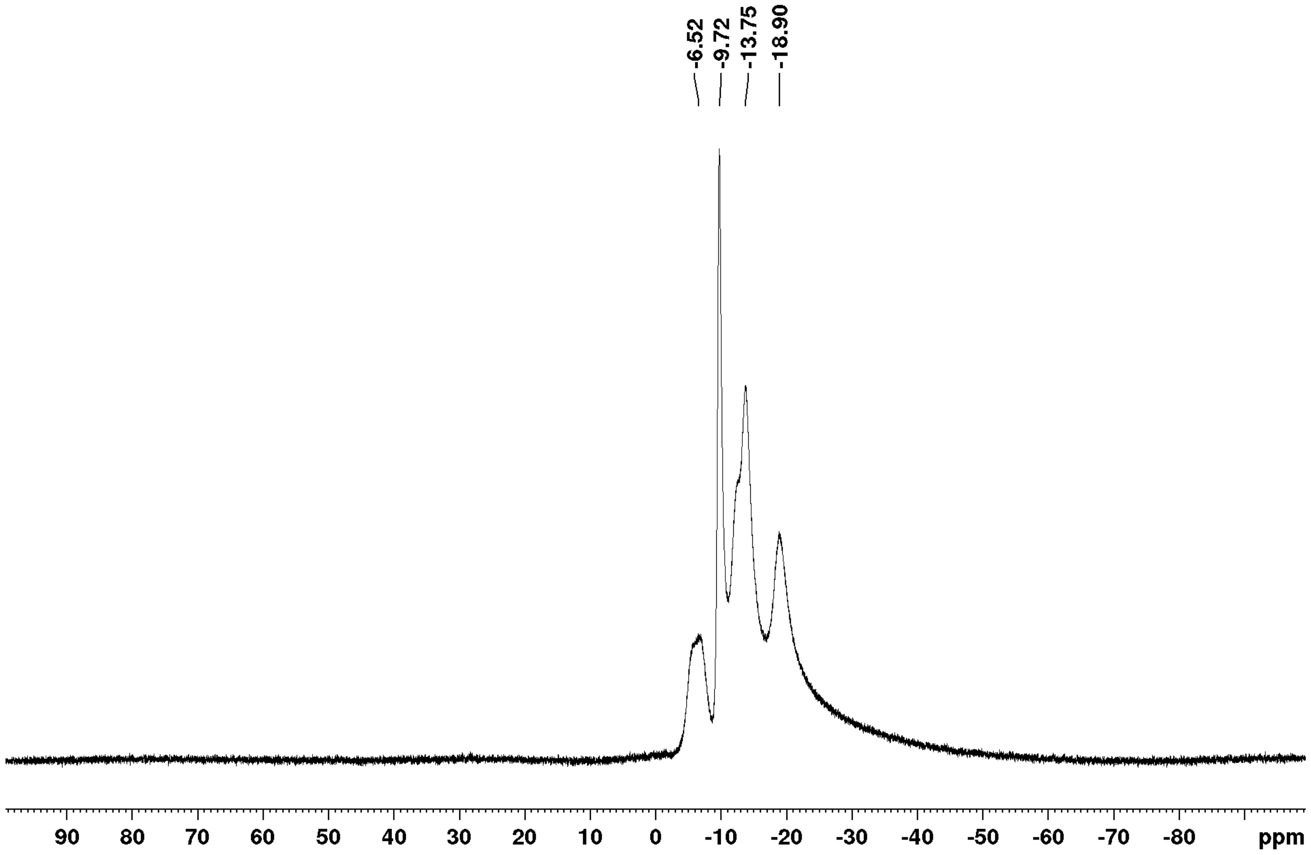

1-benzylaminocarboxyl-9,10-bis((trimethylsilyl)ethynyl)-1,7-dicarba-*closo*-dodecaborane (25)
^{1}H NMR (500 MHz; $CDCl_3$)

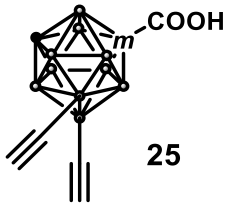

25

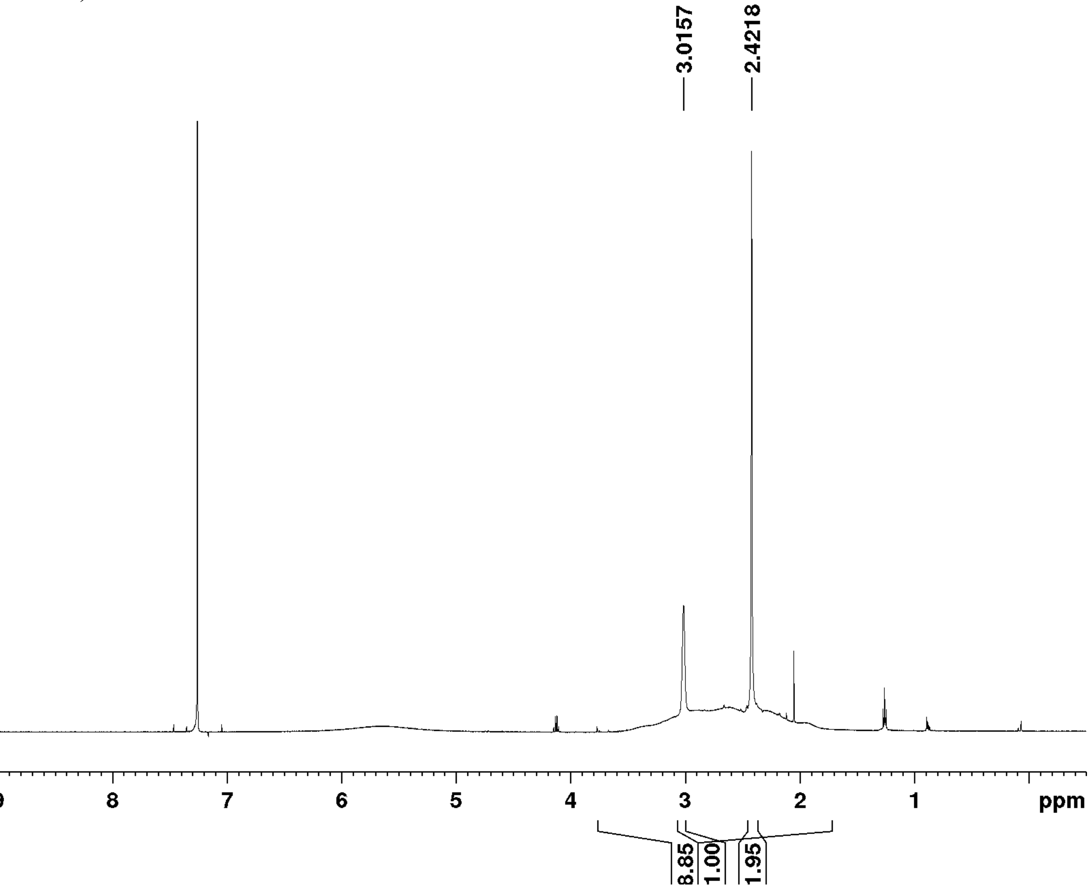

^{13}C NMR (125 MHz; $CDCl_3$)

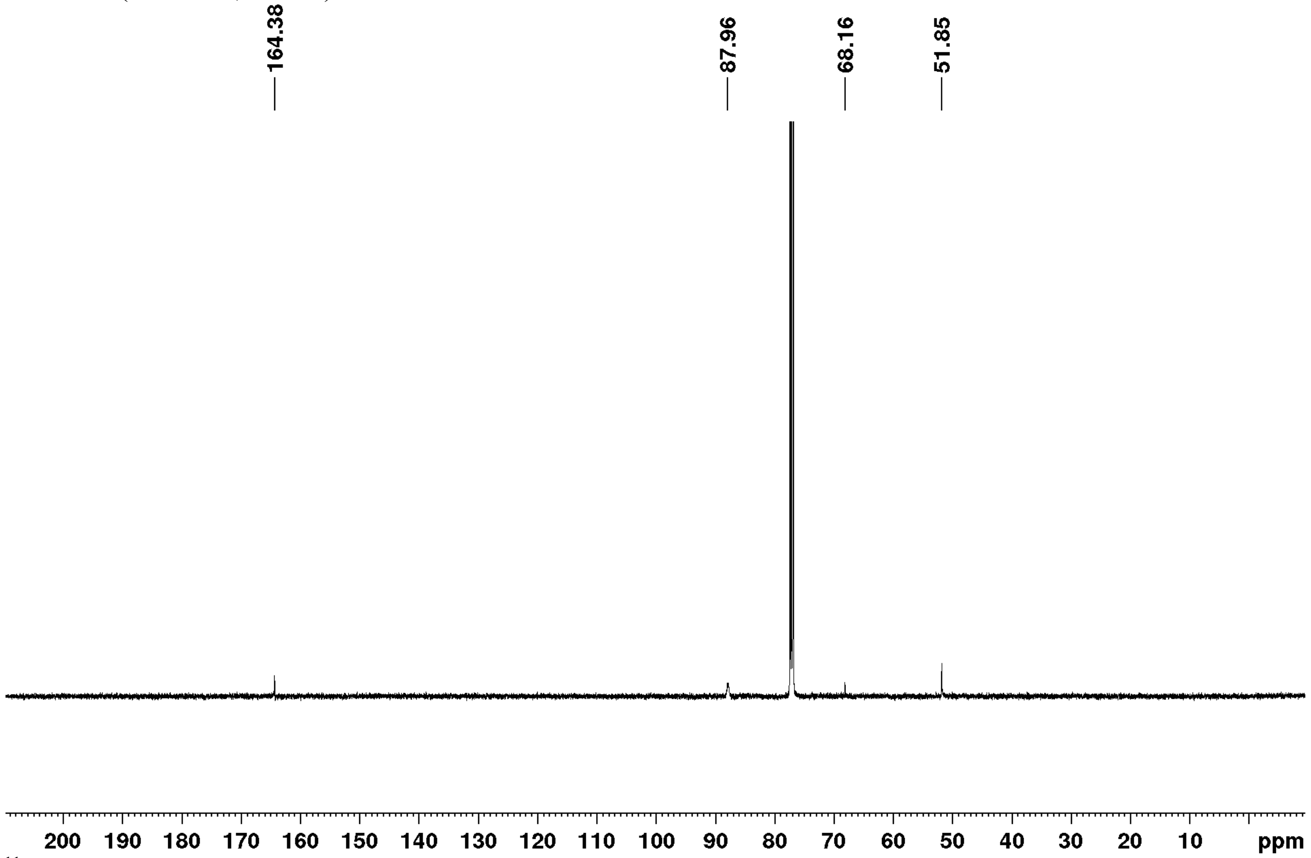

^{11}B NMR (160 MHz; $CDCl_3$)

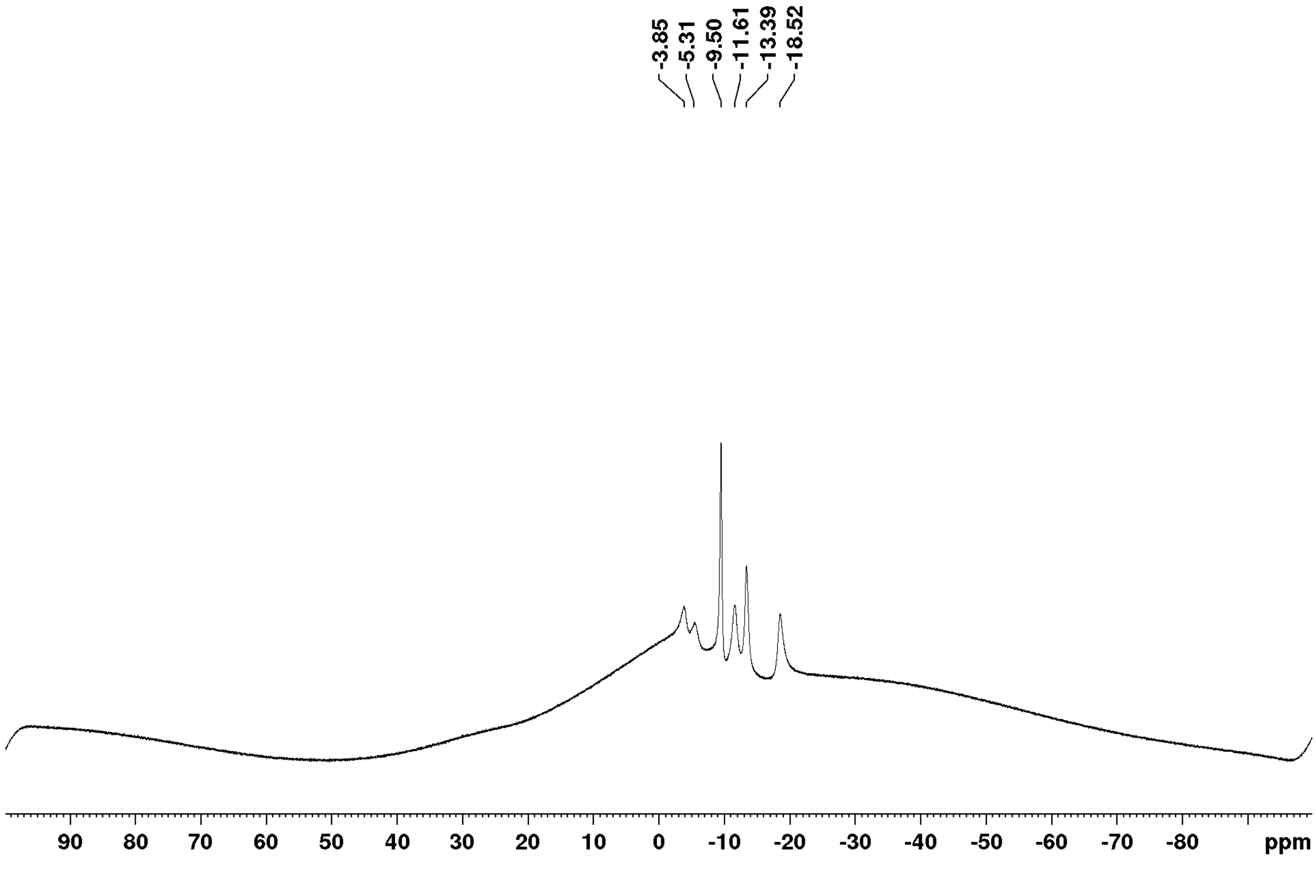

1-benzylaminocarboxyl-9,10-diethynyl-1,7-dicarba-*closo*-dodecaborane (S6a)

^{1}H NMR (400 MHz; $CDCl_3$)

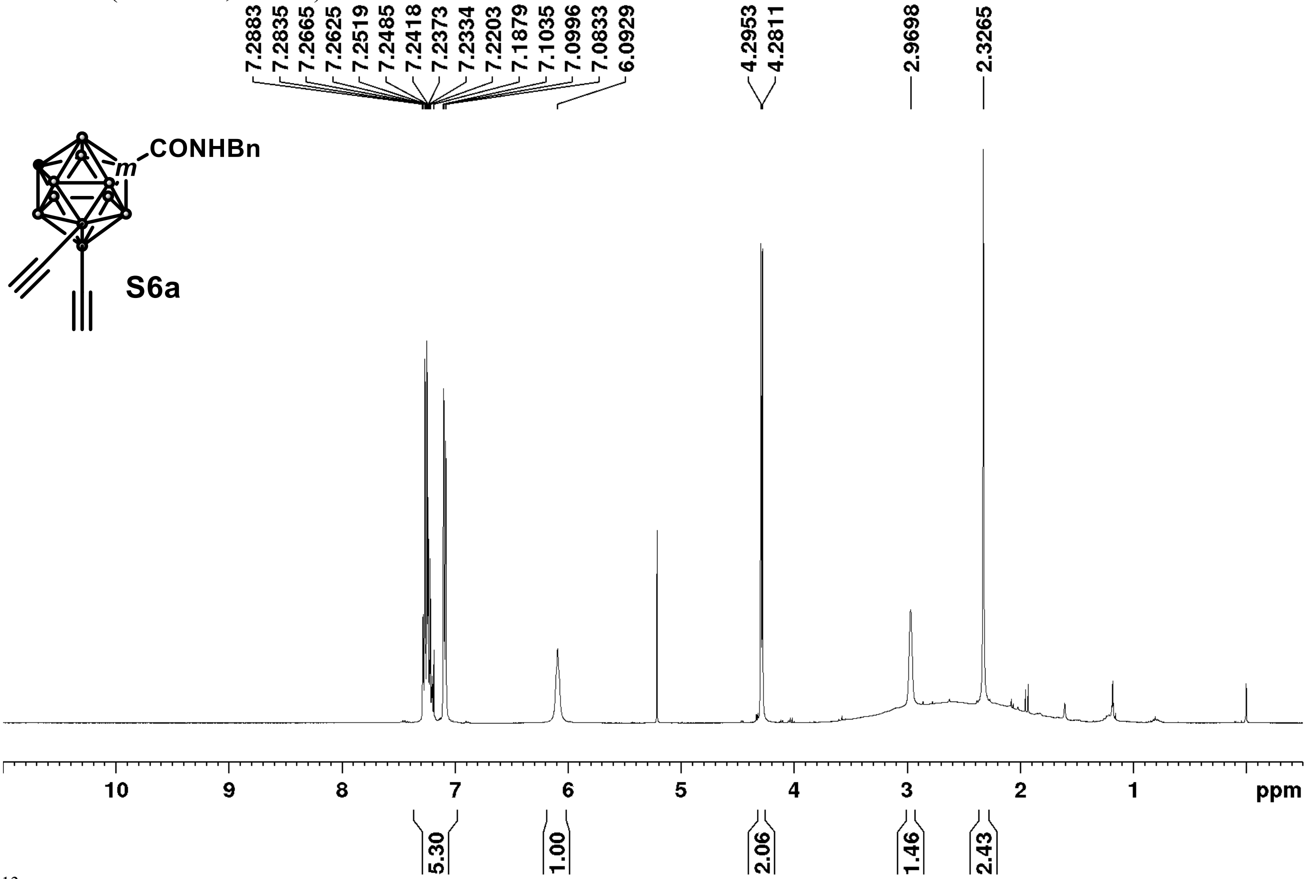

^{13}C NMR (125 MHz; $CDCl_3$)

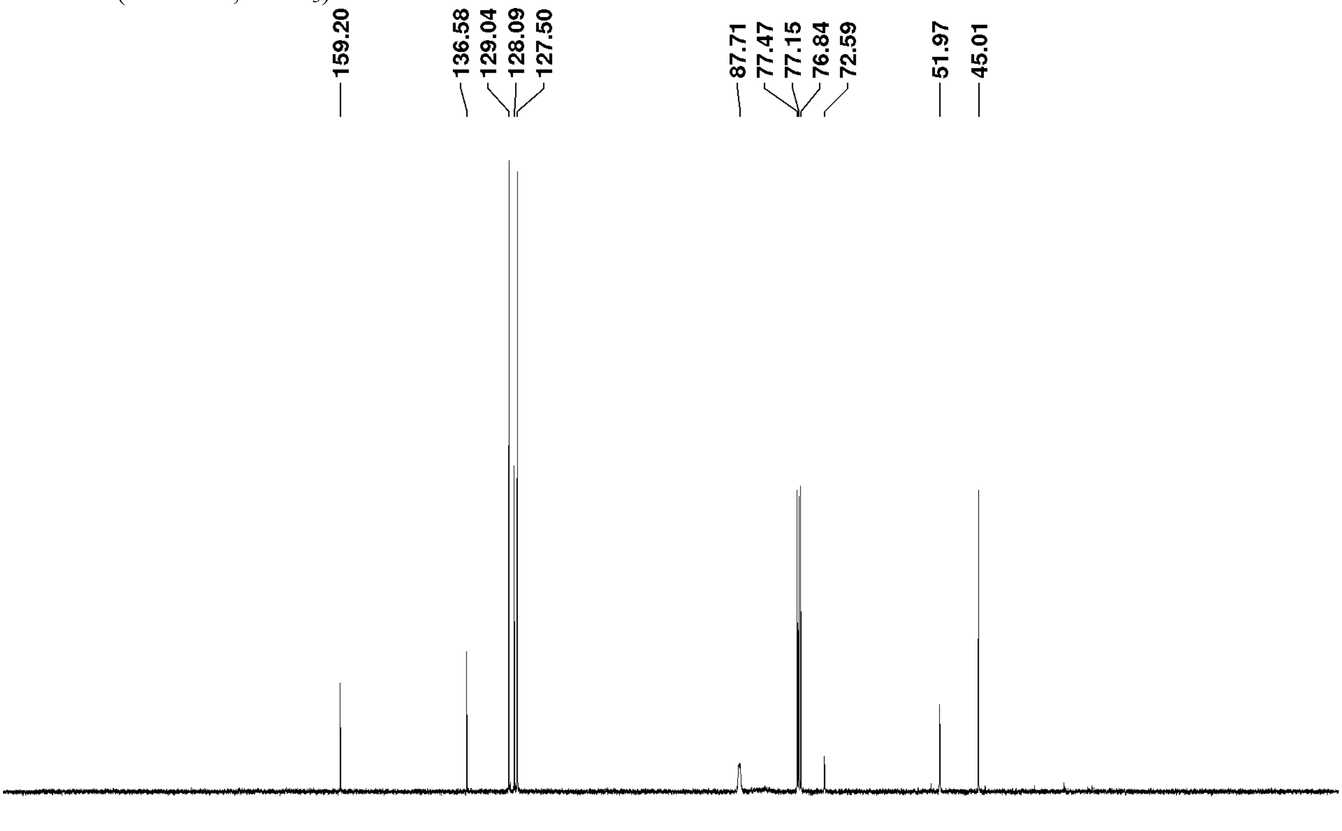

^{11}B NMR (160 MHz; $CDCl_3$)

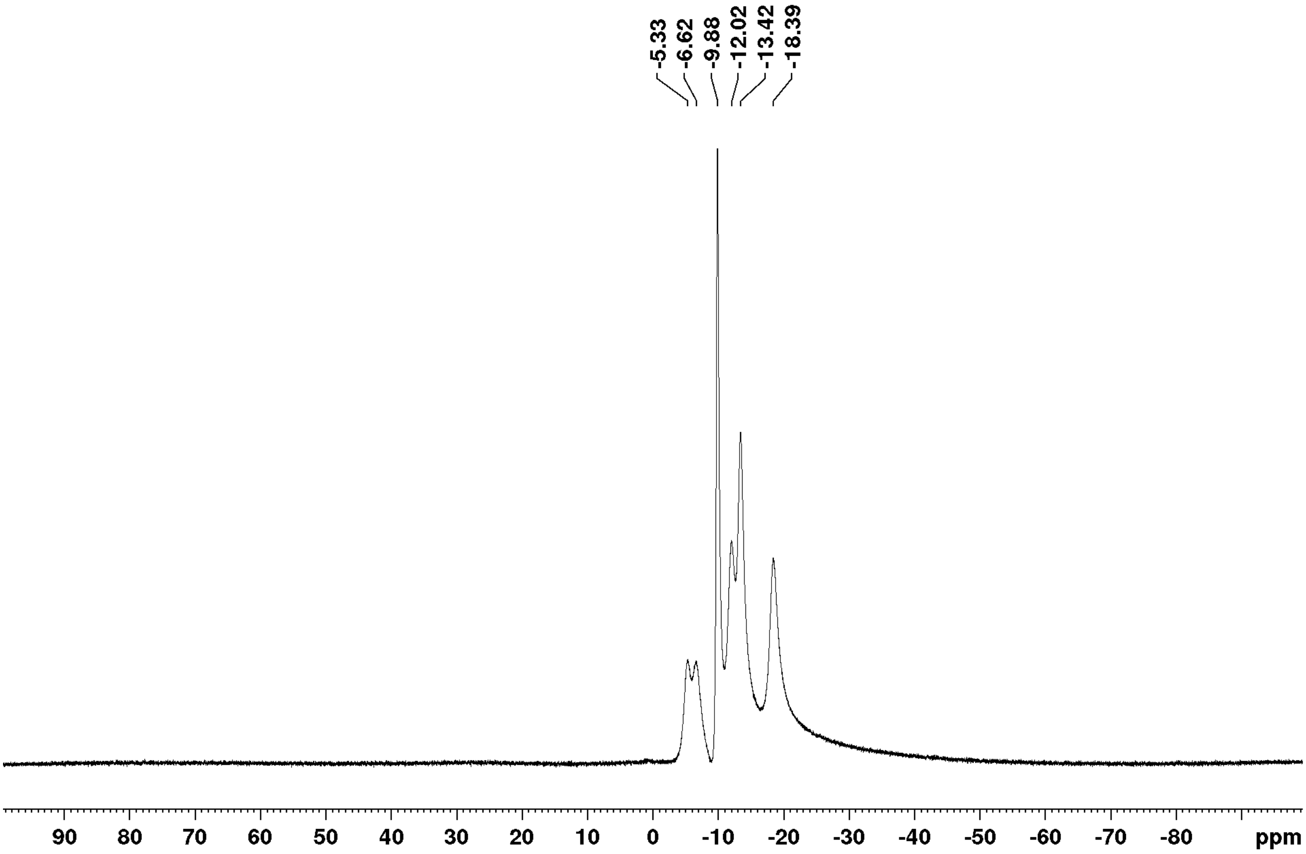

1-isobutylaminocarboxyl-9,10-diethynyl-1,7-dicarba-*closo*-dodecaborane (S6b)
^{1}H NMR (400 MHz; $CDCl_3$)

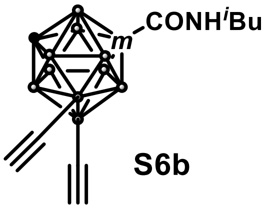

S6b

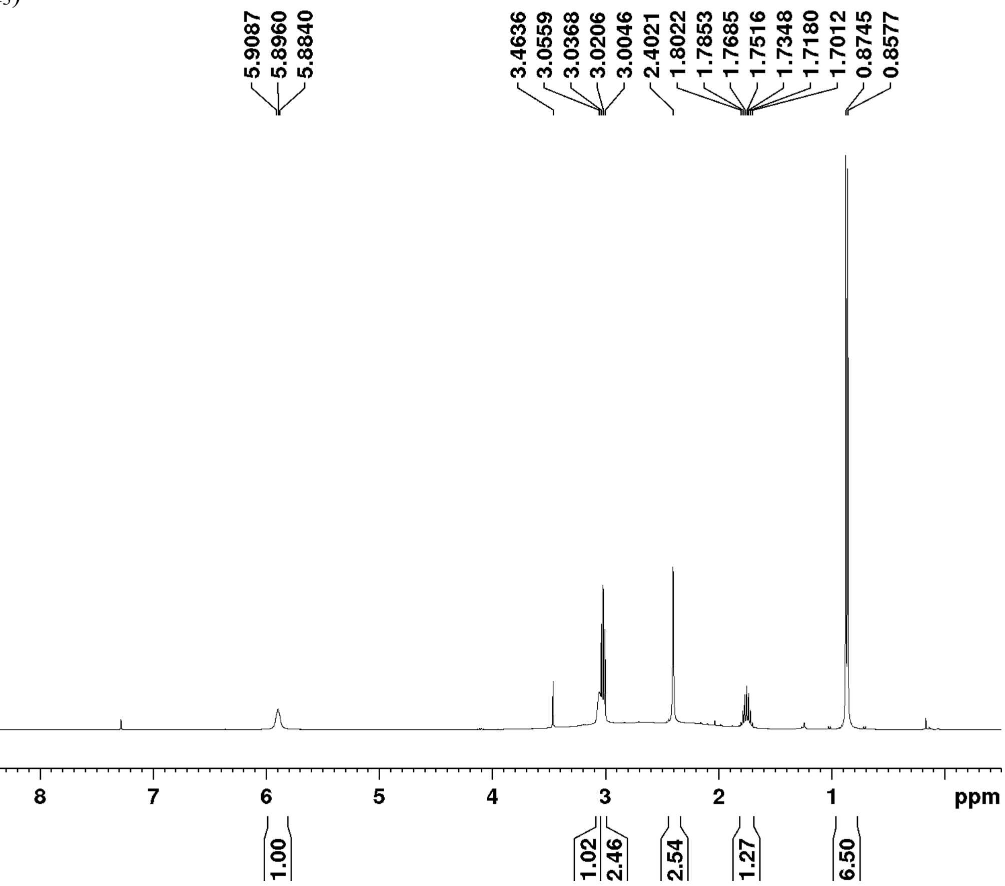

^{13}C NMR (125 MHz; $CDCl_3$)

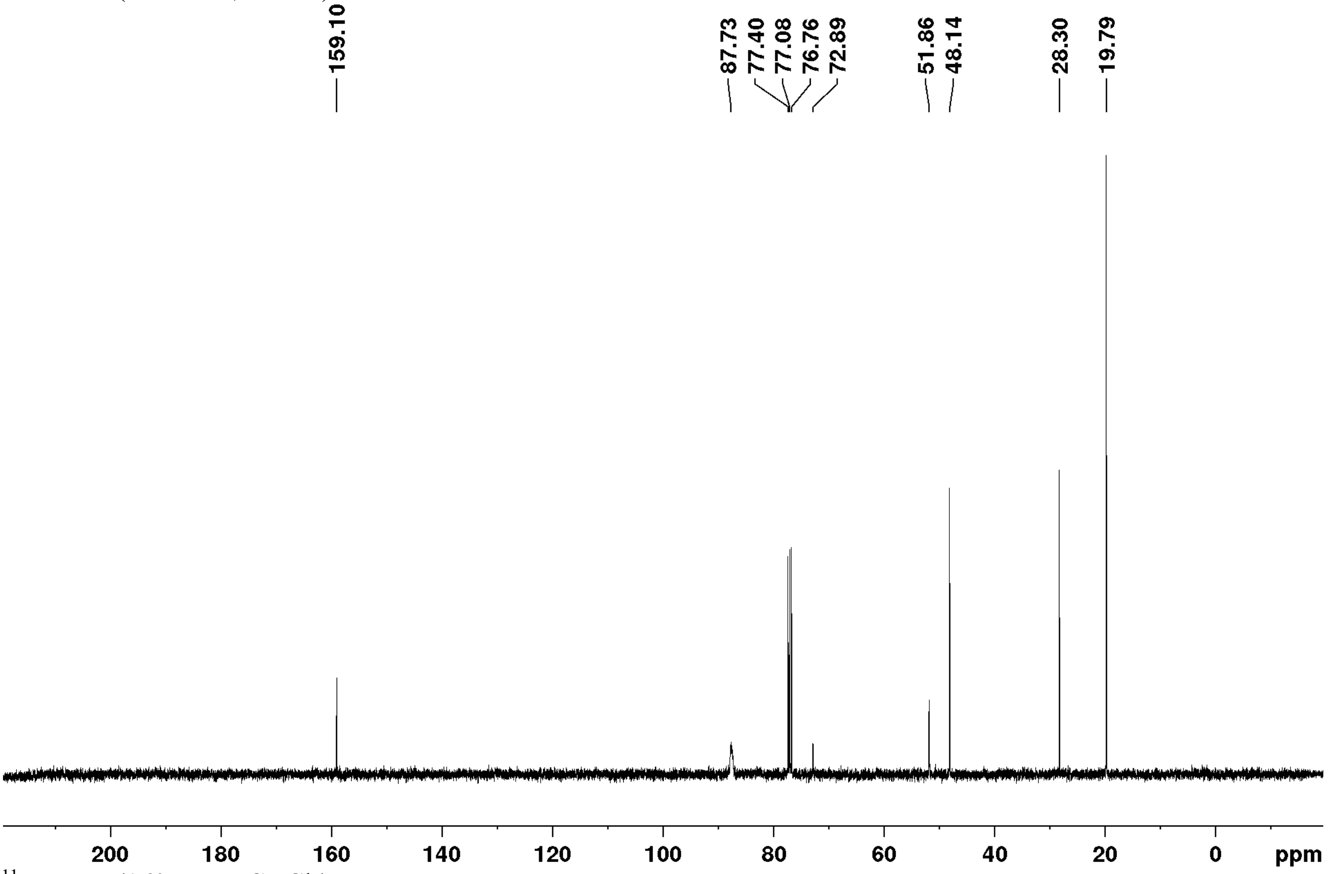

^{11}B NMR (160 MHz; $CDCl_3$)

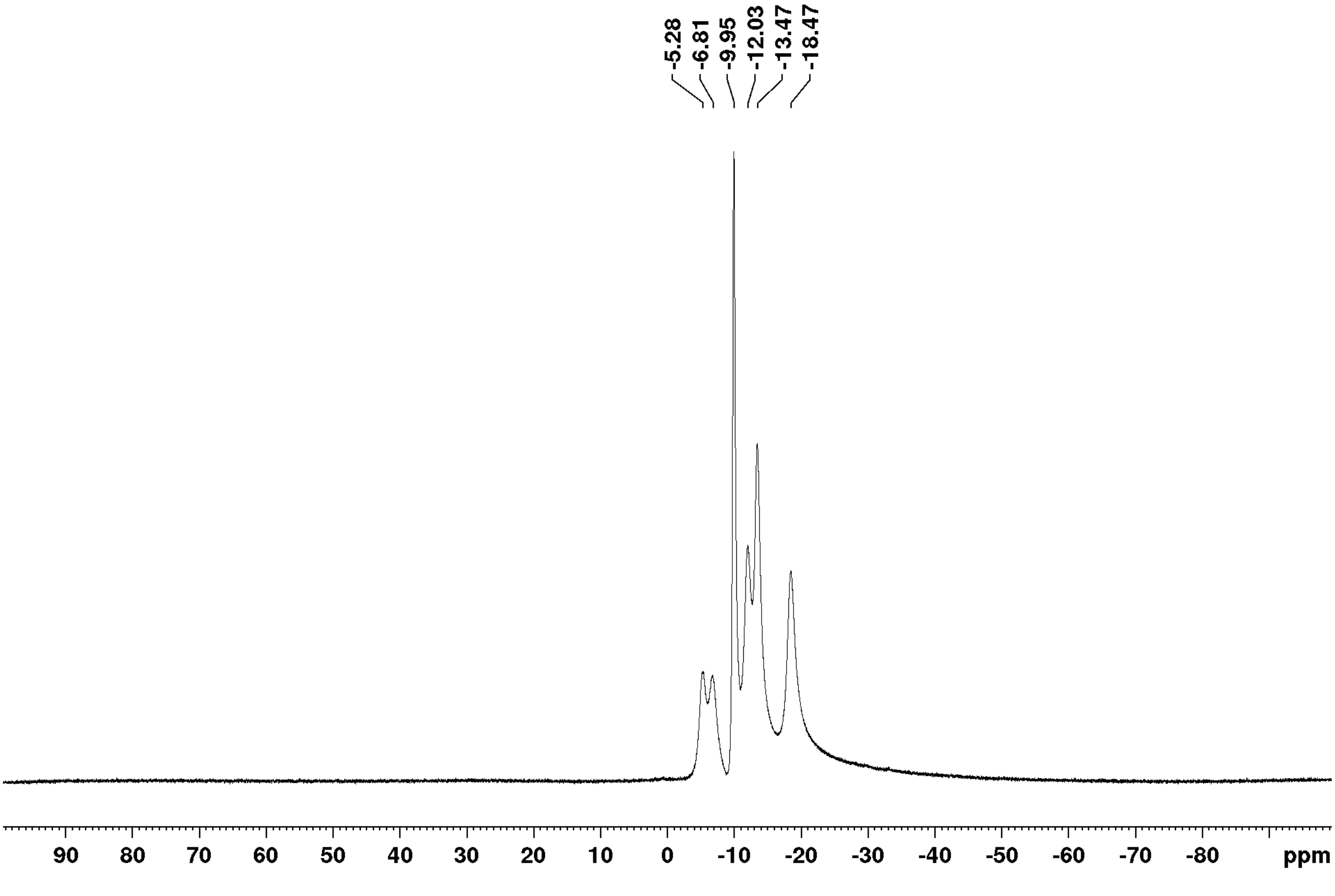

1-benzylaminocarboxyl-9,10-bis((1*H*-1,2,3-triazol-4-yl)benzylcarbamoyl)-1,7-dicarba-*closo*-dodecaborane (IVa)

^{1}H NMR (500 MHz; $CDCl_3$)

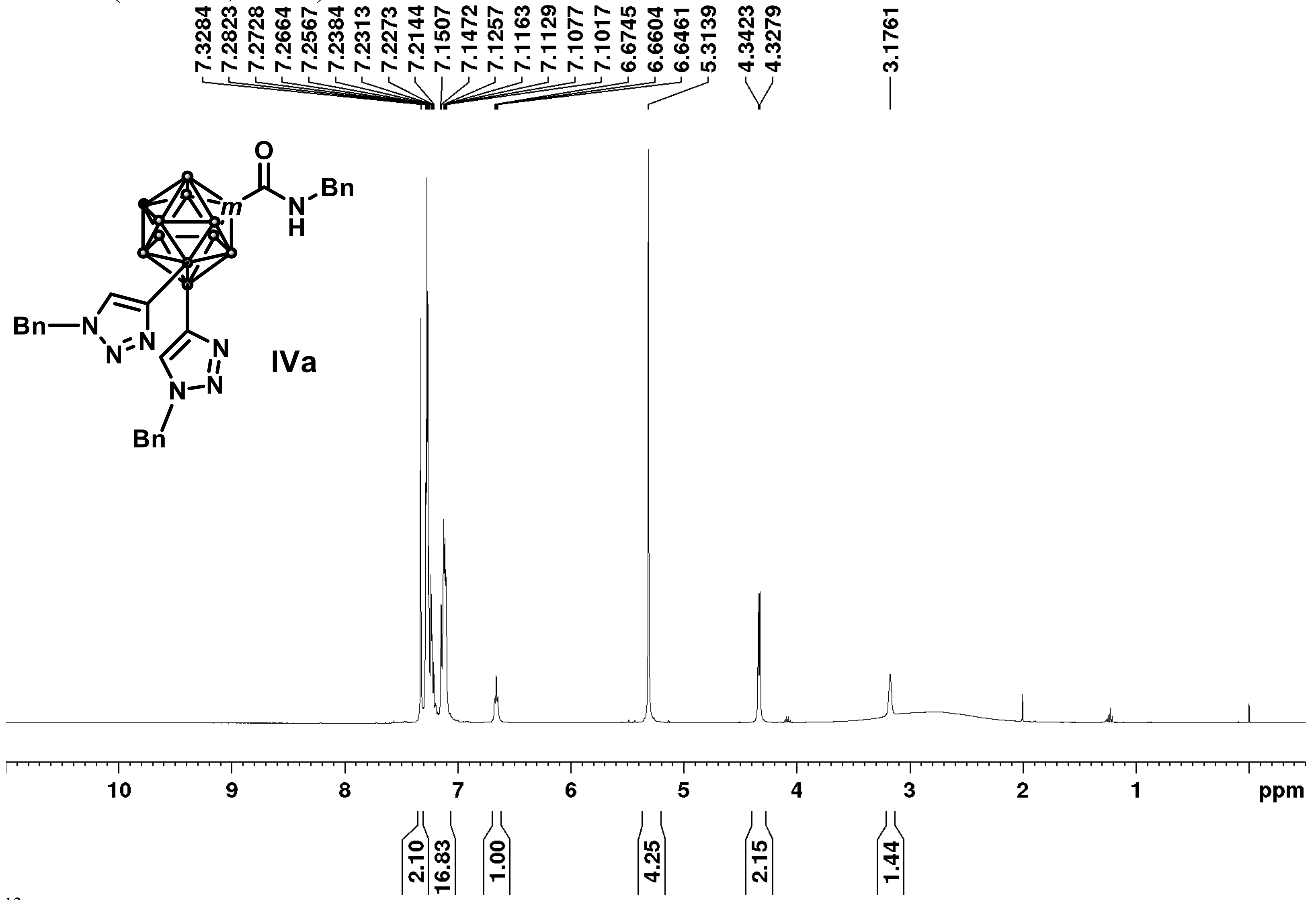

^{13}C NMR (125 MHz; $CDCl_3$)

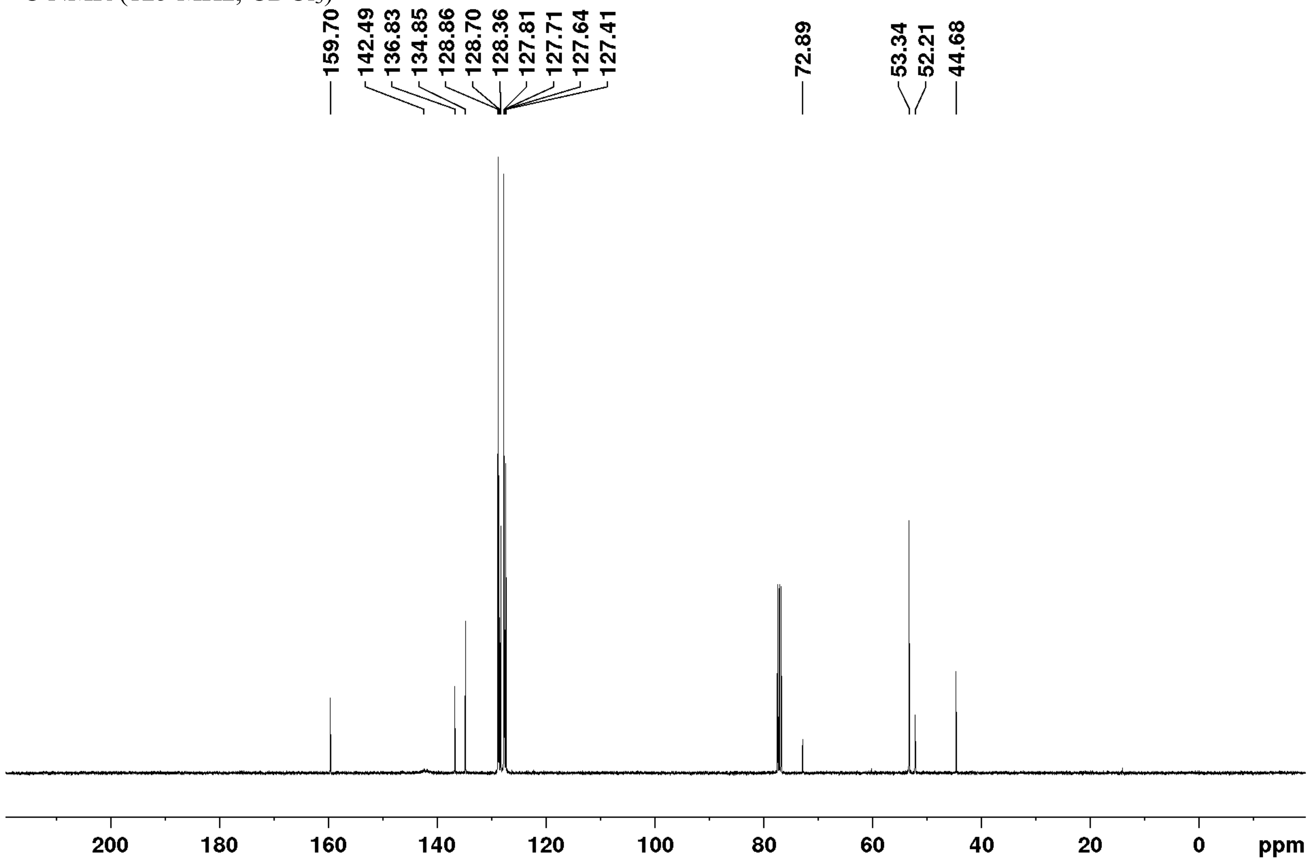

^{11}B NMR (160 MHz; CDCl$_3$)

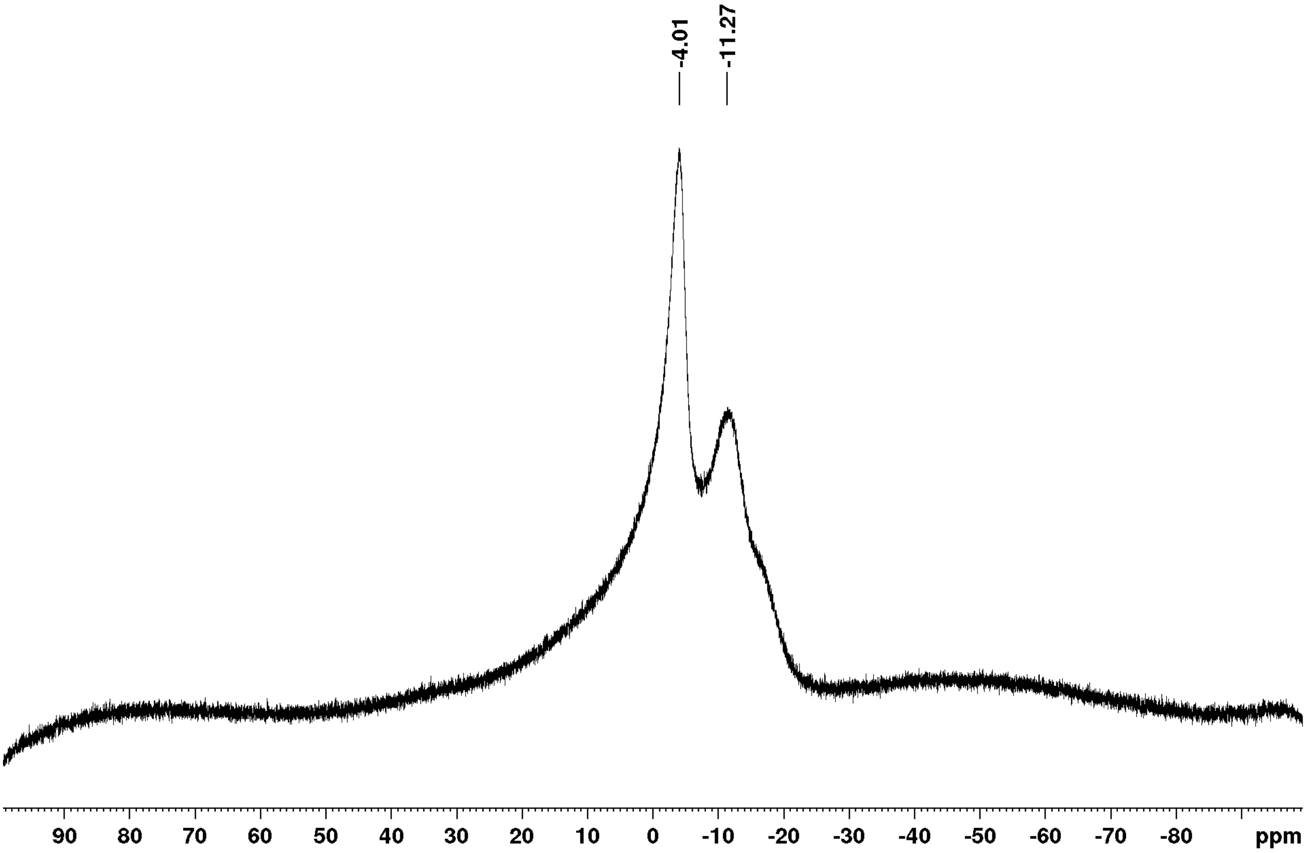

1-benzylaminocarboxyl-9,10-bis((1*H*-1,2,3-triazol-4-yl)isobutylcarbamoyl)-1,7-dicarba-*closo*-dodecaborane (IVb)

^{1}H NMR (500 MHz; CDCl$_3$)

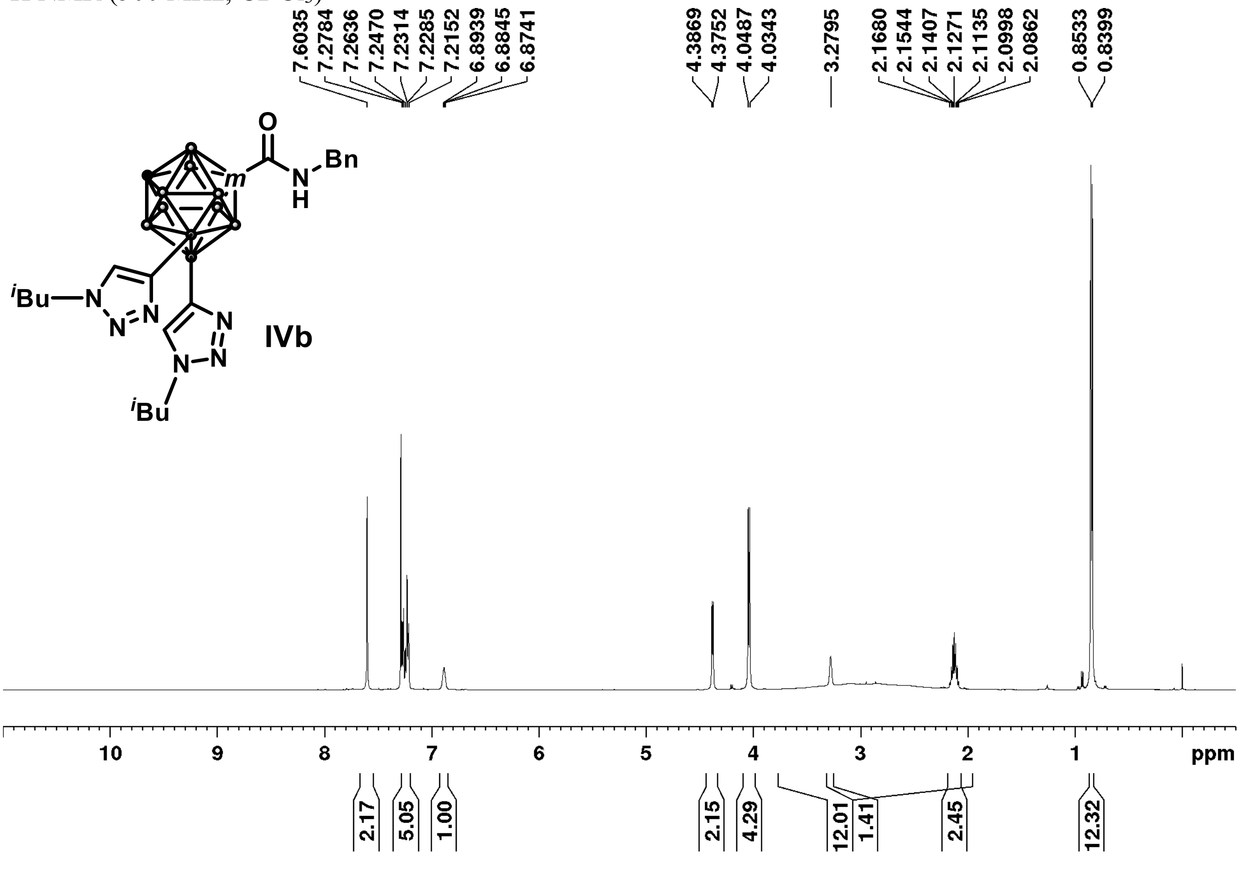

^{13}C NMR (125 MHz; $CDCl_3$)

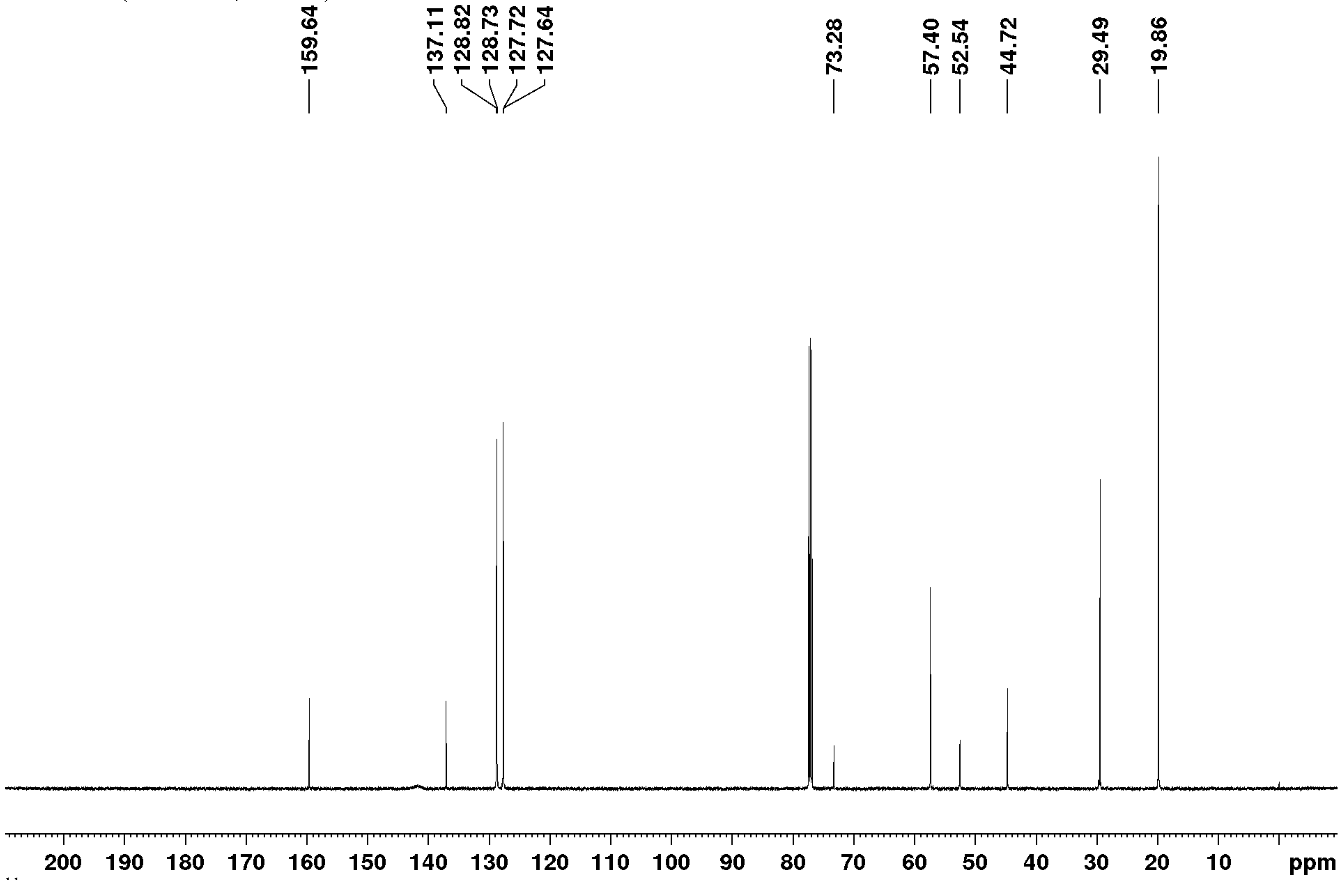

^{11}B NMR (160 MHz; $CDCl_3$)

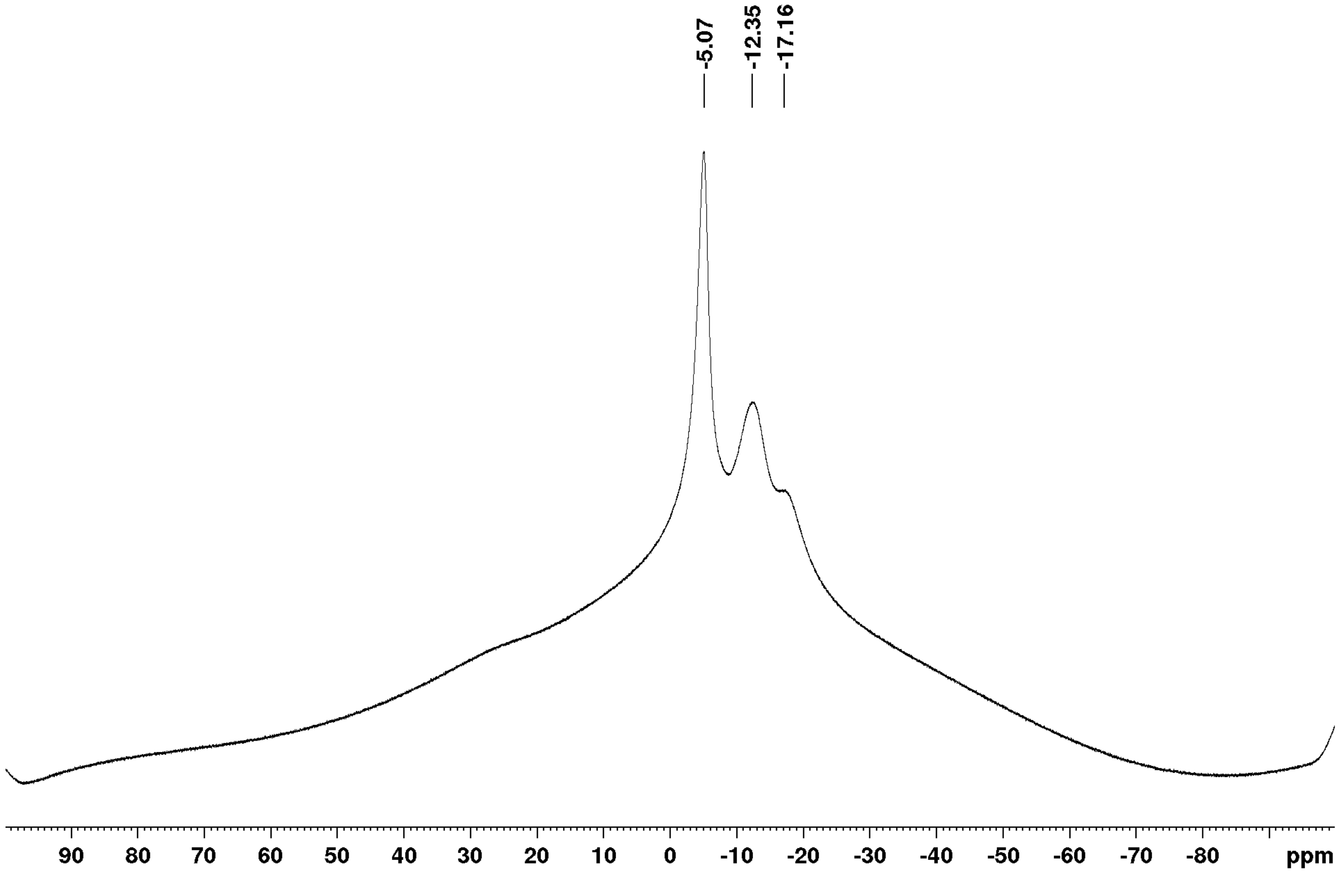

1-isobutylaminocarboxyl-9,10-bis((1*H*-1,2,3-triazol-4-yl)benzylcarbamoyl)-1,7-dicarba-*closo*-dodecaborane (IVc)

^{1}H NMR (500 MHz; $CDCl_3$)

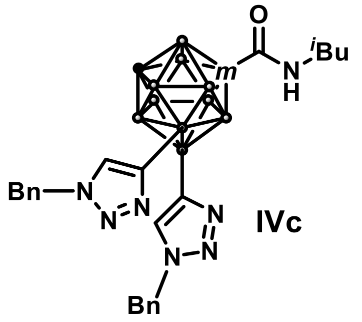

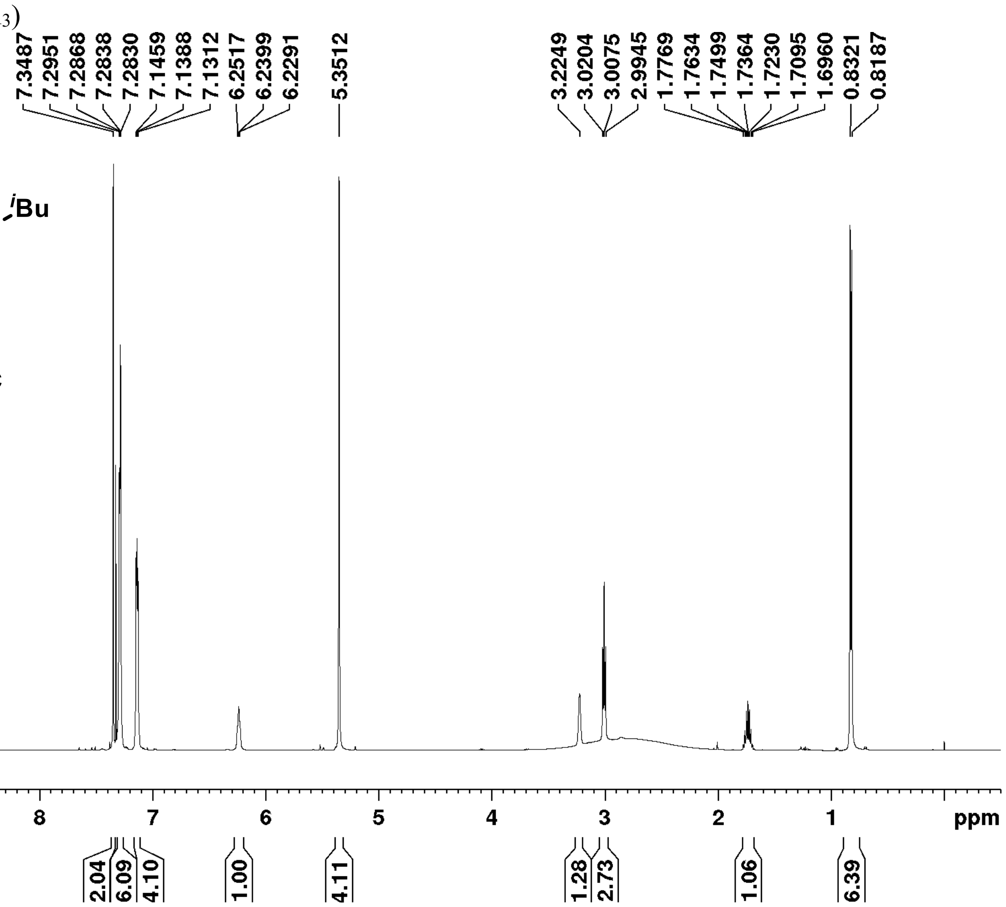

^{13}C NMR (125 MHz; $CDCl_3$)

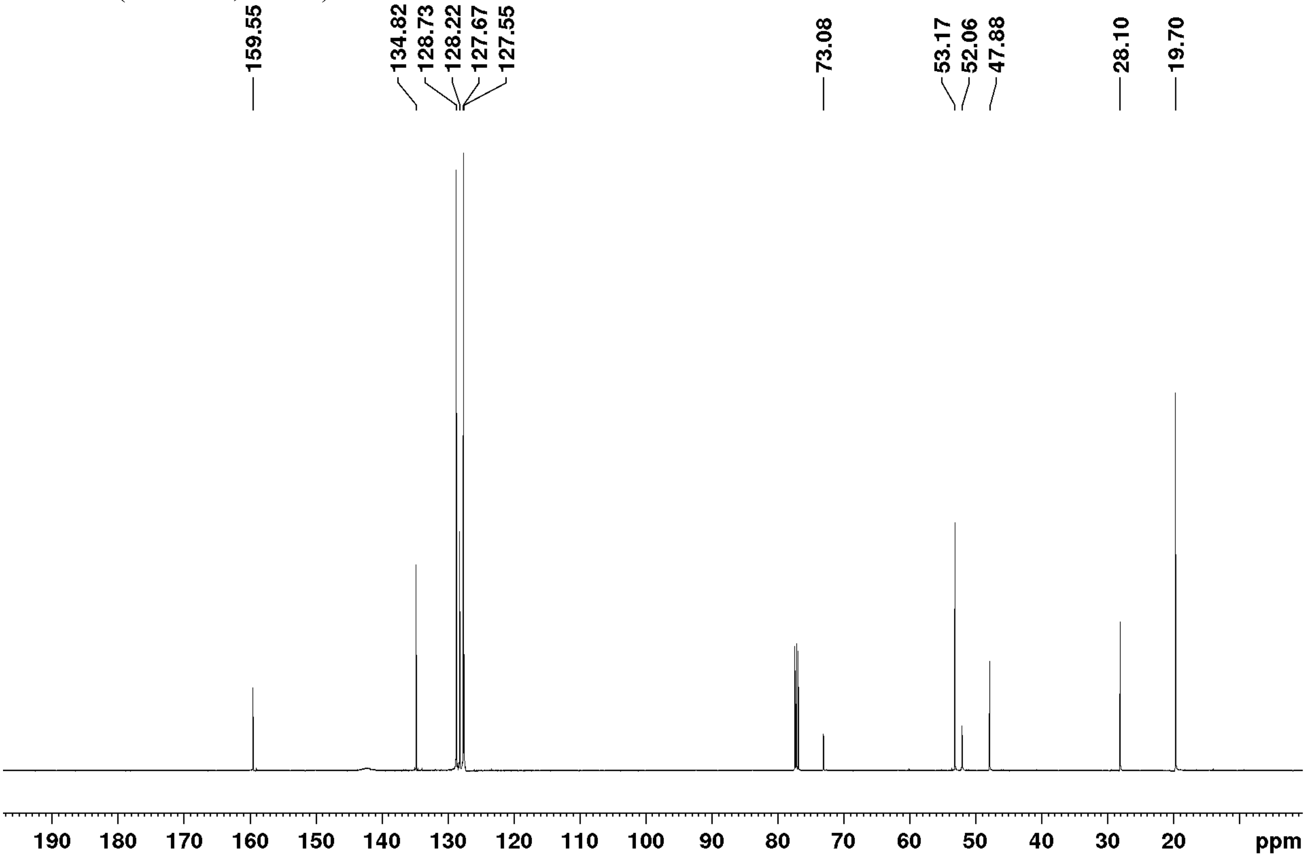

^{11}B NMR (160 MHz; $CDCl_3$)

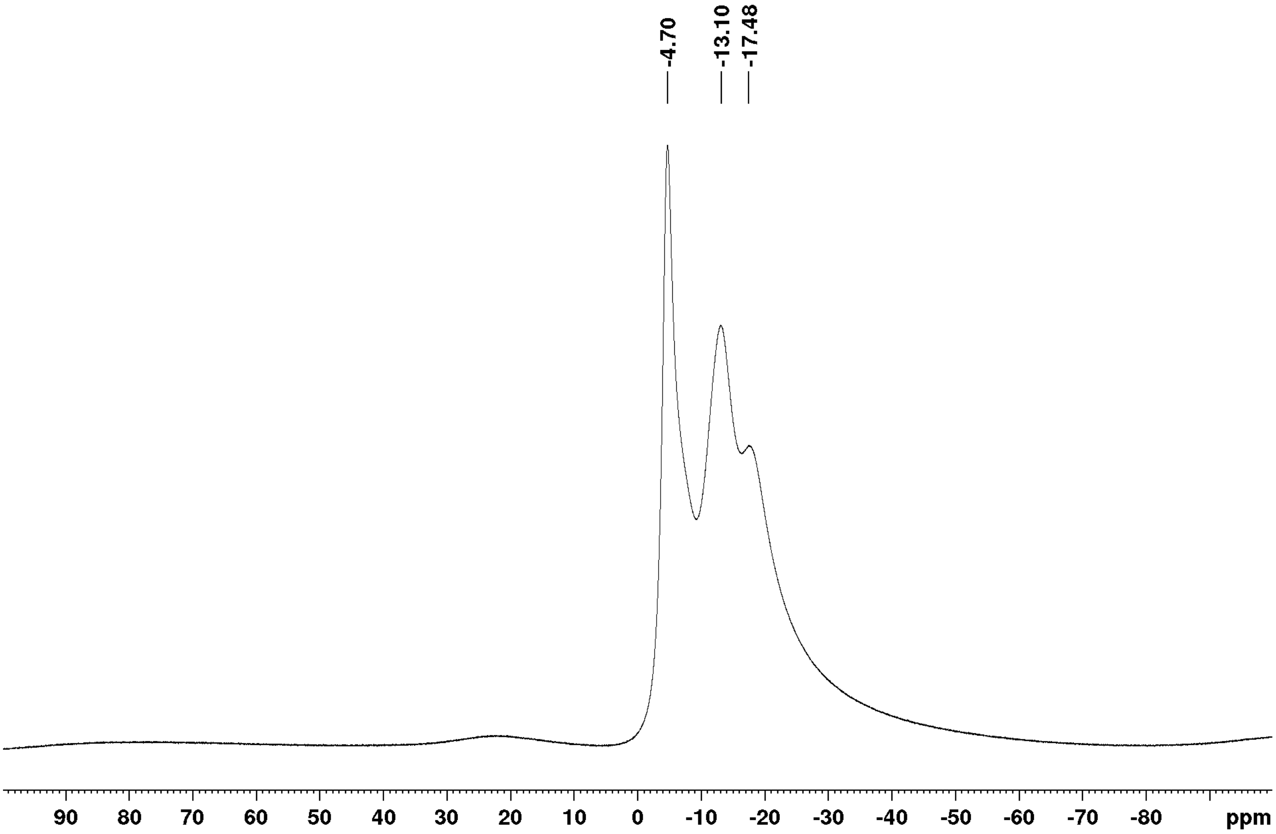

1-isobutylaminocarboxyl-9,10-bis((1*H*-1,2,3-triazol-4-yl)isobutylcarbamoyl)-1,7-dicarba-*closo*-dodecaborane (IVd)

^{1}H NMR (500 MHz; $CDCl_3$)

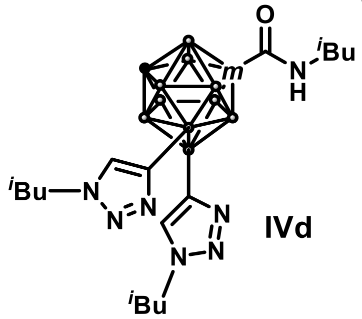

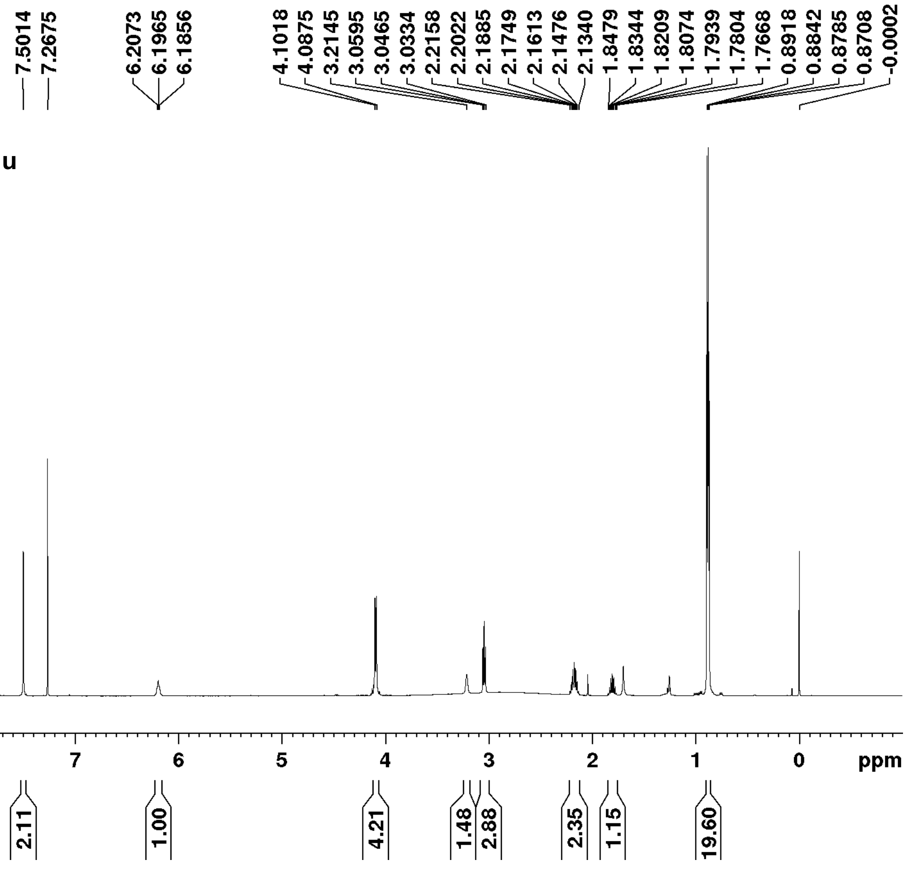

^{13}C NMR (125 MHz; $CDCl_3$)

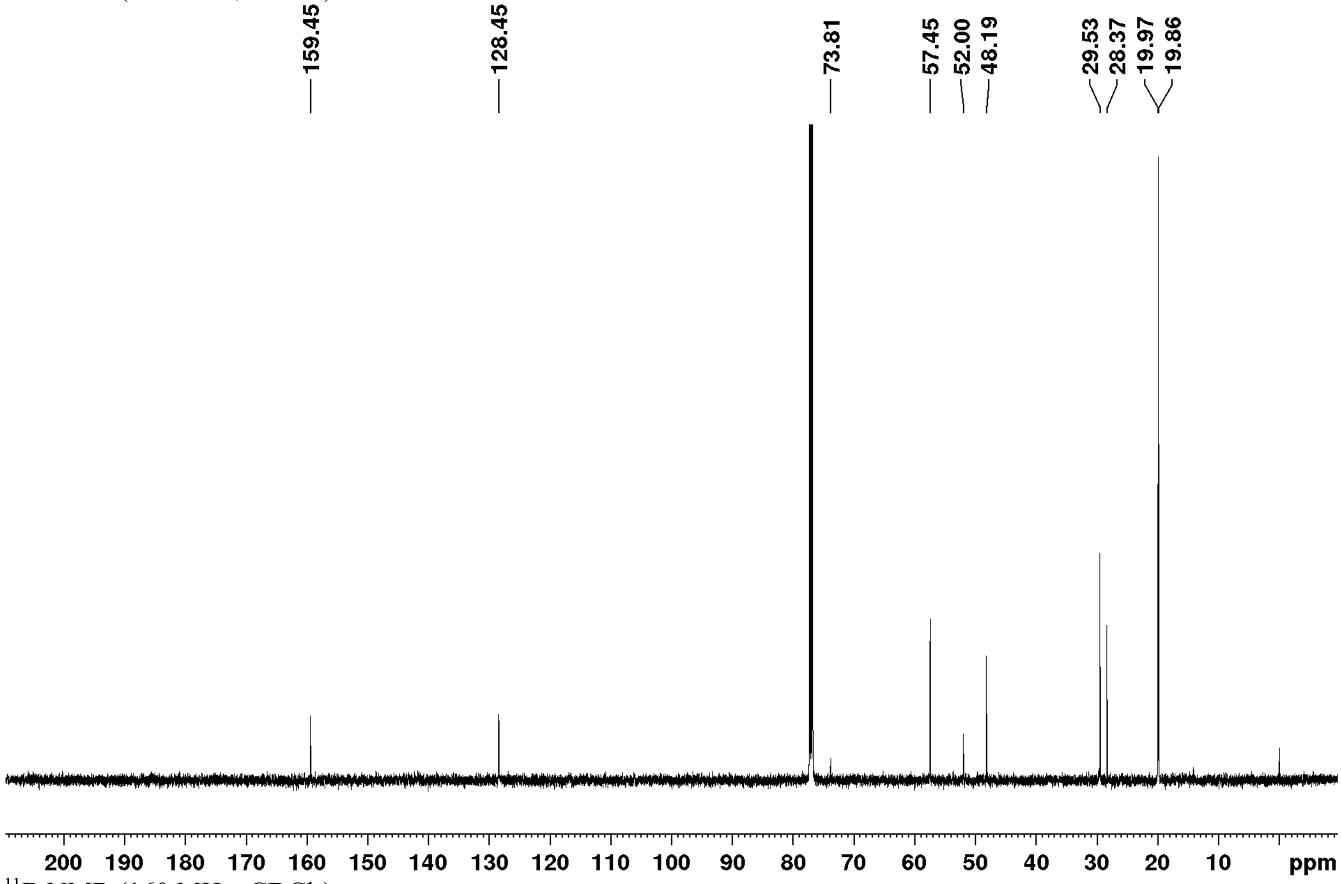

^{11}B NMR (160 MHz; $CDCl_3$)

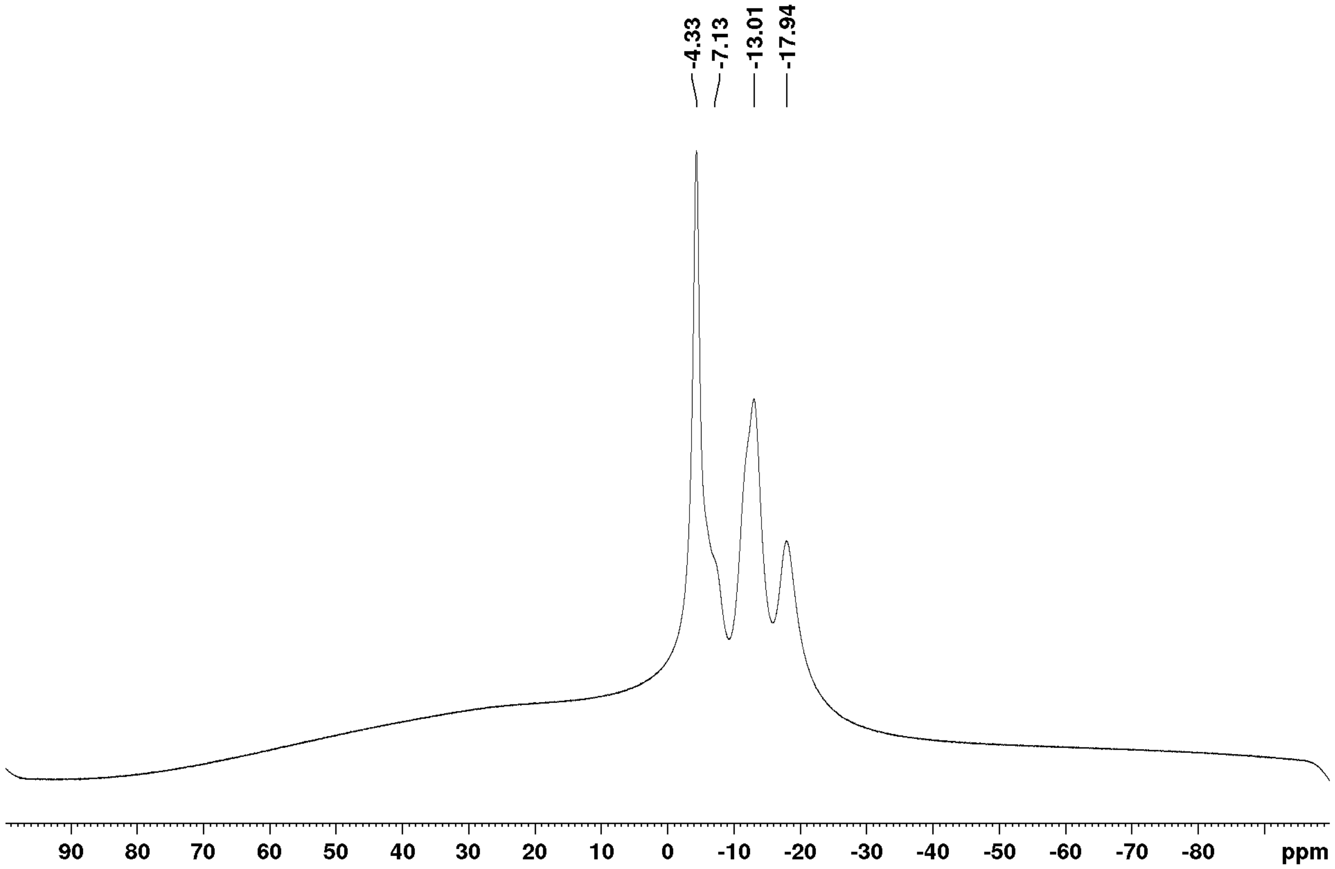

2-(3-((4-methoxybenzyl)oxy)prop-1-yn-1-yl)-1,12-dicarba-*closo*-dodecaborane (26)

^{1}H NMR (500 MHz; $CDCl_3$)

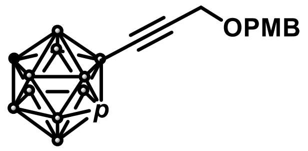

26

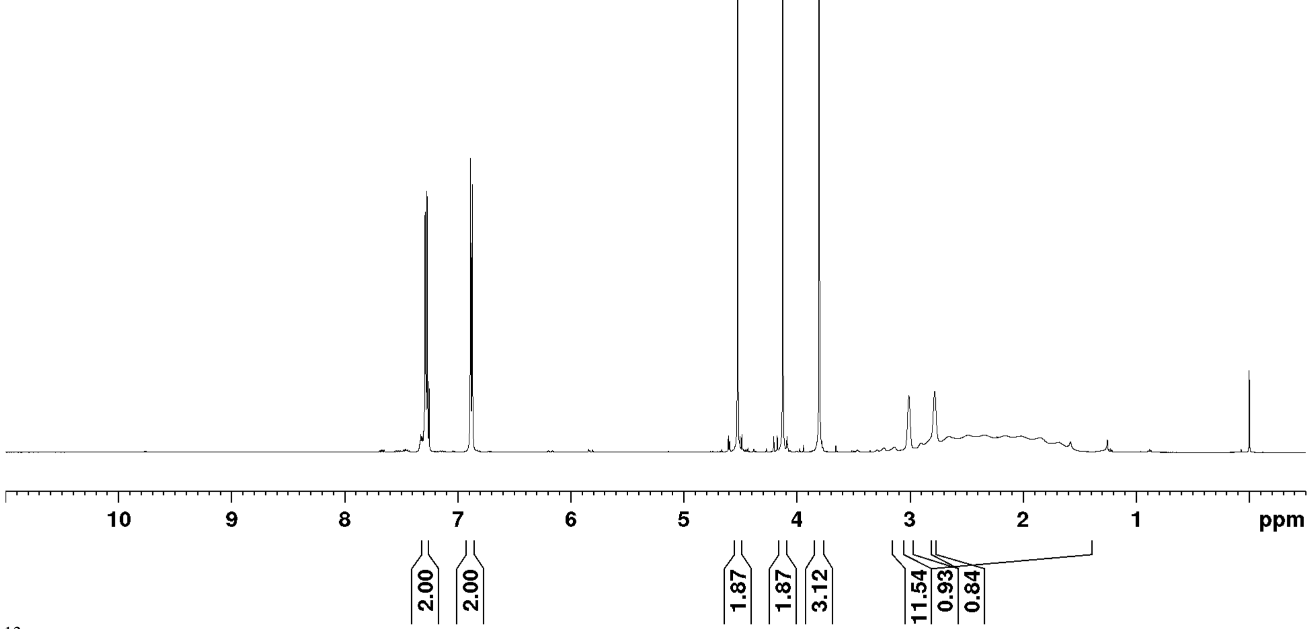

^{13}C NMR (125 MHz; $CDCl_3$)

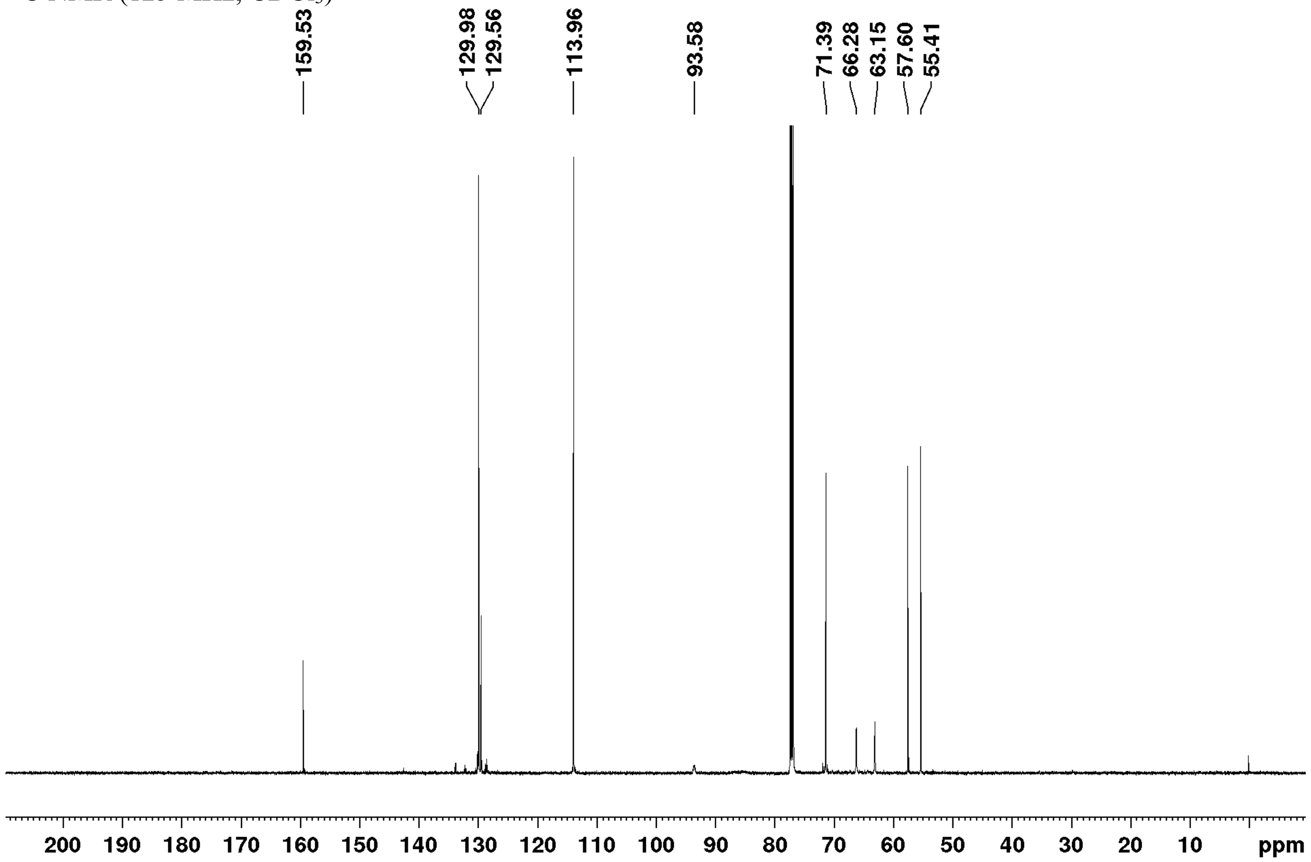

^{11}B NMR (160 MHz; $CDCl_3$)

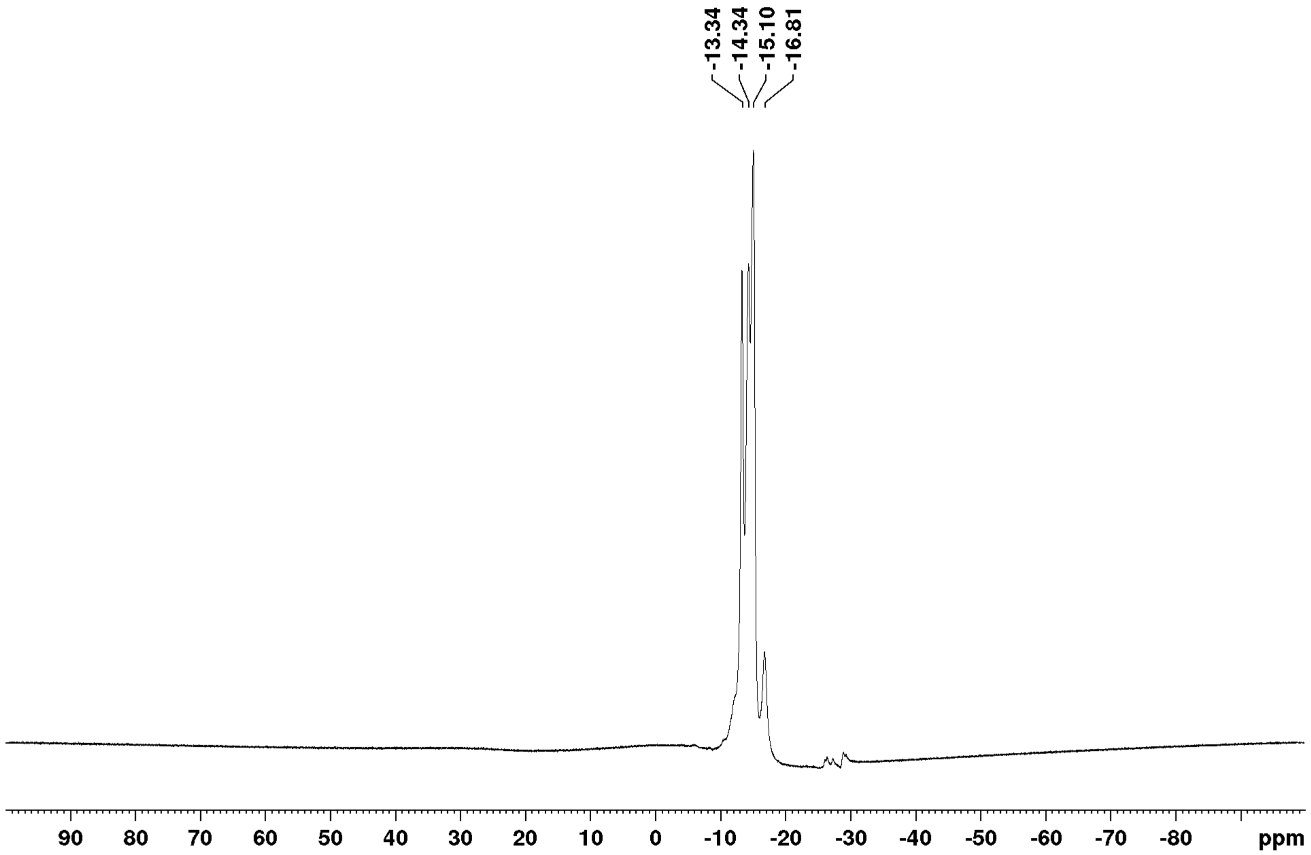

12-((*tert*-butyl)dimethylsilyl)hydroxymethyl)-7-(3-((4-methoxybenzyl)oxy)prop-1-yn-1-yl)-1,12-dicarba-*closo*-dodecaboranyl-1-carboxylic acid (27)

^{1}H NMR (400 MHz; $CDCl_3$)

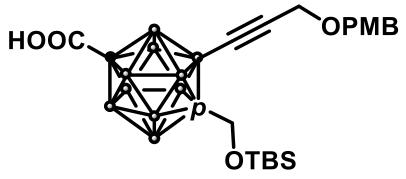

27

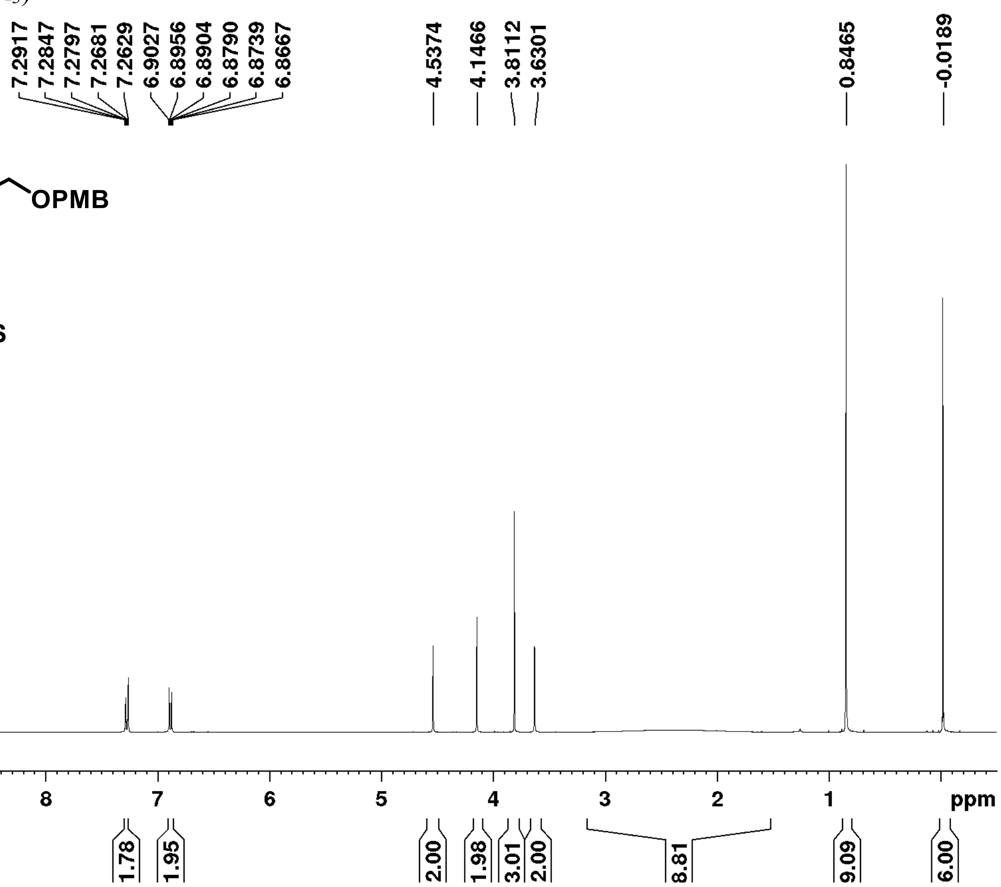

^{13}C NMR (100 MHz; $CDCl_3$)

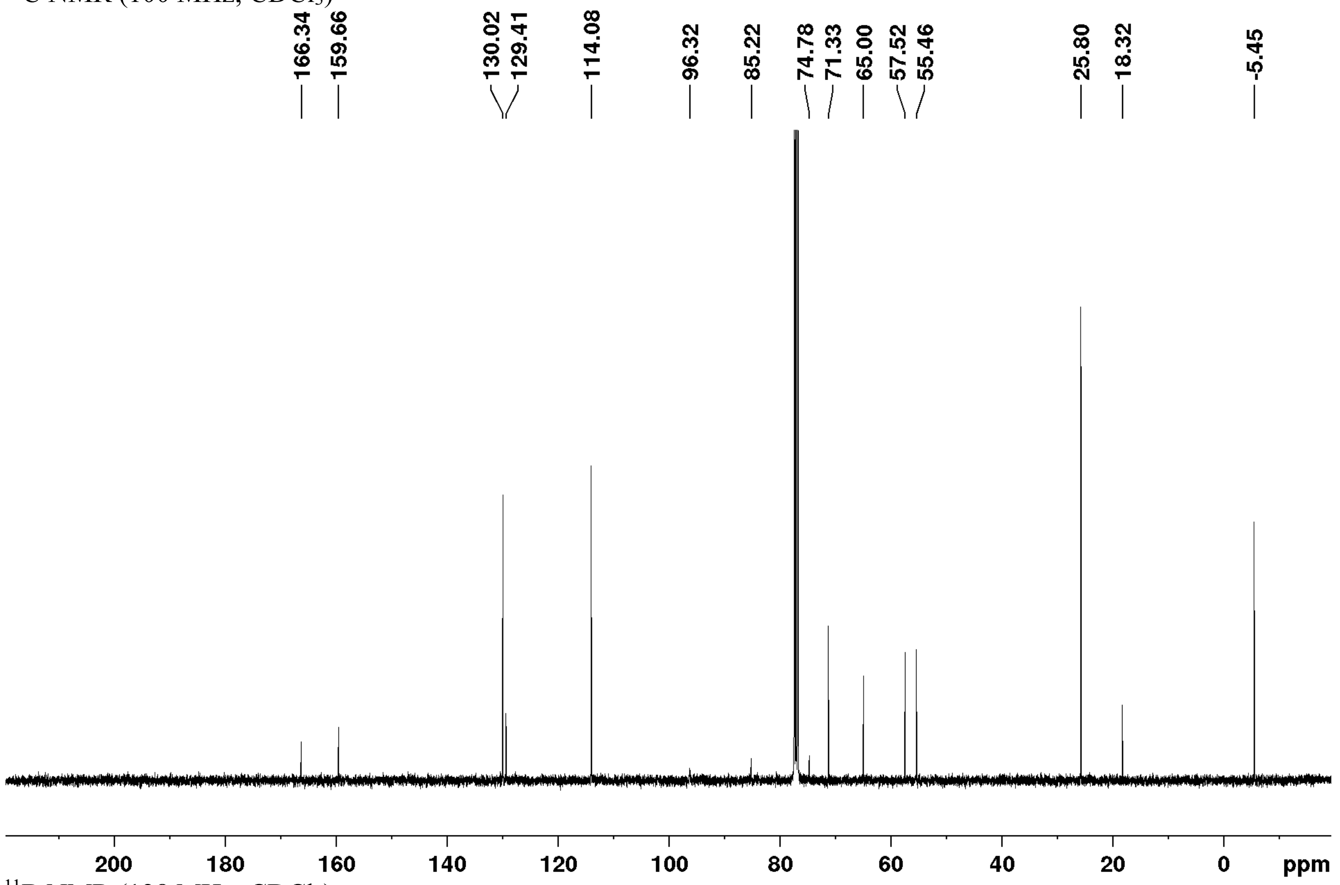

^{11}B NMR (128 MHz; $CDCl_3$)

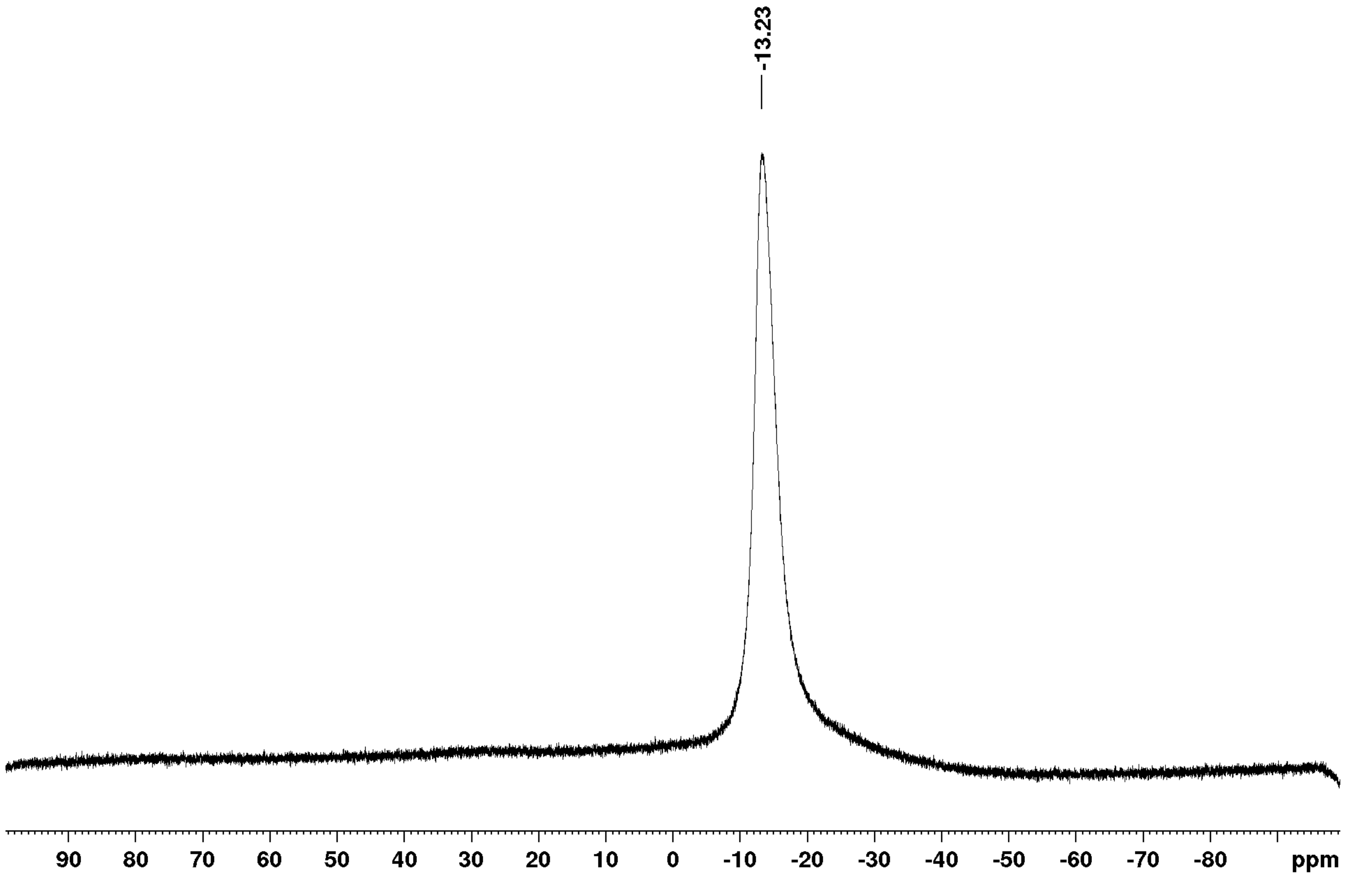

12-((*tert*-butyl)dimethylsilyl)hydroxymethyl)-2-(3-((4-methoxybenzyl)oxy)prop-1-yn-1-yl)-1,12-dicarba-*closo*-dodecaboranyl-1-carboxylic acid (28)

^{1}H NMR (400 MHz; $CDCl_3$)

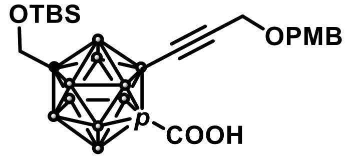

28

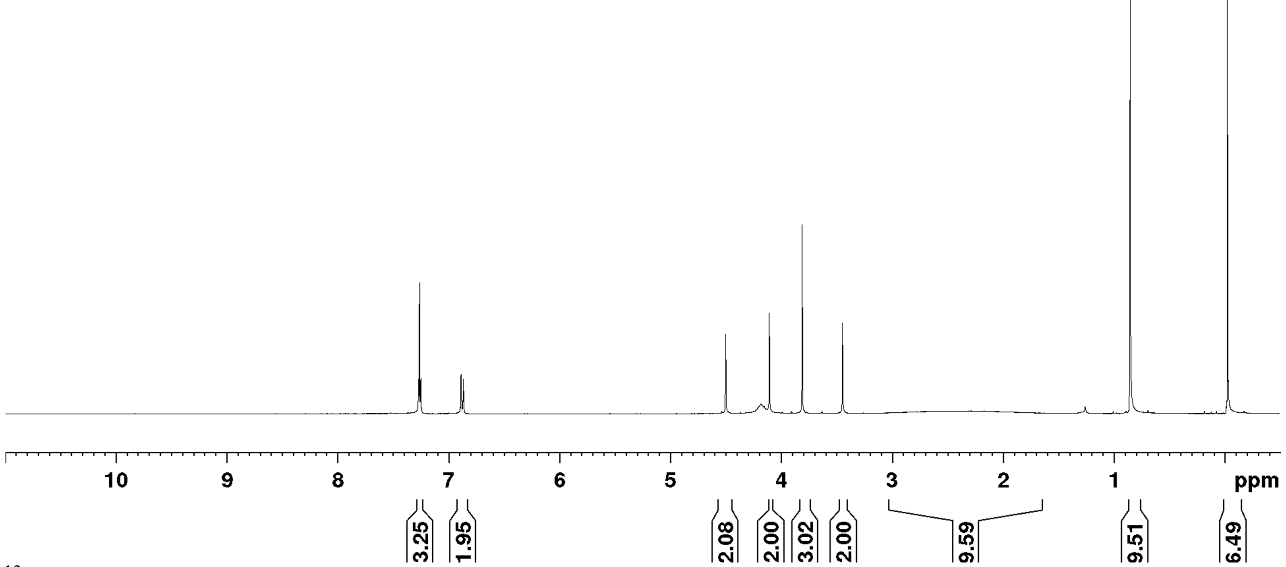

^{13}C NMR (100 MHz; $CDCl_3$)

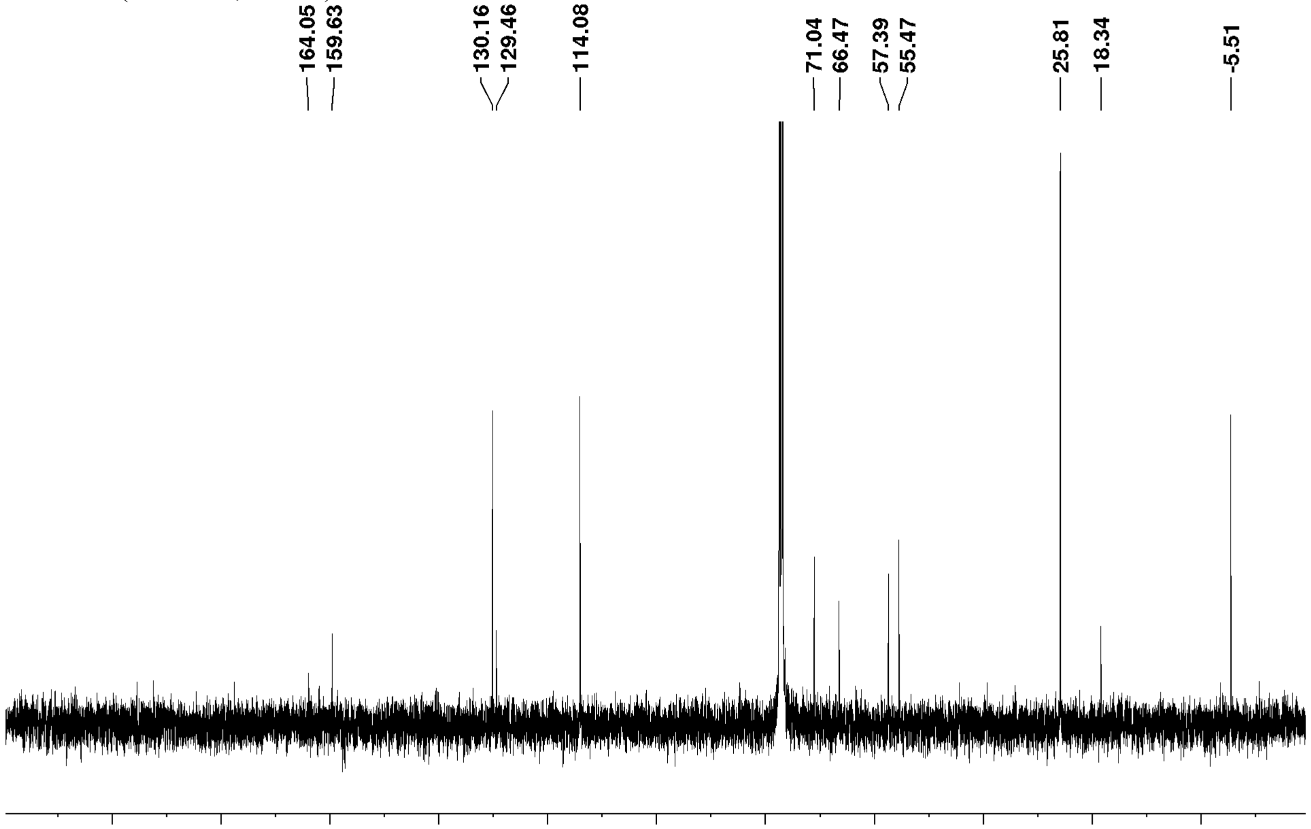

^{11}B NMR (128 MHz; CDCl$_3$)

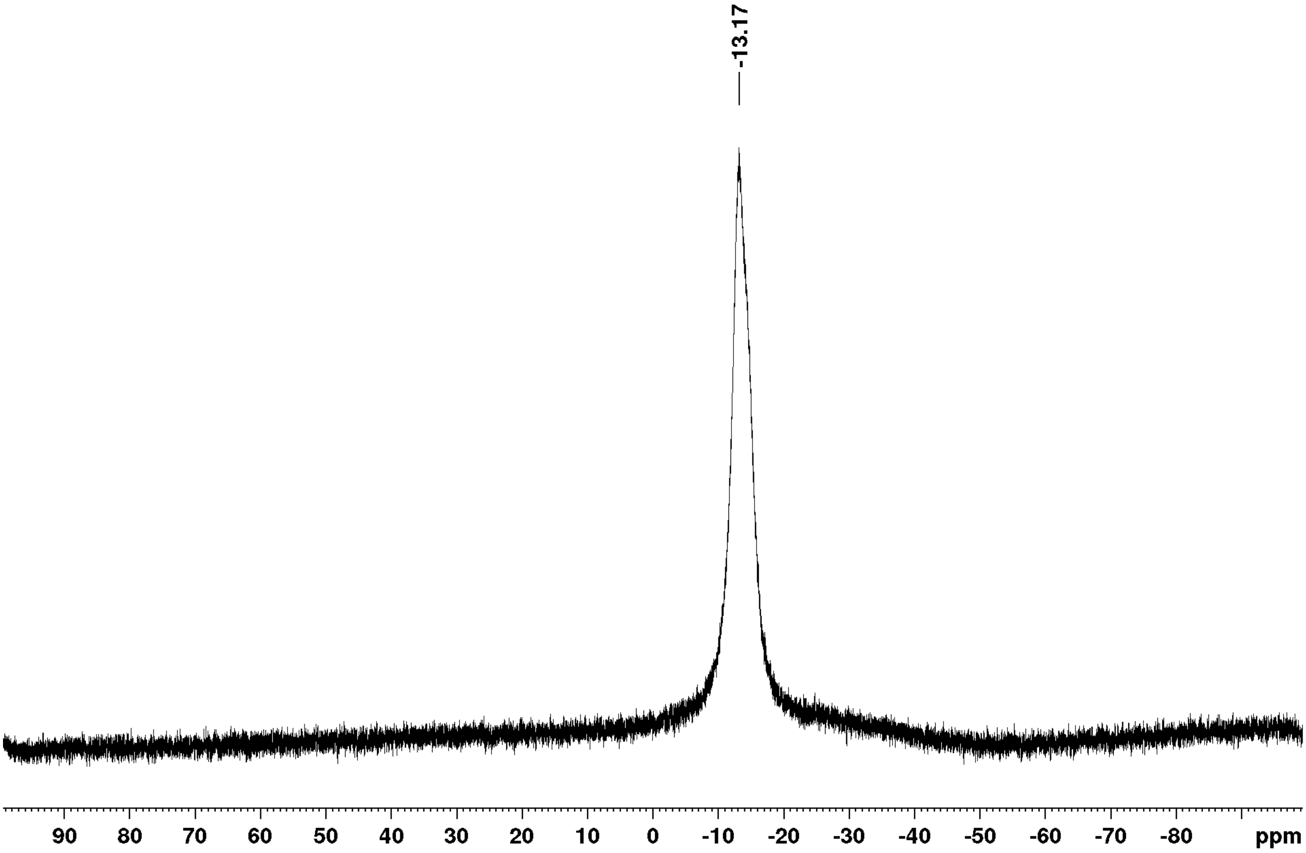

1-((*tert*-butyl)dimethylsilyl)hydroxymethyl)-2-(3-((4-methoxybenzyl)oxy)prop-1-yn-1-yl)-12-benzylaminocarboxyl-1,12-dicarba-*closo*-dodecaborane (29a)

^{1}H NMR (400 MHz; CDCl$_3$)

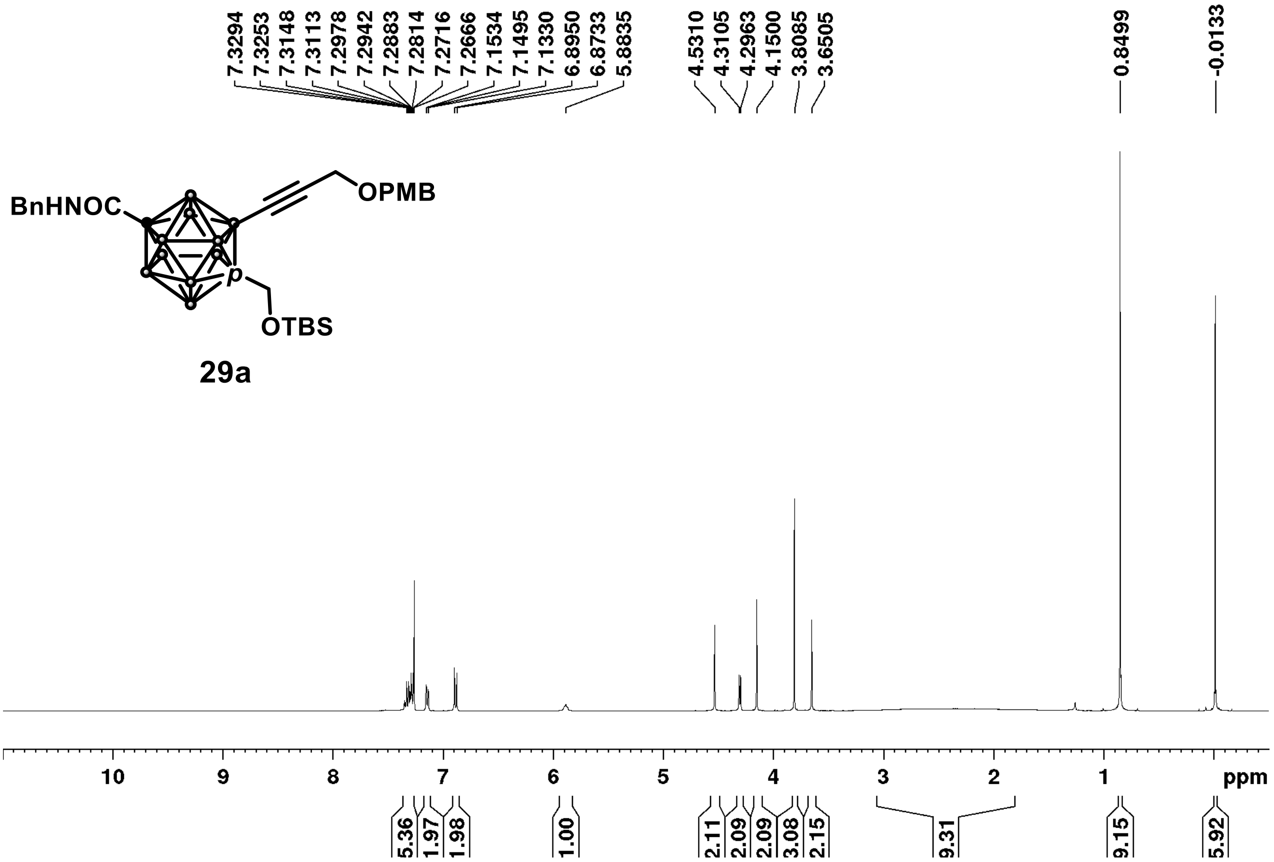

^{13}C NMR (100 MHz; $CDCl_3$)

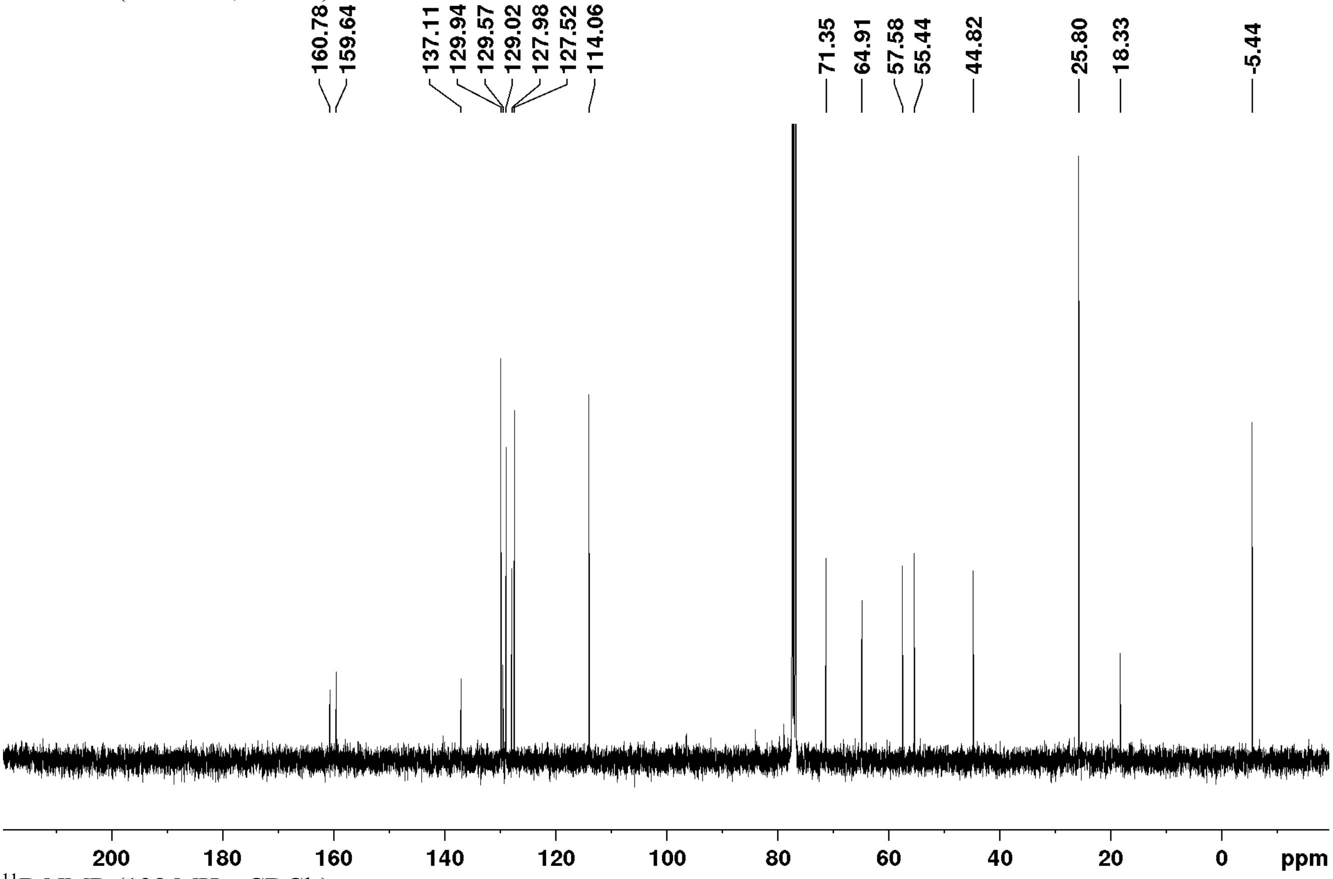

^{11}B NMR (128 MHz; $CDCl_3$)

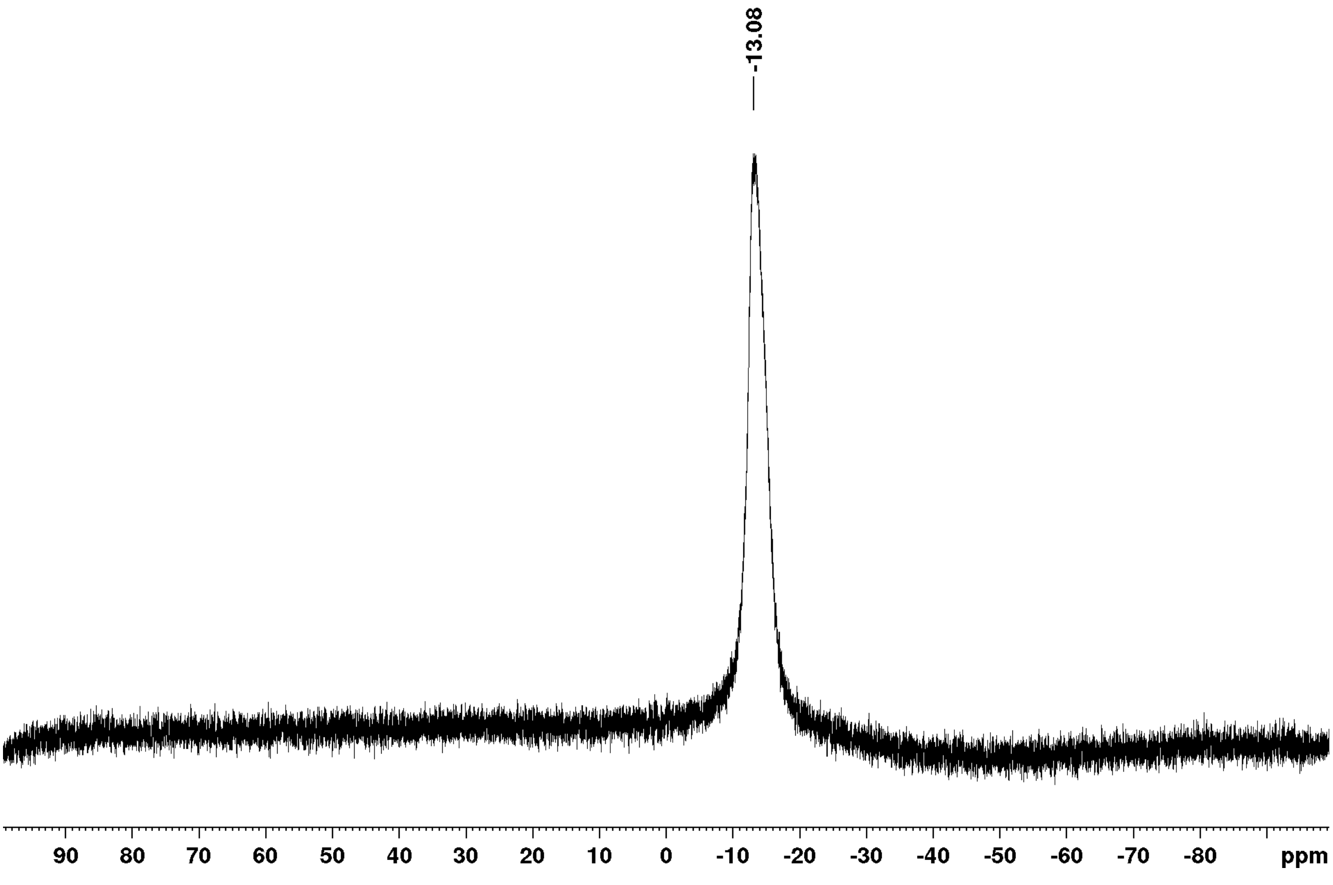

1-((*tert*-butyl)dimethylsilyl)hydroxymethyl)-2-(3-((4-methoxybenzyl)oxy)prop-1-yn-1-yl)-12-isobutylcarbamoyl-1,12-dicarba-*closo*-dodecaborane (29b)

^{1}H NMR (400 MHz; $CDCl_3$)

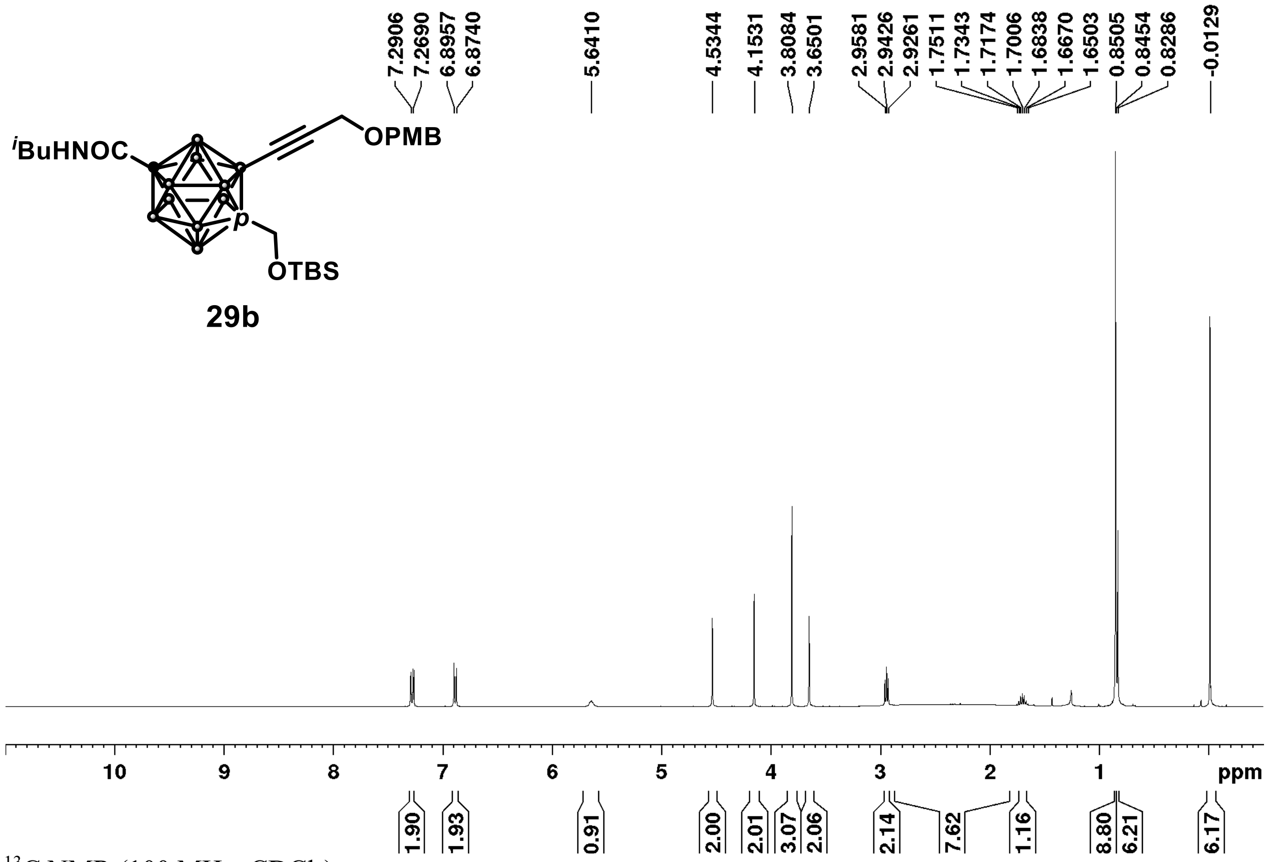

^{13}C NMR (100 MHz; $CDCl_3$)

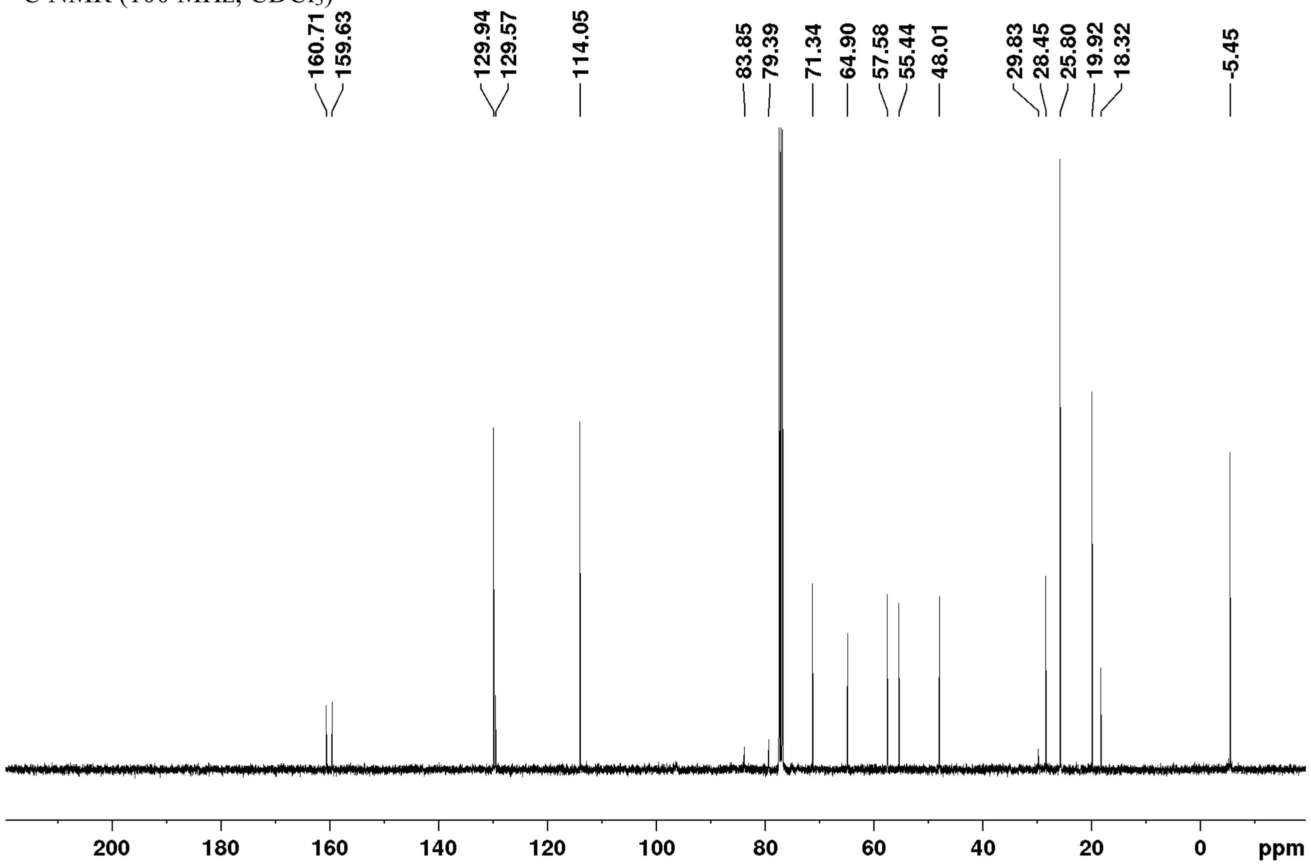

^{11}B NMR (128 MHz; $CDCl_3$)

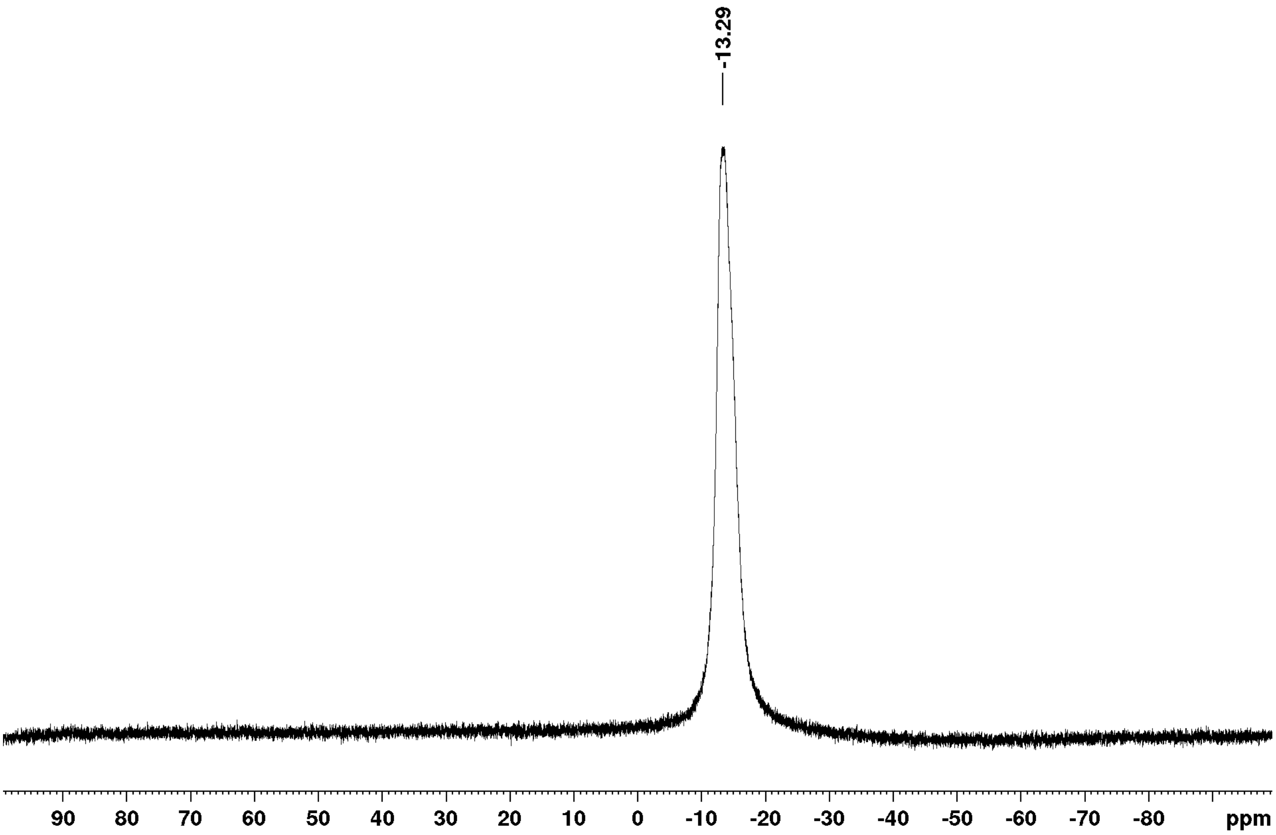

12-((*tert*-butyl)dimethylsilyl)hydroxymethyl)-2-(3-((4-methoxybenzyl)oxy)prop-1-yn-1-yl)-1-benzylaminocarboxyl-1,12-dicarba-*closo*-dodecaborane (33a)

^{1}H NMR (500 MHz; $CDCl_3$)

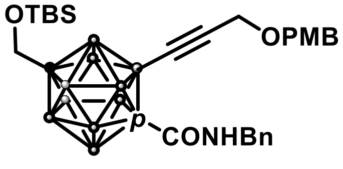

33a

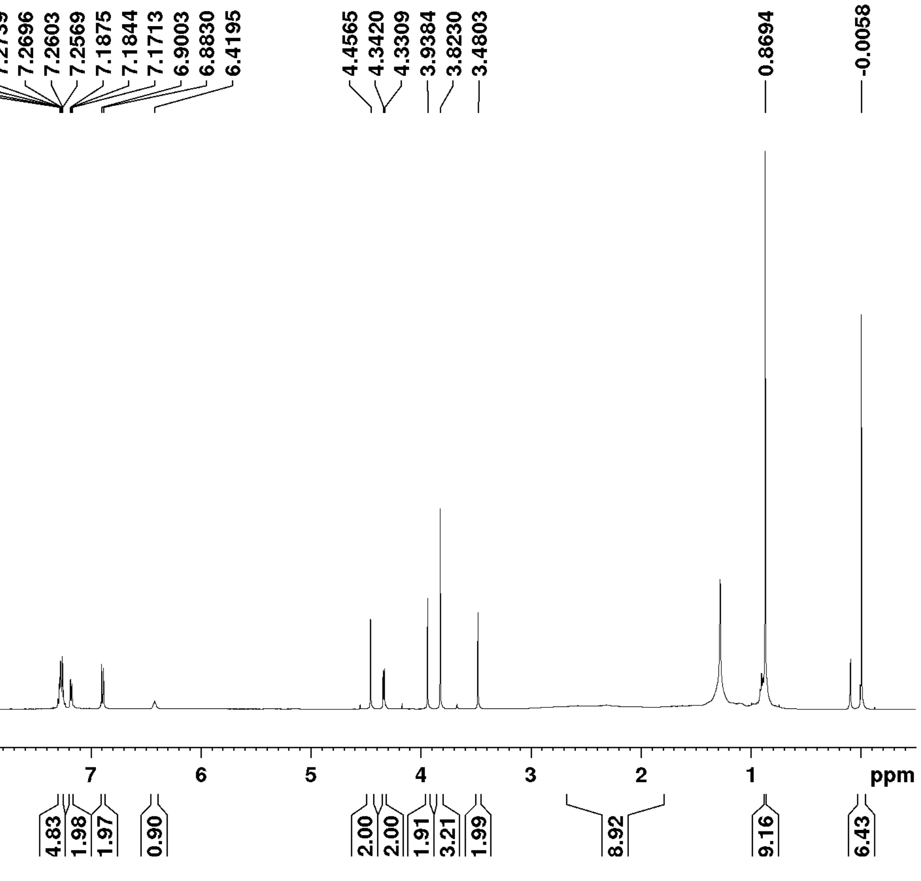

^{13}C NMR (125 MHz; $CDCl_3$)

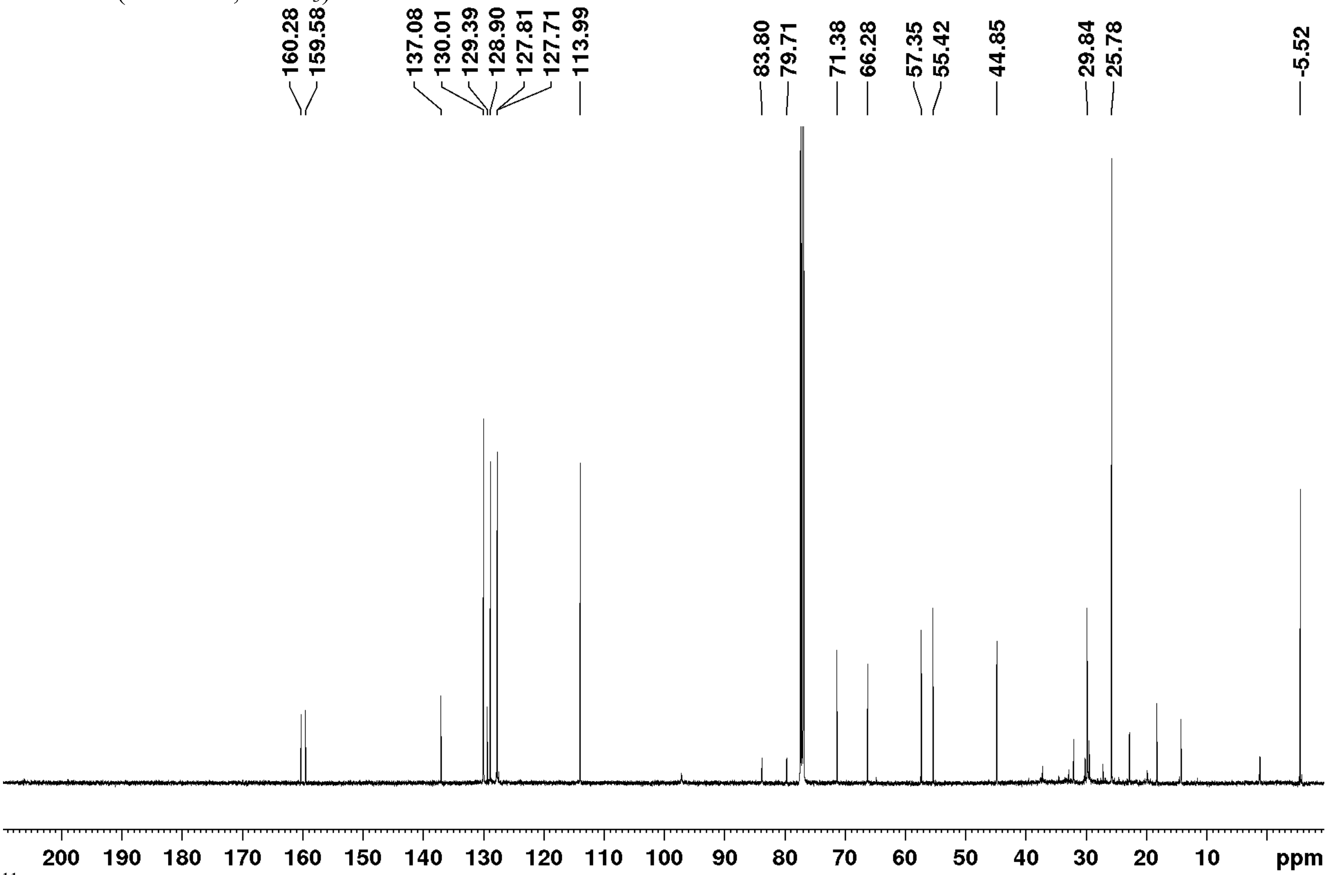

^{11}B NMR (160 MHz; $CDCl_3$)

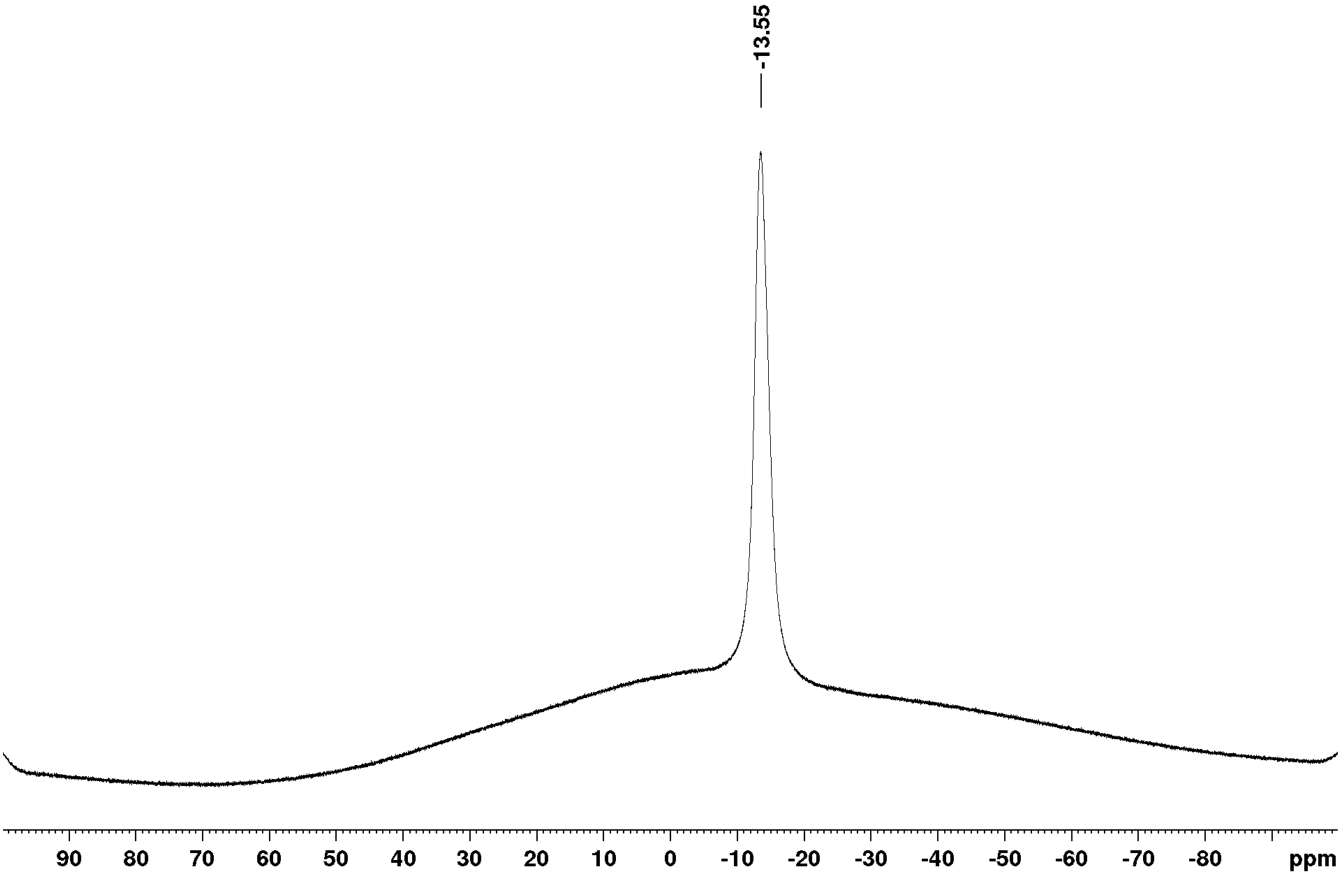

12-((*tert*-butyl)dimethylsilyl)hydroxymethyl)-2-(3-((4-methoxybenzyl)oxy)prop-1-yn-1-yl)-1-isobutylcarbamoyl-1,12-dicarba-*closo*-dodecaborane (33b)

^{1}H NMR (500 MHz; CDCl$_3$)

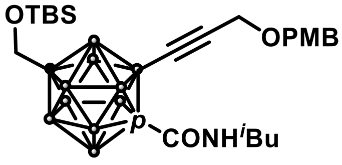

33b

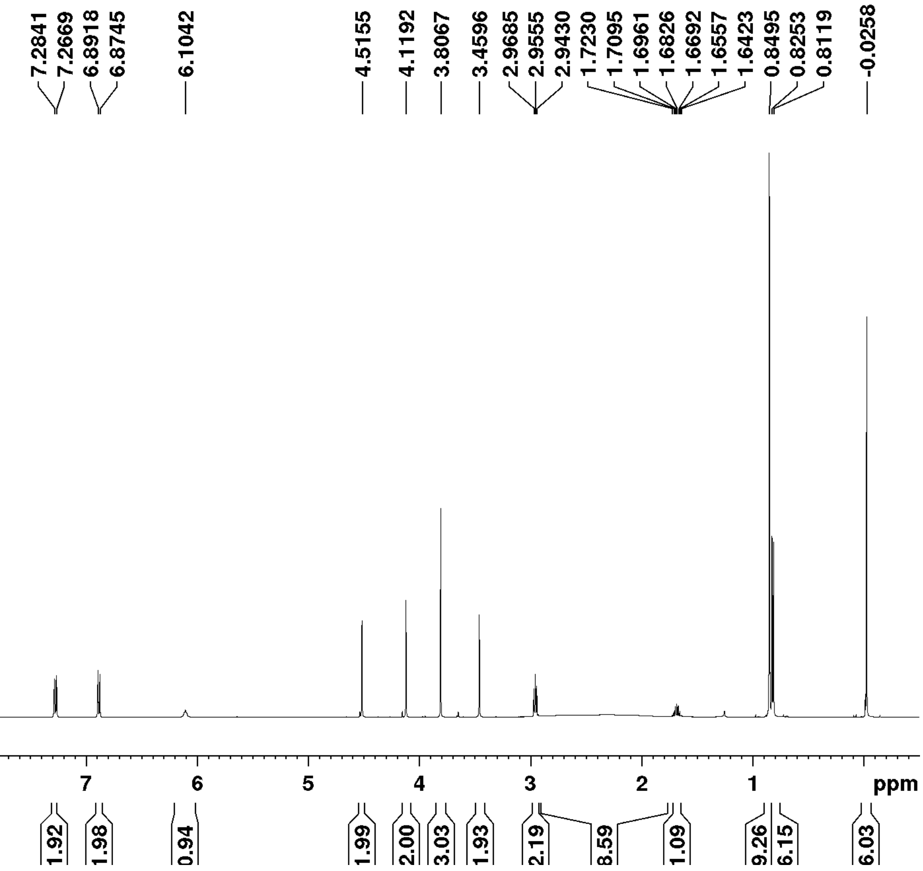

^{13}C NMR (125 MHz; CDCl$_3$)

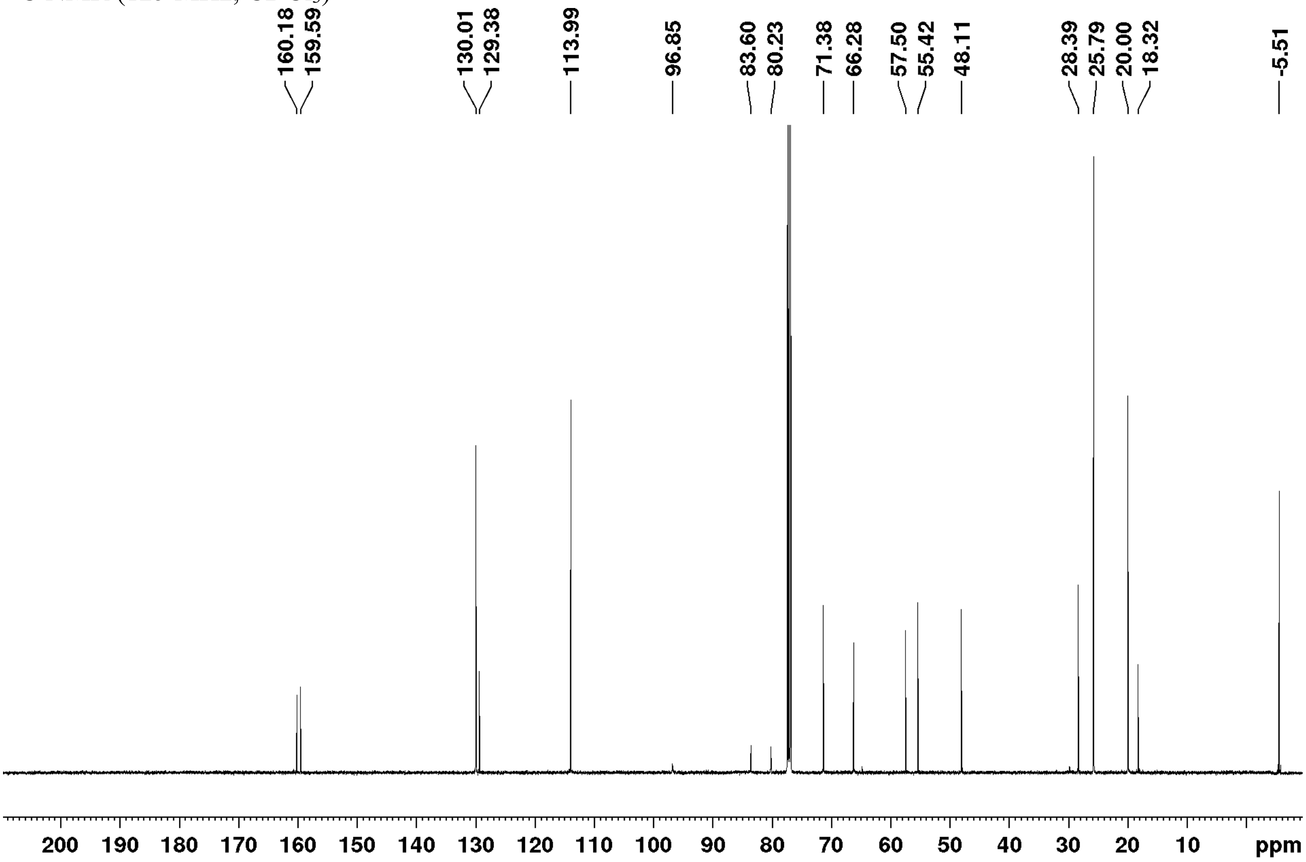

^{11}B NMR (160 MHz; $CDCl_3$)

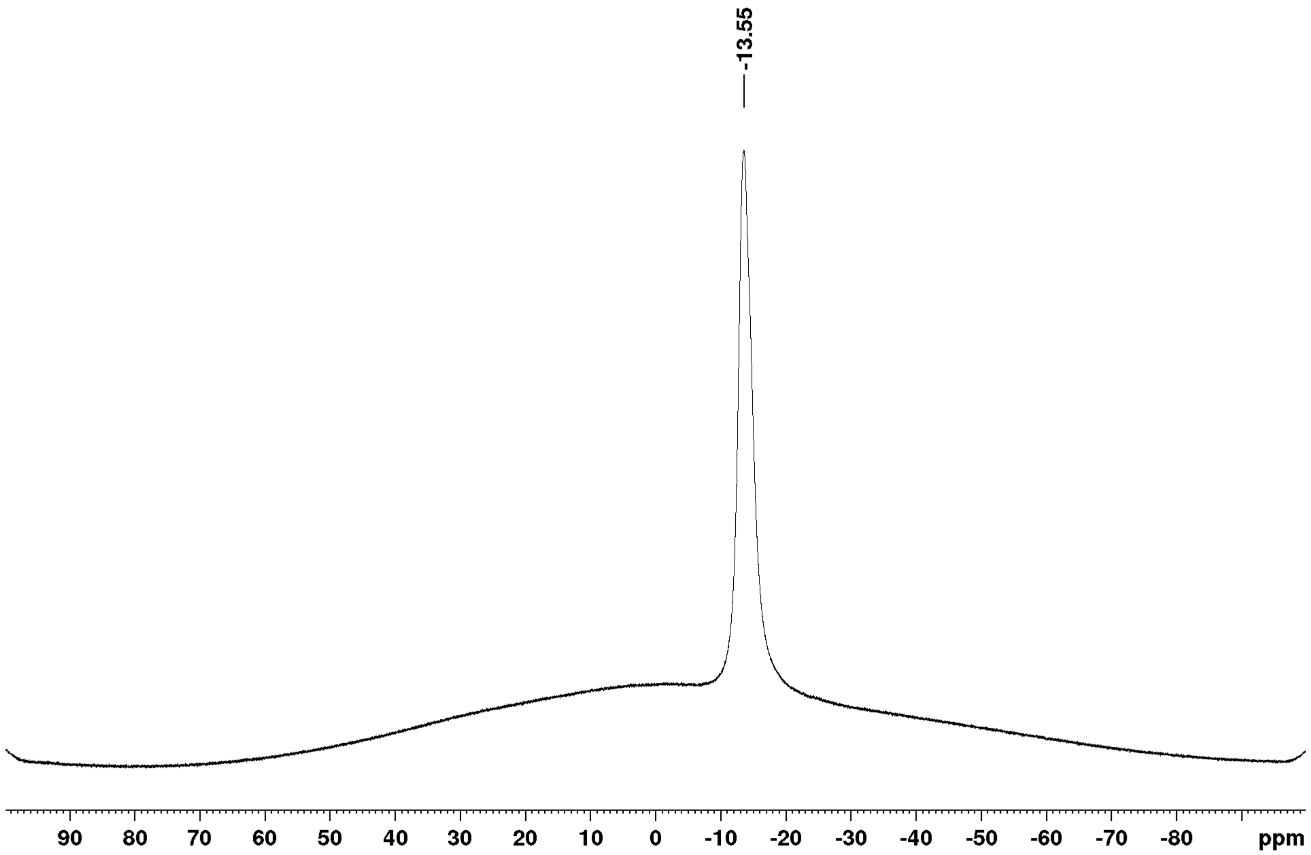

1-((*tert*-butyl)dimethylsilyl)hydroxymethyl)-2-(3-oxoprop-1-yn-1-yl)-12-benzylaminocarboxyl-1,12-dicarba-*closo*-dodecaborane (30a)

^{1}H NMR (500 MHz; $CDCl_3$)

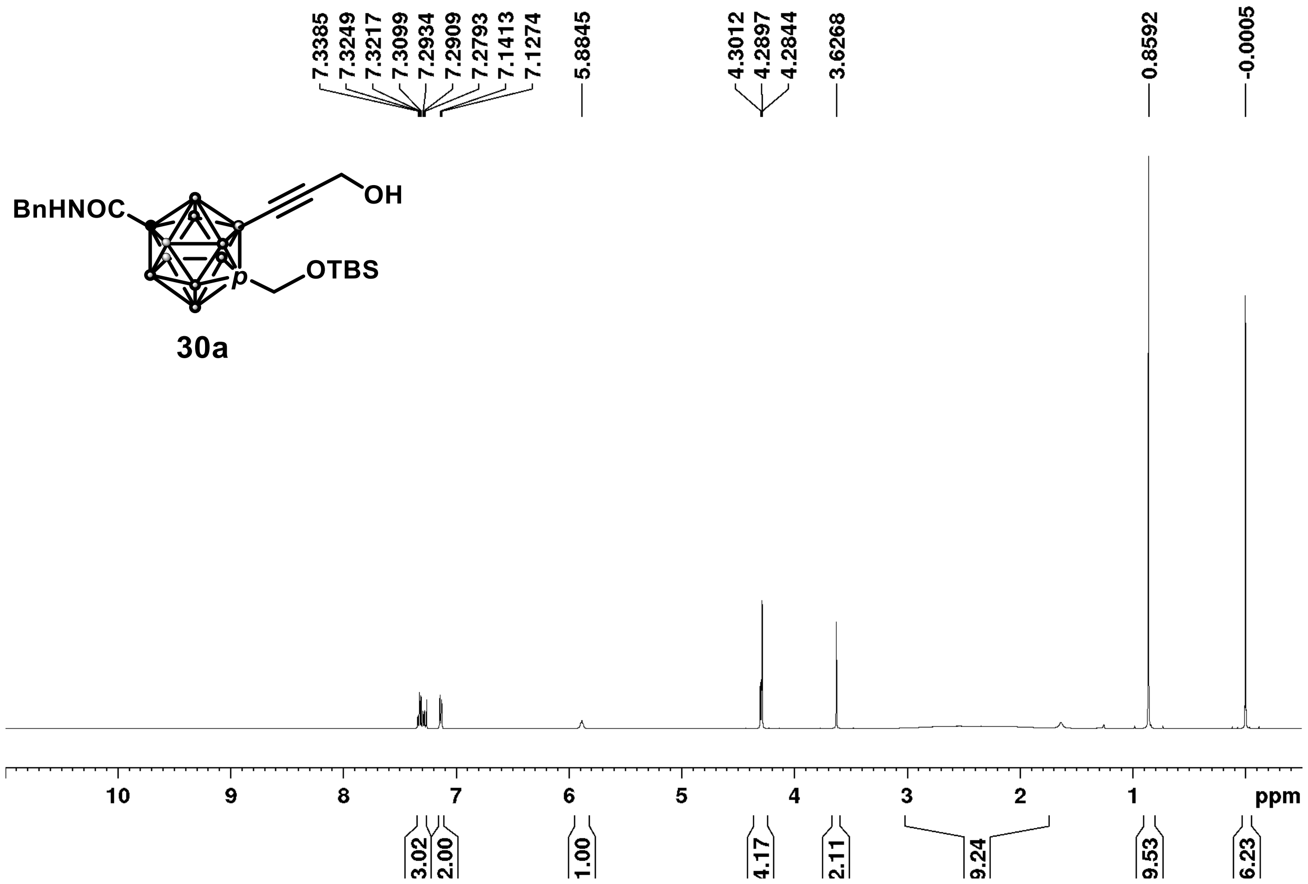

^{13}C NMR (125 MHz; $CDCl_3$)

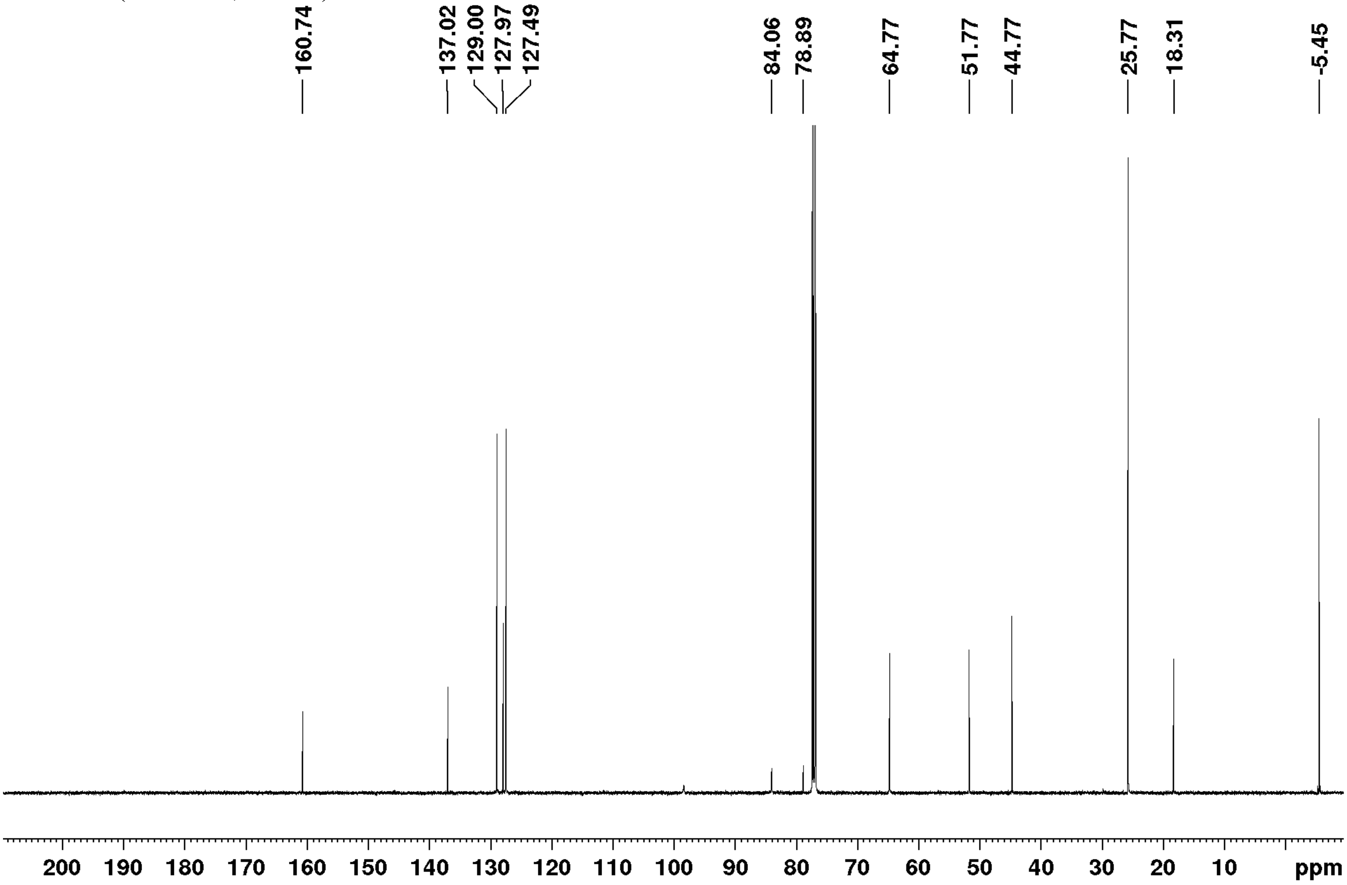

^{11}B NMR (160 MHz; $CDCl_3$)

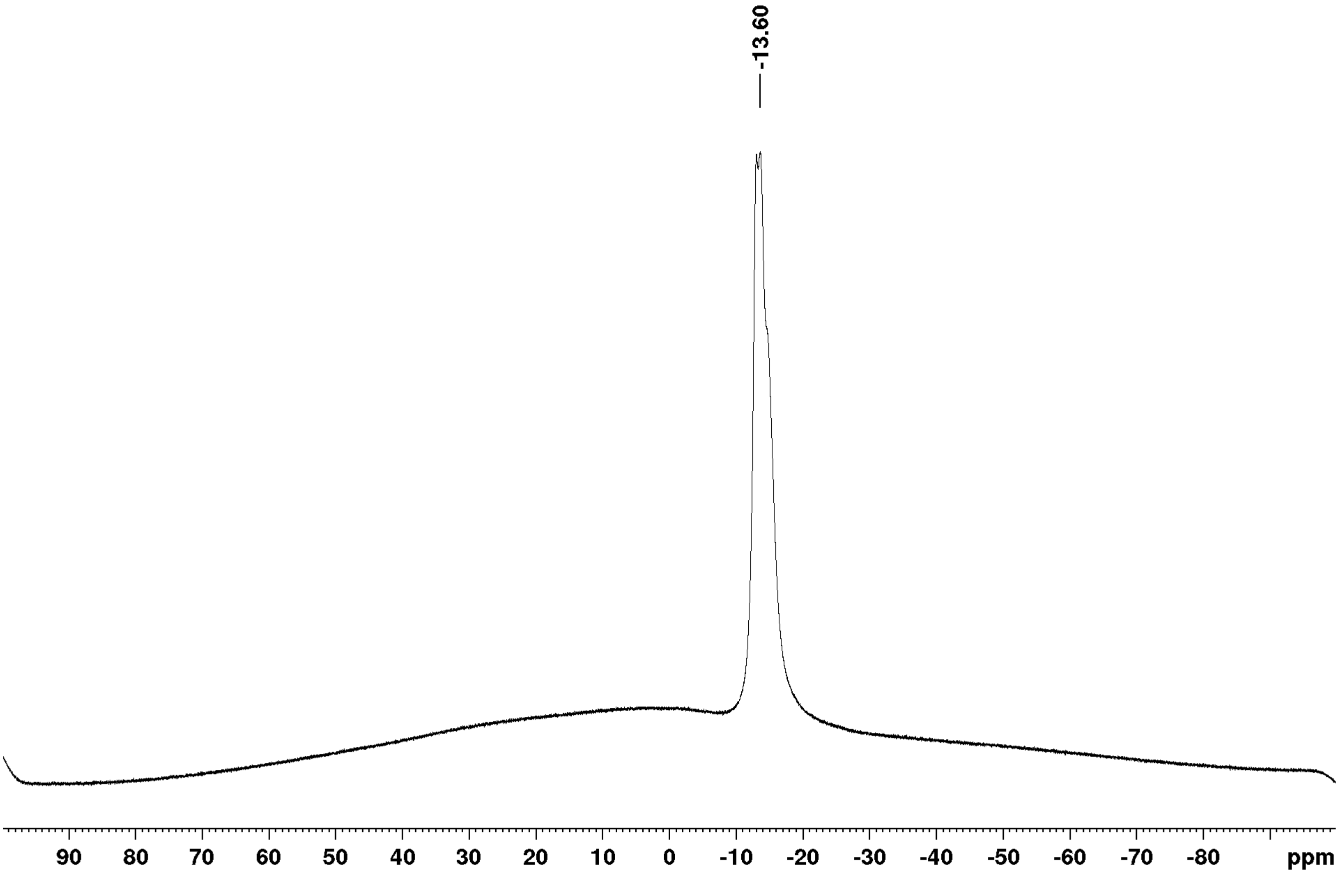

1-((*tert*-butyl)dimethylsilyl)hydroxymethyl)-2-(3-oxoprop-1-yn-1-yl)-12-isobutylcarbamoyl-1,12-dicarba-*closo*-dodecaborane (30b)

^{1}H NMR (500 MHz; $CDCl_3$)

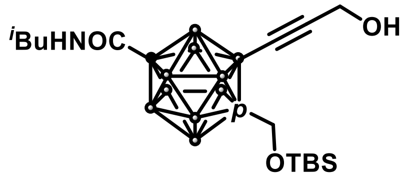

30b

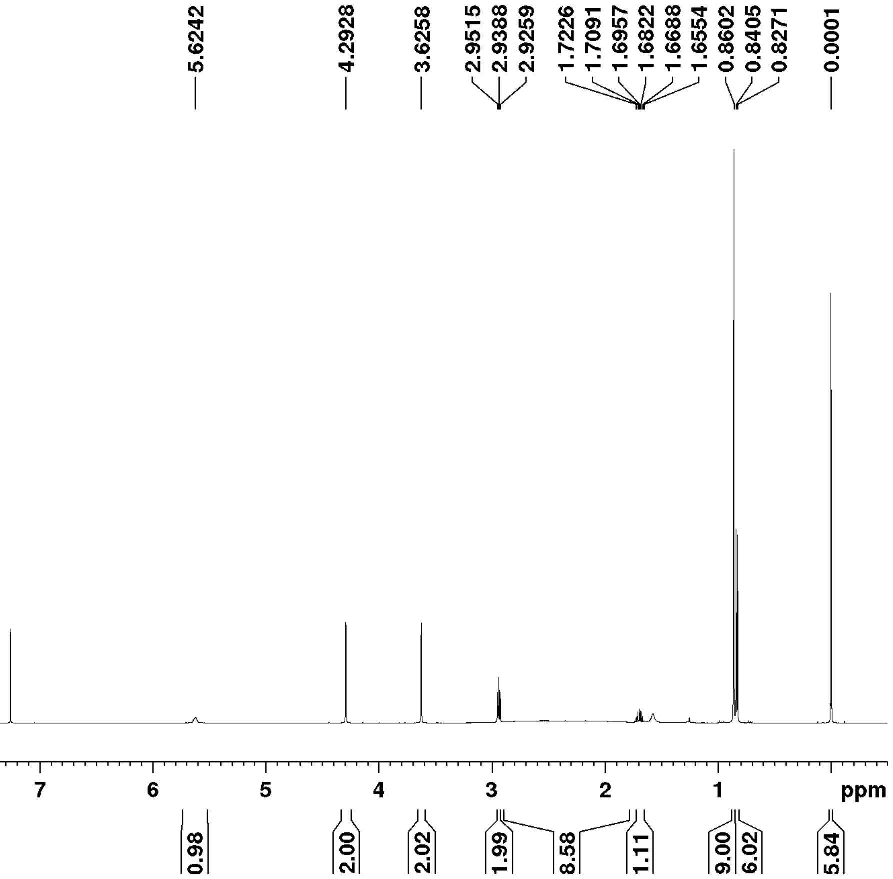

^{13}C NMR (125 MHz; $CDCl_3$)

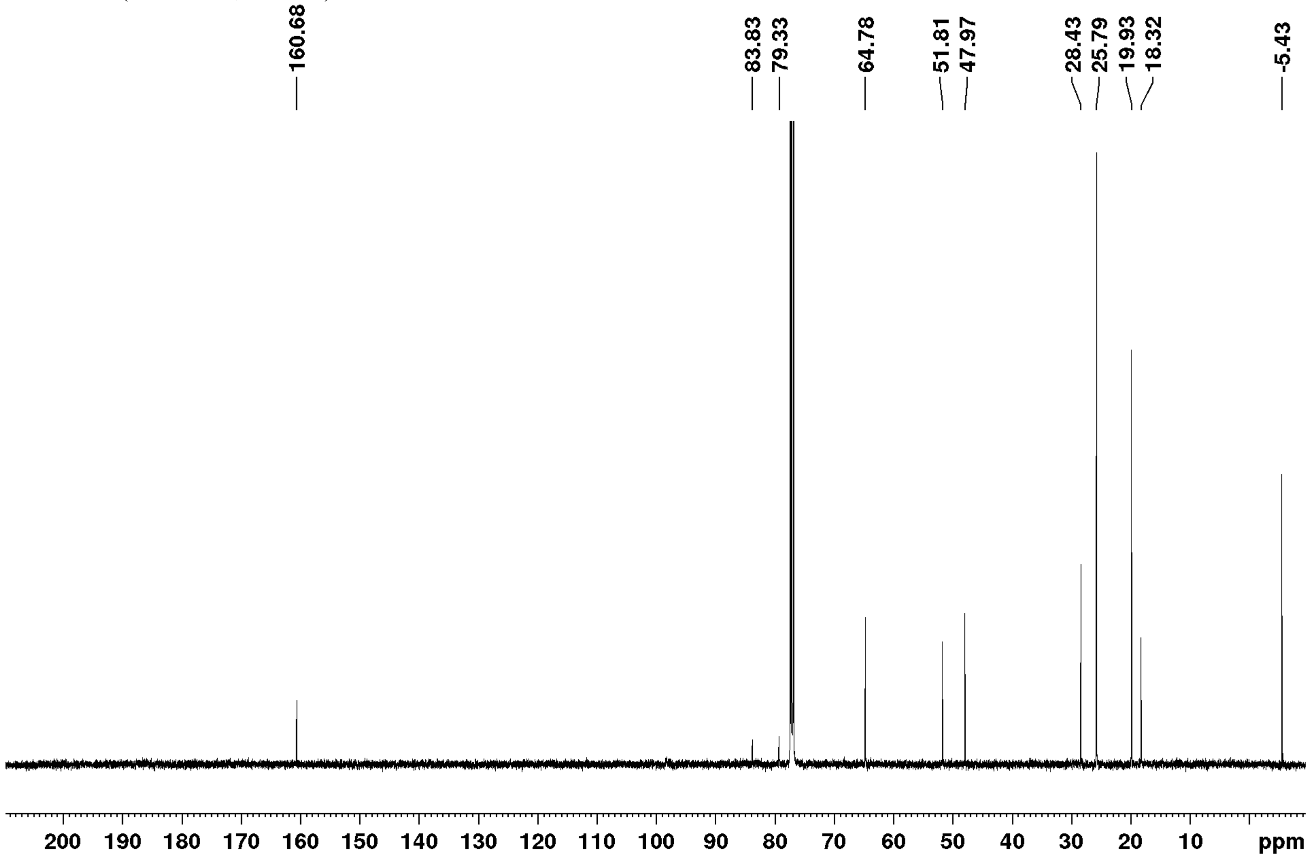

^{11}B NMR (160 MHz; $CDCl_3$)

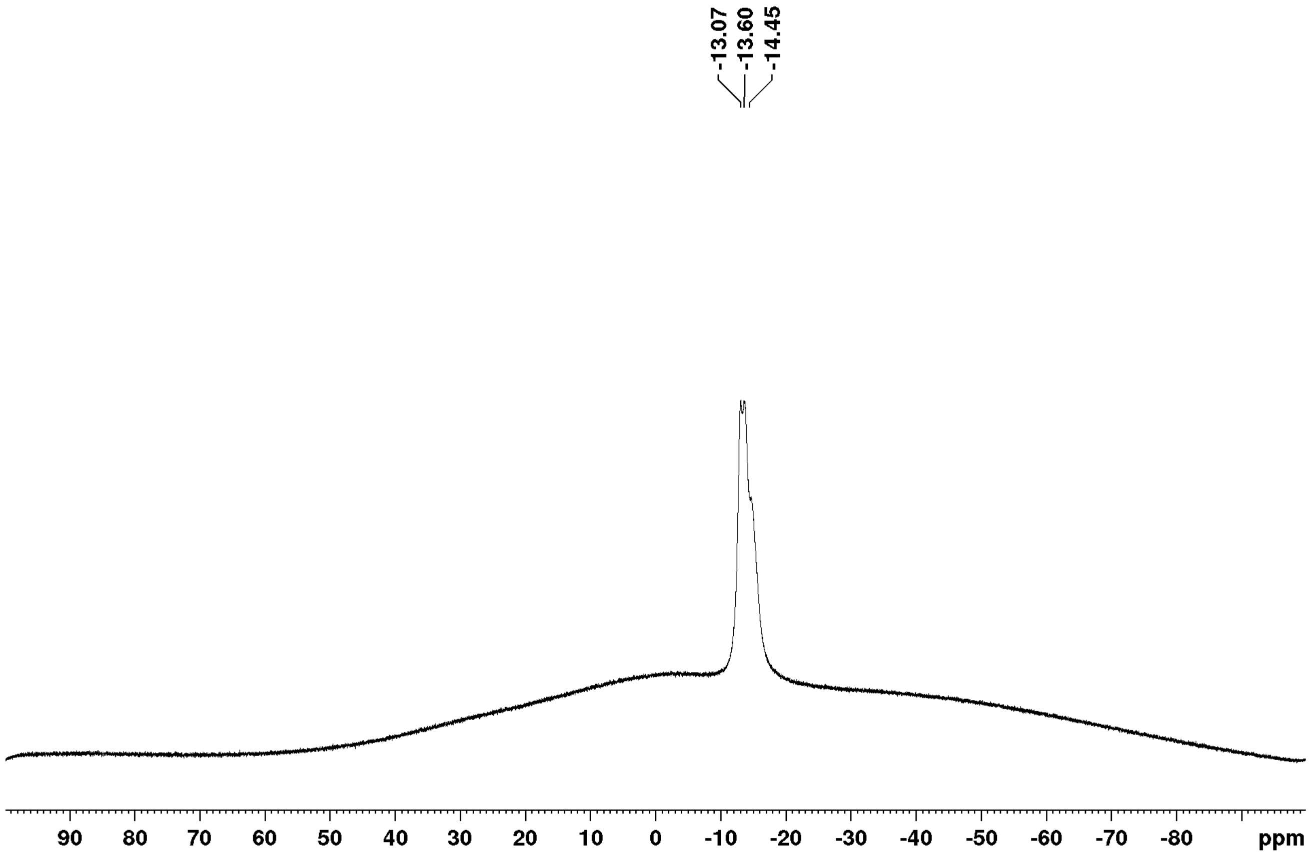

12-((*tert*-butyl)dimethylsilyl)hydroxymethyl)-2-(3-oxoprop-1-yn-1-yl)-1-benzylaminocarboxyl-1,12-dicarba-*closo*-dodecaborane (34a)

^{1}H NMR (500 MHz; $CDCl_3$)

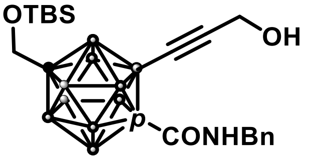

34a

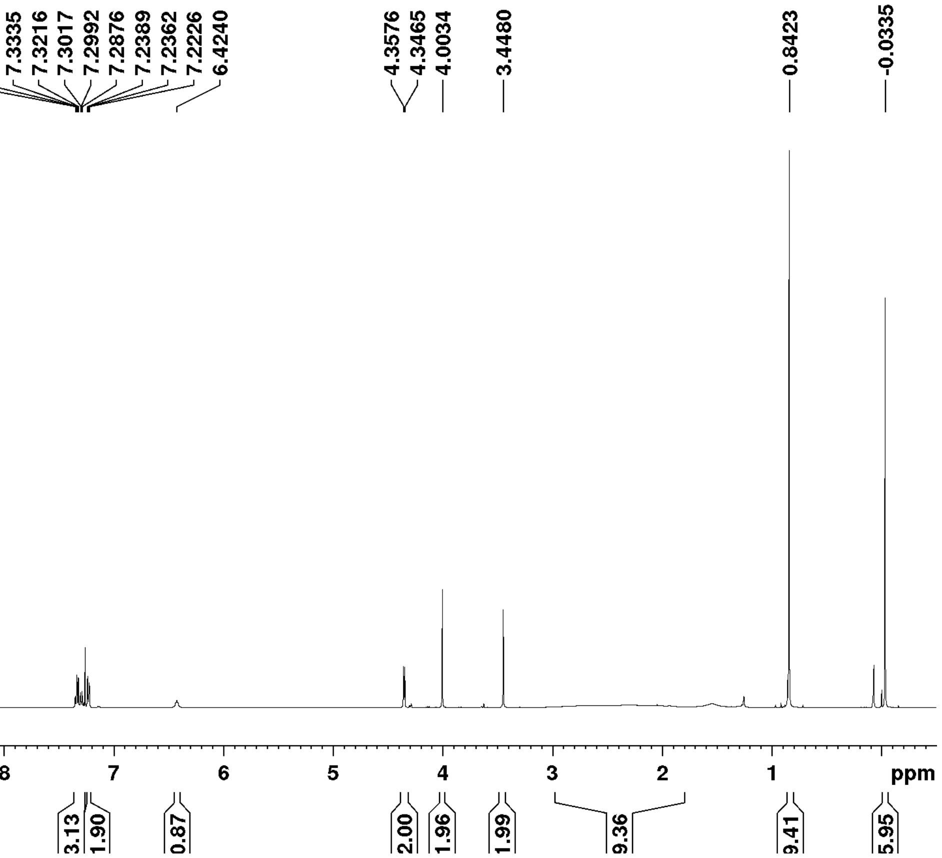

^{13}C NMR (125 MHz; $CDCl_3$)

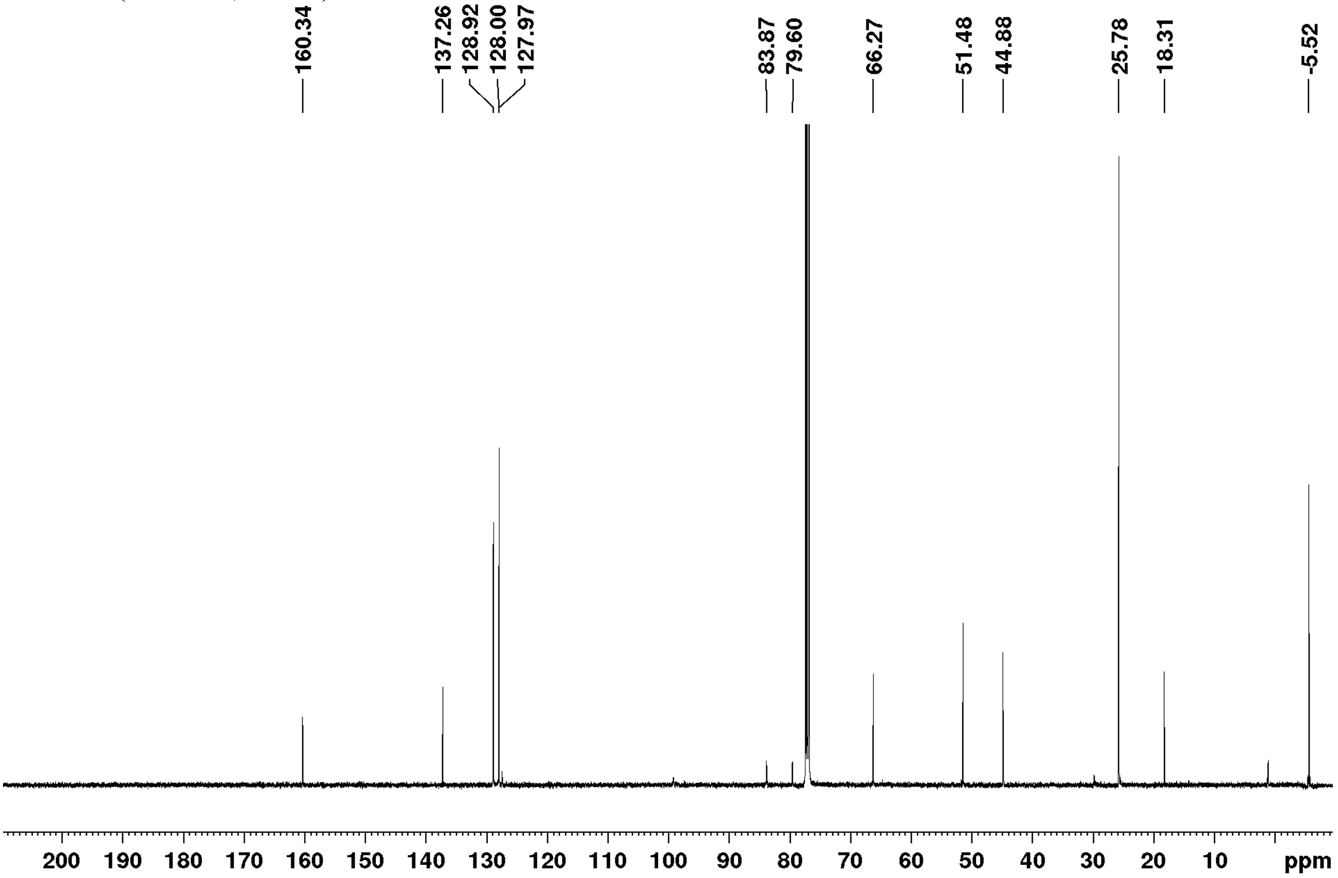

^{11}B NMR (160 MHz; $CDCl_3$)

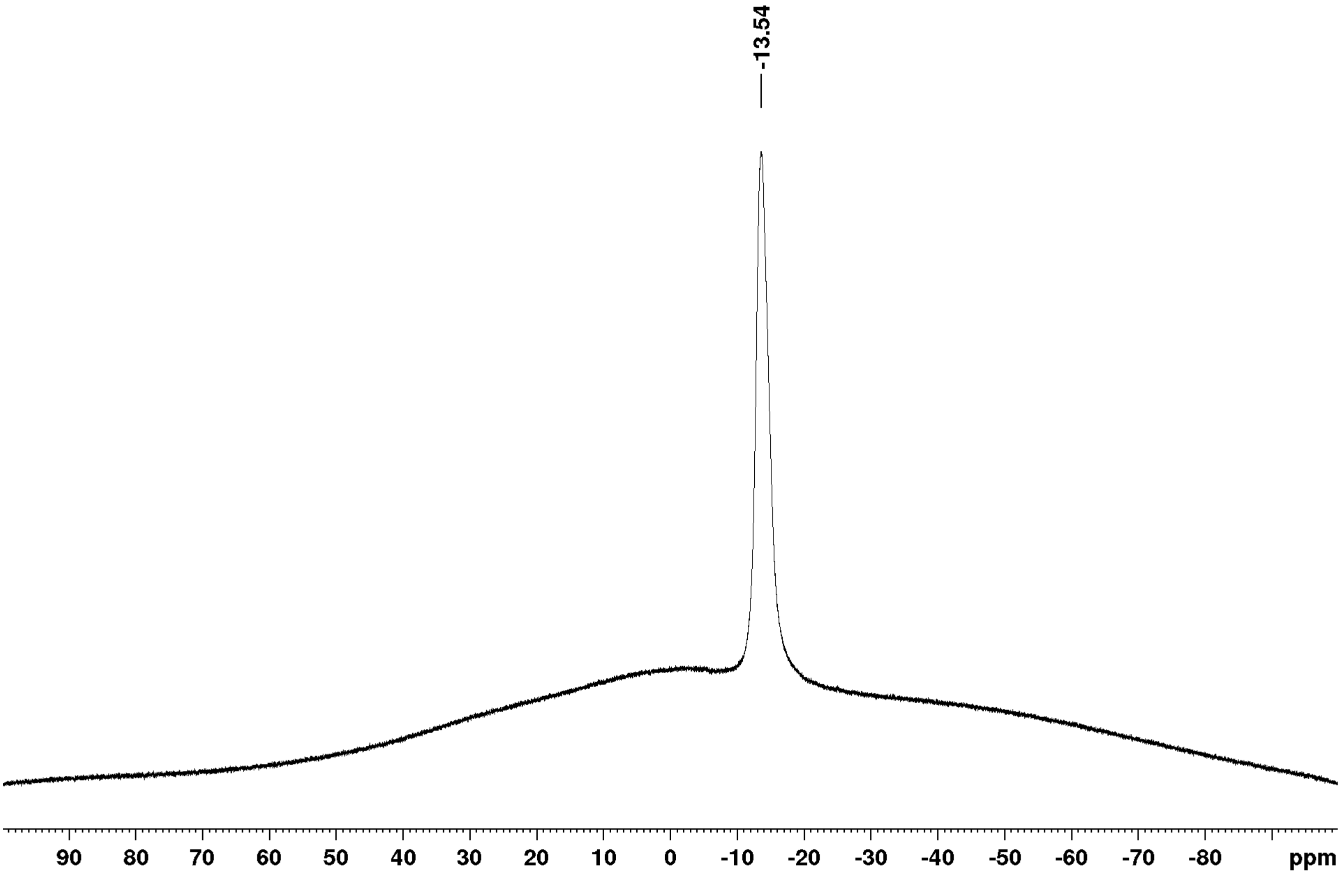

12-((*tert*-butyl)dimethylsilyl)hydroxymethyl)-2-(3-oxoprop-1-yn-1-yl)-1-isobutylcarbamoyl-1,12-dicarba-*closo*-dodecaborane (34b)

^{1}H NMR (500 MHz; $CDCl_3$)

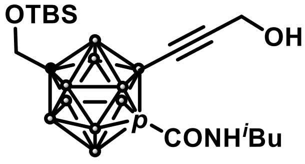

34b

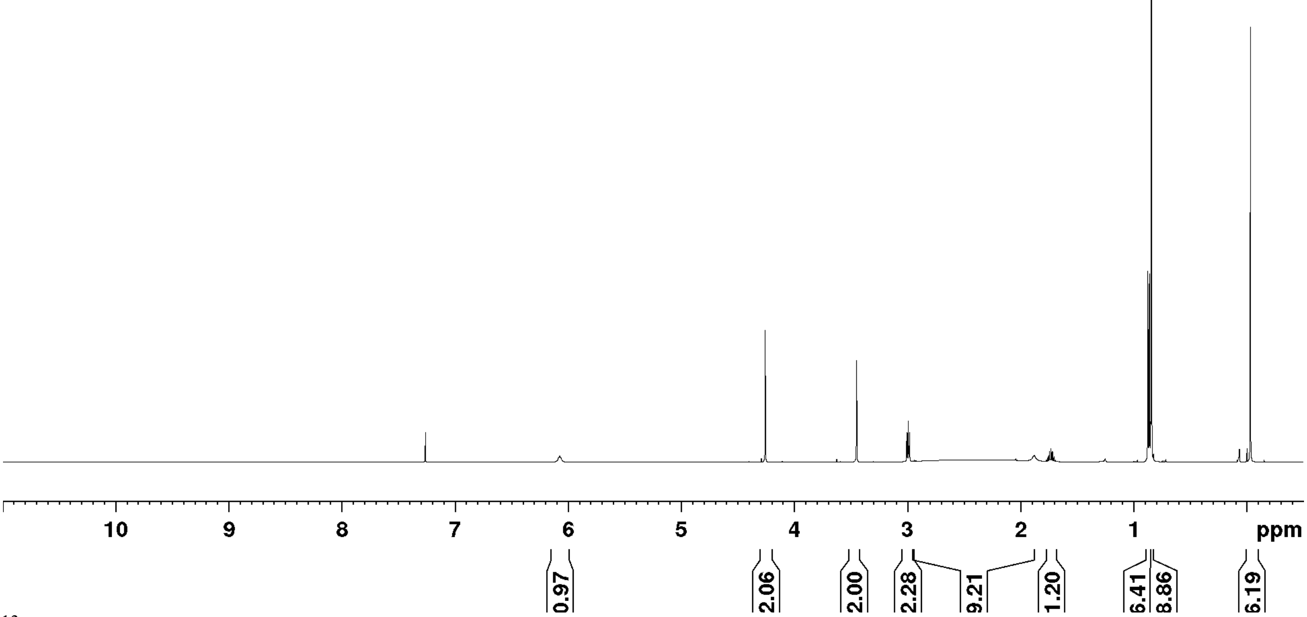

^{13}C NMR (125 MHz; $CDCl_3$)

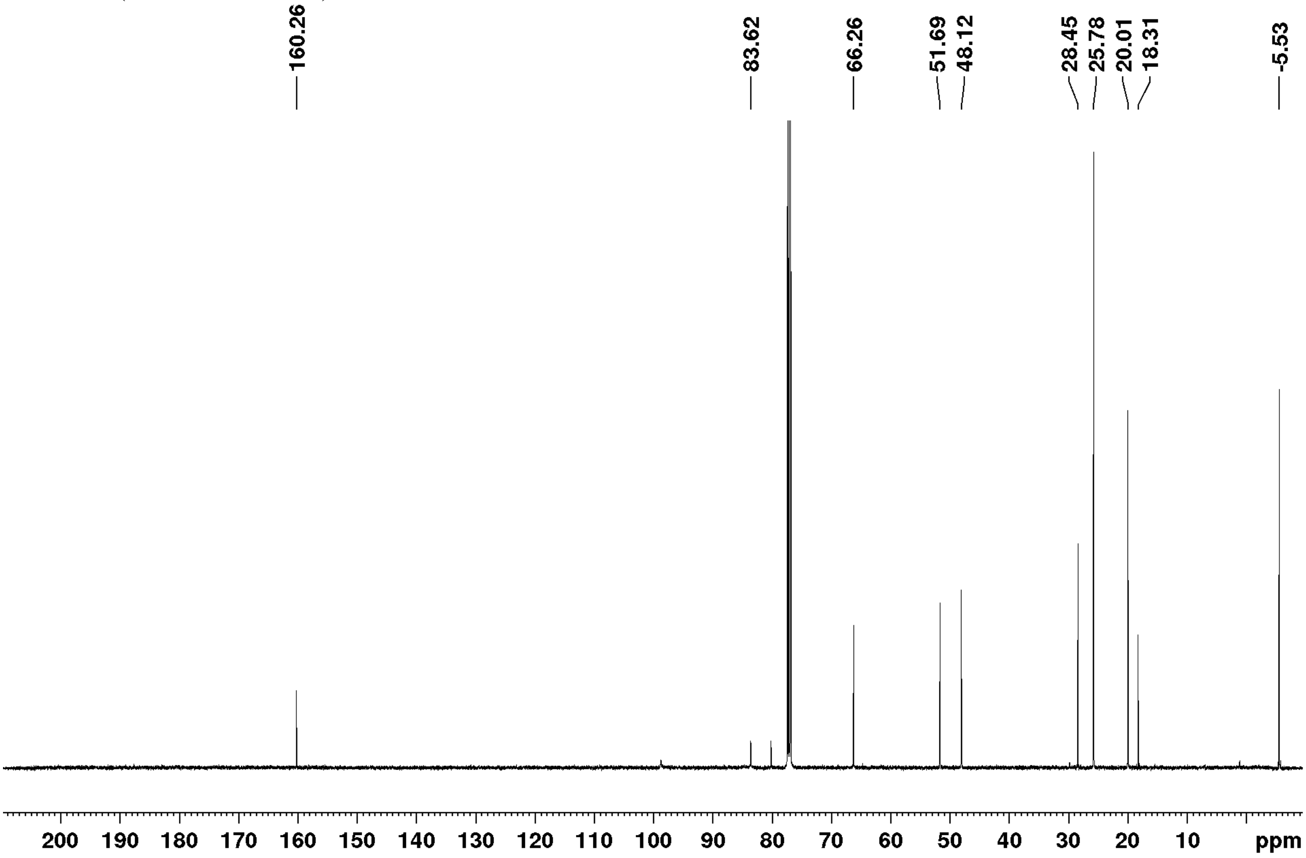

^{11}B NMR (160 MHz; $CDCl_3$)

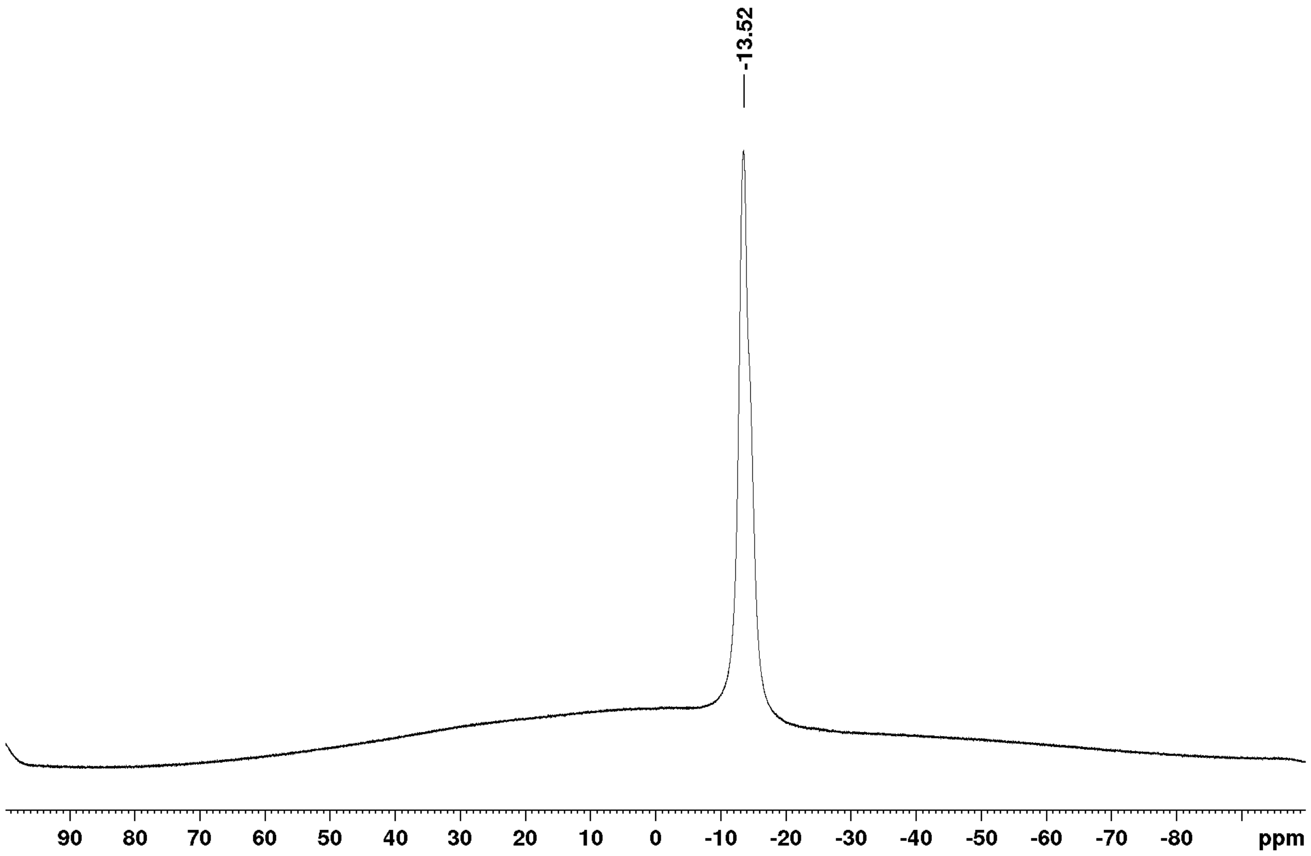

1-((*tert*-butyl)dimethylsilyl)hydroxymethyl)-2-(3-oxoprop-1-yn-1-yl)-12-benzylaminocarboxyl-1,12-dicarba-*closo*-dodecaborane (31a)

^{1}H NMR (500 MHz; $CDCl_3$)

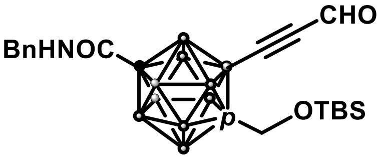

31a

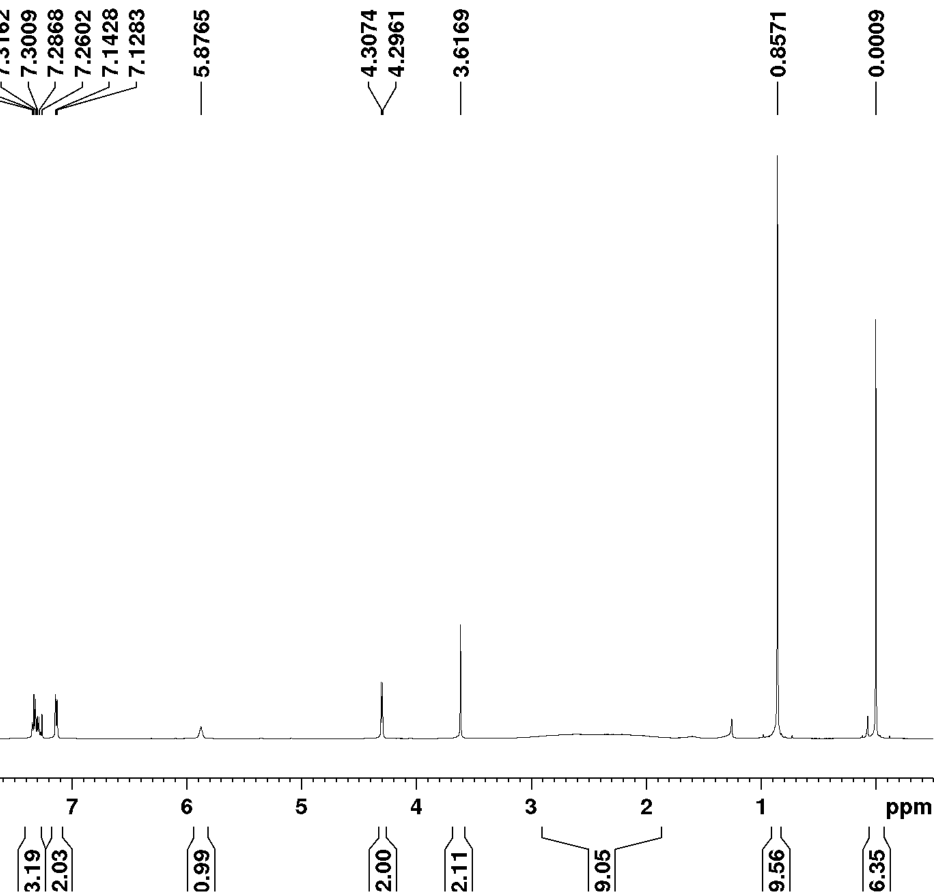

^{13}C NMR (125 MHz; $CDCl_3$)

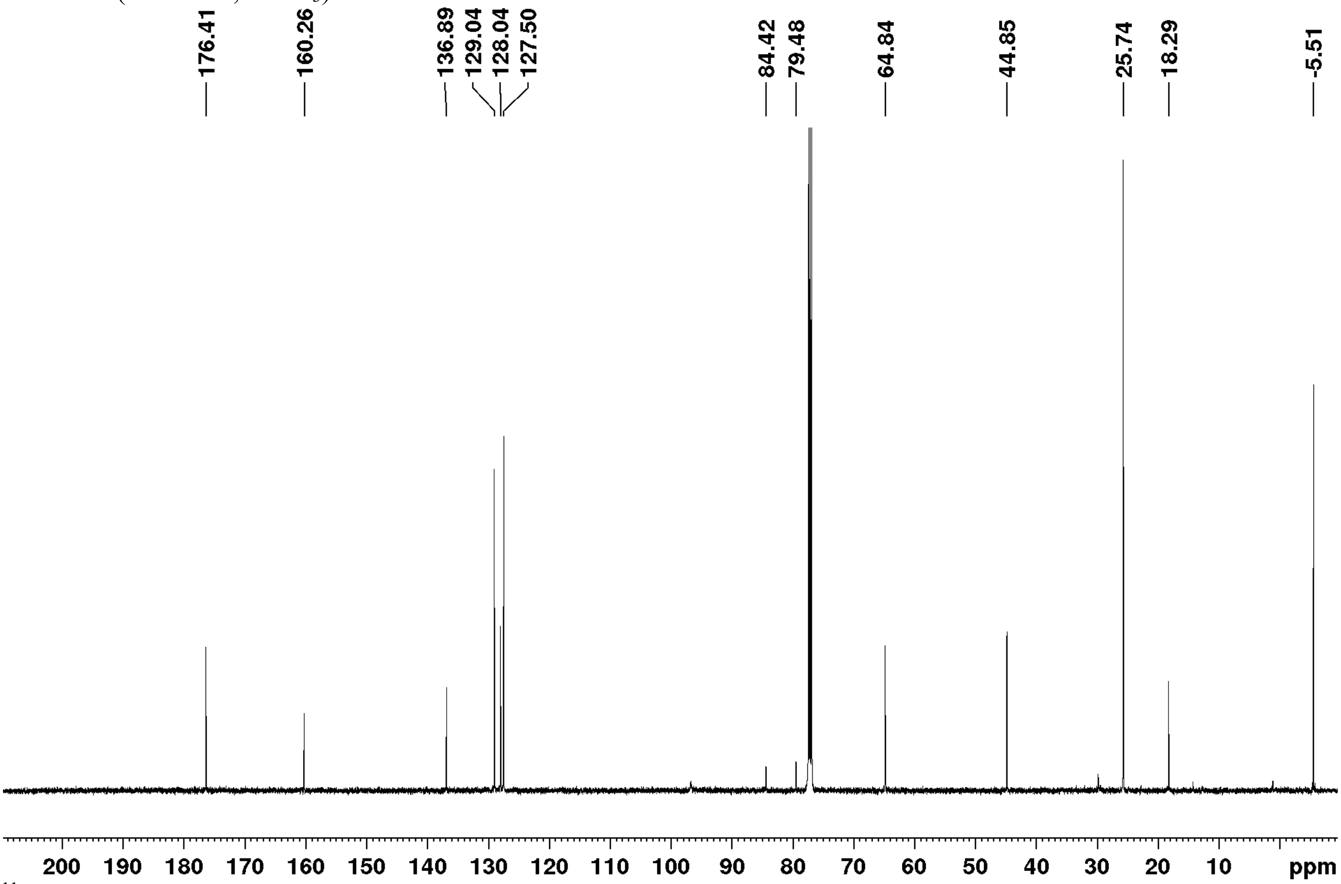

^{11}B NMR (160 MHz; $CDCl_3$)

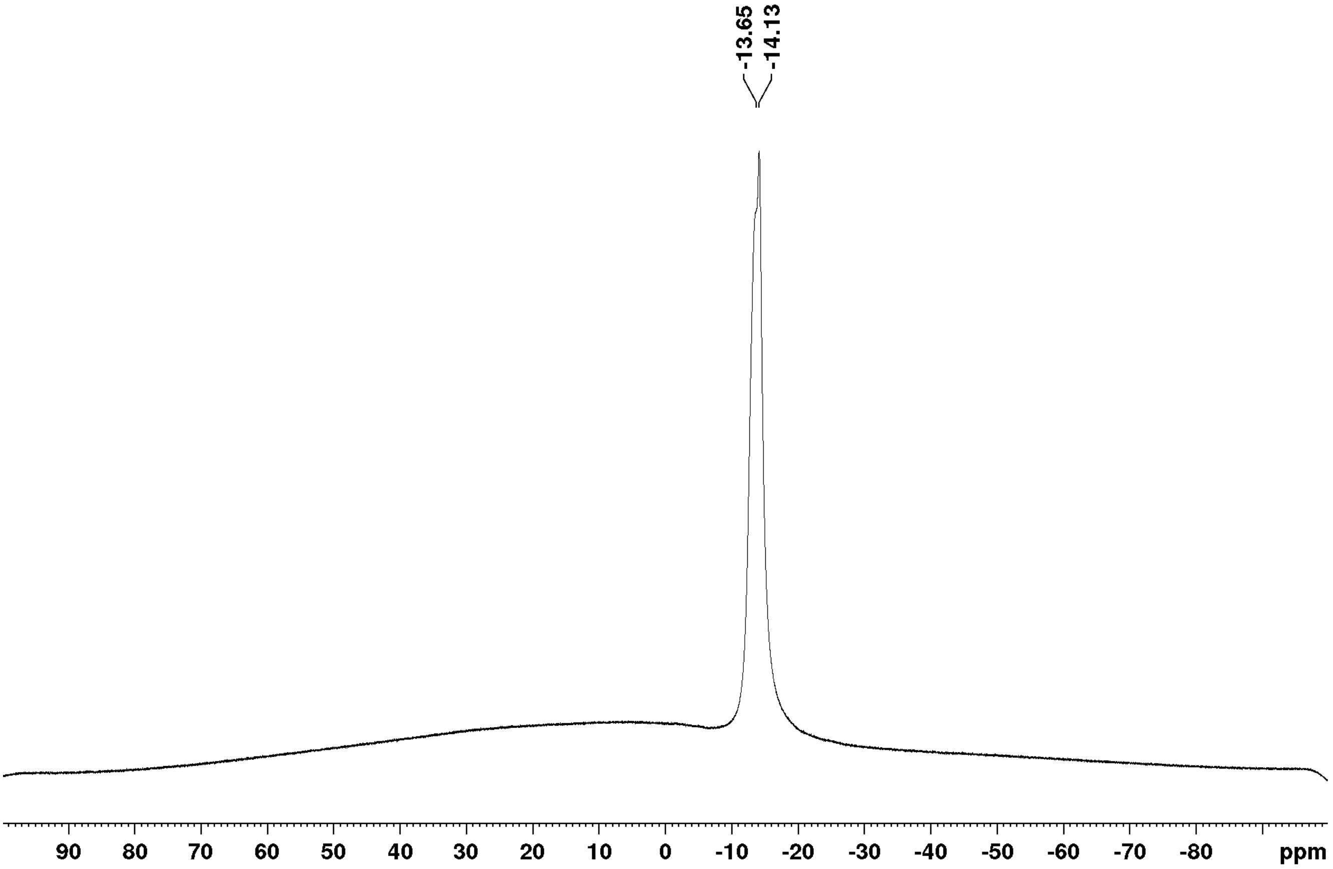

1-((*tert*-butyl)dimethylsilyl)hydroxymethyl)-2-(3-oxoprop-1-yn-1-yl)-12-isobutylcarbamoyl-1,12-dicarba-*closo*-dodecaborane (31b)

^{1}H NMR (500 MHz; $CDCl_3$)

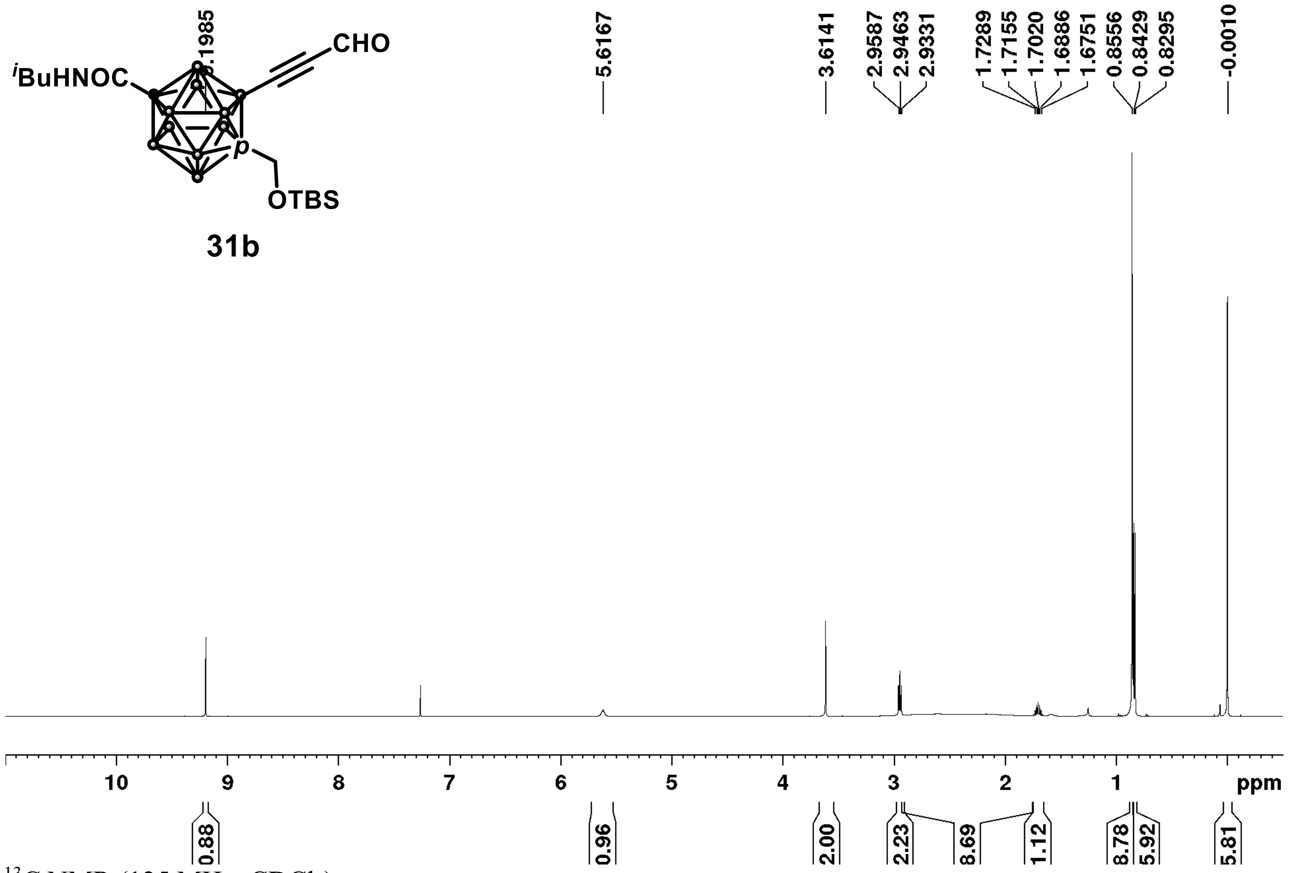

^{13}C NMR (125 MHz; $CDCl_3$)

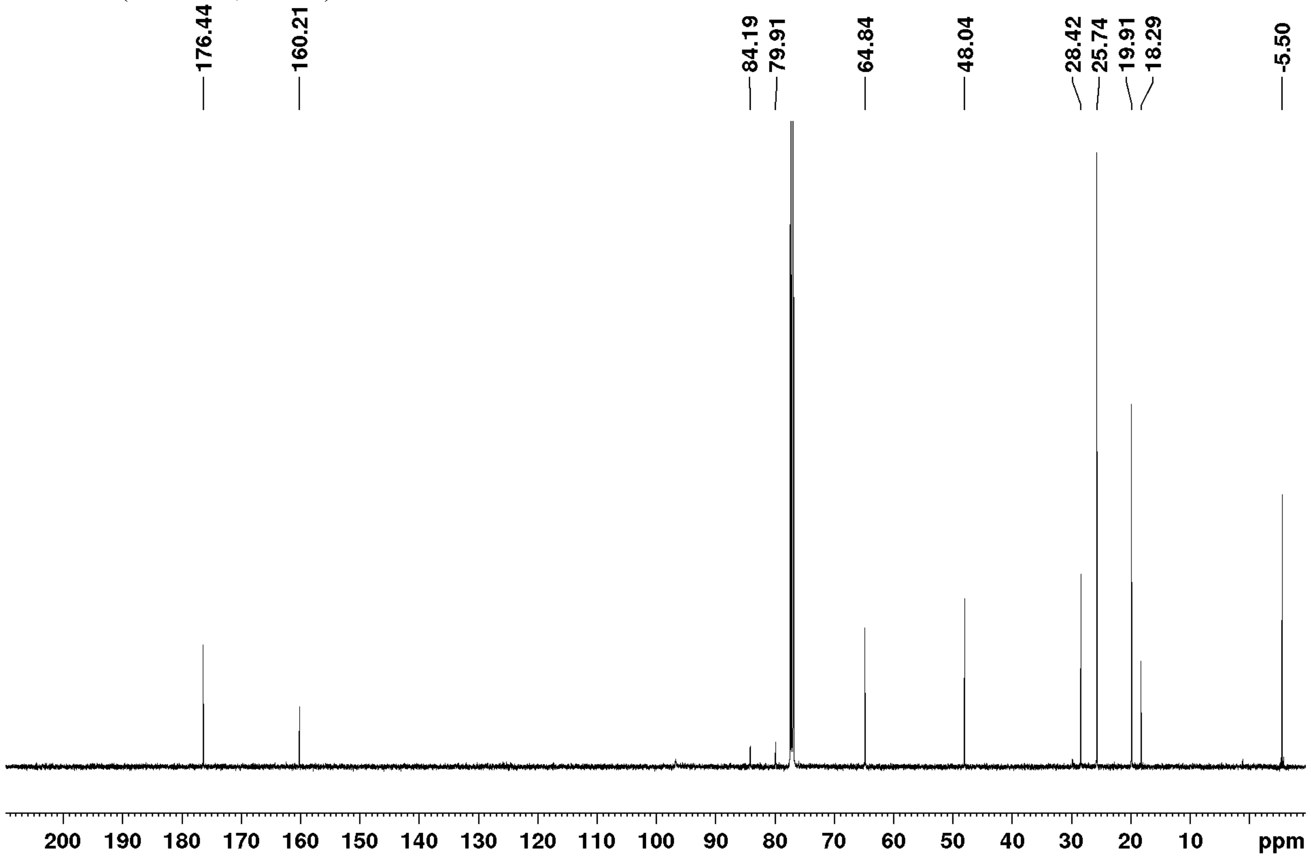

^{11}B NMR (160 MHz; $CDCl_3$)

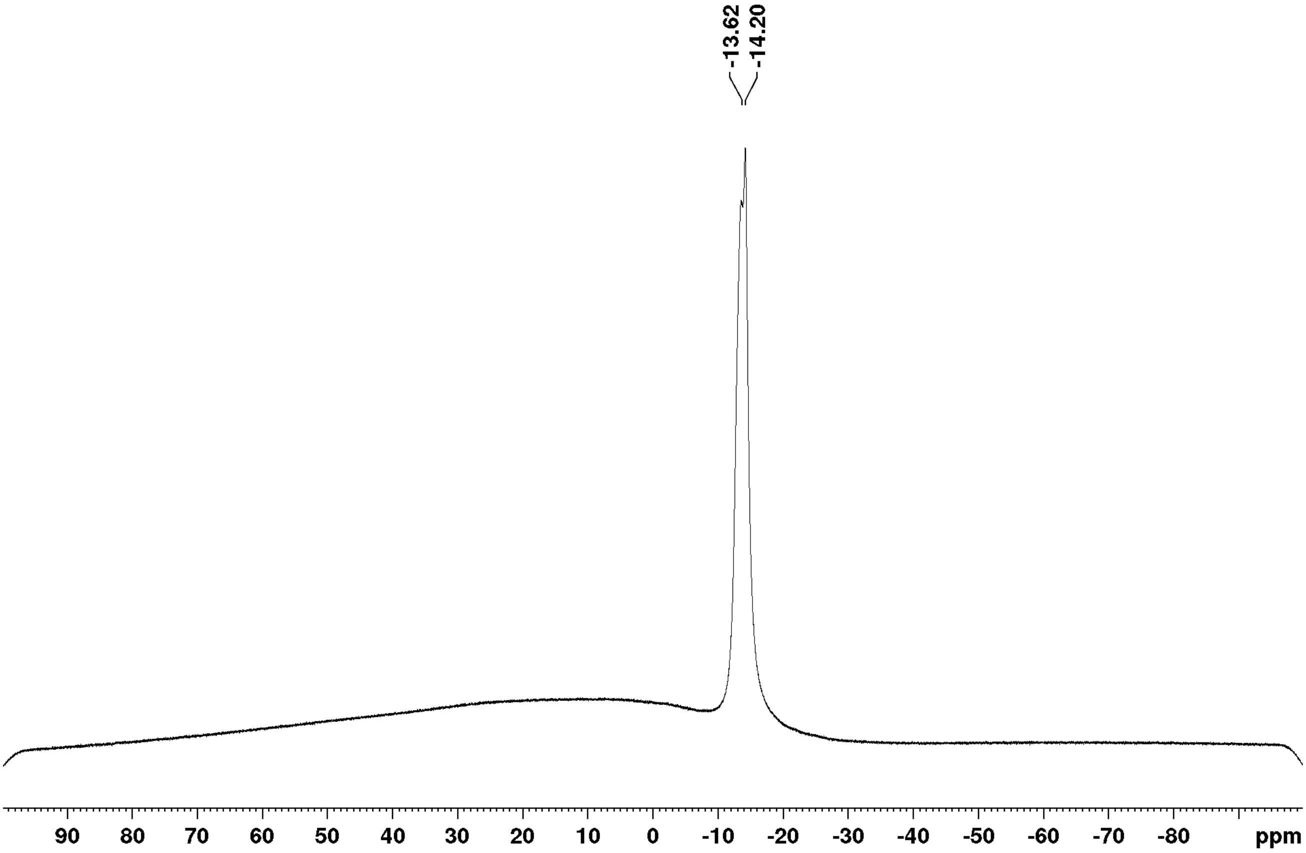

12-((*tert*-butyl)dimethylsilyl)hydroxymethyl)-2-(3-oxoprop-1-yn-1-yl)-1-benzylaminocarboxyl-1,12-dicarba-*closo*-dodecaborane (35a)

^{1}H NMR (500 MHz; $CDCl_3$)

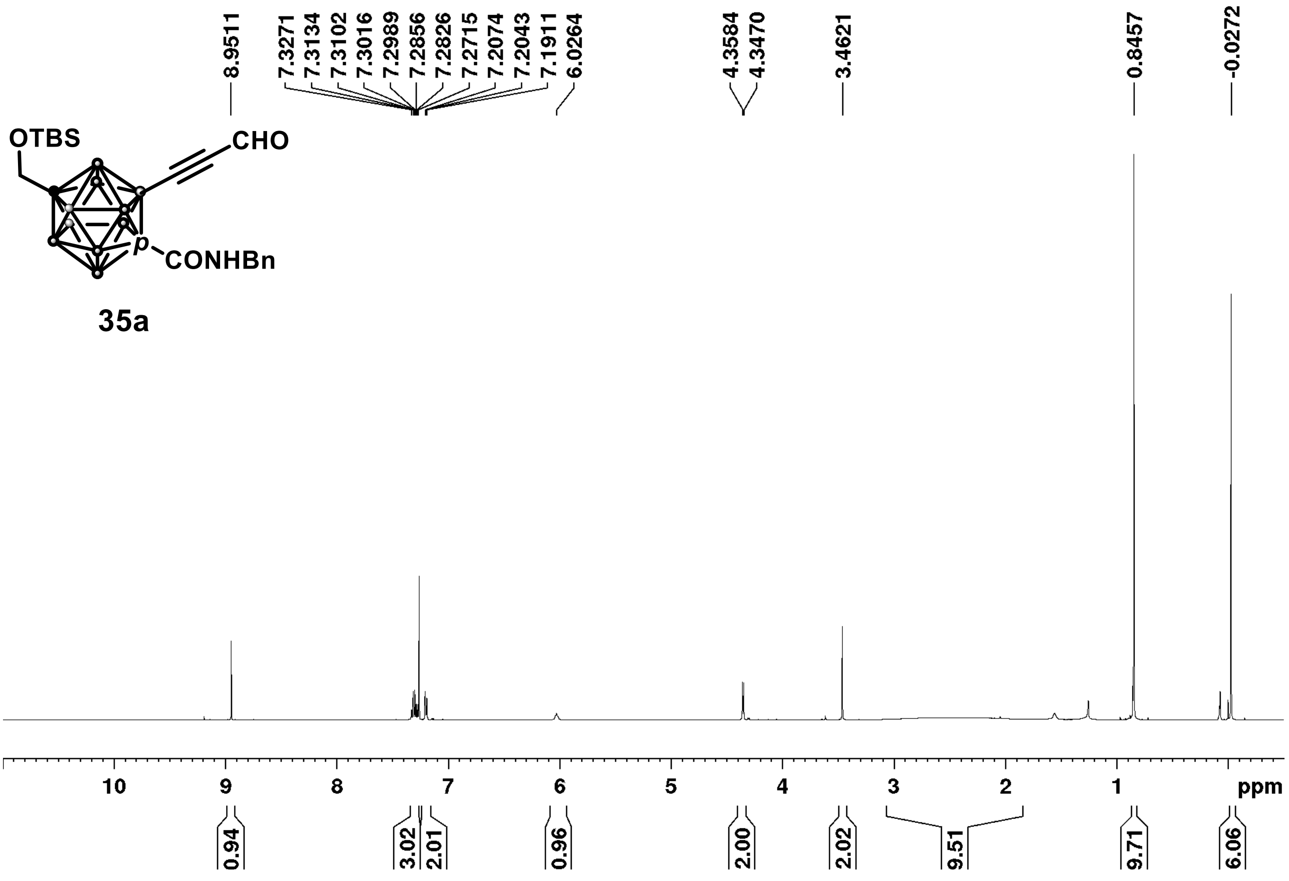

^{13}C NMR (125 MHz; $CDCl_3$)

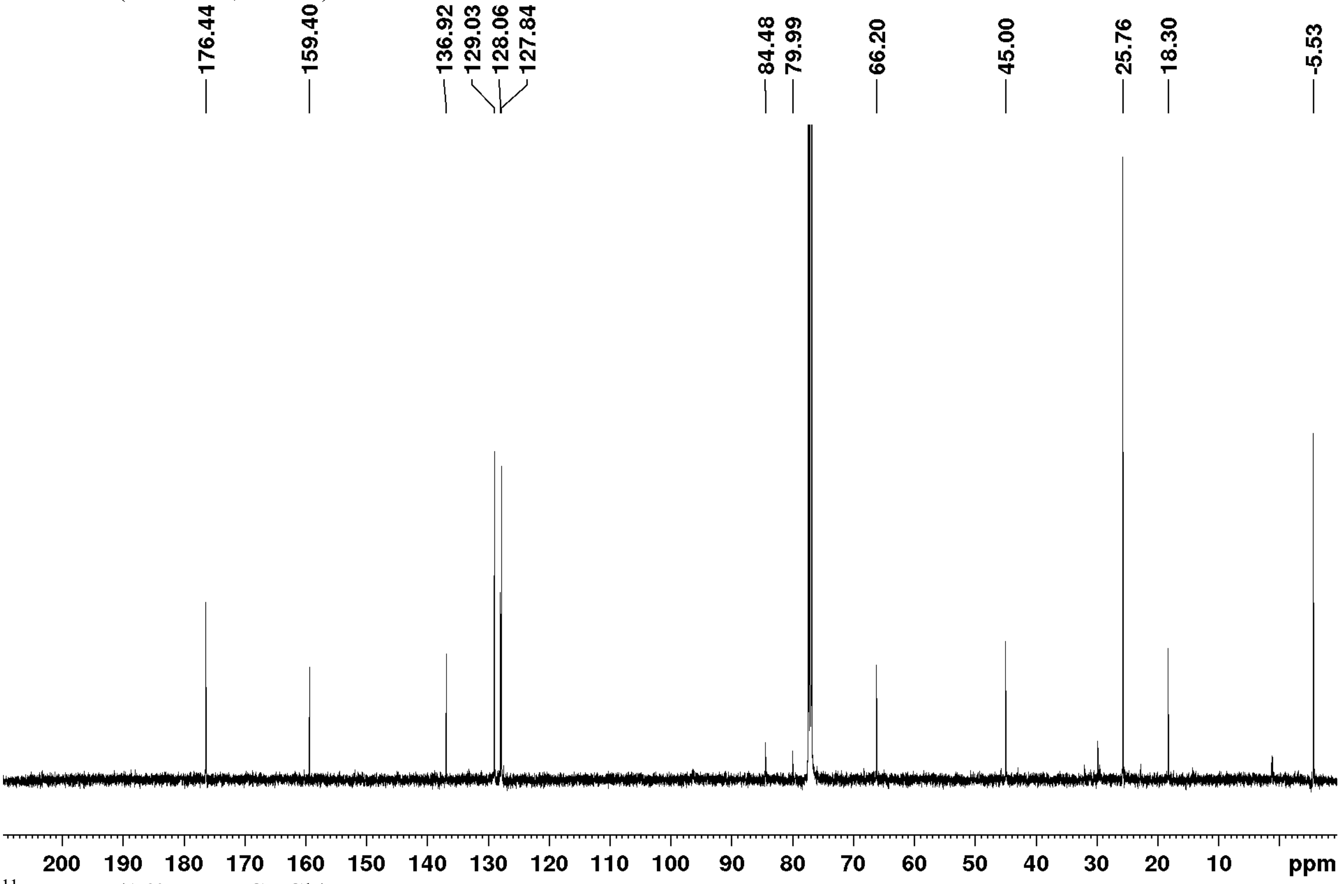

^{11}B NMR (160 MHz; $CDCl_3$)

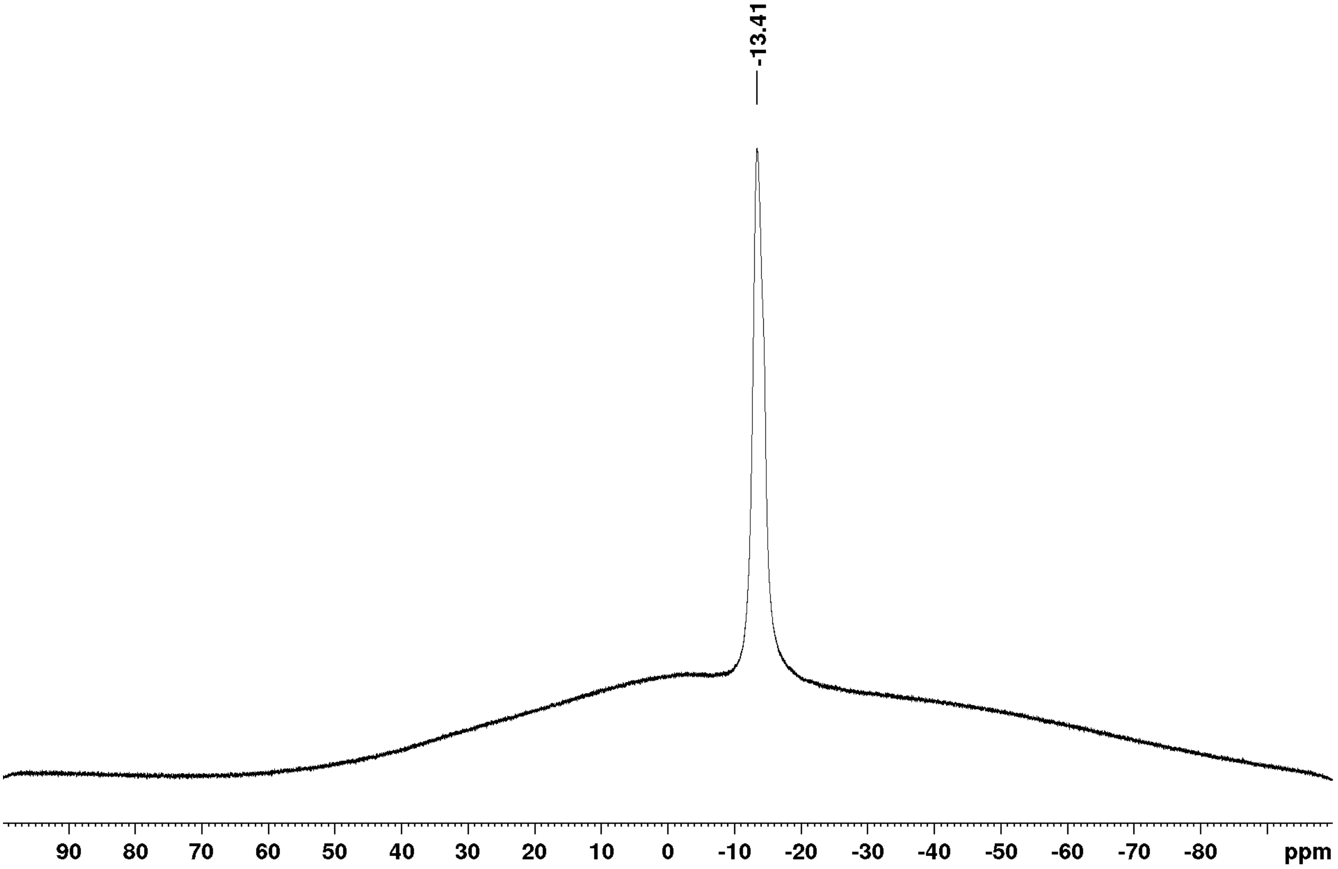

12-((*tert*-butyl)dimethylsilyl)hydroxymethyl)-2-(3-oxoprop-1-yn-1-yl)-1-isobutylcarbamoyl-1,12-dicarba-*closo*-dodecaborane (35b)

^{1}H NMR (500 MHz; $CDCl_3$)

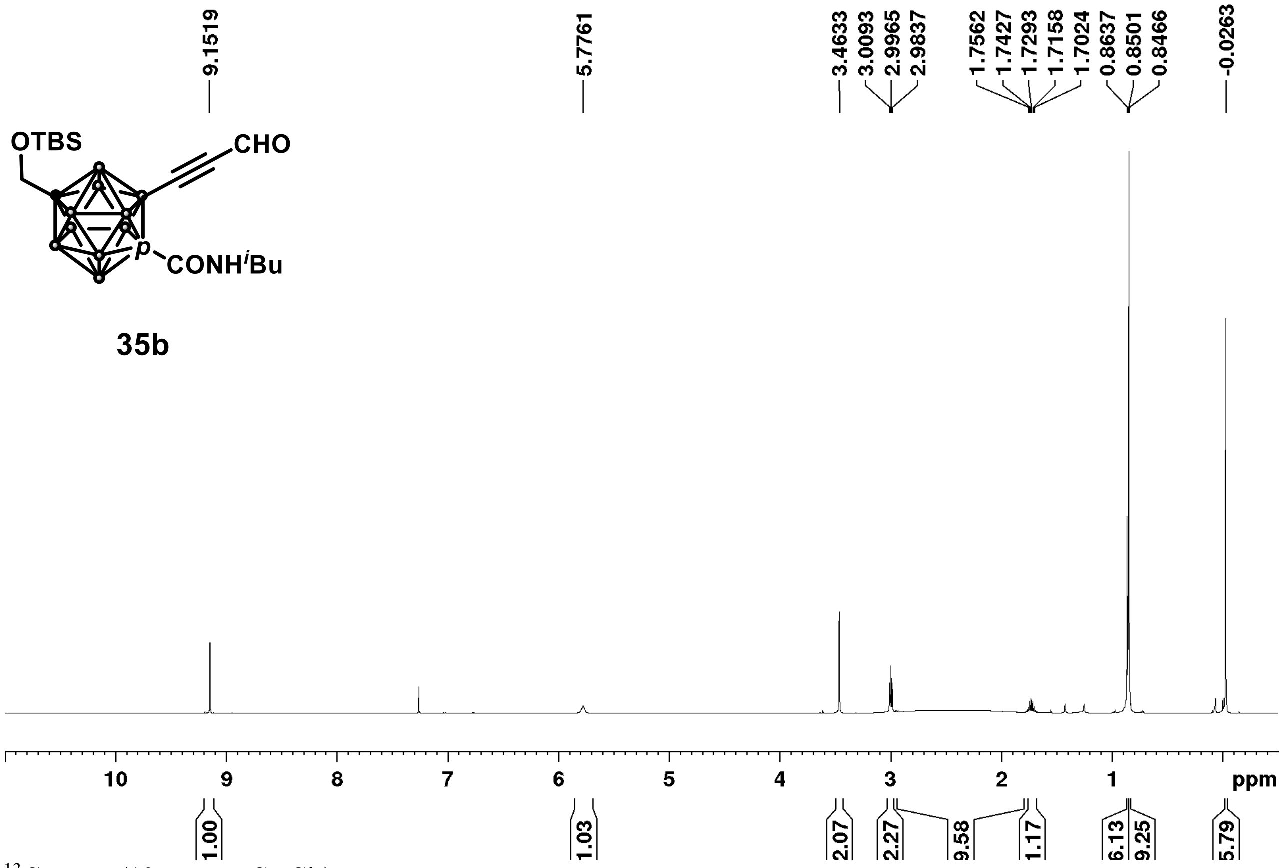

^{13}C NMR (125 MHz; $CDCl_3$)

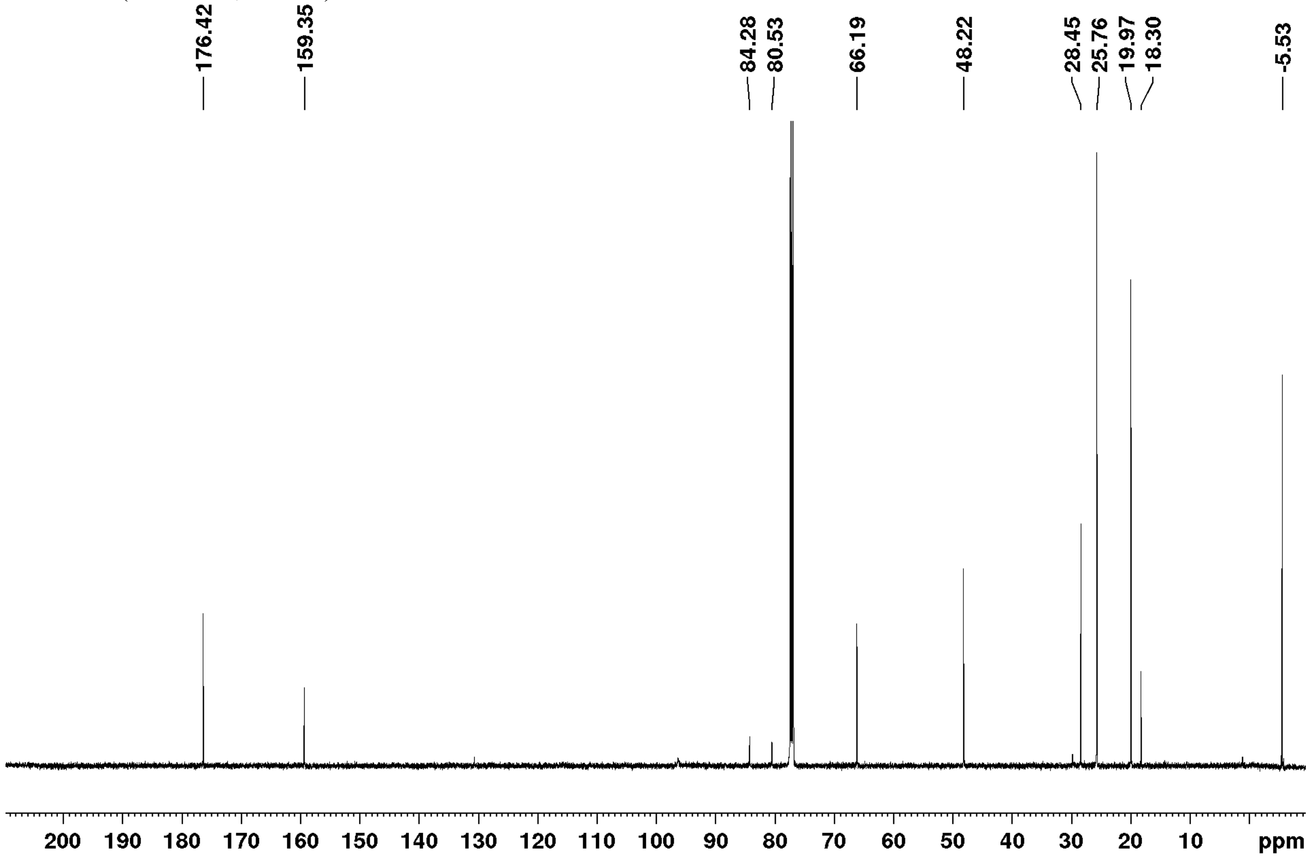

^{11}B NMR (160 MHz; $CDCl_3$)

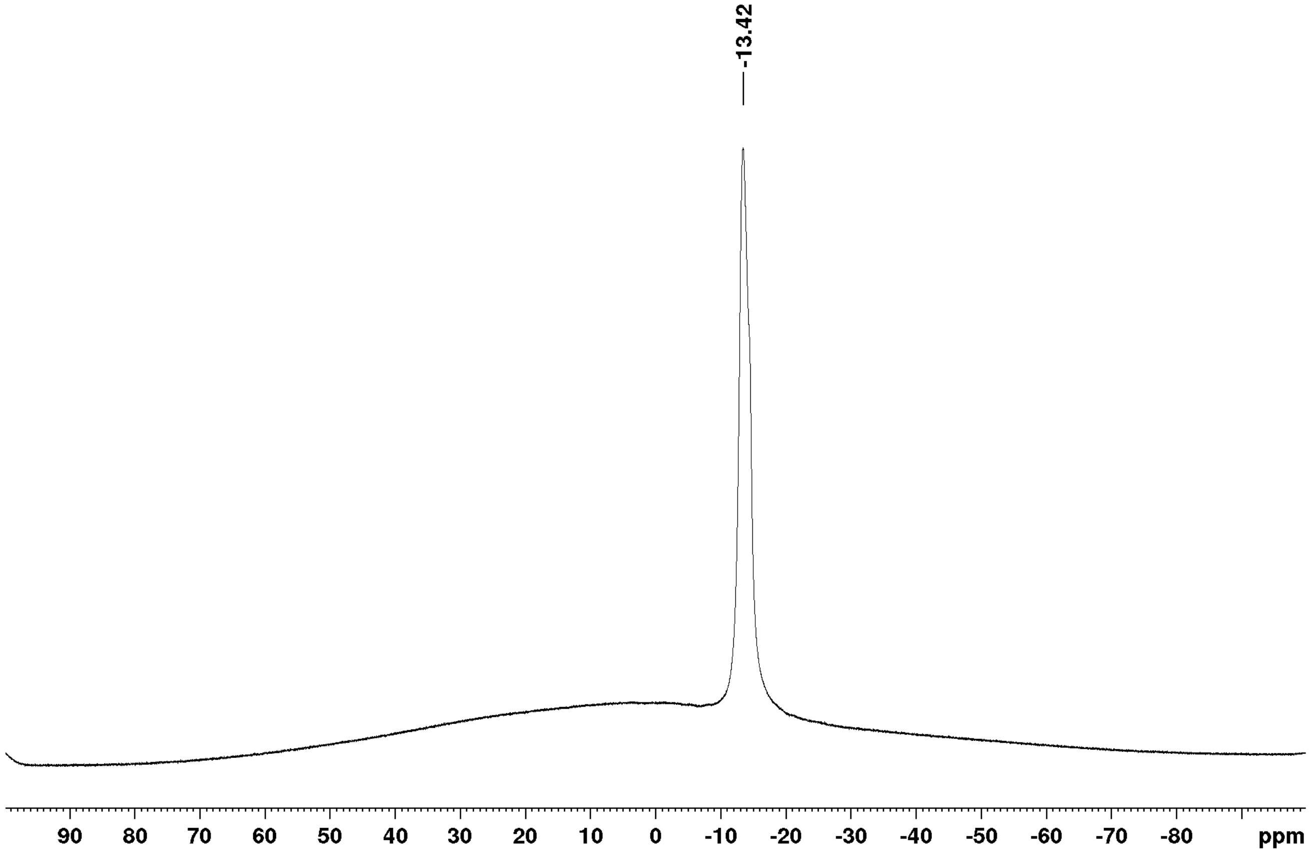

1-(hydroxymethyl)-2-(3-(benzylamino)-3-oxoprop-1-yn-1-yl)-12-benzylaminocarboxyl-1,12-dicarba-*closo*-dodecaborane (32a)

^{1}H NMR (500 MHz; $CDCl_3$)

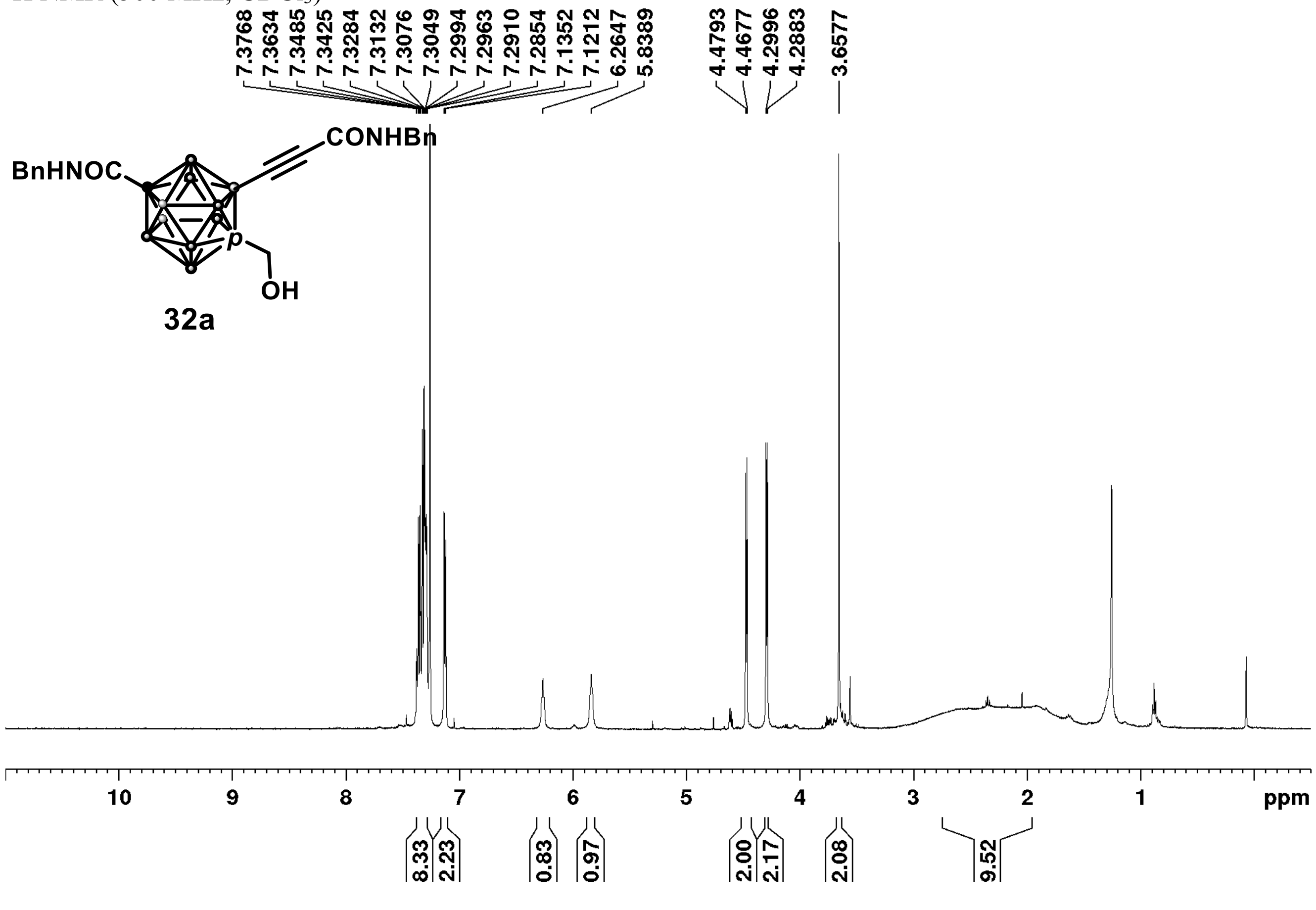

^{13}C NMR (125 MHz; $CDCl_3$)

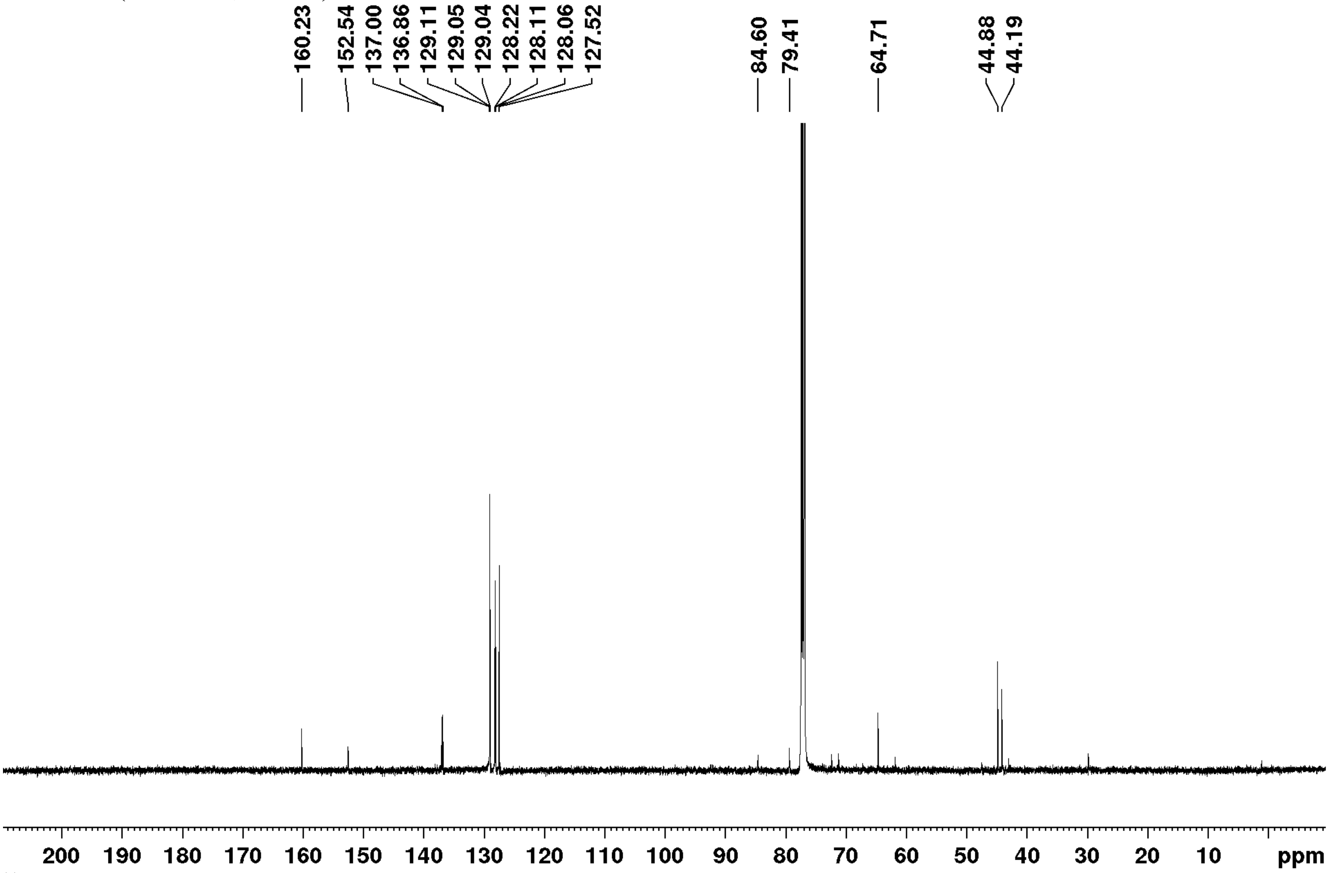

^{11}B NMR (160 MHz; $CDCl_3$)

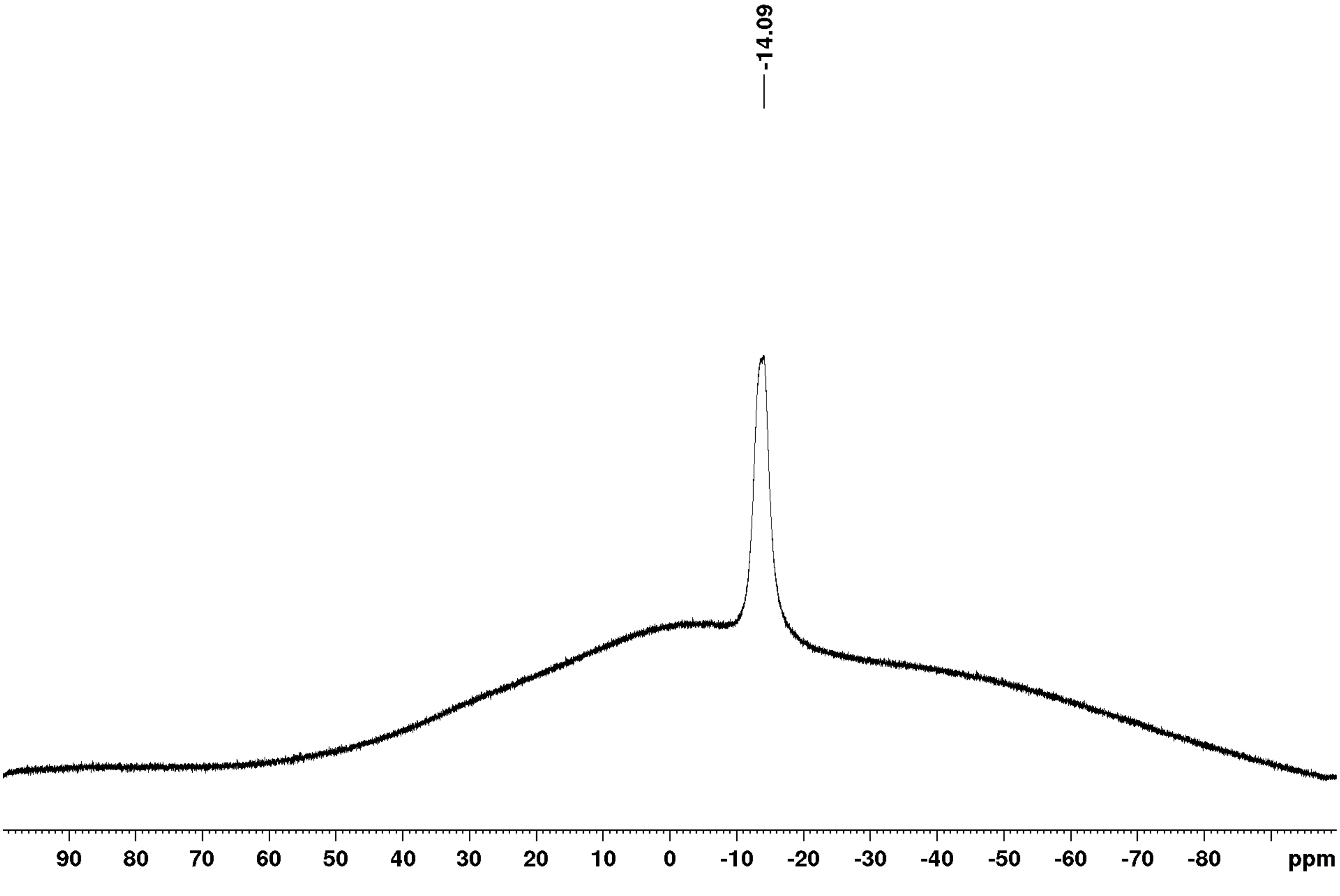

1-(hydroxymethyl)-2-(3-(isobutylamino)-3-oxoprop-1-yn-1-yl)-12-benzylaminocarboxyl-1,12-dicarba-*closo*-dodecaborane (32b)

^{1}H NMR (500 MHz; $CDCl_3$)

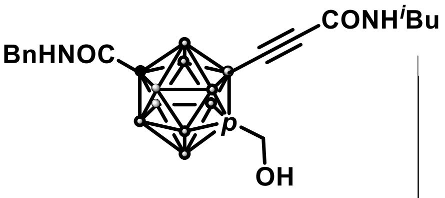

32b

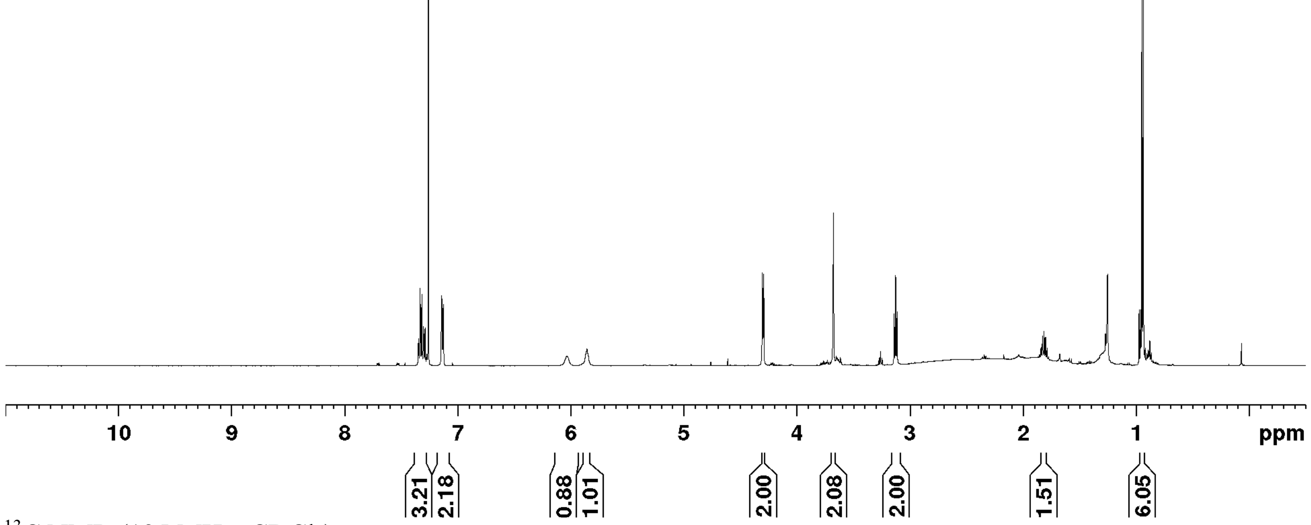

^{13}C NMR (125 MHz; $CDCl_3$)

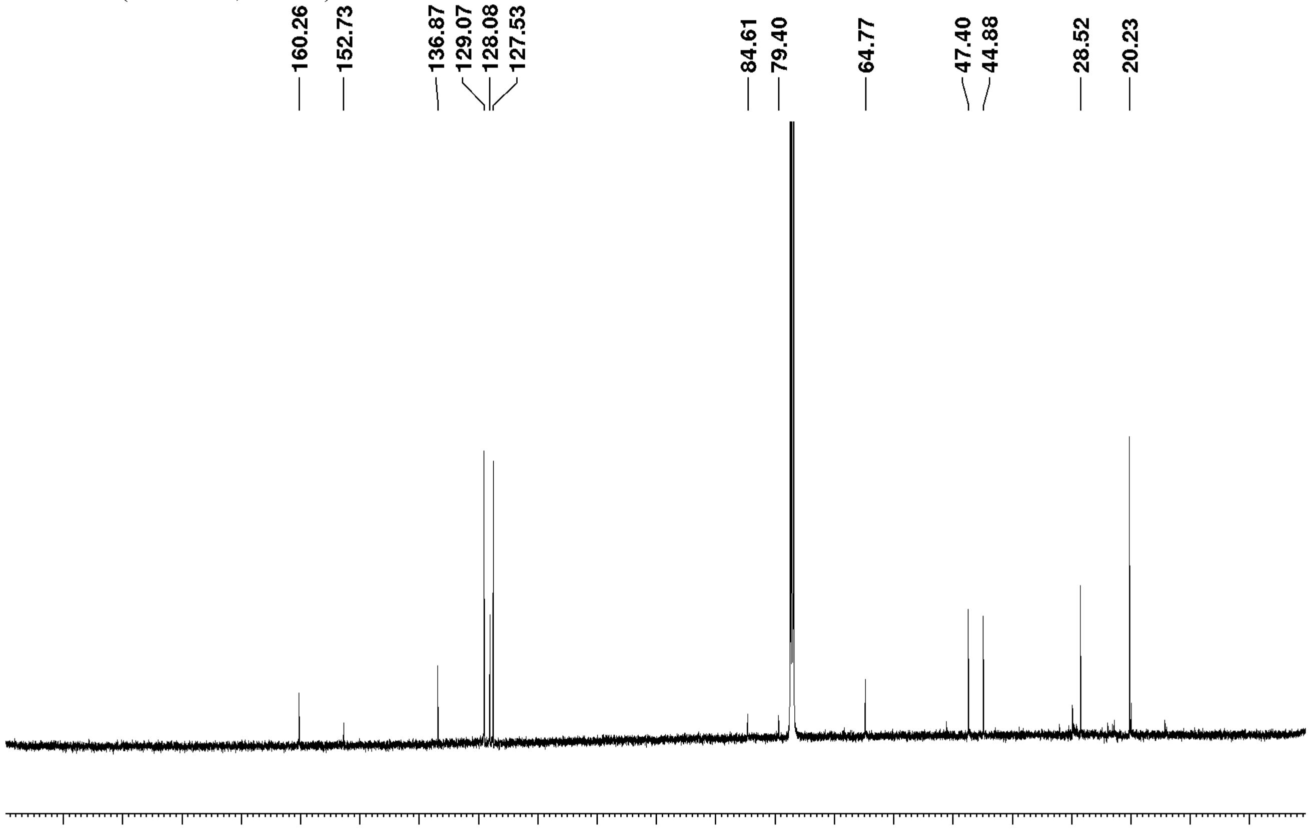

^{11}B NMR (160 MHz; $CDCl_3$)

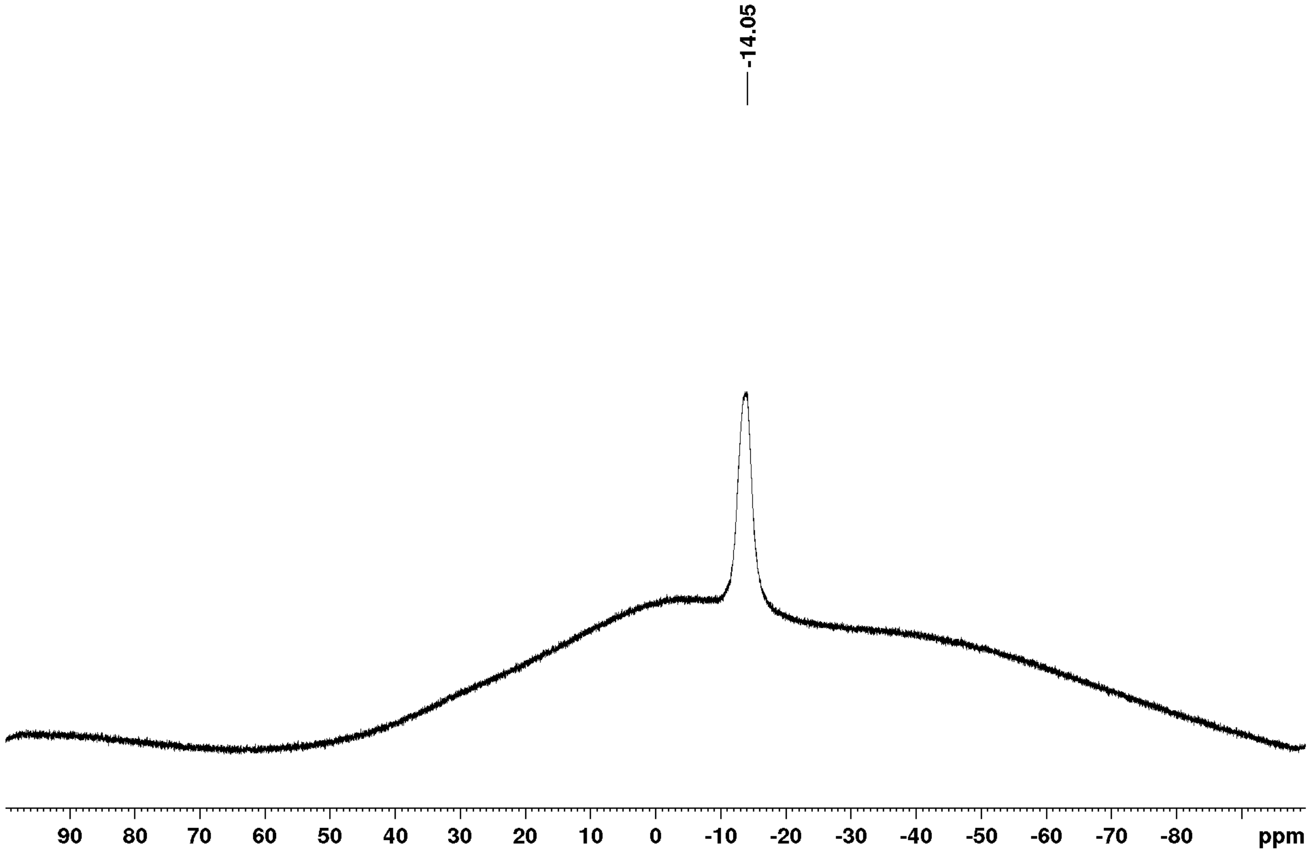

1-(hydroxymethyl)-2-(3-(benzylamino)-3-oxoprop-1-yn-1-yl)-12-isobutylcarbamoyl-1,12-dicarba-*closo*-dodecaborane (32c)

^1H NMR (500 MHz; $CDCl_3$)

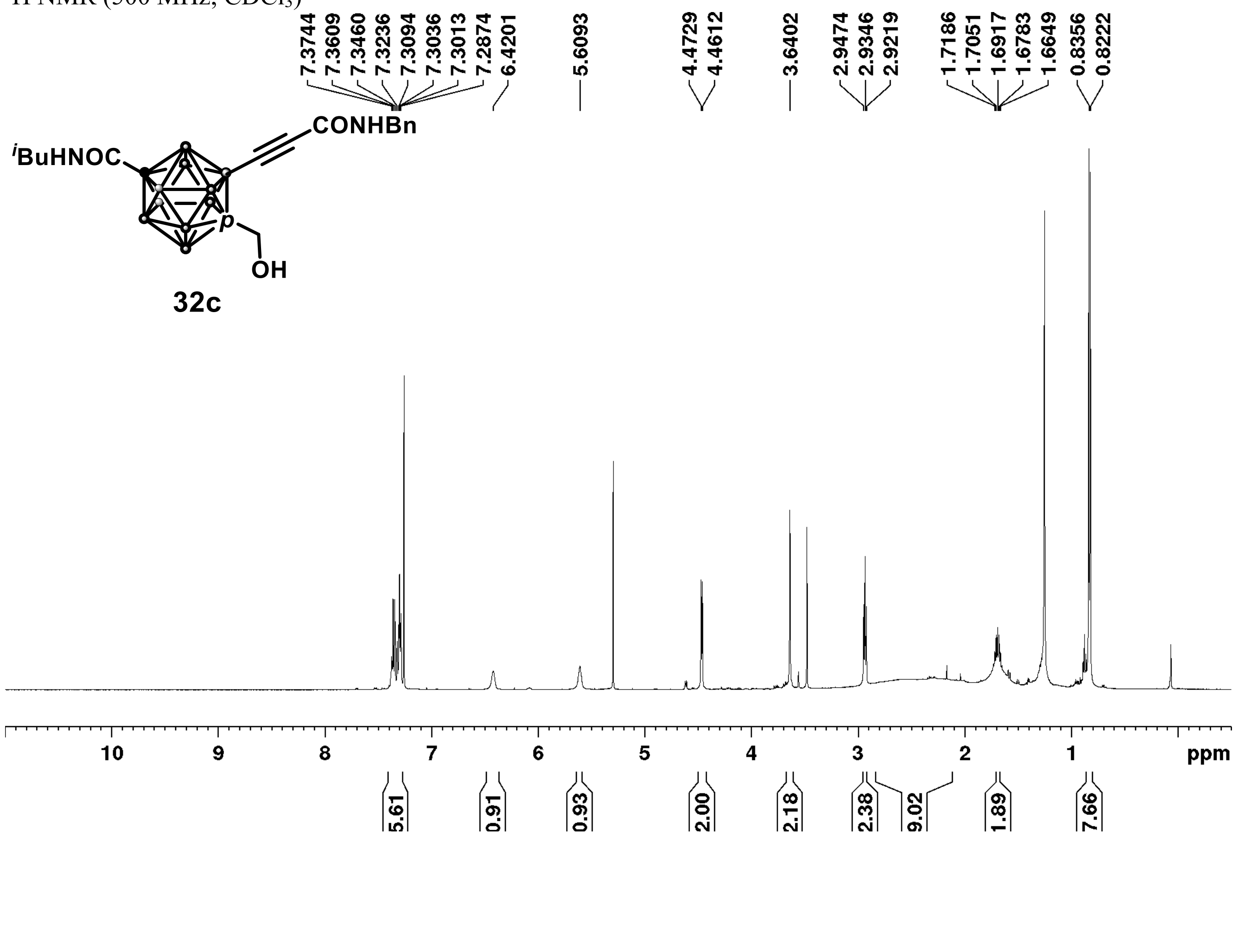

^{13}C NMR (125 MHz; $CDCl_3$)

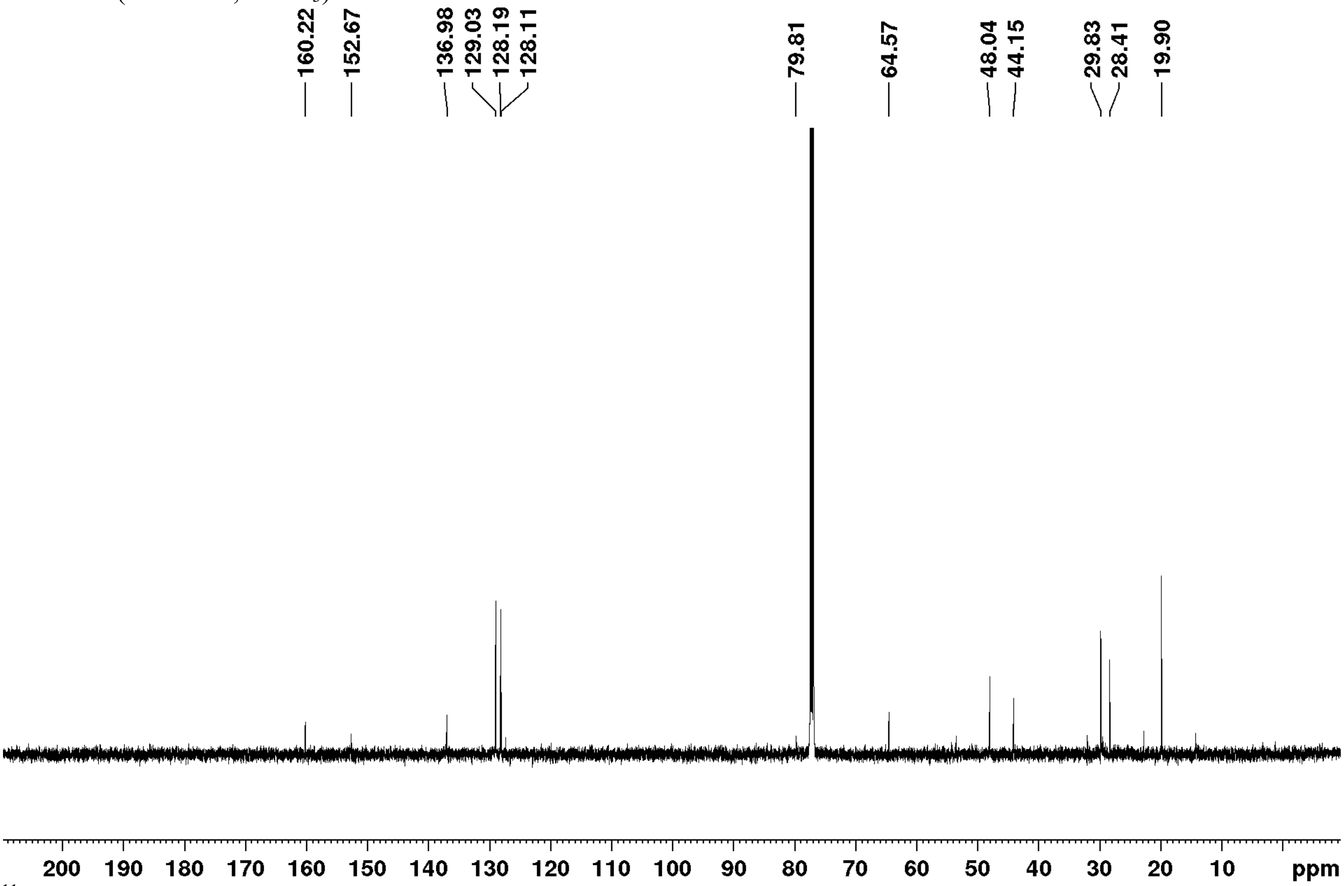

^{11}B NMR (160 MHz; $CDCl_3$)

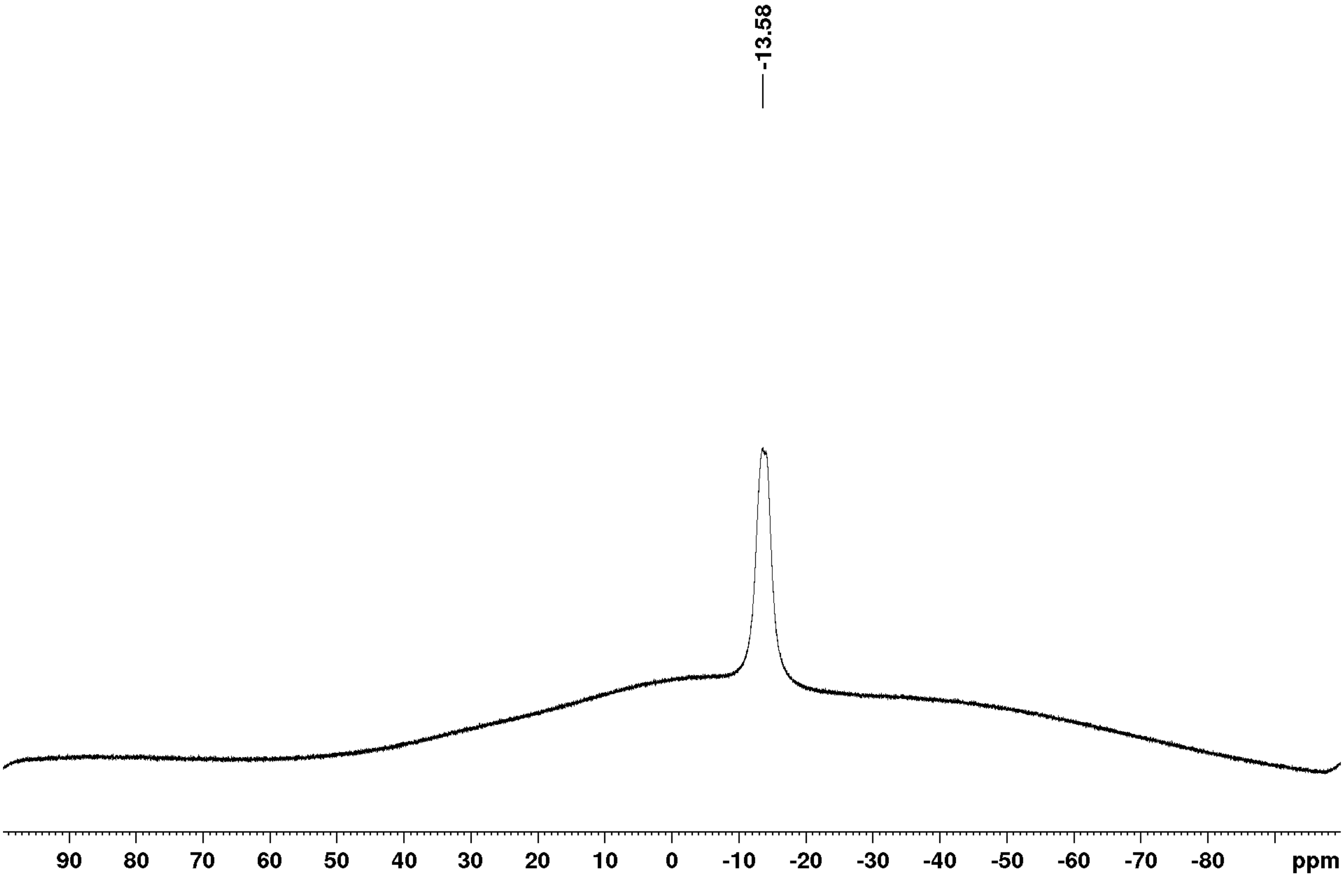

1-(hydroxymethyl)-2-(3-(isobutylamino)-3-oxoprop-1-yn-1-yl)-12-isobutylcarbamoyl-1,12-dicarba-*closo*-dodecaborane (32d)

^{1}H NMR (500 MHz; $CDCl_3$)

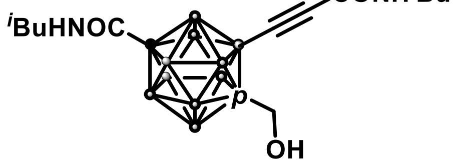

32d

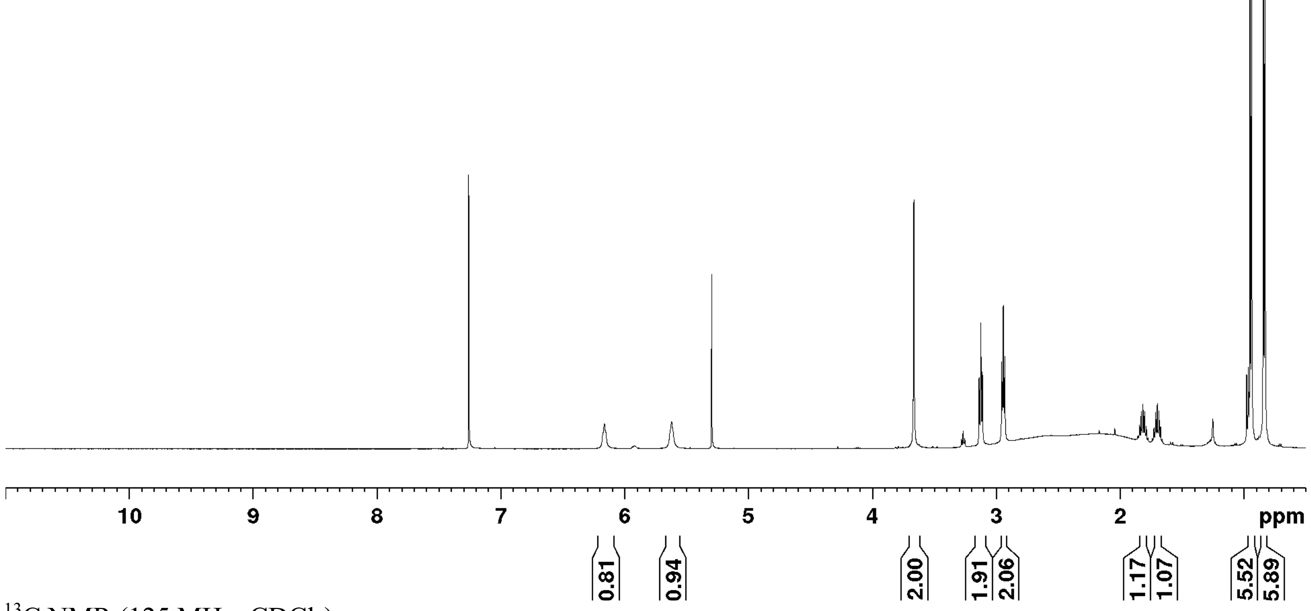

^{13}C NMR (125 MHz; $CDCl_3$)

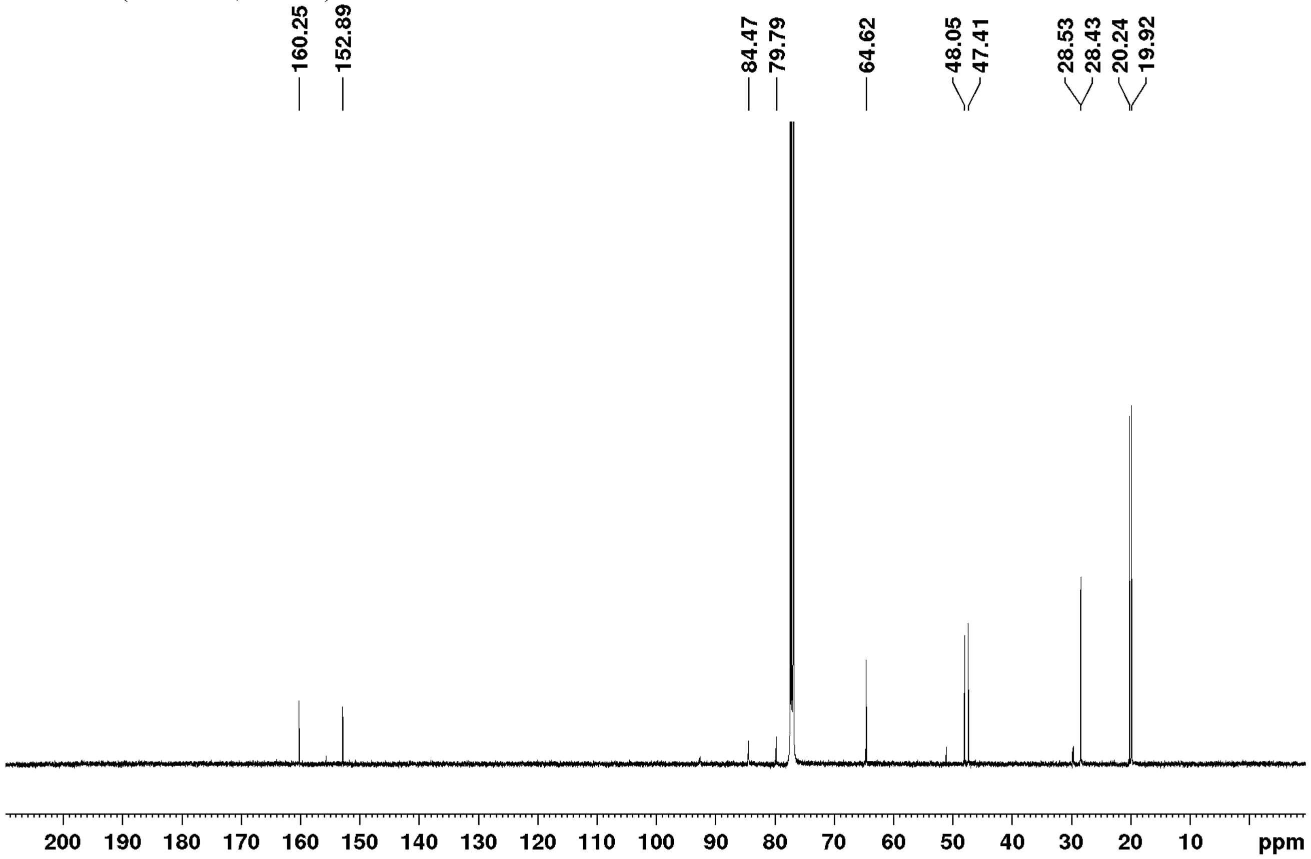

^{11}B NMR (160 MHz; $CDCl_3$)

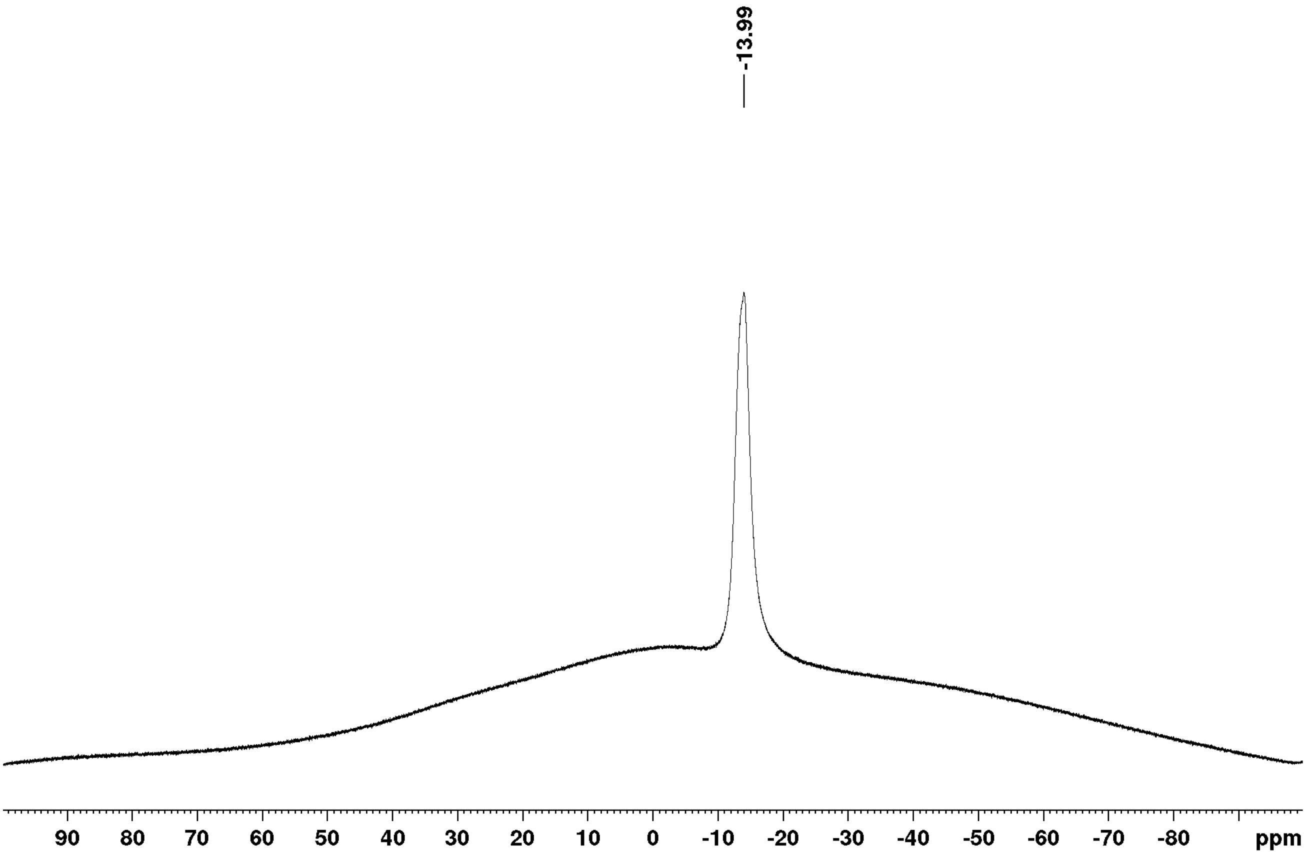

12-(hydroxymethyl)-2-(3-(benzylamino)-3-oxoprop-1-yn-1-yl)-1-benzylaminocarboxyl-1,12-dicarba-*closo*-dodecaborane (36a)

^1H NMR (500 MHz; $CDCl_3$)

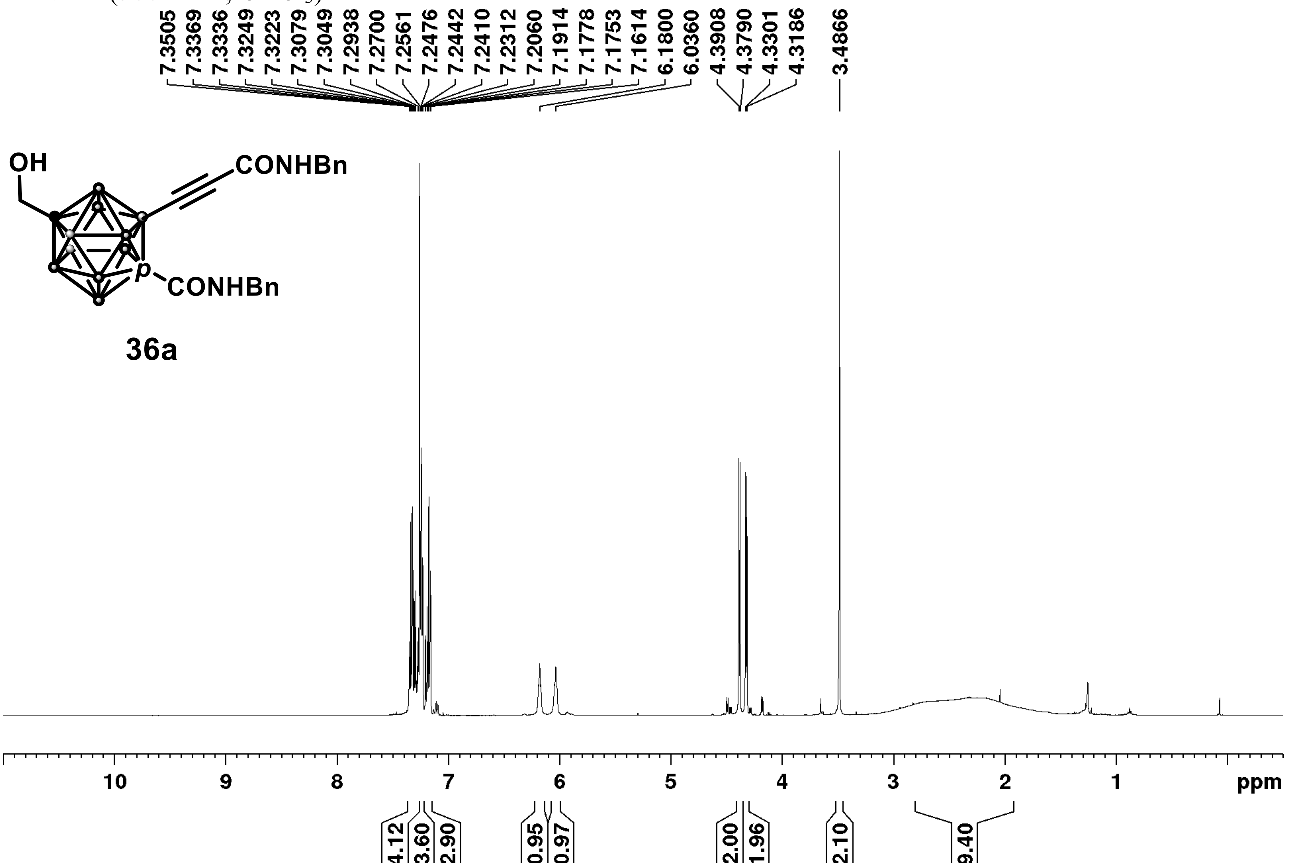

^{13}C NMR (125 MHz; $CDCl_3$)

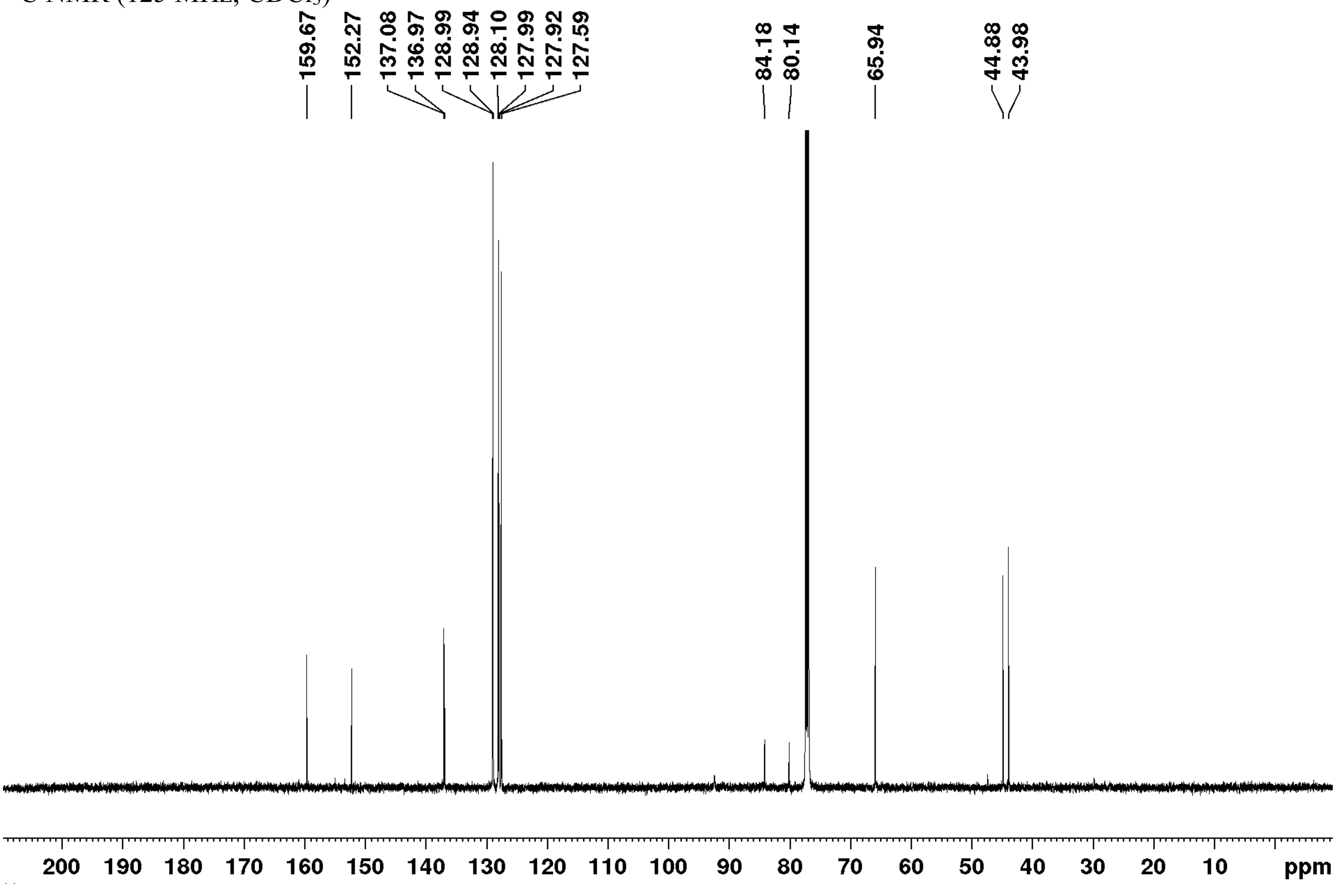

^{11}B NMR (160 MHz; $CDCl_3$)

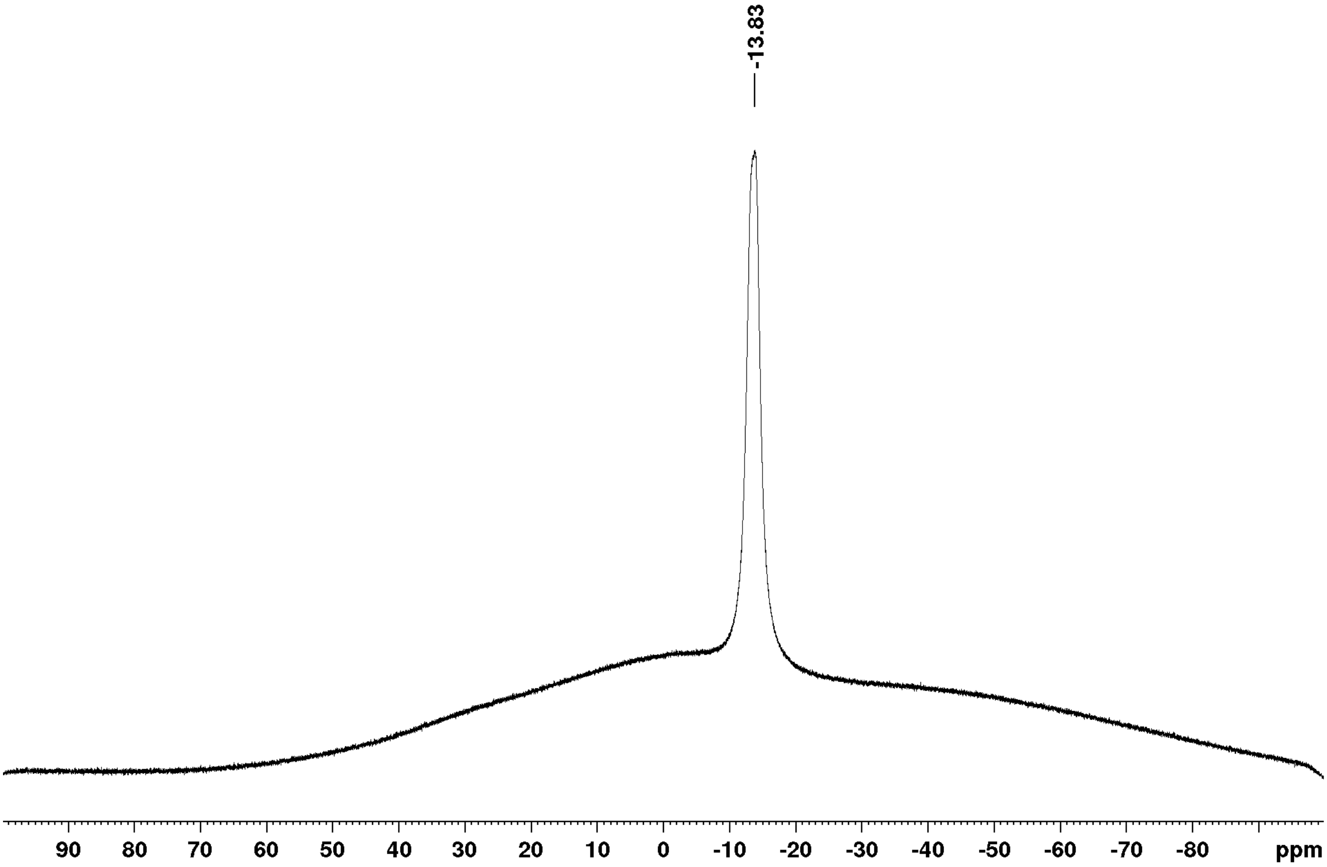

12-(hydroxymethyl)-2-(3-(isobutylamino)-3-oxoprop-1-yn-1-yl)-1-benzylaminocarboxyl-1,12-dicarba-*closo*-dodecaborane (36b)

^{1}H NMR (500 MHz; $CDCl_3$)

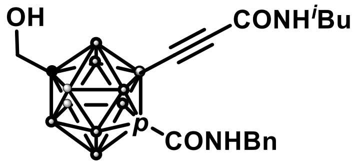

36b

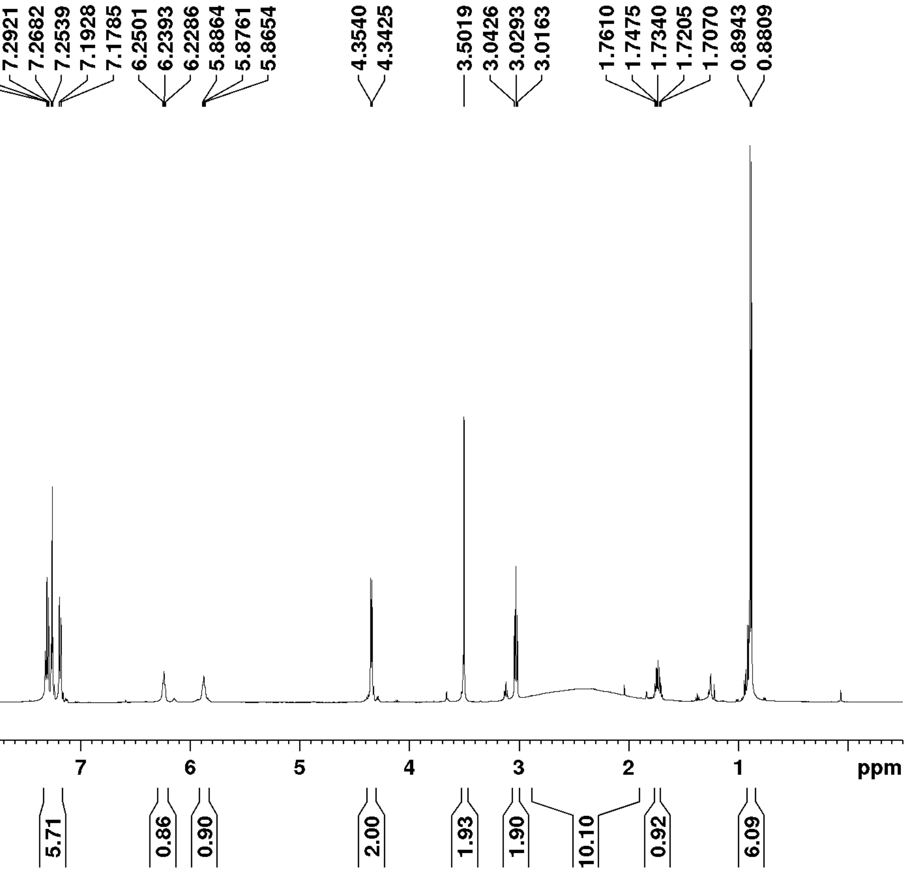

^{13}C NMR (125 MHz; $CDCl_3$)

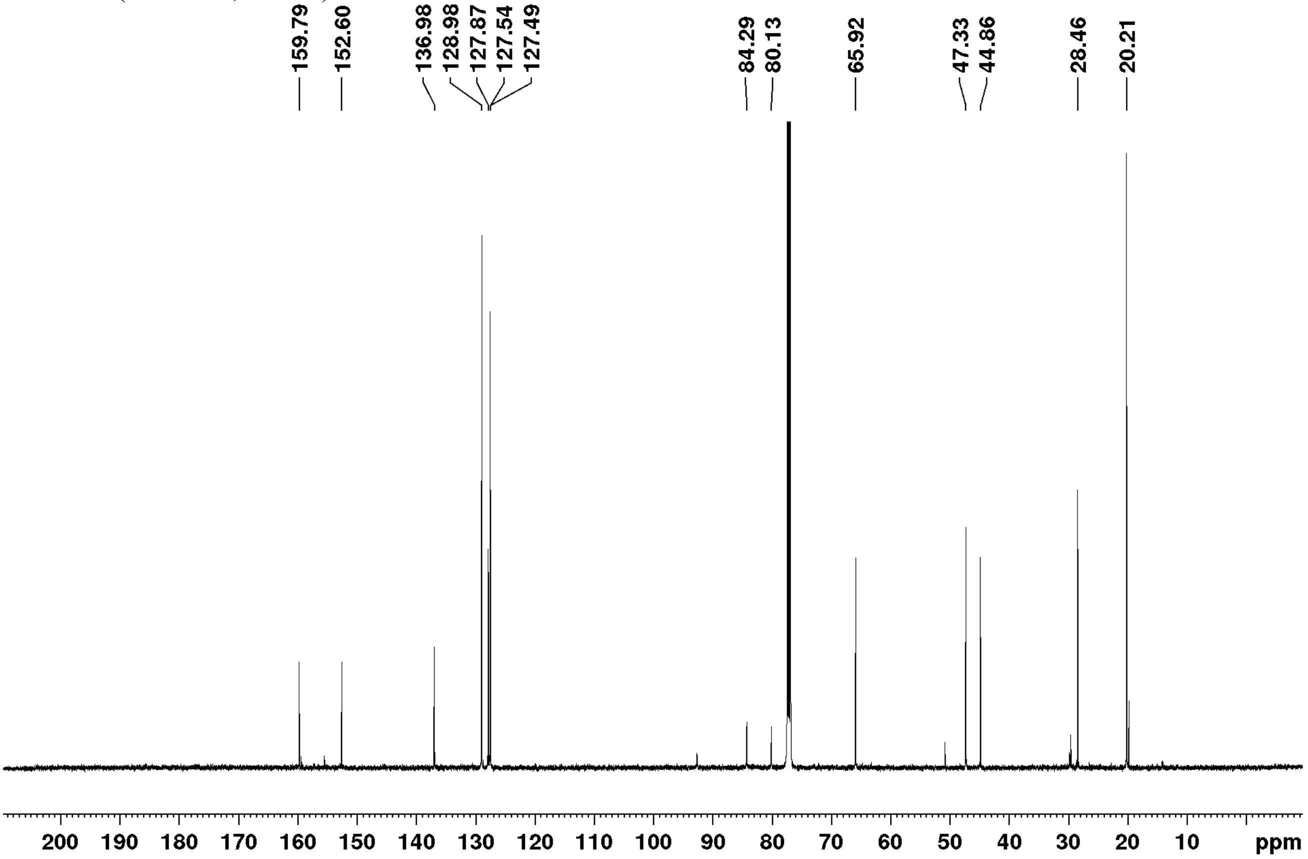

^{11}B NMR (160 MHz; $CDCl_3$)

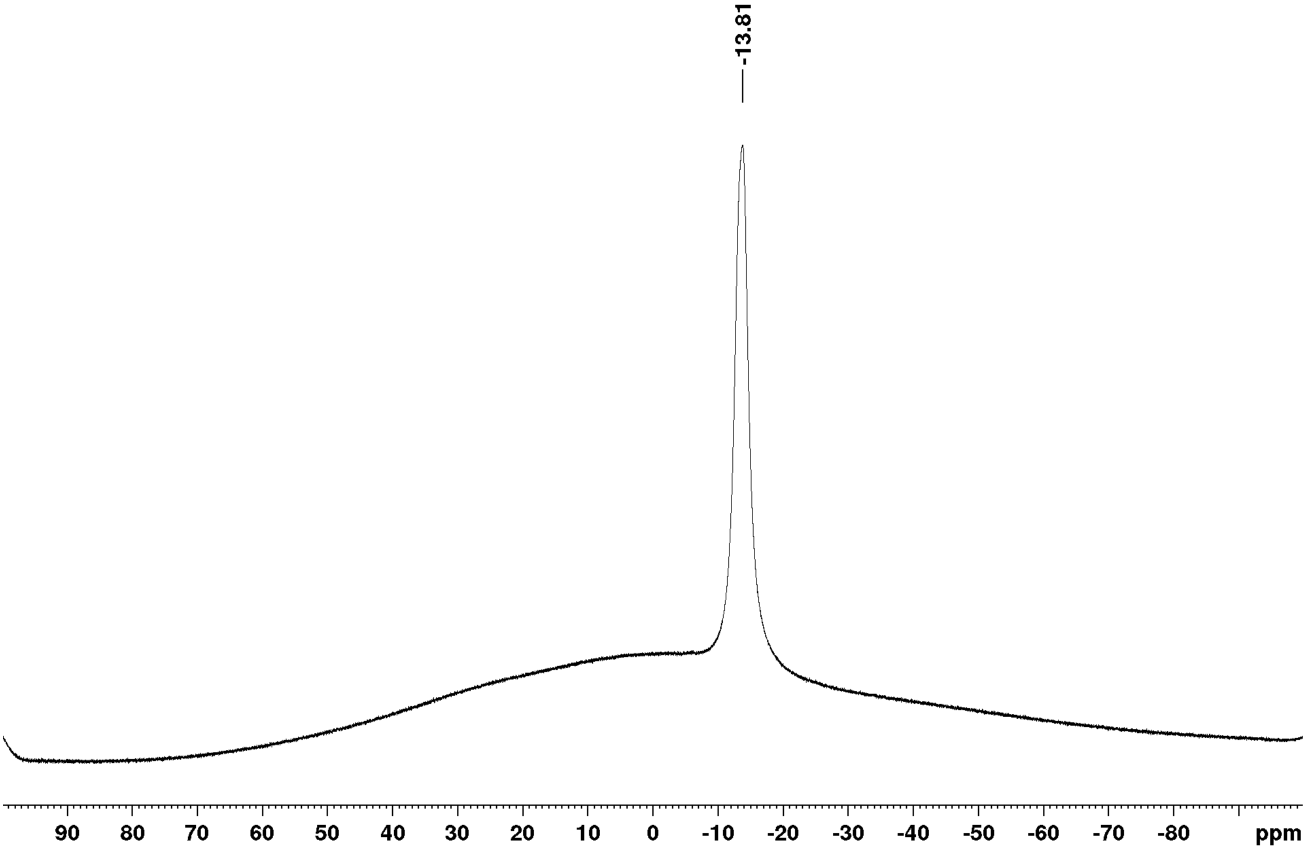

12-(hydroxymethyl)-2-(3-(benzylamino)-3-oxoprop-1-yn-1-yl)-1-isobutylcarbamoyl-1,12-dicarba-*closo*-dodecaborane (36c)

^{1}H NMR (500 MHz; $CDCl_3$)

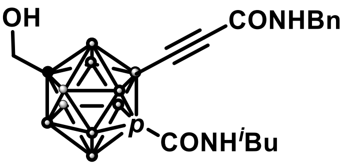

36c

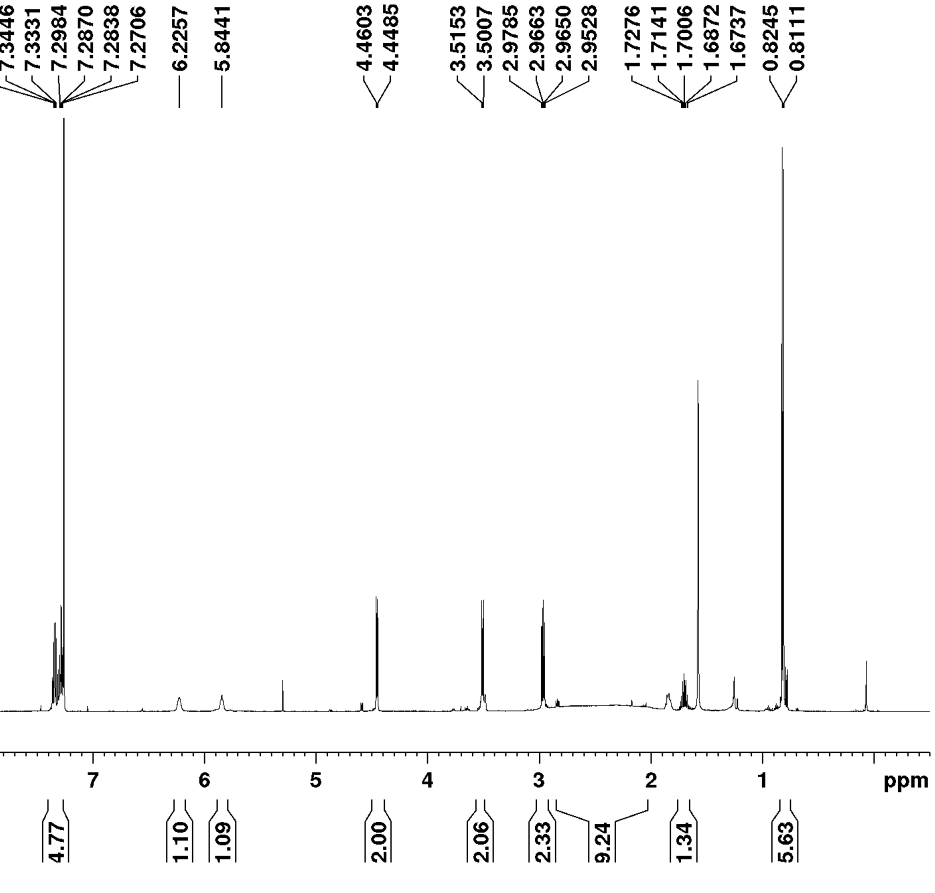

^{13}C NMR (125 MHz; $CDCl_3$)

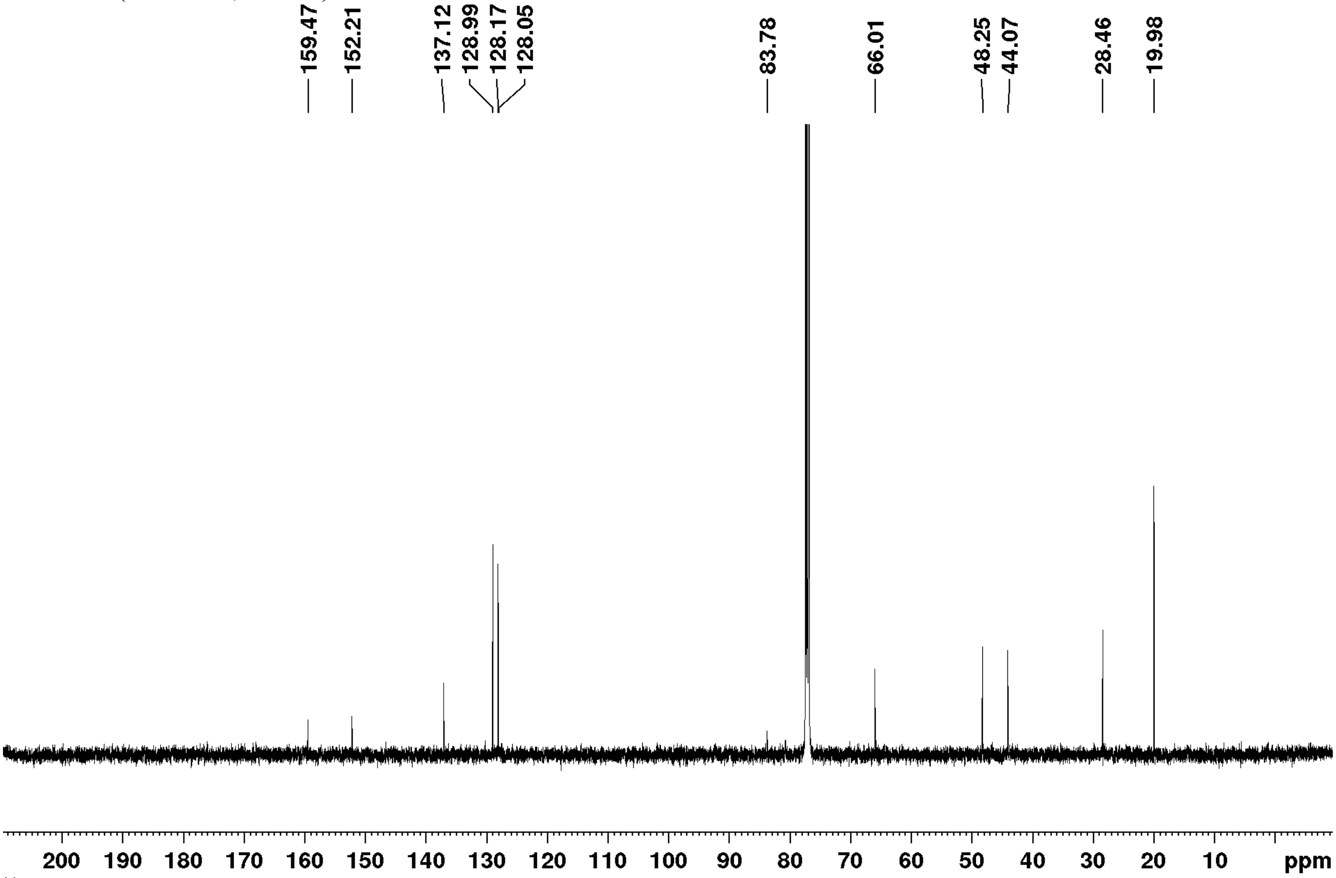

^{11}B NMR (160 MHz; $CDCl_3$)

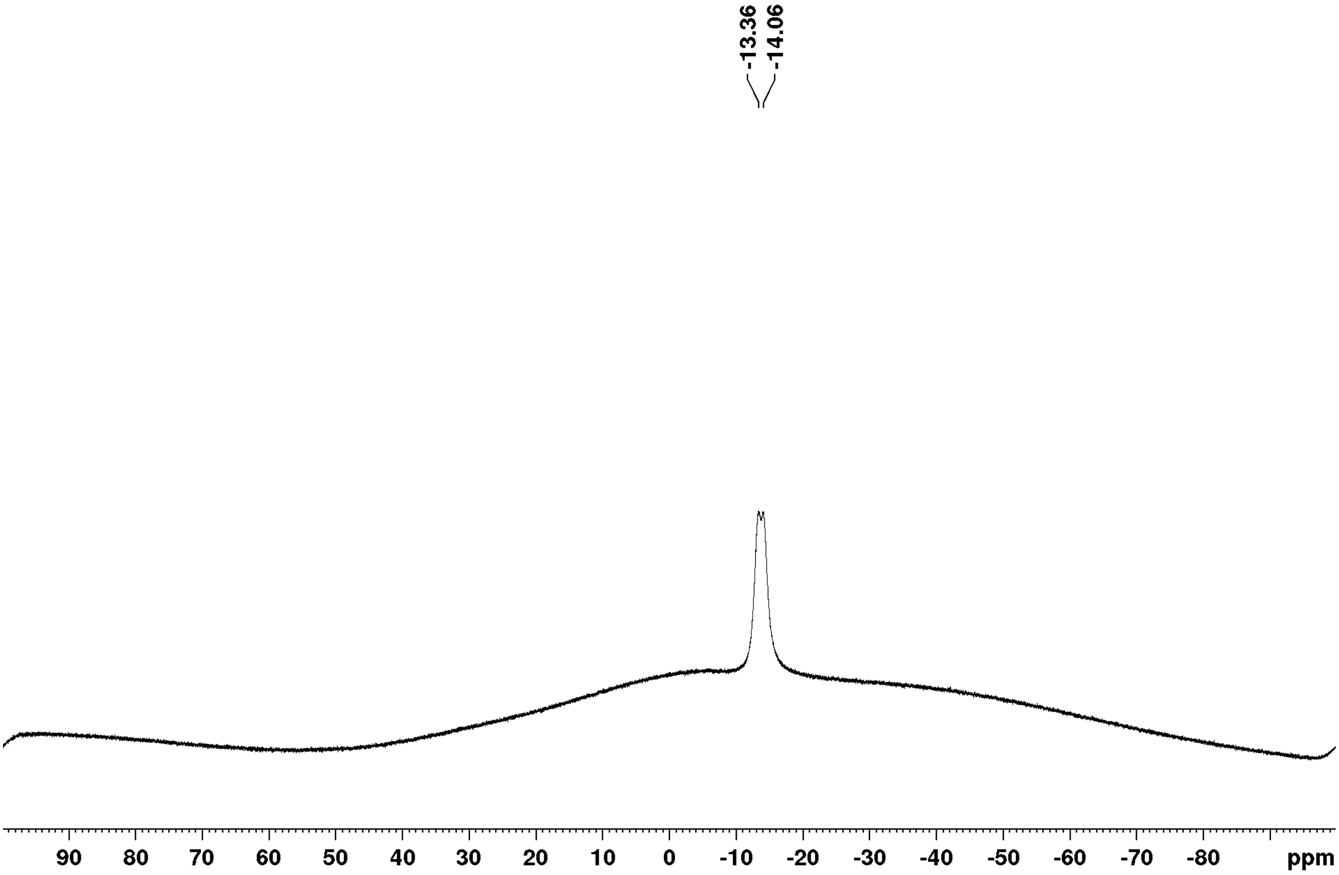

12-(hydroxymethyl)-2-(3-(isobutylamino)-3-oxoprop-1-yn-1-yl)-1-isobutylcarbamoyl-1,12-dicarba-*closo*-dodecaborane (36d)

^{1}H NMR (500 MHz; $CDCl_3$)

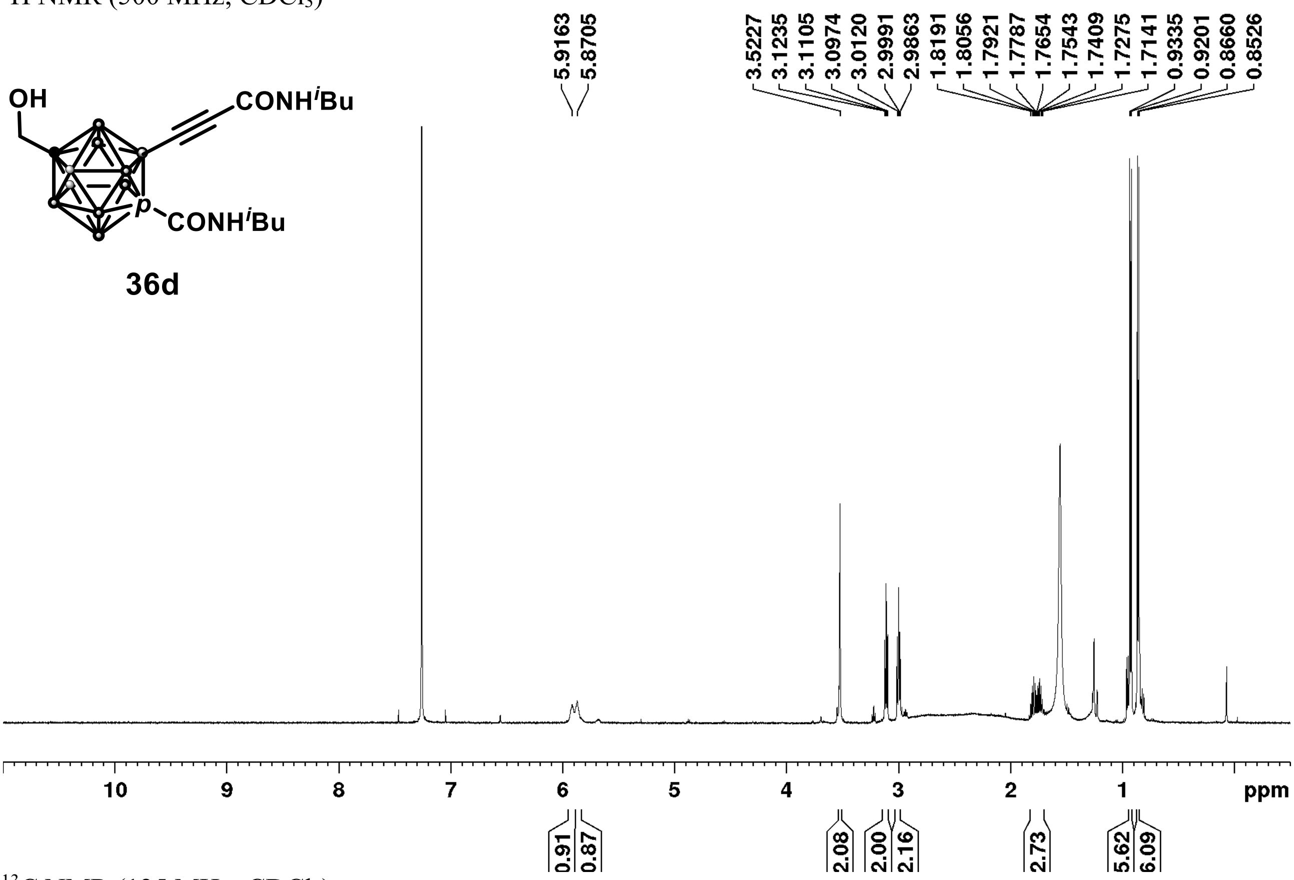

^{13}C NMR (125 MHz; $CDCl_3$)

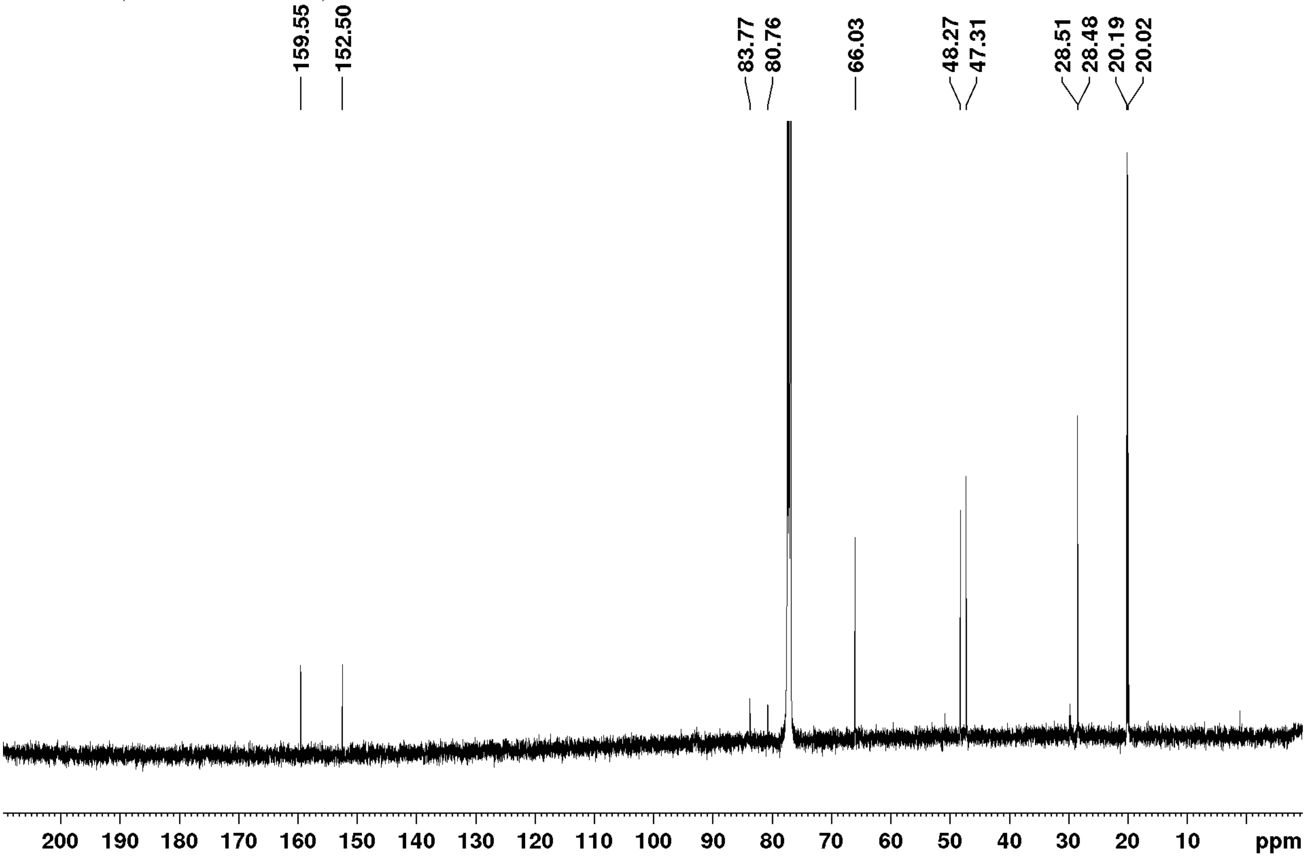

^{11}B NMR (160 MHz; $CDCl_3$)

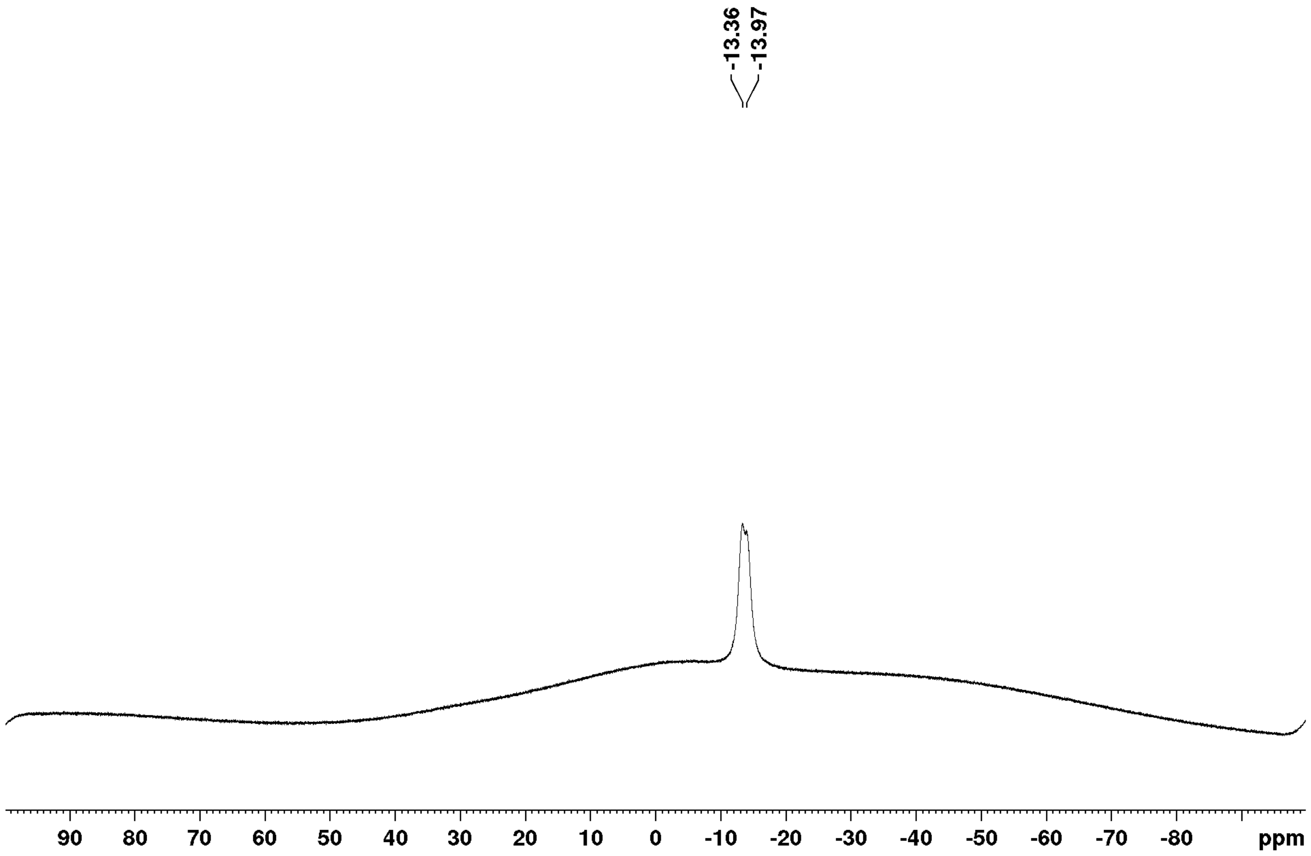

1-phenylacetoxymethyl-2-(3-(benzylamino)-3-oxoprop-1-yn-1-yl)-12-benzylaminocarboxyl-1,12-dicarba-*closo*-dodecaborane (Va)

^{1}H NMR (500 MHz; $CDCl_3$)

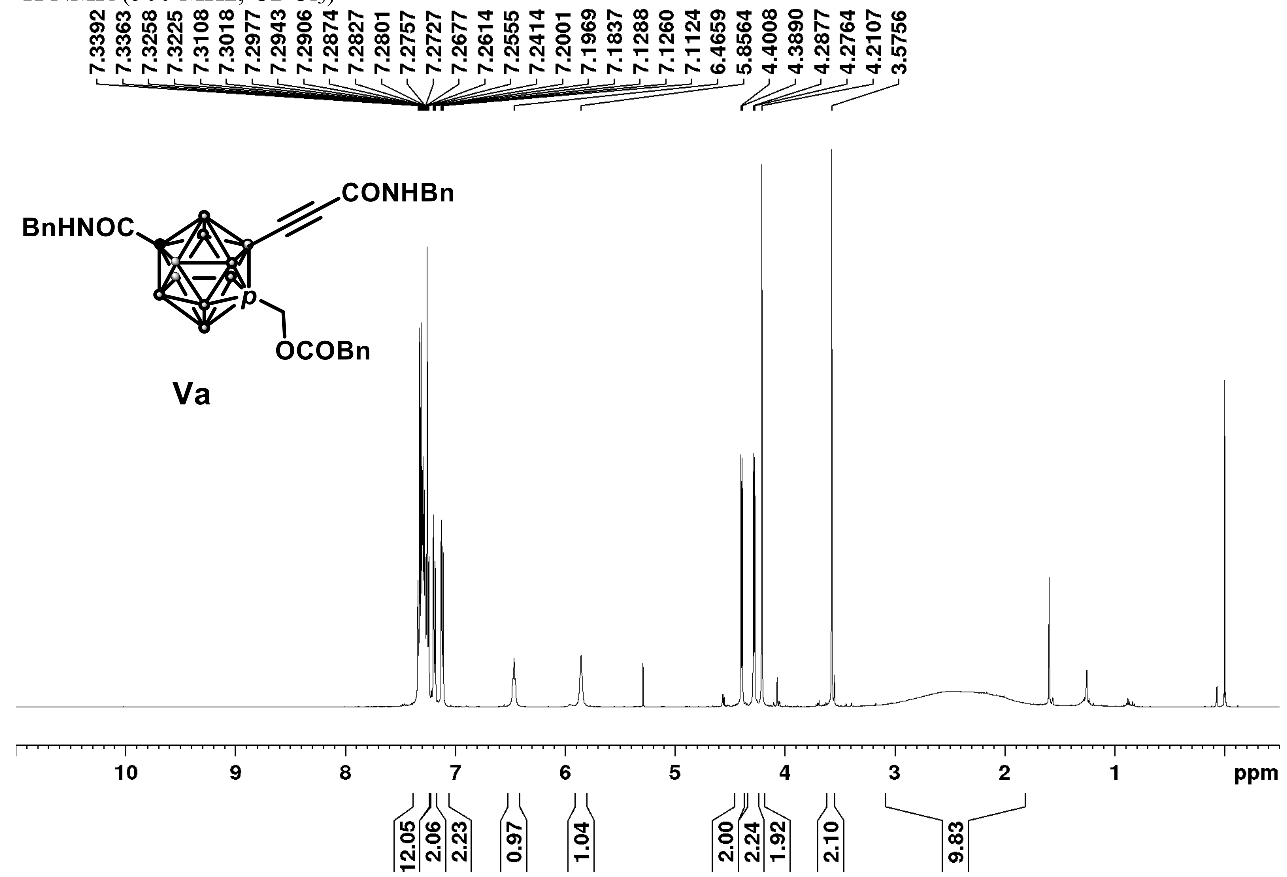

^{13}C NMR (125 MHz; $CDCl_3$)

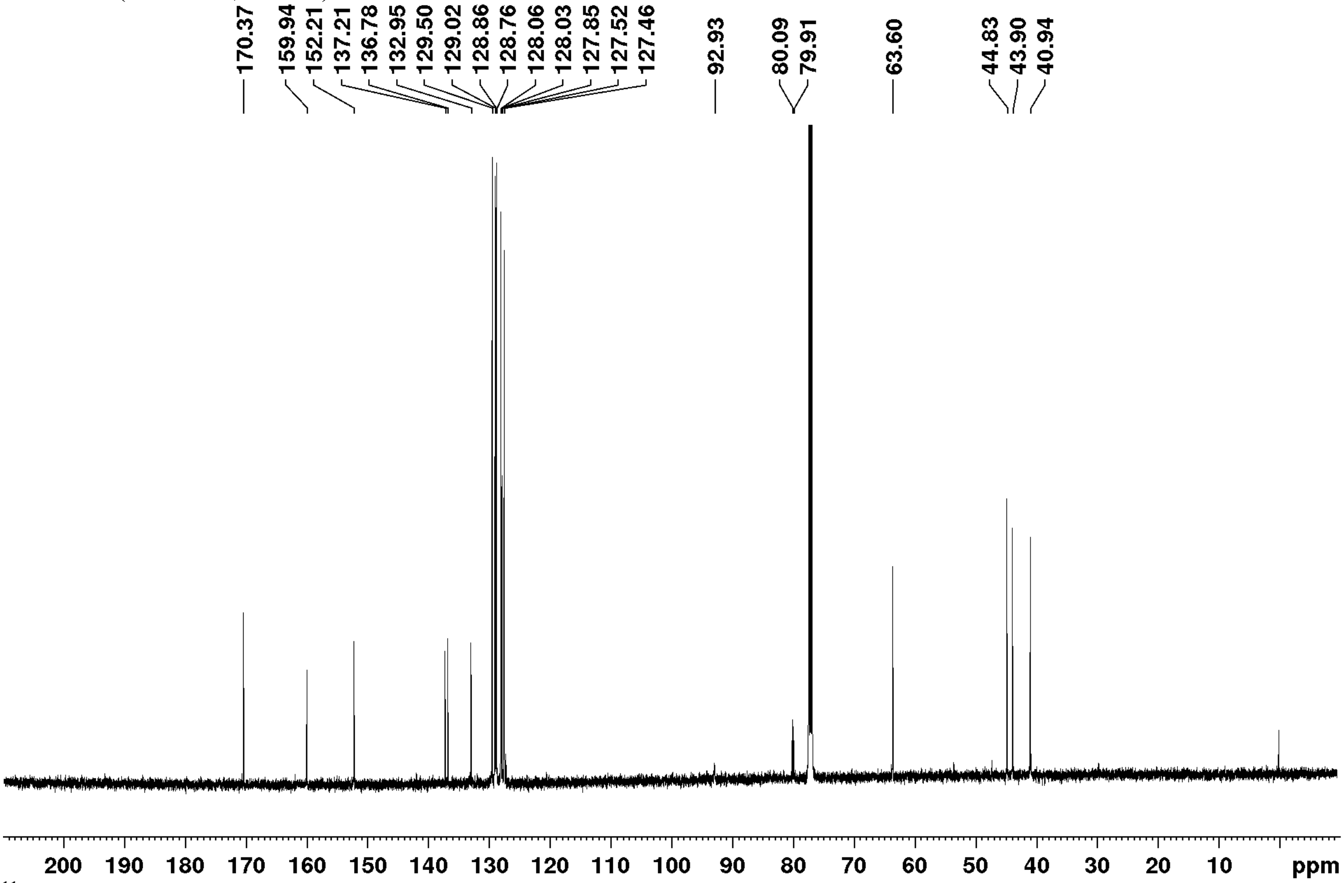

^{11}B NMR (160 MHz; $CDCl_3$)

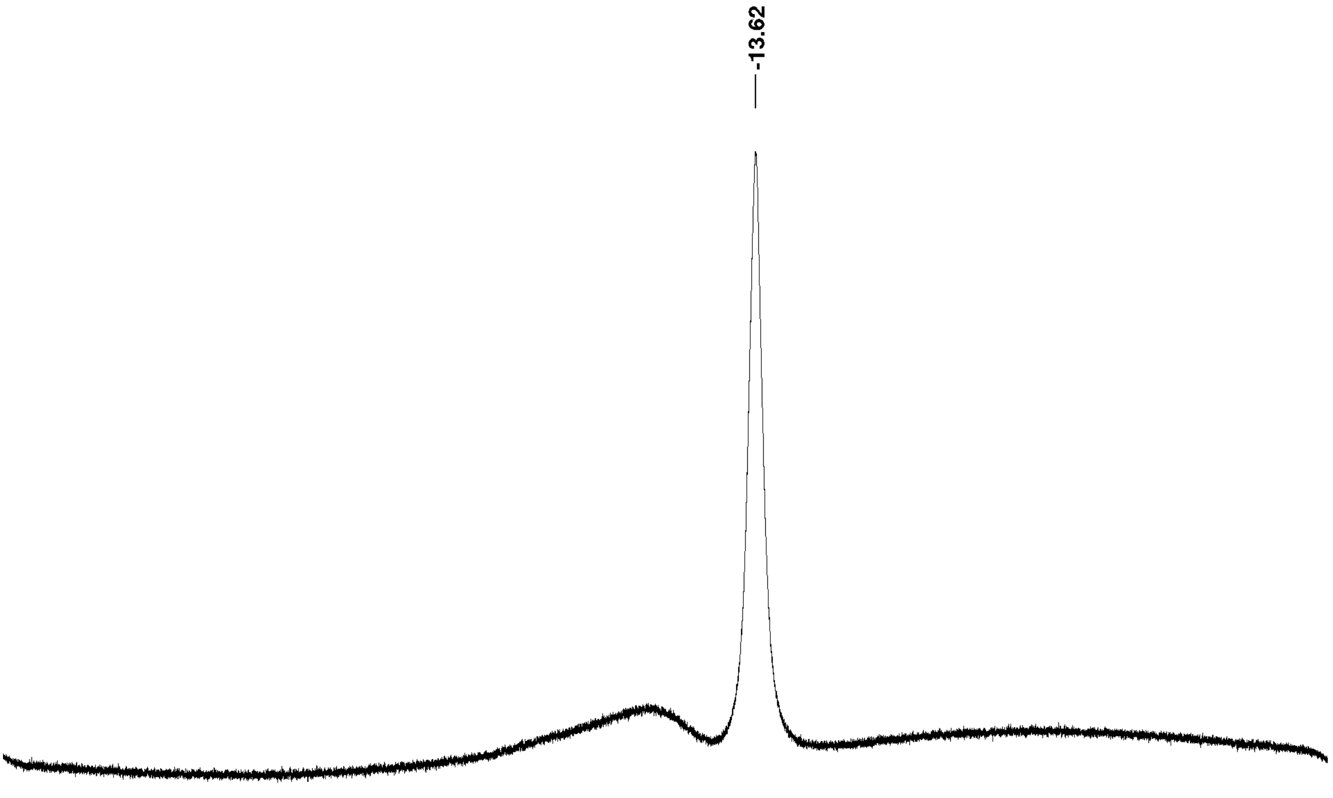

1-((3-methylbutanoyl)oxy)methyl-2-(3-(benzylamino)-3-oxoprop-1-yn-1-yl)-12- benzylaminocarboxyl-1,12-dicarba-*closo*-dodecaborane (Vb)

^{1}H NMR (500 MHz; $CDCl_3$)

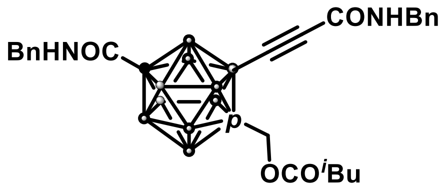

Vb

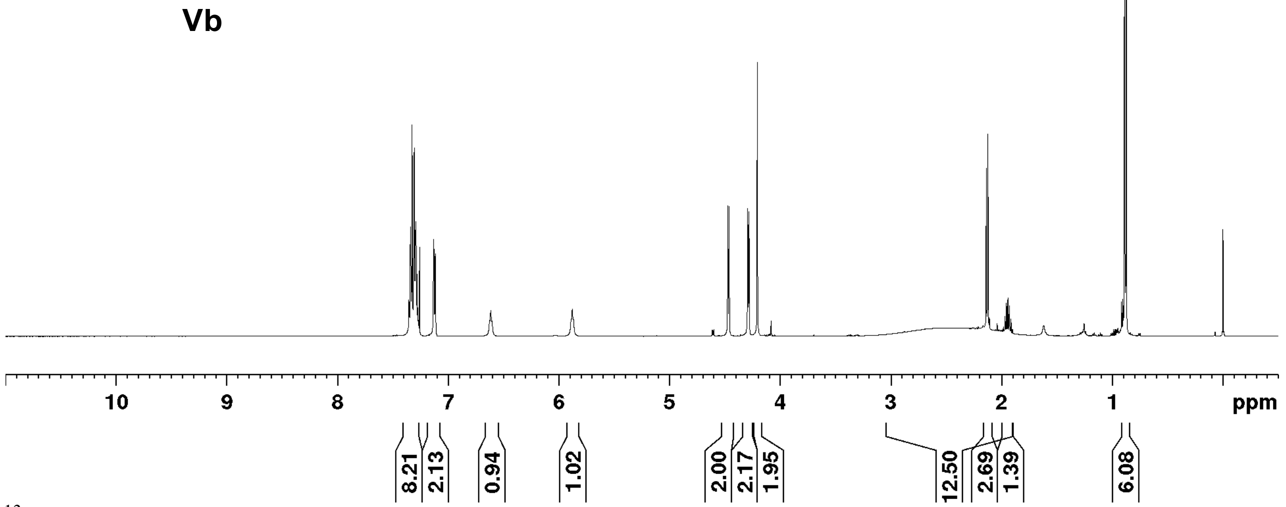

^{13}C NMR (125 MHz; $CDCl_3$)

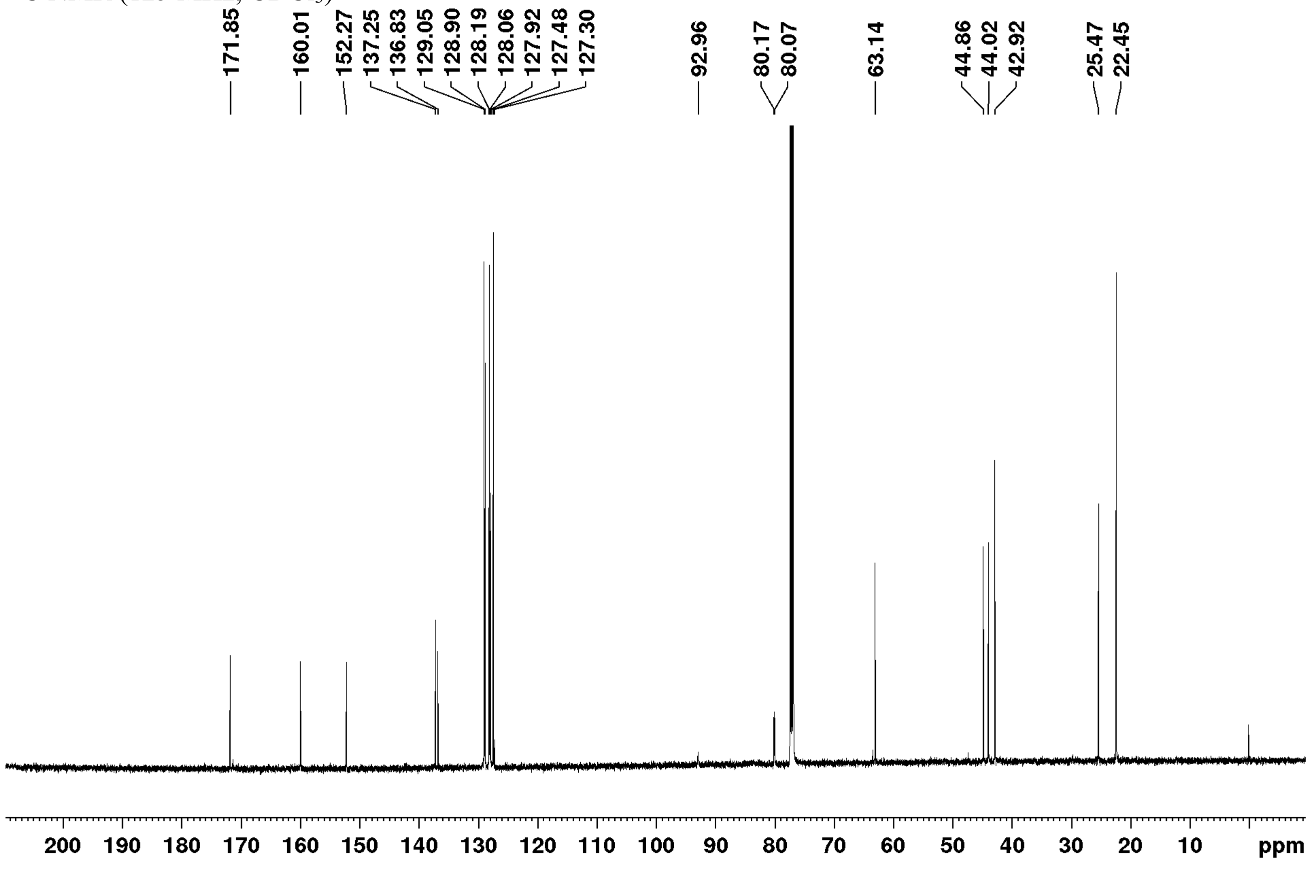

^{11}B NMR (160 MHz; $CDCl_3$)

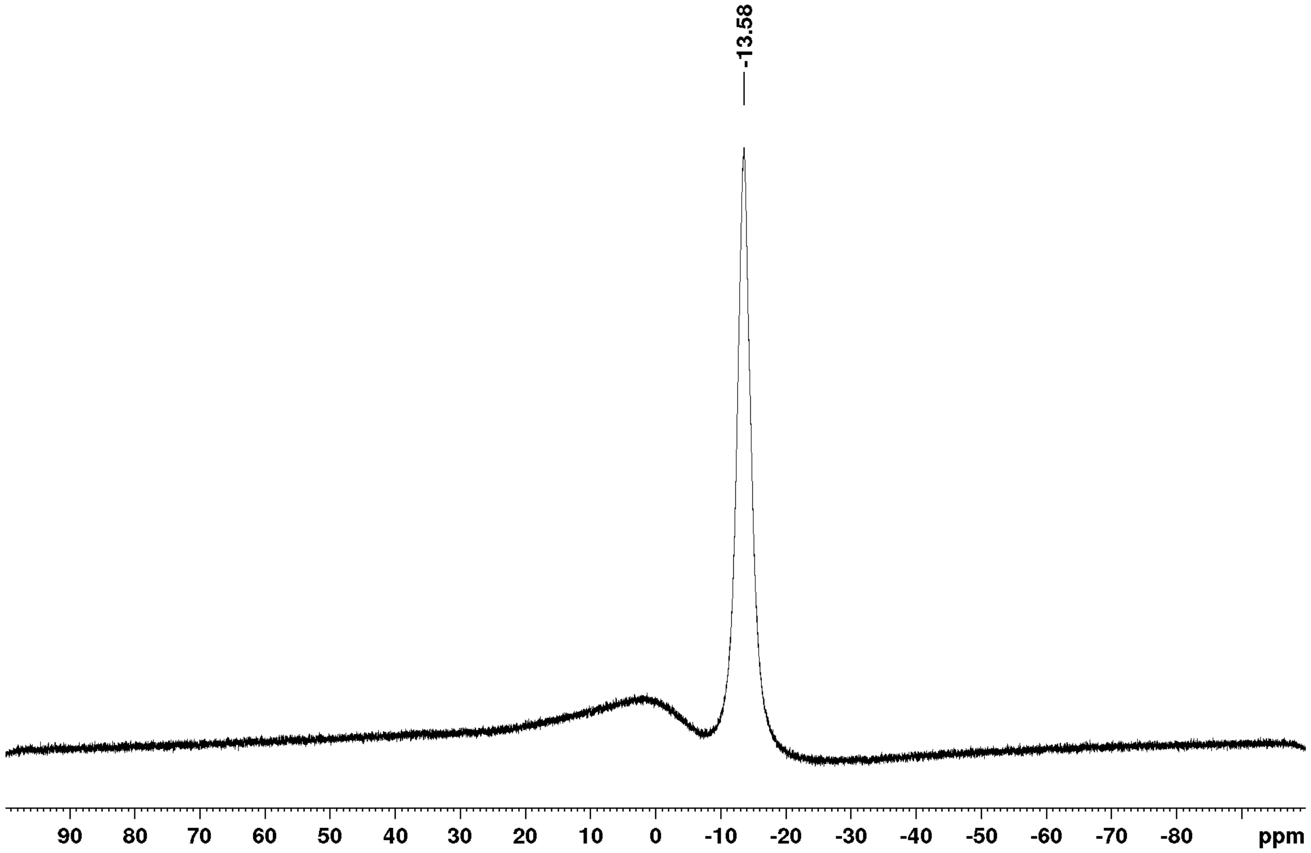

1-phenylacetoxymethyl-2-(3-(isobutylamino)-3-oxoprop-1-yn-1-yl)-12-benzylaminocarboxyl-1,12-dicarba-*closo*-dodecaborane (Vc)

^{1}H NMR (500 MHz; $CDCl_3$)

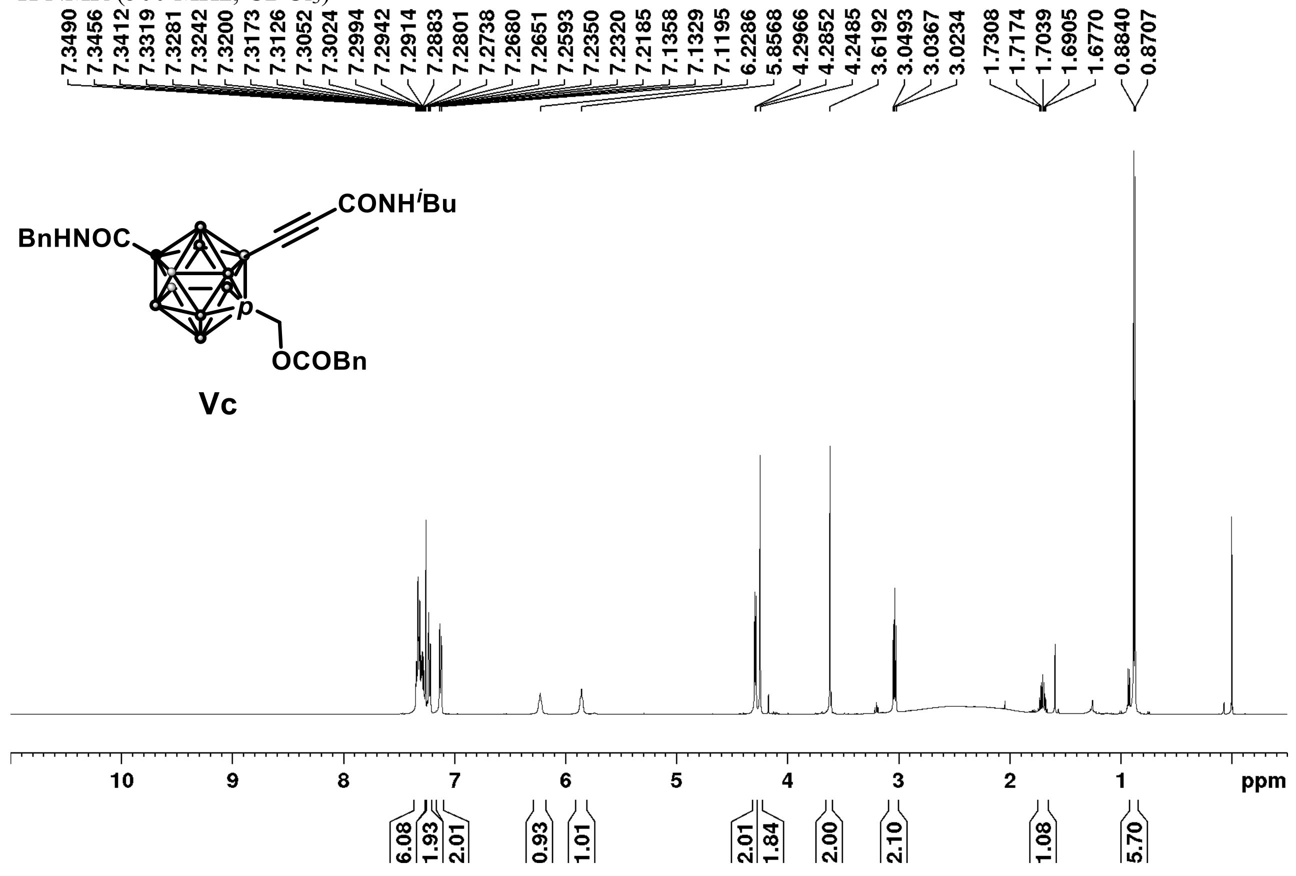

^{13}C NMR (125 MHz; $CDCl_3$)

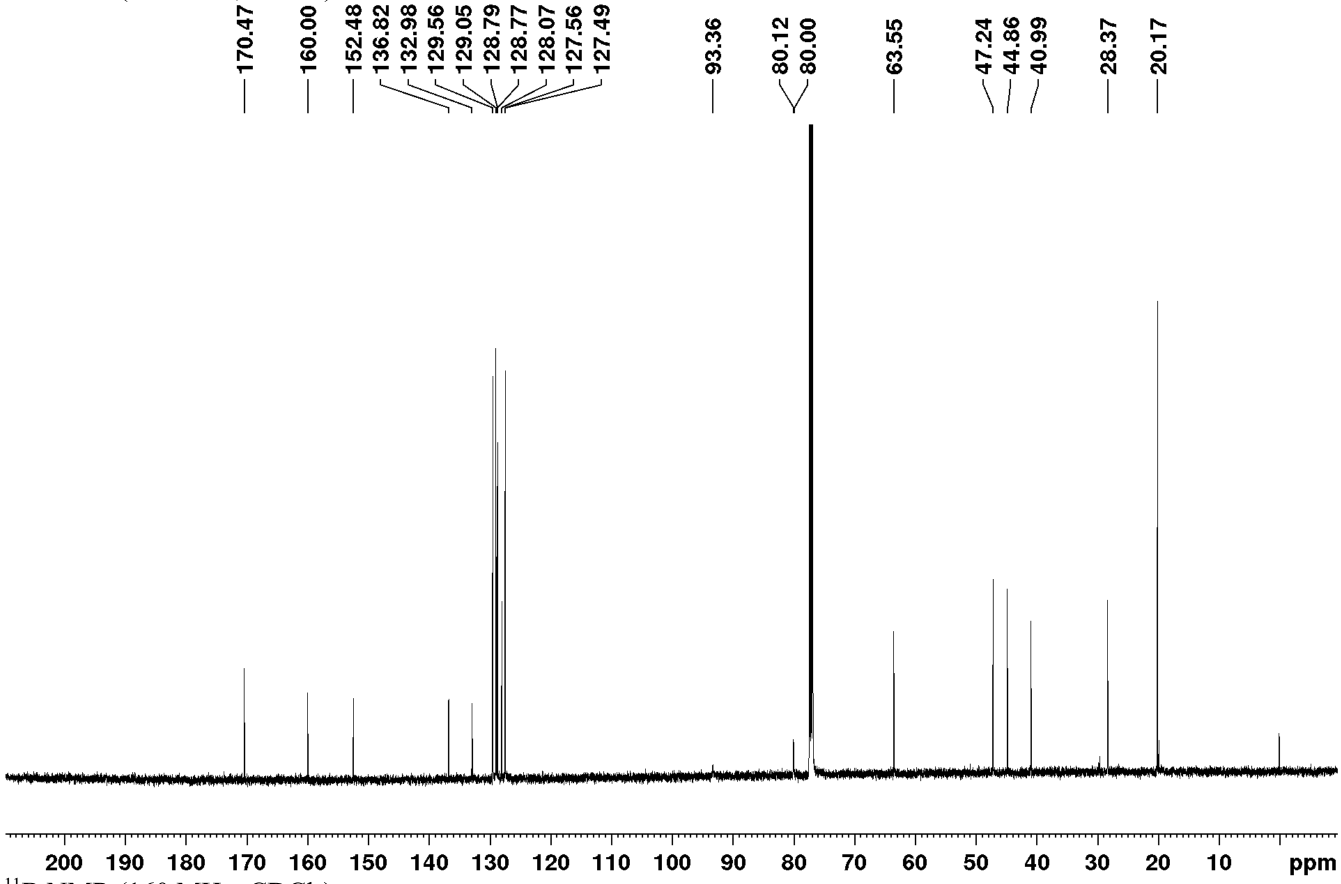

^{11}B NMR (160 MHz; $CDCl_3$)

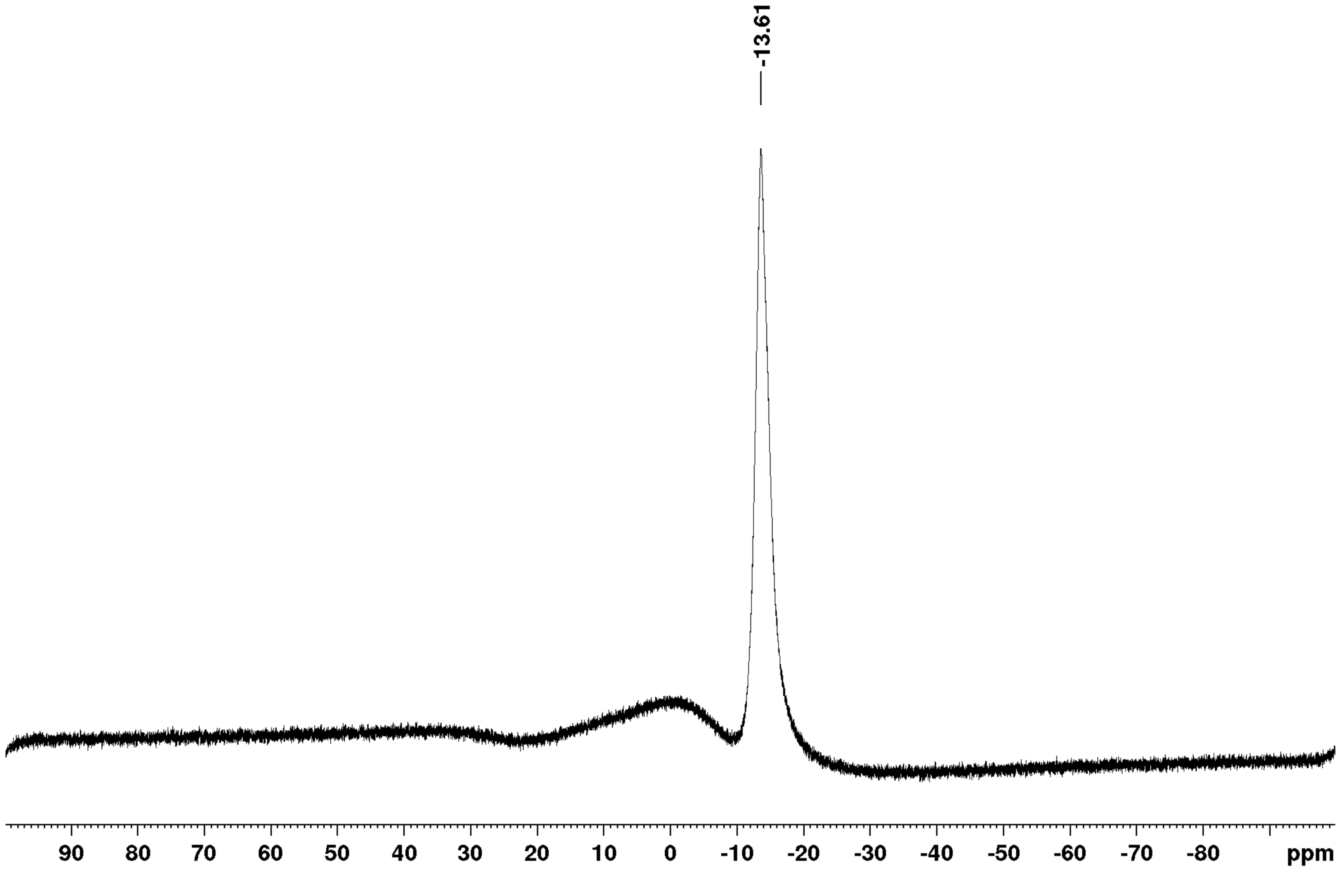

1-((3-methylbutanoyl)oxy)methyl-2-(3-(isobutylamino)-3-oxoprop-1-yn-1-yl)-12- benzylaminocarboxyl-1,12-dicarba-*closo*-dodecaborane (Vd)

^{1}H NMR (500 MHz; $CDCl_3$)

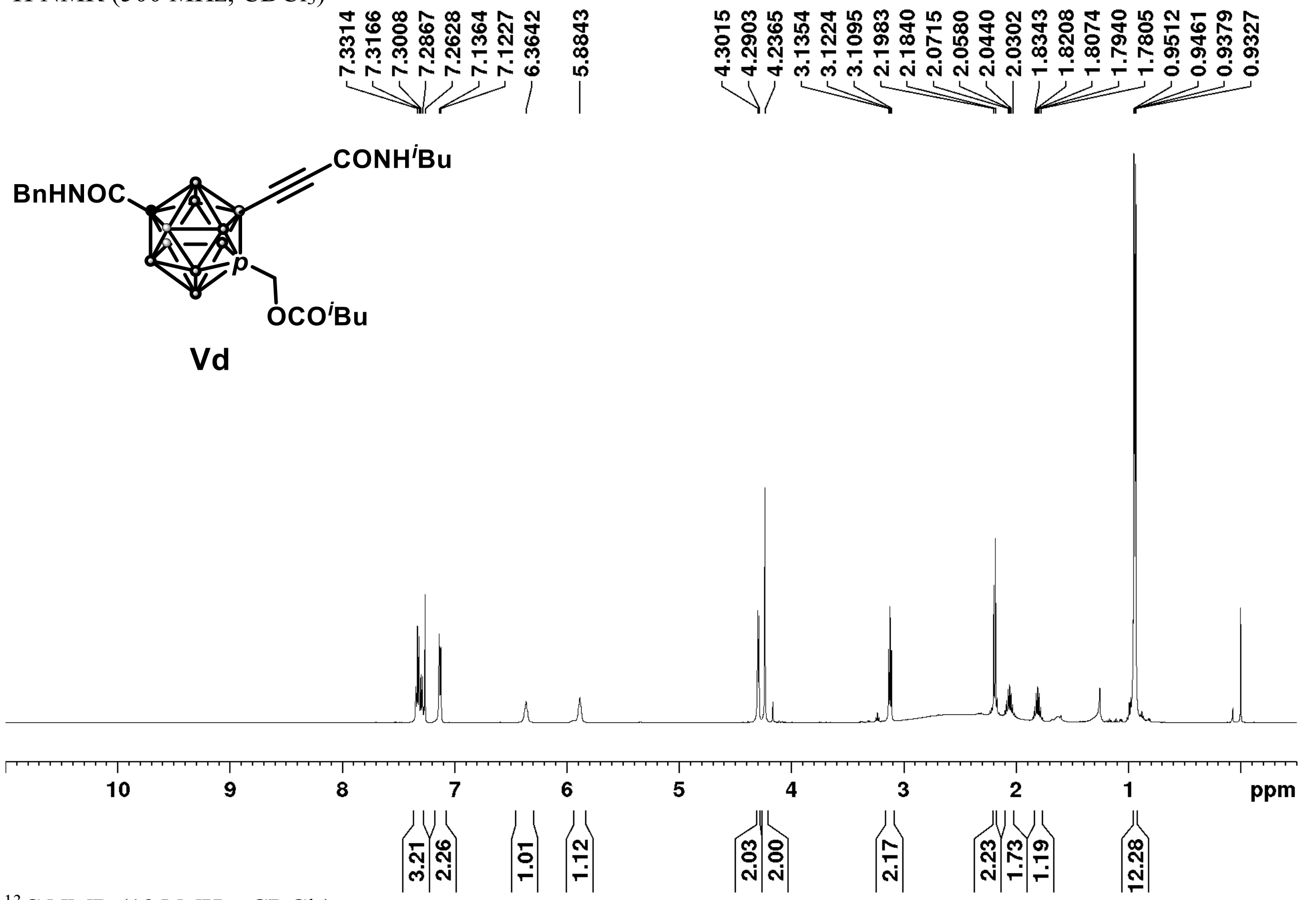

^{13}C NMR (125 MHz; $CDCl_3$)

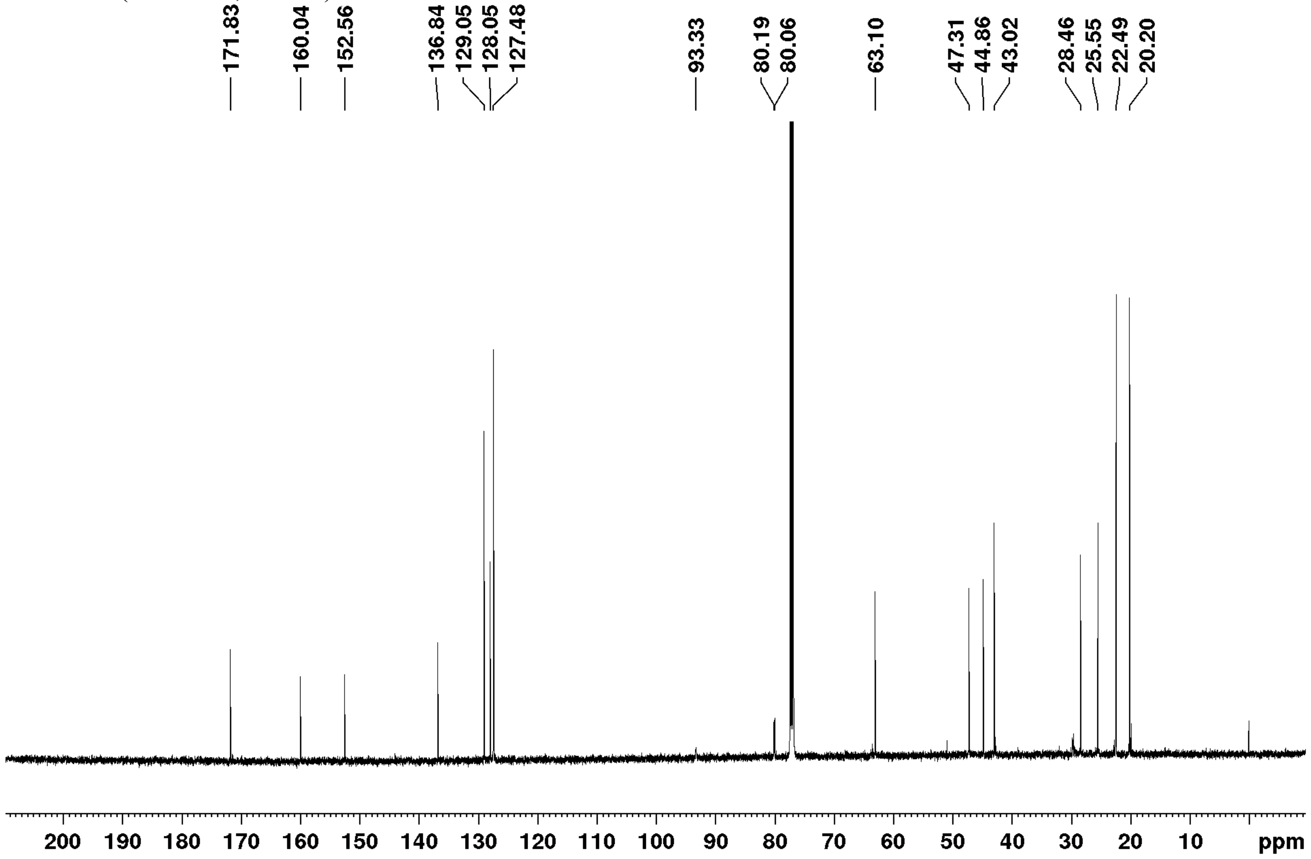

^{11}B NMR (160 MHz; $CDCl_3$)

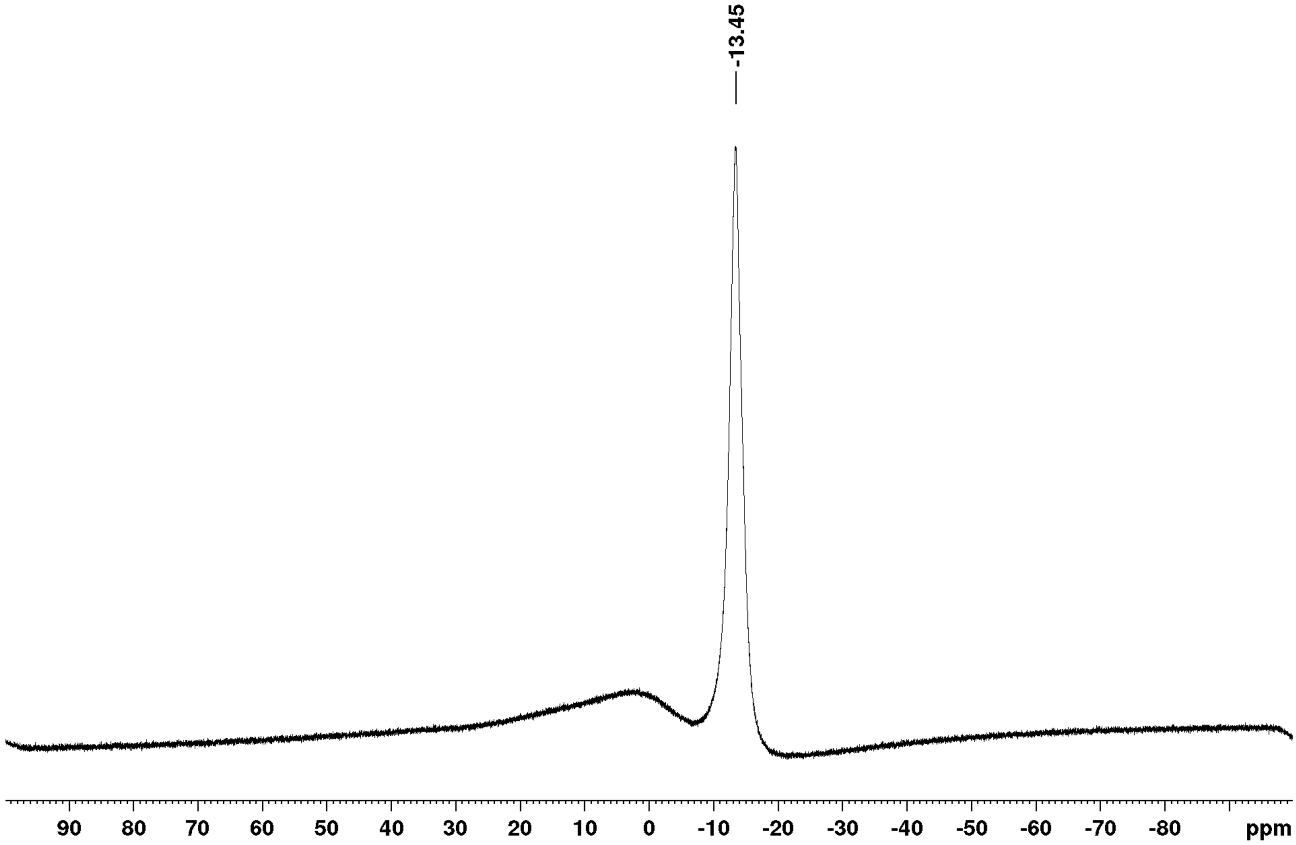

Synthesis of 1-phenylacetoxymethyl-2-(3-(benzylamino)-3-oxoprop-1-yn-1-yl)-12-isobutylcarbamoyl-1,12-dicarba-*closo*-dodecaborane (Ve)

^{1}H NMR (500 MHz; $CDCl_3$)

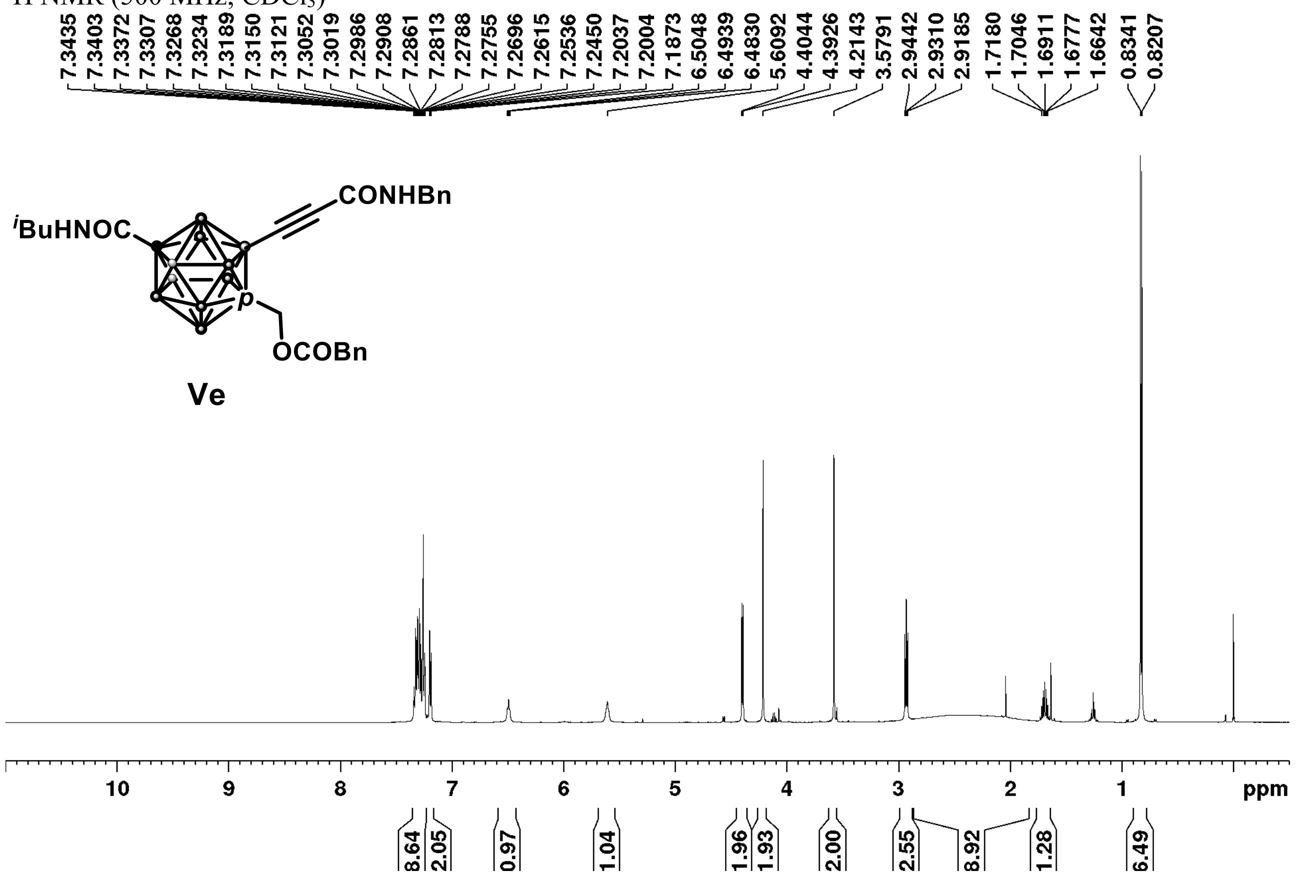

¹³C NMR (125 MHz; $CDCl_3$)

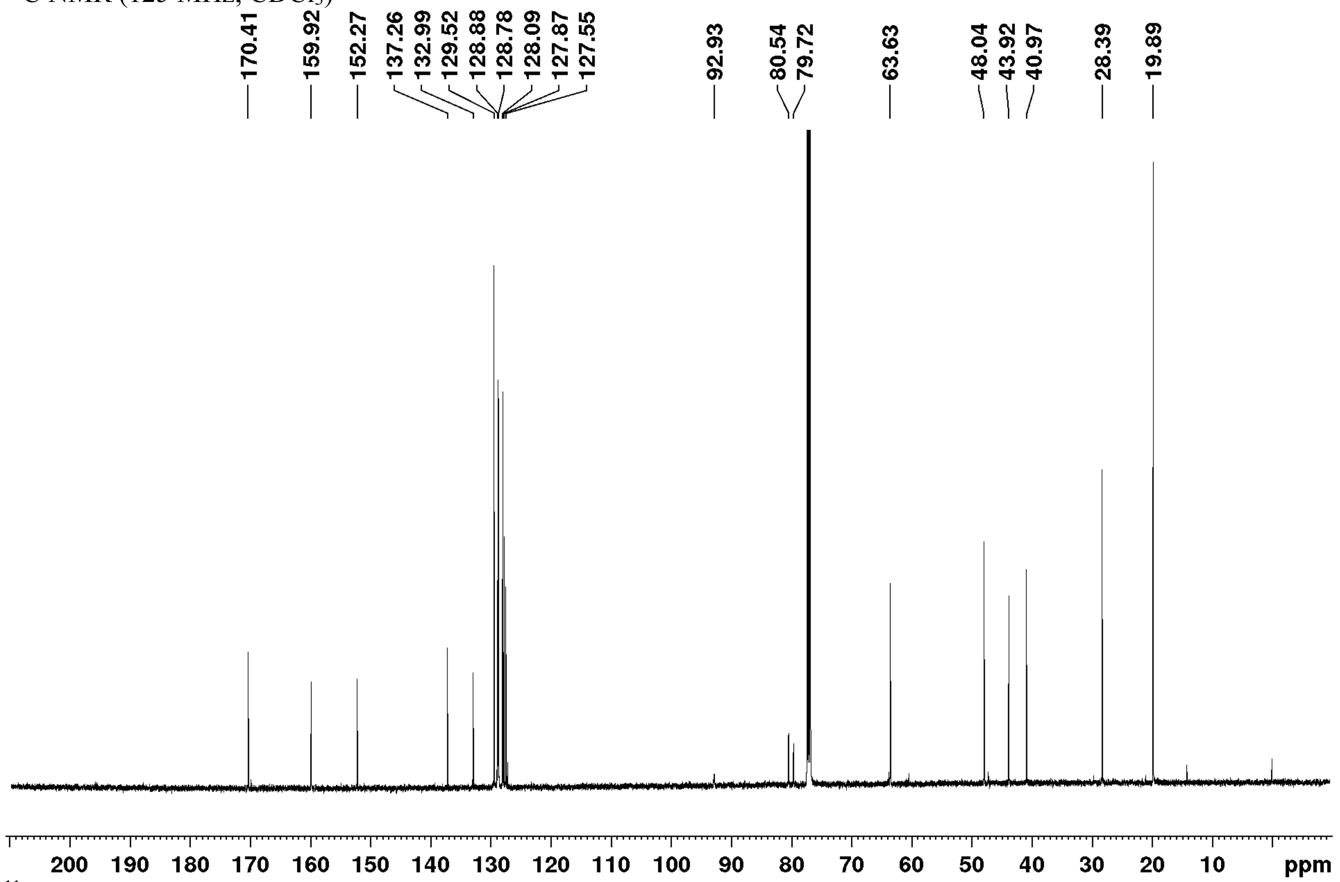

¹¹B NMR (160 MHz; $CDCl_3$)

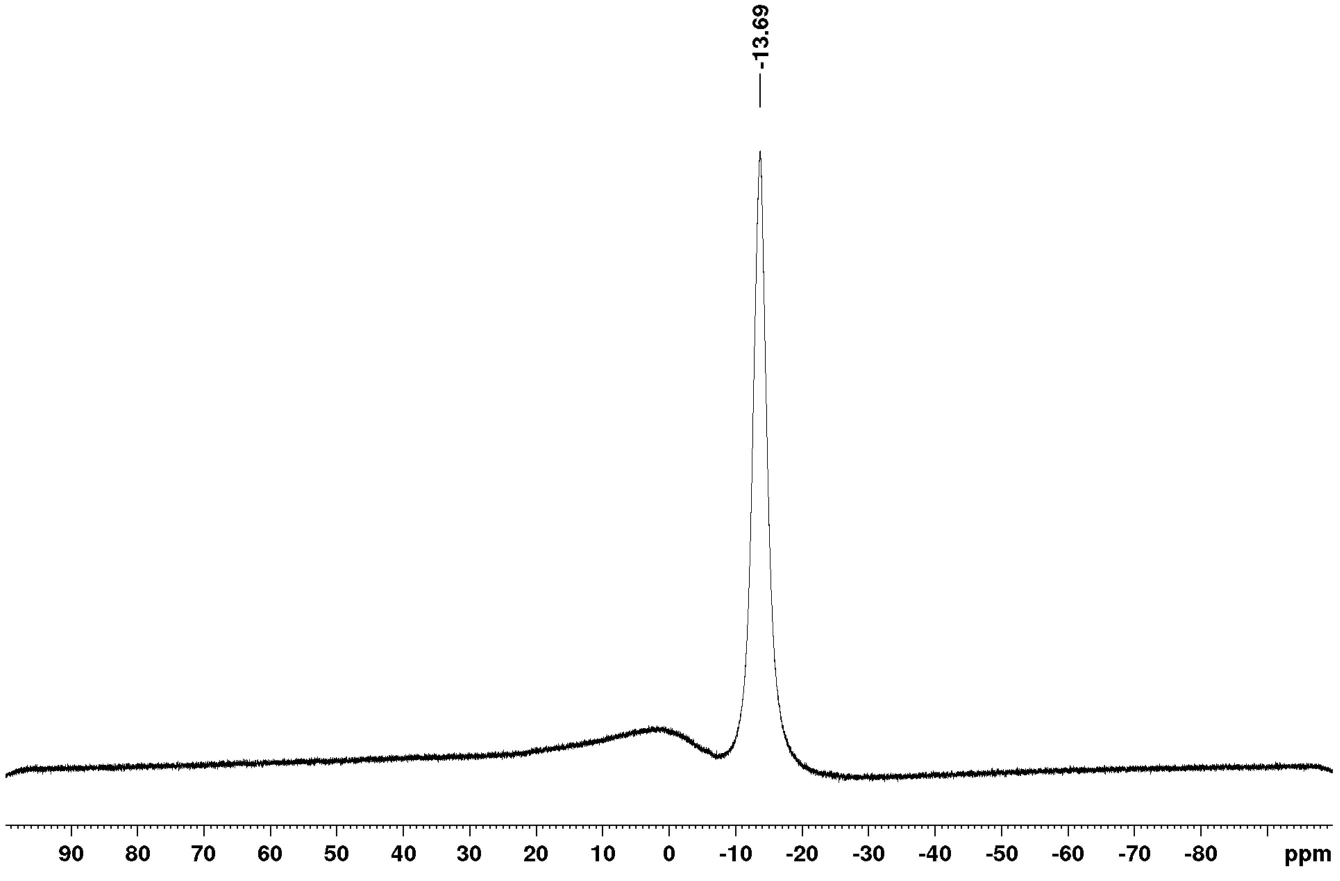

1-((3-methylbutanoyl)oxy)methyl-2-(3-(benzylamino)-3-oxoprop-1-yn-1-yl)-12-isobutylcarbamoyl-1,12-dicarba-*closo*-dodecaborane (Vf)

^{1}H NMR (500 MHz; $CDCl_3$)

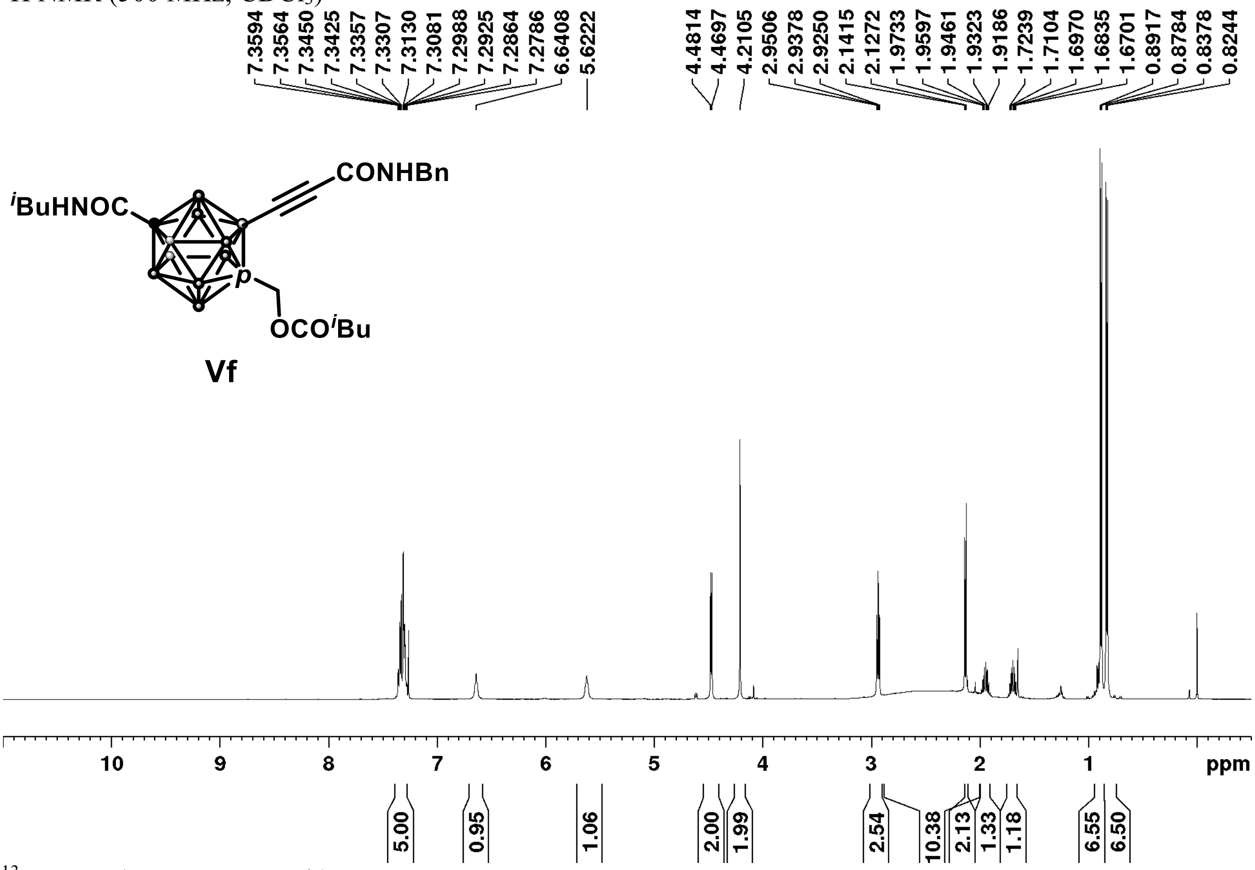

^{13}C NMR (125 MHz; $CDCl_3$)

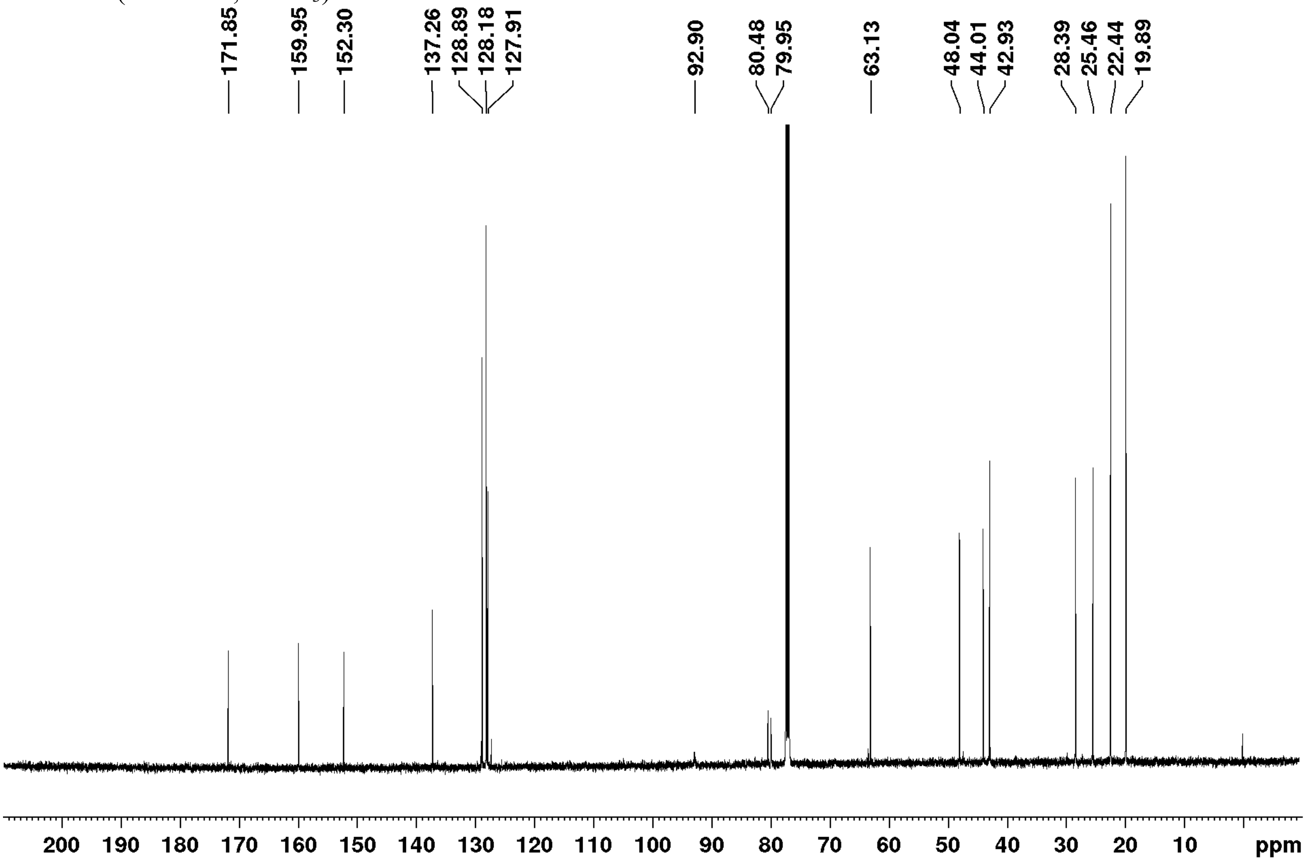

^{11}B NMR (160 MHz; $CDCl_3$)

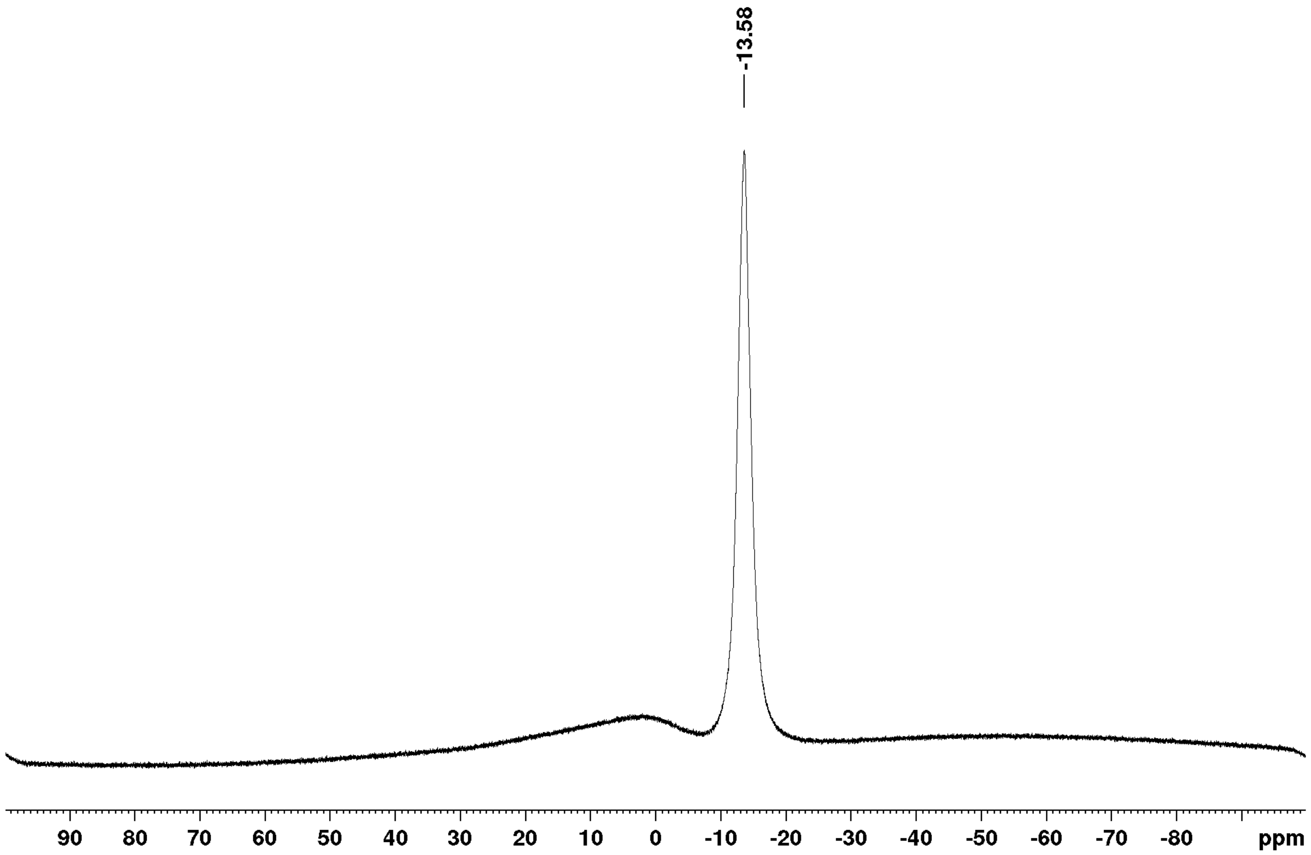

1-phenylacetoxymethyl-2-(3-(isobutylamino)-3-oxoprop-1-yn-1-yl)-12-isobutylcarbamoyl-1,12-dicarba-*closo*-dodecaborane (Vg)

^{1}H NMR (500 MHz; $CDCl_3$)

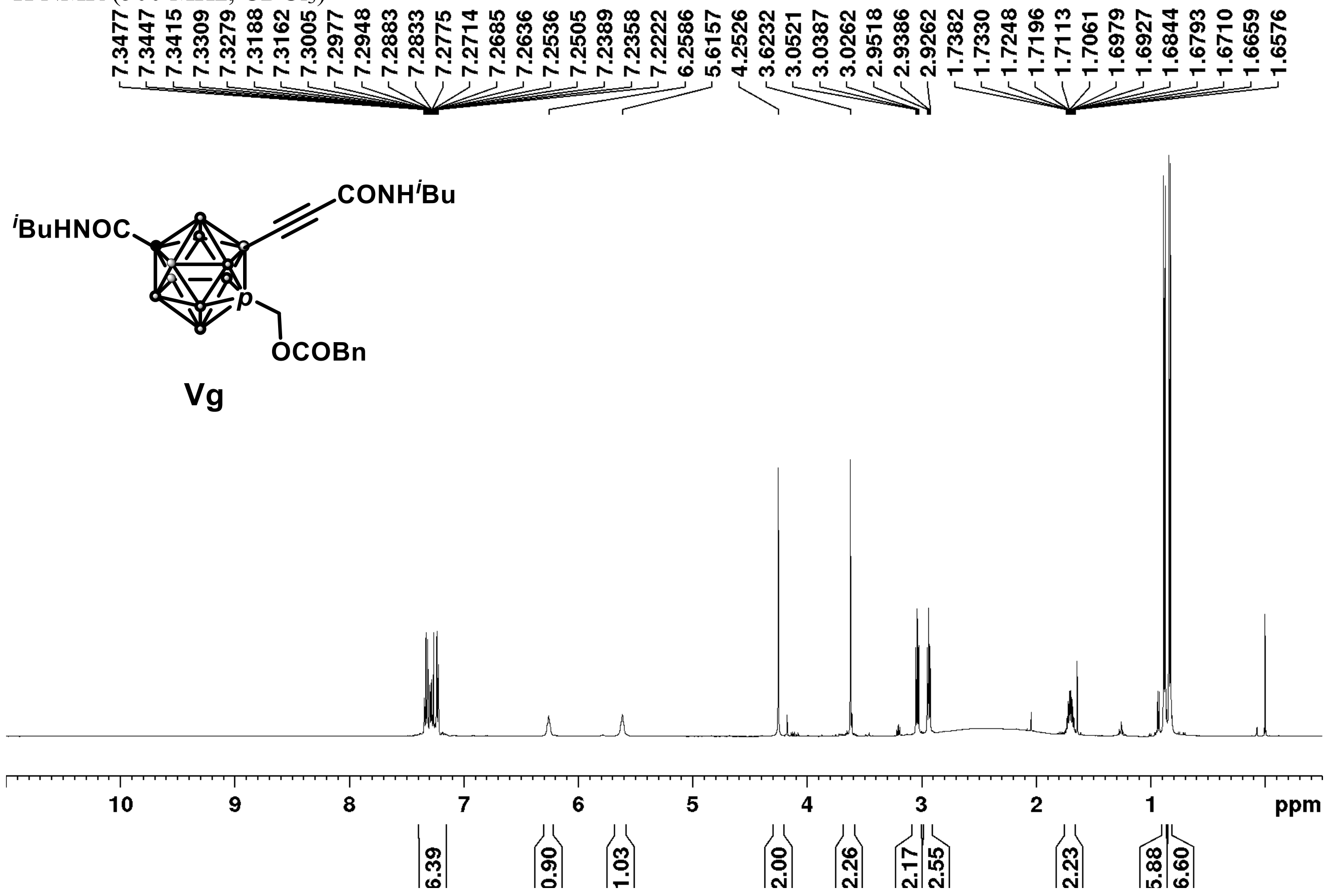

^{13}C NMR (125 MHz; $CDCl_3$)

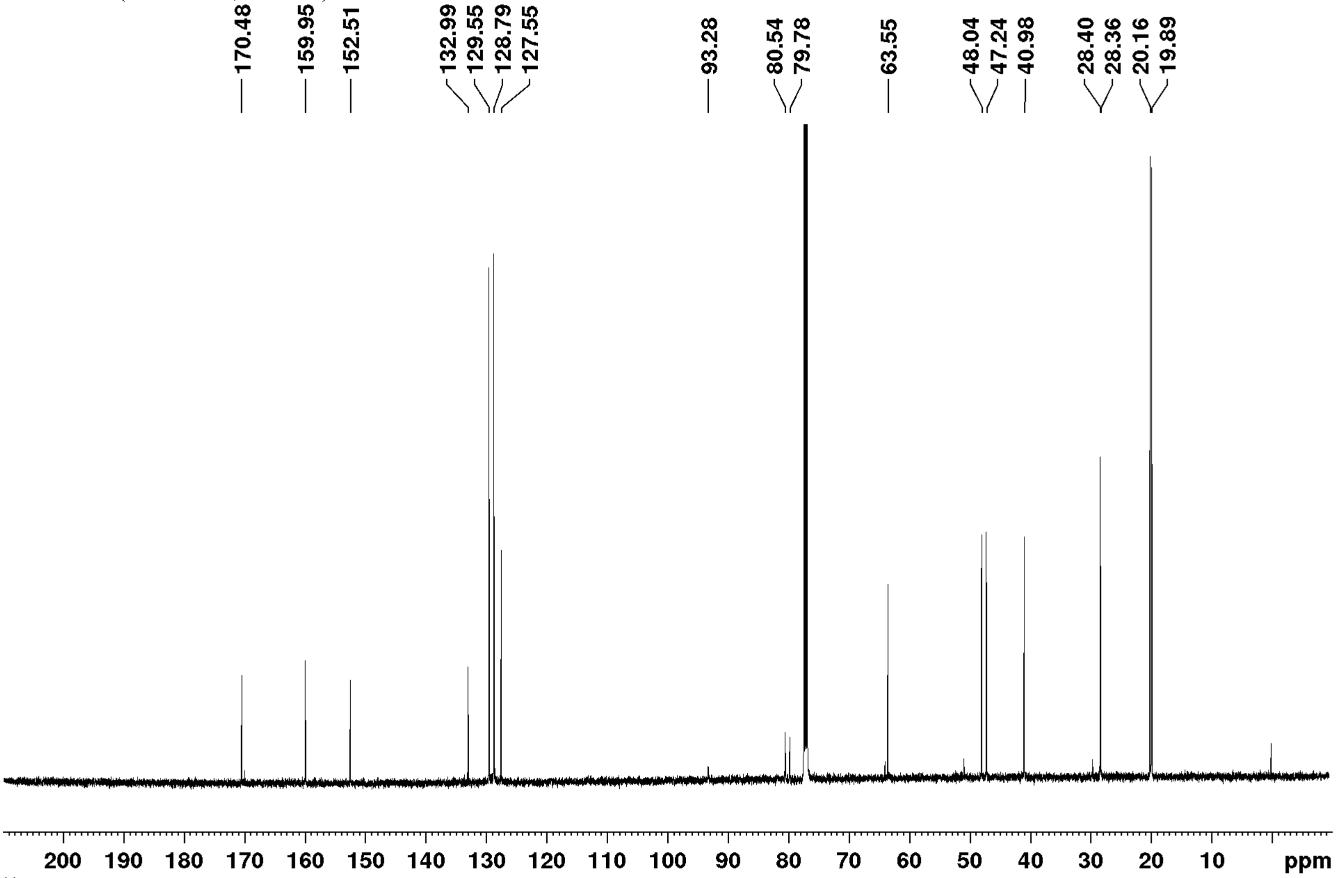

^{11}B NMR (160 MHz; $CDCl_3$)

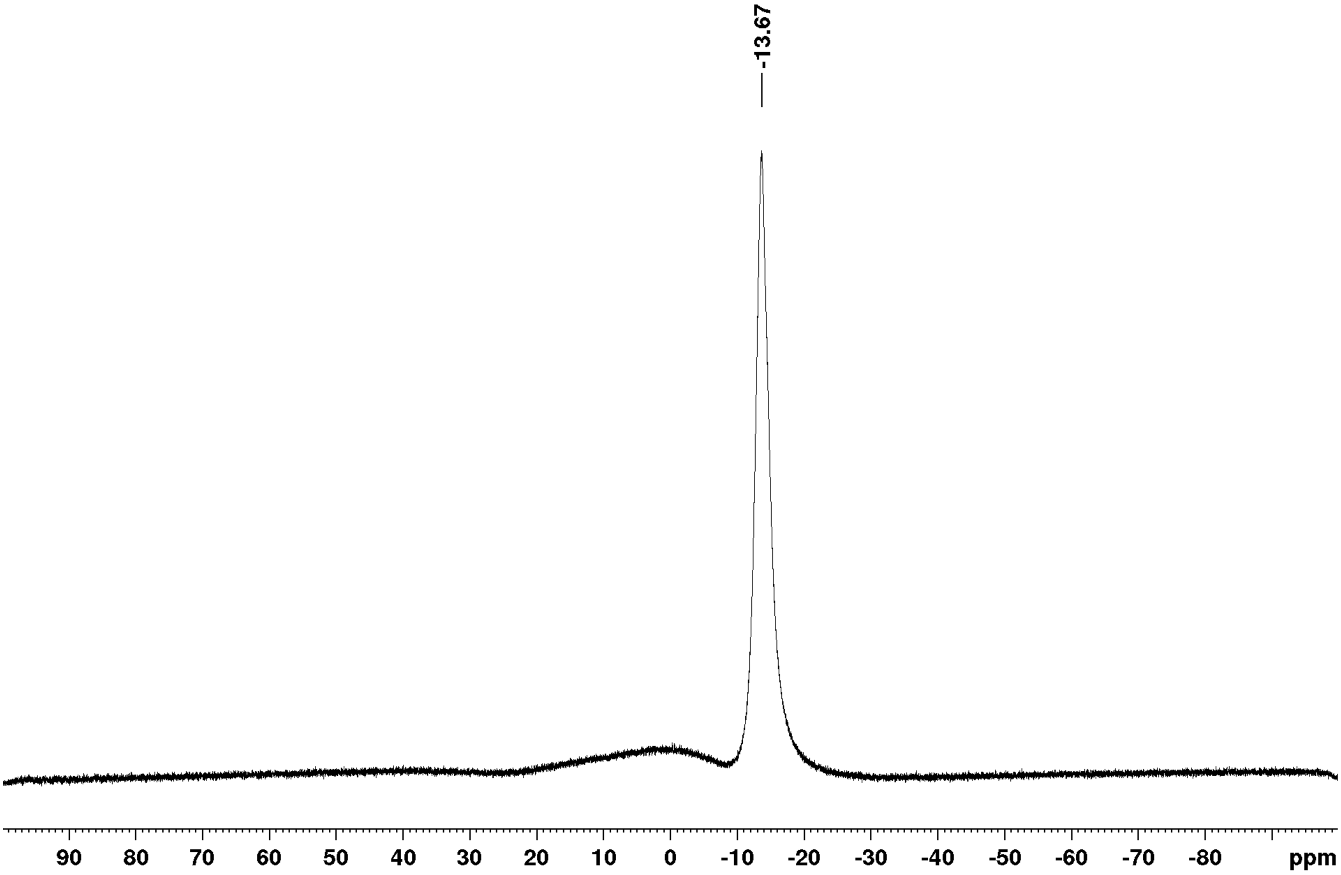

((3-methylbutanoyl)oxy)methyl-2-(3-(isobutylamino)-3-oxoprop-1-yn-1-yl)-12-isobutylcarbamoyl-1,12-dicarba-*closo*-dodecaborane (Vh)

^{1}H NMR (500 MHz; $CDCl_3$)

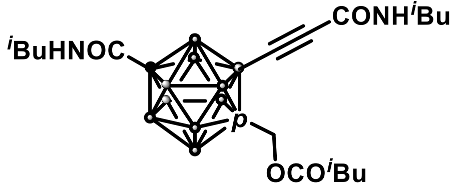

Vh

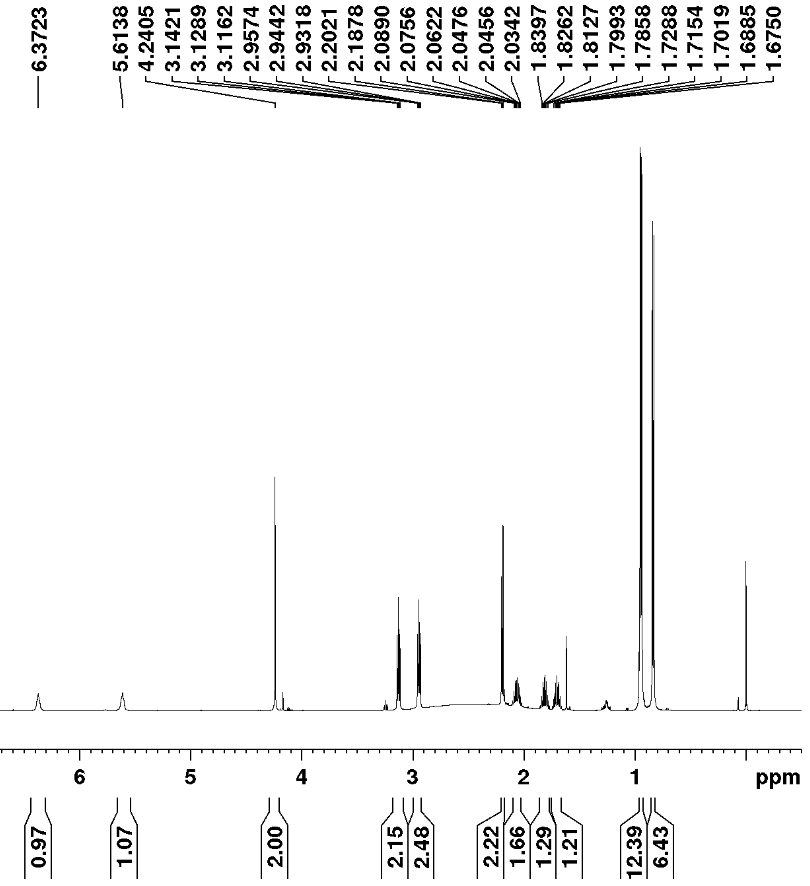

^{13}C NMR (125 MHz; $CDCl_3$)

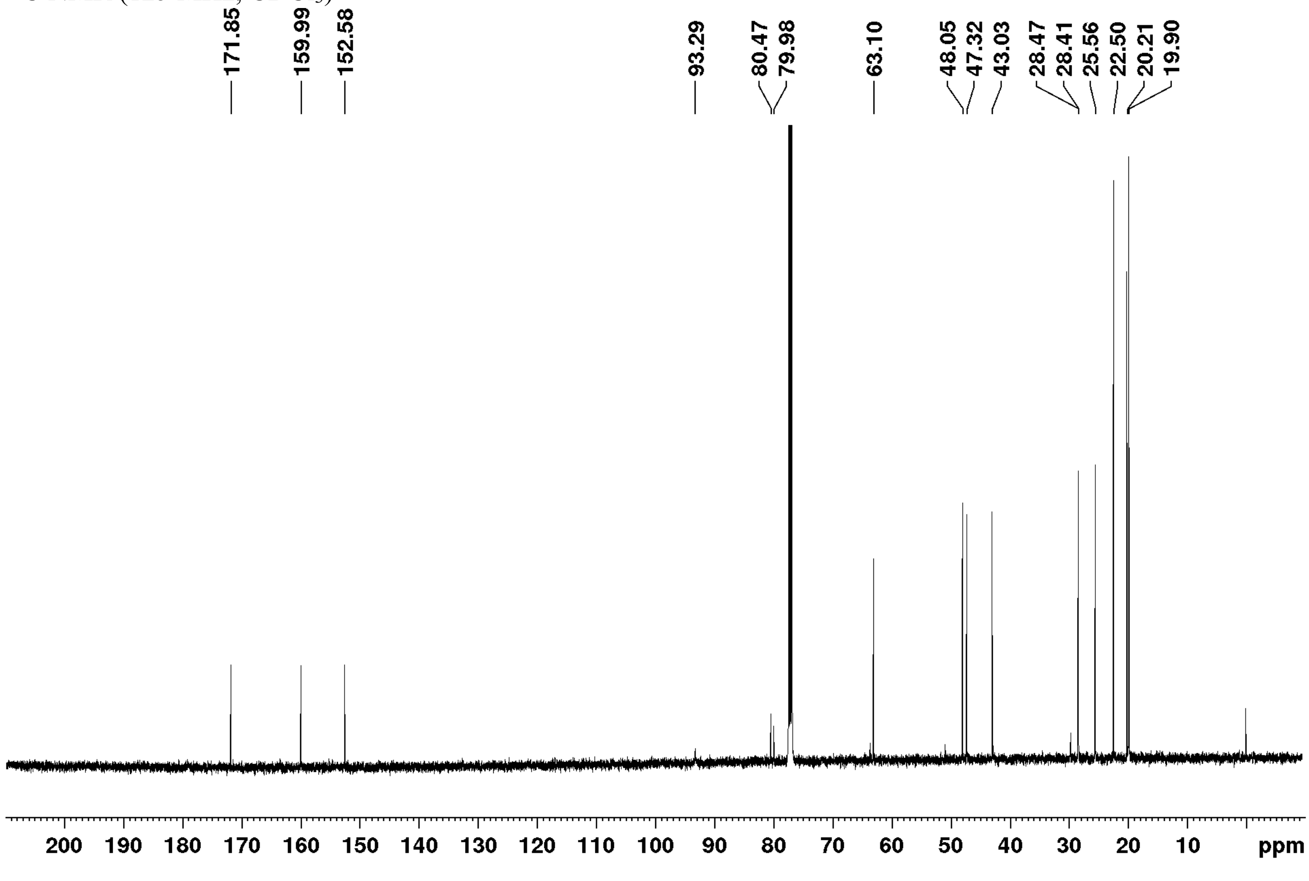

^{11}B NMR (160 MHz; $CDCl_3$)

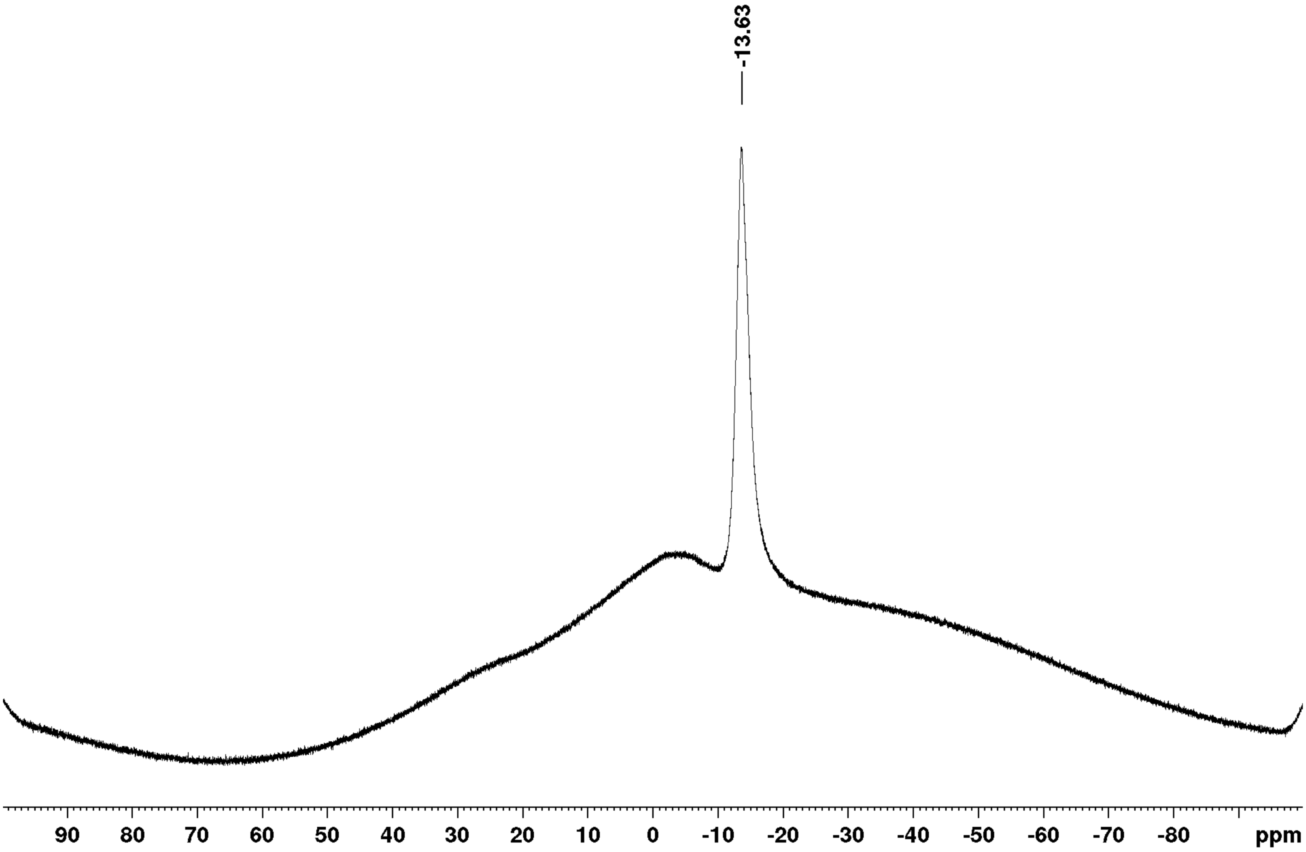

12-phenylacetoxymethyl-2-(3-(benzylamino)-3-oxoprop-1-yn-1-yl)-1-benzylaminocarboxyl-1,12-dicarba-*closo*-dodecaborane (Vi)

^{1}H NMR (500 MHz; $CDCl_3$)

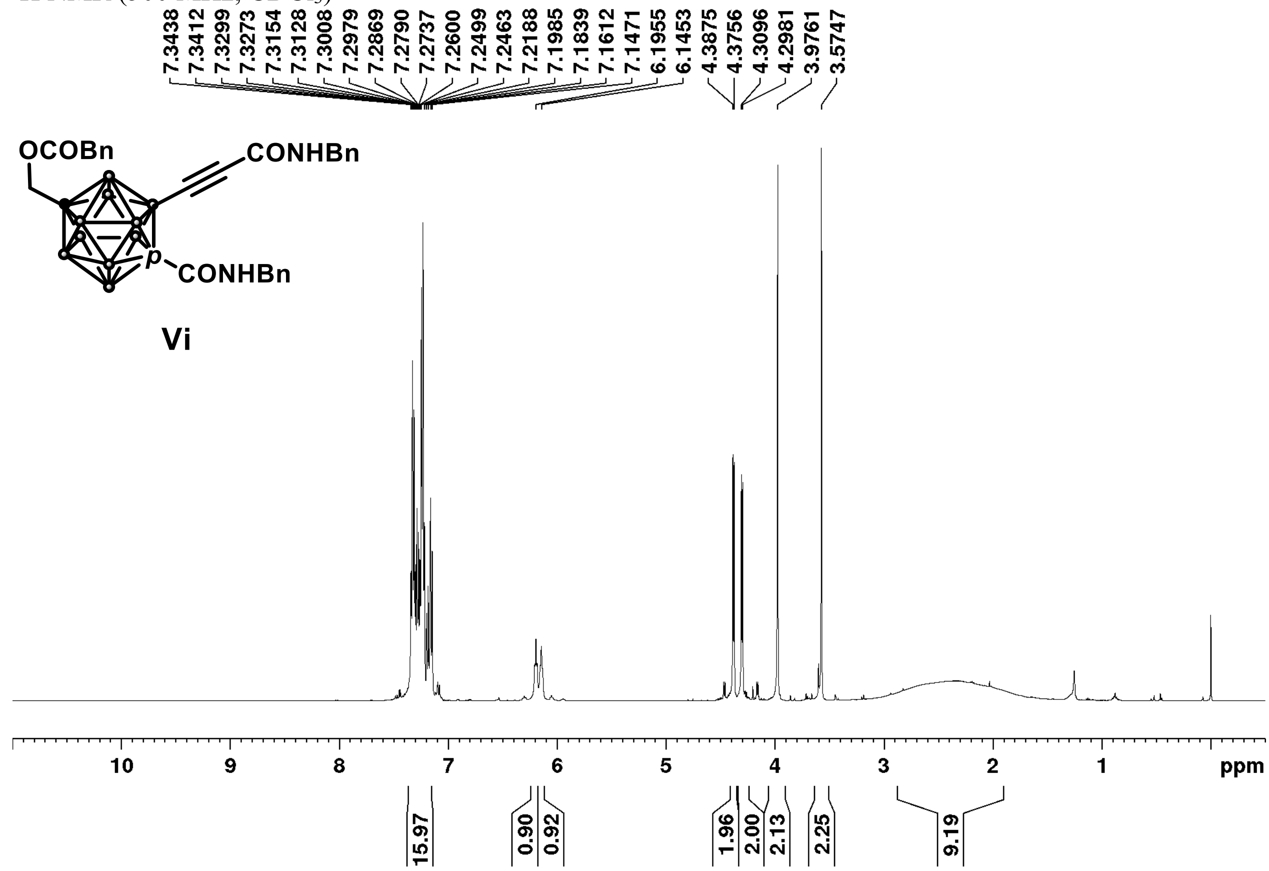

^{13}C NMR (125 MHz; $CDCl_3$)

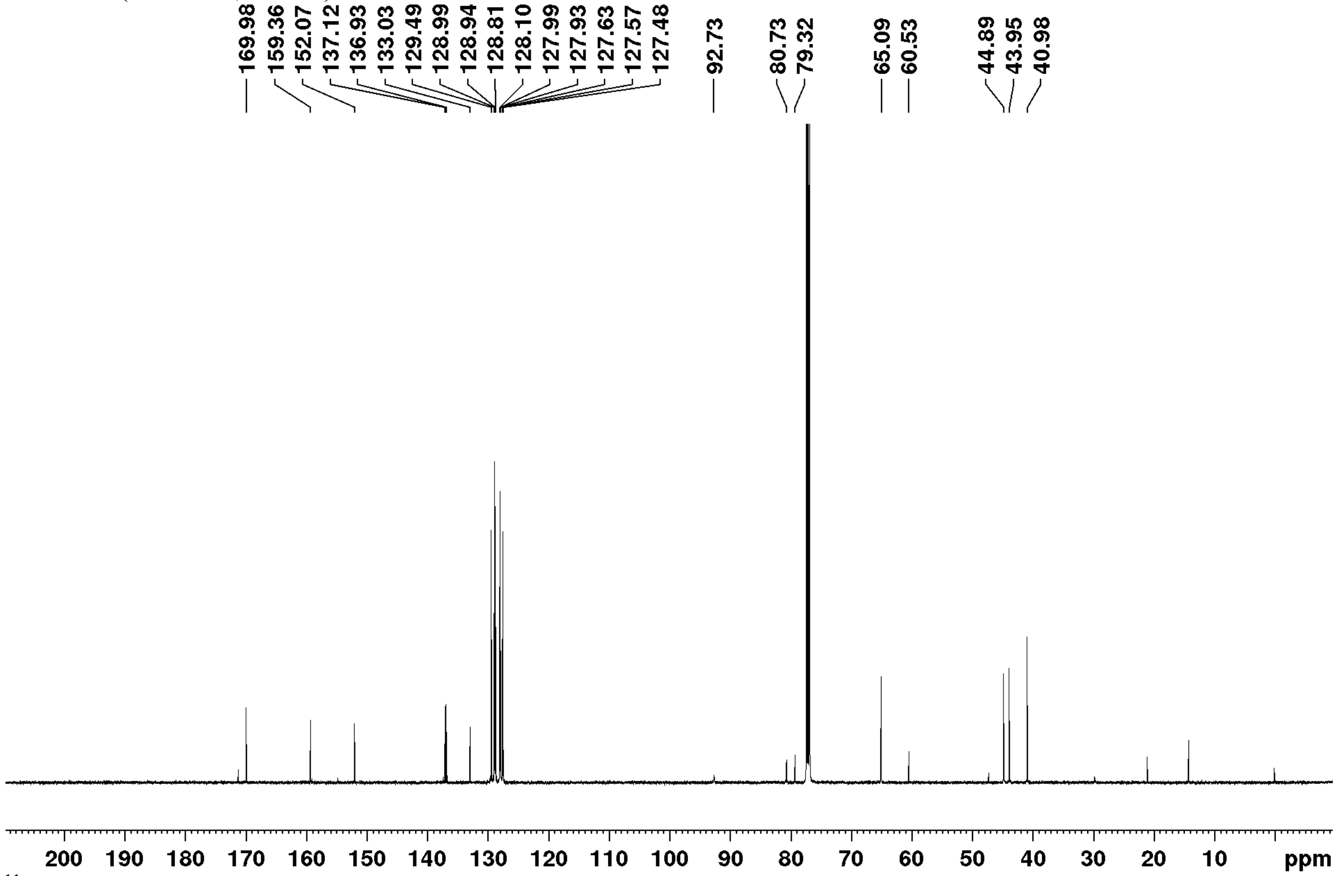

^{11}B NMR (160 MHz; $CDCl_3$)

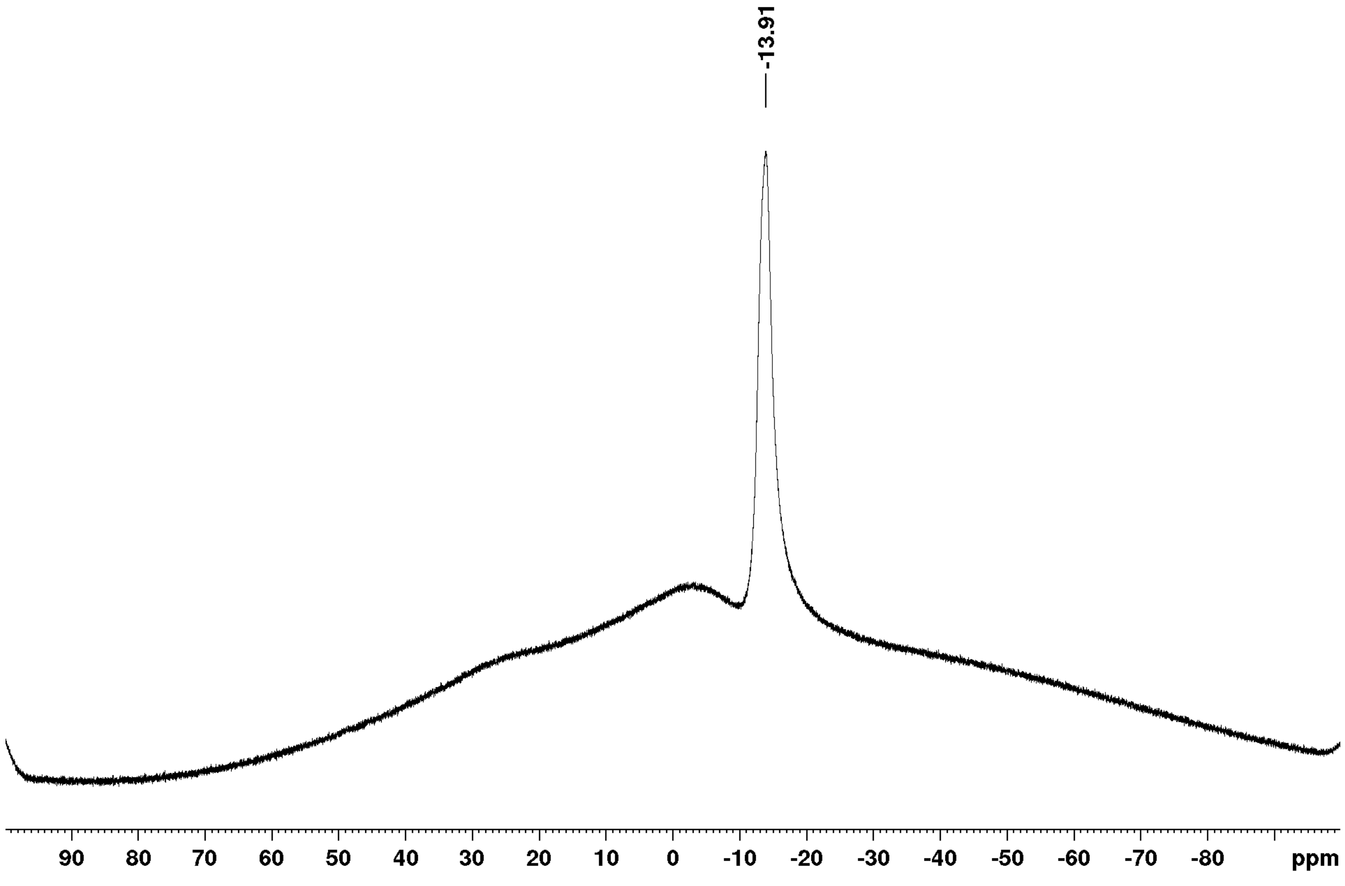

12-((3-methylbutanoyl)oxy)methyl-2-(3-(benzylamino)-3-oxoprop-1-yn-1-yl)-1-benzylaminocarboxyl-1,12-dicarba-*closo*-dodecaborane (Vj)

^{1}H NMR (500 MHz; $CDCl_3$)

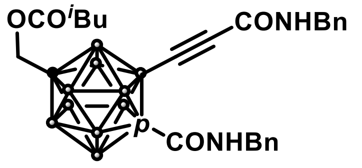

Vj

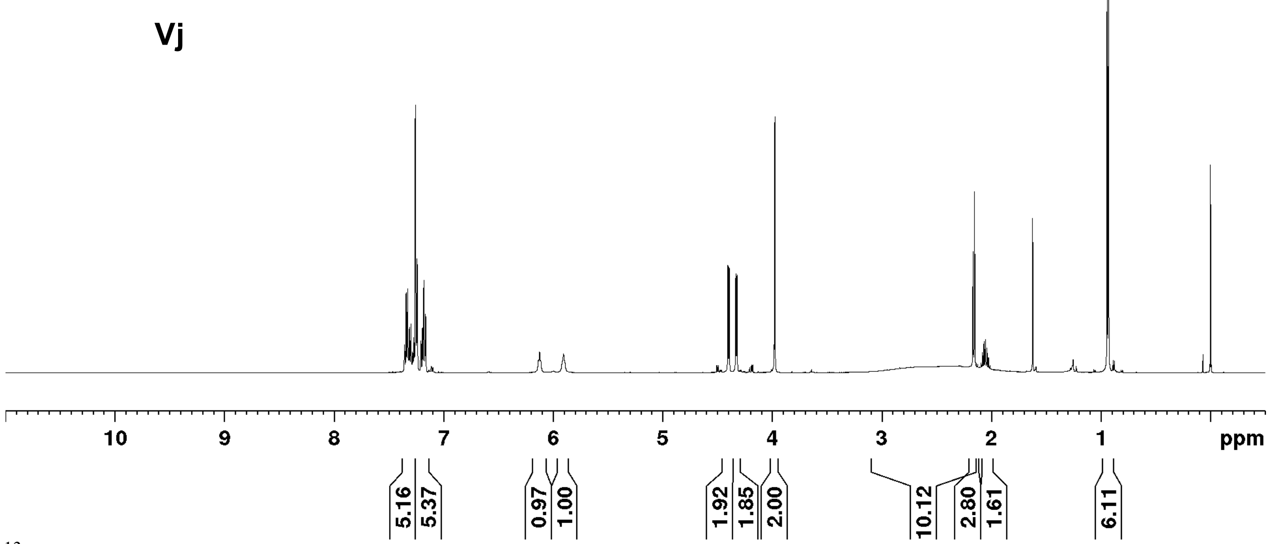

^{13}C NMR (125 MHz; $CDCl_3$)

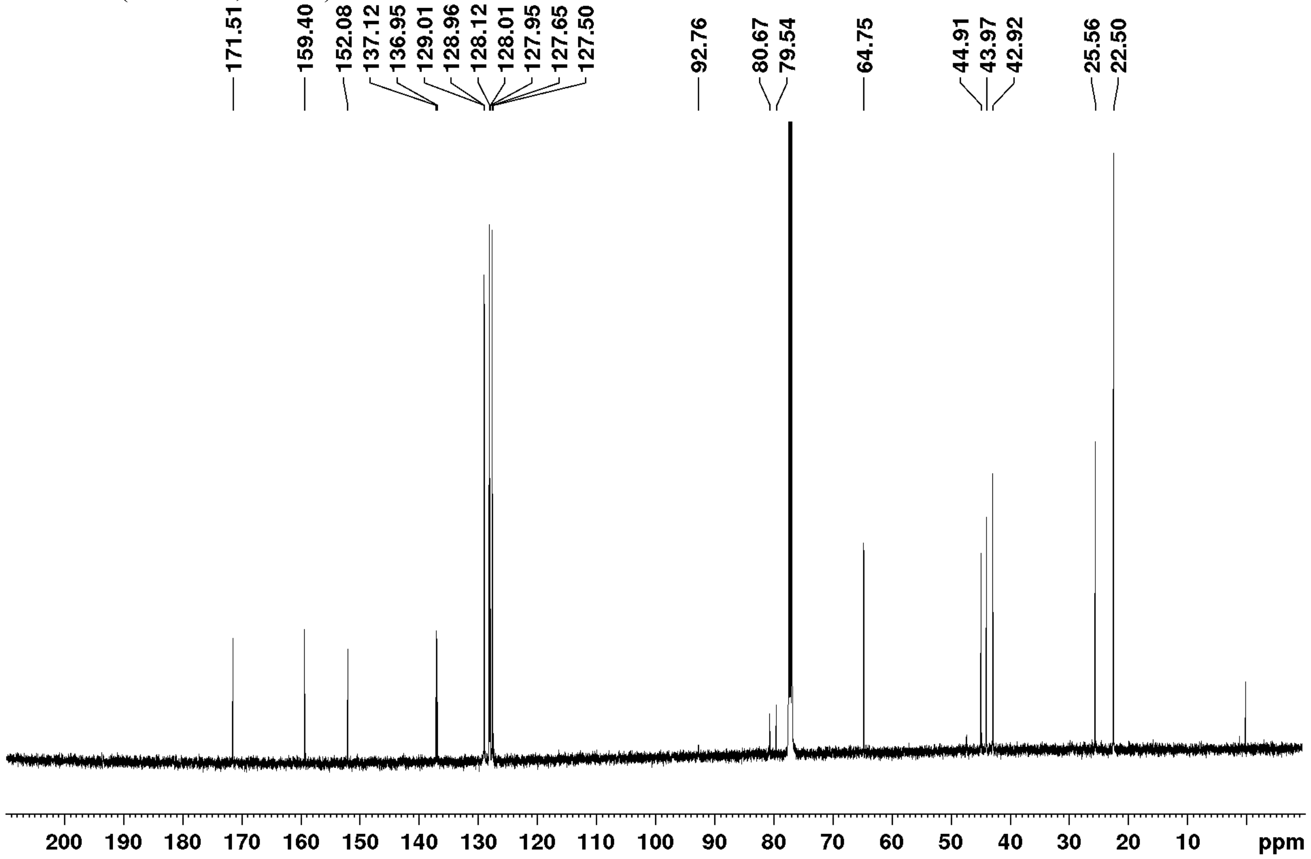

^{11}B NMR (160 MHz; $CDCl_3$)

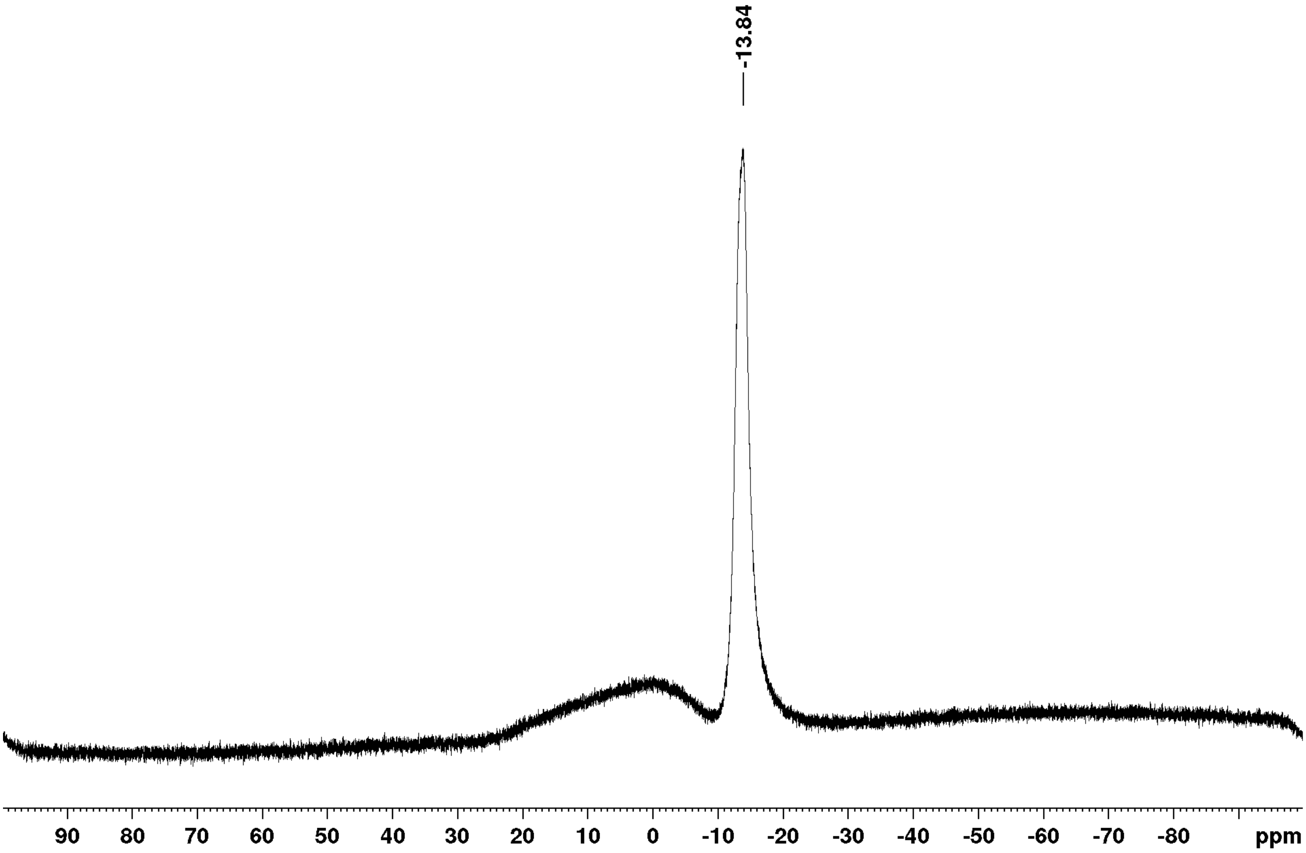

12-phenylacetoxymethyl-2-(3-(isobutylamino)-3-oxoprop-1-yn-1-yl)-1-benzylaminocarboxyl-1,12-dicarba-*closo*-dodecaborane (Vk)

^{1}H NMR (500 MHz; $CDCl_3$)

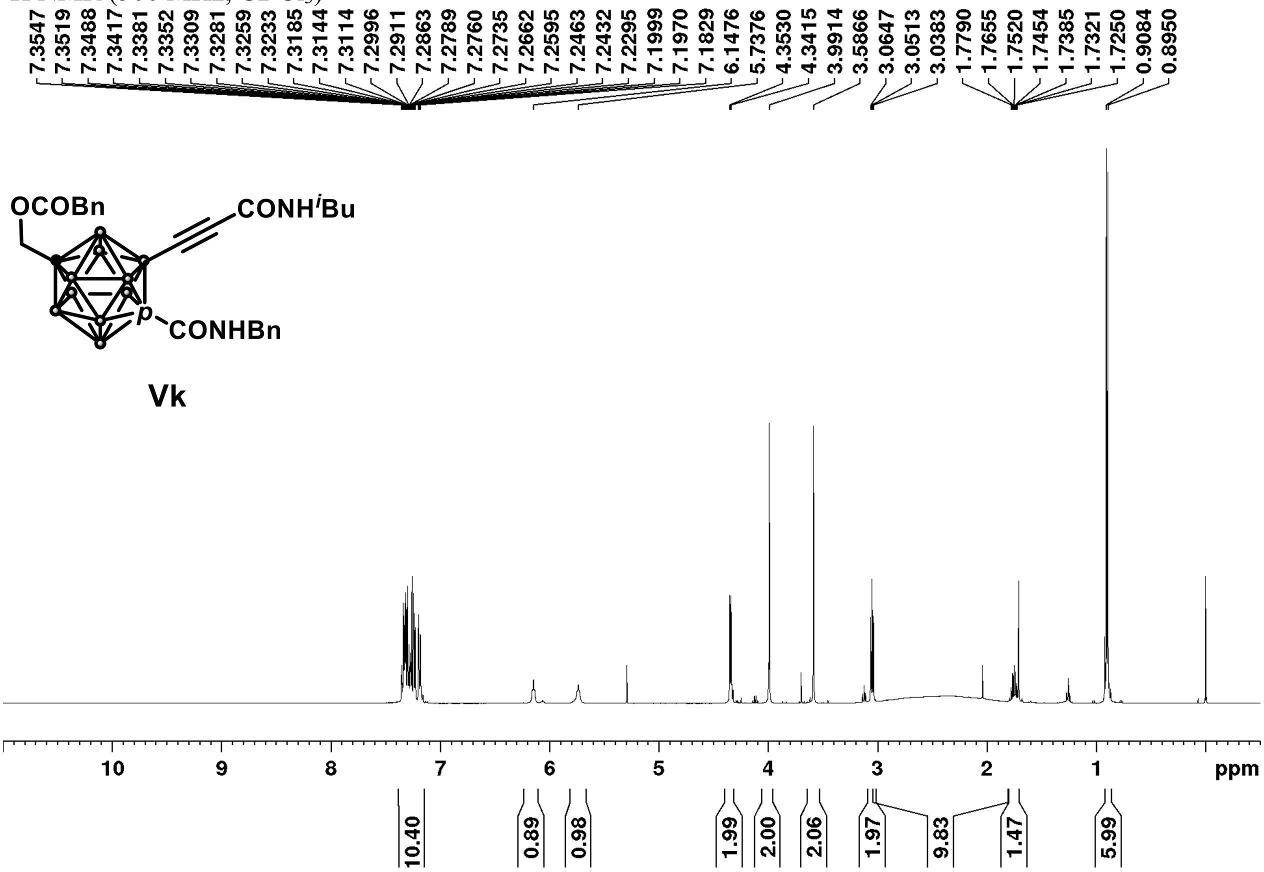

^{13}C NMR (125 MHz; $CDCl_3$)

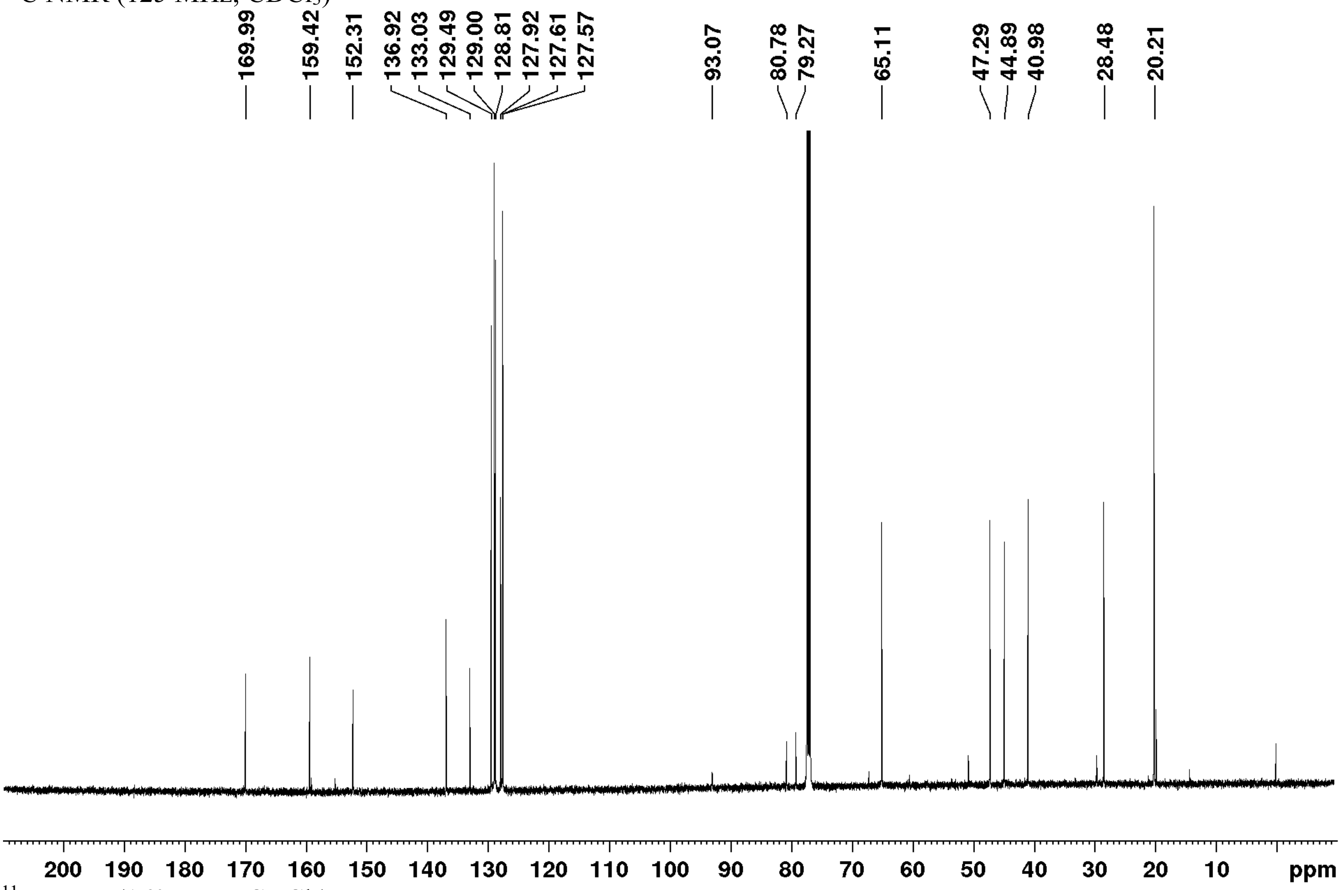

^{11}B NMR (160 MHz; $CDCl_3$)

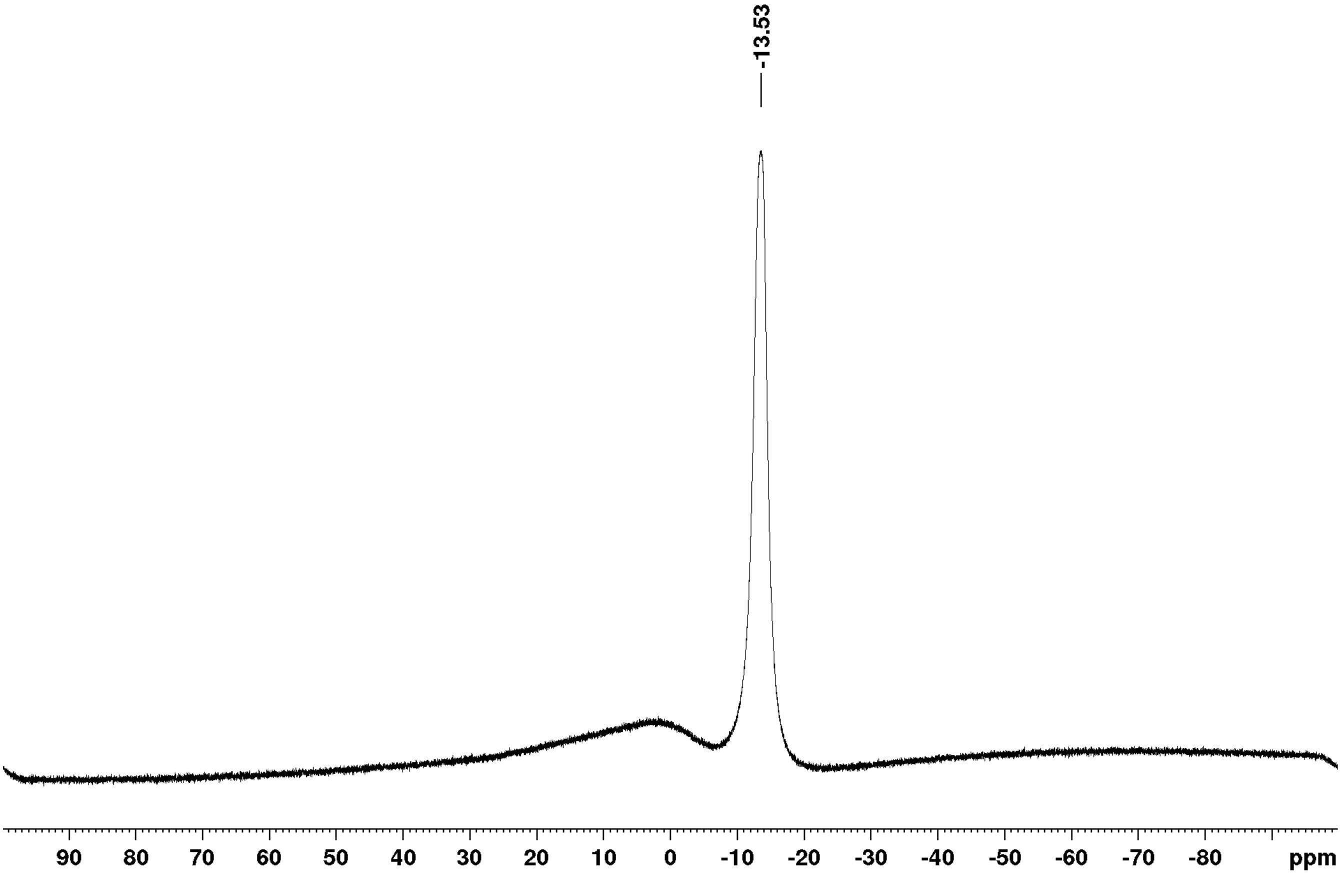

12-((3-methylbutanoyl)oxy)methyl-2-(3-(isobutylamino)-3-oxoprop-1-yn-1-yl)-1- benzylaminocarboxyl-1,12-dicarba-*closo*-dodecaborane (VI)

^{1}H NMR (500 MHz; $CDCl_3$)

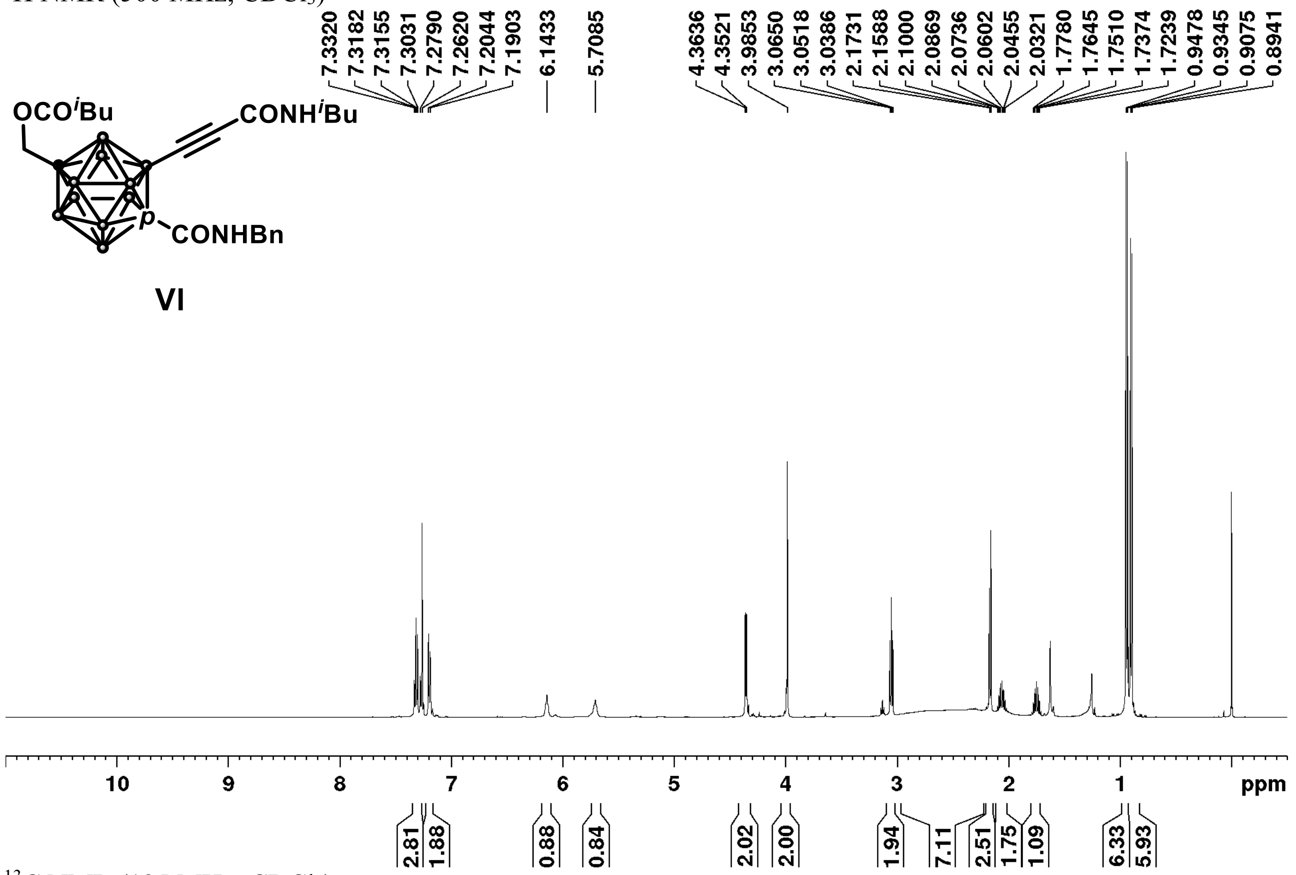

^{13}C NMR (125 MHz; $CDCl_3$)

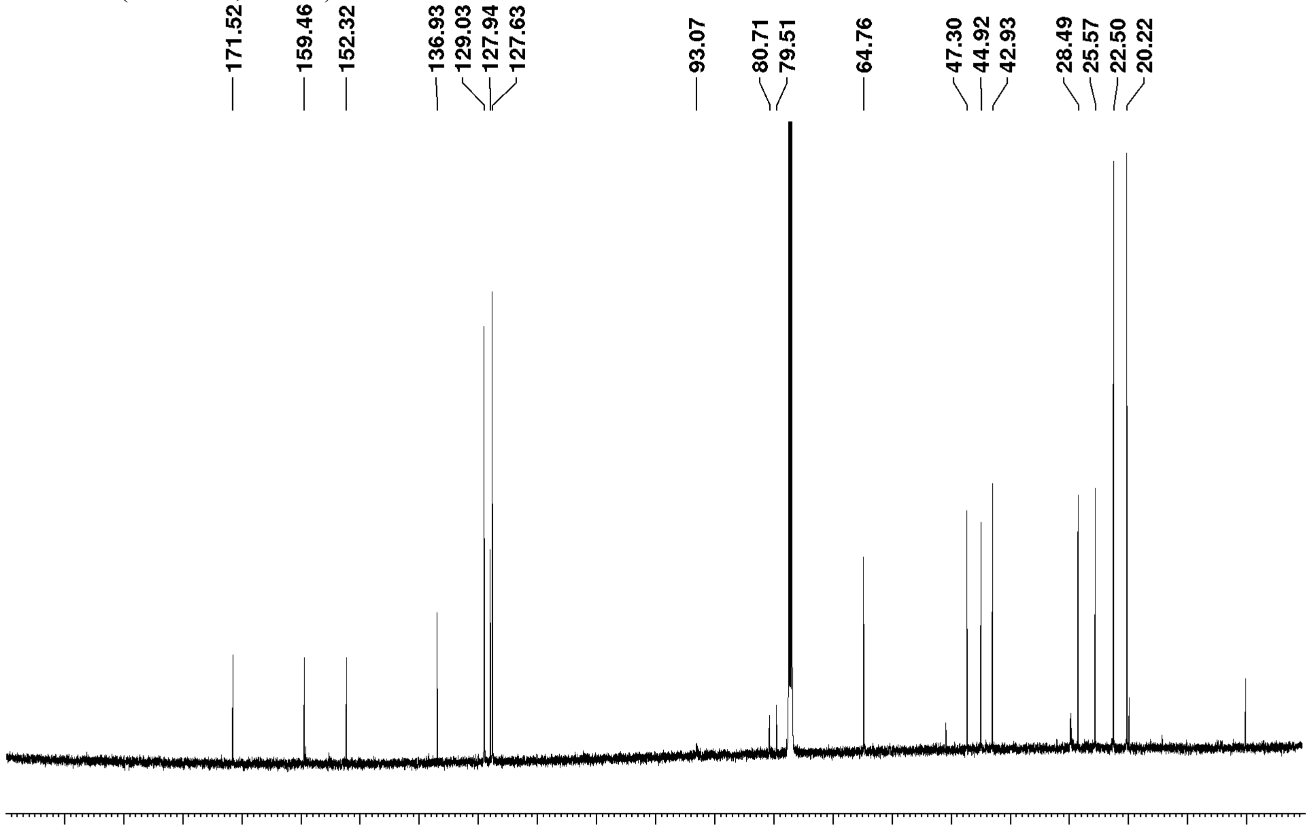

^{11}B NMR (160 MHz; $CDCl_3$)

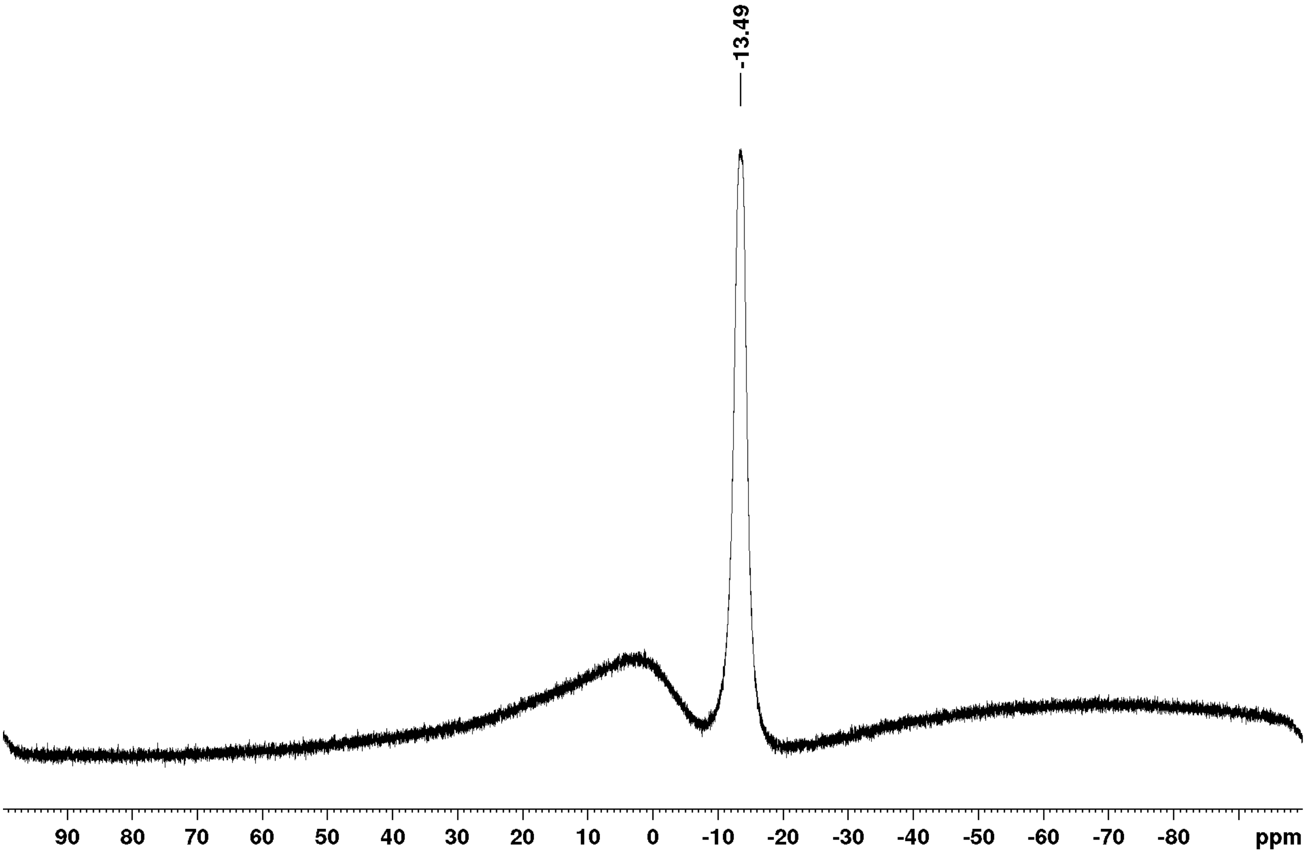

Synthesis of 12-phenylacetoxymethyl-2-(3-(benzylamino)-3-oxoprop-1-yn-1-yl)-1-isobutylcarbamoyl-1,12-dicarba-*closo*-dodecaborane (Vm)

^{1}H NMR (500 MHz; $CDCl_3$)

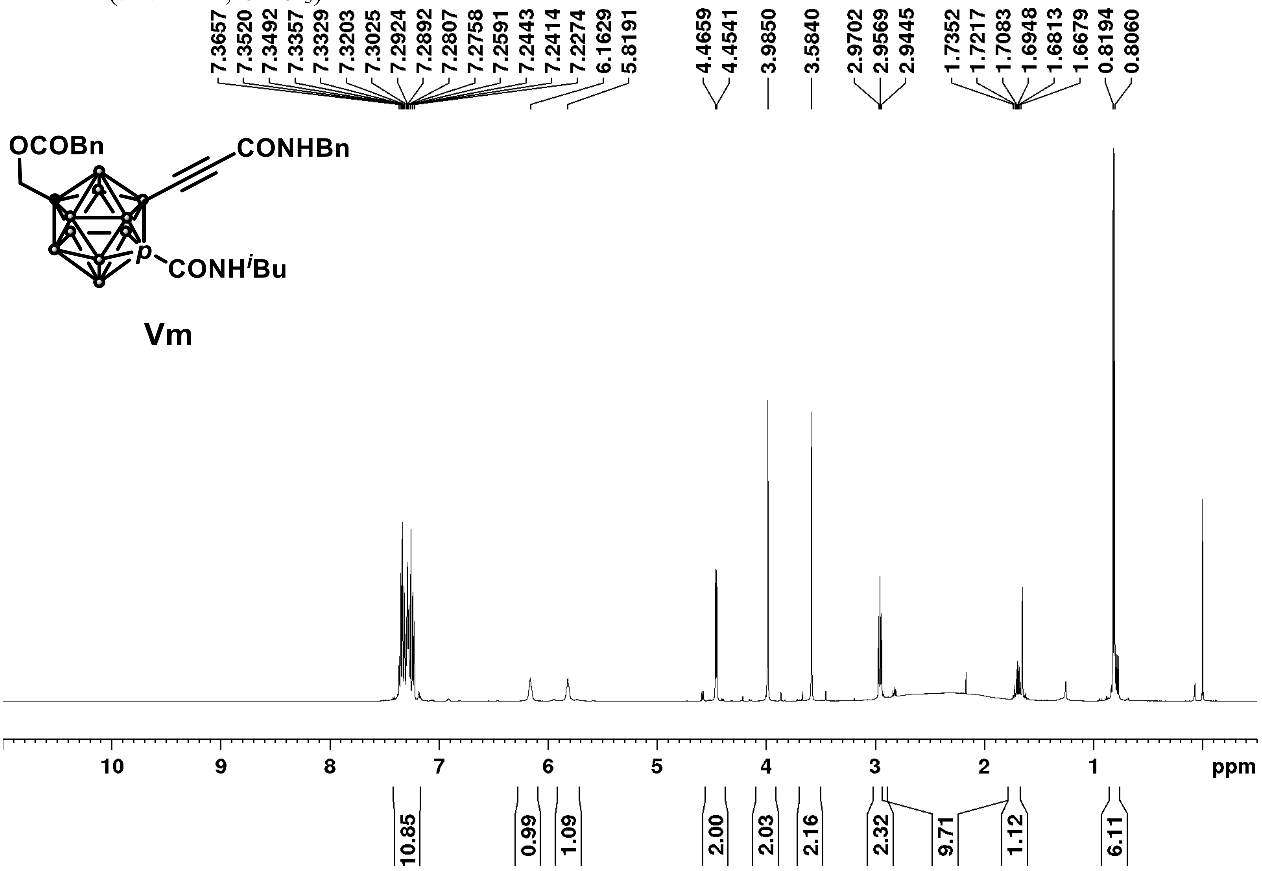

^{13}C NMR (125 MHz; $CDCl_3$)

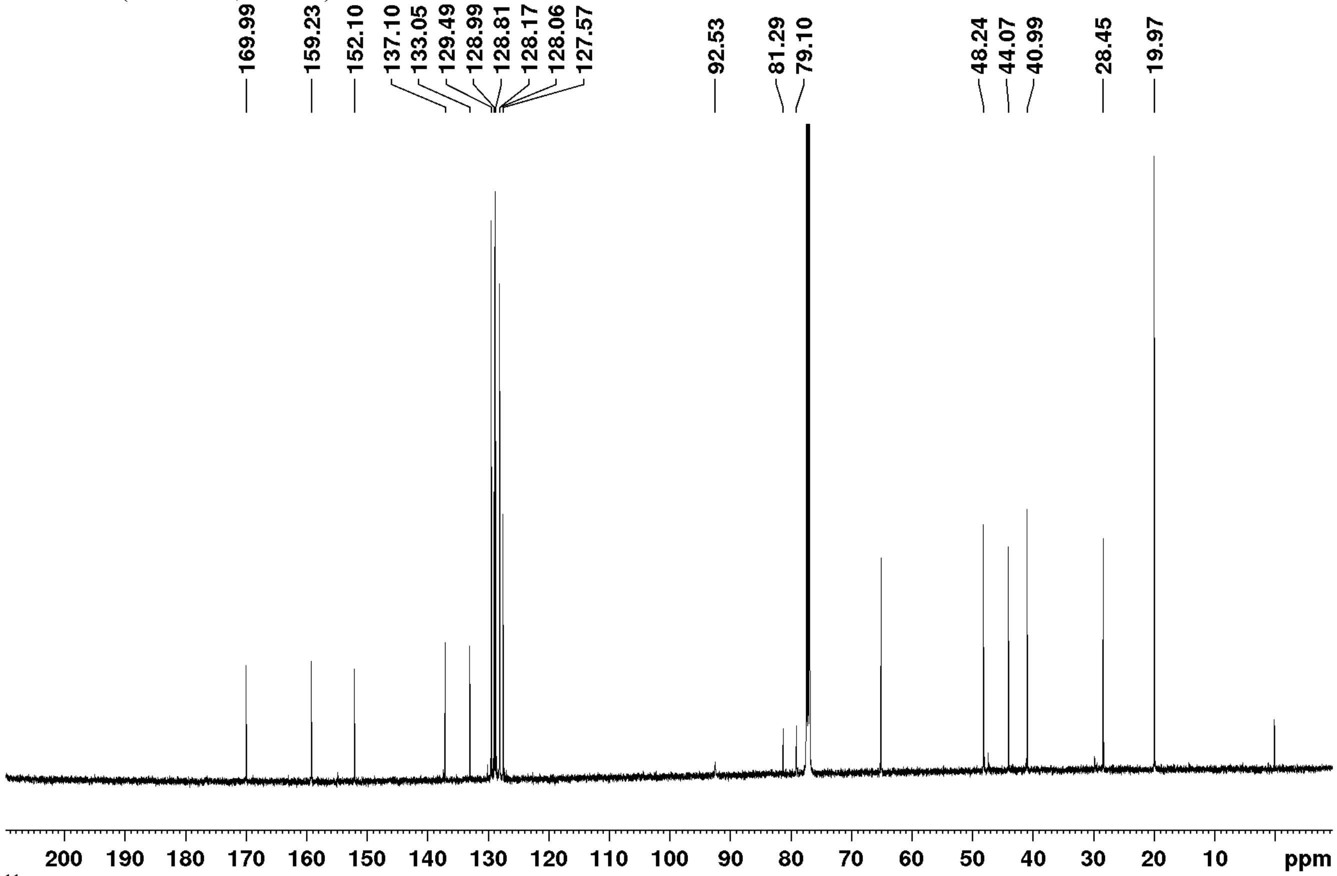

^{11}B NMR (160 MHz; $CDCl_3$)

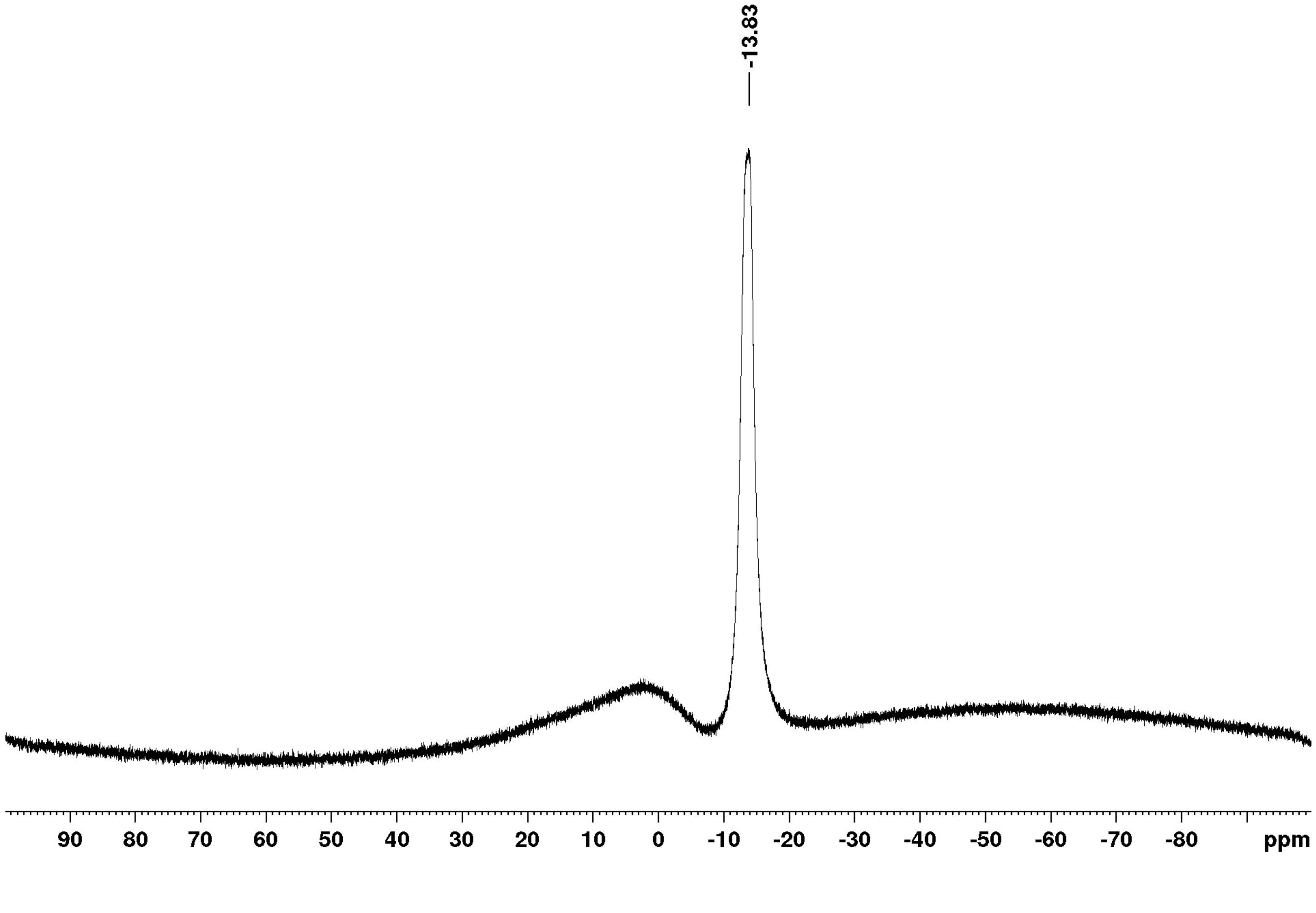

12-((3-methylbutanoyl)oxy)methyl-2-(3-(benzylamino)-3-oxoprop-1-yn-1-yl)-1-isobutylcarbamoyl-1,12-dicarba-*closo*-dodecaborane (Vn)

^{1}H NMR (500 MHz; $CDCl_3$)

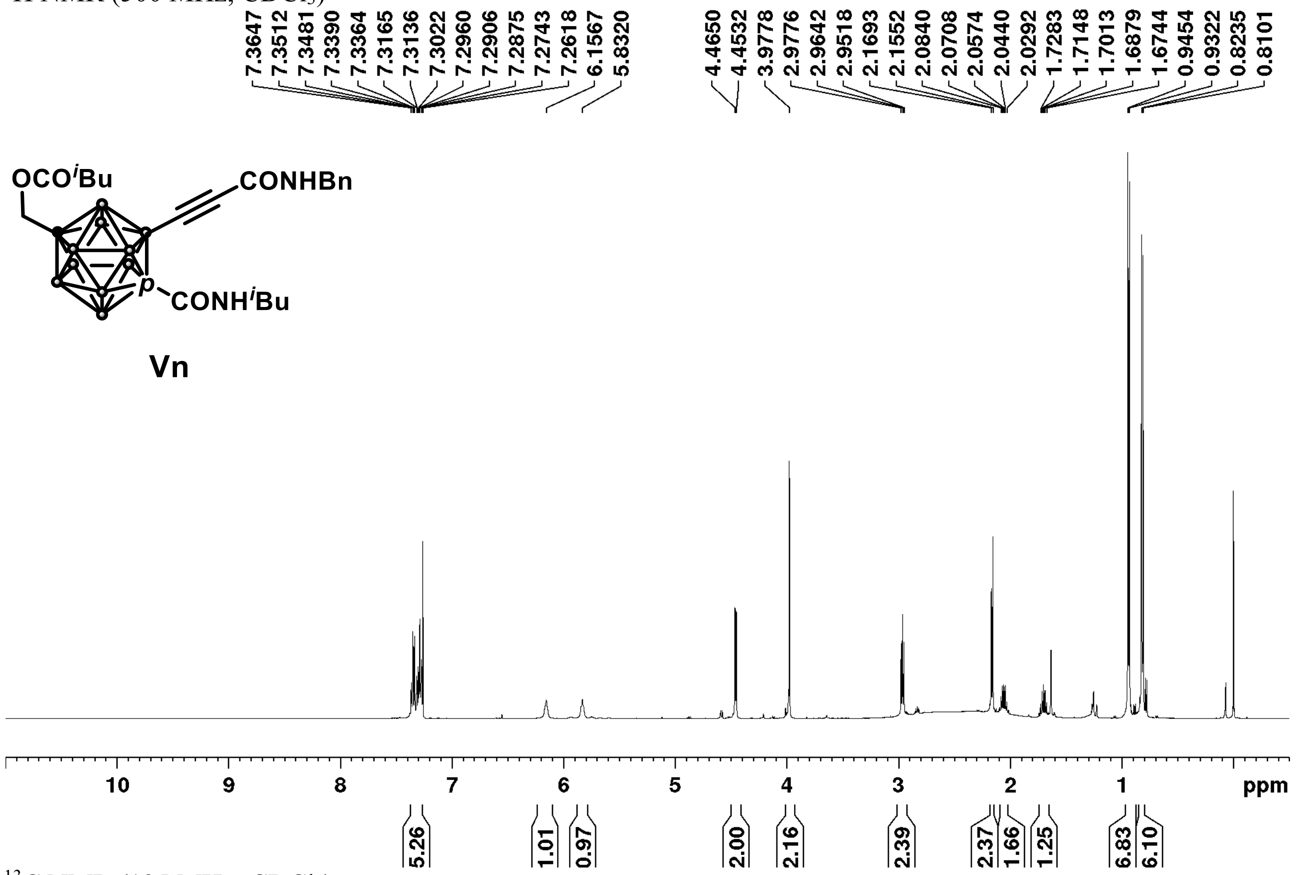

^{13}C NMR (125 MHz; $CDCl_3$)

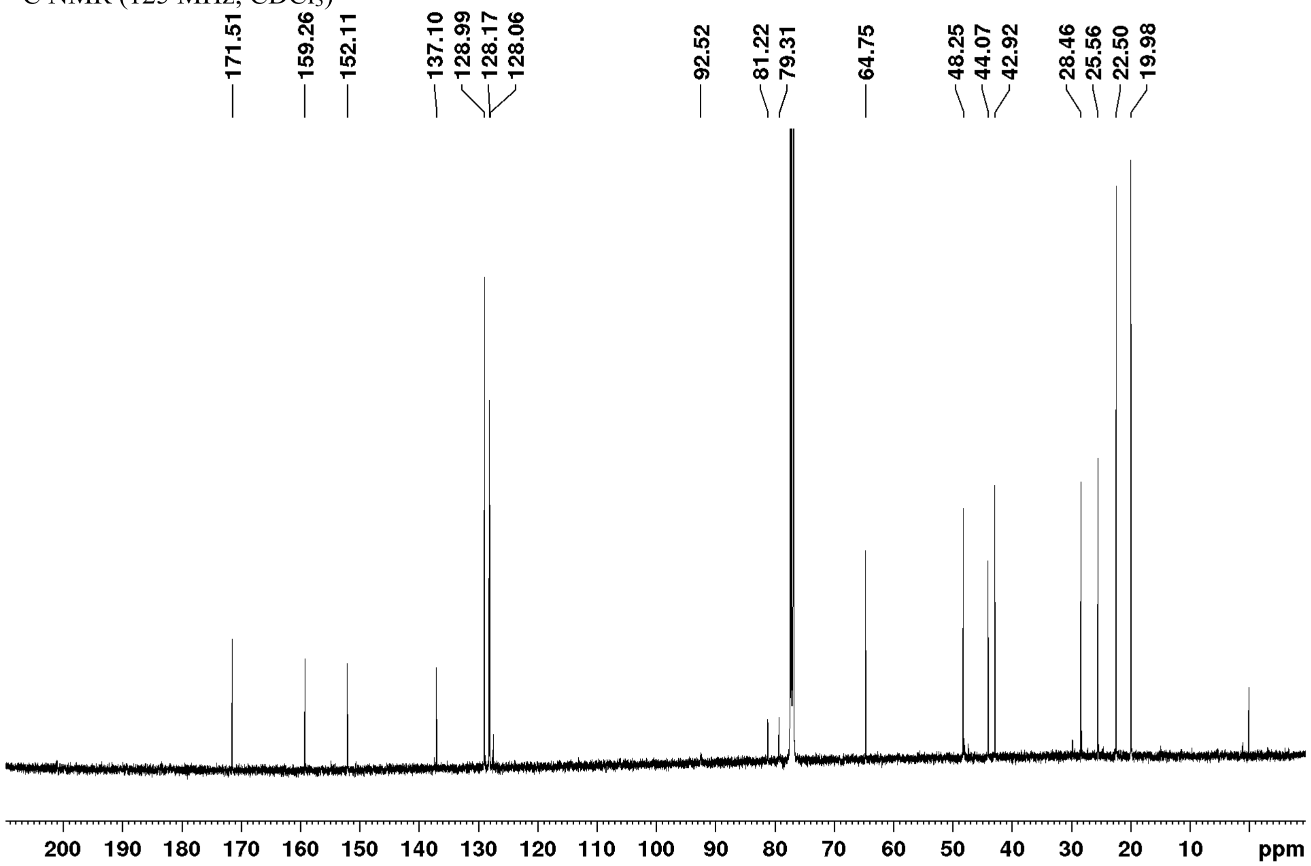

^{11}B NMR (160 MHz; $CDCl_3$)

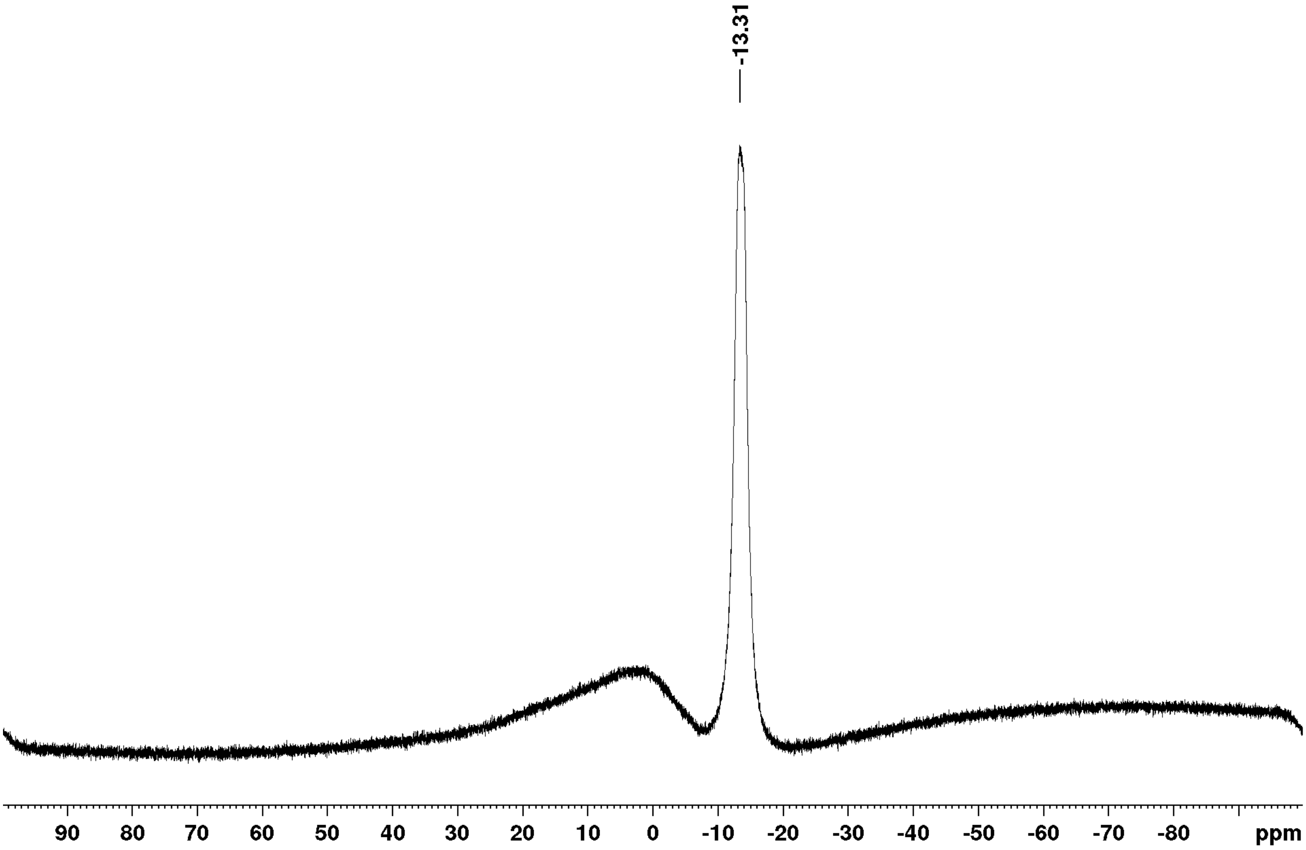

12-phenylacetoxymethyl-2-(3-(isobutylamino)-3-oxoprop-1-yn-1-yl)-1-isobutylcarbamoyl-1,12-dicarba-*closo*-dodecaborane (Vo)

^{1}H NMR (500 MHz; $CDCl_3$)

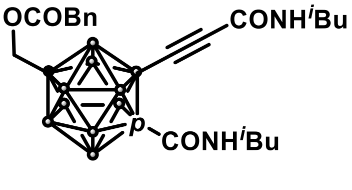

Vo

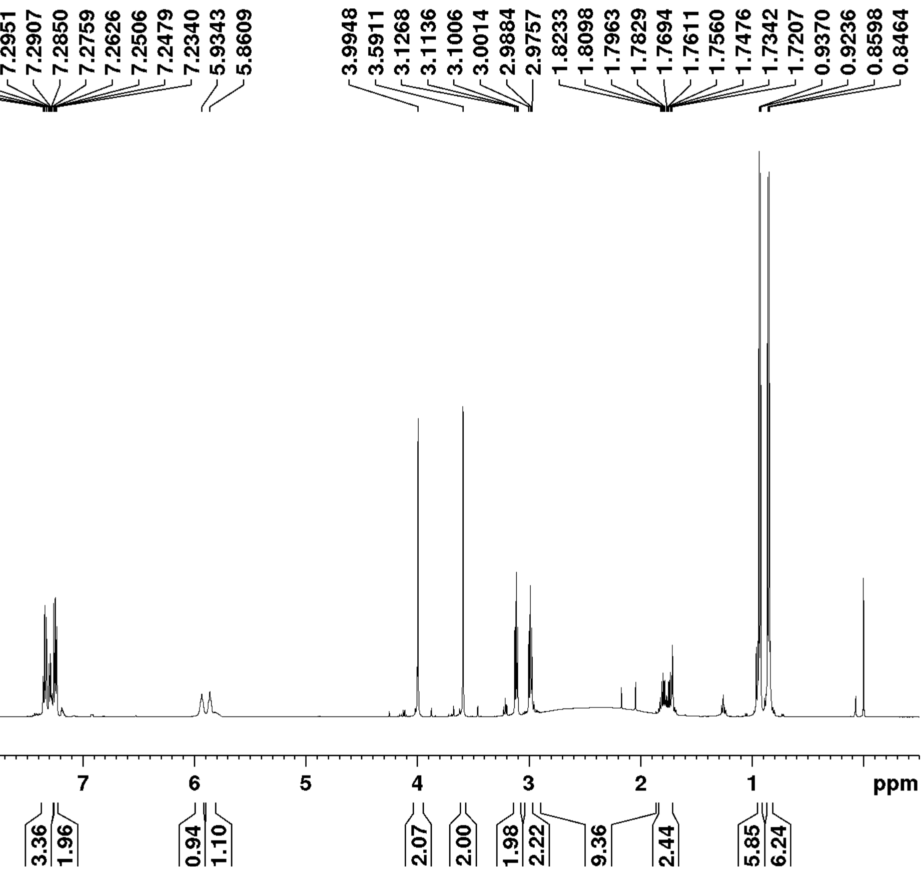

^{13}C NMR (125 MHz; $CDCl_3$)

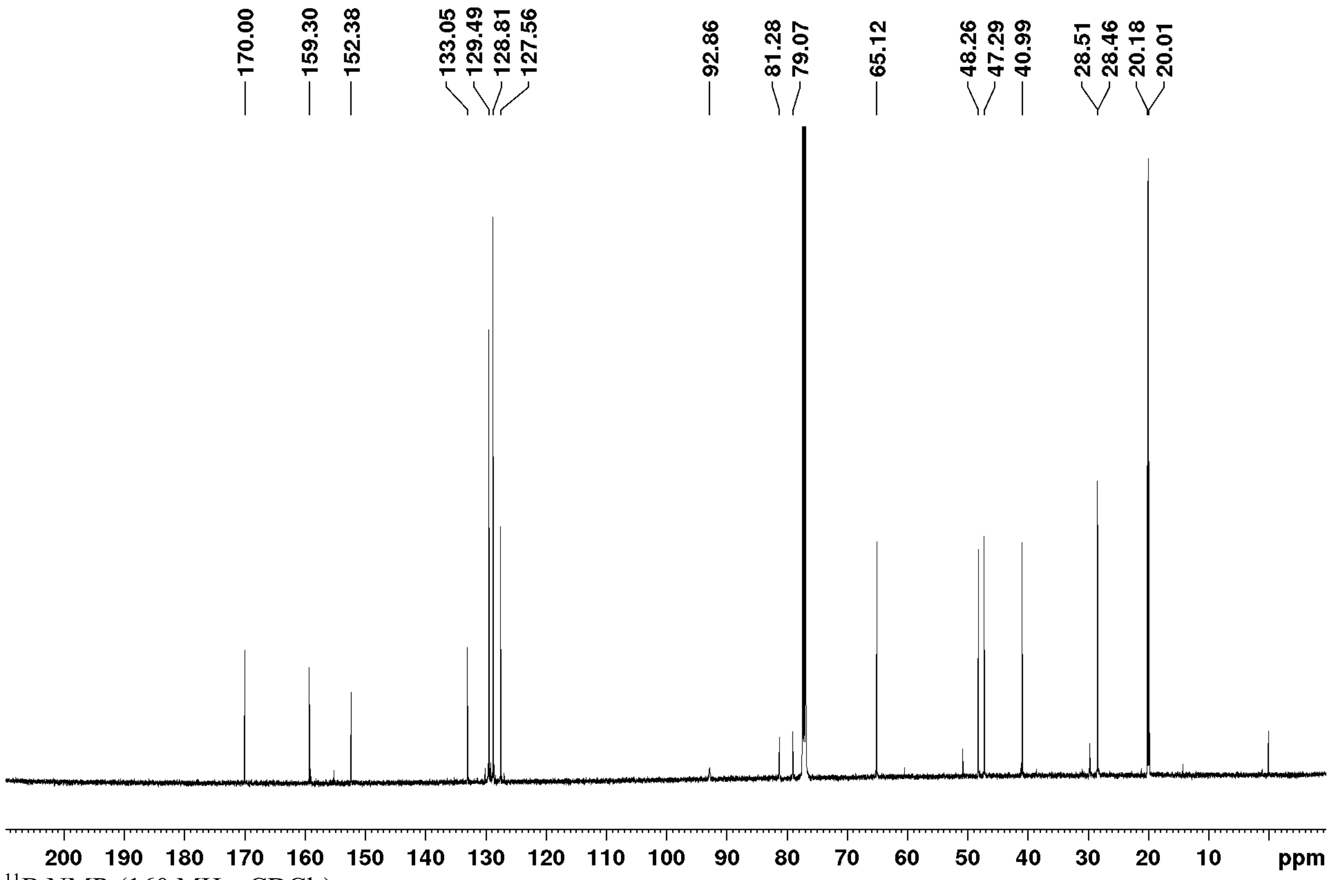

^{11}B NMR (160 MHz; $CDCl_3$)

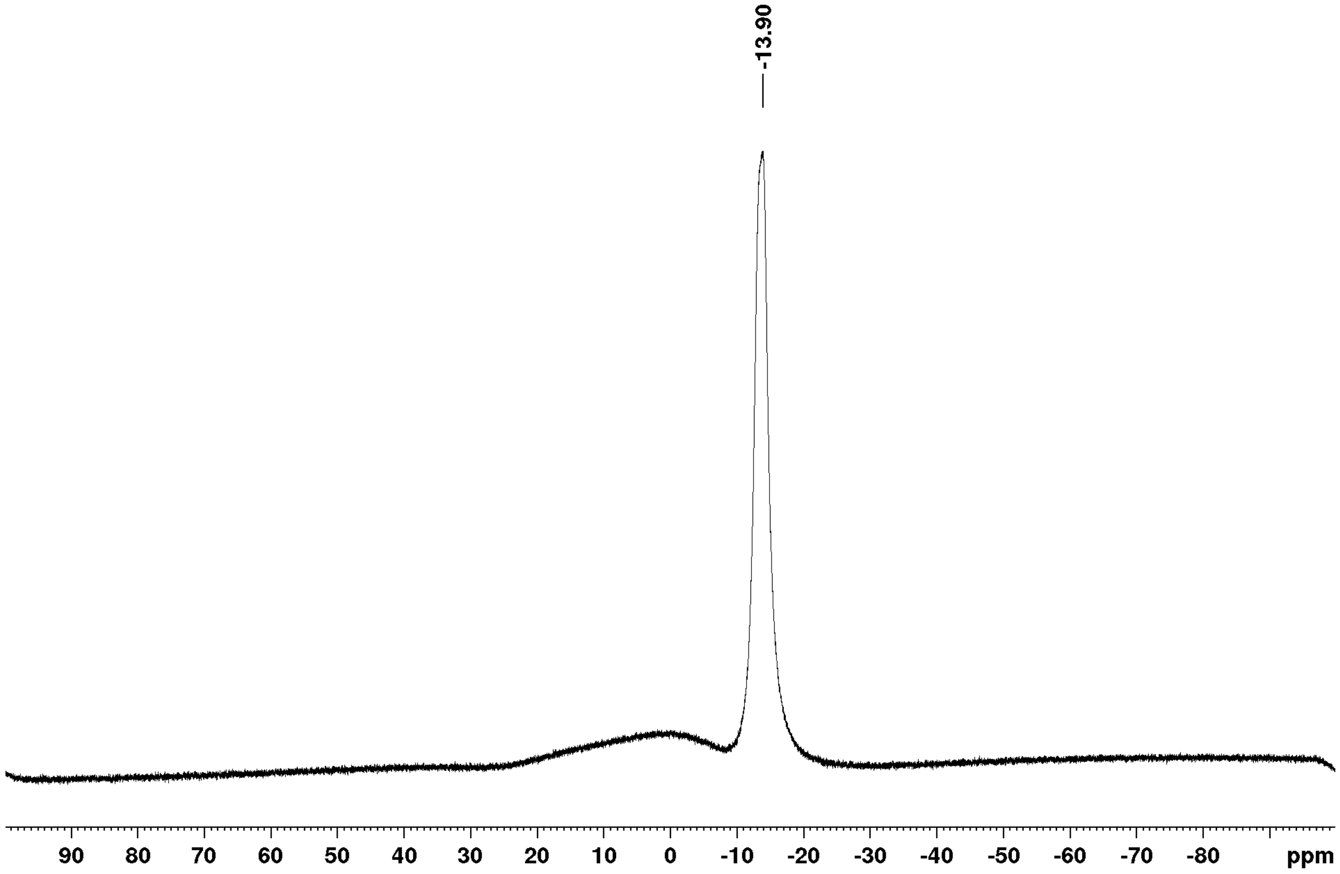

12-((3-methylbutanoyl)oxy)methyl-2-(3-(isobutylamino)-3-oxoprop-1-yn-1-yl)-1-isobutylcarbamoyl-1,12-dicarba-*closo*-dodecaborane (Vp)

^{1}H NMR (500 MHz; $CDCl_3$)

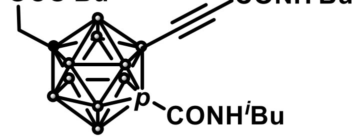

Vp

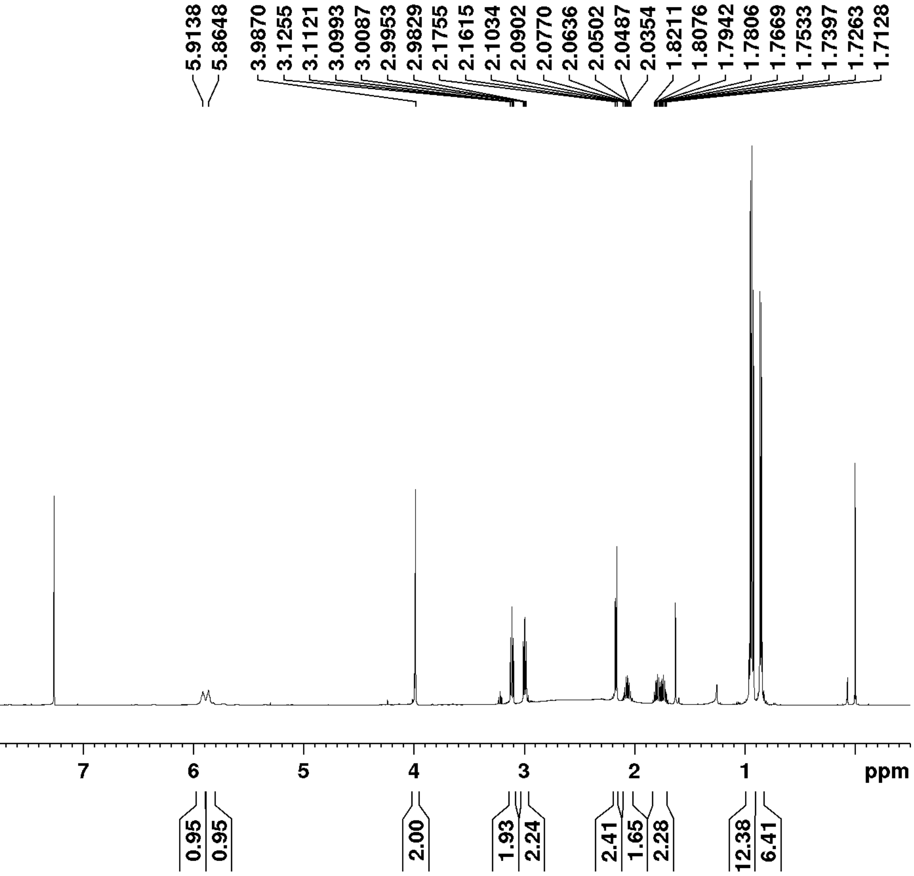

^{13}C NMR (125 MHz; $CDCl_3$)

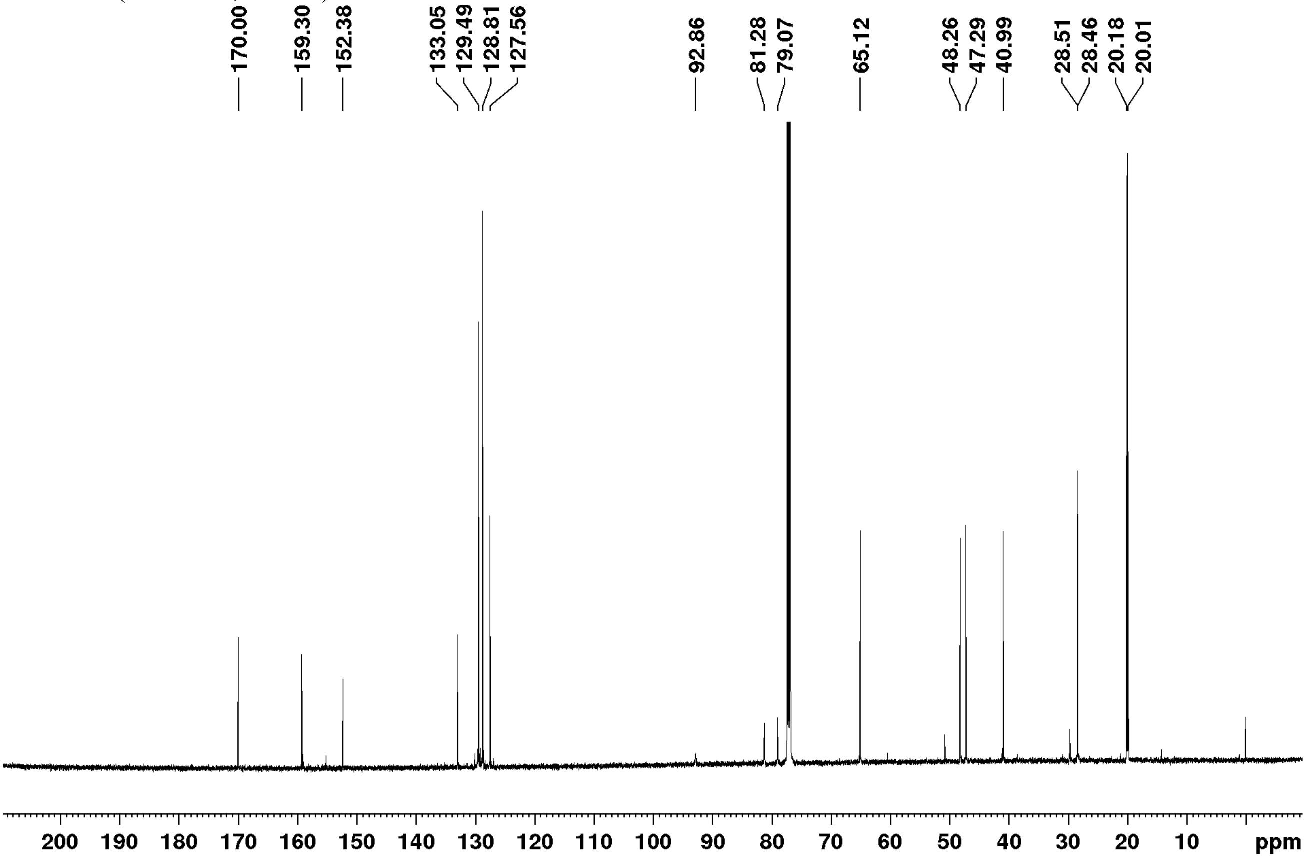

^{11}B NMR (160 MHz; $CDCl_3$)

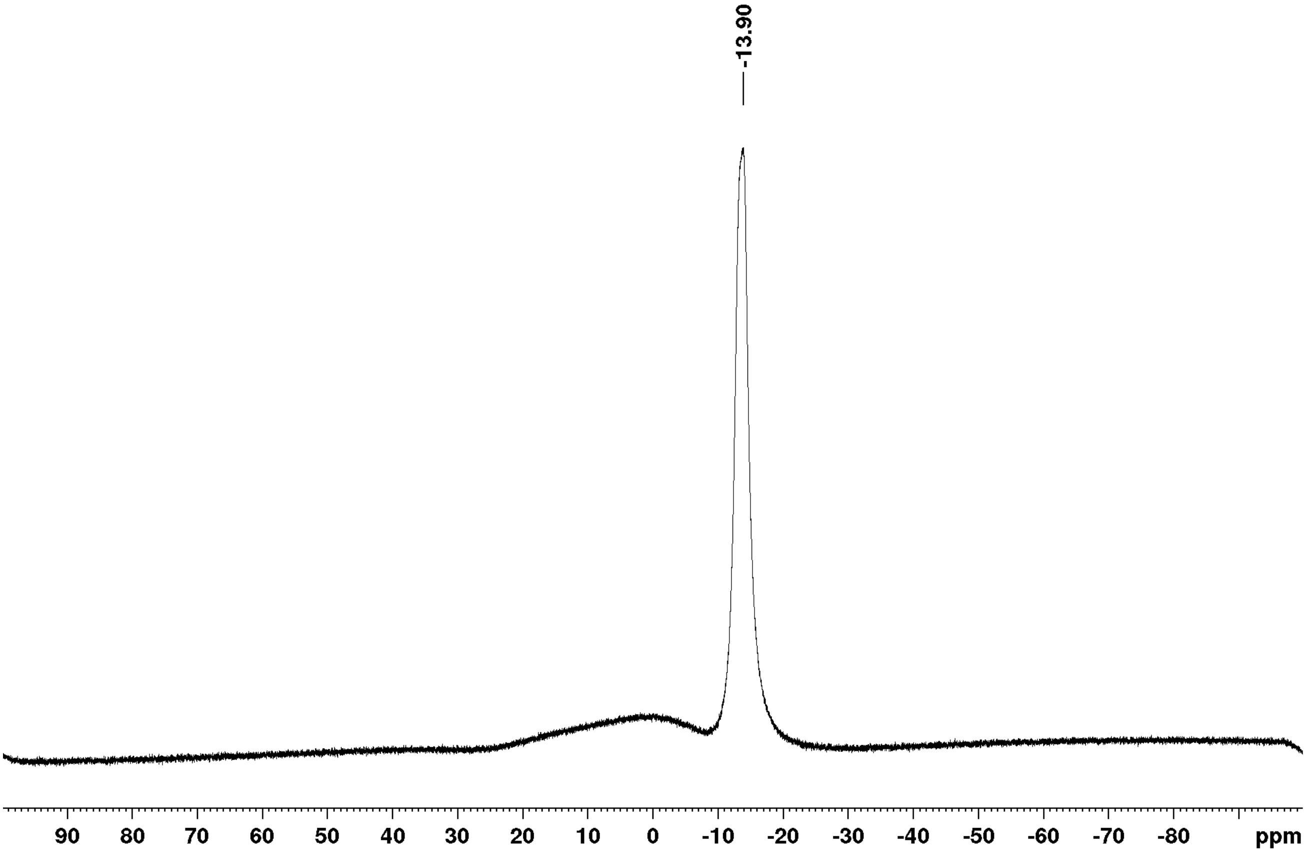

Supplement: Supplementary file 1 — Supplementary Information. [file 41598_2021_3459_MOESM1_ESM.pdf]
